# The global burden of cancer attributable to risk factors, 2010–19: a systematic analysis for the Global Burden of Disease Study 2019

**DOI:** 10.1016/S0140-6736(22)01438-6

**Published:** 2022-08-20

**Authors:** GBD 2019 Cancer Risk Factors Collaborators, Khanh Bao Tran, Justin J Lang, Kelly Compton, Rixing Xu, Alistair R Acheson, Hannah Jacqueline Henrikson, Jonathan M Kocarnik, Louise Penberthy, Amirali Aali, Qamar Abbas, Behzad Abbasi, Mohsen Abbasi-Kangevari, Zeinab Abbasi-Kangevari, Hedayat Abbastabar, Michael Abdelmasseh, Sherief Abd-Elsalam, Ahmed Abdelwahab Abdelwahab, Gholamreza Abdoli, Hanan Abdulkadir Abdulkadir, Aidin Abedi, Kedir Hussein Abegaz, Hassan Abidi, Richard Gyan Aboagye, Hassan Abolhassani, Abdorrahim Absalan, Yonas Derso Abtew, Hiwa Abubaker Ali, Eman Abu-Gharbieh, Basavaprabhu Achappa, Juan Manuel Acuna, Daniel Addison, Isaac Yeboah Addo, Oyelola A Adegboye, Miracle Ayomikun Adesina, Mohammad Adnan, Qorinah Estiningtyas Sakilah Adnani, Shailesh M Advani, Sumia Afrin, Muhammad Sohail Afzal, Manik Aggarwal, Bright Opoku Ahinkorah, Araz Ramazan Ahmad, Rizwan Ahmad, Sajjad Ahmad, Sohail Ahmad, Sepideh Ahmadi, Haroon Ahmed, Luai A Ahmed, Muktar Beshir Ahmed, Tarik Ahmed Rashid, Wajeeha Aiman, Marjan Ajami, Gizachew Taddesse Akalu, Mostafa Akbarzadeh-Khiavi, Addis Aklilu, Maxwell Akonde, Chisom Joyqueenet Akunna, Hanadi Al Hamad, Fares Alahdab, Fahad Mashhour Alanezi, Turki M Alanzi, Saleh Ali Alessy, Abdelazeem M Algammal, Mohammed Khaled Al-Hanawi, Robert Kaba Alhassan, Beriwan Abdulqadir Ali, Liaqat Ali, Syed Shujait Ali, Yousef Alimohamadi, Vahid Alipour, Syed Mohamed Aljunid, Motasem Alkhayyat, Sadeq Ali Ali Al-Maweri, Sami Almustanyir, Nivaldo Alonso, Shehabaldin Alqalyoobi, Rajaa M Al-Raddadi, Rami H Hani Al-Rifai, Salman Khalifah Al-Sabah, Ala'a B Al-Tammemi, Haya Altawalah, Nelson Alvis-Guzman, Firehiwot Amare, Edward Kwabena Ameyaw, Javad Javad Aminian Dehkordi, Mohammad Hosein Amirzade-Iranaq, Hubert Amu, Ganiyu Adeniyi Amusa, Robert Ancuceanu, Jason A Anderson, Yaregal Animut Animut, Amir Anoushiravani, Ali Arash Anoushirvani, Alireza Ansari-Moghaddam, Mustafa Geleto Ansha, Benny Antony, Maxwell Hubert Antwi, Sumadi Lukman Anwar, Razique Anwer, Anayochukwu Edward Anyasodor, Jalal Arabloo, Morteza Arab-Zozani, Olatunde Aremu, Ayele Mamo Argaw, Hany Ariffin, Timur Aripov, Muhammad Arshad, Al Artaman, Judie Arulappan, Raphael Taiwo Aruleba, Armin Aryannejad, Malke Asaad, Mulusew A Asemahagn, Zatollah Asemi, Mohammad Asghari-Jafarabadi, Tahira Ashraf, Reza Assadi, Mohammad Athar, Seyyed Shamsadin Athari, Maha Moh'd Wahbi Atout, Sameh Attia, Avinash Aujayeb, Marcel Ausloos, Leticia Avila-Burgos, Atalel Fentahun Awedew, Mamaru Ayenew Awoke, Tewachew Awoke, Beatriz Paulina Ayala Quintanilla, Tegegn Mulatu Ayana, Solomon Shitu Ayen, Davood Azadi, Sina Azadnajafabad, Saber Azami-Aghdash, Melkalem Mamuye Azanaw, Mohammadreza Azangou-Khyavy, Amirhossein Azari Jafari, Hosein Azizi, Ahmed Y Y Azzam, Amirhesam Babajani, Muhammad Badar, Ashish D Badiye, Nayereh Baghcheghi, Nader Bagheri, Sara Bagherieh, Saeed Bahadory, Atif Amin Baig, Jennifer L Baker, Ahad Bakhtiari, Ravleen Kaur Bakshi, Maciej Banach, Indrajit Banerjee, Mainak Bardhan, Francesco Barone-Adesi, Fabio Barra, Amadou Barrow, Nasir Z Bashir, Azadeh Bashiri, Saurav Basu, Abdul-Monim Mohammad Batiha, Aeysha Begum, Alehegn Bekele Bekele, Alemayehu Sayih Belay, Melaku Ashagrie Belete, Uzma Iqbal Belgaumi, Arielle Wilder Bell, Luis Belo, Habib Benzian, Alemshet Yirga Berhie, Amiel Nazer C Bermudez, Eduardo Bernabe, Akshaya Srikanth Bhagavathula, Neeraj Bhala, Bharti Bhandari Bhandari, Nikha Bhardwaj, Pankaj Bhardwaj, Krittika Bhattacharyya, Vijayalakshmi S Bhojaraja, Soumitra S Bhuyan, Sadia Bibi, Awraris Hailu Bilchut, Bagas Suryo Bintoro, Antonio Biondi, Mesfin Geremaw Birega Birega, Habitu Eshetu Birhan, Tone Bjørge, Oleg Blyuss, Belay Boda Abule Bodicha, Srinivasa Rao Bolla, Archith Boloor, Cristina Bosetti, Dejana Braithwaite, Michael Brauer, Hermann Brenner, Andrey Nikolaevich Briko, Nikolay Ivanovich Briko, Christina Maree Buchanan, Norma B Bulamu, Maria Teresa Bustamante-Teixeira, Muhammad Hammad Butt, Nadeem Shafique Butt, Zahid A Butt, Florentino Luciano Caetano dos Santos, Luis Alberto Cámera, Chao Cao, Yin Cao, Giulia Carreras, Márcia Carvalho, Francieli Cembranel, Ester Cerin, Promit Ananyo Chakraborty, Periklis Charalampous, Vijay Kumar Chattu, Odgerel Chimed-Ochir, Jesus Lorenzo Chirinos-Caceres, Daniel Youngwhan Cho, William C S Cho, Devasahayam J Christopher, Dinh-Toi Chu, Isaac Sunday Chukwu, Aaron J Cohen, Joao Conde, Sandra Cortés, Vera Marisa Costa, Natália Cruz-Martins, Garland T Culbreth, Omid Dadras, Fentaw Teshome Dagnaw, Saad M A Dahlawi, Xiaochen Dai, Lalit Dandona, Rakhi Dandona, Parnaz Daneshpajouhnejad, Anna Danielewicz, An Thi Minh Dao, Reza Darvishi Cheshmeh Soltani, Aso Mohammad Darwesh, Saswati Das, Dragos Virgil Davitoiu, Elham Davtalab Esmaeili, Fernando Pio De la Hoz, Sisay Abebe Debela, Azizallah Dehghan, Biniyam Demisse, Fitsum Wolde Demisse, Edgar Denova-Gutiérrez, Afshin Derakhshani, Meseret Derbew Molla, Diriba Dereje, Kalkidan Solomon Deribe, Rupak Desai, Markos Desalegn Desalegn, Fikadu Nugusu Dessalegn, Samuel Abebe A Dessalegni, Gashaw Dessie, Abebaw Alemayehu Desta, Syed Masudur Rahman Dewan, Samath Dhamminda Dharmaratne, Meghnath Dhimal, Mostafa Dianatinasab, Nancy Diao, Daniel Diaz, Lankamo Ena Digesa, Shilpi Gupta Dixit, Saeid Doaei, Linh Phuong Doan, Paul Narh Doku, Deepa Dongarwar, Wendel Mombaque dos Santos, Tim Robert Driscoll, Haneil Larson Dsouza, Oyewole Christopher Durojaiye, Sareh Edalati, Fatemeh Eghbalian, Elham Ehsani-Chimeh, Ebrahim Eini, Michael Ekholuenetale, Temitope Cyrus Ekundayo, Donatus U Ekwueme, Maha El Tantawi, Mostafa Ahmed Elbahnasawy, Iffat Elbarazi, Hesham Elghazaly, Muhammed Elhadi, Waseem El-Huneidi, Mohammad Hassan Emamian, Luchuo Engelbert Bain, Daniel Berhanie Enyew, Ryenchindorj Erkhembayar, Tegegne Eshetu, Babak Eshrati, Sharareh Eskandarieh, Juan Espinosa-Montero, Farshid Etaee, Azin Etemadimanesh, Tahir Eyayu, Ifeanyi Jude Ezeonwumelu, Sayeh Ezzikouri, Adeniyi Francis Fagbamigbe, Saman Fahimi, Ildar Ravisovich Fakhradiyev, Emerito Jose A Faraon, Jawad Fares, Abbas Farmany, Umar Farooque, Hossein Farrokhpour, Abidemi Omolara Fasanmi, Ali Fatehizadeh, Wafa Fatima, Hamed Fattahi, Ginenus Fekadu, Berhanu Elfu Feleke, Allegra Allegra Ferrari, Simone Ferrero, Lorenzo Ferro Desideri, Irina Filip, Florian Fischer, Roham Foroumadi, Masoud Foroutan, Takeshi Fukumoto, Peter Andras Gaal, Mohamed M Gad, Muktar A Gadanya, Abduzhappar Gaipov, Nasrin Galehdar, Silvano Gallus, Tushar Garg, Mariana Gaspar Fonseca, Yosef Haile Gebremariam, Teferi Gebru Gebremeskel, Mathewos Alemu Gebremichael, Yohannes Fikadu Geda, Yibeltal Yismaw Gela, Belete Negese Belete Gemeda, Melaku Getachew, Motuma Erena Getachew, Kazem Ghaffari, Mansour Ghafourifard, Seyyed-Hadi Ghamari, Mohammad Ghasemi Nour, Fariba Ghassemi, Ajnish Ghimire, Nermin Ghith, Maryam Gholamalizadeh, Jamshid Gholizadeh Navashenaq, Sherief Ghozy, Syed Amir Gilani, Paramjit Singh Gill, Themba G Ginindza, Abraham Tamirat T Gizaw, James C Glasbey, Justyna Godos, Amit Goel, Mahaveer Golechha, Pouya Goleij, Davide Golinelli, Mohamad Golitaleb, Giuseppe Gorini, Bárbara Niegia Garcia Goulart, Giuseppe Grosso, Habtamu Alganeh Guadie, Mohammed Ibrahim Mohialdeen Gubari, Temesgen Worku Gudayu, Maximiliano Ribeiro Guerra, Damitha Asanga Gunawardane, Bhawna Gupta, Sapna Gupta, Veer Bala Gupta, Vivek Kumar Gupta, Mekdes Kondale Gurara, Alemu Guta, Parham Habibzadeh, Atlas Haddadi Avval, Nima Hafezi-Nejad, Adel Hajj Ali, Arvin Haj-Mirzaian, Esam S Halboub, Aram Halimi, Rabih Halwani, Randah R Hamadeh, Sajid Hameed, Samer Hamidi, Asif Hanif, Sanam Hariri, Netanja I Harlianto, Josep Maria Haro, Risky Kusuma Hartono, Ahmed I Hasaballah, S M Mahmudul Hasan, Hamidreza Hasani, Seyedeh Melika Hashemi, Abbas M Hassan, Soheil Hassanipour, Khezar Hayat, Golnaz Heidari, Mohammad Heidari, Zahra Heidarymeybodi, Brenda Yuliana Herrera-Serna, Claudiu Herteliu, Kamal Hezam, Yuta Hiraike, Mbuzeleni Mbuzeleni Hlongwa, Ramesh Holla, Marianne Holm, Nobuyuki Horita, Mohammad Hoseini, Md Mahbub Hossain, Mohammad Bellal Hossain Hossain, Mohammad-Salar Hosseini, Ali Hosseinzadeh, Mehdi Hosseinzadeh, Mihaela Hostiuc, Sorin Hostiuc, Mowafa Househ, Junjie Huang, Fernando N Hugo, Ayesha Humayun, Salman Hussain, Nawfal R Hussein, Bing-Fang Hwang, Segun Emmanuel Ibitoye, Pulwasha Maria Iftikhar, Kevin S Ikuta, Olayinka Stephen Ilesanmi, Irena M Ilic, Milena D Ilic, Mustapha Immurana, Kaire Innos, Pooya Iranpour, Lalu Muhammad Irham, Md Shariful Islam, Rakibul M Islam, Farhad Islami, Nahlah Elkudssiah Ismail, Gaetano Isola, Masao Iwagami, Linda Merin J, Abhishek Jaiswal, Mihajlo Jakovljevic, Mahsa Jalili, Shahram Jalilian, Elham Jamshidi, Sung-In Jang, Chinmay T Jani, Tahereh Javaheri, Umesh Umesh Jayarajah, Shubha Jayaram, Seyed Behzad Jazayeri, Rime Jebai, Bedru Jemal, Wonjeong Jeong, Ravi Prakash Jha, Har Ashish Jindal, Yetunde O John-Akinola, Jost B Jonas, Tamas Joo, Nitin Joseph, Farahnaz Joukar, Jacek Jerzy Jozwiak, Mikk Jürisson, Ali Kabir, Salah Eddine Oussama Kacimi, Vidya Kadashetti, Farima Kahe, Pradnya Vishal Kakodkar, Laleh R Kalankesh, Leila R Kalankesh, Rohollah Kalhor, Vineet Kumar Kamal, Farin Kamangar, Ashwin Kamath, Tanuj Kanchan, Eswar Kandaswamy, Himal Kandel, HyeJung Kang, Girum Gebremeskel Kanno, Neeti Kapoor, Sitanshu Sekhar Kar, Shama D Karanth, Ibraheem M Karaye, André Karch, Amirali Karimi, Bekalu Getnet Kassa, Patrick DMC Katoto, Joonas H Kauppila, Harkiran Kaur, Abinet Gebremickael Kebede, Leila Keikavoosi-Arani, Gemechu Gemechu Kejela, Phillip M Kemp Bohan, Maryam Keramati, Mohammad Keykhaei, Himanshu Khajuria, Abbas Khan, Abdul Aziz Khan Khan, Ejaz Ahmad Khan, Gulfaraz Khan, Md Nuruzzaman Khan, Moien AB Khan, Javad Khanali, Khaled Khatab, Moawiah Mohammad Khatatbeh, Mahalaqua Nazli Khatib, Maryam Khayamzadeh, Hamid Reza Khayat Kashani, Mohammad Amin Khazeei Tabari, Mehdi Khezeli, Mahmoud Khodadost, Min Seo Kim, Yun Jin Kim, Adnan Kisa, Sezer Kisa, Miloslav Klugar, Jitka Klugarová, Ali-Asghar Kolahi, Pavel Kolkhir, Farzad Kompani, Parvaiz A Koul, Sindhura Lakshmi Koulmane Laxminarayana, Ai Koyanagi, Kewal Krishan, Yuvaraj Krishnamoorthy, Burcu Kucuk Bicer, Nuworza Kugbey, Mukhtar Kulimbet, Akshay Kumar, G Anil Kumar, Narinder Kumar, Om P Kurmi, Ambily Kuttikkattu, Carlo La Vecchia, Arista Lahiri, Dharmesh Kumar Lal, Judit Lám, Qing Lan, Iván Landires, Bagher Larijani, Savita Lasrado, Jerrald Lau, Paolo Lauriola, Caterina Ledda, Sang-woong Lee, Shaun Wen Huey Lee, Wei-Chen Lee, Yeong Yeh Lee, Yo Han Lee, Samson Mideksa Legesse, James Leigh, Elvynna Leong, Ming-Chieh Li, Stephen S Lim, Gang Liu, Jue Liu, Chun-Han Lo, Ayush Lohiya, Platon D Lopukhov, László Lorenzovici, Mojgan Lotfi, Joana A Loureiro, Raimundas Lunevicius, Farzan Madadizadeh, Ahmad R Mafi, Sameh Magdeldin, Soleiman Mahjoub, Ata Mahmoodpoor, Morteza Mahmoudi, Marzieh Mahmoudimanesh, Rashidul Alam Mahumud, Azeem Majeed, Jamal Majidpoor, Alaa Makki, Konstantinos Christos Makris, Elaheh Malakan Rad, Mohammad-Reza Malekpour, Reza Malekzadeh, Ahmad Azam Malik, Tauqeer Hussain Mallhi, Sneha Deepak Mallya, Mohammed A Mamun, Ana Laura Manda, Fariborz Mansour-Ghanaei, Borhan Mansouri, Mohammad Ali Mansournia, Lorenzo Giovanni Mantovani, Santi Martini, Miquel Martorell, Sahar Masoudi, Seyedeh Zahra Masoumi, Clara N Matei, Elezebeth Mathews, Manu Raj Mathur, Vasundhara Mathur, Martin McKee, Jitendra Kumar Meena, Khalid Mehmood, Entezar Mehrabi Nasab, Ravi Mehrotra, Addisu Melese, Walter Mendoza, Ritesh G Menezes, SIsay Derso Mengesha, Laverne G Mensah, Alexios-Fotios A Mentis, Andry Yasmid Mera Mera-Mamián, Tuomo J Meretoja, Mehari Woldemariam Merid, Amanual Getnet Mersha, Belsity Temesgen Meselu, Mahboobeh Meshkat, Tomislav Mestrovic, Junmei Miao Jonasson, Tomasz Miazgowski, Irmina Maria Michalek, Gelana Fekadu Worku Mijena, Ted R Miller, Shabir Ahmad Mir, Seyed Kazem Mirinezhad, Seyyedmohammadsadeq Mirmoeeni, Mohammad Mirza-Aghazadeh-Attari, Hamed Mirzaei, Hamid Reza Mirzaei, Abay Sisay Misganaw, Sanjeev Misra, Karzan Abdulmuhsin Mohammad, Esmaeil Mohammadi, Mokhtar Mohammadi, Abdollah Mohammadian-Hafshejani, Reza Mohammadpourhodki, Arif Mohammed, Shafiu Mohammed, Syam Mohan, Mohammad Mohseni, Nagabhishek Moka, Ali H Mokdad, Alex Molassiotis, Mariam Molokhia, Kaveh Momenzadeh, Sara Momtazmanesh, Lorenzo Monasta, Ute Mons, Ahmed Al Montasir, Fateme Montazeri, Arnulfo Montero, Mohammad Amin Moosavi, Abdolvahab Moradi, Yousef Moradi, Mostafa Moradi Sarabi, Paula Moraga, Lidia Morawska, Shane Douglas Morrison, Jakub Morze, Abbas Mosapour, Ebrahim Mostafavi, Seyyed Meysam Mousavi, Haleh Mousavi Isfahani, Amin Mousavi Khaneghah, Christine Mpundu-Kaambwa, Sumaira Mubarik, Francesk Mulita, Daniel Munblit, Sandra B Munro, Efrén Murillo-Zamora, Jonah Musa, Ashraf F Nabhan, Ahamarshan Jayaraman Nagarajan, Shankar Prasad Nagaraju, Gabriele Nagel, Mohammadreza Naghipour, Mukhammad David Naimzada, Tapas Sadasivan Nair, Atta Abbas Naqvi, Sreenivas Narasimha Swamy, Aparna Ichalangod Narayana, Hasan Nassereldine, Zuhair S Natto, Biswa Prakash Nayak, Rawlance Ndejjo, Sabina Onyinye Nduaguba, Wogene Wogene Negash, Seyed Aria Nejadghaderi, Kazem Nejati, Sandhya Neupane Kandel, Huy Van Nguyen Nguyen, Robina Khan Niazi, Nurulamin M Noor, Maryam Noori, Nafise Noroozi, Hasti Nouraei, Ali Nowroozi, Virginia Nuñez-Samudio, Chimezie Igwegbe Nzoputam, Ogochukwu Janet Nzoputam, Bogdan Oancea, Oluwakemi Ololade Odukoya, Onome Bright Oghenetega, Ropo Ebenezer Ogunsakin, Ayodipupo Sikiru Oguntade, In-Hwan Oh, Hassan Okati-Aliabad, Akinkunmi Paul Okekunle, Andrew T Olagunju, Tinuke O Olagunju, Babayemi Oluwaseun Olakunde, Isaac Iyinoluwa Olufadewa, Emad Omer, Abidemi E Emmanuel Omonisi, Sokking Ong, Obinna E Onwujekwe, Hans Orru, Stanislav S Otstavnov, Abderrahim Oulhaj, Bilcha Oumer, Oluwatomi Funbi Owopetu, Babatunji Emmanuel Oyinloye, Mahesh P A, Alicia Padron-Monedero, Jagadish Rao Padubidri, Babak Pakbin, Keyvan Pakshir, Reza Pakzad, Tamás Palicz, Adrian Pana, Anamika Pandey, Ashok Pandey, Suman Pant, Shahina Pardhan, Eun-Cheol Park, Eun-Kee Park, Seoyeon Park, Jay Patel, Siddhartha Pati, Rajan Paudel, Uttam Paudel, Mihaela Paun, Hamidreza Pazoki Toroudi, Minjin Peng, Jeevan Pereira, Renato B Pereira, Simone Perna, Navaraj Perumalsamy, Richard G Pestell, Raffaele Pezzani, Cristiano Piccinelli, Julian David Pillay, Zahra Zahid Piracha, Tobias Pischon, Maarten J Postma, Ashkan Pourabhari Langroudi, Akram Pourshams, Naeimeh Pourtaheri, Akila Prashant, Mirza Muhammad Fahd Qadir, Zahiruddin Quazi Syed, Mohammad Rabiee, Navid Rabiee, Amir Radfar, Raghu Anekal Radhakrishnan, Venkatraman Radhakrishnan, Mojtaba Raeisi, Ata Rafiee, Alireza Rafiei, Nasiru Raheem, Fakher Rahim, Md Obaidur Rahman, Mosiur Rahman, Muhammad Aziz Rahman, Amir Masoud Rahmani, Shayan Rahmani, Vahid Rahmanian, Nazanin Rajai, Aashish Rajesh, Pradhum Ram, Kiana Ramezanzadeh, Juwel Rana, Kamal Ranabhat, Priyanga Ranasinghe, Chythra R Rao, Sowmya J Rao, Sina Rashedi, Amirfarzan Rashidi, Mahsa Rashidi, Mohammad-Mahdi Rashidi, Zubair Ahmed Ratan, David Laith Rawaf, Salman Rawaf, Lal Rawal, Reza Rawassizadeh, Mohammad Sadegh Razeghinia, Ashfaq Ur Rehman, Inayat ur Rehman, Marissa B Reitsma, Andre M N Renzaho, Maryam Rezaei, Nazila Rezaei, Negar Rezaei, Nima Rezaei, Saeid Rezaei, Mohsen Rezaeian, Aziz Rezapour, Abanoub Riad, Reza Rikhtegar, Maria Rios-Blancas, Thomas J Roberts, Peter Rohloff, Esperanza Romero-Rodríguez, Gholamreza Roshandel, Godfrey M Rwegerera, Manjula S, Maha Mohamed Saber-Ayad, Bahar Saberzadeh-Ardestani, Siamak Sabour, Basema Saddik, Erfan Sadeghi, Mohammad Reza Saeb, Umar Saeed, Mohsen Safaei, Azam Safary, Maryam Sahebazzamani, Amirhossein Sahebkar, Harihar Sahoo, Mirza Rizwan Sajid, Hedayat Salari, Sana Salehi, Marwa Rashad Salem, Hamideh Salimzadeh, Yoseph Leonardo Samodra, Abdallah M Samy, Juan Sanabria, Senthilkumar Sankararaman, Francesco Sanmarchi, Milena M Santric-Milicevic, Muhammad Arif Nadeem Saqib, Arash Sarveazad, Fatemeh Sarvi, Brijesh Sathian, Maheswar Satpathy, Nicolas Sayegh, Ione Jayce Ceola Schneider, Michaël Schwarzinger, Mario Šekerija, Subramanian Senthilkumaran, Sadaf G Sepanlou, Allen Seylani, Kenbon Seyoum, Feng Sha, Omid Shafaat, Pritik A Shah, Saeed Shahabi, Izza Shahid, Mohammad Amin Shahrbaf, Hamid R Shahsavari, Masood Ali Shaikh, Mohammed Feyisso Shaka, Elaheh Shaker, Mohammed Shannawaz, Mequannent Melaku Sharew Sharew, Azam Sharifi, Javad Sharifi-Rad, Purva Sharma, Bereket Beyene Shashamo, Aziz Sheikh, Mahdi Sheikh, Sara Sheikhbahaei, Rahim Ali Sheikhi, Ali Sheikhy, Peter Robin Shepherd, Adithi Shetty, Jeevan K Shetty, Ranjitha S Shetty, Kenji Shibuya, Reza Shirkoohi, Hesamaddin Shirzad-Aski, K M Shivakumar, Siddharudha Shivalli, Velizar Shivarov, Parnian Shobeiri, Zahra Shokri Varniab, Seyed Afshin Shorofi, Sunil Shrestha, Migbar Mekonnen Sibhat, Sudeep K Siddappa Malleshappa, Negussie Boti Sidemo, Diego Augusto Santos Silva, Luís Manuel Lopes Rodrigues Silva, Guilherme Silva Julian, Nicola Silvestris, Wudneh Simegn, Achintya Dinesh Singh, Ambrish Singh, Garima Singh, Harpreet Singh, Jasvinder A Singh, Jitendra Kumar Singh, Paramdeep Singh, Surjit Singh, Dhirendra Narain Sinha, Abiy H Sinke, Md Shahjahan Siraj, Freddy Sitas, Samarjeet Singh Siwal, Valentin Yurievich Skryabin, Anna Aleksandrovna Skryabina, Bogdan Socea, Matthew J Soeberg, Ahmad Sofi-Mahmudi, Yonatan Solomon, Mohammad Sadegh Soltani-Zangbar, Suhang Song, Yimeng Song, Reed J D Sorensen, Sergey Soshnikov, Houman Sotoudeh, Alieu Sowe, Mu'awiyyah Babale Sufiyan, Ryan Suk, Muhammad Suleman, Rizwan Suliankatchi Abdulkader, Saima Sultana, Daniel Sur, Miklós Szócska, Seidamir Pasha Tabaeian, Rafael Tabarés-Seisdedos, Seyyed Mohammad Tabatabaei, Takahiro Tabuchi, Hooman Tadbiri, Ensiyeh Taheri, Majid Taheri, Moslem Taheri Soodejani, Ken Takahashi, Iman M Talaat, Mircea Tampa, Ker-Kan Tan, Nathan Y Tat, Vivian Y Tat, Ahmad Tavakoli, Arash Tavakoli, Arash Tehrani-Banihashemi, Yohannes Tekalegn, Fisaha Haile Tesfay, Rekha Thapar, Aravind Thavamani, Viveksandeep Thoguluva Chandrasekar, Nihal Thomas, Nikhil Kenny Thomas, Jansje Henny Vera Ticoalu, Amir Tiyuri, Daniel Nigusse Tollosa, Roman Topor-Madry, Mathilde Touvier, Marcos Roberto Tovani-Palone, Eugenio Traini, Mai Thi Ngoc Tran, Jaya Prasad Tripathy, Gebresilasea Gendisha Ukke, Irfan Ullah, Saif Ullah, Sana Ullah, Bhaskaran Unnikrishnan, Marco Vacante, Maryam Vaezi, Sahel Valadan Tahbaz, Pascual R Valdez, Constantine Vardavas, Shoban Babu Varthya, Siavash Vaziri, Diana Zuleika Velazquez, Massimiliano Veroux, Paul J Villeneuve, Francesco S Violante, Sergey Konstantinovitch Vladimirov, Vasily Vlassov, Bay Vo, Linh Gia Vu, Abdul Wadood Wadood, Yasir Waheed, Mandaras Tariku Walde, Richard G Wamai, Cong Wang, Fang Wang, Ning Wang, Yu Wang, Paul Ward, Abdul Waris, Ronny Westerman, Nuwan Darshana Wickramasinghe, Melat Woldemariam, Berhanu Woldu, Hong Xiao, Suowen Xu, Xiaoyue Xu, Lalit Yadav, Seyed Hossein Yahyazadeh Jabbari, Lin Yang, Fereshteh Yazdanpanah, Yigizie Yeshaw, Yazachew Yismaw, Naohiro Yonemoto, Mustafa Z Younis, Zabihollah Yousefi, Fatemeh Yousefian, Chuanhua Yu, Yong Yu, Ismaeel Yunusa, Mazyar Zahir, Nazar Zaki, Burhan Abdullah Zaman, Moein Zangiabadian, Fariba Zare, Iman Zare, Zahra Zareshahrabadi, Armin Zarrintan, Mikhail Sergeevich Zastrozhin, Mohammad A Zeineddine, Dongyu Zhang, Jianrong Zhang, Yunquan Zhang, Zhi-Jiang Zhang, Linghui Zhou, Sanjay Zodpey, Mohammad Zoladl, Theo Vos, Simon I Hay, Lisa M Force, Christopher J L Murray

## Abstract

**Background:**

Understanding the magnitude of cancer burden attributable to potentially modifiable risk factors is crucial for development of effective prevention and mitigation strategies. We analysed results from the Global Burden of Diseases, Injuries, and Risk Factors Study (GBD) 2019 to inform cancer control planning efforts globally.

**Methods:**

The GBD 2019 comparative risk assessment framework was used to estimate cancer burden attributable to behavioural, environmental and occupational, and metabolic risk factors. A total of 82 risk–outcome pairs were included on the basis of the World Cancer Research Fund criteria. Estimated cancer deaths and disability-adjusted life-years (DALYs) in 2019 and change in these measures between 2010 and 2019 are presented.

**Findings:**

Globally, in 2019, the risk factors included in this analysis accounted for 4·45 million (95% uncertainty interval 4·01–4·94) deaths and 105 million (95·0–116) DALYs for both sexes combined, representing 44·4% (41·3–48·4) of all cancer deaths and 42·0% (39·1–45·6) of all DALYs. There were 2·88 million (2·60–3·18) risk-attributable cancer deaths in males (50·6% [47·8–54·1] of all male cancer deaths) and 1·58 million (1·36–1·84) risk-attributable cancer deaths in females (36·3% [32·5–41·3] of all female cancer deaths). The leading risk factors at the most detailed level globally for risk-attributable cancer deaths and DALYs in 2019 for both sexes combined were smoking, followed by alcohol use and high BMI. Risk-attributable cancer burden varied by world region and Socio-demographic Index (SDI), with smoking, unsafe sex, and alcohol use being the three leading risk factors for risk-attributable cancer DALYs in low SDI locations in 2019, whereas DALYs in high SDI locations mirrored the top three global risk factor rankings. From 2010 to 2019, global risk-attributable cancer deaths increased by 20·4% (12·6–28·4) and DALYs by 16·8% (8·8–25·0), with the greatest percentage increase in metabolic risks (34·7% [27·9–42·8] and 33·3% [25·8–42·0]).

**Interpretation:**

The leading risk factors contributing to global cancer burden in 2019 were behavioural, whereas metabolic risk factors saw the largest increases between 2010 and 2019. Reducing exposure to these modifiable risk factors would decrease cancer mortality and DALY rates worldwide, and policies should be tailored appropriately to local cancer risk factor burden.

**Funding:**

Bill & Melinda Gates Foundation.

## Introduction

Cancer is the second leading cause of death worldwide, and exposure to risk factors plays an important role in the biology and burden of many cancer types.^1^, ^2^, ^3^, ^4^ Understanding the relative contribution of modifiable risk factors to cancer burden and their trends over time is crucial to informing cancer control efforts both locally and globally. In 2015, the UN released the Sustainable Development Goals (SDGs), with SDG target 3.4 focusing on reducing global premature mortality by a third for non-communicable diseases, including cancer, by 2030. Effectively addressing the growing burden of cancer globally will require comprehensive measures that incorporate both curative and preventive interventions, particularly in light of the anticipated challenges that the COVID-19 pandemic will bring in progress towards SDG target 3.4.^5^, ^6^, ^7^

Although some cancer cases are not preventable, governments can work on a population level to support an environment that minimises exposure to known cancer risk factors. Primary prevention, or the prevention of a cancer developing, is a particularly cost-effective strategy,^8^ although it must be paired with more comprehensive efforts to address cancer burden, including secondary prevention initiatives, such as screening programmes, and ensuring effective capacity to diagnose and treat those with cancer. As part of cancer control strategies, prevention requires identification of causal risk factors, determination of contribution to local cancer burden, and development of effective strategies for their mitigation. Previous studies have quantified the burden of cancer attributable to individual risk factors globally or for several risk factors in select countries and regions,^9^, ^10^, ^11^, ^12^, ^13^, ^14^, ^15^, ^16^, ^17^, ^18^, ^19^, ^20^ providing crucial location and risk-factor-specific information. However, comprehensive cancer risk factor estimates do not exist for many countries, leaving an important void as countries develop and update their cancer control plans. The Global Cancer Observatory from the International Agency for Research on Cancer provides estimates of global, regional, and national risk-attributable cancer burden for a subset of potentially modifiable risk factors (eg, obesity, alcohol consumption, infections, and ultraviolet radiation), but these estimates are not provided together in a comprehensive fashion across time, and some potentially modifiable risk factors are not estimated as part of this effort.^21^, ^22^, ^23^, ^24^

**Research in context**

**Evidence before this study**

We identified previous work that primarily estimated the attributable cancer burden for single risk factors globally or multiple risk factors for single countries. The Global Cancer Observatory also provides estimates of cancer-attributable burden for select risk factor categories separately. One previous comparative risk assessment project estimated risk-attributable cancer mortality for nine risk factors. We searched titles and abstracts in PubMed for English-language research papers that were published between Jan 1, 2010, and June 1, 2021, using the search terms “cancer or neoplasm or tumor or malignancy” and “risk factor or attributable risk or population attributable fraction” and “global or international or worldwide or world” and “burden or metrics or incidence or mortality”, but did not identify additional informative studies. There is a gap in the literature on global estimates of risk-attributable cancer burden for a comprehensive list of risk factors that incorporate both cancer-related mortality and disability.

**Added value of this study**

We report, for the first time, the global cancer burden attributable to a comprehensive list of behavioural, metabolic, and environmental and occupational risk factors using Global Burden of Diseases, Injuries, and Risk Factors 2019 results. By estimating risk-attributable cancer burden nationally and globally using both mortality and disability-adjusted life-years (DALYs), this study provides a new perspective on attributable cancer burden. Globally, a large portion of cancer deaths and DALYs were attributable to the modifiable risk factors included, with behavioural risks representing the largest attributable burden. We identified substantial differences in attributable cancer death and DALY burden across Socio-demographic Index quintiles and between sexes. Risk-attributable cancer death and DALY burden increased globally from 2010 to 2019, with metabolic risk factors contributing to the largest percentage increases, most notably in low and low-middle Socio-demographic Index countries.

**Implications of all the available evidence**

The burden of cancer remains an important public health challenge that is growing in magnitude globally. Modifiable risk factors are important contributors to cancer mortality and DALYs globally, with contribution varying by setting. The results from this study highlight the need for context-specific policies aimed at reducing exposure to risk factors as part of comprehensive cancer control efforts.

To our knowledge, the Global Burden of Diseases, Injuries, and Risk Factors Study (GBD) is the only study to date that quantifies cancer burden attributable to a broad set of modifiable risk factors for each GBD round, for all countries around the world, across age groups, for both sexes, and over time. GBD 2019, the most recent iteration of the GBD study, provides an opportunity to evaluate the global burden of cancer attributable to risk factors. A previous study used a similar comparative risk framework to estimate mortality from 12 cancer types and nine behavioural and environmental risk factors based on WHO cancer mortality data, but this analysis was limited to 2001 and has not been updated in a formal GBD analysis since.^25^ Herein, we present estimates of 82 risk–outcome pairs, including cancer deaths and disability-adjusted life-years (DALYs) attributable to risk factors at global, regional, and national levels in 2019, and assess the temporal trends from 2010 to 2019 of cancer burden attributable to environmental and occupational, behavioural, and metabolic risk factors to inform efforts to reduce exposure to cancer risk factors (appendix pp 157–60).^26^ This manuscript was produced as part of the GBD Collaborator Network and in accordance with the GBD Protocol.

## Methods

### Overview of the GBD study

The GBD study was developed to provide global health estimates that are comprehensive and comparable for causes of death, disability, and their associated risk factors. GBD 2019 estimates mortality, incidence, prevalence, years of life lost (YLLs), years of life lived with disability (YLDs), and DALYs for 369 causes of death and disability and 87 risk factors, and groups of risk factors at the global level, regionally, and for 204 countries and territories. The 2019 iteration of the GBD is the most up to date and supersedes all previous iterations. This Article reports estimates from 2010 to 2019, but extended years of estimates (1990–2019) are available online via the GBD Compare Tool and GBD Results Tool. Rates are reported per 100 000 person-years, and age-standardised rates were calculated with the GBD world population standard.^27^ Select results in this Article are presented by Socio-demographic Index (SDI) to describe differences in cancer burden attributable to risk factors across the spectrum of sociodemographic development.^28^ SDI is a summary index calculated from the total fertility rate in women younger than 25 years, lag-distributed income per capita, and mean education for individuals aged 15 years and older (appendix p 55). The index ranges from 0 (low SDI) to 100 (high SDI), with quintiles used to describe low, low-middle, middle, high-middle, and high SDI countries in 2019. Although both cancer deaths and DALYs attributable to risk factors are presented here, the majority of cancer DALYs globally are due to YLLs, reflective of cancer deaths with weighting for the age at death.^1^ Thus, we do not present here estimates of risk-attributable YLLs and YLDs, but they are available in the online tools.

The GBD study is compliant with the Guidelines for Accurate and Transparent Health Estimates Reporting, and additional details from this Article and the appendix are available in the GBD 2019 summary papers.^1^, ^26^, ^27^, ^28^, ^29^

### Data sources for cancer burden estimates

Cancers included in the GBD study were those defined in chapter 2 (neoplasms) in the tenth revision of the International Classification of Diseases, with the exception of Kaposi sarcoma, for which most deaths are attributed to HIV/AIDS (appendix p 29).^30^ Data sources used to inform the cancer estimates were obtained from vital registration systems, sample vital registration systems, verbal autopsy reports, and national and subnational population-based cancer registries. All data sources are provided with a unique identifier and compiled in the Global Health Data Exchange, which is publicly accessible.

### Cancer burden estimation

Cancer registry, vital registration systems, and verbal autopsy data are used to inform cancer mortality modelling in the GBD study, as one of these data sources might exist in a location where others do not. In some locations, cancer mortality data are sparse, but cancer incidence is reported by national or subnational population-based cancer registries. To maximise data informing mortality models, cancer incidence data were transformed into cancer mortality estimates with modelled mortality-to-incidence ratios (MIRs). MIRs specific to cancer causes (cancer types) were modelled with a spatiotemporal Gaussian process regression (appendix pp 30–31).^1^, ^26^ Mortality estimates from MIR-transformed incidence data were then pooled with mortality data from vital registration systems and verbal autopsies and used as inputs in cancer-specific Cause of Death Ensemble models.^31^ These models use all available data and location-level covariates to test individual and ensemble models, and produce estimates of death for every cause, sex, age group, location, and year estimated within GBD 2019, selecting models on the basis of out-of-sample predictive validity. Finally, the predicted mortality estimates were adjusted to align with independently modelled all-cause mortality estimates for each age, group, sex, location, and year.^28^

Non-fatal cancer-specific computations began by generating incidence estimates from the modelled cancer mortality estimates using the MIRs corresponding to each cancer cause, age group, sex, location, and year. Cancer 10-year prevalence was modelled with incidence, background mortality, and estimated relative survival curves and their correlation with modelled MIRs (appendix pp 49–53). Cancer-specific prevalence for each sequela, age, group, sex, location, and year combination is made up by four sequelae phases: (1) diagnosis and treatment, (2) remission, (3) metastatic and disseminated, and (4) terminal. To generate YLDs, each sequela prevalence was multiplied by its corresponding sequelae-specific disability weight (appendix pp 53–54). Disability weights describe the severity of health loss associated with a sequelae-specific condition and range from 0 (equivalent to full health) to 1 (equivalent to death).^28^ YLLs were computed by multiplying the number of deaths in a specific age group for each cancer cause by the remaining standard life expectancy at the age of death.^27^ Finally, DALYs were calculated as the sum of YLDs and YLLs.

### Risk-factor-attributable cancer burden estimation

GBD 2019 includes risk factors that are broadly categorised into three groups: (1) environmental and occupational, (2) behavioural, and (3) metabolic. This study includes 82 cancer risk–outcome pairs (23 cancer types and 34 risk factors), with risk factors identified with the World Cancer Research Fund criteria^32^ (appendix pp 154–56). Risk–outcome pairs are organised into four mutually exclusive levels with increasing risk factor resolution (appendix pp 152–53). The general approach to risk factor estimation in the GBD study is described in this paper, and details on the modelling approach for each risk factor are available in the GBD 2019 risk factors summary paper^26^ and the appendix (pp 72–145). For percentages of risk-attributable cancer deaths or DALYs reported, the total cancer burden (risk and non-risk cancer burden) included non-melanoma skin cancers.

The GBD comparative risk assessment framework was used to compute the fraction of cancer-specific burden attributable to each risk factor. The framework is divided into six main steps that are followed for each risk–outcome pair. First, the World Cancer Research Fund criteria were used to identify risk factors with convincing or probable evidence for a causal association.^32^ For GBD 2019, systematic reviews were updated to ensure appropriate inclusion of risk–outcome pairs.^26^ Second, to estimate relative risks for each risk–outcome pair as a function of exposure, existing systematic reviews were updated and meta-analyses of relative risks were done. In GBD 2019, the meta-analytic approach was updated for a selected set of continuous risk factors with GBD's meta-regression-Bayesian, regularised, trimmed tool (appendix pp 58–59). Third, risk factor exposure levels and distributions were modelled for each age, sex, location, and year combination with data available from published studies, household surveys, censuses, administrative data, ground monitor data, or remote sensing data. To model risk factor exposure level, the GBD uses either Bayesian meta-regression modelling (DisMod-MR 2.1), a flexible approach that can incorporate sex-specific and age-specific data, or spatiotemporal Gaussian process regression, the preferred approach when exposure is stable across age groups (appendix p 62). Fourth, for each risk factor, the theoretical minimum risk exposure level was identified, a counterfactual scenario in which a given population receives the optimal level of risk exposure (ie, no exposure for monotonically increasing risk functions such as smoking, the lowest point of the risk function of exposure for J-shaped or U-shaped risks such as high BMI, and the 85th percentile of exposure in cohorts and trials for protective risks such as fruit intake, weighted by the relative global magnitude of each outcome). Fifth, the population attributable fraction for each risk–outcome pair was calculated across age, sex, location, and year, taking into account the risk function (ie, relative risk), exposure level, and the theoretical minimum risk exposure level (appendix pp 62–64). Sixth, for some risk factors it was necessary to estimate the population attributable fraction in combination with other risk factors by considering mediation. For instance, calculating the population attributable fraction for fruit intake should account for the potential mediating effect of fibre intake. Thus, a mediation matrix was used to correct for population attributable fraction overestimation that would occur if independence of specific risk factors was assumed.^26^ Last, to estimate the cancer burden attributable to each estimated risk factor, the YLLs, YLDs, and deaths for a given cancer type were multiplied by the corresponding risk factor population attributable fraction. The sum of YLLs and YLDs was used to estimate the cancer DALYs attributable to risk factors.

### Estimating uncertainty

To account for uncertainty in the attributable burden estimates, a total of 1000 draws were estimated, from which the lower and upper bounds of the 95% uncertainty interval (UI) were obtained from the 25th and 975th ranked values. Error was propagated through each estimation step, including the estimation of cancer deaths and DALYs, exposure, relative risk functions, and for relevant risk factors the theoretical minimum risk exposure level.

### Role of the funding source

The funders of the study had no role in study design, data collection, data analysis, data interpretation, or writing of the report.

## Results

In 2019, the total number of cancer deaths globally attributable to all estimated risk factors was 4·45 million (95% UI 4·01–4·94) for both sexes combined, accounting for 44·4% (41·3–48·4) of all cancer deaths. There were 2·88 million (2·60–3·18) risk-attributable cancer deaths in males and 1·58 million (1·36–1·84) in females, representing 50·6% (47·8–54·1) of all cancer deaths in males and 36·3% (32·5–41·3) in females (appendix pp 191–99). The total number of cancer DALYs globally attributable to all estimated risk factors in 2019 was 105 million (95·0–116) for both sexes combined, which accounted for 42·0% (39·1–45·6) of all cancer DALYs. Males were estimated to have 67·5 million (60·8–75·1) cancer DALYs attributable to risk factors, or 48·0% (45·3–51·5) of all cancer DALYs in males, whereas females were estimated to have 37·6 million (32·8–43·1) cancer DALYs attributable to risk factors, or 34·3% (30·9–38·7) of all cancer DALYs in females.

**Figure 1:**
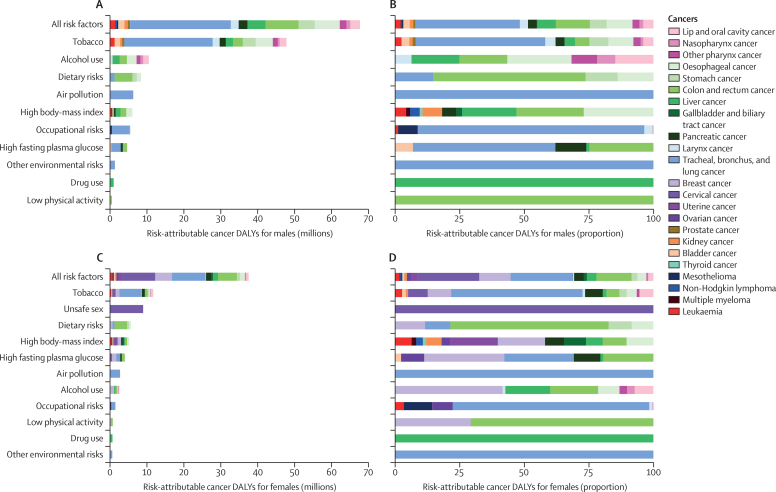
Cancer DALYs attributable to 11 Level 2 risk factors globally in 2019 (A) Absolute cancer DALYs for males. (B) Proportional cancer DALYs for males. (C) Absolute cancer DALYs for females. (D) Proportional cancer DALYs for females. Air pollution includes ambient particulate matter pollution and household air pollution from solid fuels. Other environmental risks include residential radon. Occupational risks include exposure to 13 specific carcinogens. Dietary risks include nine specific risk factors relevant to cancer. Tobacco includes smoking, chewing tobacco, and second-hand smoke. See appendix (pp 157–60) for details and definitions of each Level 2 risk factor on the y-axis. See appendix (p 161) for further details about global absolute and proportional cancer deaths attributable to Level 2 risk factors. DALYs=disability-adjusted life-years.

The leading Level 2 risk factor (appendix pp 152–53) in males in terms of attributable cancer DALYs was tobacco (figure 1), which accounted for 33·9% (32·3–35·4) of all cancer DALYs in males in 2019 (appendix pp 191–93). Alcohol use, dietary risks, and air pollution were the next greatest risk factors, accounting for 7·4% (6·7–8·2), 5·9% (4·4–8·3), and 4·4% (3·4–5·5), respectively, of all male cancer DALYs in 2019. Tobacco was also the leading Level 2 risk factor for females globally in terms of attributable cancer DALYs (figure 1) and accounted for 10·7% (9·9–11·5) of all female cancer DALYs in 2019 (appendix pp 191–93). Unsafe sex was the second leading risk factor for females, accounting for 8·2% (7·0–8·8) of all female cancer DALYs in 2019, followed by dietary risks (5·1% [4·0–6·7]), high BMI (4·7% [2·8–7·0]), and high fasting plasma glucose (3·6% [1·0–7·5]). Ranking of Level 2 risk factors by attributable cancer deaths globally in 2019 showed similar ranking as by attributable cancer DALYs (appendix pp 181–87).

**Figure 2:**
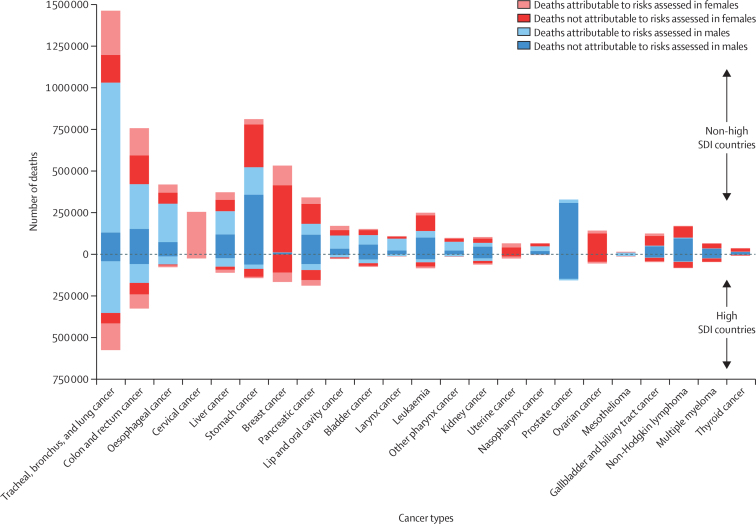
Global deaths from cancers attributable to risk factors in 2019 by sex and SDI Non-high SDI countries include low, low-middle, middle, and high-middle SDI countries. Cancer types are listed from left to right in order of greatest to least risk-attributable deaths. See appendix (pp 213–14, 217–18) for estimates for risk-attributable cancer deaths in high and non-high SDI locations by sex. See appendix (p 162) for further details about DALYs from cancers attributable to risk factors in 2019 by sex and SDI. For additional versions of this figure showing age-standardised mortality and DALY rates see appendix (pp 163–64). DALYs=disability-adjusted life-years. SDI=Socio-demographic Index.

The leading cancer in terms of risk-attributable deaths globally in 2019 for both males and females was tracheal, bronchus, and lung cancer (36·9% [34·2–39·3] of all attributable cancer deaths), followed by colon and rectum cancer, oesophageal cancer, and stomach cancer in males, and cervical cancer, colon and rectum cancer, and breast cancer in females (figure 2, appendix p 210). Deaths caused by cancer and risk-attributable deaths caused by cancer tended to be greater in males than females for leading causes of cancer death globally, with the exception of cancer types that occur predominantly in women (eg, breast) or are exclusively estimated in women in the GBD study (eg, cervical, ovarian, and uterine cancers; figure 2; for male-to-female ratios for risk-attributable cancer deaths see appendix pp 206–07). When excluding the sex-specific cancers with risk-attributable burden (cervical, ovarian, uterine, and prostate cancers) the male-to-female ratios for risk-attributable cancer deaths tended to be smaller in high SDI countries than in non-high SDI countries (low, low-middle, middle, and high-middle SDI countries; appendix pp 213–14, 217–18). Cancer deaths and risk-attributable cancer deaths globally for both sexes combined in 2019 occurred over-proportionally frequently in high SDI countries, with 25·4% (24·0–26·7) of cancer deaths and 26·5% (24·9–28·1) of risk-attributable cancer deaths occurring in high SDI countries, even though these countries had only 13·1% (12·5–13·8) of the global population (appendix p 221). The leading cancers, sex analysis, and results by SDI remained largely the same when the cancers were ranked by risk-attributable DALYs instead of deaths (appendix p 162).

**Figure 3:**
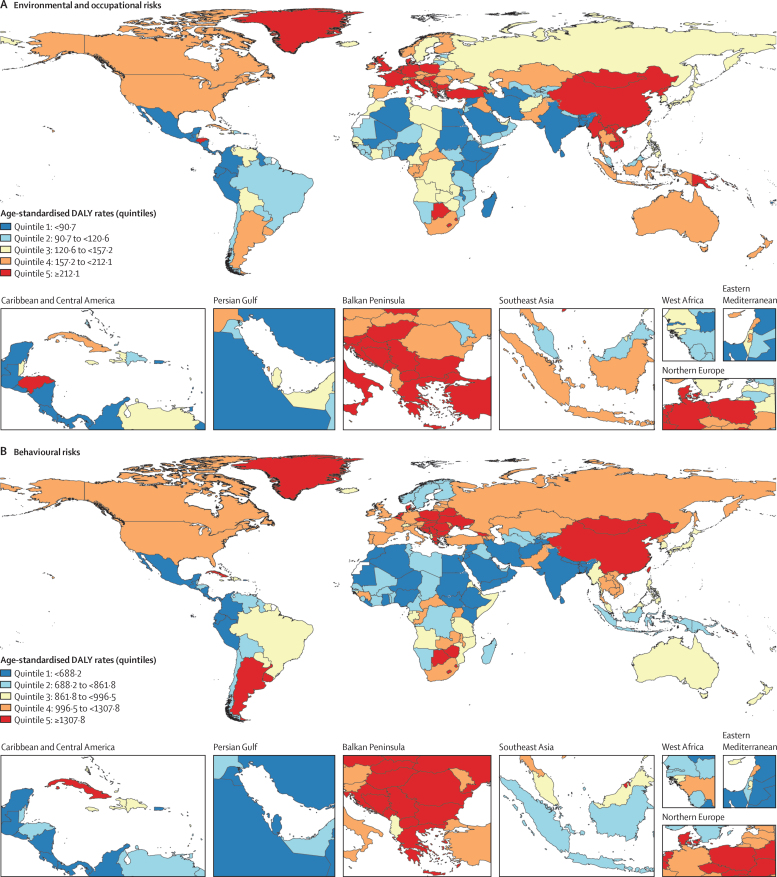

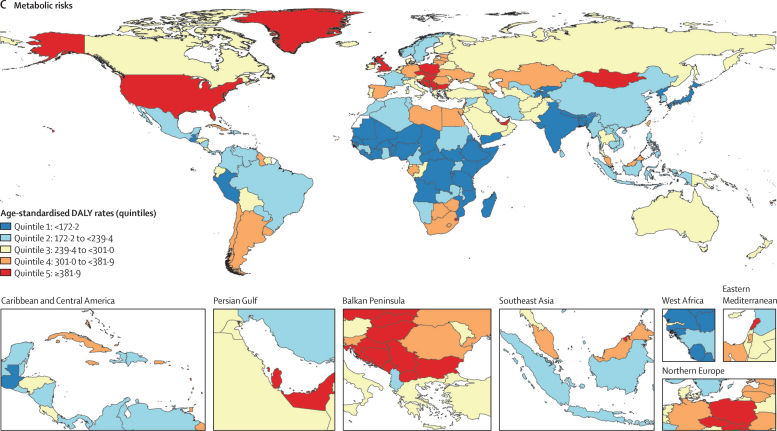
Global map of age-standardised DALY rate quintiles for risk-attributable cancer burden, both sexes combined, 2019 (A) Environmental and occupational risks. (B) Behavioural risks. (C) Metabolic risks. Each map represents estimates at the national level. Quintiles are based on age-standardised DALY rates per 100 000 person-years. See appendix (pp 165–68, 234–39) for further details of risk-attributable cancer deaths and DALYs for each country and territory. DALYs=disability-adjusted life-years.

Geographical patterns for cancer age-standardised death and DALY rates attributable to environmental and occupational, behavioural, and metabolic risks in 2019 differed around the world (figure 3, appendix pp 165–66), with generally higher age-standardised DALY rates within these Level 1 risk factor categories (appendix pp 152–53) notable in high-income North America, and central, western, and eastern Europe, and variably elevated rates by risk category in east and southeast Asia, southern Latin America, and southern Africa. Globally in 2019, the leading five regions in terms of risk-attributable cancer age-standardised death rates were central Europe (82·0 [71·0–94·9] per 100 000 person-years), east Asia (69·8 [58·0–83·0]), high-income North America (66·0 [60·5–72·1]), southern Latin America (64·2 [58·2–71·8]), and western Europe (63·8 [58·4–69·7]; appendix pp 174, 234–39). Details of risk-attributable cancer age-standardised death and DALY rates at the country level are available in the appendix (pp 234–239) and in the online GBD Compare and GBD Results Tools.

**Figure 4:**
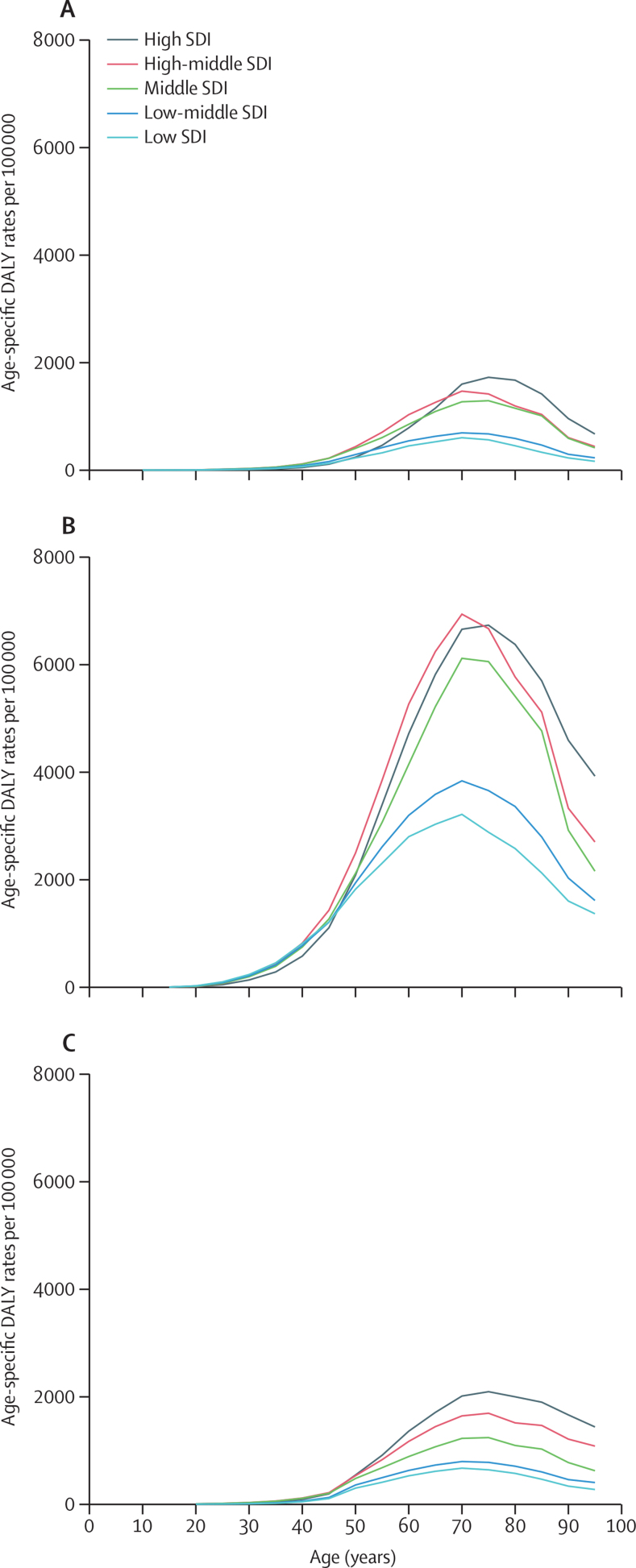
Estimates of age-specific rates of risk-attributable cancer DALYs, SDI quintiles, both sexes combined, 2019 (A) Environmental and occupational risks. (B) Behavioural risks. (C) Metabolic risks. Rates are expressed per 100 000 person-years. See appendix (pp 146–47) for details and definitions of the SDI regions. DALYs=disability-adjusted life-years. SDI=Socio-demographic Index.

Figure 4 shows age-specific attributable cancer DALY rates in 2019 by SDI quintiles. For the same age group, cancer DALY rates attributable to behavioural risks were generally greater than those attributable to environmental and occupational risks and metabolic risks, and attributable cancer DALY rates were generally greater with increasing SDI quintile. Attributable cancer DALY rates increased with age for each Level 1 risk category, before peaking at ages 70–74 years or ages 75–79 years, depending on the SDI quintile, with a later age peak generally on the higher end of the SDI spectrum.

For all risk factors estimated for both sexes combined between 2010 and 2019, global attributable cancer deaths increased by 20·4% (12·6–28·4) and DALYs by 16·8% (8·8–25·0), whereas the global age-standardised rates of attributable cancer deaths decreased by 6·9% (0·9–12·8) and cancer DALYs by 7·8% (1·4–14·0; appendix pp 240–42). The greatest percentage increase in attributable cancer deaths and DALYs among the Level 1 risk factor categories was in metabolic risks, which increased by 34·7% (27·9–42·8) and 33·3% (25·8–42·0), respectively, from 2010 to 2019, whereas behavioural risk-attributable cancer deaths and DALYs increased by 17·9% (10·4–26·0) and 14·4% (6·5–22·5), and environmental and occupational risk-attributable cancer deaths and DALYs increased by 16·7% (7·9–26·2) and 13·1% (3·9–23·1), respectively. Similarly, the greatest percentage increase in global risk-attributable cancer age-standardised death and DALY rates was in metabolic risk factors, which increased by 2·8% (–2·2 to 8·8) and 3·8% (–2·0 to 10·5), respectively, whereas behavioural risk factors decreased by 8·7% (2·7–14·5) and 9·6% (3·2–15·8), respectively, and environmental and occupational risk factors declined by 10·0% (2·8–16·7) and 11·4% (3·5–18·5), respectively.

**Figure 5:**
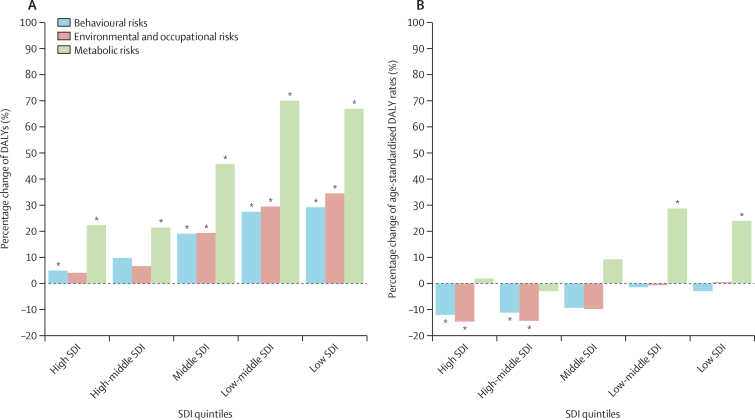
Percentage change of risk-attributable cancer DALY counts and age-standardised DALY rates for Level 1 risk factors by SDI quintile, both sexes combined, 2010–19 (A) Percentage change of risk-attributable cancer DALY counts by SDI quintile. (B) Percentage change of risk-attributable age-standardised cancer DALY rates by SDI quintile. See appendix (pp 146–47) for more information on SDI quintiles. See appendix (p172) for further information about the percentage change of risk-attributable cancer DALYs and age-standardised DALY rates for risk factors by GBD world super-region. See appendix (p 173) for percentage change of risk-attributable cancer deaths and age-standardised mortality rates by SDI quintile and GBD world super-region. DALYs=disability-adjusted life-years. GBD=Global Burden of Disease, Injuries, and Risk Factors Study. SDI=Socio-demographic Index. *95% uncertainty intervals that do not include zero.

Furthermore, the magnitude of change in risk-attributable cancer DALYs and deaths and age-standardised DALY and mortality rates varied greatly among super-regions and SDI quintiles (figure 5, appendix pp 148, 172, 223–25). The greatest increases in age-standardised DALY rates due to metabolic risks were seen in south Asia, north Africa and the Middle East, and sub-Saharan African super-regions, and in the low-middle and low SDI quintiles, whereas the greatest decreases in behavioural and environmental and occupational risks were seen in central Europe, eastern Europe, and central Asia; high-income; Latin American and the Caribbean super-regions; and in the high and high-middle SDI quintiles (appendix p 224). Generally, the super-regions and SDI quintiles with the greatest increase in age-standardised cancer DALY burden attributable to metabolic risks between 2010 and 2019 were those with the least improvement in cancer burden attributable to behavioural risks and environmental and occupational risks across the same time period (appendix p 224).

**Figure 6:**
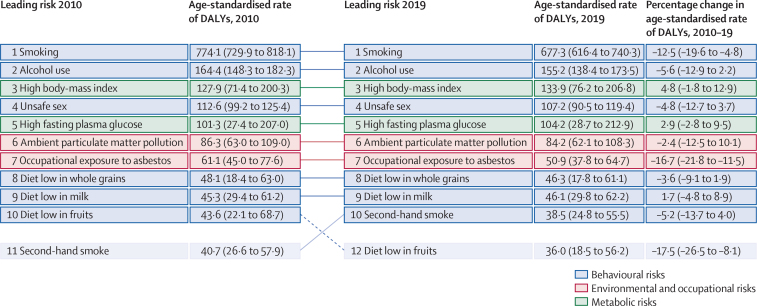
Leading risk factors at the most detailed level for risk-attributable cancer age-standardised DALY rates globally, both sexes combined, 2010–19 Top ten risk factors for age-standardised rates of cancer DALYs and risk factors moving in or out of the top ten between 2010 and 2019 are displayed for the global level. Dashed lines indicate decrease in rank. Solid lines indicate increase or no change in rank. Data in parentheses are 95% uncertainty intervals. Risk factors at the most detailed level reflect the GBD hierarchy in which these categories of risks fall, ranging from Levels 2 to 4 (see appendix p 152 for more information on risk factor levels in the GBD hierarchy). See appendix (pp 175–80) for an expanded version of this figure, which contains the top ten risk factors for risk-attributable cancer age-standardised DALY rates in males, females, and both sexes combined globally and by SDI quintile. See appendix (pp 181–87) for further details about the top ten risk factors for risk-attributable cancer age-standardised death rates for males, females, and both sexes combined globally and by SDI quintile. DALYs=disability-adjusted life-years. GBD=Global Burden of Disease, Injuries, and Risk Factors Study. SDI=Socio-demographic Index.

Finally, different patterns in the leading risk factors for attributable cancer age-standardised DALY rates were observed globally and across the SDI spectrum (figure 6, appendix pp 175–80). The leading nine risk factors at the most detailed level contributing to global cancer burden defined by age-standardised DALY rates did not change between 2010 and 2019, and the top three risk factors (smoking, alcohol use, and high BMI) were the same in the high SDI quintile as globally. Smoking and alcohol use remained the top two leading risk factors in the middle SDI quintile in 2010 and 2019, with unsafe sex decreasing from third to fifth position, high BMI rising from fourth to third position, and ambient particulate matter pollution rising from fifth to fourth position (appendix p 178). In the low SDI quintile, smoking remained the leading risk factor for risk-attributable cancer burden, with unsafe sex ranked second and alcohol use third in both 2010 and 2019. Within the top five leading risk factors in the low SDI quintile, high BMI and high fasting plasma glucose both increased (fifth to fourth, and sixth to fifth, respectively), and household air pollution from solid fuels decreased (fourth to sixth) between 2010 and 2019 (appendix pp 175–80).

## Discussion

Our analysis found that 44·4% (95% UI 41·3–48·4) of global cancer deaths and 42·0% (39·1–45·6) of global cancer DALYs were attributable to estimated risk factors in 2019. These findings highlight that a substantial proportion of cancer burden globally has potential for prevention through interventions aimed at reducing exposure to known cancer risk factors but also that a large proportion of cancer burden might not be avoidable through control of the risk factors currently estimated. Thus, cancer risk reduction efforts must be coupled with comprehensive cancer control strategies that include efforts to support early diagnosis and effective treatment. Most attributable cancer DALYs were accounted for by behavioural risk factors, such as tobacco use, alcohol use, unsafe sex, and dietary risks, suggesting a need for concerted efforts to address behavioural risk factors to effectively reduce cancer burden globally. Attributable cancer DALYs from each Level 1 risk factor group generally increased with increasing SDI, and although there were similarities in the leading risk factors across the SDI spectrum for both sexes combined (ie, smoking and alcohol use), there were differences in risk factor patterns following these leading risks, highlighting the need for cancer risk reduction efforts to be context specific. Between 2010 and 2019, age-standardised cancer DALYs attributable to all risk factors declined by 7·8% (1·4 to 14·0). Despite this decline, a global increase in age-standardised cancer DALYs (3·8% [–2·0 to 10·5]) attributable to metabolic risks was seen, largely driven by substantial increases in low and low-middle SDI countries. Furthermore, total risk-attributable cancer absolute DALY burden globally and in all SDI quintiles grew between 2010 and 2019, underscoring an expanding need for health systems around the world with capacity to comprehensively care for individuals with cancer, while developing and implementing cancer control efforts that consider risk reduction strategies. These estimates might help inform cancer control planning by identifying leading modifiable risk factors for cancer around the world, including for countries that might not have previous local research on cancer burden and cancer risk factor exposures.

To our knowledge, this study represents the largest effort to date to determine the global burden of cancer attributable to risk factors, and it contributes to a growing body of evidence aimed at estimating the risk-attributable burden for specific cancers nationally,^9^, ^13^, ^14^, ^15^, ^16^, ^17^, ^18^, ^19^, ^20^, ^33^, ^34^ internationally,^35^ and globally.^21^, ^22^, ^23^, ^24^, ^25^ Our study builds on existing evidence by estimating both deaths and DALYs due to risk-attributable cancer burden, across a spectrum of cancer types and risk factors, for all countries, age groups, and sexes, over time. When comparing the results from this study with studies reporting national-level population attributable fraction estimates, GBD 2019 generally reported higher values for all risk factors combined. These comparisons are between cases and deaths for a subset of countries and differences might be due to a greater number of risk factors estimated and greater estimates for select risk factors, such as smoking, potentially due to differences in exposure definitions and risk–outcome pairs estimated.^13^, ^14^, ^15^, ^16^, ^17^, ^18^, ^19^, ^20^ When compared with a previous effort to quantify the fatal burden of cancer attributable to risk factors globally, this study found a greater percentage of cancer deaths attributable to risk factors when estimating more risk factors (44·4% [95% UI 41·6–48·2] in 2001 in GBD 2019 compared with 35% in 2001 in the previous study), although both studies found leading contributions by smoking and alcohol use globally and unsafe sex in lower-income settings.^25^ Comparisons to comprehensive global risk-attributable cancer burden in the Global Cancer Observatory are not possible, as incidence estimates are provided only for individual risk categories, but for alcohol consumption and elevated BMI, risk factors estimated by both studies, similar estimates of risk-attributable cancer burden were noted (4·1% of new cancer cases in 2020 attributable to alcohol consumption in the Global Cancer Observatory and 4·9% [4·4–5·5] of cancer deaths in 2019 attributable to alcohol use in the GBD study; 3·6% of new cancer cases in 2012 attributable to high BMI in the Global Cancer Observatory and 4·6% [2·7–7·1] of cancer deaths in 2019 attributable to high BMI in the GBD study).^21^, ^22^ For cancer risk factors not included in this study, estimates from the Global Cancer Observatory suggest that an additional approximately 8·9% of cancer cases would be attributable to infections^23^ (appendix p 67) and an additional 1·2% of cancer cases would be attributable to ultraviolet radiation.^24^ These estimates should be interpreted with some caution given the different estimation approaches used, but might provide useful information for crucial remaining risk factors not yet included in the GBD study.

In GBD 2019, large all-age sex differences were seen in the global cancer burden attributable to all risk factors combined (48·0% [95% UI 45·3–51·5] of male cancer DALYs versus 34·3% [30·9–38·7] of female cancer DALYs). These sex differences are well documented, with several studies reporting higher attributable cancer burden in males compared with females.^13^, ^17^, ^18^, ^19^ In this study, we identified sex differences across two primary risk factor groupings. For instance, there were disparities in cancer DALYs attributable to behavioural risk factors, such as smoking (33·2% [31·7–34·7] for males *vs* 8·9% [8·3–9·6] for females) and alcohol use (7·4% [6·7–8·2] for males *vs* 2·3% [2·0–2·6] for females), which might be driven by higher exposure to these behavioural risk factors among males than females. Similarly, for environmental and occupational risks, for example, the cancer DALYs attributable to occupational carcinogens were three times higher among males (3·9% [3·1–4·8]) than females (1·3% [1·0–1·6]), which might reflect that males are more likely than females to be employed in workplaces with higher risk of exposure to carcinogens. Between 2010 and 2019, the change in global age-standardised risk-attributable cancer DALY rates decreased slightly among females (–4·6% [–11·0 to 2·2]), whereas there was a more notable decline among males (–9·6% [–17·6 to –1·3]). This result might suggest inequities in our approach to cancer prevention by sex and a need for future sex-specific assessments of effective cancer risk factor interventions.

Our results show a gradient across the sociodemographic spectrum in 2019, with the risk-attributable cancer age-standardised DALY rates generally increasing with higher SDI quintiles. However, from 2010 to 2019, age-standardised cancer DALY rates attributable to all risks combined declined in high, high-middle, and middle SDI countries, whereas these values increased in low-middle SDI countries or were approximately stable in low SDI countries. This increase was largely due to metabolic risks, which include risk factors such as high BMI. The growth in metabolic risk-attributable cancer burden might be the result of these countries experiencing an epidemiological transition in which improvements in country-level developmental status are related to increasing obesity levels.^36^, ^37^

Globally, there has been substantial progress in reducing exposure to tobacco that can be linked to coordinated international and national prevention efforts.^38^, ^39^ Interventions through taxation and regulatory policies for tobacco smoking, including smoke-free policies, increased tobacco taxes, and advertisement bans guided by the WHO Framework Convention on Tobacco Control, have played a major role in these efforts.^38^, ^40^ Similar efforts, including taxation and advertisement bans, have been recommended to help reduce the harmful use of alcohol.^41^, ^42^, ^43^ Behavioural risk factors are strongly influenced by the environment in which people live and individuals with cancer should not be blamed for their disease. Future research is needed to investigate the effect of population health approaches to cancer risk factor reduction that go beyond individual-oriented prevention and might be more effective long-term strategies than placing the onus on individuals to modify exposures to prevent cancer.^44^ Many risk factors for cancer have been well established for decades, but greater political commitment to implementing policies addressing cancer prevention is needed. Improving social determinants of health, such as access to education and reduction of poverty, might be a feasible approach to reducing exposure to certain risks across populations.^45^, ^46^ Population-based approaches aimed at improving social determinants of health might provide an equitable cancer control approach to overcome the systemic barriers promoting disproportionate risk-attributable cancer burden growth in some regions, countries, and subpopulations within countries. For these reasons, future research should not overlook the importance of context-specific interventions that are guided or led by those with an understanding of local cultural and behavioural patterns. Finally, cancers remain fundamentally linked to genetics and ageing, and although addressing contributing risk factors is crucial for cancer prevention, this will never eliminate cancer burden. As a result, countries should continue to invest in comprehensive cancer control strategies beyond risk factor reduction, which include health-care systems capable of early diagnosis, detection through screening for select cancers, and effective treatment options for those diagnosed with cancer.

Although GBD 2019 is the largest effort to date to estimate the global burden of cancer attributable to risk factors, there remain opportunities for improvement. First, some limitations are inherent in the data sources available. For instance, some countries do not have population-based cancer registries, which are an important data source for estimating cancer burden. As is apparent in the relative uncertainty of risk-attributable cancer burden estimated by GBD 2019 (appendix p 171), there is greater uncertainty relative to point estimates in many lower SDI countries as compared with higher SDI countries. GBD study models rely on available data, and estimates should not supplant but rather complement the ongoing crucial work to expand and improve directly observed data around the world. Cancer registry development and support are integral in cancer control efforts and should be considered in broader cancer control planning initiatives. Delays are inherent with the release of cancer registry and vital statistics reports, which result in more recent cancer mortality estimates often relying on historical data. The data used to estimate risk factor exposure is at times sparse and many data sources do not provide sufficient information to assess for potential measurement error or bias. Where there is information available, the GBD study aims to correct for systematic bias in risk exposure data by establishing a reference definition of each risk exposure and adjusting acceptable alternative exposure measurements on the basis of studies with observed data pairs of the two different definitions. However, after these adjustments, residual measurement bias is likely to persist and might vary around the world, over time, and by risk factor. Formal assessments of exposure model performance would be beneficial in future GBD iterations. Second, the risk factors included in this study are based on current knowledge of risk factors for cancer, but as knowledge expands there might be additional risk factors important to incorporate in future iterations of the GBD study. In addition, there are known risk factors for cancer, such as sunlight exposure (ie, ultraviolet radiation), and infectious agents, such as *Helicobacter pylori*, which are not included in the GBD study.^47^, ^48^ Unsafe sex is estimated as a risk factor, but human papillomavirus, a known risk factor for several cancer types, is not explicitly estimated; and although liver cancer burden due to hepatitis B and C is estimated within the GBD cause hierarchy, these viral infections are not estimated as risk factors, making their inclusion in robust risk-attributable cancer burden estimation challenging. Infection-associated cancers are more notable in lower SDI settings, so addressing these will be important to producing comprehensive global assessments of cancer-attributable risk and disparities. Third, second-order measures of cancer-relevant risk factors, including aspects such as income inequality and racism, would be challenging to comprehensively account for, but could add important context for future health policy work. Finally, GBD 2019 results were estimated before the COVID-19 pandemic. Evaluating the effect of the COVID-19 pandemic on risk-attributable cancer burden is an important area for future research. However, several leading risk factors identified in this study are also linked to an increase in the severity of illness in individuals with COVID-19 and to burden of other non-communicable diseases besides cancer. Thus, reducing exposure to these harmful risk factors might not only have a positive effect on cancer burden reduction efforts, but synergistically improve population health more broadly.

Worldwide, a large percentage of cancer deaths and DALYs were attributable to risk factors in 2019, with most being attributable to behavioural risks. Smoking continues to be the leading cancer risk factor globally, with other substantial contributors to cancer burden varying around the world. Targeting leading location-specific cancer risk factors might help countries make progress towards reducing non-communicable disease premature mortality by a third by 2030, as highlighted in SDG target 3.4. Although progress has been seen in high and high-middle SDI countries for behavioural and environmental and occupational risk-attributable cancer age-standardised DALY rates between 2010 and 2019, in low and low-middle SDI countries, metabolic risk-attributable cancer burden has grown considerably. Considerable cancer burden is not avoidable through the currently estimated risk factors, and, as such, countries should continue to simultaneously invest in risk reduction strategies while strengthening health systems to support early diagnosis and effective treatment of those with cancer. Given the increasing burden of cancer worldwide, this study can help policy makers and researchers identify important modifiable risk factors that could be targeted in efforts to reduce cancer burden globally, regionally, and nationally.

## Data sharing

This paper summarises key findings from our analysis of GBD 2019 estimates. Citations for the data used in this study can be accessed from the Global Health Data Exchange data input sources tool (http://ghdx.healthdata.org/gbd-2019/data-input-sources). Files containing all GBD 2019 estimates are available on the Global Health Data Exchange website (http://ghdx.healthdata.org/gbd-2019) and can also be downloaded from the Global Health Data Exchange results tool (http://healthdata.org/gbd-results-tool). Additional results can be explored through online interactive visualisations (https://vizhub.healthdata.org/gbd-compare/).

## Declaration of interests

R Ancuceanu reports consulting fees from AbbVie; payment or honoraria for lectures, presentations, speakers’ bureaus, manuscript writing, or educational events from AbbVie, Sandoz, B Braun, and Laropharm; all outside the submitted work. J Conde reports grants or contracts from European Research Council Starting Grant (ERC-StG-2019-848325; funding of €1·5 million); patents planned, issued, or pending for functionalised nanoparticles and compositions for cancer treatment and methods (US application number 62/334538), and TRPV2 antagonists (US patent application 17/590,061); all outside the submitted work. S Das reports grants or contracts from a Department of Science and Technology Grant for COVID-19 research; support for attending meetings or travel through the American Society of Clinical Pathology Travel Grant (US$1500); leadership or fiduciary role in other board, society, committee or advocacy group, paid or unpaid, with the American Association of Clinical Chemistry CME Committee, Personalised Division Committee, and as a HEA Committee member; all outside the submitted work. T R Driscoll reports leadership or fiduciary role in other board, society, committee, or advocacy group, unpaid, as Chair of the Occupational and Environmental Cancer Committee of Cancer Council Australia, Chair of the Australian Mesothelioma Registry Expert Advisory Group for the Australian Institute of Health and Welfare, and a member of the Research and Evaluation Committee of the Australian Safety and Eradication Agency, all outside the submitted work. H Elghazaly reports payment or honoraria for lectures, presentations, speakers bureaus, manuscript writing or educational events from Roche, BMS, Lilly, Pfizer, AstraZeneca, Janssen, MSD, Novartis, and Sandoz, all as personal payments; payment for expert testimony from Roche as personal payments; participation on a data safety monitoring board or advisory board for Roche, BMS, Lilly, Pfizer, AstraZeneca, Janssen, MSD, Novartis, Sandoz, including personal payments; all outside the submitted work. L M Force reports support for the present manuscript from the Bill & Melinda Gates Foundation; grants or contracts from St Baldrick's Foundation, St Jude Children's Research Hospital, and National Institutes of Health (NIH) Loan Repayment Program; leadership or fiduciary role in other board, society, committee, or advocacy group, unpaid, with *The Lancet Oncology* International Advisory Board; all outside the submitted work. N Ghith reports their salary is covered by a grant from Novo Nordisk Foundation (NNF16OC0021856), outside the submitted work. C Herteliu reports grants from the Romanian National Authority for Scientific Research and Innovation, CNDS-UEFISCDI (PN-III-P4-ID-PCCF-2016-0084, Oct, 2018, to Sep, 2022; and PN-III-P2-2.1-SOL-2020-2-0351, June–Oct, 2020), outside the submitted work. K Innos reports support for the present manuscript from Estonian Research Council, grant number PRG722 as payment to their institution. N E Ismail reports leadership or fiduciary role in other board, society, committee, or advocacy group, unpaid, as a Council Member for the Malaysian Academy of Pharmacy, outside the submitted work. J J Jozwiak reports payment or honoraria for lectures, presentations, speakers bureaus, manuscript writing or educational events from Teva, Amgen, Synexus, Boehringer Ingelheim, ALAB Laboratories, Zentiva, all as personal payments outside the submitted work. M Klugar reports grants Czech-Norwegian Collaboration on Meta-Research and Critical Thinking Education in Healthcare (EHP-CZ-ICP-2-009), Evidence Implementation in Clinical Practice (2020-1-DE01-KA203-005669), Towards an International Network for Evidence-based Research in Clinical Health Research in the Czech Republic (LTC20031), and Strategic Partnership in Innovation and Development of Evidence-Based Healthcare (2019-1-CZ01-KA202-061350), all as payments to their institution; participation on a data safety monitoring board or advisory board as an unpaid member of Cochrane advisory board for Evidence Advocacy; other non-financial interest as Director of Cochrane, JBI and GRADE Centres of the Czech Republic; all outside the submitted work. K Krishan reports non-financial support from UGC Centre of Advanced Study, CAS II, Department of Anthropology, Panjab University, Chandigarh, India, outside the submitted work. J A Loureiro reports support for the present manuscript from Scientific Employment Stimulus (FCT; CEECINST/00049/2018) as support to their salary and from UIDB/00511/2020 of the LEPABE, funded by national funds through the FCT/MCTES (PIDDAC) as research support. M Mahmoudi reports other financial and non-financial interest with Academic Parity Movement, a non-profit organisation dedicated to addressing academic discrimination, violence, and incivility as co-founder and director, Partners in Global Wound Care as founding partner, and receiving royalties or honoraria for his published books, plenary lectures, and licensed patent, all outside the submitted work. A-F A Mentis reports grants or contracts from “MilkSafe: a novel pipeline to enrich formula milk using omics technologies”, a research cofinanced by the European Regional Development Fund of the European Union and Greek national funds through the Operational Program Competitiveness, Entrepreneurship and Innovation, under the call RESEARCH—CREATE—INNOVATE (project code T2EDK-02222), as well as from ELIDEK (Hellenic Foundation for Research and Innovation, MIMS-860); stock or stock options in a family winery; other financial or non-financial interests as a scientific officer with BGI group; all outside the submitted work. S Mohammed reports support for the present manuscript from the Gates Foundation; a fellowship grant from Alexander von Humboldt Foundation, outside the submitted work. N Moka reports leadership or fiduciary role in other board, society, committee, or advocacy group, unpaid, with Kentucky Society of Clinical Oncology as treasurer, outside the submitted work. S B Munro reports stock or stock options in Invitae, and other financial or non-financial interests as an employee of Invitae, a genetics testing company. O O Odukoya reports support for the present manuscript from the Fogarty International Center of the National Institutes of Health under award number K43TW010704. The content is solely the responsibility of the authors and does not necessarily represent the official views of the National Institutes of Health. R G Pestell reports support for the present manuscript from W81XWH1810605 Breast Cancer Research Breakthrough Grant and R21 CA235139-01. NIH grant; patents issued and pending in the area of cancer diagnostics and treatment; participation on a data safety monitoring board or advisory board as a member of the VGI Health Technology Scientific Advisory Board, Chair Scientific Advisory Board for MD Anderson, and Cancer Center Breast Cancer SPORE Program; leadership or fiduciary role in other board, society, committee or advocacy group, paid or unpaid, as Founder and CEO of biotechnology companies LightSeed, EcoGenome and StromaGenesis; stock or stock options in CytoDyn and VGI Health Technology; all outside the submitted work. M J Postma reports stock or stock options in Health-Ecore (25%) and Pharmacoeconomics Advice Groningen (100%) outside the submitted work. A Radfar and I Filip report other financial or non-financial interest with Avicenna Medical and Clinical Research Institute, outside the submitted work. T J Roberts reports support for attending meetings or travel from Conquer Cancer Foundation; leadership or fiduciary role in other board, society, committee or advocacy group, paid or unpaid, with Biocon Biologics; all outside the submitted work. S Shrestha reports other financial or non-financial interests in the School of Pharmacy, Monash University Malaysia by receiving Graduate Research Merit Scholarship. L M L R Silva reports grants or contracts with the project code CENTRO-04-3559-FSE-000162, Fundo Social Europeu (FSE), outside the submitted work. J A Singh reports consulting fees from Crealta/Horizon, Medisys, Fidia, PK Med, Two Labs, Adept Field Solutions, Clinical Care options, Clearview healthcare partners, Putnam associates, Focus forward, Navigant consulting, Spherix, MedIQ, Jupiter Life, UBM, Trio Health, Medscape, WebMD, and Practice Point communications, and the National Institutes of Health and the American College of Rheumatology; payment or honoraria for participating in the speakers bureau for Simply Speaking; support for attending meetings or travel from the steering committee of OMERACT, to attend their meeting every 2 years; participation on a data safety monitoring board or advisory board as an unpaid member of the FDA Arthritis Advisory Committee; leadership or fiduciary role in other board, society, committee or advocacy group, paid or unpaid, as a member of the steering committee of OMERACT, an international organisation that develops measures for clinical trials and receives arms length funding from 12 pharmaceutical companies, with the Veterans Affairs Rheumatology Field Advisory Committee as Chair, and with the UAB Cochrane Musculoskeletal Group Satellite Center on Network Meta-analysis as a director and editor; stock or stock options in TPT Global Tech, Vaxart pharmaceuticals, Atyu Biopharma, Adaptimmune Therapeutics, GeoVax Labs, Pieris Pharmaceuticals, Enzolytics, Series Therapeutics, Tonix Pharmaceuticals, and Charlotte's Web Holdings and previously owned stock options in Amarin, Viking, and Moderna pharmaceuticals; all outside the submitted work.
