# Supplementary material for: The global burden of cancer attributable to risk factors, 2010–19: a systematic analysis for the Global Burden of Disease Study 2019
Source: Lancet. 2022 Aug 20;400(10352):563–91. doi: 10.1016/S0140-6736(22)01438-6 (PMC9395583; doi:10.1016/S0140-6736(22)01438-6)
Supplement: Supplementary appendix [file mmc1.pdf]

THE LANCET

Supplementary appendix

This appendix formed part of the original submission and has been peer reviewed. We post it as supplied by the authors.

Supplement to: GBD 2019 Cancer Risk Factors Collaborators. The global burden of cancer attributable to risk factors, 2010–19: a systematic analysis for the Global Burden of Disease Study 2019. *Lancet* 2022; **400:** 563–91.

Supplementary Appendix 1

This appendix formed part of the original submission and has been peer reviewed. We post it as supplied by the authors.

Supplement to: The global burden of cancer attributable to risk factors, 2010–2019: a systematic analysis for the Global Burden of Disease Study 2019. *Lancet* 2022;

Supplementary Appendix 1: Methods appendix to "The global burden of cancer attributable to risk factors, 2010–2019: a systematic analysis for the Global Burden of Disease Study 2019"

This appendix provides further methodological detail for "The global burden of cancer attributable to risk factors, 2010–2019: a systematic analysis for the Global Burden of Disease Study 2019". This study complies with the Guidelines for Accurate and Transparent Health Estimates Reporting (GATHER) recommendations.[5] It includes detailed tables and information on data to maximise transparency in our estimation processes and provides a comprehensive description of analytical steps. A completed GATHER checklist can be found on page 8 of this appendix.

Please note that portions of this supplement were copied from the supplementary content to the recent GBD publications:

Kocarnik J, Compton K, Dean FE, *et al*. Cancer incidence, mortality, years of life lost, years lived with disability, and disability-adjusted life years for 29 cancer groups from 2010 to 2019: a systematic analysis for the Global Burden of Disease Study 2019. *JAMA Oncol*. 2022;8(3):420-444. doi:10.1001/jamaoncol.2021.6987.[1] ;

Force LM, Abdollahpour I, Advani SM, *et al*. The global burden of childhood and adolescent cancer in 2017: an analysis of the Global Burden of Disease Study 2017. *The Lancet Oncology* 2019; 20: 1211–25.[2];

Vos T, Lim SS, Abbafati C, *et al*. Global burden of 369 diseases and injuries in 204 countries and territories, 1990–2019: a systematic analysis for the Global Burden of Disease Study 2019. *The Lancet* 2020; **396**: 1204–22.[3] ;

and

Murray CJL, Aravkin AY, Zheng P, *et al*. Global burden of 87 risk factors in 204 countries and territories, 1990–2019: a systematic analysis for the Global Burden of Disease Study 2019. *The Lancet* 2020; **396**: 1223–49.[4]

References are provided and renumbered for reproduced sections.

Table of Contents *3*

The Global Burden of Disease (GBD) Study **7**
GATHER Guidelines Checklist **8**
Definition of Indicator **10**
GBD Cancer Estimation Process **11**
Appendix Figure 1: Flowchart of GBD cancer mortality and Years of Life Lost (YLLs) estimation **11**
Appendix Figure 2: Flowchart of GBD cancer incidence and Years Lived with Disability (YLDs) estimation **12**

Data sources **13**
Cancer registry (CR) data sources **13**
Mortality-to-incidence (MIR) ratio data sources **13**
Cancer mortality data in the cause of death (CoD) database other than cancer registry data **13**
Bias of categories of input data **13**

Cancer types estimated in the GBD 2019 study **14**
ICD cancer codes mapped to GBD 2019 cancer causes **14**
Cancers in the GBD cause hierarchy **14**

Data analysis **14**
Cancer registry data processing **14**
Appendix Table 1: List of International Classification of Diseases (ICD) codes mapped to the Global Burden of Disease cause list for cancer incidence data **15**
Appendix Table 2: List of International Classification of Diseases (ICD) codes mapped to the Global Burden of Disease cause list for cancer mortality data **21**
Cause of death database formatting **31**
Appendix Table 3: Restrictions on age and sex by each cancer type in GBD 2019 **32**
CODEm models **32**
Appendix Table 4: GBD 2019 covariates and level of covariates used in cause of death modelling for cancer types estimated **33**
CoDCorrect **49**
Calculating YLLs **49**
Incidence estimation **49**
Prevalence estimation **49**
Appendix Table 5: Duration of four prevalence phases by cancer in GBD 2019 **50**
Appendix Table 6: Lay description of cancer states and corresponding disability weights in GBD 2019 **53**
Calculating DALYs **54**
Calculating Proportional Burden **54**
Reporting Standards **54**
Socio-demographic Index (SDI) Definition and Calculation **55**
Uncertainty Estimation **55**
Limitations **56**

GBD Risk Factor Estimation Process **57**
Appendix Figure 3: Analytical flowchart of the comparative risk assessment for the estimation of population attributable fractions by geography, age, sex, and year for GBD 2019 **57**
GBD risk factor hierarchy **58**
Risk factors data input sources overview **58**
Overview of risk factor estimation **58**

Uncertainty in Risk Factor Estimation 66
Calculation for infection-associated cancer burden not estimated by the GBD 2019 study 67
References 68

GBD risk-specific methods summaries 72
Ambient particulate matter pollution 73
Household air pollution from solid fuels 80
Radon exposure (residential radon) 85
Occupational risks 90
Smoking 96
Chewing tobacco 101
Secondhand smoke 104
Alcohol use 107
Drug use 115
Dietary risks 122
Unsafe sex 129
Low physical activity 131
High fasting plasma glucose 135
High body-mass index 139

Additional Methodology Tables and Figures 146
Appendix Figure 4: Socio-demographic Index quintiles for the Global Burden of Disease Study 2019 146
Appendix Table 7: SDI quintiles for countries estimated in GBD 2019 147
Appendix Figure 5: Map of GBD world super-regions, 2019 148
Appendix Figure 6: Map of GBD world regions, 2019 149
Appendix Table 8: GBD cause hierarchy with levels for all cancers 150
Appendix Table 9: GBD risk hierarchy with levels 152
Appendix Table 10: GBD 2019 Cancer risk-outcome pairs included in this analysis 154
Appendix Table 11: GBD 2019 risk factor hierarchy, exposure definition, theoretical minimum risk exposure level, and risk-cancer pairs included in GBD 2019 157

Additional Results in Tables and Figures 161
Appendix Figure 7: Global absolute and proportional cancer deaths attributable to Level 2 risk factors for (A) males and (B) females in 2019 161
Appendix Figure 8: DALYs from cancers attributable to risk factors in 2019 by sex and SDI 162
Appendix Figure 9: Age-standardised mortality rates from cancers attributable to risk factors in 2019 by sex and SDI 163
Appendix Figure 10: Age-standardised DALY rates from cancers attributable to risk factors in 2019 by sex and SDI 164
Appendix Figure 11: Global map of age-standardised mortality rate quintiles for cancer burden attributable to (A) environmental and occupational risks, (B) behavioural risks, and (C) metabolic risks, both sexes combined, 2019 165
Appendix Figure 12: Global map of proportion of risk-attributable cancer age-standardised DALY rates over total cancer age-standardised DALY rates, by quintile for cancer burden attributable to (A) environmental and occupational risks, (B) behavioural risks, and (C) metabolic risks, both sexes combined, 2019 167
Appendix Figure 13: Global map of proportion of risk-attributable cancer age-standardised mortality rates over total cancer age-standardised mortality rates, by quintile for cancer burden attributable to (A) environmental and occupational risks, (B) behavioural risks, and (C) metabolic risks, both sexes combined, 2019 169

Appendix Figure 14: Relative uncertainty in the percent of risk-attributable cancer age-standardised DALY rates over total cancer (risk + non-risk) age-standardised DALY rates, by quintile, for both sexes combined in 2019 **171**
Appendix Figure 15: Percent change of attributable A. cancer DALY counts and B. age-standardised DALY rates for Level 1 risk factors by GBD super-regions, both sexes combined, 2010 – 2019 **172**
Appendix Figure 16: Percent change of attributable cancer deaths and age-standardised mortality rates for Level 1 risk factors by GBD super-regions and SDI quintiles, both sexes combined, 2010 – 2019 **173**
Appendix Figure 17: Regional age-standardised rates of attributable cancer deaths and DALYs, both sexes, 2019 **174**
Appendix Figure 18: Leading risk factors at the most detailed level for attributable cancer age-standardised DALY rates, 2010-2019 for (A) the global level, (B) high SDI quintile, (C) high-middle SDI quintile, (D) middle SDI quintile, (E) low-middle SDI quintile, and (F) low SDI quintile, for males, females, and both sexes combined **175**
Appendix Figure 19: Leading risk factors at the most detailed level for attributable cancer age-standardised death rates, 2010-2019 for (A) the global level, (B) high SDI quintile, (C) high-middle SDI quintile, (D) middle SDI quintile, (E) low-middle SDI quintile, and (F) low SDI quintile, for both sexes combined, males, and females **181**
Appendix Figure 20: Level 1 risk-attributable age-standardised cancer DALY rates per 100,000, by SDI value for (A) males and (B) females **188**
Appendix Figure 21: Level 1 risk-attributable age-standardised cancer mortality rates per 100,000, by SDI value for (A) males and (B) females **189**
Appendix Figure 22: Trends in risk-attributable age-standardised cancer DALY rates, 1990-2019, for tobacco (left) and all risk factors estimated (right), by sex **190**
Appendix Table 12: Global risk-attributable cancer deaths and DALYs in males and females reported in all-age numbers, age-standardised rates, and percentages of total cancer deaths and DALYs in 2019 **191**
Appendix Table 13: Global risk-attributable cancer deaths and DALYs in males and females reported in all-age numbers, age-standardised rates, and percentages of risk-attributable cancer deaths and DALYs in 2019 for all risk-cancer pairs measured **194**
Appendix Table 14: Absolute and age-standardised deaths and DALYs attributable to risks assessed by cancer type in 2019, both sexes combined **200**
Appendix Table 15: Global deaths attributable vs. not attributable to risks assessed for each cancer type by sex in 2019 **206**
Appendix Table 16: Global age-standardised death rates attributable vs. not attributable to risks assessed for each cancer type by sex in 2019 **208**
Appendix Table 17: Percent of global risk-attributable deaths over total cancer risk-attributable deaths by sex in 2019 **210**
Appendix Table 18: Global risk-attributable deaths vs. total deaths for each cancer by sex in 2019 **211**
Appendix Table 19: Deaths attributable vs. not attributable to risks assessed for each cancer type by sex in 2019 in high SDI locations **213**
Appendix Table 20: Age-standardised death rates attributable vs. not attributable to risks assessed for each cancer type by sex in 2019 in high SDI locations **215**
Appendix Table 21: Deaths attributable vs. not attributable to risks assessed for each cancer type by sex in 2019 in non-high SDI locations **217**
Appendix Table 22: Age-standardised death rates attributable vs. not attributable to risks assessed for each cancer type by sex in 2019 in non-high SDI locations **219**
Appendix Table 23: Proportion of total cancer deaths vs. risk-attributable cancer deaths in high and non-high SDI settings in 2019, both sexes combined **221**

Appendix Table 24: Global percentages of risk-attributable cancer deaths and DALYs out of total cancer deaths and DALYs for both sexes, males, and females in 2019 **222**
Appendix Table 25: Change in age-standardised DALY rates and absolute DALYs from 2010 to 2019 for all risk factors combined by SDI quintile and GBD super-region, both sexes combined **223**
Appendix Table 26: Change in age-standardised DALY rates and absolute DALYs from 2010 to 2019 by SDI quintile and GBD super-region, both sexes combined **224**
Appendix Table 27: Change in age-standardised mortality rates and absolute deaths from 2010 to 2019 by SDI quintile and GBD super-region, both sexes combined **225**
Appendix Table 28: Percentage of cancer deaths, age-standardised mortality rate, DALYs, and age-standardised mortality rate attributable to risks over total cancer deaths and DALYs in 2019, both sexes combined, by country **226**
Appendix Table 29: Attributable cancer deaths and DALYs in 2019 and percentage change of age-standardised death rates and DALY rates, 2010-2019 for all regions, countries, and territories **234**
Appendix Table 30: Global numbers and age-standardised rates of risk-attributable total cancer deaths and DALYs, 2010 and 2019, and percentage change in global numbers and age-standardised rates of risk-attributable total cancer deaths and cancer DALYs, 2010-2019, both sexes combined **240**
Appendix Table 31: Global numbers and age-standardised rates of risk-attributable total cancer deaths and DALYs, 2010 and 2019, and percentage change in global numbers and age-standardised rates of risk-attributable total cancer deaths and cancer DALYs, 2010-2019, males **243**
Appendix Table 32: Global numbers and age-standardised rates of risk-attributable total cancer deaths and DALYs, 2010 and 2019, and percentage change in global numbers and age-standardised rates of risk-attributable total cancer deaths and cancer DALYs, 2010-2019, females **246**
Appendix Table 33: Global numbers and age-standardised rates of attributable cancer deaths and DALYs, 2010 and 2019, and percentage change in global numbers and age-standardised rates of attributable cancer deaths and cancer DALYs, 2010-2019, for all risk-cancer pairs measured, both sexes combined **248**
Appendix Table 34: Comparison of individual country studies and GBD 2019 study population-attributable fraction estimates **254**

Additional Results in Tables and Figures **260**

The Global Burden of Disease (GBD) study

The Global Burden of Disease (GBD) study was created in an effort to establish comprehensive and comparable health metrics. A key principle in the GBD approach to estimation of disease burden is that an individual can have only one cause of death, while recognising that this may underestimate disease burden due to intermediate causes of death. In addition to reporting estimates of mortality and years of life lost (YLLs) for over 300 diseases and injuries, the GBD study also quantifies non-fatal components of disease including years lived with disability (YLDs) and disability-adjusted life-years (DALYs), a metric that represents a combination of both the fatal and non-fatal components of disease. The GBD approach uses all relevant data sources, rather than a single type of data. Finally, as there is continual methodological refinement with each GBD iteration, the results in each successive iteration supersede the results of prior GBD studies for the entire newly estimated time series. A protocol for the GBD study can be found online at http://www.healthdata.org/sites/default/files/files/Projects/GBD/GBD_Protocol.pdf.

GATHER[5] Guidelines Checklist

Item #	Checklist item	Reported on page #
**Objectives and funding**		
**1**	Define the indicator(s), populations (including age, sex, and geographic entities), and time period(s) for which estimates were made.	Appendix pg. 10
**2**	List the funding sources for the work.	See main manuscript
**Data Inputs**		
For all data inputs from multiple sources that are synthesised as part of the study:		
**3**	Describe how the data were identified and how the data were accessed.	Appendix pg. 13
**4**	Specify the inclusion and exclusion criteria. Identify all ad-hoc exclusions.	Appendix pg. 13
**5**	Provide information on all included data sources and their main characteristics. For each data source used, report reference information or contact name/institution, population represented, data collection method, year(s) of data collection, sex and age range, diagnostic criteria or measurement method, and sample size, as relevant.	http://ghdx.healthdata.org/gbd-2019
**6**	Identify and describe any categories of input data that have potentially important biases (eg, based on characteristics listed in item 5).	Appendix pg. 13
For data inputs that contribute to the analysis but were not synthesised as part of the study:		
**7**	Describe and give sources for any other data inputs.	http://ghdx.healthdata.org/gbd-2019
For all data inputs:		
**8**	Provide all data inputs in a file format from which data can be efficiently extracted (eg, a spreadsheet rather than a PDF), including all relevant meta-data listed in item 5. For any data inputs that cannot be shared because of ethical or legal reasons, such as third-party ownership, provide a contact name or the name of the institution that retains the right to the data.	http://ghdx.healthdata.org/gbd-2019
**Data analysis**		
**9**	Provide a conceptual overview of the data analysis method. A diagram may be helpful.	Appendix pg. 11–12 (Appendix Figures 1 & 2)
**10**	Provide a detailed description of all steps of the analysis, including mathematical formulae. This description should cover, as relevant, data cleaning, data pre-processing, data adjustments and weighting of data sources, and mathematical or statistical model(s).	Appendix pg. 13–145
**11**	Describe how candidate models were evaluated and how the final model(s) were selected.	Found in *Section 3: Causes of death modelling methods* of the Supplementary appendix 1 to "GBD 2019 Diseases and Injuries Collaborators. Global burden of 369 diseases and injuries in 204 countries and territories, 1990–2019: a systematic analysis for the Global Burden of Disease Study

		2019”.[3] Details of covariate selection for cancer models can be found in: Appendix pg. 33–48 (Appendix Table 4)
**12**	Provide the results of an evaluation of model performance, if done, as well as the results of any relevant sensitivity analysis.	Found in eTable 10 of the Supplementary Appendix to “Morbidity and mortality for 29 cancer groups by country and territory and Socio-demographic Index, 1990-2019: a systematic analysis for the Global Burden of Disease Study 2019”.
**13**	Describe methods for calculating uncertainty of the estimates. State which sources of uncertainty were, and were not, accounted for in the uncertainty analysis.	Appendix pg. 55 (uncertainty in cancer estimation); pg. 66 (uncertainty in the general risk factor estimation process), and pg. 72–145 (uncertainty in estimation of specific risk factors)
**14**	State how analytic or statistical source code used to generate estimates can be accessed.	http://ghdx.healthdata.org/gbd-2019/code
**Results and Discussion**		
**15**	Provide published estimates in a file format from which data can be efficiently extracted.	GBD 2019 estimates are available online (https://vizhub.healthdata.org/gbd-compare/ and http://ghdx.healthdata.org/gbd-results-tool)
**16**	Report a quantitative measure of the uncertainty of the estimates (eg, uncertainty intervals).	See main manuscript, “Results”
**17**	Interpret results in light of existing evidence. If updating a previous set of estimates, describe the reasons for changes in estimates.	See main manuscript, “Discussion”
**18**	Discuss limitations of the estimates. Include a discussion of any modelling assumptions or data limitations that affect interpretation of the estimates.	Appendix pg. 56

Definition of Indicator

In this publication, estimates are presented for 82 cancer risk-outcome pairs, representing 23 cancer groups and 34 risk factor groups, for both sexes, for the time period 2010 to 2019 and for five-year GBD age groups (such as 10-14, 15-19, 20-24, etc. until 95+). These estimates are presented globally and for regions which include 204 countries or territories. All ICD-9 codes pertaining to cancer (140-209) and ICD-10 codes (C00-C96) except for non-melanoma skin cancer (ICD-10: C44) and the majority of Kaposi sarcoma (ICD-10: C46) are included in these estimates (see section “5. Cause disaggregation” on pg. 29 of this appendix for more information on Kaposi sarcoma code handling in GBD). Leukaemias were assessed at the parent level for this analysis (more specific leukaemia subtypes were aggregated to parent in results tables and figures). For a complete list of ICD codes and their respective GBD causes, refer to Appendix tables 1 & 2. The Global Burden of Disease 2019 analysis estimates mortality and morbidity across more cancer groups than are highlighted in this analysis because not all GBD cancer causes have risk factors currently estimated for them within the GBD study. For completeness and accuracy, the cancer estimation methods described in this appendix at times reference cancers outside the scope of this paper, however the results (both in the manuscript and in this appendix) focus only on the 23 cancer groups with associated risk factors found in Appendix Table 8.

A complete list of countries and territories estimated in GBD 2019 can be found in “Table S3: GBD location hierarchy with levels” on page 1459 in Supplementary Appendix 1 to “Global burden of 369 diseases and injuries in 204 countries and territories, 1990–2019: a systematic analysis for the global burden of disease study 2019”.[3] We recognise that location-specific results are helpful; although limited country-specific results are reported in this analysis, more detailed estimates can be found in the GBD Results tool, http://ghdx.healthdata.org/gbd-results-tool, and the GBD Compare tool, https://vizhub.healthdata.org/gbd-compare/.

GBD Cancer Estimation Process

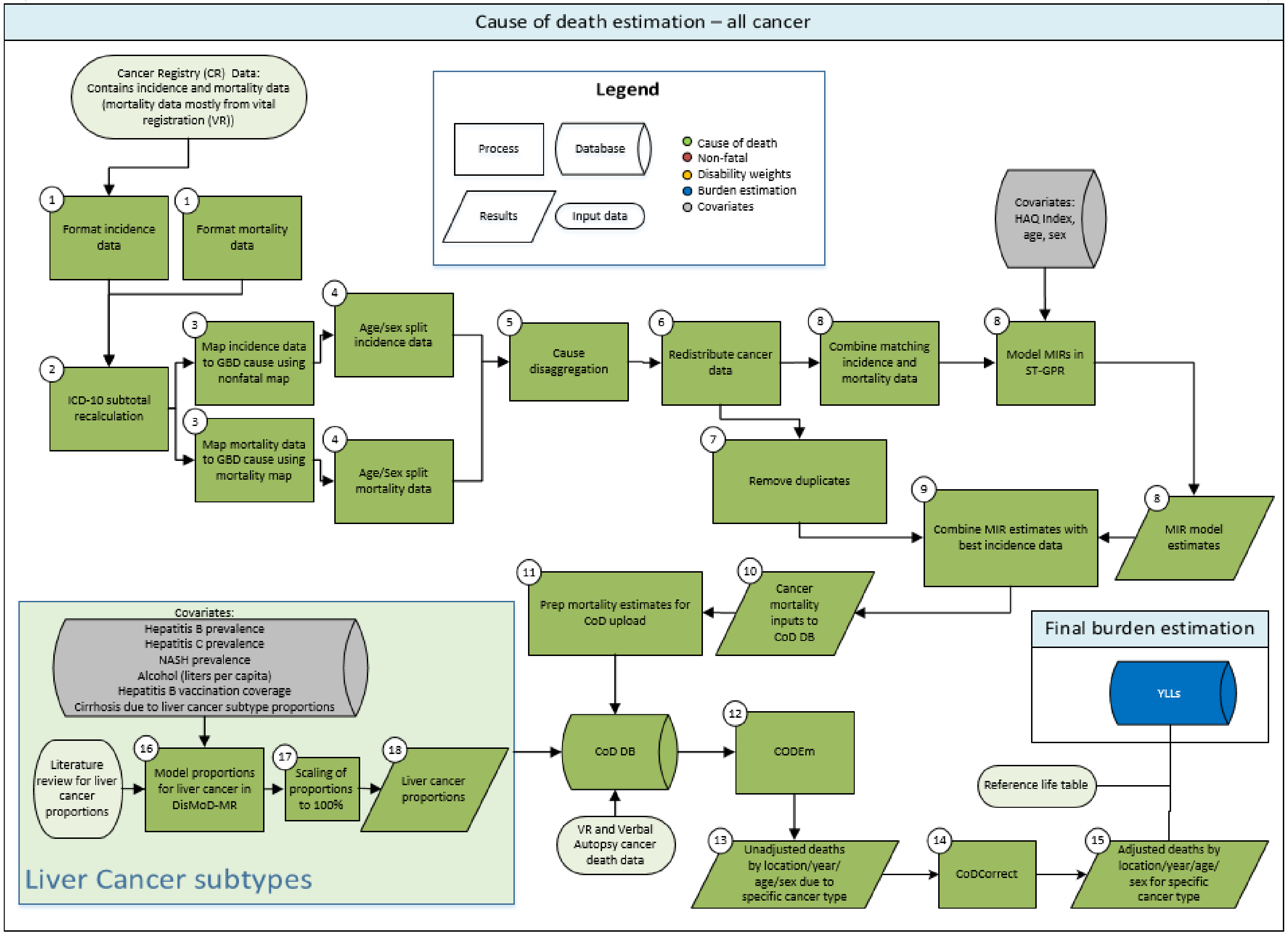

Appendix Figure 1: Flowchart of GBD cancer mortality and Years of Life Lost (YLLs) estimation. Abbreviations: CoD, causes of death; CODEm, cause of death ensemble model; DB, database; DisMod-MR, disease model - Bayesian meta-regression; HAQ Index, Healthcare Access and Quality Index; ICD, International Classification of Diseases; ST-GPR, spaciotemporal Gaussian process regression; MIR, mortality-to-incidence ratio; NASH, nonalcoholic steatohepatitis; VR, vital registration; YLL, years of life lost.

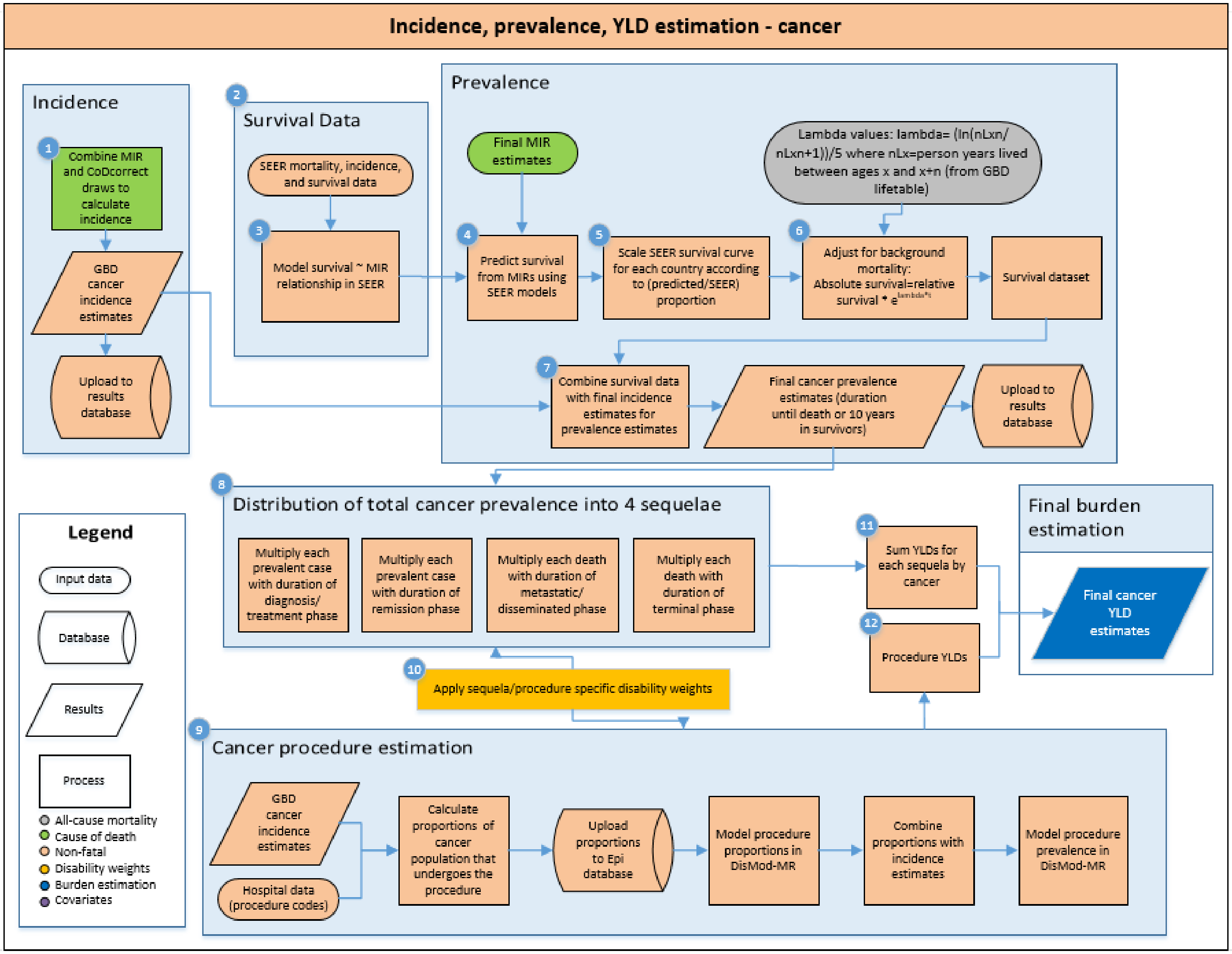

Appendix Figure 2: Flowchart of GBD cancer incidence and Years Lived with Disability (YLDs) estimation. Abbreviations: GBD, Global Burden of Disease Study; MIR, mortality-to-incidence ratio; SEER, Surveillance, Epidemiology and End Results Program; YLD, years lived with disability.

Data sources

Cancer registry (CR) data sources

Cancer incidence and mortality data were sought from individual cancer registries, such as the Surveillance, Epidemiology, and End Results (SEER) Program[6]; provided by collaborators; or downloaded from aggregated databases of cancer registry data such as "Cancer Incidence In Five Continents" (CI5)",[7–17] EUREG,[18] or NORDCAN.[19] Only population-based cancer registries were included, with inclusion criteria that they included all cancers (ie, were not specialty registries), reported data for all age groups (except for pediatric cancer registries), and reported data for both sexes. Pathology-based cancer registries were included if they had a defined population. Hospital-based cancer registries were excluded. Redundant cancer registry data were excluded from either the final incidence data input or the MIR model input if a more detailed source (eg, providing more detailed age or diagnostic groups) was available for the same population. Preference was given to registries with national coverage over those with only local coverage, except those from countries where the GBD study provides subnational estimates. Data were excluded if the coverage population was unknown, except for in high SDI quintile locations with full geographic coverage where the GBD estimated population could be substituted. A list of the cancer registries included in our analysis and the years covered can be found in the online GBD citation tool http://ghdx.healthdata.org/gbd-2019. Additionally, CR data sources can be found in eTable 6 of the Supplementary appendix to "Cancer Incidence, Mortality, Years of Life Lost, Years Lived with Disability, and Disability-Adjusted Life Years for 29 Cancer Groups from 2010 to 2019: A Systematic Analysis of Cancer Burden Globally, Nationally, and by Socio-demographic Index for the Global Burden of Disease Study 2019".[1]

Mortality-to-incidence ratio (MIR) data sources

Most cancer registries only report cancer incidence. However, if a cancer registry also reported cancer mortality, mortality data were also extracted. CR sources with matching incidence and mortality data were used in the mortality-to-incidence ratio estimation.[1]

Cancer mortality data in the cause of death (CoD) database other than cancer registry data

In addition to cancer registry data, the GBD cause of death (CoD) database also contains cancer mortality data originating from multiple sources, including vital registration (VR) and verbal autopsy (VA) data. In countries without VR systems, VA studies are a viable data source to inform CoD. VA data are obtained by trained interviewers who use a standardised questionnaire to ask relatives about the signs, symptoms, and demographic characteristics of recently deceased family members. CoD is assigned based on the answers to the questionnaires. A detailed description of the data sources and processing steps for the cause of death database can be found in Supplementary Appendix 1 to the GBD 2019 paper "Global burden of 369 diseases and injuries in 204 countries and territories, 1990–2019: a systematic analysis for the Global Burden of Disease Study 2019",[3] as well as in the online GBD citation tool http://ghdx.healthdata.org/gbd-2019.

Bias of categories of input data

Potential biases of the input data included for the CoD database can also be found in the Supplementary Appendix 1 to the GBD 2019 paper "Global burden of 369 diseases and injuries in 204 countries and territories, 1990–2019: a systematic analysis for the Global Burden of Disease Study 2019".[3] Cancer registry data can be biased in multiple ways. A high proportion of ill-defined cancer cases in the cancer registry data requires redistribution of these cases to other cancers, which introduces a potential for bias. Changes between coding systems can lead to artificial differences in disease estimates; however, we adjust for this bias by mapping the different coding systems to GBD cancer causes. Underreporting of cancers that require advanced diagnostic techniques (eg, leukaemia, brain, pancreatic, and liver cancer) can be an issue in cancer registries from low-income countries. On the other hand, misclassification of

metastatic sites as primary cancer can lead to overestimation of cancer sites that are common sites for metastases (eg, brain cancer). Since many cancer registries are located in urban areas, the representativeness of the registry for the general non-urban population can also be problematic. The accuracy of mortality data reported in cancer registries usually depends on the quality of the vital registration system. If the vital registration system is incomplete or of poor quality, the mortality-to-incidence ratio can be biased to lower ratios.

<u>Cancer types estimated in the GBD 2019 study</u>

ICD cancer codes mapped to GBD 2019 cancer causes

Please refer to Appendix tables 1 & 2 in this appendix in section "3. Mapping data to GBD causes" for a list of International Classification of Diseases (ICD) codes mapped to the Global Burden of Disease cause list for causes of death.

Cancers in the GBD cause hierarchy

The Global Burden of Disease (GBD) cause list is organised in a hierarchy. Levels 1 and 2 represent general groupings of causes, while Levels 3 and 4 represent increasingly specific causes. The general Level 1 group "Non-communicable diseases" includes the broad Level 2 group "Total Cancers", which includes all malignant neoplasms. Level 3 represents specific site-based cancers, such as "Stomach cancer" and "Liver cancer", and some Level 3 cancers are further subdivided into Level 4 subtypes (ie, Level 3 "Leukaemia" is divided into Level 4 causes "Acute lymphoid leukaemia", "Chronic lymphoid leukaemia", "Acute myeloid leukaemia", "Chronic myeloid leukaemia", and "Other leukaemia"). Level 4 cancer causes are not included in this analysis; only Level 2 ("Total Cancers") and Level 3 cancer groups are presented in this paper.

<u>Data analysis</u>

Cancer registry data processing

Cancer registry data goes through multiple processing steps before entering the CoD database.

1. Formatting incidence and mortality data. First, the original data are transformed into standardised files, which included standardisation of format, categorisation, and registry names (#1 in Appendix figure 1).

2. Subtotal recalculation. Some cancer registries report individual codes as well as aggregated totals. An example of this would be where the registry data reports C18, C19, and C20 individually, and also the aggregated group of C18–C20 (colon and rectum cancer). The data processing step, "subtotal recalculation" (#2 in Appendix figure 1), verifies these totals and subtracts the values of any individual codes from the aggregates.

3. Mapping data to GBD causes. In the third step (#3 in in Appendix figure 1), cancer registry incidence data and cancer registry mortality data are mapped to GBD causes. A different map is used for incidence and for mortality data because of the assumption that there are no deaths for certain cancers. One example is benign or in situ neoplasms. Because cancer registries do not collect non-malignant neoplasms in a standardised way, any benign or in situ neoplasms reported in a cancer registry incidence dataset are dropped from that dataset. The same neoplasms reported in a cancer registry mortality dataset are instead mapped to the respective invasive cancer. For example, cases of "ductal carcinoma in situ" in a cancer registry incidence dataset are dropped from the dataset, while deaths from "ductal carcinoma in situ" in a cancer registry mortality dataset are mapped to breast cancer. Maps of ICD-codes to GBD causes for incidence and mortality data can be found in Appendix tables 1 & 2.

Appendix Table 1: List of International Classification of Diseases (ICD) codes mapped to the Global Burden of Disease cause list for cancer incidence data

Cause	ICCC3	ICD-10	ICD-9
Lip and oral cavity cancer	XIf1	C00, C00.0, C00.1, C00.2, C00.3, C00.4, C00.5, C00.6, C00.8, C00.9, C01, C01.9, C02, C02.0, C02.1, C02.2, C02.3, C02.4, C02.8, C02.9, C03, C03.0, C03.1, C03.9, C04, C04.0, C04.1, C04.8, C04.9, C05, C05.0, C05.1, C05.2, C05.8, C05.9, C06, C06.0, C06.1, C06.2, C06.8, C06.80, C06.89, C06.9, C07, C07.0, C07.9, C08, C08.0, C08.1, C08.8, C08.9	140, 140.0, 140.1, 140.2, 140.3, 140.4, 140.5, 140.6, 140.7, 140.8, 140.9, 141, 141.0, 141.1, 141.2, 141.3, 141.4, 141.5, 141.6, 141.8, 141.9, 142, 142.0, 142.1, 142.2, 142.3, 142.8, 142.9, 143, 143.0, 143.1, 143.8, 143.9, 144, 144.0, 144.1, 144.4, 144.8, 144.9, 145, 145.0, 145.1, 145.2, 145.3, 145.4, 145.5, 145.6, 145.8, 145.9
Nasopharynx cancer	XIc	C11, C11.0, C11.1, C11.2, C11.3, C11.8, C11.9	147, 147.0, 147.1, 147.2, 147.3, 147.8, 147.9
Other pharynx cancer	NA	C09, C09.0, C09.1, C09.8, C09.9, C1, C10, C10.0, C10.1, C10.2, C10.3, C10.4, C10.8, C10.9, C12, C12.0, C12.9, C13, C13.0, C13.1, C13.2, C13.8, C13.9	146, 146.0, 146.1, 146.2, 146.3, 146.4, 146.5, 146.6, 146.7, 146.8, 146.9, 148, 148.0, 148.1, 148.2, 148.3, 148.4, 148.5, 148.8, 148.9
Oesophageal cancer	NA	C15, C15.0, C15.1, C15.2, C15.3, C15.4, C15.5, C15.8, C15.9	150, 150.0, 150.1, 150.2, 150.3, 150.4, 150.5, 150.6, 150.7, 150.8, 150.9
Stomach cancer	NA	C16, C16.0, C16.1, C16.2, C16.3, C16.4, C16.5, C16.6, C16.7, C16.8, C16.9	151, 151.0, 151.1, 151.2, 151.3, 151.4, 151.5, 151.6, 151.8, 151.9, 209.23
Colon and rectum cancer	XIf2, XIf3	C18, C18.0, C18.1, C18.2, C18.3, C18.4, C18.5, C18.6, C18.7, C18.8, C18.9, C19, C19.0, C19.9, C2, C20, C20.0, C20.8, C20.9, C21, C21.0, C21.1, C21.2, C21.8, C21.9	153, 153.0, 153.1, 153.2, 153.3, 153.4, 153.5, 153.6, 153.7, 153.8, 153.9, 154, 154.0, 154.1, 154.2, 154.3, 154.4, 154.8, 154.9, 209.1, 209.10, 209.11, 209.12, 209.13, 209.14, 209.15, 209.16, 209.17, 569.0, 569.43, 569.44, 569.84, 569.85
Liver cancer	VIIb, VIIc	C22, C22.0, C22.1, C22.3, C22.4, C22.5, C22.7, C22.8	155, 155.0, 155.1, 155.3, 155.5, 155.9
Gallbladder and biliary tract cancer	NA	C23, C23.0, C23.9, C24, C24.0, C24.1, C24.4, C24.8, C24.9	156, 156.0, 156.1, 156.2, 156.3, 156.8, 156.9
Pancreatic cancer	XIIa2	C25, C25.0, C25.1, C25.2, C25.3, C25.4, C25.7, C25.8, C25.9	157, 157.0, 157.1, 157.2, 157.3, 157.4, 157.5, 157.7, 157.8, 157.9
Larynx cancer	NA	C32, C32.0, C32.1, C32.2, C32.3, C32.8, C32.9	161, 161.0, 161.1, 161.2, 161.3, 161.8, 161.9

Cause	ICCC3	ICD-10	ICD-9
Tracheal, bronchus, and lung cancer	XIIa3, XIf4	C33, C33.0, C33.2, C33.9, C34, C34.0, C34.00, C34.01, C34.02, C34.1, C34.10, C34.11, C34.12, C34.2, C34.3, C34.30, C34.31, C34.32, C34.4, C34.7, C34.8, C34.80, C34.81, C34.82, C34.9, C34.90, C34.91, C34.92	162, 162.0, 162.1, 162.2, 162.3, 162.4, 162.5, 162.8, 162.9, 209.21
Malignant skin melanoma*	XId	C43, C43.0, C43.1, C43.10, C43.11, C43.12, C43.2, C43.20, C43.21, C43.22, C43.3, C43.30, C43.31, C43.39, C43.4, C43.5, C43.51, C43.52, C43.59, C43.6, C43.60, C43.61, C43.62, C43.7, C43.70, C43.71, C43.72, C43.8, C43.9	172, 172.0, 172.1, 172.2, 172.3, 172.4, 172.5, 172.6, 172.7, 172.8, 172.9
Breast cancer	XIf6	C50, C50.0, C50.01, C50.011, C50.012, C50.019, C50.02, C50.021, C50.022, C50.029, C50.1, C50.11, C50.111, C50.112, C50.119, C50.12, C50.121, C50.122, C50.129, C50.2, C50.21, C50.211, C50.212, C50.219, C50.22, C50.221, C50.222, C50.229, C50.3, C50.31, C50.311, C50.312, C50.319, C50.32, C50.321, C50.322, C50.329, C50.4, C50.41, C50.411, C50.412, C50.419, C50.42, C50.421, C50.422, C50.429, C50.5, C50.51, C50.511, C50.512, C50.519, C50.52, C50.521, C50.522, C50.529, C50.6, C50.61, C50.611, C50.612, C50.619, C50.62, C50.621, C50.622, C50.629, C50.7, C50.8, C50.81, C50.811, C50.812, C50.819, C50.82, C50.821, C50.822, C50.829, C50.9, C50.91, C50.911, C50.912, C50.919, C50.92, C50.921, C50.922, C50.929	174, 174.0, 174.1, 174.2, 174.3, 174.4, 174.5, 174.6, 174.8, 174.9, 175, 175.0, 175.3, 175.9, 610, 610.0, 610.1, 610.2, 610.3, 610.4, 610.8, 610.9
Cervical cancer	XIf7	C53, C53.0, C53.1, C53.3, C53.4, C53.8, C53.9	180, 180.0, 180.1, 180.2, 180.3, 180.4, 180.5, 180.6, 180.8, 180.9, 622.1, 622.10, 622.11, 622.12, 622.2, 622.7
Uterine cancer	NA	C54, C54.0, C54.1, C54.2, C54.3, C54.4, C54.8, C54.9	182, 182.0, 182.1, 182.8, 182.9
Ovarian cancer	NA	C56, C56.0, C56.1, C56.2, C56.4, C56.9	183, 183.0
Prostate cancer	NA	C61, C61.0, C61.9	185, 185.0, 185.9

Cause	ICCC3	ICD-10	ICD-9
Testicular cancer*	NA	C62, C62.0, C62.00, C62.01, C62.02, C62.1, C62.10, C62.11, C62.12, C62.9, C62.90, C62.91, C62.92	186, 186.0, 186.9
Kidney cancer	VI, VIa, VIa1, VIa2, VIa3, VIa4, VIb, VIc	C64, C64.0, C64.1, C64.2, C64.4, C64.5, C64.6, C64.8, C64.9, C65, C65.0, C65.1, C65.2, C65.9	189.0, 189.1, 189.5, 189.6, 209.24
Bladder cancer	XIf8	C67, C67.0, C67.1, C67.2, C67.3, C67.4, C67.5, C67.6, C67.7, C67.8, C67.9	188, 188.0, 188.1, 188.2, 188.3, 188.4, 188.5, 188.6, 188.7, 188.8, 188.9
Brain and central nervous system cancer*	III, IIIa, IIIa1, IIIa2, IIIb, IIIc, IIIc1, IIIc2, IIIc3, IIIc4, IIId, IIId1, IIId2, IIId3, IIIe, IIIe1, IIIe2, IIIe3, IIIe4, IIIe5, IIIf, Xa, Xa1, Xa2, Xa3, Xa4, Xa5, Xa6	C70, C70.0, C70.1, C70.5, C70.6, C70.9, C71, C71.0, C71.1, C71.2, C71.3, C71.4, C71.5, C71.6, C71.7, C71.8, C71.9, C72, C72.0, C72.1, C72.2, C72.20, C72.21, C72.22, C72.3, C72.30, C72.31, C72.32, C72.4, C72.40, C72.41, C72.42, C72.5, C72.50, C72.59, C72.8, C72.9	191, 191.0, 191.1, 191.2, 191.3, 191.4, 191.5, 191.6, 191.7, 191.8, 191.9, 192, 192.0, 192.1, 192.2, 192.3, 192.4, 192.8, 192.9
Thyroid cancer	XIb	C73, C73.0, C73.1, C73.2, C73.3, C73.4, C73.5, C73.8, C73.9	193, 193.0, 193.2, 193.9
Mesothelioma	XIIa5	C45, C45.0, C45.1, C45.2, C45.3, C45.4, C45.5, C45.6, C45.7, C45.8, C45.9	NA
Hodgkin lymphoma*	IIa	C81, C81.0, C81.00, C81.01, C81.02, C81.03, C81.04, C81.05, C81.06, C81.07, C81.08, C81.09, C81.1, C81.10, C81.11, C81.12, C81.13, C81.14, C81.15, C81.16, C81.17, C81.18, C81.19, C81.2, C81.20, C81.21, C81.22, C81.23, C81.24, C81.25, C81.26, C81.27, C81.28, C81.29, C81.3, C81.30, C81.31, C81.32, C81.33, C81.34, C81.35, C81.36, C81.37, C81.38, C81.39, C81.4, C81.40, C81.41, C81.42, C81.43, C81.44, C81.45, C81.46, C81.47, C81.48, C81.49, C81.5, C81.6, C81.7, C81.70, C81.71, C81.72, C81.73, C81.74, C81.75, C81.76, C81.77, C81.78, C81.79, C81.8, C81.9, C81.90, C81.91, C81.92, C81.93, C81.94, C81.95, C81.96, C81.97, C81.98, C81.99	201, 201.0, 201.00, 201.01, 201.02, 201.03, 201.04, 201.05, 201.06, 201.07, 201.08, 201.1, 201.10, 201.11, 201.12, 201.13, 201.14, 201.15, 201.16, 201.17, 201.18, 201.2, 201.20, 201.21, 201.22, 201.23, 201.24, 201.25, 201.26, 201.27, 201.28, 201.4, 201.40, 201.41, 201.42, 201.43, 201.44, 201.45, 201.46, 201.47, 201.48, 201.5, 201.50, 201.51, 201.52, 201.53, 201.54, 201.55, 201.56, 201.57, 201.58, 201.6, 201.60, 201.61, 201.62, 201.63, 201.64, 201.65, 201.66, 201.67, 201.68, 201.7, 201.70, 201.71, 201.72, 201.73, 201.74, 201.75, 201.76, 201.77, 201.78, 201.9, 201.90, 201.91, 201.92, 201.93, 201.94, 201.95, 201.96, 201.97, 201.98

Cause	ICCC3	ICD-10	ICD-9
Non-Hodgkin lymphoma	IIb, IIb1, IIb2, IIb3, IIb4, IIc, IId, IIe	C83.7, C83.70, C83.71, C83.72, C83.73, C83.74, C83.75, C83.76, C83.77, C83.78, C83.79, C83.8, C82, C82.0, C82.00, C82.01, C82.02, C82.03, C82.04, C82.05, C82.06, C82.07, C82.08, C82.09, C82.1, C82.10, C82.11, C82.12, C82.13, C82.14, C82.15, C82.16, C82.17, C82.18, C82.19, C82.2, C82.20, C82.21, C82.22, C82.23, C82.24, C82.25, C82.26, C82.27, C82.28, C82.29, C82.3, C82.30, C82.31, C82.32, C82.33, C82.34, C82.35, C82.36, C82.37, C82.38, C82.39, C82.4, C82.40, C82.41, C82.42, C82.43, C82.44, C82.45, C82.46, C82.47, C82.48, C82.49, C82.5, C82.50, C82.51, C82.52, C82.53, C82.54, C82.55, C82.56, C82.57, C82.58, C82.59, C82.6, C82.60, C82.61, C82.62, C82.63, C82.64, C82.65, C82.66, C82.67, C82.68, C82.69, C82.7, C82.8, C82.80, C82.81, C82.82, C82.83, C82.84, C82.85, C82.86, C82.87, C82.88, C82.89, C82.9, C82.90, C82.91, C82.92, C82.93, C82.94, C82.95, C82.96, C82.97, C82.98, C82.99, C83, C83.0, C83.00, C83.01, C83.02, C83.03, C83.04, C83.05, C83.06, C83.07, C83.08, C83.09, C83.1, C83.10, C83.11, C83.12, C83.13, C83.14, C83.15, C83.16, C83.17, C83.18, C83.19, C83.2, C83.3, C83.30, C83.31, C83.32, C83.33, C83.34, C83.35, C83.36, C83.37, C83.38, C83.39, C83.4, C83.5, C83.50, C83.51, C83.52, C83.53, C83.54, C83.55, C83.56, C83.57, C83.58, C83.59, C83.6, C83.80, C83.81, C83.82, C83.83, C83.84, C83.85, C83.86, C83.87, C83.88, C83.89, C83.9, C83.90, C83.91, C83.92, C83.93, C83.94, C83.95, C83.96, C83.97, C83.98, C83.99, C84, C84.0, C84.00, C84.01, C84.02, C84.03, C84.04, C84.05, C84.06, C84.07, C84.08, C84.09, C84.1, C84.10, C84.11, C84.12, C84.13, C84.14, C84.15, C84.16, C84.17, C84.18, C84.19, C84.2, C84.3,	200.2, 200.20, 200.21, 200.22, 200.23, 200.24, 200.25, 200.26, 200.27, 200.28, 200, 200.0, 200.00, 200.01, 200.02, 200.03, 200.04, 200.05, 200.06, 200.07, 200.08, 200.1, 200.10, 200.11, 200.12, 200.13, 200.14, 200.15, 200.16, 200.17, 200.18, 200.3, 200.30, 200.31, 200.32, 200.33, 200.34, 200.35, 200.36, 200.37, 200.38, 200.4, 200.40, 200.41, 200.42, 200.43, 200.44, 200.45, 200.46, 200.47, 200.48, 200.5, 200.50, 200.51, 200.52, 200.53, 200.54, 200.55, 200.56, 200.57, 200.58, 200.6, 200.60, 200.61, 200.62, 200.63, 200.64, 200.65, 200.66, 200.67, 200.68, 200.7, 200.70, 200.71, 200.72, 200.73, 200.74, 200.75, 200.76, 200.77, 200.78, 200.8, 200.80, 200.81, 200.82, 200.83, 200.84, 200.85, 200.86, 200.87, 200.88, 200.9, 202, 202.0, 202.00, 202.01, 202.02, 202.03, 202.04, 202.05, 202.06, 202.07, 202.08, 202.1, 202.10, 202.11, 202.12, 202.13, 202.14, 202.15, 202.16, 202.17, 202.18, 202.2, 202.20, 202.21, 202.22, 202.23, 202.24, 202.25, 202.26, 202.27, 202.28, 202.3, 202.30, 202.31, 202.32, 202.33, 202.34, 202.35, 202.36, 202.37, 202.38, 202.4, 202.40, 202.41, 202.42, 202.43, 202.44, 202.45, 202.46, 202.47, 202.48, 202.5, 202.50, 202.51, 202.52, 202.53, 202.54, 202.55, 202.56, 202.57, 202.58, 202.6, 202.60, 202.61, 202.62, 202.63, 202.64, 202.65, 202.66, 202.67, 202.68, 202.7, 202.70, 202.71, 202.72, 202.73, 202.74, 202.75, 202.76, 202.77, 202.78, 202.8, 202.80, 202.81, 202.82, 202.83, 202.84, 202.85, 202.86, 202.87, 202.88, 202.9, 202.90, 202.91, 202.92, 202.93, 202.94, 202.95, 202.96, 202.97, 202.98

Cause	ICCC3	ICD-10	ICD-9
		C84.4, C84.40, C84.41, C84.42, C84.43, C84.44, C84.45, C84.46, C84.47, C84.48, C84.49, C84.5, C84.6, C84.60, C84.61, C84.62, C84.63, C84.64, C84.65, C84.66, C84.67, C84.68, C84.69, C84.7, C84.70, C84.71, C84.72, C84.73, C84.74, C84.75, C84.76, C84.77, C84.78, C84.79, C84.8, C84.9, C84.90, C84.91, C84.92, C84.93, C84.94, C84.95, C84.96, C84.97, C84.98, C84.99, C85, C85.0, C85.1, C85.10, C85.11, C85.12, C85.13, C85.14, C85.15, C85.16, C85.17, C85.18, C85.19, C85.2, C85.20, C85.21, C85.22, C85.23, C85.24, C85.25, C85.26, C85.27, C85.28, C85.29, C85.3, C85.4, C85.5, C85.6, C85.7, C85.8, C85.80, C85.81, C85.82, C85.83, C85.84, C85.85, C85.86, C85.87, C85.88, C85.89, C85.9, C85.90, C85.91, C85.92, C85.93, C85.94, C85.95, C85.96, C85.97, C85.98, C85.99, C86, C86.0, C86.1, C86.2, C86.3, C86.4, C86.5, C86.6, C96, C96.0, C96.1, C96.2, C96.3, C96.4, C96.5, C96.6, C96.7, C96.8, C96.9	
Multiple myeloma	NA	C88, C88.0, C88.00, C88.01, C88.1, C88.2, C88.20, C88.3, C88.4, C88.40, C88.7, C88.70, C88.71, C88.8, C88.9, C89, C90, C90.0, C90.00, C90.01, C90.02, C90.1, C90.10, C90.11, C90.12, C90.2, C90.20, C90.21, C90.22, C90.3, C90.30, C90.31, C90.32, C90.4, C90.5, C90.6, C90.7, C90.8, C90.9	203, 203.0, 203.00, 203.01, 203.02, 203.1, 203.10, 203.11, 203.12, 203.8, 203.80, 203.81, 203.82, 203.9
Acute lymphoid leukaemia*	Ia, Ia1, Ia2, Ia3, Ia4	C91.0, C91.00, C91.01, C91.02, C91.2, C91.3, C91.30, C91.31, C91.32, C91.6, C91.60, C91.61, C91.62	204.0, 204.00, 204.01, 204.02, 204.2, 204.20, 204.21, 204.22
Chronic lymphoid leukaemia*[i]	Custom mapping	Custom mapping	Custom mapping
Acute myeloid leukaemia*	Ib	C92.0, C92.00, C92.01, C92.02, C92.3, C92.30, C92.31, C92.32, C92.4, C92.40, C92.41, C92.42, C92.5, C92.50, C92.51, C92.52, C92.6, C92.60, C92.61, C92.62, C93.0,	205.0, 205.00, 205.01, 205.02, 205.2, 205.20, 205.21, 205.22, 205.3, 205.30, 205.31, 205.32, 206.0, 206.00, 206.01,

Cause	ICCC3	ICD-10	ICD-9
		C93.00, C93.01, C93.02, C94.0, C94.00, C94.01, C94.02, C94.2, C94.20, C94.21, C94.22, C94.4, C94.40, C94.41, C94.42, C94.5	206.02, 207.0, 207.00, 207.01, 207.02, 207.20, 207.8, 207.80, 207.81, 207.82
Chronic myeloid leukaemia*	Ic	C92.1, C92.10, C92.11, C92.12, C92.2, C92.20, C92.21, C92.22	205.1, 205.10, 205.11, 205.12
Other leukaemia*	Ie	C91.20, C91.70, C92.70, C92.80, C93, C93.1, C93.10, C93.11, C93.12, C93.3, C93.30, C93.31, C93.32, C93.8, C94, C94.1, C94.3, C94.30, C94.31, C94.32, C94.50, C94.6, C94.60, C94.7, C94.70, C94.8, C94.80, C94.81, C94.82, C95, C95.0, C95.00, C95.01, C95.02, C95.1, C95.10, C95.11, C95.12, C95.2, C95.4, C95.6, C95.7, C95.70, C95.9, C95.90, C95.91, C95.92	205.92, 206.1, 206.10, 206.11, 206.12, 207, 207.1, 207.10, 207.11, 207.12, 207.2, 207.21, 207.22, 207.9, 208, 208.0, 208.00, 208.01, 208.02, 208.1, 208.10, 208.11, 208.12, 208.2, 208.20, 208.21, 208.22, 208.4, 208.7, 208.8, 208.80, 208.81, 208.82, 208.9, 208.90, 208.91, 208.92
Other malignant neoplasms*	VIII, VIIIa, VIIIb, VIIIc, VIIIc1, VIIIc2, VIIId, VIIId1, VIIId2, VIIId3, VIIId4, VIIIe, XIf9, V, IVa, IVb, XIIa1, XIIa4, XIIa6, XIIb, XIa, XIf10, XIf11, XIf5, Xb, Xb1, Xb2, Xb3, Xb4, Xb5, Xb6, IX, IXa, IXb, IXb1, IXb2, IXb3, IXd, IXd1, IXd10, IXd11, IXd2, IXd3, IXd4, IXd5, IXd6, IXd7, IXd8, IXd9, IXe	C40, C40.0, C40.00, C40.01, C40.02, C40.1, C40.10, C40.11, C40.12, C40.2, C40.20, C40.21, C40.22, C40.3, C40.30, C40.31, C40.32, C40.8, C40.80, C40.81, C40.82, C40.9, C40.90, C40.91, C40.92, C41, C41.0, C41.01, C41.02, C41.1, C41.2, C41.3, C41.4, C41.5, C41.6, C41.7, C41.8, C41.9, C42.0, C42.1, C42.2, C42.3, C42.4, C69.0, C69.00, C69.01, C69.02, C69.1, C69.10, C69.11, C69.12, C69.3, C69.30, C69.31, C69.32, C69.4, C69.40, C69.41, C69.42, C69.5, C69.50, C69.51, C69.52, C69.6, C69.60, C69.61, C69.62, C69.7, C69.8, C69.80, C69.81, C69.82, C69.2, C69.20, C69.21, C69.22, C47, C47.0, C47.1, C47.10, C47.11, C47.12, C47.2, C47.20, C47.21, C47.22, C47.3, C47.4, C47.5, C47.6, C47.8, C47.9, C74.90, C17, C17.0, C17.1, C17.2, C17.3, C17.8, C17.9, C3, C30, C30.0, C30.1, C30.2, C30.3, C30.5, C30.8, C30.9, C31, C31.0, C31.1, C31.2, C31.3, C31.8, C31.9, C37, C37.0, C37.1, C37.2, C37.3, C37.9, C38, C38.0, C38.1, C38.2, C38.3, C38.4, C38.8, C4, C48, C48.0, C48.1, C48.2, C48.8, C48.9, C4A, C5, C51, C51.0, C51.1, C51.2, C51.8, C51.9, C52, C52.0, C52.9, C57, C57.0, C57.00, C57.01,	170, 170.0, 170.1, 170.2, 170.3, 170.4, 170.5, 170.6, 170.7, 170.8, 170.9, 190, 190.0, 190.1, 190.2, 190.3, 190.4, 190.6, 190.7, 190.8, 190.5, 152, 152.0, 152.1, 152.2, 152.3, 152.4, 152.6, 152.8, 152.9, 158, 158.0, 158.3, 158.4, 158.5, 158.6, 158.8, 158.9, 160, 160.0, 160.1, 160.2, 160.3, 160.4, 160.5, 160.6, 160.8, 160.9, 163, 163.0, 163.1, 163.3, 163.5, 163.8, 163.9, 164, 164.0, 164.1, 164.2, 164.3, 164.8, 164.9, 181, 181.0, 181.9, 183.2, 183.3, 183.4, 183.5, 183.8, 184.0, 184.1, 184.2, 184.3, 184.4, 184.8, 187.1, 187.2, 187.3, 187.4, 187.5, 187.6, 187.7, 187.8, 189.2, 189.3, 189.4, 189.8, 194.1, 194.3, 194.4, 194.5, 194.6, 194.8, 209.0, 209.00, 209.01, 209.02, 209.03, 209.22, 209.25, 209.26, 209.27, 209.31, 209.32, 209.33, 209.34, 209.35, 209.36, 171, 171.0, 171.2, 171.3, 171.4, 171.5, 171.6, 171.7, 171.8, 171.9

Cause	ICCC3	ICD-10	ICD-9
		C57.02, C57.1, C57.10, C57.11, C57.12, C57.2, C57.20, C57.21, C57.22, C57.3, C57.4, C57.7, C57.8, C58, C58.0, C58.9, C60, C60.0, C60.1, C60.2, C60.8, C60.9, C63, C63.0, C63.00, C63.01, C63.02, C63.1, C63.10, C63.11, C63.12, C63.2, C63.7, C63.8, C66, C66.0, C66.1, C66.2, C66.9, C68.0, C68.1, C68.8, C7, C75, C75.0, C75.1, C75.2, C75.3, C75.4, C75.5, C75.6, C75.8, C49, C49.0, C49.1, C49.10, C49.11, C49.12, C49.2, C49.20, C49.21, C49.22, C49.3, C49.4, C49.5, C49.6, C49.8, C49.9	

Abbreviations: ICCC3, International Classification of Childhood Cancer, Third Edition; ICD-9, International Classification of Diseases, Ninth Revision; ICD-10, International Classification of Diseases, Tenth Revision; NA, not applicable.

*The GBD study does not currently estimate burden attributable to any risk factors for these cancer types, so these cancers are not shown in results for this analysis. These cancers are included in this table for completeness and accuracy in reporting GBD cancer estimation methods.

[i]Chronic lymphoid leukaemia is only modeled for ages 20 years and above in GBD 2019. ICD codes (ICD-9: 204.1, 204.10, 204.11, and 204.12; ICD-10: C91.1, C91.10, C91.11, and C91.12) under 20 years are redistributed (see Section “6. Redistribution” on pg. 29 for more information) to “Acute lymphoid leukaemia”, while these ICD codes over 20 years old are mapped directly to “Chronic lymphoid leukaemia”.

Appendix Table 2: List of International Classification of Diseases (ICD) codes mapped to the Global Burden of Disease cause list for cancer mortality data

Cause	ICCC3	ICD-10	ICD-9
Lip and oral cavity cancer	XIf1	C00, C00.0, C00.1, C00.2, C00.3, C00.4, C00.5, C00.6, C00.8, C00.9, C01, C01.9, C02, C02.0, C02.1, C02.2, C02.3, C02.4, C02.8, C02.9, C03, C03.0, C03.1, C03.9, C04, C04.0, C04.1, C04.8, C04.9, C05, C05.0, C05.1, C05.2, C05.8, C05.9, C06, C06.0, C06.1, C06.2, C06.8, C06.80, C06.89, C06.9, C07, C07.0, C07.9, C08, C08.0, C08.1, C08.8, C08.9, D00.00, D00.01, D00.02, D00.03, D00.04, D00.05, D00.06, D00.07, D10.0, D10.1, D10.2, D10.3, D10.30, D10.39, D10.4, D10.5, D11, D11.0, D11.7, D11.9, D37.01, D37.02, D37.03, D37.030, D37.031, D37.032, D37.039, D37.04, D37.09	140, 140.0, 140.1, 140.2, 140.3, 140.4, 140.5, 140.6, 140.7, 140.8, 140.9, 141, 141.0, 141.1, 141.2, 141.3, 141.4, 141.5, 141.6, 141.8, 141.9, 142, 142.0, 142.1, 142.2, 142.3, 142.8, 142.9, 143, 143.0, 143.1, 143.8, 143.9, 144, 144.0, 144.1, 144.4, 144.8, 144.9, 145, 145.0, 145.1, 145.2, 145.3, 145.4, 145.5, 145.6, 145.8, 145.9, 210, 210.0, 210.1, 210.2, 210.3, 210.4, 210.5, 210.6, 235, 235.0

Cause	ICCC3	ICD-10	ICD-9
Nasopharynx cancer	XIc	C11, C11.0, C11.1, C11.2, C11.3, C11.8, C11.9, D00.08, D10.6, D37.05	147, 147.0, 147.1, 147.2, 147.3, 147.8, 147.9, 210.7, 210.8, 210.9
Other pharynx cancer	NA	C09, C09.0, C09.1, C09.8, C09.9, C1, C10, C10.0, C10.1, C10.2, C10.3, C10.4, C10.8, C10.9, C12, C12.0, C12.9, C13, C13.0, C13.1, C13.2, C13.8, C13.9, D10.7	146, 146.0, 146.1, 146.2, 146.3, 146.4, 146.5, 146.6, 146.7, 146.8, 146.9, 148, 148.0, 148.1, 148.2, 148.3, 148.4, 148.5, 148.8, 148.9
Oesophageal cancer	NA	C15, C15.0, C15.1, C15.2, C15.3, C15.4, C15.5, C15.8, C15.9, D00.1, D13.0	150, 150.0, 150.1, 150.2, 150.3, 150.4, 150.5, 150.6, 150.7, 150.8, 150.9, 211, 211.0, 230.1
Stomach cancer	NA	C16, C16.0, C16.1, C16.2, C16.3, C16.4, C16.5, C16.6, C16.7, C16.8, C16.9, D00.2, D13.1, D37.1	151, 151.0, 151.1, 151.2, 151.3, 151.4, 151.5, 151.6, 151.8, 151.9, 209.23, 209.63, 211.1, 230.2
Colon and rectum cancer	XIf2, XIf3	C18, C18.0, C18.1, C18.2, C18.3, C18.4, C18.5, C18.6, C18.7, C18.8, C18.9, C19, C19.0, C19.9, C2, C20, C20.0, C20.8, C20.9, C21, C21.0, C21.1, C21.2, C21.8, C21.9, D01.0, D01.1, D01.2, D01.3, D12, D12.0, D12.1, D12.2, D12.3, D12.4, D12.5, D12.6, D12.7, D12.8, D12.9, D37.3, D37.4, D37.5	153, 153.0, 153.1, 153.2, 153.3, 153.4, 153.5, 153.6, 153.7, 153.8, 153.9, 154, 154.0, 154.1, 154.2, 154.3, 154.4, 154.8, 154.9, 209.1, 209.10, 209.11, 209.12, 209.13, 209.14, 209.15, 209.16, 209.17, 209.5, 209.50, 209.51, 209.52, 209.53, 209.54, 209.55, 209.56, 209.57, 211.3, 211.4, 230.3, 230.4, 230.5, 230.6, 569.0, 569.43, 569.44, 569.84, 569.85
Liver cancer	VIIb, VIIc	C22, C22.0, C22.1, C22.3, C22.4, C22.5, C22.7, C22.8, D13.4	155, 155.0, 155.1, 155.3, 155.5, 155.9, 211.5
Gallbladder and biliary tract cancer	NA	C23, C23.0, C23.9, C24, C24.0, C24.1, C24.4, C24.8, C24.9, D13.5	156, 156.0, 156.1, 156.2, 156.3, 156.8, 156.9, 209.65, 209.66, 209.67
Pancreatic cancer	XIIa2	C25, C25.0, C25.1, C25.2, C25.3, C25.4, C25.7, C25.8, C25.9, D13.6, D13.7	157, 157.0, 157.1, 157.2, 157.3, 157.4, 157.5, 157.7, 157.8, 157.9, 211.6, 211.7
Larynx cancer	NA	C32, C32.0, C32.1, C32.2, C32.3, C32.8, C32.9, D02.0, D14.1, D38.0	161, 161.0, 161.1, 161.2, 161.3, 161.8, 161.9, 212.1, 231, 231.0, 235.6
Tracheal, bronchus, and lung cancer	XIIa3, XIf4	C33, C33.0, C33.2, C33.9, C34, C34.0, C34.00, C34.01, C34.02, C34.1, C34.10, C34.11, C34.12, C34.2, C34.3, C34.30, C34.31, C34.32, C34.4, C34.7, C34.8, C34.80, C34.81, C34.82, C34.9, C34.90, C34.91, C34.92, D02.1, D02.2, D02.20, D02.21, D02.22, D02.3, D14.2, D14.3, D14.30, D14.31, D14.32, D38.1	162, 162.0, 162.1, 162.2, 162.3, 162.4, 162.5, 162.8, 162.9, 209.21, 209.61, 212.2, 212.3, 231.1, 231.2, 235.7
Malignant skin melanoma*	XId	C43, C43.0, C43.1, C43.10, C43.11, C43.12, C43.2, C43.20, C43.21, C43.22, C43.3, C43.30, C43.31, C43.39, C43.4, C43.5, C43.51, C43.52, C43.59, C43.6, C43.60, C43.61, C43.62, C43.7, C43.70,	172, 172.0, 172.1, 172.2, 172.3, 172.4, 172.5, 172.6, 172.7, 172.8, 172.9

Cause	ICCC3	ICD-10	ICD-9
		C43.71, C43.72, C43.8, C43.9, D03, D03.0, D03.1, D03.10, D03.11, D03.12, D03.2, D03.20, D03.21, D03.22, D03.3, D03.30, D03.39, D03.4, D03.5, D03.51, D03.52, D03.59, D03.6, D03.60, D03.61, D03.62, D03.7, D03.70, D03.71, D03.72, D03.8, D03.9, D22, D22.0, D22.1, D22.10, D22.11, D22.12, D22.2, D22.20, D22.21, D22.22, D22.3, D22.30, D22.39, D22.4, D22.5, D22.6, D22.60, D22.61, D22.62, D22.7, D22.70, D22.71, D22.72, D22.9, D23, D23.0, D23.1, D23.10, D23.11, D23.12, D23.2, D23.20, D23.21, D23.22, D23.3, D23.30, D23.39, D23.4, D23.5, D23.6, D23.60, D23.61, D23.62, D23.7, D23.70, D23.71, D23.72, D23.9	
Breast cancer	XIf6	C50, C50.0, C50.01, C50.011, C50.012, C50.019, C50.02, C50.021, C50.022, C50.029, C50.1, C50.11, C50.111, C50.112, C50.119, C50.12, C50.121, C50.122, C50.129, C50.2, C50.21, C50.211, C50.212, C50.219, C50.22, C50.221, C50.222, C50.229, C50.3, C50.31, C50.311, C50.312, C50.319, C50.32, C50.321, C50.322, C50.329, C50.4, C50.41, C50.411, C50.412, C50.419, C50.42, C50.421, C50.422, C50.429, C50.5, C50.51, C50.511, C50.512, C50.519, C50.52, C50.521, C50.522, C50.529, C50.6, C50.61, C50.611, C50.612, C50.619, C50.62, C50.621, C50.622, C50.629, C50.7, C50.8, C50.81, C50.811, C50.812, C50.819, C50.82, C50.821, C50.822, C50.829, C50.9, C50.91, C50.911, C50.912, C50.919, C50.92, C50.921, C50.922, C50.929, D05, D05.0, D05.00, D05.01, D05.02, D05.1, D05.10, D05.11, D05.12, D05.7, D05.8, D05.80, D05.81, D05.82, D05.9, D05.90, D05.91, D05.92, D24, D24.0, D24.1, D24.2, D24.9, D48.6, D48.60, D48.61, D48.62, D49.3	174, 174.0, 174.1, 174.2, 174.3, 174.4, 174.5, 174.6, 174.8, 174.9, 175, 175.0, 175.3, 175.9, 217, 217.0, 217.8, 233, 233.0, 238.3, 239.3, 610, 610.0, 610.1, 610.2, 610.3, 610.4, 610.8, 610.9
Cervical cancer	XIf7	C53, C53.0, C53.1, C53.3, C53.4, C53.8, C53.9, D06, D06.0, D06.1, D06.7, D06.9, D26.0	180, 180.0, 180.1, 180.2, 180.3, 180.4, 180.5, 180.6, 180.8, 180.9, 219, 219.0, 233.1, 622.1, 622.10, 622.11, 622.12, 622.2, 622.7

Cause	ICCC3	ICD-10	ICD-9
Uterine cancer	NA	C54, C54.0, C54.1, C54.2, C54.3, C54.4, C54.8, C54.9, D07.0, D07.1, D07.2, D26.1, D26.7, D26.9	182, 182.0, 182.1, 182.8, 182.9, 233.2
Ovarian cancer	NA	C56, C56.0, C56.1, C56.2, C56.4, C56.9, D27, D27.0, D27.1, D27.9, D39.1, D39.10, D39.11, D39.12	183, 183.0, 220, 220.0, 220.9, 236.2
Prostate cancer	NA	C61, C61.0, C61.9, D07.5, D29.1, D40.0	185, 185.0, 185.9, 222.2, 236.5
Testicular cancer*	NA	C62, C62.0, C62.00, C62.01, C62.02, C62.1, C62.10, C62.11, C62.12, C62.9, C62.90, C62.91, C62.92, D29.2, D29.20, D29.21, D29.22, D29.3, D29.30, D29.31, D29.32, D29.4, D29.7, D29.8, D40.1, D40.10, D40.11, D40.12, D40.7, D40.8	186, 186.0, 186.9, 222, 222.0, 222.3, 236.4
Kidney cancer	VI, VIa, VIa1, VIa2, VIa3, VIa4, VIb, VIc	C64, C64.0, C64.1, C64.2, C64.4, C64.5, C64.6, C64.8, C64.9, C65, C65.0, C65.1, C65.2, C65.9, D30.0, D30.00, D30.01, D30.02, D30.1, D30.10, D30.11, D30.12, D41.0, D41.00, D41.01, D41.02, D41.1, D41.10, D41.11, D41.12	189.0, 189.1, 189.5, 189.6, 209.24, 209.64, 223, 223.0, 223.1, 236.91
Bladder cancer	XIf8	C67, C67.0, C67.1, C67.2, C67.3, C67.4, C67.5, C67.6, C67.7, C67.8, C67.9, D09.0, D30.3, D41.4, D41.7, D41.8, D49.4	188, 188.0, 188.1, 188.2, 188.3, 188.4, 188.5, 188.6, 188.7, 188.8, 188.9, 223.3, 233.7, 236.7, 239.4
Brain and central nervous system cancer*	III, IIIa, IIIa1, IIIa2, IIIb, IIIc, IIIc1, IIIc2, IIIc3, IIIc4, IIId, IIId1, IIId2, IIId3, IIIe, IIIe1, IIIe2, IIIe3, IIIe4, IIIe5, IIIf, Xa, Xa1, Xa2, Xa3, Xa4, Xa5, Xa6	C70, C70.0, C70.1, C70.5, C70.6, C70.9, C71, C71.0, C71.1, C71.2, C71.3, C71.4, C71.5, C71.6, C71.7, C71.8, C71.9, C72, C72.0, C72.1, C72.2, C72.20, C72.21, C72.22, C72.3, C72.30, C72.31, C72.32, C72.4, C72.40, C72.41, C72.42, C72.5, C72.50, C72.59, C72.8, C72.9	191, 191.0, 191.1, 191.2, 191.3, 191.4, 191.5, 191.6, 191.7, 191.8, 191.9, 192, 192.0, 192.1, 192.2, 192.3, 192.4, 192.8, 192.9
Thyroid cancer	XIb	C73, C73.0, C73.1, C73.2, C73.3, C73.4, C73.5, C73.8, C73.9, D09.3, D09.8, D34, D34.0, D34.9, D44.0	193, 193.0, 193.2, 193.9, 226, 226.0, 226.9
Mesothelioma	XIIa5	C45, C45.0, C45.1, C45.2, C45.3, C45.4, C45.5, C45.6, C45.7, C45.8, C45.9	NA
Hodgkin lymphoma*	IIa	C81, C81.0, C81.00, C81.01, C81.02, C81.03, C81.04, C81.05, C81.06, C81.07, C81.08, C81.09, C81.1, C81.10, C81.11, C81.12, C81.13, C81.14, C81.15, C81.16,	201, 201.0, 201.00, 201.01, 201.02, 201.03, 201.04, 201.05, 201.06, 201.07, 201.08, 201.1, 201.10, 201.11, 201.12, 201.13, 201.14, 201.15, 201.16, 201.17,

Cause	ICCC3	ICD-10	ICD-9
		C81.17, C81.18, C81.19, C81.2, C81.20, C81.21, C81.22, C81.23, C81.24, C81.25, C81.26, C81.27, C81.28, C81.29, C81.3, C81.30, C81.31, C81.32, C81.33, C81.34, C81.35, C81.36, C81.37, C81.38, C81.39, C81.4, C81.40, C81.41, C81.42, C81.43, C81.44, C81.45, C81.46, C81.47, C81.48, C81.49, C81.5, C81.6, C81.7, C81.70, C81.71, C81.72, C81.73, C81.74, C81.75, C81.76, C81.77, C81.78, C81.79, C81.8, C81.9, C81.90, C81.91, C81.92, C81.93, C81.94, C81.95, C81.96, C81.97, C81.98, C81.99	201.18, 201.2, 201.20, 201.21, 201.22, 201.23, 201.24, 201.25, 201.26, 201.27, 201.28, 201.4, 201.40, 201.41, 201.42, 201.43, 201.44, 201.45, 201.46, 201.47, 201.48, 201.5, 201.50, 201.51, 201.52, 201.53, 201.54, 201.55, 201.56, 201.57, 201.58, 201.6, 201.60, 201.61, 201.62, 201.63, 201.64, 201.65, 201.66, 201.67, 201.68, 201.7, 201.70, 201.71, 201.72, 201.73, 201.74, 201.75, 201.76, 201.77, 201.78, 201.9, 201.90, 201.91, 201.92, 201.93, 201.94, 201.95, 201.96, 201.97, 201.98
Non-Hodgkin lymphoma	IIb, IIb1, IIb2, IIb3, IIb4, IIc, IId, IIe	C83.7, C83.70, C83.71, C83.72, C83.73, C83.74, C83.75, C83.76, C83.77, C83.78, C83.79, C83.8, C82, C82.0, C82.00, C82.01, C82.02, C82.03, C82.04, C82.05, C82.06, C82.07, C82.08, C82.09, C82.1, C82.10, C82.11, C82.12, C82.13, C82.14, C82.15, C82.16, C82.17, C82.18, C82.19, C82.2, C82.20, C82.21, C82.22, C82.23, C82.24, C82.25, C82.26, C82.27, C82.28, C82.29, C82.3, C82.30, C82.31, C82.32, C82.33, C82.34, C82.35, C82.36, C82.37, C82.38, C82.39, C82.4, C82.40, C82.41, C82.42, C82.43, C82.44, C82.45, C82.46, C82.47, C82.48, C82.49, C82.5, C82.50, C82.51, C82.52, C82.53, C82.54, C82.55, C82.56, C82.57, C82.58, C82.59, C82.6, C82.60, C82.61, C82.62, C82.63, C82.64, C82.65, C82.66, C82.67, C82.68, C82.69, C82.7, C82.8, C82.80, C82.81, C82.82, C82.83, C82.84, C82.85, C82.86, C82.87, C82.88, C82.89, C82.9, C82.90, C82.91, C82.92, C82.93, C82.94, C82.95, C82.96, C82.97, C82.98, C82.99, C83, C83.0, C83.00, C83.01, C83.02, C83.03, C83.04, C83.05, C83.06, C83.07, C83.08, C83.09, C83.1, C83.10, C83.11, C83.12, C83.13, C83.14, C83.15, C83.16, C83.17, C83.18, C83.19, C83.2, C83.3, C83.30, C83.31, C83.32, C83.33, C83.34, C83.35, C83.36, C83.37, C83.38, C83.39, C83.4, C83.5, C83.50, C83.51, C83.52, C83.53, C83.54, C83.55, C83.56, C83.57, C83.58, C83.59, C83.6, C83.80, C83.81, C83.82, C83.83, C83.84, C83.85, C83.86, C83.87, C83.88, C83.89, C83.9, C83.90, C83.91, C83.92, C83.93, C83.94, C83.95, C83.96, C83.97,	200.2, 200.20, 200.21, 200.22, 200.23, 200.24, 200.25, 200.26, 200.27, 200.28, 200, 200.0, 200.00, 200.01, 200.02, 200.03, 200.04, 200.05, 200.06, 200.07, 200.08, 200.1, 200.10, 200.11, 200.12, 200.13, 200.14, 200.15, 200.16, 200.17, 200.18, 200.3, 200.30, 200.31, 200.32, 200.33, 200.34, 200.35, 200.36, 200.37, 200.38, 200.4, 200.40, 200.41, 200.42, 200.43, 200.44, 200.45, 200.46, 200.47, 200.48, 200.5, 200.50, 200.51, 200.52, 200.53, 200.54, 200.55, 200.56, 200.57, 200.58, 200.6, 200.60, 200.61, 200.62, 200.63, 200.64, 200.65, 200.66, 200.67, 200.68, 200.7, 200.70, 200.71, 200.72, 200.73, 200.74, 200.75, 200.76, 200.77, 200.78, 200.8, 200.80, 200.81, 200.82, 200.83, 200.84, 200.85, 200.86, 200.87, 200.88, 200.9, 202, 202.0, 202.00, 202.01, 202.02, 202.03, 202.04, 202.05, 202.06, 202.07, 202.08, 202.1, 202.10, 202.11, 202.12, 202.13, 202.14, 202.15, 202.16, 202.17, 202.18, 202.2, 202.20, 202.21, 202.22, 202.23, 202.24, 202.25, 202.26, 202.27, 202.28, 202.3, 202.30, 202.31, 202.32, 202.33, 202.34, 202.35, 202.36, 202.37, 202.38, 202.4, 202.40, 202.41, 202.42, 202.43, 202.44, 202.45, 202.46, 202.47, 202.48, 202.5, 202.50, 202.51, 202.52, 202.53, 202.54, 202.55, 202.56, 202.57, 202.58, 202.6, 202.60, 202.61, 202.62, 202.63, 202.64, 202.65, 202.66, 202.67, 202.68, 202.7, 202.70, 202.71, 202.72, 202.73, 202.74, 202.75, 202.76, 202.77, 202.78, 202.8, 202.80, 202.81, 202.82, 202.83, 202.84, 202.85, 202.86,

Cause	ICCC3	ICD-10	ICD-9
		C83.98, C83.99, C84, C84.0, C84.00, C84.01, C84.02, C84.03, C84.04, C84.05, C84.06, C84.07, C84.08, C84.09, C84.1, C84.10, C84.11, C84.12, C84.13, C84.14, C84.15, C84.16, C84.17, C84.18, C84.19, C84.2, C84.3, C84.4, C84.40, C84.41, C84.42, C84.43, C84.44, C84.45, C84.46, C84.47, C84.48, C84.49, C84.5, C84.6, C84.60, C84.61, C84.62, C84.63, C84.64, C84.65, C84.66, C84.67, C84.68, C84.69, C84.7, C84.70, C84.71, C84.72, C84.73, C84.74, C84.75, C84.76, C84.77, C84.78, C84.79, C84.8, C84.9, C84.90, C84.91, C84.92, C84.93, C84.94, C84.95, C84.96, C84.97, C84.98, C84.99, C85, C85.0, C85.1, C85.10, C85.11, C85.12, C85.13, C85.14, C85.15, C85.16, C85.17, C85.18, C85.19, C85.2, C85.20, C85.21, C85.22, C85.23, C85.24, C85.25, C85.26, C85.27, C85.28, C85.29, C85.3, C85.4, C85.5, C85.6, C85.7, C85.8, C85.80, C85.81, C85.82, C85.83, C85.84, C85.85, C85.86, C85.87, C85.88, C85.89, C85.9, C85.90, C85.91, C85.92, C85.93, C85.94, C85.95, C85.96, C85.97, C85.98, C85.99, C86, C86.0, C86.1, C86.2, C86.3, C86.4, C86.5, C86.6, C96, C96.0, C96.1, C96.2, C96.3, C96.4, C96.5, C96.6, C96.7, C96.8, C96.9	202.87, 202.88, 202.9, 202.90, 202.91, 202.92, 202.93, 202.94, 202.95, 202.96, 202.97, 202.98
Multiple myeloma	NA	C88, C88.0, C88.00, C88.01, C88.1, C88.2, C88.20, C88.3, C88.4, C88.40, C88.7, C88.70, C88.71, C88.8, C88.9, C89, C90, C90.0, C90.00, C90.01, C90.02, C90.1, C90.10, C90.11, C90.12, C90.2, C90.20, C90.21, C90.22, C90.3, C90.30, C90.31, C90.32, C90.4, C90.5, C90.6, C90.7, C90.8, C90.9	203, 203.0, 203.00, 203.01, 203.02, 203.1, 203.10, 203.11, 203.12, 203.8, 203.80, 203.81, 203.82, 203.9
Acute lymphoid leukaemia*	Ia, Ia1, Ia2, Ia3, Ia4	C91.0, C91.00, C91.01, C91.02, C91.2, C91.3, C91.30, C91.31, C91.32, C91.6, C91.60, C91.61, C91.62	204.0, 204.00, 204.01, 204.02, 204.2, 204.20, 204.21, 204.22
Chronic lymphoid leukaemia*[i]	Custom mapping	Custom mapping	Custom mapping
Acute myeloid leukaemia*	Ib	C92.0, C92.00, C92.01, C92.02, C92.3, C92.30, C92.31, C92.32, C92.4, C92.40, C92.41, C92.42, C92.5, C92.50, C92.51, C92.52, C92.6, C92.60, C92.61, C92.62, C93.0, C93.00, C93.01, C93.02, C94.0,	205.0, 205.00, 205.01, 205.02, 205.2, 205.20, 205.21, 205.22, 205.3, 205.30, 205.31, 205.32, 206.0, 206.00, 206.01,

Cause	ICCC3	ICD-10	ICD-9
		C94.00, C94.01, C94.02, C94.2, C94.20, C94.21, C94.22, C94.4, C94.40, C94.41, C94.42, C94.5	206.02, 207.0, 207.00, 207.01, 207.02, 207.20, 207.8, 207.80, 207.81, 207.82
Chronic myeloid leukaemia*	Ic	C92.1, C92.10, C92.11, C92.12, C92.2, C92.20, C92.21, C92.22	205.1, 205.10, 205.11, 205.12
Other leukaemia*	Ie	C91.20, C91.70, C92.70, C92.80, C93, C93.1, C93.10, C93.11, C93.12, C93.3, C93.30, C93.31, C93.32, C93.8, C94, C94.1, C94.3, C94.30, C94.31, C94.32, C94.50, C94.6, C94.60, C94.7, C94.70, C94.8, C94.80, C94.81, C94.82, C95, C95.0, C95.00, C95.01, C95.02, C95.1, C95.10, C95.11, C95.12, C95.2, C95.4, C95.6, C95.7, C95.70, C95.9, C95.90, C95.91, C95.92	205.92, 206.1, 206.10, 206.11, 206.12, 207, 207.1, 207.10, 207.11, 207.12, 207.2, 207.21, 207.22, 207.9, 208, 208.0, 208.00, 208.01, 208.02, 208.1, 208.10, 208.11, 208.12, 208.2, 208.20, 208.21, 208.22, 208.4, 208.7, 208.8, 208.80, 208.81, 208.82, 208.9, 208.90, 208.91, 208.92
Other malignant neoplasms*	VIII, VIIIa, VIIIb, VIIIc, VIIIc1, VIIIc2, VIIId, VIIId1, VIIId2, VIIId3, VIIId4, VIIIe, XIf9, V, IVa, IVb, XIIa1, XIIa4, XIIa6, XIIb, XIa, XIf10, XIf11, XIf5, Xb, Xb1, Xb2, Xb3, Xb4, Xb5, Xb6, IX, IXa, IXb, IXb1, IXb2, IXb3, IXd, IXd1, IXd10, IXd11, IXd2, IXd3, IXd4, IXd5, IXd6, IXd7, IXd8, IXd9, IXe	C40, C40.0, C40.00, C40.01, C40.02, C40.1, C40.10, C40.11, C40.12, C40.2, C40.20, C40.21, C40.22, C40.3, C40.30, C40.31, C40.32, C40.8, C40.80, C40.81, C40.82, C40.9, C40.90, C40.91, C40.92, C41, C41.0, C41.01, C41.02, C41.1, C41.2, C41.3, C41.4, C41.5, C41.6, C41.7, C41.8, C41.9, C42.0, C42.1, C42.2, C42.3, C42.4, C69.0, C69.00, C69.01, C69.02, C69.1, C69.10, C69.11, C69.12, C69.3, C69.30, C69.31, C69.32, C69.4, C69.40, C69.41, C69.42, C69.5, C69.50, C69.51, C69.52, C69.6, C69.60, C69.61, C69.62, C69.7, C69.8, C69.80, C69.81, C69.82, C69.2, C69.20, C69.21, C69.22, C47, C47.0, C47.1, C47.10, C47.11, C47.12, C47.2, C47.20, C47.21, C47.22, C47.3, C47.4, C47.5, C47.6, C47.8, C47.9, C74.90, C17, C17.0, C17.1, C17.2, C17.3, C17.8, C17.9, C3, C30, C30.0, C30.1, C30.2, C30.3, C30.5, C30.8, C30.9, C31, C31.0, C31.1, C31.2, C31.3, C31.8, C31.9, C37, C37.0, C37.1, C37.2, C37.3, C37.9, C38, C38.0, C38.1, C38.2, C38.3, C38.4, C38.8, C4, C48, C48.0, C48.1, C48.2, C48.8, C48.9, C4A, C5, C51, C51.0, C51.1, C51.2, C51.8, C51.9, C52, C52.0, C52.9, C57, C57.0, C57.00, C57.01, C57.02, C57.1, C57.10, C57.11, C57.12, C57.2, C57.20, C57.21, C57.22, C57.3, C57.4, C57.7, C57.8, C58, C58.0, C58.9, C60, C60.0, C60.1, C60.2, C60.8, C60.9,	170, 170.0, 170.1, 170.2, 170.3, 170.4, 170.5, 170.6, 170.7, 170.8, 170.9, 190, 190.0, 190.1, 190.2, 190.3, 190.4, 190.6, 190.7, 190.8, 190.5, 152, 152.0, 152.1, 152.2, 152.3, 152.4, 152.6, 152.8, 152.9, 158, 158.0, 158.3, 158.4, 158.5, 158.6, 158.8, 158.9, 160, 160.0, 160.1, 160.2, 160.3, 160.4, 160.5, 160.6, 160.8, 160.9, 163, 163.0, 163.1, 163.3, 163.5, 163.8, 163.9, 164, 164.0, 164.1, 164.2, 164.3, 164.8, 164.9, 181, 181.0, 181.9, 183.2, 183.3, 183.4, 183.5, 183.8, 184.0, 184.1, 184.2, 184.3, 184.4, 184.8, 187.1, 187.2, 187.3, 187.4, 187.5, 187.6, 187.7, 187.8, 189.2, 189.3, 189.4, 189.8, 194.1, 194.3, 194.4, 194.5, 194.6, 194.8, 209.0, 209.00, 209.01, 209.02, 209.03, 209.22, 209.25, 209.26, 209.27, 209.31, 209.32, 209.33, 209.34, 209.35, 209.36, 209.4, 209.40, 209.41, 209.42, 209.43, 211.2, 211.8, 212.0, 212.4, 212.5, 212.6, 212.7, 212.8, 213, 213.0, 213.1, 213.2, 213.3, 213.4, 213.5, 213.6, 213.7, 213.8, 213.9, 221.0, 221.1, 221.2, 221.8, 222.1, 222.8, 223.2, 223.8, 223.81, 223.89, 224, 224.0, 224.1, 224.2, 224.3, 224.4, 224.5, 224.6, 224.7, 224.8, 224.9, 225, 225.0, 225.1, 225.2, 225.3, 225.4, 225.8, 225.9, 227, 227.0, 227.1, 227.3, 227.4, 227.5, 227.6, 227.8, 227.9, 228, 228.0, 228.00, 228.01, 228.02, 228.03, 228.04, 228.09, 228.1, 228.9, 229.0, 229.8, 230.7, 230.8, 233.31, 233.32,

Cause	ICCC3	ICD-10	ICD-9
		C63, C63.0, C63.00, C63.01, C63.02, C63.1, C63.10, C63.11, C63.12, C63.2, C63.7, C63.8, C66, C66.0, C66.1, C66.2, C66.9, C68.0, C68.1, C68.8, C7, C75, C75.0, C75.1, C75.2, C75.3, C75.4, C75.5, C75.6, C75.8, D07.4, D09.2, D09.20, D09.21, D09.22, D13.2, D13.3, D13.30, D13.39, D14.0, D15, D15.0, D15.1, D15.2, D15.7, D15.9, D16, D16.0, D16.00, D16.01, D16.02, D16.1, D16.10, D16.11, D16.12, D16.2, D16.20, D16.21, D16.22, D16.3, D16.30, D16.31, D16.32, D16.4, D16.5, D16.6, D16.7, D16.8, D16.9, D28.0, D28.1, D28.7, D29.0, D30.2, D30.20, D30.21, D30.22, D30.4, D30.7, D30.8, D31, D31.0, D31.00, D31.01, D31.02, D31.1, D31.10, D31.11, D31.12, D31.2, D31.20, D31.21, D31.22, D31.3, D31.30, D31.31, D31.32, D31.4, D31.40, D31.41, D31.42, D31.5, D31.50, D31.51, D31.52, D31.6, D31.60, D31.61, D31.62, D31.9, D31.90, D31.91, D31.92, D32, D32.0, D32.1, D32.9, D33, D33.0, D33.1, D33.2, D33.3, D33.4, D33.7, D33.9, D35, D35.0, D35.00, D35.01, D35.02, D35.1, D35.2, D35.3, D35.4, D35.5, D35.6, D35.7, D35.8, D35.9, D36, D36.1, D36.10, D36.11, D36.12, D36.13, D36.14, D36.15, D36.16, D36.17, D36.7, D37.2, D38.2, D38.3, D38.4, D38.5, D39.2, D39.8, D41.2, D41.20, D41.21, D41.22, D41.3, D42, D42.0, D42.1, D42.9, D43, D43.0, D43.1, D43.2, D43.3, D43.4, D43.7, D43.8, D43.9, D44.1, D44.10, D44.11, D44.12, D44.2, D44.3, D44.4, D44.5, D44.6, D44.7, D44.8, D48.0, D48.1, D48.2, D48.3, D48.4, D49.6, D49.81, C49, C49.0, C49.1, C49.10, C49.11, C49.12, C49.2, C49.20, C49.21, C49.22, C49.3, C49.4, C49.5, C49.6, C49.8, C49.9	233.4, 233.5, 234.0, 234.5, 234.8, 235.4, 235.8, 236.1, 236.99, 237, 237.0, 237.1, 237.2, 237.3, 237.5, 237.6, 237.7, 237.70, 237.71, 237.72, 237.73, 237.79, 237.9, 238.0, 238.1, 239.2, 239.6, 171, 171.0, 171.2, 171.3, 171.4, 171.5, 171.6, 171.7, 171.8, 171.9

Abbreviations: ICCC3, International Classification of Childhood Cancer, Third Edition; ICD-9, International Classification of Diseases, Ninth Revision; ICD-10, International Classification of Diseases, Tenth Revision; NA, not applicable.

*The GBD study does not currently estimate burden attributable to any risk factors for these cancers, so these cancers are not shown in results for this analysis. These cancers are included in this table for completeness and accuracy in reporting GBD cancer estimation methods.

[i] Chronic lymphoid leukaemia is only modeled for ages 20 years and above in GBD. ICD codes (ICD-9: 204.1, 204.10, 204.11, and 204.12; ICD-10: C91.1, C91.10, C91.11, and C91.12) under 20 years are redistributed (see Section "6. Redistribution" on pg. 29 for more information) to "Acute lymphoid leukaemia", while these ICD codes over 20 years old are mapped directly to "Chronic lymphoid leukaemia".

4. Age/sex splitting. In the fourth data processing step (#4 in in Appendix figure 1 cancer registry data are standardised to the GBD age groups. For each cancer, the minimum age group estimated was determined as the youngest age-group where SEER reported at least 50 cases over the period 1990 to 2015.[6] Global age-specific incidence rates are generated using hospital inpatient data as described in Section 4.3 of the Supplementary Appendix 1 to the GBD 2019 paper "Global burden of 369 diseases and injuries in 204 countries and territories, 1990–2019: a systematic analysis for the Global Burden of Disease Study 2019".[3] Reference age-specific mortality rates were generated using aggregated deaths from processed VR data, using the approach described in Section 2.5 of the aforementioned GBD 2019 paper.[3] For incidence or mortality datasets that require age-splitting, age-specific proportions are then generated by applying the reference age-specific rates to the registry population to produce the expected number of cases (or deaths for a mortality dataset) for that registry by age. The expected number of cases (or deaths) for each sex, age, and cancer were normalised to 1, creating final, age-specific proportions. These proportions were then applied to the total number of cases (or deaths) by sex and cancer to get the GBD age group-specific number of cases (or deaths) related to that dataset.

In the rare case that the cancer registry only contains data for both sexes combined, the age-specific cases or deaths are split and reassigned to separate sexes using the same weights that are used for the age-splitting process. Starting from the expected number of deaths, global proportions are generated by sex for each age. For example, if for ages 15-19 years old there are 6 expected deaths for males from cause of death data and 4 expected deaths for females, then 60% of the combined-sex deaths for ages 15-19 years would be assigned to males and the remaining 40% would be assigned to females.

5. Cause disaggregation. In the fifth step (#5 in Appendix figure 1), data for cause entries that are aggregates of GBD causes were redistributed across those GBD causes. Examples of these aggregated causes include some cancer registries reporting ICD-10 codes C00-C14 together as "lip, oral cavity, and pharyngeal cancer". These groups are broken down into subcauses that can be individually mapped to single GBD causes. In this example, the more specific ICD-10 codes within C00-C14 are "lip and oral cavity cancer" (C00-C08), "nasopharynx cancer" (C11), "cancer of other parts of the pharynx" (C09-C10, C12-C13), and "Malignant neoplasm of other and ill-defined sites in the lip, oral cavity, and pharynx" (C14). To redistribute the data, weights were created using the same "rate-applied-to-population" method employed in age-sex splitting (see step four above). For the undefined code (C14 in the example) an "average all cancer" weight was used, calculated on the high-quality cancer registry data from SEER/NORDCAN/CI5 by dividing the sum of the cases across these registries by the combined population across these registries. Then, proportions were generated by subcause for each aggregate cause as in the sex-splitting example above (see step four). The total number of cases from the aggregated group (C00-C14) was recalculated for each subgroup and the undefined code (C14). C14 was then redistributed as a "garbage code" in step six. For two exceptions, C44 (non-melanoma skin cancer) and C46 (Kaposi's sarcoma), fixed proportions were used to redistribute into GBD causes. C46 entries were primarily redistributed to HIV according to age (100% for age <15 years, 95% for age 15-49 years, and 90% for age ≥50 years), with the remainder redistributed to the GBD cancer cause "Other malignant neoplasms".

*6. Redistribution***.** In the sixth step (#6 in in Appendix figure 1), unspecified ICD codes ("garbage codes") such as "ill-defined cancer site" (for example, C76 or C80) are redistributed across relevant causes estimated within the GBD hierarchy. Redistribution of cancer registry incidence and mortality data mirrored the process of the redistribution used in the cause of death database and utilised the same

redistribution maps as specified in Section 2.4 of the Supplementary Appendix 1 to the GBD 2019 Diseases and Injuries capstone, "Global burden of 369 diseases and injuries in 204 countries and territories, 1990–2019: a systematic analysis for the Global Burden of Disease Study 2019".[3] Sources and targets of garbage codes can be found in eTable 5 of the Supplementary Appendix to "Cancer Incidence, Mortality, Years of Life Lost, Years Lived with Disability, and Disability-Adjusted Life Years for 29 Cancer Groups from 2010 to 2019: A Systematic Analysis of Cancer Burden Globally, Nationally, and by Socio-demographic Index for the Global Burden of Disease Study 2019".[1]

7. Removal of duplicates. In the seventh step (#7 in in Appendix figure 1), duplicate or redundant data sources were removed from the processed cancer registry dataset. Duplicate sources were present if, for example, a cancer registry was part of the CI5 database but we also had data from that registry directly. Redundancies occurred and were removed as described in "Cancer Incidence Data Sources", where more detailed data were available, or when national registry data could replace regionally representative data. From here, two parallel selection processes were run; one to generate input data for the mortality-to-incidence ratio (MIR) models, and one to generate incidence for final mortality estimation. When creating the final incidence input, higher priority was given to registry data from the most standardised source; whereas for the MIR model input, only sources that reported both incidence and mortality were used.

8. Combine matching incidence and mortality data and model MIRs. In the eighth step (#8 in Appendix figure 1), the processed incidence and mortality data from cancer registries were matched by cancer cause, age, sex, year, and location to generate MIRs. The resulting MIRs were used as input for a three-step modelling approach using the general GBD spatiotemporal Gaussian process regression (ST-GPR)[4] approach, with the Healthcare Access and Quality (HAQ) Index as a covariate in the linear mixed effects model using logit transformed MIR as outcome.[20]

$$\text{logit}\left(MIR_{c,a,s,t}\right) = \alpha + \beta_1(HAQIndex_{c,t}) + \sum_{a}^{A} \beta_2 I_a + \beta_3 I_s + \epsilon_{c,a,s,t}$$

MIR: mortality-to-incidence ratio
c: country (or subnational for subnationally modeled locations), a: age group, t: time (years); s: sex
HAQIndex: Healthcare Access and Quality Index
I: indicator variable
$\epsilon_{c,a,s,t}$: error term

Information on ST-GPR can be found in "Section 4.3.3: Spatiotemporal Gaussian process regression (ST-GPR) modelling" in Supplementary Appendix 1 to "Global burden of 369 diseases and injuries in 204 countries and territories, 1990–2019: a systematic analysis for the global burden of disease study 2019".[3] Predictions were made without the random effects. The ST-GPR model has three main hyper-parameters that control for smoothing across time, age, and geography.[3] These hyper-parameters were adjusted for GBD 2019 in order to improve model performance in locations with sparse data. The time adjustment parameter lambda (λ) aims to borrow strength from neighboring time points (ie, the value in this year is highly correlated with the value in the previous year but less so further back in time). For GBD 2019, lambda was lowered from 2 to 0.05, increasing the weight of more distant years. The age adjustment parameter omega (ω) borrows strength from data in neighboring age groups and was lowered from 1.0 to 0.5, increasing the weight of more distant age groups. The space adjustment parameter zeta (ξ) aims to borrow strength across the hierarchy of geographical locations. Zeta was lowered from 0.95 to 0.01, reducing the weight of more distant geographical data at the region or super-region level. For the remaining parameters in the Gaussian process regression, we lowered the amplitude from 2 to 1 (reducing fluctuation from the mean function) and reduced the scale value from 15 to 10 (reducing the time distance over which points are correlated).

Data-cleaning steps for MIR estimation were similar to those for GBD 2017. For each cancer, MIRs from locations in HAQ Index quintiles 1-4 were dropped if they were below the median of MIRs from locations in HAQ Index quintile 5. We also dropped MIRs from locations in HAQ Index quintiles 1-4 if the MIRs were above an outlier threshold calculated as the third quartile + 1.5 * IQR (inter-quartile range). We dropped all MIR data that were based on fewer than 15 incident cases to avoid excessive variation in the ratio due to small numbers (this threshold was 25 cases in GBD 2017, but was lowered in GBD 2019 in order to include additional data). An exception to this threshold was made for mesothelioma and acute myeloid leukaemia, where instead we dropped MIRs that were based on fewer than ten cases because of lower data availability for these two cancers. For the lower end of the age spectrum where cancers are generally rarer, we also aggregated incidence and mortality to the youngest five-year age bin where SEER[6] reported at least 50 cases from 1990 to 2015, to avoid unstable MIR predictions in young age groups because of too few data. The MIR estimates in this SEER-based minimum age-bin were then copied down to all younger GBD age groups estimated for that cancer.

Since MIRs can be above 1, especially in older age groups and cancers with low cure rates, we used the 95th percentile (by age group) of the cleaned dataset (detailed above) to cap the MIR input data. These "upper cap" values were used to allow MIRs over 1 in some age groups but to constrain the MIRs to a maximum level. Any MIR values over this cap were Winsorised to the cap value. To run the logit model, the input data were first divided by the upper caps to get proportional data ranging from 0 to 1. Model predictions from ST-GPR were then rescaled back by multiplying them by the upper caps. To constrain the MIRs at the lower end, we used the fifth percentile of the cancer and age-specific cleaned MIR input data to Winsorise all model predictions below this lower cap.

9. Generate mortality estimates from incidence and MIRs. Final estimated MIRs were matched with the cleaned cancer registry incidence dataset finalised in the ninth step (#9 in Appendix figure 1) to generate mortality estimates (#10 in Appendix figure 1):

$$MIR_{estimates} * \ incidence_{registry} = mortality_{CR\ inputs}$$

These mortality estimates were then smoothed by a Bayesian noise-reduction algorithm (to deal with zero counts; this is also applied to the VR and VA data), as specified in Section 2.14 of the Supplemenatary Appendix 1 to the GBD 2019 paper "Global burden of 369 diseases and injuries in 204 countries and territories, 1990–2019: a systematic analysis for the Global Burden of Disease Study 2019".[3] These data were uploaded into the CoD database as CR data (#11 in Appendix figure 1). Cancer-specific mortality modelling then followed the general CODEm process[21] using the totality of VA, VR, and CR data.

Cause of death database formatting
Formatting of data sources for the cause of death (CoD) database, including VR and VA data, is similar to many of the steps outlined above for CR data (#11 in Appendix figure 1) and is described in Section 2 of the Supplementary Appendix 1 to the GBD 2019 paper "Global burden of 369 diseases and injuries in 204 countries and territories, 1990–2019: a systematic analysis for the Global Burden of Disease Study 2019".[3]

VA data may not capture cancer deaths as accurately or comprehensively as cancer registries or vital registration systems, but provides a useful contribution to cancer models in locations without VR or CR data. Additional processing and restrictions are performed on VA to ensure quality standards and feasible inputs. More details on VA data processing are provided in the appendix noted above, particularly Sections 2.2 (VA overview), 2.10 (VA cause restrictions), 2.14 (noise reduction), 2.15 (outlier identification), and 2.16 (data quality ratings).

Appendix Table 3: Restrictions on age and sex by each cancer type in GBD 2019

Cause	Minimum age modelled in GBD 2019 (years)	Maximum age modelled in GBD 2019 (years)	Sex restrictions
Bladder cancer	15	95+	None
Breast cancer	15	95+	None
Cervical cancer	15	95+	Females Only
Colon and rectum cancer	5	95+	None
Gallbladder and biliary tract cancer	20	95+	None
Kidney cancer	0	95+	None
Larynx cancer	20	95+	None
Leukaemia	0	95+	None
Lip and oral cavity cancer	5	95+	None
Liver cancer	0	95+	None
Mesothelioma	20	95+	None
Multiple myeloma	20	95+	None
Nasopharynx cancer	5	95+	None
Non-Hodgkin lymphoma	1	95+	None
Oesophageal cancer	20	95+	None
Other pharynx cancer	20	95+	None
Ovarian cancer	5	95+	Females Only
Pancreatic cancer	15	95+	None
Prostate cancer	20	95+	Males Only
Stomach cancer	15	95+	None
Thyroid cancer	5	95+	None
Tracheal, bronchus, and lung cancer	10	95+	None
Uterine cancer	20	95+	Females Only

CODEm models
Mortality estimates for each cancer were generated using the GBD Cause of Death Ensemble model (CODEm, #12 in Appendix figure 1) approach, the methods of which have been described in previous

publications.[3,21] Additional details are specified in Section 3.1 of the Supplementary Appendix 1 to the GBD 2019 paper "Global burden of 369 diseases and injuries in 204 countries and territories, 1990–2019: a systematic analysis for the Global Burden of Disease Study 2019".[3] In brief, the CODEm approach is based on several principles: that all types of available data should be used, even if data quality varies; that a diverse set of plausible models with different combinations of covariates should be evaluated; that both individual models and the overall ensemble models should be tested for their predictive validity; and that the best model or sets of models should be chosen based on the out–of-sample predictive validity.

Covariates are provided for potential use in the ensemble based on a possible predictive relationship between the covariate and the specific cancer mortality, with an expected level and direction of association. Generally, Level 1 covariates have a proven strong relationship with the outcome, such as aetiological or biological roles. Level 2 covariates have a strong relationship but not a known direct biological link. Level 3 covariates have a relationship that may be more distal in the causal chain, or are mediated through Level 1 or 2 covariates.[21] The covariates provided to CODEm, as well as their level and direction, differ by cause and sex.

To generate an ensemble model, CODEm generates submodels that evaluate all plausible relationships between covariates and the response variable. Three additive components of data variance are used in CODEm: sampling variance, non-sampling variance, and garbage code redistribution variance. Model performance of all models is evaluated through out-of-sample predictive validity tests. Ensemble models are constructed from the individual models, with the contribution of individual models to the ensemble weighted by the basis of their predictive validity ranking. The final ensemble contains 1000 draws from these individual component models, from which a mean estimate and a 95% uncertainty interval are calculated. The 95% uncertainty interval represents the 0.025 and 0.975 quantiles of the draws.

Appendix Table 4: GBD 2019 covariates and level of covariates used in cause of death modelling for cancer types estimated

Cause	Sex	Covariate	Level	Direction
Bladder cancer	Male	Cumulative Cigarettes (10 Years)	2	1
Bladder cancer	Male	Diabetes Fasting Plasma Glucose (mmol/L), age-standardised 25+	2	1
Bladder cancer	Male	LDI (I$ per capita)	3	1
Bladder cancer	Male	Smoking Prevalence	1	1
Bladder cancer	Male	Schistosomiasis Prevalence Results	1	1
Bladder cancer	Male	Log-transformed SEV scalar: Bladder C	1	1
Bladder cancer	Male	Socio-demographic Index	3	1
Bladder cancer	Male	Healthcare Access and Quality Index	2	-1
Bladder cancer	Male	Age- and sex-specific SEV for Low fruit	3	1
Bladder cancer	Male	Age- and sex-specific SEV for Low vegetables	2	1
Bladder cancer	Male	Liters of alcohol consumed per capita	2	1
Bladder cancer	Female	Cumulative Cigarettes (10 Years)	2	1
Bladder cancer	Female	Diabetes Fasting Plasma Glucose (mmol/L), age-standardised 25+	2	1
Bladder cancer	Female	LDI (I$ per capita)	3	1
Bladder cancer	Female	Smoking Prevalence	1	1
Bladder cancer	Female	Schistosomiasis Prevalence Results	1	1

Bladder cancer	Female	Log-transformed SEV scalar: Bladder C	1	1
Bladder cancer	Female	Socio-demographic Index	3	1
Bladder cancer	Female	Healthcare Access and Quality Index	2	-1
Bladder cancer	Female	Age- and sex-specific SEV for Low fruit	3	1
Bladder cancer	Female	Age- and sex-specific SEV for Low vegetables	2	1
Bladder cancer	Female	Liters of alcohol consumed per capita	2	1
Breast cancer	Male	Cumulative cigarettes (10 years)	2	1
Breast cancer	Male	Cumulative cigarettes (20 years)	2	1
Breast cancer	Male	Diabetes Fasting Plasma Glucose (mmol/L), age-standardised 25+	2	1
Breast cancer	Male	Mean BMI	1	1
Breast cancer	Male	Total Fertility Rate	1	1
Breast cancer	Male	Socio-demographic Index	2	1
Breast cancer	Male	Age- and sex- specific SEV for low fruit	1	1
Breast cancer	Male	Liters of alcohol consumed per capita	1	1
Breast cancer	Male	Healthcare Access and Quality Index	2	-1
Breast cancer	Male	Age- and sex- specific SEV for low fruit	2	1
Breast cancer	Male	Age- and sex- specific SEV for Low vegetables	2	1
Breast cancer	Male	Liters of alcohol consumed per capita	1	1
Breast cancer	Female	Age-specific fertility rate	2	-1
Breast cancer	Female	Cumulative cigarettes (10 years)	2	1
Breast cancer	Female	Cumulative cigarettes (20 years)	2	1
Breast cancer	Female	Diabetes Fasting Plasma Glucose (mmol/L), age-standardised 25+	2	1
Breast cancer	Female	LDI (I$ per capita)	3	-1
Breast cancer	Female	Mean BMI	1	1
Breast cancer	Female	Smoking Prevalence	2	1
Breast cancer	Female	Total Fertility Rate	2	-1
Breast cancer	Female	Log-transformed SEV scalar: Breast C	1	1
Breast cancer	Female	Socio-demographic Index	3	1
Breast cancer	Female	Healthcare Access and Quality Index	2	-1
Breast cancer	Female	Age- and sex- specific SEV for low fruit	2	1
Breast cancer	Female	Age- and sex- specific SEV for Low vegetables	2	1
Breast cancer	Female	Liters of alcohol consumed per capita	1	1
Cervical cancer	Female	Age-specific fertility rate	2	1
Cervical cancer	Female	Cumulative Cigarettes (5 Years)	1	1
Cervical cancer	Female	Education (years per capita)	3	-1
Cervical cancer	Female	LDI (I$ per capita)	3	-1
Cervical cancer	Female	Smoking Prevalence	2	1
Cervical cancer	Female	Total Fertility Rate	2	1
Cervical cancer	Female	Socio-demographic Index	3	-1

Cervical cancer	Female	HIV age-standardised prevalence	1	1
Cervical cancer	Female	Healthcare Access and Quality Index	2	-1
Cervical cancer	Female	Age- and sex- specific SEV for low fruit	2	1
Cervical cancer	Female	Age- and sex- specific SEV for Low vegetables	2	1
Colon and rectum cancer	Male	Tobacco (cigarettes per capita)	1	1
Colon and rectum cancer	Male	Cumulative cigarettes (20 years)	2	1
Colon and rectum cancer	Male	Diabetes Fasting Plasma Glucose (mmol/L), age-standardised 25+	2	1
Colon and rectum cancer	Male	Education (years per capita)	3	-1
Colon and rectum cancer	Male	LDI (I$ per capita)	3	1
Colon and rectum cancer	Male	Mean BMI	1	1
Colon and rectum cancer	Male	Log-transformed SEV scalar: Colorect C	1	1
Colon and rectum cancer	Male	Socio-demographic Index	3	1
Colon and rectum cancer	Male	pufa adjusted(percent)	2	-1
Colon and rectum cancer	Male	Healthcare Access and Quality Index	3	-1
Colon and rectum cancer	Male	Total Physical Activity (MET-min/week), Age-specific	1	-1
Colon and rectum cancer	Male	Age- and sex- specific SEV for low fruit	3	1
Colon and rectum cancer	Male	Age- and sex- specific SEV for Low vegetables	2	1
Colon and rectum cancer	Male	Age- and sex-specific SEV for Low nuts and seeds	3	1
Colon and rectum cancer	Male	Age- and sex-specific SEV for Low milk	3	1
Colon and rectum cancer	Male	Age- and sex-specific SEV for High red meat	1	1
Colon and rectum cancer	Male	Age- and sex-specific SEV for Low fibre	2	1
Colon and rectum cancer	Male	Age- and sex-specific SEV for Low calcium	2	1
Colon and rectum cancer	Male	Liters of alcohol consumed per capita	2	1
Colon and rectum cancer	Female	Tobacco (cigarettes per capita)	1	1
Colon and rectum cancer	Female	Cumulative cigarettes (5 years)	2	1
Colon and rectum cancer	Female	Diabetes Fasting Plasma Glucose (mmol/L), age-standardised 25+	2	1
Colon and rectum cancer	Female	Education (years per capita)	3	-1
Colon and rectum cancer	Female	LDI (I$ per capita)	3	1
Colon and rectum cancer	Female	Mean BMI	1	1
Colon and rectum cancer	Female	Log-transformed SEV scalar: Colorect C	1	1
Colon and rectum cancer	Female	Socio-demographic Index	3	1
Colon and rectum cancer	Female	pufa adjusted(percent)	2	-1
Colon and rectum cancer	Female	Healthcare Access and Quality Index	3	-1
Colon and rectum cancer	Female	Total Physical Activity (MET-min/week), Age-specific	1	-1
Colon and rectum cancer	Female	Age- and sex- specific SEV for low fruit	3	1
Colon and rectum cancer	Female	Age- and sex- specific SEV for Low vegetables	2	1
Colon and rectum cancer	Female	Age- and sex-specific SEV for Low nuts and seeds	3	1

Colon and rectum cancer	Female	Age- and sex-specific SEV for Low milk	3	1
Colon and rectum cancer	Female	Age- and sex-specific SEV for High red meat	1	1
Colon and rectum cancer	Female	Age- and sex-specific SEV for Low fibre	2	1
Colon and rectum cancer	Female	Age- and sex-specific SEV for Low calcium	2	1
Colon and rectum cancer	Female	Liters of alcohol consumed per capita	2	1
Gallbladder and biliary tract cancer	Male	Tobacco (cigarettes per capita)	2	1
Gallbladder and biliary tract cancer	Male	Cumulative cigarettes (10 years)	2	1
Gallbladder and biliary tract cancer	Male	Cumulative Cigarettes (5 Years)	2	1
Gallbladder and biliary tract cancer	Male	Diabetes Age-Standardised Prevalence (proportion)	2	1
Gallbladder and biliary tract cancer	Male	Education (years per capita)	3	-1
Gallbladder and biliary tract cancer	Male	LDI (I$ per capita)	3	1
Gallbladder and biliary tract cancer	Male	Mean BMI	1	1
Gallbladder and biliary tract cancer	Male	Smoking Prevalence	2	1
Gallbladder and biliary tract cancer	Male	Log-transformed SEV scalar: Gallblad C	1	1
Gallbladder and biliary tract cancer	Male	Socio-demographic Index	3	-1
Gallbladder and biliary tract cancer	Male	Healthcare Access and Quality Index	2	-1
Gallbladder and biliary tract cancer	Male	Age- and sex- specific SEV for low fruit	2	1
Gallbladder and biliary tract cancer	Male	Age- and sex- specific SEV for Low vegetables	2	1
Gallbladder and biliary tract cancer	Male	Liters of alcohol consumed per capita	2	1
Gallbladder and biliary tract cancer	Female	Tobacco (cigarettes per capita)	2	1
Gallbladder and biliary tract cancer	Female	Cumulative cigarettes (10 years)	2	1
Gallbladder and biliary tract cancer	Female	Cumulative Cigarettes (5 Years)	2	1
Gallbladder and biliary tract cancer	Female	Diabetes Age-Standardised Prevalence (proportion)	2	1
Gallbladder and biliary tract cancer	Female	Education (years per capita)	3	-1
Gallbladder and biliary tract cancer	Female	LDI (I$ per capita)	3	1
Gallbladder and biliary tract cancer	Female	Mean BMI	1	1

Gallbladder and biliary tract cancer	Female	Smoking Prevalence	2	1
Gallbladder and biliary tract cancer	Female	Log-transformed SEV scalar: Gallblad C	1	1
Gallbladder and biliary tract cancer	Female	Socio-demographic Index	3	-1
Gallbladder and biliary tract cancer	Female	Healthcare Access and Quality Index	2	-1
Gallbladder and biliary tract cancer	Female	Age- and sex- specific SEV for low fruit	2	1
Gallbladder and biliary tract cancer	Female	Age- and sex- specific SEV for Low vegetables	2	1
Gallbladder and biliary tract cancer	Female	Liters of alcohol consumed per capita	2	1
Kidney cancer	Male	Tobacco (cigarettes per capita)	1	1
Kidney cancer	Male	Cumulative cigarettes (10 years)	1	1
Kidney cancer	Male	Diabetes Age-Standardised Prevalence (proportion)	2	1
Kidney cancer	Male	Education (years per capita)	3	-1
Kidney cancer	Male	LDI (I$ per capita)	3	1
Kidney cancer	Male	Mean BMI	1	1
Kidney cancer	Male	Systolic Blood Pressure (mmHg)	2	1
Kidney cancer	Male	Log-transformed SEV scalar: Kidney C	1	1
Kidney cancer	Male	Socio-demographic Index	3	1
Kidney cancer	Male	Healthcare Access and Quality Index	2	-1
Kidney cancer	Male	Liters of alcohol consumed per capita	2	1
Kidney cancer	Female	Tobacco (cigarettes per capita)	1	1
Kidney cancer	Female	Cumulative cigarettes (10 years)	1	1
Kidney cancer	Female	Diabetes Age-Standardised Prevalence (proportion)	2	1
Kidney cancer	Female	Education (years per capita)	3	-1
Kidney cancer	Female	LDI (I$ per capita)	3	1
Kidney cancer	Female	Mean BMI	1	1
Kidney cancer	Female	Systolic Blood Pressure (mmHg)	2	1
Kidney cancer	Female	Log-transformed SEV scalar: Kidney C	1	1
Kidney cancer	Female	Socio-demographic Index	3	1
Kidney cancer	Female	Healthcare Access and Quality Index	2	-1
Kidney cancer	Female	Liters of alcohol consumed per capita	2	1
Larynx cancer	Male	Cumulative cigarettes (10 years)	2	1
Larynx cancer	Male	Cumulative cigarettes (20 years)	2	1
Larynx cancer	Male	LDI (I$ per capita)	3	1
Larynx cancer	Male	Population Density (over 1000 ppl/sqkm, proportion)	2	1
Larynx cancer	Male	Smoking Prevalence	2	1
Larynx cancer	Male	Log-transformed SEV scalar: Larynx C	1	1

Larynx cancer	Male	Socio-demographic Index	3	1
Larynx cancer	Male	Healthcare Access and Quality Index	2	-1
Larynx cancer	Male	Asbestos consumption (metric tons per year per capita)	2	1
Larynx cancer	Male	Age- and sex- specific SEV for low fruit	2	1
Larynx cancer	Male	Age- and sex- specific SEV for Low vegetables	3	1
Larynx cancer	Male	Liters of alcohol consumed per capita	1	1
Larynx cancer	Female	Cumulative cigarettes (10 years)	2	1
Larynx cancer	Female	Cumulative cigarettes (20 years)	2	1
Larynx cancer	Female	LDI (I$ per capita)	3	1
Larynx cancer	Female	Population Density (over 1000 ppl/sqkm, proportion)	2	1
Larynx cancer	Female	Smoking Prevalence	2	1
Larynx cancer	Female	Log-transformed SEV scalar: Larynx C	1	1
Larynx cancer	Female	Socio-demographic Index	3	1
Larynx cancer	Female	Healthcare Access and Quality Index	2	-1
Larynx cancer	Female	Asbestos consumption (metric tons per year per capita)	2	1
Larynx cancer	Female	Age- and sex- specific SEV for low fruit	3	1
Larynx cancer	Female	Age- and sex- specific SEV for Low vegetables	2	1
Larynx cancer	Female	Liters of alcohol consumed per capita	1	1
Leukaemia	Male	Tobacco (cigarettes per capita)	2	1
Leukaemia	Male	Cumulative cigarettes (10 years)	2	1
Leukaemia	Male	Cumulative cigarettes (20 years)	2	1
Leukaemia	Male	Education (years per capita)	3	-1
Leukaemia	Male	LDI (I$ per capita)	3	1
Leukaemia	Male	Mean BMI	2	1
Leukaemia	Male	Log-transformed SEV scalar: Leukaemia	1	1
Leukaemia	Male	Log-transformed age-standardised SEV scalar: Leukaemia	1	1
Leukaemia	Male	Socio-demographic Index	3	-1
Leukaemia	Male	Healthcare Access and Quality Index	2	-1
Leukaemia	Male	Liters of alcohol consumed per capita	2	1
Leukaemia	Female	Tobacco (cigarettes per capita)	2	1
Leukaemia	Female	Cumulative cigarettes (10 years)	2	1
Leukaemia	Female	Cumulative cigarettes (20 years)	2	1
Leukaemia	Female	Education (years per capita)	3	-1
Leukaemia	Female	LDI (I$ per capita)	3	1
Leukaemia	Female	Mean BMI	2	1
Leukaemia	Female	Log-transformed SEV scalar: Leukaemia	1	1
Leukaemia	Female	Log-transformed age-standardised SEV scalar: Leukaemia	1	1

Leukaemia	Female	Socio-demographic Index	3	-1
Leukaemia	Female	Healthcare Access and Quality Index	2	-1
Leukaemia	Female	Liters of alcohol consumed per capita	2	1
Lip and oral cavity cancer	Male	Tobacco (cigarettes per capita)	1	1
Lip and oral cavity cancer	Male	Cumulative cigarettes (10 years)	1	1
Lip and oral cavity cancer	Male	Cumulative cigarettes (20 years)	1	1
Lip and oral cavity cancer	Male	Education (years per capita)	3	-1
Lip and oral cavity cancer	Male	LDI (I$ per capita)	3	1
Lip and oral cavity cancer	Male	Log-transformed SEV scalar: Lip oral C	1	1
Lip and oral cavity cancer	Male	Socio-demographic Index	3	1
Lip and oral cavity cancer	Male	Healthcare Access and Quality Index	2	-1
Lip and oral cavity cancer	Male	Age- and sex- specific SEV for low fruit	2	1
Lip and oral cavity cancer	Male	Age- and sex- specific SEV for Low vegetables	2	1
Lip and oral cavity cancer	Male	Age- and sex- specific SEV for High red meat	2	1
Lip and oral cavity cancer	Male	Liters of alcohol consumed per capita	1	1
Lip and oral cavity cancer	Female	Tobacco (cigarettes per capita)	1	1
Lip and oral cavity cancer	Female	Cumulative cigarettes (10 years)	1	1
Lip and oral cavity cancer	Female	Cumulative cigarettes (20 years)	1	1
Lip and oral cavity cancer	Female	Education (years per capita)	3	-1
Lip and oral cavity cancer	Female	LDI (I$ per capita)	3	1
Lip and oral cavity cancer	Female	Log-transformed SEV scalar: Lip oral C	1	1
Lip and oral cavity cancer	Female	Socio-demographic Index	3	1
Lip and oral cavity cancer	Female	Healthcare Access and Quality Index	2	-1
Lip and oral cavity cancer	Female	Age- and sex- specific SEV for low fruit	2	1
Lip and oral cavity cancer	Female	Age- and sex- specific SEV for Low vegetables	2	1
Lip and oral cavity cancer	Female	Age- and sex- specific SEV for High red meat	2	1
Lip and oral cavity cancer	Female	Liters of alcohol consumed per capita	1	1
Liver cancer	Male	Tobacco (cigarettes per capita)	2	1
Liver cancer	Male	Cumulative cigarettes (20 years)	2	1
Liver cancer	Male	Diabetes Fasting Plasma Glucose (mmol/L), age-standardised 25+	2	1
Liver cancer	Male	Education (years per capita)	3	-1
Liver cancer	Male	LDI (I$ per capita)	3	-1
Liver cancer	Male	Mean BMI	2	1
Liver cancer	Male	Log-transformed SEV scalar: Liver C	1	1
Liver cancer	Male	Socio-demographic Index	3	-1
Liver cancer	Male	HIV age-standardised prevalence	1	1
Liver cancer	Male	Healthcare Access and Quality Index	2	-1
Liver cancer	Male	Hepatitis B 3-dose coverage (proportion)	2	-1
Liver cancer	Male	Intravenous drug use (age-standardised proportion)	2	1

Liver cancer	Male	Hepatitis B vaccine coverage (proportion), aged through time	2	-1
Liver cancer	Male	Age- and sex-specific SEV for High red meat	3	1
Liver cancer	Male	Hepatitis B Seroprevalence (HBsAg) age standardised	1	1
Liver cancer	Male	Hepatitis C Seroprevalence (anti-HCV) age standardised	1	1
Liver cancer	Male	Liters of alcohol consumed per capita	1	1
Liver cancer	Female	Tobacco (cigarettes per capita)	2	1
Liver cancer	Female	Cumulative cigarettes (20 years)	2	1
Liver cancer	Female	Diabetes Fasting Plasma Glucose (mmol/L), age-standardised 25+	2	1
Liver cancer	Female	Education (years per capita)	3	-1
Liver cancer	Female	LDI (I$ per capita)	3	-1
Liver cancer	Female	Mean BMI	2	1
Liver cancer	Female	Log-transformed SEV scalar: Liver C	1	1
Liver cancer	Female	Socio-demographic Index	3	-1
Liver cancer	Female	HIV age-standardised prevalence	1	1
Liver cancer	Female	Healthcare Access and Quality Index	2	-1
Liver cancer	Female	Hepatitis B 3-dose coverage (proportion)	2	-1
Liver cancer	Female	Intravenous drug use (age-standardised proportion)	2	1
Liver cancer	Female	Hepatitis B vaccine coverage (proportion), aged through time	2	-1
Liver cancer	Female	Age- and sex-specific SEV for High red meat	3	1
Liver cancer	Female	Hepatitis B Seroprevalence (HBsAg) age standardised	1	1
Liver cancer	Female	Hepatitis C Seroprevalence (anti-HCV) age standardised	1	1
Liver cancer	Female	Liters of alcohol consumed per capita	1	1
Mesothelioma	Male	Cumulative Cigarettes (5 Years)	2	1
Mesothelioma	Male	Education (years per capita)	3	-1
Mesothelioma	Male	Gold production (binary)	2	1
Mesothelioma	Male	LDI (I$ per capita)	3	-1
Mesothelioma	Male	Indoor Air Pollution (All Cooking Fuels)	2	1
Mesothelioma	Male	Population Density (over 1000 ppl/sqkm, proportion)	2	1
Mesothelioma	Male	Smoking Prevalence	1	1
Mesothelioma	Male	Log-transformed SEV scalar: Mesothel	1	1
Mesothelioma	Male	Log-transformed age-standardised SEV scalar: Mesothel	1	1
Mesothelioma	Male	Socio-demographic Index	3	1
Mesothelioma	Male	Healthcare Access and Quality Index	2	-1
Mesothelioma	Male	Asbestos consumption (metric tons per year per capita)	1	1

Mesothelioma	Female	Cumulative Cigarettes (5 Years)	2	1
Mesothelioma	Female	Education (years per capita)	3	-1
Mesothelioma	Female	Gold production (binary)	2	1
Mesothelioma	Female	LDI (I$ per capita)	3	-1
Mesothelioma	Female	Indoor Air Pollution (All Cooking Fuels)	2	1
Mesothelioma	Female	Population Density (over 1000 ppl/sqkm, proportion)	2	1
Mesothelioma	Female	Smoking Prevalence	1	1
Mesothelioma	Female	Socio-demographic Index	3	1
Mesothelioma	Female	Healthcare Access and Quality Index	2	-1
Mesothelioma	Female	Asbestos consumption (metric tons per year per capita)	1	1
Multiple myeloma	Male	Tobacco (cigarettes per capita)	1	1
Multiple myeloma	Male	Education (years per capita)	3	-1
Multiple myeloma	Male	LDI (I$ per capita)	3	1
Multiple myeloma	Male	Mean BMI	2	1
Multiple myeloma	Male	Sanitation (proportion with access)	2	-1
Multiple myeloma	Male	Smoking Prevalence	1	1
Multiple myeloma	Male	Improved Water Source (proportion with access)	2	-1
Multiple myeloma	Male	Socio-demographic Index	3	1
Multiple myeloma	Male	Healthcare Access and Quality Index	2	-1
Multiple myeloma	Male	Age- and sex- specific SEV for low fruit	2	1
Multiple myeloma	Male	Age- and sex- specific SEV for Low vegetables	2	1
Multiple myeloma	Male	Age- and sex-specific SEV for High red meat	2	1
Multiple myeloma	Male	Liters of alcohol consumed per capita	1	1
Multiple myeloma	Female	Tobacco (cigarettes per capita)	1	1
Multiple myeloma	Female	Education (years per capita)	3	-1
Multiple myeloma	Female	LDI (I$ per capita)	3	1
Multiple myeloma	Female	Mean BMI	2	1
Multiple myeloma	Female	Sanitation (proportion with access)	2	-1
Multiple myeloma	Female	Smoking Prevalence	1	1
Multiple myeloma	Female	Improved Water Source (proportion with access)	2	-1
Multiple myeloma	Female	Socio-demographic Index	3	1
Multiple myeloma	Female	Healthcare Access and Quality Index	2	-1
Multiple myeloma	Female	Age- and sex- specific SEV for low fruit	2	1
Multiple myeloma	Female	Age- and sex- specific SEV for Low vegetables	2	1
Multiple myeloma	Female	Age- and sex-specific SEV for High red meat	2	1
Multiple myeloma	Female	Liters of alcohol consumed per capita	1	1
Nasopharynx cancer	Male	Tobacco (cigarettes per capita)	1	1
Nasopharynx cancer	Male	Cumulative cigarettes (10 years)	1	1
Nasopharynx cancer	Male	Cumulative cigarettes (20 years)	1	1

Nasopharynx cancer	Male	Education (years per capita)	3	-1
Nasopharynx cancer	Male	LDI (I$ per capita)	3	-1
Nasopharynx cancer	Male	Population Density (over 1000 ppl/sqkm, proportion)	2	1
Nasopharynx cancer	Male	Log-transformed SEV scalar: Nasoph C	1	1
Nasopharynx cancer	Male	Socio-demographic Index	3	1
Nasopharynx cancer	Male	Healthcare Access and Quality Index	2	-1
Nasopharynx cancer	Male	Age- and sex- specific SEV for low fruit	3	1
Nasopharynx cancer	Male	Age- and sex- specific SEV for Low vegetables	2	1
Nasopharynx cancer	Male	Liters of alcohol consumed per capita	1	1
Nasopharynx cancer	Female	Tobacco (cigarettes per capita)	1	1
Nasopharynx cancer	Female	Cumulative cigarettes (10 years)	1	1
Nasopharynx cancer	Female	Cumulative cigarettes (20 years)	1	1
Nasopharynx cancer	Female	Education (years per capita)	3	-1
Nasopharynx cancer	Female	LDI (I$ per capita)	3	-1
Nasopharynx cancer	Female	Population Density (over 1000 ppl/sqkm, proportion)	2	1
Nasopharynx cancer	Female	Log-transformed SEV scalar: Nasoph C	1	1
Nasopharynx cancer	Female	Socio-demographic Index	3	1
Nasopharynx cancer	Female	Healthcare Access and Quality Index	2	-1
Nasopharynx cancer	Female	Age- and sex- specific SEV for low fruit	3	1
Nasopharynx cancer	Female	Age- and sex- specific SEV for Low vegetables	2	1
Nasopharynx cancer	Female	Liters of alcohol consumed per capita	1	1
Non-Hodgkin lymphoma	Male	Cumulative cigarettes (10 years)	2	1
Non-Hodgkin lymphoma	Male	Cumulative cigarettes (15 years)	2	1
Non-Hodgkin lymphoma	Male	Cumulative cigarettes (20 years)	2	1
Non-Hodgkin lymphoma	Male	Cumulative Cigarettes (5 Years)	2	1
Non-Hodgkin lymphoma	Male	LDI (I$ per capita)	3	1
Non-Hodgkin lymphoma	Male	Mean BMI	2	1
Non-Hodgkin lymphoma	Male	Smoking Prevalence	2	1
Non-Hodgkin lymphoma	Male	Socio-demographic Index	3	1
Non-Hodgkin lymphoma	Male	Healthcare Access and Quality Index	2	-1
Non-Hodgkin lymphoma	Male	Liters of alcohol consumed per capita	2	1
Non-Hodgkin lymphoma	Female	Cumulative cigarettes (10 years)	2	1
Non-Hodgkin lymphoma	Female	Cumulative cigarettes (15 years)	2	1
Non-Hodgkin lymphoma	Female	Cumulative cigarettes (20 years)	2	1
Non-Hodgkin lymphoma	Female	Cumulative Cigarettes (5 Years)	2	1
Non-Hodgkin lymphoma	Female	LDI (I$ per capita)	3	1
Non-Hodgkin lymphoma	Female	Mean BMI	2	1
Non-Hodgkin lymphoma	Female	Smoking Prevalence	2	1
Non-Hodgkin lymphoma	Female	Total Fertility Rate	3	-1

Non-Hodgkin lymphoma	Female	Socio-demographic Index	3	1
Non-Hodgkin lymphoma	Female	Healthcare Access and Quality Index	2	-1
Non-Hodgkin lymphoma	Female	Liters of alcohol consumed per capita	2	1
Oesophageal cancer	Male	Tobacco (cigarettes per capita)	2	1
Oesophageal cancer	Male	Education (years per capita)	3	-1
Oesophageal cancer	Male	LDI (I$ per capita)	3	1
Oesophageal cancer	Male	Mean BMI	1	1
Oesophageal cancer	Male	Indoor Air Pollution (All Cooking Fuels)	2	1
Oesophageal cancer	Male	Sanitation (proportion with access)	3	-1
Oesophageal cancer	Male	Smoking Prevalence	1	1
Oesophageal cancer	Male	Improved Water Source (proportion with access)	3	-1
Oesophageal cancer	Male	Log-transformed age-standardised SEV scalar: Esophag C	1	1
Oesophageal cancer	Male	Socio-demographic Index	3	1
Oesophageal cancer	Male	Healthcare Access and Quality Index	2	-1
Oesophageal cancer	Male	Age- and sex- specific SEV for low fruit	2	1
Oesophageal cancer	Male	Age- and sex- specific SEV for Low vegetables	2	1
Oesophageal cancer	Male	Liters of alcohol consumed per capita	1	1
Oesophageal cancer	Female	Tobacco (cigarettes per capita)	2	1
Oesophageal cancer	Female	Education (years per capita)	3	-1
Oesophageal cancer	Female	LDI (I$ per capita)	3	1
Oesophageal cancer	Female	Mean BMI	1	1
Oesophageal cancer	Female	Indoor Air Pollution (All Cooking Fuels)	2	1
Oesophageal cancer	Female	Sanitation (proportion with access)	3	-1
Oesophageal cancer	Female	Smoking Prevalence	1	1
Oesophageal cancer	Female	Improved Water Source (proportion with access)	3	-1
Oesophageal cancer	Female	Log-transformed age-standardised SEV scalar: Esophag C	1	1
Oesophageal cancer	Female	Socio-demographic Index	3	1
Oesophageal cancer	Female	Healthcare Access and Quality Index	2	-1
Oesophageal cancer	Female	Age- and sex- specific SEV for low fruit	2	1
Oesophageal cancer	Female	Age- and sex- specific SEV for Low vegetables	2	1
Oesophageal cancer	Female	Liters of alcohol consumed per capita	1	1
Other pharynx cancer	Male	Cumulative cigarettes (5 years)	2	1
Other pharynx cancer	Male	Education (years per capita)	3	-1
Other pharynx cancer	Male	LDI (I$ per capita)	3	1
Other pharynx cancer	Male	Population Density (over 1000 ppl/sqkm, proportion)	2	1
Other pharynx cancer	Male	Population Density (under 150 ppl/sqkm, proportion)	2	1
Other pharynx cancer	Male	Smoking Prevalence	1	1

Other pharynx cancer	Male	Log-transformed SEV scalar: Oth Phar C	1	1
Other pharynx cancer	Male	Socio-demographic Index	3	1
Other pharynx cancer	Male	Healthcare Access and Quality Index	2	-1
Other pharynx cancer	Male	Age- and sex- specific SEV for low fruit	2	1
Other pharynx cancer	Male	Age- and sex- specific SEV for Low vegetables	2	1
Other pharynx cancer	Male	Liters of alcohol consumed per capita	1	1
Other pharynx cancer	Female	Cumulative cigarettes (5 years)	2	1
Other pharynx cancer	Female	Education (years per capita)	3	-1
Other pharynx cancer	Female	LDI (I$ per capita)	3	1
Other pharynx cancer	Female	Population Density (over 1000 ppl/sqkm, proportion)	2	1
Other pharynx cancer	Female	Population Density (under 150 ppl/sqkm, proportion)	2	1
Other pharynx cancer	Female	Smoking Prevalence	1	1
Other pharynx cancer	Female	Log-transformed SEV scalar: Oth Phar C	1	1
Other pharynx cancer	Female	Socio-demographic Index	3	1
Other pharynx cancer	Female	Healthcare Access and Quality Index	2	-1
Other pharynx cancer	Female	Age- and sex- specific SEV for low fruit	2	1
Other pharynx cancer	Female	Age- and sex- specific SEV for Low vegetables	2	1
Other pharynx cancer	Female	Liters of alcohol consumed per capita	1	1
Ovarian cancer	Female	Contraception (Modern) Prevalence (proportion)	2	-1
Ovarian cancer	Female	Cumulative cigarettes (10 years)	2	1
Ovarian cancer	Female	Cumulative cigarettes (20 years)	2	1
Ovarian cancer	Female	Diabetes Age-Standardised Prevalence (proportion)	2	1
Ovarian cancer	Female	Education (years per capita)	3	-1
Ovarian cancer	Female	LDI (I$ per capita)	3	-1
Ovarian cancer	Female	Mean BMI	2	1
Ovarian cancer	Female	Smoking Prevalence	2	1
Ovarian cancer	Female	Total Fertility Rate	2	-1
Ovarian cancer	Female	Log-transformed SEV scalar: Ovary C	1	1
Ovarian cancer	Female	Socio-demographic Index	3	1
Ovarian cancer	Female	energy unadjusted(kcal)	2	1
Ovarian cancer	Female	Healthcare Access and Quality Index	2	-1
Ovarian cancer	Female	Asbestos consumption (metric tons per year per capita)	2	1
Ovarian cancer	Female	Age- and sex- specific SEV for low fruit	3	1
Ovarian cancer	Female	Age- and sex- specific SEV for Low vegetables	3	1
Ovarian cancer	Female	Liters of alcohol consumed per capita	1	1
Pancreatic cancer	Male	Tobacco (cigarettes per capita)	1	1
Pancreatic cancer	Male	Cumulative cigarettes (10 years)	1	1
Pancreatic cancer	Male	Cumulative cigarettes (20 years)	1	1

Pancreatic cancer	Male	Diabetes Fasting Plasma Glucose (mmol/L), age-standardised 25+	2	1
Pancreatic cancer	Male	Diabetes Age-Standardised Prevalence (proportion)	2	1
Pancreatic cancer	Male	Education (years per capita)	3	-1
Pancreatic cancer	Male	LDI (I$ per capita)	3	1
Pancreatic cancer	Male	Mean BMI	1	1
Pancreatic cancer	Male	Log-transformed SEV scalar: Pancreas C	1	1
Pancreatic cancer	Male	Socio-demographic Index	3	1
Pancreatic cancer	Male	energy unadjusted(kcal)	2	1
Pancreatic cancer	Male	Healthcare Access and Quality Index	2	-1
Pancreatic cancer	Male	Age- and sex- specific SEV for low fruit	3	1
Pancreatic cancer	Male	Age- and sex- specific SEV for Low vegetables	3	1
Pancreatic cancer	Male	Age- and sex-specific SEV for High red meat	2	1
Pancreatic cancer	Male	Liters of alcohol consumed per capita	2	1
Pancreatic cancer	Female	Tobacco (cigarettes per capita)	1	1
Pancreatic cancer	Female	Cumulative cigarettes (10 years)	1	1
Pancreatic cancer	Female	Cumulative cigarettes (20 years)	1	1
Pancreatic cancer	Female	Diabetes Fasting Plasma Glucose (mmol/L), age-standardised 25+	2	1
Pancreatic cancer	Female	Diabetes Age-Standardised Prevalence (proportion)	2	1
Pancreatic cancer	Female	Education (years per capita)	3	-1
Pancreatic cancer	Female	LDI (I$ per capita)	3	1
Pancreatic cancer	Female	Mean BMI	1	1
Pancreatic cancer	Female	Log-transformed SEV scalar: Pancreas C	1	1
Pancreatic cancer	Female	Socio-demographic Index	3	1
Pancreatic cancer	Female	energy unadjusted(kcal)	2	1
Pancreatic cancer	Female	Healthcare Access and Quality Index	2	-1
Pancreatic cancer	Female	Age- and sex- specific SEV for low fruit	3	1
Pancreatic cancer	Female	Age- and sex- specific SEV for Low vegetables	3	1
Pancreatic cancer	Female	Age- and sex-specific SEV for High red meat	2	1
Pancreatic cancer	Female	Liters of alcohol consumed per capita	2	1
Prostate cancer	Male	Education (years per capita)	3	-1
Prostate cancer	Male	LDI (I$ per capita)	3	-1
Prostate cancer	Male	Smoking Prevalence	2	1
Prostate cancer	Male	Log-transformed SEV scalar: Prostate C	1	1
Prostate cancer	Male	Socio-demographic Index	3	1
Prostate cancer	Male	Healthcare Access and Quality Index	2	-1
Stomach cancer	Male	Tobacco (cigarettes per capita)	1	1
Stomach cancer	Male	Cumulative cigarettes (20 years)	2	1
Stomach cancer	Male	Education (years per capita)	3	-1
Stomach cancer	Male	LDI (I$ per capita)	3	1

Stomach cancer	Male	Mean BMI	2	1
Stomach cancer	Male	Sanitation (proportion with access)	2	-1
Stomach cancer	Male	Improved Water Source (proportion with access)	2	-1
Stomach cancer	Male	Log-transformed SEV scalar: Stomach C	1	1
Stomach cancer	Male	Age- and sex-specific SEV for Unsafe water	2	1
Stomach cancer	Male	Age- and sex-specific SEV for Unsafe sanitation	2	1
Stomach cancer	Male	Socio-demographic Index	3	-1
Stomach cancer	Male	Healthcare Access and Quality Index	2	-1
Stomach cancer	Male	Diet high in sodium	1	1
Stomach cancer	Male	Age- and sex- specific SEV for low fruit	3	1
Stomach cancer	Male	Age- and sex- specific SEV for Low vegetables	3	1
Stomach cancer	Female	Tobacco (cigarettes per capita)	1	1
Stomach cancer	Female	Cumulative cigarettes (20 years)	2	1
Stomach cancer	Female	Education (years per capita)	3	-1
Stomach cancer	Female	LDI (I$ per capita)	3	1
Stomach cancer	Female	Mean BMI	2	1
Stomach cancer	Female	Sanitation (proportion with access)	2	-1
Stomach cancer	Female	Improved Water Source (proportion with access)	2	-1
Stomach cancer	Female	Log-transformed SEV scalar: Stomach C	1	1
Stomach cancer	Female	Age- and sex-specific SEV for Unsafe water	2	1
Stomach cancer	Female	Age- and sex-specific SEV for Unsafe sanitation	2	1
Stomach cancer	Female	Socio-demographic Index	3	-1
Stomach cancer	Female	Healthcare Access and Quality Index	2	-1
Stomach cancer	Female	Diet high in sodium	1	1
Stomach cancer	Female	Age- and sex- specific SEV for low fruit	3	1
Stomach cancer	Female	Age- and sex- specific SEV for Low vegetables	3	1
Thyroid cancer	Male	Tobacco (cigarettes per capita)	2	1
Thyroid cancer	Male	Education (years per capita)	3	-1
Thyroid cancer	Male	LDI (I$ per capita)	3	1
Thyroid cancer	Male	Mean BMI	2	1
Thyroid cancer	Male	Sanitation (proportion with access)	3	-1
Thyroid cancer	Male	Improved Water Source (proportion with access)	3	-1
Thyroid cancer	Male	Log-transformed SEV scalar: Thyroid C	1	1
Thyroid cancer	Male	Socio-demographic Index	3	1
Thyroid cancer	Male	Healthcare Access and Quality Index	2	-1
Thyroid cancer	Male	Age- and sex- specific SEV for low fruit	3	1
Thyroid cancer	Male	Age- and sex- specific SEV for Low vegetables	2	1
Thyroid cancer	Male	Age- and sex-specific SEV for High red meat	2	1
Thyroid cancer	Male	Liters of alcohol consumed per capita	1	1
Thyroid cancer	Female	Tobacco (cigarettes per capita)	2	1

Thyroid cancer	Female	Education (years per capita)	3	-1
Thyroid cancer	Female	LDI (I$ per capita)	3	1
Thyroid cancer	Female	Mean BMI	2	1
Thyroid cancer	Female	Sanitation (proportion with access)	3	-1
Thyroid cancer	Female	Improved Water Source (proportion with access)	3	-1
Thyroid cancer	Female	Log-transformed SEV scalar: Thyroid C	1	1
Thyroid cancer	Female	Socio-demographic Index	3	1
Thyroid cancer	Female	Healthcare Access and Quality Index	2	-1
Thyroid cancer	Female	Age- and sex- specific SEV for low fruit	3	1
Thyroid cancer	Female	Age- and sex- specific SEV for Low vegetables	2	1
Thyroid cancer	Female	Age- and sex-specific SEV for High red meat	2	1
Thyroid cancer	Female	Liters of alcohol consumed per capita	1	1
Tracheal, bronchus, and lung cancer	Male	Cumulative cigarettes (10 years)	2	1
Tracheal, bronchus, and lung cancer	Male	Cumulative cigarettes (20 years)	2	1
Tracheal, bronchus, and lung cancer	Male	Diabetes Fasting Plasma Glucose (mmol/L), age-standardised 25+	2	1
Tracheal, bronchus, and lung cancer	Male	Education (years per capita)	3	-1
Tracheal, bronchus, and lung cancer	Male	LDI (I$ per capita)	3	1
Tracheal, bronchus, and lung cancer	Male	Indoor Air Pollution (All Cooking Fuels)	2	1
Tracheal, bronchus, and lung cancer	Male	Outdoor Air Pollution (PM2.5)	2	1
Tracheal, bronchus, and lung cancer	Male	Smoking Prevalence	1	1
Tracheal, bronchus, and lung cancer	Male	Log-transformed SEV scalar: Lung C	1	1
Tracheal, bronchus, and lung cancer	Male	Log-transformed age-standardised SEV scalar: Lung C	1	1
Tracheal, bronchus, and lung cancer	Male	Socio-demographic Index	3	1
Tracheal, bronchus, and lung cancer	Male	Healthcare Access and Quality Index	2	-1
Tracheal, bronchus, and lung cancer	Male	Residential radon	2	1
Tracheal, bronchus, and lung cancer	Male	Second-hand smoke	2	1
Tracheal, bronchus, and lung cancer	Male	Asbestos consumption (metric tons per year per capita)	1	1
Tracheal, bronchus, and lung cancer	Female	Cumulative cigarettes (10 years)	2	1
Tracheal, bronchus, and lung cancer	Female	Cumulative cigarettes (20 years)	2	1

Tracheal, bronchus, and lung cancer	Female	Diabetes Fasting Plasma Glucose (mmol/L), age-standardised 25+	2	1
Tracheal, bronchus, and lung cancer	Female	Education (years per capita)	3	-1
Tracheal, bronchus, and lung cancer	Female	LDI (I$ per capita)	3	1
Tracheal, bronchus, and lung cancer	Female	Indoor Air Pollution (All Cooking Fuels)	2	1
Tracheal, bronchus, and lung cancer	Female	Outdoor Air Pollution (PM2.5)	2	1
Tracheal, bronchus, and lung cancer	Female	Smoking Prevalence	1	1
Tracheal, bronchus, and lung cancer	Female	Log-transformed SEV scalar: Lung C	1	1
Tracheal, bronchus, and lung cancer	Female	Log-transformed age-standardised SEV scalar: Lung C	1	1
Tracheal, bronchus, and lung cancer	Female	Socio-demographic Index	3	1
Tracheal, bronchus, and lung cancer	Female	Healthcare Access and Quality Index	2	-1
Tracheal, bronchus, and lung cancer	Female	Residential radon	2	1
Tracheal, bronchus, and lung cancer	Female	Second-hand smoke	2	1
Tracheal, bronchus, and lung cancer	Female	Asbestos consumption (metric tons per year per capita)	1	1
Uterine cancer	Female	Tobacco (cigarettes per capita)	2	1
Uterine cancer	Female	Cumulative cigarettes (10 years)	2	1
Uterine cancer	Female	Cumulative Cigarettes (5 Years)	2	1
Uterine cancer	Female	Diabetes Age-Standardised Prevalence (proportion)	2	1
Uterine cancer	Female	Education (years per capita)	3	-1
Uterine cancer	Female	LDI (I$ per capita)	3	1
Uterine cancer	Female	Mean BMI	1	1
Uterine cancer	Female	Smoking Prevalence	2	1
Uterine cancer	Female	Total Fertility Rate	2	-1
Uterine cancer	Female	Log-transformed SEV scalar: Uterus C	1	1
Uterine cancer	Female	Socio-demographic Index	3	1
Uterine cancer	Female	Healthcare Access and Quality Index	2	-1
Uterine cancer	Female	Age- and sex- specific SEV for low fruit	2	1
Uterine cancer	Female	Age- and sex- specific SEV for Low vegetables	2	1

BMI = body-mass index
GBD = Global Burden of Disease Study;
HBsAg = Heptatitis B surface antigen;
HCV = Hepatitis C virus;
LDI = lag distributed income per capita (I$): gross domestic product per capita that has been smoothed over the preceding 10 years;
MET = metabolic equivalent of task;

PM2.5 = particulate matter ≤ 2.5 micrometres;
pufa = polyunsaturated fatty acid;
SEV = summary exposure value: for definitions and calculations, please see Section 2.6: "Step 5. Estimate summary exposure values" in the Supplementary Appendix 1 to "GBD 2019 Risk Factors Collaborators. Global burden of 87 risk factors in 204 countries and territories, 1990–2019: a systematic analysis for the Global Burden of Disease Study 2019. Lancet 2020; 396: 1223–49".[4] ; covariates with "C" following a cancer site name refer to a cancer site (eg, uterus C = uterus cancer) and were shortened due to space limitations in covariate names.

CoDCorrect

CODEm models estimate the individual cause-level mortality without taking into account the independently modeled all-cause mortality (#13 in Appendix figure 1). To ensure that all single causes add up to the all-cause mortality and that all child-causes add up to the parent cause, an algorithm called "CoDCorrect" is used (#14 and #15 in Appendix figure 1). Further details on the CoDCorrect algorithm can be found in Section 3.3.2 of the Supplementary Appendix 1 to the GBD 2019 paper "Global burden of 369 diseases and injuries in 204 countries and territories, 1990–2019: a systematic analysis for the Global Burden of Disease Study 2019".[3] Final mortality estimates at the 1000-draw level provide an estimated mean mortality with 95% uncertainty interval.

Calculating YLLs

To calculate years of life lost (YLLs), final death estimates after CoDCorrect adjustment are multiplied by the standard GBD life expectancy given the age at death, sex, and location. Further details on GBD life expectancy values can be found in the GBD 2019 paper "Global age-sex-specific fertility, mortality, health life expectancy (HALE), and population estimates in 2014 countries and territories, 1950-2019: a comprehensive demographic analysis for the Global Burden of Disease Study 2019".[22] Uncertainty is propagated from the CoDCorrect mortality estimates, calculating YLLs for each of the 1000 CoDCorrect draws to provide estimated mean YLLs with corresponding 95% uncertainty intervals.

Incidence estimation

The final GBD cancer mortality estimates (after CoDCorrect adjustment) were transformed to incidence estimates by using the MIRs specific to that cancer cause (#1 in Appendix figure 2). Final mortality estimates at the 1000-draw level were divided by the modeled MIR estimates (also at the 1000-draw level) to generate 1000 draws of incidence estimates (which provides an estimated mean incidence with 95% uncertainty interval). It was assumed that uncertainty in the MIRs is independent of uncertainty in the estimated mortality.

Prevalence estimation

After transforming the final GBD cancer mortality estimates to incidence estimates (step 1 in Appendix figure 2), incidence was combined with annual relative survival estimates from 1 to 10 years after diagnosis (step 7 in the Appendix figure 2). Previous reports suggest that the value of (1 – MIR) may serve as a proxy for 5-year relative survival, with the exact correlation varying slightly by cancer type.[23] Because this correlation varies, we trained cancer-specific prediction models to estimate 5-year survival from MIRs, using data from SEER.[6] We used SEER*Stat[24] to obtain mortality, incidence, and relative survival statistics from the nine SEER registries reporting from 1980-2014 (step 2), by cancer type, sex, 5-year blocks (ie, 1980-84, 1985-1989, etc.), and 5-year age groups (except combining 80+). For each cancer, we modelled SEER 5-year relative survival using MIRs calculated from SEER mortality and incidence. For GBD 2019 we updated this model from the Poisson regression used in GBD 2017[25] to using a generalised linear model with a quasibinomial family and logit link, weighted by the number of index cases (step 3 in Appendix figure 2). To reduce variability due to small samples, we only included MIRs based on at least 25 incident cases (except for the cancers mesothelioma, nasopharynx cancer, and acute lymphoid leukaemia, where MIRs based on at least 10 cases were included). These models were

then applied to the GBD MIR estimates to predict an estimated 5-year survival for each age/sex/year/location (step 4). To prevent unrealistic values, predicted 5-year survival values were Winsorised to be between 0% and 100% survival.

To generate yearly survival estimates up to 10 years, we downloaded SEER[6] sex- and age-specific annual 1- through 10-year relative survival data from persons diagnosed between 2001 and 2010 (2001 through 2010 so that all cases had at least 5 years of follow-up, with half having the full 10 years of follow-up). This is updated from GBD 2017, where we downloaded all-ages survival data from persons diagnosed in 2004 (2004 so that all cases had the full 10 years of follow-up).[26] A proportional scalar was calculated as the predicted GBD 5-year survival estimate divided by the SEER 5-year survival statistic, and was then used to generate yearly survival estimates by scaling the 1-10 year SEER curve to the GBD survival predictions under the proportional hazard assumption (step 5).

The estimated relative survival is next transformed into absolute survival estimates (steps 6 and 7 in Appendix figure 2). To account for background mortality in the relative survival estimates, GBD 2019 lifetables were used to calculate lambda (λ) values:[22]

$$\lambda = \frac{\ln\left(\frac{nLx_n}{nLx_{n+1}}\right)}{5}$$

nLx = person-years lived between ages x and x+n (from GBD lifetable).

Absolute survival was then calculated using an exponential survival function:

$$absolute\ survival = relative\ survival * e^{\lambda * t}$$

t = time (in years)

Absolute survival is combined with incidence to estimate the prevalence at each year 1 through 10 after diagnosis, which is then split into the four sequelae (step 8 in the Appendix figure 2). For the purposes of calculating disability due to cancer, survivors beyond 10 years were considered cured. For this group, the survivor population prevalence was divided into two sequelae: 1) diagnosis and primary therapy phase; and 2) controlled phase. For the population that did not survive beyond 10 years, the yearly prevalence was divided into the four sequelae by assigning the fixed durations for each of the (1) diagnosis and primary therapy phase, (2) metastatic phase, and (3) terminal phase, and assigning the remaining prevalence to the (4) controlled phase (step 8 in Appendix figure 2). Appendix Table 5 lists the durations of each, along with the sources used to determine their length.[27–32]

Appendix Table 5: Duration of four prevalence phases by cancer

GBD Cause*	Diagnosis / Treatment (months)*	Remission (months)	Disseminated/ metastatic (months)*	Note	Terminal (months)
Oesophageal cancer	5.0[27]	The remission phase duration is calculated based on the remaining time after attributing other sequelae durations.	4.6[28]	SEER Summary Stage 1997 (Distant site/node involved) 1995-2000	1
Stomach cancer	5.2[27]		3.9[28]	SEER Summary Stage 1997 (Distant site/node involved) 1995-2000	1

GBD Cause*	Diagnosis / Treatment (months)*	Remission (months)	Disseminated/ metastatic (months)*	Note	Terminal (months)
Liver cancer	4.0	The remission phase duration is calculated based on the remaining time after attributing other sequelae durations.	2.5[28]	SEER Summary Stage 1997 (Distant site/node involved) 1995-2000	1
Larynx cancer	5.3[27]		8.8[28]	SEER Stage IVc	1
Tracheal, bronchus, and lung cancer	3.3[29]		4.5[28]	SEER Summary Stage 1997 (Distant site/node involved) 1995-2000	1
Breast cancer	3.0[29]		17.7[28]	SEER Summary Stage 1997 (Distant site/node involved) 1995-2000	1
Cervical cancer	4.8[27]		9.2[28]	SEER Summary Stage 1997 (Distant site/node involved) 1995-2000	1
Uterine cancer	4.6[27]		11.6[28]	SEER Summary Stage 1997 (Distant site/node involved) 1995-2000	1
Prostate cancer	4.0[29]		30.4[28]	SEER Summary Stage 1997 (Distant site/node involved) 1995-2000	1
Colon and rectum cancer	4.0[29]		9.7[28]	SEER Summary Stage 1997 (Distant site/node involved) 1995-2000	1
Lip and oral cavity cancer	5.3[27]		9.3[28]	SEER Stage IVc	1
Nasopharynx cancer	5.3[27]		13.2[28]	SEER Stage IVc	1
Other pharynx cancer	5.3[27]		7.9[28]	SEER Stage IVc	1
Gallbladder and biliary tract cancer	4.0		3.5[28]	SEER Summary Stage 1997 (Distant site/node involved) 1995-2000	1
Pancreatic cancer	4.1[27]		2.5[28]	SEER Summary Stage 1977 (Distant site/node involved) 1995-2000	1
Ovarian cancer	3.2[29]		25.6[28]	SEER Summary Stage 1977 (Distant site/node involved) 1995-2000	1
Kidney cancer	5.3[27]		5.4[28]	SEER Summary Stage 1977 (Distant site/node involved) 1995-2000	1

GBD Cause*	Diagnosis / Treatment (months)*	Remission (months)	Disseminated/ metastatic (months)*	Note	Terminal (months)
Bladder cancer	5.1[27]	The remission phase duration is calculated based on the remaining time after attributing other sequelae durations.	5.8[28]	SEER Summary Stage 1977 (Distant site/node involved) 1995-2000	1
Thyroid cancer	3.0		19.4[28]	SEER Stage IVc	1
Mesothelioma	4.0		7.8[28]	SEER Summary Stage 1977 (Distant site/node involved) 1995-2000	1
Non-Hodgkin lymphoma	3.7[29]		7.7[31]		1
Multiple myeloma	7.0[27]		36.8[28]	SEER Median age standardised survival all patients, all years	1
Leukaemia[27]	5.0		43.7[28]	SEER Median age standardised survival all patients, all years	1

* Superscripts refer to references used to inform these values.

For cancer-specific procedure sequelae, hospital data were used to estimate the number of cancer patients undergoing mastectomy, laryngectomy, stoma, prostatectomy, and cystectomy (step 9 in Appendix figure 2). Proportions were generated by dividing the rate of procedures generated from the diagnostic codes in the hospital dataset and the coverage population by the GBD age-, and sex-specific disease incidence rates for that country.

To estimate procedure-related disability for each of these five cancers, the procedure proportions (proportion of each cancer population that undergo these procedures) from hospital data were used as input for a proportion model in DisMod-MR 2.1[4] to estimate the proportions for all locations, by age, year, and sex. Details of clinical and claims data processing are available in section 4.3.4 of the appendix to the GBD 2019 paper "Global burden of 369 diseases and injuries in 204 countries and territories, 1990–2019: a systematic analysis for the Global Burden of Disease Study 2019".[3]

Since colostomy or ileostomy procedures are done for reasons other than cancer, a literature review was conducted to determine the proportion of ostomies due to colon and rectum cancer. Based on the results of the literature review that an average of 58% of ostomies are done for Colon and rectum cancer, the "all cause" colostomy proportions were multiplied by 0.58.[33–35]

The final procedure proportions were applied to the incident cases of the respective cancers and multiplied with the proportion of the incident population surviving for 10 years to determine the incident cases of the cancer population that underwent procedures and that survived beyond 10 years. These incident cases were used again as an input for DisMod-MR 2.1, with a remission specification of zero and an excess mortality rate prior of 0 to 0.1, as well as with increasing both the age of the population and the year by 10 years to reflect prevalence after that population has survived 10 years. The results from this

model are incidence and lifetime prevalent cases of persons with these cancer-related sequelae who have survived beyond 10 years.

Since disability associated with prostatectomy comes from impotence and incontinence, and not from the prostatectomy itself, 18% of the prostatectomy prevalence was assumed to have incontinence and 55% was assumed to have impotence, based on a literature review done for GBD 2013.[36–43] Cases were assigned disability for either impotence or incontinence, but no cases were assigned disability from both.

We assumed that for the population surviving up to 10 years, only the prevalence population being in remission experiences additional disability due to procedures (eg, women suffering from metastatic breast cancer do not experience additional disability due to a mastectomy during this phase). To estimate the prevalence of the cancer population in remission during the first 10 years after diagnosis with and without procedure-related disability, we multiplied the prevalence of the population in the remission phase with the proportion of the population undergoing a procedure. This step allowed us to estimate disability during the remission phase for both the population experiencing disability due to the remission phase alone, as well as the population experiencing disability from the remission phase and the additional procedure-related disability.

Lastly, the procedure sequelae prevalence and general sequelae prevalence were multiplied with their respective disability weights (Appendix table 6) to obtain the number of YLDs (steps 11 and 12 Appendix figure 2). A description of non-procedure disability weights calculations can be found in "Section 4.8: Disability weights" in the Supplementary appendix 1 to "Global burden of 369 diseases and injuries in 204 countries and territories, 1990–2019: a systematic analysis for the Global Burden of Disease Study 2019".[3] In brief, disability weights are created from survey data to represent the magnitude of health loss associated with an outcome. These disability weights range from 0, implying a state equivalent to full health, to 1, a state equivalent to death. The sum of these YLDs is the final YLD estimate associated with each cancer cause.

Appendix Table 6: Lay description of cancer states and corresponding disability weights

**Health state**	**Lay description**	**Disability weight (95% uncertainty interval)**
Cancer, diagnosis and primary therapy<br>*All cancers*	This person has pain, nausea, fatigue, weight loss and high anxiety.	0.288<br>(0.193 to 0.399)
Cancer, controlled phase<br>*All cancers*	This person has a chronic disease that requires medication every day and causes some worry but minimal interference with daily activities.	0.049<br>(0.031 to 0.072)
Cancer, metastatic<br>*All cancers*	This person has severe pain, extreme fatigue, weight loss and high anxiety.	0.451<br>(0.307 to 0.600)
Terminal phase, with medication<br>*All cancers*	This person has lost a lot of weight and regularly uses strong medication to avoid constant pain. The person has no appetite, feels nauseous, and needs to spend most of the day in bed.	0.540<br>(0.377 to 0.687)

Mastectomy<br>*Breast cancer*	This person had one of her breasts removed and sometimes has pain or swelling in the arms.	0.036<br>(0.020 to 0.057)
Stoma<br>*Colon and rectum cancer*	This person has a pouch attached to an opening in the belly to collect and empty stools.	0.095<br>(0.063 to 0.131)
Laryngectomy<br>*Larynx cancer*	This person has difficulty speaking, and others find it difficult to understand.	0.051<br>(0.032 to 0.078)
Urinary incontinence<br>*Bladder cancer; Prostate cancer*	This person cannot control urinating.	0.139<br>(0.094 to 0.198)
Impotence<br>*Prostate cancer*	This person has difficulty in obtaining or maintaining an erection.	0.017<br>(0.009 to 0.030)

Calculating DALYs

To estimate DALYs for GBD 2019, we started by estimating cause-specific mortality and non-fatal health loss. For each year for which YLDs have been estimated, we computed DALYs by adding YLLs and YLDs for each age-sex-location. Uncertainty in YLLs was assumed to be independent of uncertainty in YLDs. We calculated 1000 draws for DALYs by summing the first draw of the 1000 draws for YLLs and YLDs and then repeating for each subsequent draw. 95% UIs were computed by using the 25th and 975th ordered draw of the DALY uncertainty distribution. We calculated DALYs as the sum of YLLs and YLDs for each cause, location, age group, sex, and year.

Calculating Proportional Burden

Proportional burden was calculated by taking the proportion of the 1000 draws (numerator draws/denominator draws) to get the proportion draws, then taking the mean and 95% UIs of the 1000 draws to get the proportion mean and 95% UIs. For example, for calculating proportional risk-attributable DALY burden, the numerator draws would be the risk-attributable DALY burden for an individual cancer for an individual risk for a specific age group, year, sex, location, while the denominator draws would be the risk-attributable DALY burden for total cancers for an individual risk for a specific age group, year, sex, location.

Reporting Standards

All rates are reported per 100 000 person-years. Annualised rates of change (ARC) from 2010 to 2019 represent the average percentage change per year over this period, and are calculated as:

$$ARC = \frac{\ln\left(\frac{X\_y2}{X\_y1}\right)}{y2 - y1}$$

X_y_n = value of measure (e.g. deaths) at year $\_y_n$
$y1$ = starting year (e.g. 2010)
$y2$ = ending year (e.g. 2019)

The GBD world population age standard was used to calculate age-standardised rates presented throughout GBD. In GBD 2019, we used the non-weighted mean of the GBD year's age-specific proportional distributions for national locations with populations greater than 5 million in the GBD year to update the world population age standard.[22] The final values used for the age standard are specified in Appendix table 13 of the GBD 2019 paper "Global age-sex-specific fertility, mortality, health life expectancy (HALE), and population estimates in 204 countries and territories, 1950-2019: a comprehensive demographic analysis for the Global Burden of Disease Study 2019".[22]

Socio-demographic Index (SDI) Definition and Calculation

The Socio-demographic Index (SDI) is a summary indicator to represent background levels of social and economic conditions that can influence health outcomes in a given location. This summary indicator comprises three indices: lag-distributed income per capita, mean education for those aged 15 years or older, and total fertility rate for those younger than 25 years of age. Possible values for each of these three indices range from 0 to 1, representing the bounds with which lower or higher values of the level of development for that index would no longer worsen or improve health outcomes, respectively. The composite SDI is the geometric mean of these three indices for a given location-year. For reporting purposes, values were multiplied by 100 to obtain SDI on a scale of 0 to 100. The SDI cutoffs for determining SDI quintiles for analysis were computed by using the country-level estimates of SDI for the year 2019, excluding countries with populations less than 1 million. For GBD 2019 analyses, all locations are assigned to these quintiles according to their SDI value in the year 2019. See Section 6 in Supplementary Appendix 1 to the GBD 2019 Diseases & Injuries capstone[3] for more details regarding SDI estimation, and page 147 of this appendix for the SDI quintile estimate for each country in GBD 2019.

Uncertainty Estimation

Uncertainty in cancer estimates begins with the availability of and variability in cancer cause-specific data by age, sex, location and year. The uncertainty in cancer mortality estimates arises from CODEm and CoDCorrect. For more information see the CODEm methodology paper by Foreman et al., and Supplementary Appendix 1 to "Global burden of 369 diseases and injuries in 204 countries and territories, 1990–2019: a systematic analysis for the global burden of disease study 2019".[3,21] Uncertainty in cancer incidence estimates results from both the uncertainty in mortality estimates as well as the uncertainty in the MIR estimates, which result from the ST-GPR models. Uncertainty from the mortality estimates and the MIRs were assumed to be independent. Cancer prevalence uncertainty results from both the incidence uncertainty as well as the uncertainty from survival estimates. These were assumed to be independent. Uncertainty in cancer YLD estimation results from the uncertainty in the prevalence of each cancer sequela and uncertainty in the disability weight and is propagated into the final comorbidity-corrected YLD result. The uncertainty in prevalence and the uncertainty in disability weights are assumed to have no correlation. Cancer YLL uncertainty results from uncertainty in mortality estimates as well as uncertainty in life expectancy estimates. Uncertainty in cancer DALY estimates results from the uncertainty in YLLs and the uncertainty in YLDs, which were assumed to be independent. The same technique for propagating uncertainty elsewhere in the GBD study is applied in the cancer estimation process. In brief, the distribution of each step in the computation process is stored in 1000 draws. The distributions are determined from the data input sampling error, the uncertainty of the model coefficients, and the uncertainty of severity distributions and disability weights. The 1000 draws are used for every step in the process, with final estimates computed using the mean estimate across 1000 draws. The 95% uncertainty intervals are determined by the 25th and 975th ranked values across all 1000 draws.[3] More specific information regarding uncertainty intervals can be found in the GBD 2019 capstone papers.[3,4,22]

Limitations

There are certain limitations to consider when interpreting the GBD mortality cancer estimates. First, even though every effort is made to include the most recently available data for each country, data seeking resources are not limitless and new data cannot always be accessed as soon as they are made available. It is therefore possible that the GBD study does not include all available data sources for cancer incidence or cancer mortality. Second, different redistribution methods can potentially change the cancer estimates substantially if the data sources used for the estimated location contain a large number of undefined causes; however, neglecting to account for these undefined deaths would likely introduce an even greater bias in the disease estimates. Third, using mortality-to-incidence ratios to transform cancer registry incidence data to mortality estimates requires accurate MIRs. For GBD 2019 we have made further refinements to the estimation of MIRs, but the method remains sensitive to under-diagnosis of cancer cases or under-ascertainment of cancer deaths. However, given that the majority of data used for the cancer mortality estimation come from vital registration data and not cancer registry data, this is not a major limitation. Finally, no estimates are available for some locations, such as Western Sahara and French Guiana, as they were not modelled locations in the Global Burden of Diseases, Injuries, and Risk Factors Study 2019. These countries are shaded white in the global map figures included in this paper.

GBD Risk factor estimation[4]

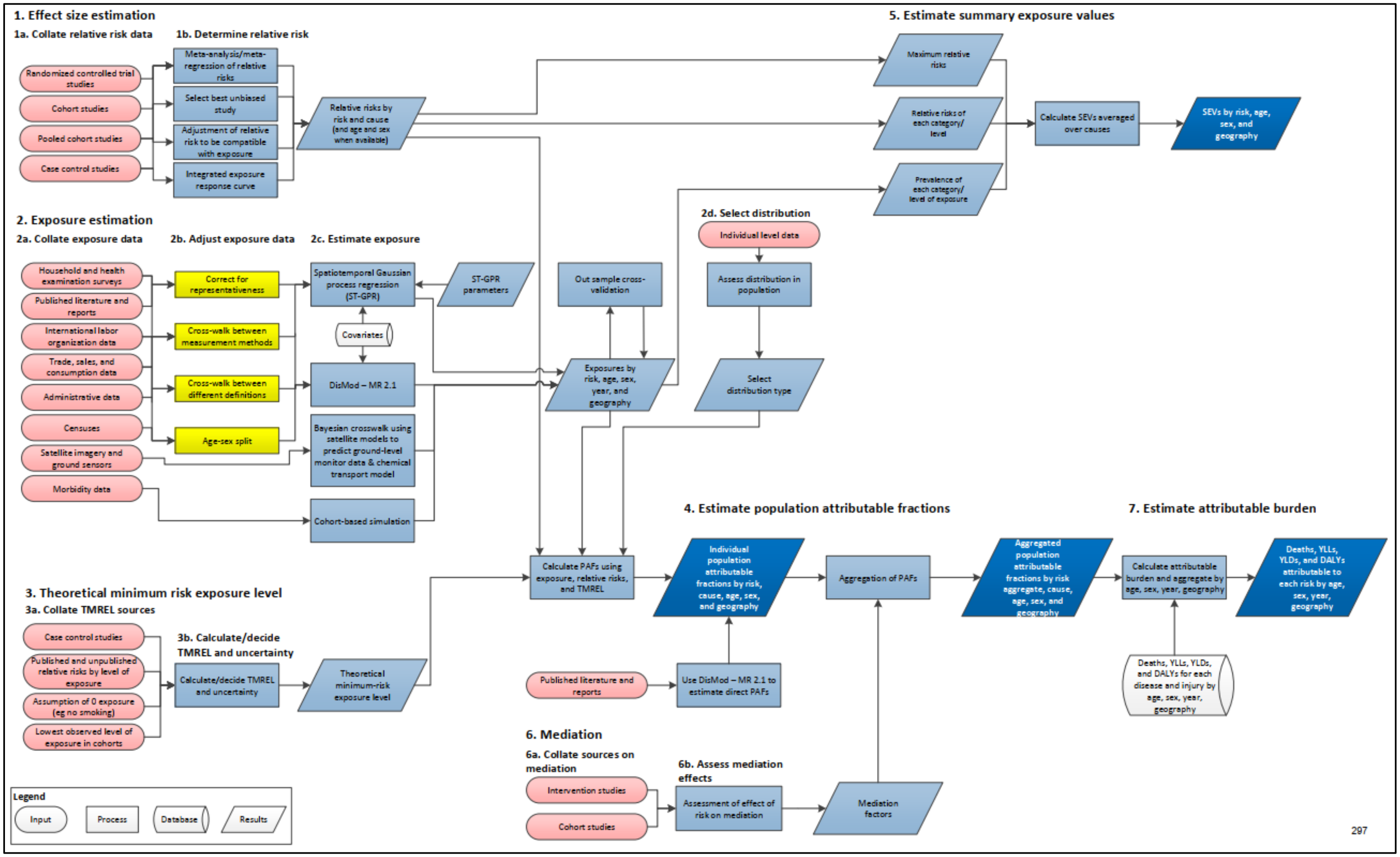

Appendix Figure 3: Analytical flowchart of the comparative risk assessment for the estimation of population attributable fractions by geography, age, sex, and year for GBD 2019. GBD = Global Burden of Disease, SEV = Summary exposure values, TMREL = Theoretical minimum-risk exposure level, PAFs = Population attributable fraction, YLL = Years of life lost, YLD = Years lived with disability, DALYs = Disability-adjusted life-years.

GBD risk factor hierarchy

The GBD 2019 risk factors hierarchy and levels are summarised in Appendix table 9. The risk hierarchy is based on common features of individual risks; for example, risk factors that represent behavioural factors are grouped together. Modeling risk factors in GBD 2019 often requires disease context across many different communicable and non-communicable diseases; although cancers specifically are the focus of this analysis, the following risk factors methods section will often reference GBD causes (diseases and injuries) that are outside of the scope of this paper. This broader disease context is included for completeness and accuracy, however the results (both in the manuscript and in this appendix) focus only on the 34 risk factor groups (at the most detailed level) that are currently estimated in GBD as contributing burden to various cancer groups. The cancer risk-outcome pairs included in the analysis for this paper are summarised in Appendix table 10, and definitions and the theoretical minimum risk exposure level (TMREL) are described in Appendix table 11.

Risk factors data input sources overview

As with the input data described above to inform mortality and morbidity estimation for causes of death and disability, GBD 2019 also incorporated a large number and wide variety of input sources for risk factors for 204 countries and territories from 1990-2019. These input sources are accessible through the interactive citation tool available in the GHDx at http://ghdx.healthdata.org/gbd-2019/data-inputsources.

Overview of risk factor estimation

The comparative risk assessment (CRA) conceptual framework was developed by Murray and Lopez,[44] who established a causal web of hierarchically organised risks or causes that contribute to health outcomes, which allows for quantification of risks or causes at any level in the framework. The GBD 2019 study evaluated a set of behavioural, environmental and occupational, and metabolic risks, in which risk-outcome pairs were included based on evidence rules. These risks were organised in four hierarchical Levels, where Level 1 represents the overarching categories (behavioural, environmental and occupational, and metabolic) nested within Level 1 risks; Level 2 contains both single risks and risk clusters (such as drug use and occupational risks); Level 3 contains the disaggregated single risks from within Level 2 risk clusters (such as occupational carcinogens); and Level 4 details risks with the most granular disaggregation (such as occupational exposure to arsenic). At each level, risk combinations were evaluated in order to determine additive, multiplicative, or shared common pathways for intervention. This approach allows the quantification of the proportion of risk-attributable burden shared with another risk or combination of risks and the measurement of potential overlaps between behavioural, environmental and occupational, and metabolic risks.

Two types of risk assessments are possible within the CRA framework: attributable burden and avoidable burden. Attributable burden is the reduction in current disease burden that would have been possible if past population exposure had shifted to an alternative or counterfactual distribution of risk exposure. Avoidable burden is the potential reduction in future disease burden that could be achieved by changing the current distribution of exposure to a counterfactual distribution of exposure. Murray and Lopez identified four types of counterfactual exposure distributions: (1) theoretical minimum risk; (2) plausible minimum risk; (3) feasible minimum risk; and (4) cost-effective minimum risk.[45] The TMREL is the level of risk exposure that minimises risk at the population level or the level of risk that captures the maximum attributable burden. Other possible forms of risk quantification include plausible minimum risk – which reflects the distribution of risk that is conceivably possible and would minimise population-level risk if achieved – whereas feasible minimum risk describes the lowest risk distribution that has been attained within a population and cost-effective minimum risk is the lowest risk distribution for a population that can be attained in a cost-effective manner. Because no robust set of forecasts for all components of GBD is available, in this study we focus on quantifying attributable burden by using the theoretical minimum risk counterfactual distribution. Given the focus in this study on attributable burden, risk reversibility is

not a criterion used in estimation here. In general, this analysis follows the CRA methods used since GBD 2015.[46] The risk factor methods from pg. 57–145 in this appendix methods described here have been copied and summarised from the appendix to “Global burden of 87 risk factors in 204 countries and territories, 1990–2019: a systematic analysis for the Global Burden of Disease Study 2019”.[4]

Step 1: Effect size estimation

Estimating the effect size for each risk factor took place in two steps: (1) Collation of relative risk data, and (2) Estimation of overall relative risk.

Criteria for inclusion of risk-outcome pairs

Since GBD 2010 we have included risk-outcome pairs that we have assessed as meeting the World Cancer Research Fund (WCRF) grades of convincing or probable evidence.[47] In this framework, convincing evidence consists of biologically plausible associations between exposure and disease established from multiple epidemiological studies in different populations. Evidentiary studies must be substantial, include prospective observational studies, and, where relevant, randomised controlled trials (RCTs) of sufficient size, duration, and quality that show consistent effects. Probable evidence is similarly based on epidemiological studies with consistent associations between exposure and disease but for which shortcomings in the evidence exist, such as insufficient available trials (or prospective observational studies).

The World Cancer Research Fund (WCRF) grading system was used to assign evidence as convincing, probable, possible, or insufficient.[47]

Estimation of overall relative risk

The relative risk (RR) by level of exposure or by cause for mortality or morbidity can be found in published and unpublished primary studies or in secondary studies that summarise RRs. In Step 1a of the analytical process (Appendix figure 3), we collated information from RCTs, cohort, pooled cohort, and case-control studies, and in Step 1b, used these data to determine the RR for the risk-outcome pairs included in GBD 2019 (table S7 on pg. 333 in the appendix to “Global burden of 87 risk factors in 204 countries and territories, 1990–2019: a systematic analysis for the Global Burden of Disease Study 2019)”.[4] For most risks, data from pooled cohorts or meta-analyses of cohorts were used. GBD 2019 estimated RRs of mortality and morbidity for 67 risk factors for which attributable burden was determined by using RR and exposure; for this cancer-specific analysis, we focused on 34 of these risk factors. We incorporated RRs from studies that controlled for confounding but not for factors along the causal pathway between exposure and outcome. For risk-outcome pairs with evidence available for only one element of mortality or morbidity, we generally assumed that the estimated RRs applied equally to both. Given evidence of statistically different RRs for mortality and morbidity, we incorporated different RRs for each. We did not find that RRs were consistently higher or lower for mortality compared with morbidity. Details and citation information for the data sources used for RRs are provided in searchable form through a web tool (http://ghdx.healthdata.org/). Available data sources for determining RRs varied across risks.

For the following risks estimated from a continuous exposure distribution in which the effect size was reported by categories in pooled or meta-analysis studies, we converted those categories to RR per unit increase in exposure and assumed a linear increase in the log of the RR and exposure: radon, high fasting plasma glucose, and high body-mass index. Many meta-analyses convert RRs to per unit increase for convenience, particularly when studies choose different categories that could not otherwise be compared. If samples in the primary studies at high levels of exposure were sufficient to inform the shape of the tail

of the distribution, we applied a cap to the maximum RR by using the midpoint of the last category for which an RR was reported.

In GBD 2019, for a selected set of continuous risk factors, we modelled RRs using meta-regression—Bayesian, regularised, trimmed (MR-BRT), relaxing the log-linear assumption to allow for monotonically increasing or decreasing but non-linear functions using cubic splines. Risk factors for which we undertook this re-analysis include: all dietary risk factors, low physical activity, and air pollution. Because knot placement can affect the shape of the risk function when modelling with a cubic spline, we generated a wide range of knot placements and created an ensemble across these different knot placements. We also included in the final estimation 10% trimming of the data to avoid the results being sensitive to outliers.

Specific modelling approaches for relative risk are available on pg. 18–25 in the appendix to "Global burden of 87 risk factors in 204 countries and territories, 1990–2019: a systematic analysis for the Global Burden of Disease Study 2019".[4]

Step 2: Exposure estimation

Estimating the exposure level for different risk factors took place in three steps: (1) collate exposure data, (2) adjust exposure data, and (3) estimate exposure.

Collate exposure

For GBD 2019, we conducted systematic literature reviews for a subset of the risk factors in the GBD risk factory hierarchy list. For other risk factors, only a small fraction of the existing data appears in the published literature, and other sources predominate, such as survey data and satellite data. Data were systematically screened from household surveys archived in the GHDx (http://ghdx.healthdata.org), including Demographic and Health Surveys, Multiple Indicator Cluster Surveys, Living Standards Measurement Surveys, and Reproductive Health Surveys. Other national health surveys were identified based on survey series that had yielded usable data for past rounds of GBD, sources suggested to us by in-country collaborators, and surveys identified in major multinational survey data catalogues, such as the International Household Survey Network and the WHO Central Data Catalog, as well as through country Ministry of Health and Central Statistical Office websites. Citations for all data sources used for risk factor estimation in GBD 2019 are provided in searchable form through a web-tool (http://ghdx.healthdata.org). A description of the search terms employed for risk-specific systematic reviews are detailed by cause in appendix section 4 of "Global burden of 87 risk factors in 204 countries and territories, 1990–2019: a systematic analysis for the Global Burden of Disease Study 2019".[4]

Information on systematic reviews were managed by using Research Electronic Data Capture (REDCap) electronic data capture tools hosted at the University of Washington.[48] REDCap is a secure, web-based application designed to support data capture for research studies that provides 1) an intuitive interface for validated data entry; 2) audit trails for tracking data manipulation and export procedures; 3) automated export procedures for seamless data downloads to common statistical packages; and 4) procedures for importing data from external sources.

Search terms

Search terms for updates of systematic reviews for GBD 2019 are shown by risk factor in appendix "Section 4: Risk-specific modelling descriptions" in the GBD 2019 paper "Global burden of 87 risk factors in 204 countries and territories, 1990–2019: a systematic analysis for the Global Burden of Disease Study 2019".[4]

Survey data preparation
Survey data constitutes a substantial part of the underlying data used in the estimation process. During extraction, we concentrated on demographic variables (such as location, gender, age), survey design variables (such as sampling strategy and sampling weights), and the variables used to define the population estimate (such a prevalence or a proportion) and a measure of uncertainty (standard error, confidence interval or sample size and number of cases).

Adjust exposure data
Several adjustments were applied to extracted exposure sources to make the data more consistent and suitable for modelling. In GBD 2019, we implemented adjustments of risk exposure data to deal with alternative case definitions or study methods prior to entering data into our main analytical tools of DisMod-MR 2.1 and ST-GPR.[4] This decision also included the adjustment of data presented for both sexes to a male and female equivalent. The starting point was to explicitly state the reference case definition and study method and identify alternative definitions and study characteristics that fall within our inclusion criteria.

We compiled data from both within-study comparisons (ie, data that used alternative and reference definitions in the same population) and between-study comparisons (ie, data that used an alternative definition in one population and a reference definition in another population that overlap in location, time, age, and sex) of different case definitions. For between-study comparisons, we allowed a maximum calendar year difference between studies of five years. Where validation studies (ie, those carried out at the introduction of a new set of diagnostic criteria comparing to previous criteria) were available, we extracted data on the comparison of alternative to reference. For quantities of interest with multiple alternative definitions/methods we also look for pairs comparing two alternatives. In a network analysis, if A is the reference and B and C are two alternatives, a comparison of A vs B and B vs C provides an indirect comparison of the alternative C against the reference A.

We pooled either the logit difference between alternative and reference or the natural log of the ratio of alternative to reference. From simulations we found that the two methods provide almost identical results for quantities that after adjustment do not exceed a value of 0.5 (eg, prevalence or proportion). The logit difference method much better dealt with higher values and avoided prevalence or proportions to exceed one. If the values of either the reference or alternative were zero, we aggregated values across age groups until both values had non-zero observations. We used the delta method to compute the standard error of the reference and alternative measures in logit space. The standard error of the logit difference was computed as the square root of the sum of the variances of each data point in a pair.

Data analysis
We used a network random effects meta-regression in MR-BRT to predict adjustments based on the statistical model, including uncertainty in the adjustment and sampling error of each data point. Further detail on this methodology can be found in "Section 2.2.2: Determine relative risks" in the Supplementary appendix to "Global burden of 87 risk factors in 204 countries and territories, 1990–2019: a systematic analysis for the Global Burden of Disease Study 2019".[4]

Mean exposure estimation
In Step 2a of the estimation process, we used systematic literature reviews to identify risk factor exposure studies published or identified since GBD 2017 and combined these with existing data from household and health examination surveys and census, morbidity, or satellite imagery and ground sensor data (used for estimation of particulate matter <2.5 μm in diameter [PM2.5]). Certain risks, such as poor diet and

excessive alcohol consumption, also incorporated administrative record systems. Data sources used in estimating risk factor exposure can be accessed through the data source tool at http://ghdx.healthdata.org/.

Once data were collected and compiled, Step 2b of the analytical flowchart describes the adjustments applied, where necessary, to correct for bias. Examples of these adjustments include use of urban studies for lead; crosswalks between different measurements, methods, and definitions, such as for self-report of obesity and glycated haemoglobin (HbA1C) for diabetes; and age-sex splitting of data, such as for fasting plasma glucose (FPG) level that may be reported from broad age-groups.

For the GBD, we developed two modelling approaches, a Bayesian meta-regression model (DisMod-MR 2.1) and a spatiotemporal Gaussian process regression model (ST-GPR), to pool data from different sources, control and adjust for bias in data, and incorporate other types of information such as country-level covariates. DisMod-MR 2.1 and ST-GPR are mixed effect models that borrow information across age, time, and locations to synthesise multiple data sources into unified estimates of levels and trends. A detailed description of the likelihood used for estimation and a full description of improvements made for DisMod-MR 2.1 were detailed by Vos and colleagues,[49] who provided additional detail in the appendix to that paper.[50] The ST-GPR model has three main hyperparameters that control for smoothing across time, age, and location. Values for these hyperparameters were selected on the basis of cross-validation. Cross-validation tests were conducted for different combinations of the hyper-parameters for three types of models: one data-sparse model, one data-moderate model, and one data-dense model. In each test, 20% of the data were held out, and the performance of each combination of hyperparameters was evaluated on the held-out data. For each hyperparameter combination, 10 cross-validation tests were conducted. The performance of each model in predicting the withheld 20% of the data was evaluated by using a combined measure based on root mean square error (RMSE) and uncertainty interval (UI) coverage. A detailed description of the ST-GPR process regression can be found in "Section 2.3.3: Step 2c: Estimate exposure" in the Supplementary appendix to "Global burden of 87 risk factors in 204 countries and territories, 1990–2019: a systematic analysis for the Global Burden of Disease Study 2019".[4]

The main difference between these methods is their power to include unstructured types of data by sex and age group and their degree of flexibility. DisMod-MR 2.1 is used for 6 risk factors for which data were available by different age intervals or mixed sex groups; DisMod-MR 2.1 is the preferred tool in these cases because of its ability to integrate over age and adjust for different exposure definitions in the data; however, the use of Bayesian Markov Chain Monte Carlo (MCMC) simulations with large volumes of data renders the analysis computationally intensive and reduces the number of iterations that are possible. If standard age-group data are available – as is generally the case for metabolic risks – using ST-GPR becomes the preferred approach.

In some cases, we adapted our methods of modelling exposure to risks where necessary to account for complexities in the risk-outcome relationship or the need for particular handling of data, for example, dietary risks and ambient air pollution (see section "GBD risk-specific methods summaries" in this appendix for more detail). A complete list of risks included in this analysis is reported in Appendix table 9.

Step 3: TMREL

In this and all previous GBD studies, the counterfactual level of risk exposure used is the risk exposure that is both theoretically possible and minimises risk in the exposed population that consequently captures the maximum population-attributable burden.[45] For each risk evaluated in GBD 2019, Step 4 of the analytical flowchart describes the use of the best available epidemiological evidence from published and unpublished RRs by level of exposure and the lowest observed level of exposure from cohorts, used to select a single level of risk exposure that minimises risk from all causes of deaths combined to establish

the TMREL. In principle, the TMREL for a given risk may vary by age, sex, and location if supported by clear evidence. Based on the available evidence, the TMREL itself can be uncertain.

In GBD 2019, we updated the process of estimating TMREL for dietary risks. We set the TMREL to zero for all harmful dietary risk factors with monotonically increasing risk functions (eg, processed meat intake); this excludes sodium. For protective risks with monotonically declining risk functions with exposure (eg, fruit intake), we first determined the 85th percentile of exposure in the cohorts or trials used in the meta-regression of each outcome that was associated with the risk. Then, we determined the TMREL by weighting each risk-outcome pair by the relative global magnitude of each outcome.

Step 4: Estimated population-attributable fractions

Risks are categorised on the basis of how exposure was measured: dichotomous, polytomous, and continuous. The PAF, which represents the proportion of risk that would be reduced in a given year if the exposure to a risk factor in the past were reduced to an ideal exposure scenario, is defined for a continuous risk factor as:[51]

$$PAF_{joasgt} = \frac{\int_{x=l}^{u} RR_{joasg}(x)P_{jasgt}(x)dx - RR_{joasg}(TMREL_{jas})}{\int_{x=l}^{u} RR_{joasg}(x)P_{jasgt}(x)dx}$$

where PAF_{joasgt} is the PAF for cause o due to risk factor j for age group a, sex s, location g, and year t. $RR_{joasg}(x)$ is the RR as a function of exposure level x for risk factor j for cause o, age group a, sex s, and location g with the lowest level of observed exposure as l and the highest as u; $P_{jasgt}(x)$ is the distribution of exposure at x for age group a, sex s, location g, and year t; and $TMREL_{jas}$ is the TMREL for risk factor j, age group a, and sex s.

The PAF_{joasgt} for dichotomous and polytomous risk factors for every country is defined as:

$$PAF_{joasgt} = \frac{\sum_{x=1}^{u} RR_{joasg}(x)P_{jasgt}(x) - RR_{joasg}\left(TMREL_{jas}\right)}{\sum_{x=1}^{u} RR_{joas}(x)P_{jasgt}(x)}$$

where PAF_{joasgt} is the PAF for cause o due to risk factor j for age group a, sex s, location g, and year t. (x) is the RR as a function of exposure level x for risk factor j for cause o, age group a, sex s, and location g on a plausible range of exposure levels from l to u; $P_{ja}(x)$ is the proportion of the population in risk group (prevalence) for age group a, sex s, location g, and year t; and $TMREL_{jas}$ is the TMREL for risk factor j, age group a, and sex s.

Step 5: Estimate summary exposure values

Summary exposure value (SEV) is the RR-weighted prevalence of exposure, a univariate measure of risk weighted exposure, taking the value zero when no excess risk for a population exists and the value one when the population is at the highest level of risk. We report SEVs on a scale from 0% to 100% on which a decline in SEV indicates reduced exposure to a given risk factor and an increase in SEV indicates increased exposure.

We first calculate risk, r, and cause, c, for specific SEVs by using the following equation,

$$SEV_{rc} = \frac{\frac{PAF_{rc}}{1 - PAF_{rc}}}{RR_{max} - 1}$$

for each most-detailed age, sex, location, year, and outcome. PAF is the YLL (expect for occupational noise, bullying victimisation, and occupational ergonomic factors, which are YLD only and thus use the YLD) PAF. RR_{max} for categorical risks is the RR at the highest category of exposure. For continuous risks, this is

$$RR_{max} = RR^{\frac{TMREL - 1^{st} exposure}{RR_{scalar}}}$$

if protective, or

$$= RR^{\frac{99^{th} exposure - TMREL}{RR_{scalar}}}$$

otherwise, and for custom modelled risks like ambient particulate matter pollution, HAP from solid fuels, alcohol, smoking, and physical activity, the modeller provides draws of RR_{max}. Generally, RRs do not vary across time and space. However, exceptions exist, such as risks from secondhand smoke (SHS) or HAP for which the RR is based on the integrated exposure response (IER) curve. In these cases, the RR is averaged across location and year to ensure no time or space variation. If the PAF is negative, which signifies a protective effect for that outcome, the PAF is set to 0 and the SEV is then also 0 because the SEV is univariate and constrained to be a value between 0 and 1. Once we obtained a set of risk-cause specific SEVs at the most-detailed risk, cause, age, sex, and location for all years, we averaged across causes to produce the final risk specific SEV_r,

$$SEV_r = \frac{1}{N(c)} \sum_c SEV_{rc}$$

where $N(c)$ is the total number of outcomes for a risk.

Step 6: Mediation

The portion of the burden of disease that is attributable to various combinations of risk factors or to all risk factors combined has been a topic of broad interest.[52] Since GBD 2013, we aggregated all risk factors into three large categories—behavioural, environmental and occupational, and metabolic risks—and aggregated all GBD risk factors into a single attributable fraction for each disease and eventually for all causes of burden.

Aggregating risk factors at different levels shares three essential challenges:

1. Risk factor coexistence or aggregation: for example, metabolic risk factors often occur together, or high-risk behaviours such as drug abuse and unsafe sex are related.
2. Mediation: a risk factor may affect another risk factor that lies in the physiological pathway to a disease outcome. It can be inside a cluster of risk factors, such as the effect of obesity through an

increase in FPG level and later cardiovascular disease (CVD) outcomes, or between clusters of risk factors, such as the effect of fibre on cholesterol.
3. The formula used to calculate the aggregated PAF.

The aggregation method is conceptually applicable to other aggregations such as socioeconomic factors, education, homelessness, and refugee status that are being considered for inclusion in future GBD iterations. In the next section, we explain our approach to dealing with these challenges.

There are three patterns of associations between risk factors to consider. The first concerns confounding; risk B affects risk A and outcome C (Pattern 1 in *Patterns of associations between risk factors* on pg. 64). In these cases, the RR for A should be adjusted for B; for example, the fruit RR is adjusted for smoking. If part of the effect of A is through B, a mediator, we do not adjust the effect of A for B. For example, we do not adjust the RR of body-mass index (BMI) for cholesterol because cholesterol lies in the biological pathway between BMI and cardiovascular outcomes (Pattern 2 in in *Patterns of associations between risk factors* on pg. 65). The third pattern occurs when risks A and B are proxies of a third variable Z and aggregation aims to estimate the total effect of a latent variable Z on C. An example is child growth failure, which is measured by stunting, wasting, and underweight as proxies.

Patterns of association between risk factors.

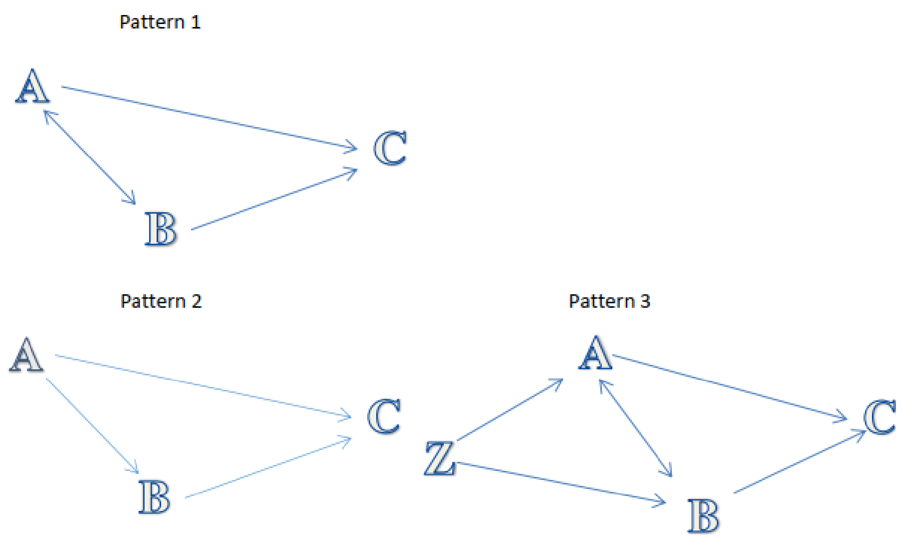

Further details on the mediation analysis can be found on pg. 42–46 in the appendix to "Global burden of 87 risk factors in 204 countries and territories, 1990–2019: a systematic analysis for the Global Burden of Disease Study 2019".[4]

Step 7: Estimate attributable burden

Four key components are included in the estimation of the burden attributable to a given risk factor: the metric of burden being assessed (the number of deaths, YLLs, YLDs, or DALYs [the sum of YLLs and YLDs]); the exposure levels for a risk factor; the RR of a given outcome due to exposure; and the counterfactual level of risk factor exposure. Estimates of attributable burden as DALYs for risk-outcome pairs were generated by using the following model:

$$AB_{jasgt} = \sum_{o=1}^{w} DALY_{joasgt}\, PAF_{joasgt}$$

where AB_{jasgt} is the attributable burden for risk factor j for age group a, sex s, location g, and year t; $DALY_{joasgt}$ is total DALYs for cause o (of w relevant outcomes for risk factor j) for age group a, sex s, location g, and year t; and PAF_{joasgt} is the PAF for cause o due to risk factor j for age group a, sex s, location g, and year t. The proportions of deaths, YLLs, or YLDs attributable to a given risk factor or risk factor cluster were analogously computed by sequentially substituting each metric in place of DALYs in the equation provided.

Uncertainty in Risk Factor Estimation

Uncertainty is generated and propagated throughout several stages of the GBD risk factors modeling pipelines. For full descriptions of these processes, please refer to the Supplementary appendix to the GBD 2019 Risk Factors capstone paper, "Global burden of 87 risk factors in 204 countries and territories, 1990–2019: a systematic analysis for the Global Burden of Disease Study 2019".[4] For uncertainty estimation methods unique to each risk factor in the GBD study, please see the section of this appendix, "GBD risk-specific methods summaries."

Calculation for infection-associated cancer burden not estimated by the GBD 2019 study

Approximately 13% of global incident cancer cases (excluding non-melanoma skin cancers) were estimated to be attributable to infection in 2018, equivalent to 2.2 million cancer cases (de Martel et al., 2020).[53] Risk-attributable cervical cancer cases comprised 570,000 of these infection-associated cancers, i.e. 25.9% of all infection-associated cancers (570,000 divided by 2.2 million). Of the remaining 9.6% of all new cancer cases attributable to infections (13*0.741) the GBD 2019 study estimates liver cancer burden attributable to drug use, or 0.70% of total cancer deaths globally in 2018 (https://ghdx.healthdata.org/gbd-results-tool?params=gbd-api-2019-permalink/6d122e54dcf3bf9f07dd0a0456d080e4) leaving 8.9% of all cancer cases or deaths as attributable to infections and not estimated by the GBD 2019 study. This calculation should be interpreted with some caution given that different estimation approaches were used.

References

For methodological summaries included on pages 7-67: ***The Global Burden of Disease (GBD) Study, GATHER Guidelines Checklist, Definition of Indicator, GBD Cancer Estimation Process, and GBD Risk Factor Estimation Process***

1 Kocarnik J, Compton K, Dean FE, *et al*. Cancer incidence, mortality, years of life lost, years lived with disability, and disability-adjusted life years for 29 cancer groups from 2010 to 2019: a systematic analysis for the Global Burden of Disease Study 2019. *JAMA Oncol*. 2022; **8**(3):420-444. doi:10.1001/jamaoncol.2021.6987.

2 Force LM, Abdollahpour I, Advani SM, *et al.* The global burden of childhood and adolescent cancer in 2017: an analysis of the Global Burden of Disease Study 2017. *The Lancet Oncology* 2019; **20**: 1211–25.

3 Vos T, Lim SS, Abbafati C, *et al*. Global burden of 369 diseases and injuries in 204 countries and territories, 1990–2019: a systematic analysis for the Global Burden of Disease Study 2019. *The Lancet* 2020; **396**: 1204–22.

4 Murray CJL, Aravkin AY, Zheng P, *et al*. Global burden of 87 risk factors in 204 countries and territories, 1990–2019: a systematic analysis for the Global Burden of Disease Study 2019. *The Lancet* 2020; **396**: 1223–49.

5 Stevens G, Alkema L, Black R, *et al*. Guidelines for Accurate and Transparent Health Estimates Reporting: the GATHER statement. *The Lancet* 2016; **388**: 19–23.

6 Surveillance, Epidemiology, and End Results (SEER) Program (Www.Seer.Cancer.Gov) SEER*Stat Database: Incidence - SEER 18.

7 Doll R, Payne P, Waterhouse J, editors. *Cancer Incidence in Five Continents, Vol. I*. Geneva: Union Internationale Contre le Cancer, 1966 https://publications.iarc.fr/Non-Series-Publications/Other-Non-Series-Publications/Cancer-Incidence-In-Five-Continents-Volume-I-1966 (accessed Feb 24, 2021).

8 Doll R, Muir CS, Waterhouse JA. *Cancer Incidence in Five Continents, Vol. II*. Geneva: Union Internationale Contre le Cancer, 1970.

9 Waterhouse J, Muir C, Correa P, Powell J. *Cancer Incidence in Five Continents III*. Lyon: IARC; 1976.

10 Waterhouse J, Muir C, Shanmugaratnam K, Powell J. *Cancer Incidence in Five Continents IV*. Lyon: IARC; 1982.

11 Muir C, Mack T, Powell J, Whelan S. *Cancer Incidence in Five Continents V*. Lyon: IARC; 1987.

12 Parkin D, Muir C, Whelan S, Gao Y, Ferlay J, Powell J. *Cancer Incidence in Five Continents VI*. Lyon: IARC; 1992.

13 Parkin D, Whelan S, Ferlay J, Raymond L, Young J. *Cancer Incidence in Five Continents VII*. Lyon: IARC; 1997.

14 Parkin D, Whelan S, Ferlay J, Teppo L, Thomas D. *Cancer Incidence in Five Continents VIII*. Lyon: IARC; 2002.

15 Curado M, Edwards B, Shin H, *et al*. *Cancer Incidence in Five Continents IX*. Lyon: IARC; 2007. http://www.iarc.fr/en/publications/pdfs-online/epi/sp160/CI5vol9-A.pdf.

16 Forman D, Bray F, Brewster D, *et al*. *Cancer Incidence in Five Continents X*. http://ci5.iarc.fr. Published 2013.

17 Bray F, Colombet M, Mery L, *et al.*, editors. *Cancer Incidence in Five Continents*. Lyon, France: International Agency for Research on Cancer, 2017 https://ci5.iarc.fr.

18 Steliarova-Foucher E, O'Callaghan M, Ferlay J, *et al*. The European Cancer Observatory: A new data resource. *Eur J Cancer* 2015; **51**: 1131–43.

19 Engholm G, Ferlay J, Christensen N, *et al.* NORDCAN--a Nordic tool for cancer information, planning, quality control and research. *Acta Oncol* 2010; **49**: 725–36.

20 Barber RM, Fullman N, Sorensen RJD, *et al*. Healthcare Access and Quality Index based on mortality from causes amenable to personal health care in 195 countries and territories, 1990-2015: a novel analysis from the Global Burden of Disease Study 2015. *Lancet* 2017; **390**: 231–66.

21 Foreman KJ, Lozano R, Lopez AD, Murray CJL. Modeling causes of death: an integrated approach using CODEm. *Popul Health Metr* 2012; **10**: 1.

22 Wang H, Abbas KM, Abbasifard M, *et al*. Global age-sex-specific fertility, mortality, healthy life expectancy (HALE), and population estimates in 204 countries and territories, 1950-2019: a comprehensive demographic analysis for the Global Burden of Disease Study 2019. *Lancet* 2020; **396**: 1160–203.

23 Asadzadeh Vostakolaei F, Karim-Kos HE, Janssen-Heijnen MLG, Visser O, Verbeek ALM, Kiemeney LALM. The validity of the mortality to incidence ratio as a proxy for site-specific cancer survival. *Eur J Public Health* 2011; **21**: 573–7.

24 SEER*Stat Software. 2014 http://seer.cancer.gov/seerstat/.

25 Fitzmaurice C, Abate D, Abbasi N, *et al.* Global, Regional, and National Cancer Incidence, Mortality, Years of Life Lost, Years Lived With Disability, and Disability-Adjusted Life-Years for 29 Cancer Groups, 1990 to 2017: A Systematic Analysis for the Global Burden of Disease Study. *JAMA Oncol* 2019; **5**: 1749–68.

26 SEER Cancer Statistics Review 1975-2011. http://seer.cancer.gov/csr/1975_2011/results_merged/topic_survival_by_year_dx.p. .

27 Neal RD, Din NU, Hamilton W, *et al.* Comparison of cancer diagnostic intervals before and after implementation of NICE guidelines: analysis of data from the UK General Practice Research Database. *British Journal of Cancer* 2014; **110**: 584–92.

28 Surveillance, Epidemiology, and End Results (SEER) Program (www.seer.cancer.gov) SEER*Stat Database: Incidence - SEER 18 Regs Research Data + Hurricane Katrina Impacted Louisiana Cases, Nov 2012 Sub (1973-2010 varying) - Linked To County Attributes - Total U.S., 1969-2011 Counties, National Cancer Institute, DCCPS, Surveillance Research Program, Surveillance Systems Branch, released April 2013, based on the November 2012 submission. .

29 Allgar VL, Neal RD. Delays in the diagnosis of six cancers: analysis of data from the National Survey of NHS Patients: Cancer. *Br J Cancer* 2005; **92**: 1959–70.

30 Neal RD, Cannings-John R, Hood K, *et al.* Excision of malignant melanomas in North Wales: effect of location and surgeon on time to diagnosis and quality of excision. *Family Practice* 2008; **25**: 221–7.

31 Kewalramani T, Nimer SD, Zelenetz AD, *et al.* Progressive disease following autologous transplantation in patients with chemosensitive relapsed or primary refractory Hodgkin's disease or aggressive non-Hodgkin's lymphoma. *Bone Marrow Transplant* 2003; **32**: 673–9.

32 Esteban D, Tovar N, Jiménez R, *et al.* Patients with relapsed/refractory chronic lymphocytic leukaemia may benefit from inclusion in clinical trials irrespective of the therapy received: a case-control retrospective analsysis. *Blood Cancer J* 2015; **5**: e356.

33 Canova C, Giorato E, Roveron G, Turrini P, Zanotti R. Validation of a stoma-specific quality of life questionnaire in a sample of patients with colostomy or ileostomy. *Colorectal Dis* 2013; **15**: e692-698.

34 Caricato M, Ausania F, Ripetti V, Bartolozzi F, Campoli G, Coppola R. Retrospective analysis of long-term defunctioning stoma complications after colorectal surgery. *Colorectal Dis* 2007; **9**: 559–61.

35 Erwin-Toth P, Thompson SJ, Davis JS. Factors impacting the quality of life of people with an ostomy in North America: results from the Dialogue Study. *J Wound Ostomy Continence Nurs* 2012; **39**: 417–22; quiz 423–4.

36 Catalona WJ, Carvalhal GF, Mager DE, Smith DS. Potency, continence and complication rates in 1,870 consecutive radical retropubic prostatectomies. *J Urol* 1999; **162**: 433–8.

37 Donnellan SM, Duncan HJ, MacGregor RJ, Russell JM. Prospective assessment of incontinence after radical retropubic prostatectomy: objective and subjective analysis. *Urology* 1997; **49**: 225–30.

38 Eastham JA, Kattan MW, Rogers E, *et al.* Risk factors for urinary incontinence after radical prostatectomy. *J Urol* 1996; **156**: 1707–13.

39 Kundu SD, Roehl KA, Eggener SE, Antenor JAV, Han M, Catalona WJ. Potency, continence and complications in 3,477 consecutive radical retropubic prostatectomies. *J Urol* 2004; **172**: 2227–31.

40 Potosky AL, Davis WW, Hoffman RM, *et al.* Five-Year Outcomes After Prostatectomy or Radiotherapy for Prostate Cancer: The Prostate Cancer Outcomes Study. *JNCI Journal of the National Cancer Institute* 2004; **96**: 1358–67.

41 Sacco E, Prayer-Galetti T, Pinto F, *et al.* Urinary incontinence after radical prostatectomy: incidence by definition, risk factors and temporal trend in a large series with a long-term follow-up. *BJU Int* 2006; **97**: 1234–41.

42 Stanford JL, Feng Z, Hamilton AS, *et al.* Urinary and sexual function after radical prostatectomy for clinically localized prostate cancer: the Prostate Cancer Outcomes Study. *JAMA* 2000; **283**: 354–60.

43 Walsh PC, Marschke P, Ricker D, Burnett AL. Patient-reported urinary continence and sexual function after anatomic radical prostatectomy. *Urology* 2000; **55**: 58–61.

44 Murray CJL, Lopez AD. Global mortality, disability, and the contribution of risk factors: Global Burden of Disease Study. *The Lancet* 1997; **349**: 1436–42.

45 Murray CJL, Lopez AD. On the comparable quantification of health risks: lessons from the Global Burden of Disease Study. *Epidemiology* 1999; **10**: 594–605.

46 Forouzanfar MH, Afshin A, Alexander LT, *et al.* Global, regional, and national comparative risk assessment of 79 behavioural, environmental and occupational, and metabolic risks or clusters of risks, 1990–2015: a systematic analysis for the Global Burden of Disease Study 2015. *The Lancet* 2016; **388**: 1659–724.

47 World Cancer Research Fund, American Institute for Cancer Research. Food, nutrition, and physical activity, and the prevention of cancer: a global perspective. Washington DC: AICR, 2007.

48 Harris PA, Taylor R, Thielke R, Payne J, Gonzalez N, Conde JG. Research Electronic Data Capture (REDCap) - A metadata-driven methodology and workflow process for providing translational research informatics support. *J Biomed Inform* 2009; **42**: 377–81.

49 Vos T, Allen C, Arora M, *et al.* Global, regional, and national incidence, prevalence, and years lived with disability for 310 diseases and injuries, 1990–2015: a systematic analysis for the Global Burden of Disease Study 2015. *Lancet* 2016; **388**: 1545–602.

50 Aravkin A, Davis D. Trimmed statistical estimation via variance reduction. *Mathematics of OR* 2019; published online July 5. https://pubsonline.informs.org/doi/10.1287/moor.2019.0992 (accessed Nov 15, 2019).

51 Vander Hoorn S, Ezzati M, Rodgers A, Lopez AD, Murray CJL. Estimating attributable burden of disease from exposure and hazard data. In: Comparative quantification of health risks: global and regional burden of disease attribution to selected major risk factors. World Health Organization, 2004: 2129–40.

52 Preston SH. Causes and Consequences of Mortality Declines in Less Developed Countries during the Twentieth Century. In: Population and economic change in developing countries. Chicago: Univ. of Chicago Pr, 1980: 289–360.

53 de Martel C, Georges D, Bray F, Ferlay J, Clifford GM. Global burden of cancer attributable to infections in 2018: a worldwide incidence analysis. The Lancet Global Health. 2020; 8: e180–90.

GBD risk-specific methods summaries

The following section provides further methodological detail and GBD case or exposure definitions for risks where the estimation process differs from the general GBD risk factors modelling framework described above. These write-ups were copied from “Section 4: Risk-specific modelling descriptions” in the appendix to the GBD 2019 paper, “Global burden of 87 risk factors in 204 countries and territories, 1990–2019: a systematic analysis for the Global Burden of Disease Study 2019”.[4]

Ambient particulate matter pollution

Flowchart

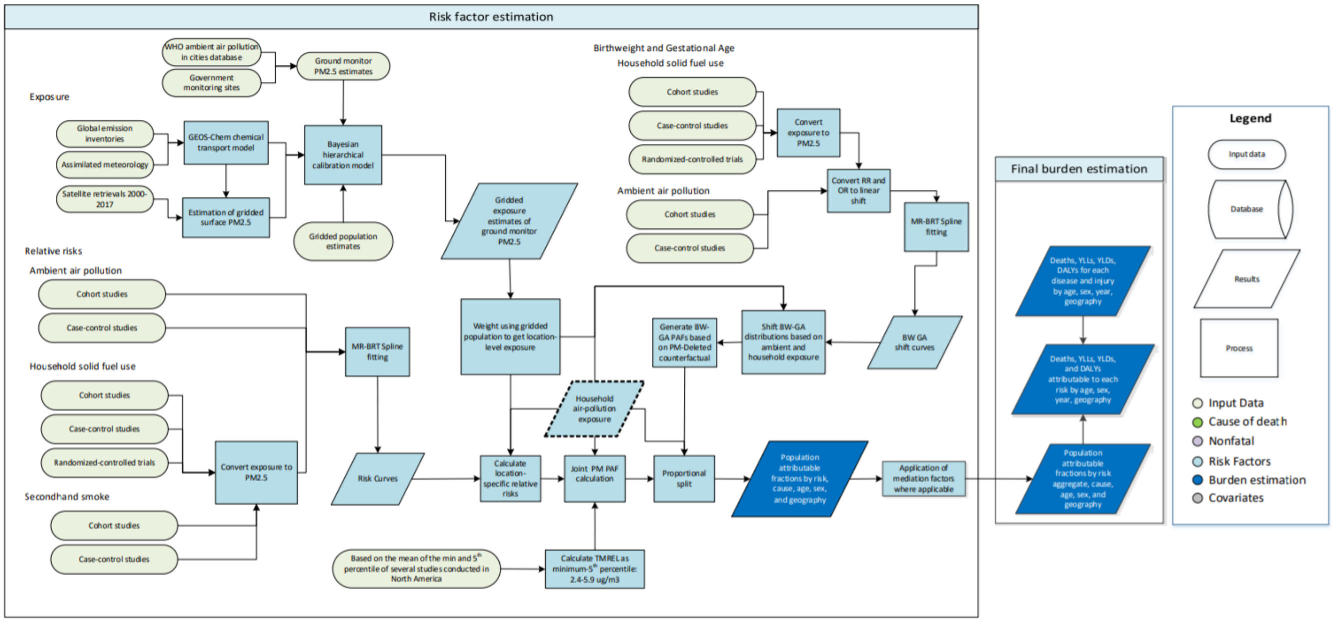

PM2.5 = particulate matter ≤ 2.5 micrometres; WHO = World Health Organization; GEOS-Chem = a chemical transport model of atmospheric chemistry developed at NASA; MR-BRT = a network meta-regression; TMREL = Theoretical minimum-risk exposure level; PAF = Population attributable fraction; RR = Relative Risk; OR = Odds Ratio; BW GA = birthweight and gestational age; YLL = Years of life lost; YLD = Years lived with disability; DALYs = disability-adjusted life-years.

Input data and modelling strategy

Exposure

Definition

Exposure to ambient particulate matter pollution is defined as the population-weighted annual average mass concentration of particles with an aerodynamic diameter less than 2.5 micrometers ($PM_{2.5}$) in a cubic meter of air. This measurement is reported in $\mu g/m^3$.

Input data

The data used to estimate exposure to ambient particulate matter pollution comes from multiple sources, including satellite observations of aerosols in the atmosphere, ground measurements, chemical transport model simulations, population estimates, and land-use data.

The following details the updates in methodology and input data used in GBD 2019.

$PM_{2.5}$ ground measurement database

Ground measurements used for GBD 2019 include updated measurements from sites included in 2017 and additional measurements from new locations. New and up-to-date data (mainly from the USA, Canada, EU, Bangladesh, China and USA embassies and consulates), were added to the data from the 2018 update of the WHO Global Ambient Air Quality Database used in GBD 2017. The updated data

included measurements of concentrations of PM_{10} and $PM_{2.5}$ from 10,408 ground monitors from 116 countries from 2010 to 2017. The majority of measurements were recorded in 2016 and 2017 (as there is a lag in reporting measurements, few data from 2018 or newer were available). Annual averages were excluded if they were based on less than 75% coverage within a year. If information on coverage was not available, then data were included unless there were already sufficient data within the same country (monitor density greater than 0.1).

For locations measuring only PM_{10}, $PM_{2.5}$ measurements were estimated from PM_{10}. This was performed using a hierarchy of conversion factors ($PM_{2.5}/PM_{10}$ ratios): (i) for any location a "local" conversation factor was used, constructed as the ratio of the average measurements (of $PM_{2.5}$ and PM_{10}) from within 50km of the location of the PM_{10} measurement, and within the same country, if such measurements were available; (ii) if there was not sufficient local information to construct a conversion factor then a country-wide conversion factor was used; and (iii) if there was no appropriate information within a country, then a regional factor was used. In each case, to avoid the possible effects of outliers in the measured data (both $PM_{2.5}$ and PM_{10}), extreme values of the ratios were excluded (defined as being greater/lesser than the 95% and 5% quantiles of the empirical distributions of conversion factors). As with GBD 2013, 2015, 2016, and 2017 databases, in addition to values of $PM_{2.5}$ and whether they were direct measurement or converted from PM_{10}, the database also included additional information, where available, related to the ground measurements such as monitor geo-coordinates and monitor site type.

Satellite-based estimates

The global geophysical $PM_{2.5}$ estimates for the years 2000–2017 are from Hammer and colleagues Version V4.GL.03.NoGWR used at $0.1^o x 0.1^o$ resolution (~11 x 11 km resolution at the equator).[1] The method is based on the algorithms of van Donkelaar and colleagues (2016) as used in GBD 2017,[2] with updated satellite retrievals, chemical transport modelling, and ground-based monitoring. The algorithm uses aerosol optical depth (AOD) from several updated satellite products (MAIAC, MODIS C6.1, and MISR v23), including finer resolution, increased global coverage, and improved long-term stability. Ground-based observations from a global sunphotometer network (AERONET version 3) are used to combine different AOD information sources. This is the first time that data from MAIAC at 1 km resolution was used to estimate $PM_{2.5}$ at the global scale. The GEOS-Chem chemical transport model with updated algorithms was used for geophysical relationships between surface $PM_{2.5}$ and AOD. Updates to the GEOS-Chem simulation included improved representation of mineral dust and secondary organic aerosol, as well as updated emission inventories. The resultant geophysical $PM_{2.5}$ estimates are highly consistent with ground monitors worldwide (R^2=0.81, slope = 1.03, n = 2541).

Population data

A comprehensive set of population data, adjusted to match UN2015 Population Prospectus, on a high-resolution grid was obtained from the Gridded Population of the World (GPW) database. Estimates for 2000, 2005, 2010, 2015, and 2020 were available from GPW version 4, with estimates for 1990 and 1995 obtained from the GPW version 3. These data are provided on a $0.0083^o \times 0.0083^o$ resolution. Aggregation to each $0.1^o \times 0.1^o$ grid cell was accomplished by summing the central 12 × 12 population cells. Population estimates for 2001–2004, 2006–2009, 2011–2014 and 2016–2019 were obtained by interpolation using natural splines with knots placed at 2000, 2005, 2010, 2015, and 2020. This was performed for each grid cell.

Chemical transport model simulations

Estimates of the sum of particulate sulfate, nitrate, ammonium, and organic carbon and the compositional concentrations of mineral dust simulated using the GEOS Chem chemical transport model, and a measure combining elevation and the distance to the nearest urban land surface (as described in van Donkelaar and colleagues 2016[2] and Hammer and colleagues[1] were available for 2000–2017 for each $0.1^o \times 0.1^o$ grid cell.

Modelling strategy

The following is a summary of the modelling approach, known as the Data Integration Model for Air Quality 2 (DIMAQ2) used in GBD 2017 and GBD 2019.[3,4]

This model used included within-country calibration variation.[5] In DIMAQ2, ground measurements were matched with other inputs (over time), and the (global-level) coefficients were allowed to vary over time, subject to smoothing that is induced by a first-order random walk process. Additionally, where there are sufficient data, the calibration equations can vary (smoothly) both within and between countries, achieved by allowing the coefficients to follow (smooth) Gaussian processes. Where there are insufficient data within a country, to produce accurate equations information is borrowed from lower down the hierarchy and it is supplemented with information from the wider region.

DIMAQ2 as described above is used for all regions except for the north Africa and Middle East and sub-Saharan Africa super-regions, where there are insufficient data across years to allow the extra complexities of the new model to be implemented. In these super-regions, a simplified version of DIMAQ2 is used in which the temporal component is dropped.

Model evaluation

Model development and comparison was performed using within- and out-of-sample assessment. In the evaluation, cross-validation was performed using 25 combinations of training (80%) and validation (20%) datasets. Validation sets were obtained by taking a stratified random sample, using sampling probabilities based on the cross-tabulation of $PM_{2.5}$ categories (0-24.9, 25-49.9, 50-74.9, 75-99.9, 100+ $\mu g/m^3$) and super-regions, resulting in them having the same distribution of $PM_{2.5}$ concentrations and super-regions as the overall set of sites. The following metrics were calculated for each training/evaluation set combination: for model fit – R^2 and deviance information criteria (DIC, a measure of model fit for Bayesian models); for predictive accuracy – root mean squared error (RMSE) and population weighted root mean squared error (PwRMSE). The median R^2 was 0.9, and the median PwRMSE was 10.1 $\mu g/m^3$.

All modelling was performed on the log-scale. The choice of which variables were included in the model was made based on their contribution to model fit and predictive ability. The following is a list of variables and model structures that were included in DIMAQ.

Continuous explanatory variables:

- (SAT) Estimate of $PM_{2.5}$ (in $\mu g/m^3$) from satellite remote sensing on the log-scale.
- (POP) Estimate of population for the same year as SAT on the log-scale.
- (SNAOC) Estimate of the sum of sulfate, nitrate, ammonium, and organic carbon simulated using the GEOS Chem chemical transport model.
- (DST) Estimate of compositional concentrations of mineral dust simulated using the GEOS-Chem chemical transport model.
- (EDxDU) The log of the elevation difference between the elevation at the ground measurement location and the mean elevation within the GEOS Chem simulation grid cell multiplied by the inverse distance to the nearest urban land surface.

Discrete explanatory variables:

- (LOC) Binary variable indicating whether exact location of ground measurement is known.
- (TYPE) Binary variable indicating whether exact type of ground monitor is known.

- (CONV) Binary variable indicating whether ground measurement is $PM_{2.5}$ or converted from PM_{10}.

Interactions:

- Interactions between the binary variables and the effects of SAT.

Random effects:

- Regional temporal (random walk) hierarchical random-effects on the intercept
- Regional hierarchical random-effects for the coefficient associated with SAT
- Regional hierarchical random-effects for the coefficient associated with POP
- Smoothed, spatially varying random-effects for the intercept
- Smoothed, spatially varying random-effects for the coefficient associated with SAT

Inference and prediction

Due to both the complexity of the models and the size of the data, notably the number of spatial predictions that are required, recently developed techniques that perform "approximate" Bayesian inference based on integrated nested Laplace approximations (INLA) were used.[6] Computation was performed using the R interface to the INLA computational engine (R-INLA). GBD 2019 also makes use of an innovation in the way that samples from the (Bayesian) model are used to represent distributions of estimated concentrations in each grid-cell. Here estimates, and distributions representing uncertainty, of concentrations for each grid are obtained by taking repeated (joint) samples from the posterior distributions of the parameters and calculating estimates based on a linear combination of those samples and the input variables.[7]

DIMAQ2 was used to produce estimates of ambient $PM_{2.5}$ for 1990, 1995, and 2010–2019 by matching the gridded estimates with the corresponding coefficients from the calibration. As there is a lag in reporting ambient air pollution based quantities, the input variables were extrapolated, allowing estimates for 2018 and 2019 to be produced in the same way as other years and, crucially, allowing measures of uncertainty to be produced within the BHM framework rather than by using post-hoc approximations.

Estimates from the satellites and the GEOS-Chem chemical transport model in 2018 and 2019 were produced by extrapolating estimates from 2000–2017 using generalised additive models,[8] on a cell-by-cell basis, except in those grid cells that saw a >100% increase between 2016 and 2017, in which case only the 2000–2016 estimates were used for extrapolating, in order to avoid unrealistic and/or unjustified extrapolation of trends. Population estimates for 2018 and 2019 were obtained by interpolation as described above.

Theoretical minimum-risk exposure level

The TMREL was assigned a uniform distribution with lower/upper bounds given by the average of the minimum and fifth percentiles of outdoor air pollution cohort studies exposure distributions conducted in North America, with the assumption that current evidence was insufficient to precisely characterise the shape of the concentration-response function below the fifth percentile of the exposure distributions. The TMREL was defined as a uniform distribution rather than a fixed value in order to represent the uncertainty regarding the level at which the scientific evidence was consistent with adverse effects of exposure. The specific outdoor air pollution cohort studies selected for this averaging were based on the criteria that their fifth percentiles were less than that of the American Cancer Society Cancer Prevention II (CPSII) cohort's fifth percentile of 8.2 based on Turner and colleagues (2016).[9] This criterion was selected since GBD 2010 used the minimum, 5.8, and fifth percentile solely from the CPS II cohort. The

resulting lower/upper bounds of the distribution for GBD 2019 were 2.4 and 5.9. This has not changed since GBD 2015.

Relative risks and population attributable fractions

For GBD 2019, we made several important changes to the risk functions. Previously, we have used relative risk estimates for active smoking, converting cigarettes-per-day to $PM_{2.5}$ exposure in order to estimate the $PM_{2.5}$ relative risk at the highest end of the $PM_{2.5}$ exposure-response curve. We took this approach because the vast majority of the air pollution epidemiological studies have been performed in low-pollution settings in high-income countries, preventing us from extrapolating the steep relationship at the beginning of the exposure range to locations with high exposure but no relative risk estimates, such as India and China. However, with the recent publication of studies in China and other higher-exposure settings and additional studies of HAP, we have been able to include more estimates at high $PM_{2.5}$ levels in the model.[10-14] Furthermore, in contrast to previous cycles of the GBD where the power function used to develop the IER required the inclusion of active smoking data to anchor the risk function, with the current use of splines and their flexibility, it is easier to fit functions to the (ambient, household, and SHS) data without active smoking data. Beginning in GBD 2019, we excluded active smoking studies from the risk curves. Removal of active smoking information removes an important source of uncertainty in our earlier estimates related to differences in dose rates and other aspects of exposure between active smoking and the other $PM_{2.5}$ sources, including differences in voluntary (active smoking) and involuntary (ambient and household $PM_{2.5}$, secondhand smoke) exposure.[15,16]

Previously we have used a fixed functional form to fit the risk curves.[15] In GBD 2019, we used MR-BRT (described in detail elsewhere) splines to fit the risk data with a more flexible shape. While previously we built in the TMREL estimates into the model fitting, this year we have fit the curve beginning at zero exposure and incorporate the TMREL into the relative risk calculation process. This allows others to use our risk curves with whatever counterfactual level is of interest to them. Relative risk curves are available upon request.

When fitting the risk curves, we consider the published relative risk over a range of exposure data. For OAP studies, the relative risk informs the curve from the fifth to the 95th percentile of observed exposure. When this is not available in the published study, we estimate the distribution from the provided information (mean and standard deviation, mean and IQR, etc.). We scale the RR to this range.
For HAP studies, we allow each study to inform the curve from the Exp_{OAP} to Exp_{OAP}+Exp_{HAP}, where Exp_{OAP} is the GBD 2017 estimate of the ambient exposure level in the study location and year, and Exp_{HAP} is the GBD 2017 estimate of the excess exposure for those who use solid fuel for cooking in the study location and year.

For SHS studies, we updated our strategy of exposure estimation in GBD 2019. For the first time, we are also accounting for outdoor exposure. Similar to the approach used for HAP, we allow each study to inform the curve from the Exp_{OAP} to Exp_{OAP}+Exp_{SHS}, where Exp_{OAP} is the GBD 2017 estimate of the ambient exposure level in the study location and year, and Exp_{SHS} is an estimate of the excess exposure for those who experience secondhand smoke. This is estimated from the number of cigarettes smoked per smoker per day in a given location and year, estimated by the smoking team of GBD, and from a study in Sweden, which measured the $PM_{2.5}$ exposure in homes of smokers.[17] We divided the household $PM_{2.5}$ exposure level by the average number of cigarettes smoked per smoker per day in Sweden over the study duration to estimate the SHS $PM_{2.5}$ exposure per cigarette (2.31 µg/m^3 [95% UI 1.53–3.39]). To calculate Exp_{SHS} we multiplied the estimated number of cigarettes per smoker per day by the average $PM_{2.5}$ exposures per cigarette to generate a predicted $PM_{2.5}$ exposure level.

MR-BRT risk splines

We fit splines on the datasets including studies of OAP, HAP, and SHS using the following functional form, where X and X_{CF} represent the range of exposure characterised by the effect size:

$$log\left(\frac{MRBRT(X)}{MRBRT(X_{CF})}\right) \sim \log(Published\ Effect\ Size)$$

For each of the risk-outcome pairs, we tested various model settings and priors in fitting the MR-BRT splines. The final models used third-order splines with two interior knots and a constraint on the right-most segment, forcing the fit to be linear rather than cubic. We used an ensemble approach to knot placement, wherein 100 different models were run with randomly placed knots and then combined by weighting based on a measure of fit that penalises excessive changes in the third derivative of the curve. Knots were free to be placed anywhere within the fifth and 95th percentile of the data, as long as a minimum width of 10% of that domain exists between them. We included shape constraints so that the risk curves were concave down and monotonically increasing, the most biologically plausible shape for the $PM_{2.5}$ risk curve. On the non-linear segments, we included a Gaussian prior on the third derivative of mean 0 and variance 0.01 to prevent over-fitting; on the linear segment, a stronger prior of mean 0 and variance 1e-6 was used to ensure that the risk curves do not continue to increase beyond the range of the data.

References

For methodological summaries included on pages 73-78: ***Ambient particulate matter pollution***

1. Hammer MS, van Donkelaar A, Li C, *et al.* Global Estimates and Long-Term Trends of Fine Particulate Matter Concentrations (1998–2018). *Environ Sci Technol* 2020; **54**: 7879–90.

2. van Donkelaar A, Martin RV, Brauer M, *et al.* Global Estimates of Fine Particulate Matter using a Combined Geophysical-Statistical Method with Information from Satellites, Models, and Monitors. *Environ Sci Technol* 2016; **50**: 3762–72.

3. Shaddick G, Thomas ML, Green A, *et al.* Data integration model for air quality: a hierarchical approach to the global estimation of exposures to ambient air pollution. *Journal of the Royal Statistical Society: Series C (Applied Statistics)* 2018; **67**: 231–53.

4. Shaddick G, Thomas ML, Mudu P, Ruggeri G, Gumy S. Half the world's population are exposed to increasing air pollution. *npj Clim Atmos Sci* 2020; **3**: 1–5.5.

5. Shaddick G, Thomas M, Amini H, *et al*. Data integration for the assessment of population exposure to ambient air pollution for global burden of disease assessment. *Environ Sci Technol*. 2018 Jun 29. doi: 10.1021/acs.est.8b02864

6. Rue H, Martino S, Chopin N. Approximate Bayesian inference for latent Gaussian models by using integrated nested Laplace approximations. *Journal of the Royal Statistical Society: Series B (Statistical Methodology)* 2009; **71**: 319–92.

7. Thomas ML, Shaddick, G, Simpson D, de Hoogh K, Zidek JV. Spatio-temporal downscaling for continental–scale estimation of air pollution concentrations. arXiv preprint arXiv:1907.00093 (also been Submitted to the *Journal of the Royal Statistical Society: Series C (Applied Statistics))*.

8. Wood SN. (2017). Generalized additive models: an introduction with R. Chapman and Hall/CRC.

9. Turner MC, Jerrett M, Pope CA, *et al.* Long-Term Ozone Exposure and Mortality in a Large Prospective Study. *Am J Respir Crit Care Med* 2016; **193**: 1134–42.

10. Yin P, Brauer M, Cohen A, *et al*. Long-term Fine Particulate Matter Exposure and Nonaccidental and Cause-specific Mortality in a Large National Cohort of Chinese Men. *Environ Health Perspect* 2017; **125**: 117002.

11. Li T, Zhang Y, Wang J, *et al*. All-cause mortality risk associated with long-term exposure to ambient PM2·5 in China: a cohort study. *Lancet Public Health* 2018; **3**: e470–7.

12. Yang Y, Tang R, Qiu H, *et al*. Long term exposure to air pollution and mortality in an elderly cohort in Hong Kong. *Environ Int* 2018; **117**: 99–106.

13. Hystad P, Larkin A, Rangarajan S, *et al*. Outdoor fine particulate matter air pollution and cardiovascular disease: Results from 747 communities across 21 countries in the PURE Study. (Submitted to *Lancet Global Health*)

14. Yusuf S, Joseph P, Rangarajan S, *et al.* Modifiable risk factors, cardiovascular disease, and mortality in 155 722 individuals from 21 high-income, middle-income, and low-income countries (PURE): a prospective cohort study. *The Lancet* 2020; **395**: 795–808.

15. Burnett RT, Pope III CA, Ezzati M, *et al.* An integrated risk function for estimating the global burden of disease attributable to ambient fine particulate matter exposure. *Environmental health perspectives* 2014; **122**: 397.

16. Pope CA, Cohen AJ, Burnett RT. Cardiovascular Disease and Fine Particulate Matter: Lessons and Limitations of an Integrated Exposure Response Approach. *Circ Res* 2018; **122**: 1645–7.

17. Semple S, Apsley A, Ibrahim TA, Turner SW, Cherrie JW. Fine particulate matter concentrations in smoking households: just how much secondhand smoke do you breathe in if you live with a smoker who smokes indoors? *Tob Control* 2015; 24: e205–11.

Household air pollution from solid fuels

Flowchart

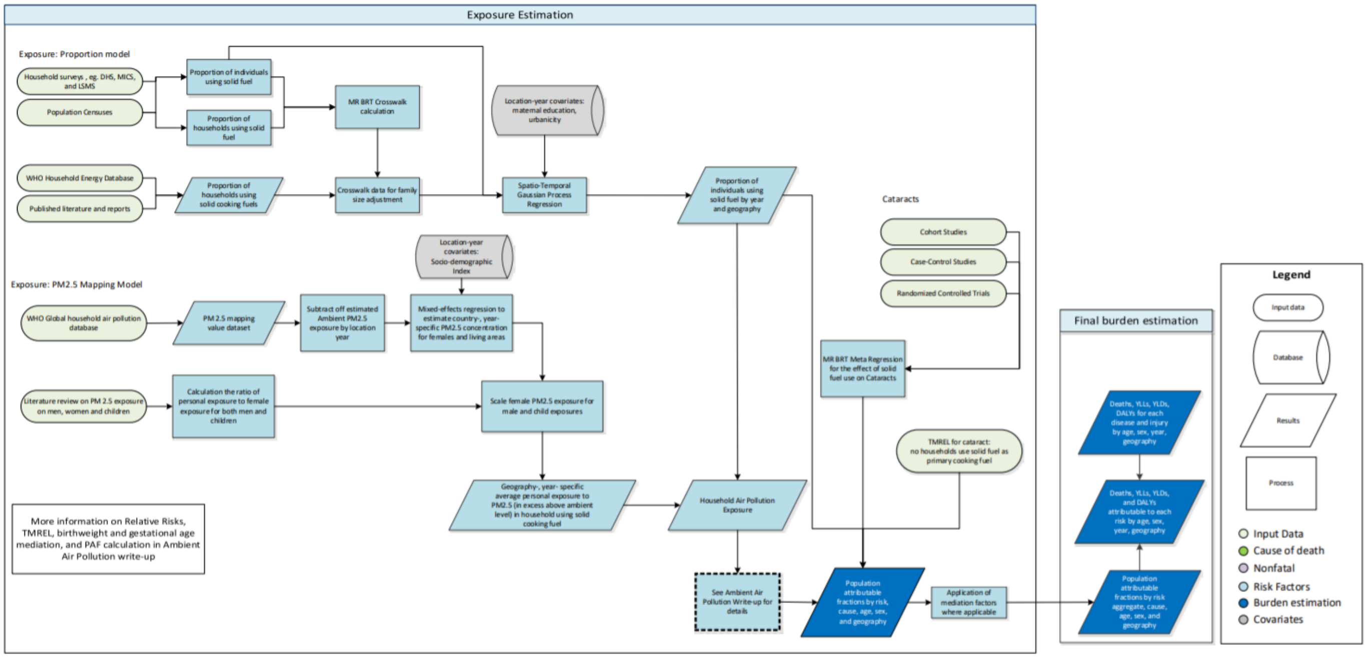

DHS = Demographic and Health Surveys; MICS = Multiple Indicator Cluster Surveys; LSMS = Living Standards Measurement Surveys; WHO = World Health Organization; MR BRT = a network meta-regression; PM2.5 = particulate matter ≤ 2.5 micrometres; TMREL = Theoretical minimum-risk exposure level; PAF = Population attributable fraction; YLL = Years of life lost; YLD = Years lived with disability; DALYs = disability-adjusted life-years.

Input data and methodological summary

Exposure

Case definition

Exposure to household air pollution from solid fuels (HAP) is estimated from both the proportion of individuals using solid cooking fuels and the level of $PM_{2.5}$ air pollution exposure for these individuals. Solid fuels in our analysis include coal, wood, charcoal, dung, and agricultural residues.

Input data

We extracted information on use of solid fuels from the standard multi-country survey series such as Demographic and Health Surveys (DHS), Living Standards Measurement Surveys (LSMS), Multiple Indicator Cluster Surveys (MICS), and World Health Surveys (WHS), as well as censuses and country-specific survey series such as Kenya Welfare Monitoring Survey and South Africa General Household Survey. To fill the gaps of data in surveys and censuses, we also downloaded and updated estimates from WHO Energy Database and extracted from literature through systematic review. Each nationally or subnationally representative datapoint provided an estimate for the percentage of households using solid cooking fuels.

We also excluded sources that did not distinguish specific primary fuel types, estimated fuel used for purposes other than cooking (eg, lighting or heating), failed to report standard error or sample size, had

over 15% of households with missing responses, reported fuel use in physical units, or were secondary sources referencing primary analyses.

Family size crosswalk

Many estimates in the WHO Energy Database and other reports quantify the proportion of households using solid fuel for cooking; however, we are interested in the proportion of individuals using solid fuel for cooking. To crosswalk these estimates, whenever we had the available information, we extracted fuel use at both the individual and household levels. We included 3676 source-specific pairs in the MR-BRT crosswalk model.

MR-BRT crosswalk adjustment factors for household air pollution exposure

**Data input**	**Reference or alternative case definition**	**Gamma**	**Beta coefficient, logit (95% CI)**
Proportion of individuals	Ref	0.097	---
Proportion of Households	Alt		-0.095 (-0.100, -0.090)

We then apply this coefficient to household-only reports with the following formula:

$$\log\left(\frac{prop_{individ}}{1-prop_{individ}}\right) = \log\left(\frac{prop_{hh}}{1-prop_{hh}}\right) - \beta$$

or

$$prop_{individ} = \frac{prop_{hh} * e^{-\beta}}{1 - prop_{hh} + prop_{hh} * e^{-\beta}}$$

$prop_{individ}$ = the proportion of individuals using solid fuel for cooking, and

$prop_{hh}$ = the proportion of households using solid fuel for cooking.

The effect is that the household studies are inflated to account for bias. Larger households are more likely to use solid fuel for cooking.

Modelling strategy

Household air pollution was modelled at individual level using a three-step modelling strategy that uses linear regression, spatiotemporal regression, and Gaussian process regression (GPR). The first step is a mixed-effect linear regression of logit-transformed proportion of individuals using solid cooking fuels. The linear model contains maternal education and the proportion of population living in urban areas as covariates and has nested random effects by GBD region and GBD super-region. The full ST-GPR process is specified in "Section 4.3.3: Spatiotemporal Gaussian process regression (ST-GPR) modelling" in Supplementary Appendix 1 to "Global burden of 369 diseases and injuries in 204 countries and territories, 1990–2019: a systematic analysis for the global burden of disease study 2019".[3]

First-stage linear model and coefficients

$$logit(proportion) \sim maternal\ education + urbanicity + (1|region) + (1|super - region)$$

Variable	Beta (95% CI)
Intercept	3.16 (1.59, 4.74)
Maternal education (years per capita)	-0.45 (-0.76, -0.15)
Urbanicity (proportion of population living in urban areas)	-1.42 (-2.67, -0.17)

Theoretical minimum-risk exposure level

For outcomes related to both ambient and household air pollution, the PAFs are estimated jointly and the TMREL is defined as uniform distribution between 2.4 and 5.9 ug/m^3 $PM_{2.5}$.

Relative risks

Prior to GBD 2019, we utilised the results of an external meta-analysis with a summary relative of 2.47 with 95% CI (1.63, 3.73).[1] While this effect estimate was for both sexes, in the past we estimated burden for women only because women are known to have higher HAP exposure than men. In GBD 2019, we made substantial changes to our particulate matter risk curves. These risk curves, utilising splines in MR-BRT, and the joint-estimation PAF approach are described in the ambient particulate matter appendix.

$PM_{2.5}$ mapping value

In order to use the particulate matter risk curves, we must estimate the level of exposure to particulate matter with diameter of less than 2.5 micrometers ($PM_{2.5}$) for individuals using solid fuels for cooking. The Global Household Air Pollution (HAP) Measurements database from WHO contains 196 studies with measurements from 43 countries of various pollution metrics in households using solid fuel for cooking.[2] From this database, we take all measurements of $PM_{2.5}$ using indoor or personal monitors. In addition to the WHO database, we included eight additional studies from a systematic review conducted in 2015 for GBD.

The final dataset included 336 estimates from 75 studies in 43 unique locations. We included 260, 64, nine, and three measurements indoors, on personal monitors for females, children (under 5), and males, respectively. 274 estimates were in households using solid fuels, 47 in households only using clean (gas or electricity) fuels, and 15 in households using a mixture of solid and clean fuels.

We use the following model:

$$\log(excess\ PM) \sim solid + measure\ group + 24\ hr\ measurement + SDI + (1|study)$$

Where,

- 24-hour measurement: binary variable equal to 1 if the measurement occurred over at least a 24-hour period and not only during mealtimes
- Measure group: categorical variable indicating indoor, female, male, or children
- Solid: indicator variable equal to 1 if the measurements were among households using solid fuel only, 0.5 if the measurements represented a mix of clean and solid fuels, and 0 if the households only used clean fuels.

We also included the Socio-demographic Index (SDI) as a variable to predict a unique value of HAP for each location and year based on development. We also included a random effect on study. We weighted each study by its sample size.

Before modelling, we calculated the excess particulate matter in households using solid fuel by subtracting off the predicted ambient $PM_{2.5}$ value in the study location and year based on the GBD 2017 $PM_{2.5}$ exposure model. The final model coefficients are included below:

HAP mapping model and coefficients

Variable	Beta, log (95% CI)	Beta, adjusted (95% CI)
Intercept	6.23 (4.58, 7.88)	506 (97, 2635)
Solid	2.60 (2.06, 3.13)	13.4 (7.8, 23.0)
Measure group		
• Indoor (ref)		
• Female	-0.56 (-1.15, 0.04)	0.57 (0.32, 1.04)
• Male	-1.56 (-3.81, 0.70)	0.21 (0.02, 2.02)
• Child	-1.13 (-2.06, -0.20)	0.32 (0.13, 0.82)
24-hour measurement	-0.29 (-1.04, 0.46)	0.75 (0.35, 1.59)
SDI	-6.42 (-9.30, -3.54)	1.6 e -3 (9.1 e -5, 2.9 e -2)

Therefore, for females in households using solid fuel, we would expect their long-term mean excess $PM_{2.5}$ exposure due to the use of solid fuels to be 1522, 117, and 9 $\mu g/m^3$ in SDI of 0.1, 0.5, and 0.9, respectively.

Because there are so few studies of personal monitoring in men and children, rather than directly using the results of the model, we generated ratios using studies that measured at least two of the population groups for any size particulate matter. For $PM_{2.5}$ we used the predicted ambient $PM_{2.5}$ value in the study location and year based on the GBD 2017 $PM_{2.5}$ exposure model as the "outdoor" measurement, and for PM_4 and PM_{10} we used published values in the studies themselves. We first subtracted off this outdoor value from each PM measurement, and then calculated the ratio of male to female and child to female exposure, weighted by sample size.

Study	Location	Year	Pollutant	Female N	Female PM	Group	N	PM	Outdoor
Balakrishnan et al., 2004	Andhra Pradesh, Rural	2004	PM_4	591	352	male	503	187	94
Gao X et al., 2009.	Tibet	2009	$PM_{2.5}$	52	127	male	85	111	27
Dasgupta et al., 2006	Bangladesh	2006	PM_{10}	944	209	male	944	166	50
Devkumar et al., 2014	Nepal	2014	$PM_{2.5}$	405	169	male	429	167	90
Balakrishnan et al., 2004	Andhra Pradesh, Rural	2004	PM_4	591	352	child	56	262	94
Dionisio et al., 2008.	The Gambia	2008	$PM_{2.5}$	13	275	child	13	219	31
Dasgupta et al., 2006	Bangladesh	2006	PM_{10}	944	209	child	944	199	50

The final ratios were 0.64 95% CI (0.45, 0.91) for males and 0.85 95% CI (0.56, 1.31) for children. We used these results to scale the $PM_{2.5}$ mapping model for these age and sex groups to input into the $PM_{2.5}$ risk curves.

<u>References</u>

For methodological summaries included on pages 80-84: ***Household air pollution from solid fuels***

1. Smith KR, Bruce N, Balakrishnan K, *et al*. Millions Dead: How Do We Know and What Does It Mean? Methods Used in the Comparative Risk Assessment of Household Air Pollution. *Annu Rev Public Health*. 2014; **35**(1):185–206.

2. Shupler M, Balakrishnan K, Ghosh S, *et al.* Global household air pollution database: Kitchen concentrations and personal exposures of particulate matter and carbon monoxide. *Data Brief* 2018; **21**: 1292–5.

Radon exposure (residential radon)

Flowchart

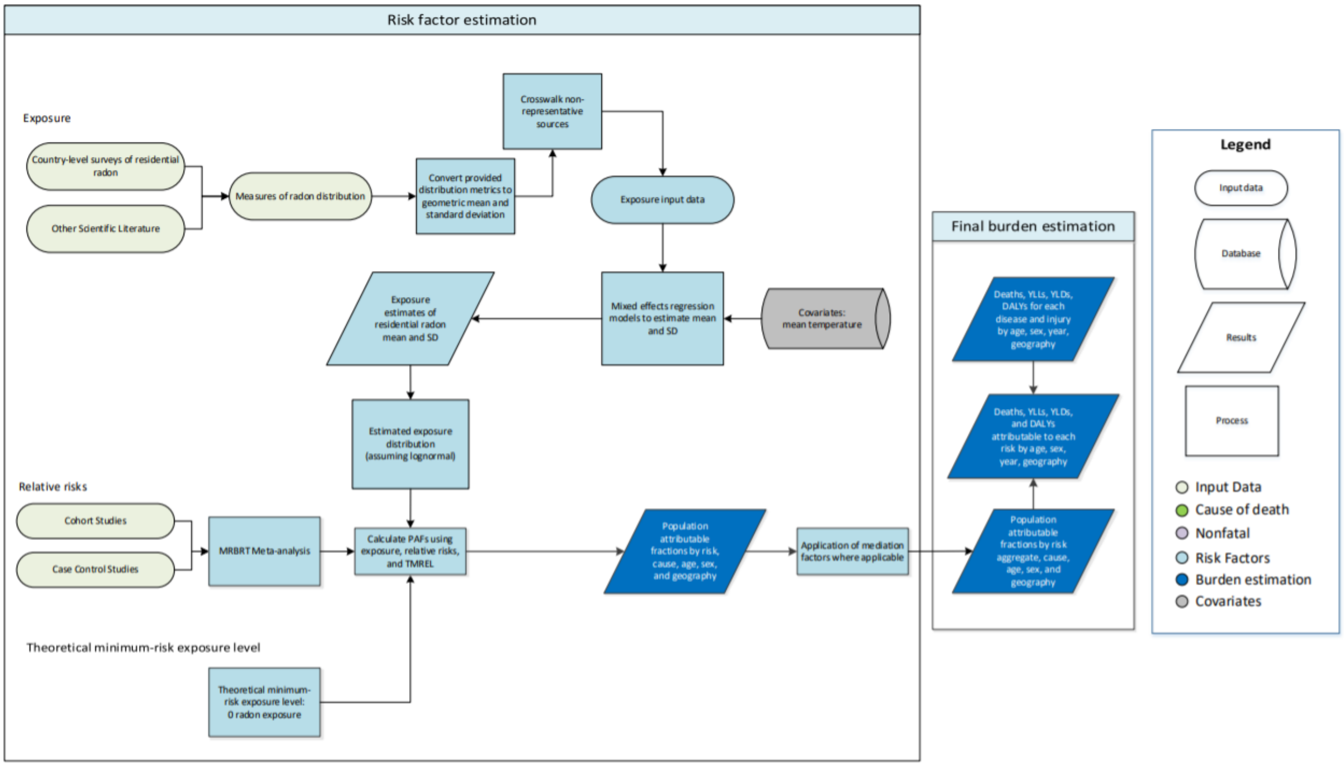

SD = standard deviation; MR-BRT = a network meta-regression; PAF = Population attributable fraction; TMREL = Theoretical minimum-risk exposure level; YLL = Years of life lost; YLD = Years lived with disability; DALYs = disability-adjusted life-years

Input data and methodological summary

Exposure

Case definition

Radon is a radioactive gas that is produced as a byproduct of the decay chain of uranium, occurring naturally within the Earth's crust. Some fraction of this natural radon production escapes into the atmosphere, where it is present at low concentrations unless build-up is caused by release into enclosed spaces such as homes, mines, or caves. Radon exposure is expressed as average daily exposure to indoor air radon gas levels measured in Becquerels (disintegrations per second) per cubic meter (Bq/m^3). In the GBD we specifically quantify the burden due to indoor radon exposure.

Input data

An expert group curated the original dataset for residential radon exposure. We have added data sources every cycle, especially as we include additional subnational locations. Data sources include national surveys, government reports, and scientific literature. We include any sources that report results of residential radon measurement in homes (not schools or workplaces). Because of a shortage of data, we

also include sources that are not representative of an entire population, but exclude studies or surveys explicitly conducted in high-radon areas.

From each source, we extracted all available information required to estimate the distribution of radon exposure, including arithmetic mean and standard deviation, geometric mean and standard deviation, median, IQR, range, max, sample size, confidence interval, and/or standard error.

Modelling strategy

Literature suggests that radon exposure follows a lognormal distribution both on the individual household and national level.[1] We therefore assume that the distribution of radon exposure is lognormal within any one GBD geography or study. For studies reporting at least one measure of central tendency (arithmetic mean, geometric mean, or median) and a measure of spread (standard deviation [arithmetic or geometric], IQR, confidence interval, or standard error), we are able to directly calculate the geometric mean and geometric standard deviation of the underlying distribution. For those only reporting a measure of central tendency and range, max, or sample size, we estimate the geometric mean and standard deviation based on several assumptions.

- When the range or max is provided, we assume that the range divided by 4 is a reasonable estimate of standard deviation because 95% of observations occur within 2 standard deviations of the mean. This calculation happens in log space.
- For studies only providing a measure of central tendency and sample size, we impute standard deviation based on sample size from what we see in other estimates.
- If we only have the mean, we impute the median standard deviation of all other studies.

Once we convert all estimates to the mean and standard deviation of a lognormal distribution, we run all analyses in log-space to meet assumptions of normality.

Though we exclude studies intentionally performed in high-exposure areas, we still see a bias in studies that are not representative of their geography. To account for this difference we perform a crosswalk adjustment using MR-BRT. We match all locations where we have both representative and nonrepresentative sources. These locations include Canada, Egypt, Gansu, Greece, Hiroshima, Ireland, Jordan, Portugal, Puebla, Querétaro, Romania, San Luis Potosí, Saudi Arabia, Shanghai, Spain, Syria, Taiwan, Turkey, Urban Andhra Pradesh, Urban Assam, Urban Gujarat, Urban Haryana, Urban Karnataka, Urban Kerala, Urban Maharashtra, Urban Meghalaya, Urban Punjab, Urban Rajasthan, Urban Tripura, and Urban Uttar Pradesh. We perform the following model on the log difference of the log of the geometric means:

Let $ref = \log(geometric\ mean\ representative)$, and

$alt = \log(geometric\ mean\ non\ representative)$.

$$log\left(\frac{alt}{ref}\right) \sim Beta$$

$$ref \sim e^{-Beta} * alt$$

$$ref \sim (adjustment\ factor) * alt$$

We use the results of this crosswalk to downscale all non-representative input sources and inflate their uncertainty in the model. The effect is equivalent to scaling the log of the geometric mean of non-representative sources by a factor of 0.899.

MR-BRT crosswalk adjustment factor for radon exposure

Data input	Reference or alternative case definition	Gamma	Beta coefficient, log (95% UI)	Adjustment factor*
Geographically representative survey or report	Ref	0.29	---	
Estimate not representative of geographic unit	Alt		0.106 (0.095, 0.112)	0.899 (0.894, 0.909)

*Adjustment factor is the transformed beta coefficient in normal space, and can be interpreted as the factor by which the alternative case definition is adjusted to reflect what it would have been if measured as the reference.

After crosswalking non-representative sources, we run a model to estimate the log(geometric mean). Because radon is naturally occurring and is not considered to have much long-term temporal fluctuation, we used a mixed effects linear model independent of time.[2] The model included nested random effects on super-region, region, and location (most detailed) and one fixed effect covariate, long-term mean temperature (average annual temperature averaged over 1990–2019) as a proxy for adequate building ventilation. We weighted the model by inverse standard error. We tried weighting by inverse variance and sample size, but did not get a stable fit. To predict the log of the geometric mean we used the following model:

$$\log(geometric\ mean) \sim \beta * long\ term\ mean\ temp + (1|super\ region) + (1|region) + (1|location)$$

Regression coefficients for predicting mean radon

Input	Coefficient (95% UI)
Intercept	4.05 (3.532, 4.560)
Long term mean temperature	-0.040 (-0.065, -0.015)

We also ran a model to predict the standard deviation (in log space) for every country. We included all studies that were representative of a geography and that included a measure of spread for which we were able to directly calculate the standard deviation. The model was a mixed effects linear regression of standard deviation on mean including random effects on location (most-detailed) and region. The model was not stable when including super-region. To predict the log of the geometric standard deviation we used the following model:

$$\log(geometric\ standard\ deviation) \sim \beta * \log(geometric\ mean) + (1|region) + (1|location)$$

Regression coefficients for predicting standard deviation of radon

Input	Coefficient (95% UI)
Intercept	0.616 (0.389, 0843)
log(geometric mean)	0.014 (-0.030, 0.057)

We used the estimated mean and standard deviation for each location to generate an exposure distribution used in PAF calculation.

Theoretical minimum-risk exposure level

While in GBD 2017 we sampled from a uniform distribution from 7-14 Bq/m3 representing outdoor air, in GBD 2019 we updated the radon TMREL to zero. This was decided because the risk we are estimating is indoor air radon, and it is theoretically possible with mitigation strategies to reduce all indoor exposure to zero.

Relative risks

In GBD 2017, the RR was based on a single meta-analysis (Darby and colleagues 2005) which reported a relative risk of 1.16 (1.05–1.31) per 100 Bq/m3 increase in radon exposure. In GBD 2019 we conducted a systematic review of studies examining residential exposure to radon and lung cancer incidence or mortality. We extracted the component studies from several meta-analyses.[3-8] We excluded studies that were cross-sectional or ecological, studied high-risk populations such as miners, or were not available in English. When multiple studies were published on the same dataset, we took the one with the longest follow-up. We also excluded studies that only reported cumulative exposure because this does not align with our exposure definition.

Some studies only reported RR between exposure categories. In these instances, we took the mean, median, or midpoint of the exposed and unexposed categories to calculate an "exposure range". We then scaled the reported RR based on that exposure range to estimate the corresponding increase per 100 units. This resulted in a total of 49 estimates from 25 studies in 12 countries: England, Czechia, Finland, France, Germany, Italy, Spain, Sweden, the USA, China, Denmark, and Japan.

For those studies that reported no confidence intervals or standard error, we imputed the standard error based on sample size. To do this we created a model of the following form:

$$se \sim \beta * \frac{1}{\sqrt{n}}$$

where we predict the standard error, se, as a function of some constant, β, times the inverse square root of the sample size, n. Here β is an estimate of the population-level standard deviation.

Once we had all 49 estimates of the RR increase per 100 unit change in exposure, we fit a MR-BRT meta-regression including covariates for selection bias and quality of exposure measurement. Studies that included a full residential history were assigned a 0 for cv_exposure_study, while those who only measured the current household or one household were assigned a 1. We assigned studies to one of three categories for selection bias. Studies with greater than 95% follow-up received a 0, those with 85% to 85% follow-up received a 1, and those with less than 85% follow-up received a 2. For case-control studies we assigned this based on the percentage of cases and controls for which exposure category could be ascertained.

We also included loose priors on Gamma and each of the covariates. The prior on Gamma was a gamma distribution with mean 0.2 and variance 0.1. The prior on each of the covariates was a gamma distribution with mean 0 and variance 0.1.

MR-BRT relative risk meta-regression for radon

**Data input**	**Gamma**	**Beta coefficient, log (95% CI)**	**Exponentiated coefficient (95% CI)**
Intercept	0	0.094 (0.023, 0.165)	1.09 (1.02, 1.18)
cv_exposure_study	0.23	0.124 (-0.060, 0.308)	1.13 (0.94, 1.36)
cv_selection_bias	0	-0.017 (-0.073, 0.038)	0.98 (1.04, 0.93)

References

For methodological summaries included on pages 85-89: ***Radon exposure (residential radon)***

1. Daraktchieva Z, Miles JCH, McColl N. Radon, the lognormal distribution and deviation from it. *J Radiol Prot* 2014; **34**: 183–190.

2. Steck DJ. Annual average indoor radon variations over two decades. *Health Phys* 2009; **96**(1): 37-47.

3. Darby S, Hill D, Auvinen A, *et al*. Radon in homes and risk of lung cancer: collaborative analysis of individual data from 13 European case-control studies. *BMJ*. 2005; **330**(7485): 223.

4. Lubin JH. Studies of radon and lung cancer in North America and China. *Radiation Protection Dosimetry* 2003; **104**(4): 315–9.

5. Krewski D, Lubin JH, Zielinski JM, Alavanja M, Catalan VS, Field RW, et al. Residential Radon and Risk of Lung Cancer. *Epidemiology* 2005; **16**(2): 137–45.

6. Zhang Z-L, Sun J, Dong J-Y, *et al*. Residential radon and lung cancer risk: an updated meta-analysis of case-control studies. *Asian Pac J Cancer Prev* 2012; **13**: 2459–65.

7. Torres-Durán MCAD, Barros-Dios JM, Fernández-Villar A, Ruano-Ravina A. Residential radon and lung cancer in never smokers. A systematic review. *Cancer Letters* 2014; **345**(1): 21–6.

8. Dobrzyński L, Fornalski KW, Reszczyńska J. Meta-analysis of thirty-two case–control and two ecological radon studies of lung cancer. *J Radiat Res* 2018; **59**: 149–63.

Occupational risks

Flowchart, Occupational risk factors (except asbestos and injuries)

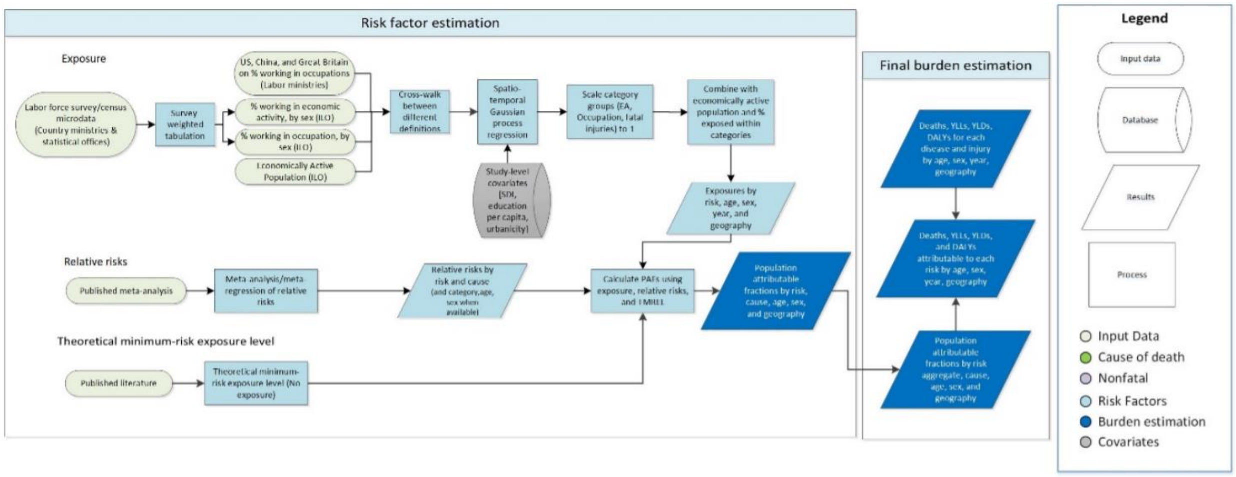

SDI = Socio-demographic Index; EA = Economic Activity; ILO = International Labor Organization; YLL = Years of life lost; YLD = Years lived with disability; DALYs = Disability-adjusted life-years.

Flowchart, Occupational risk factors (asbestos)

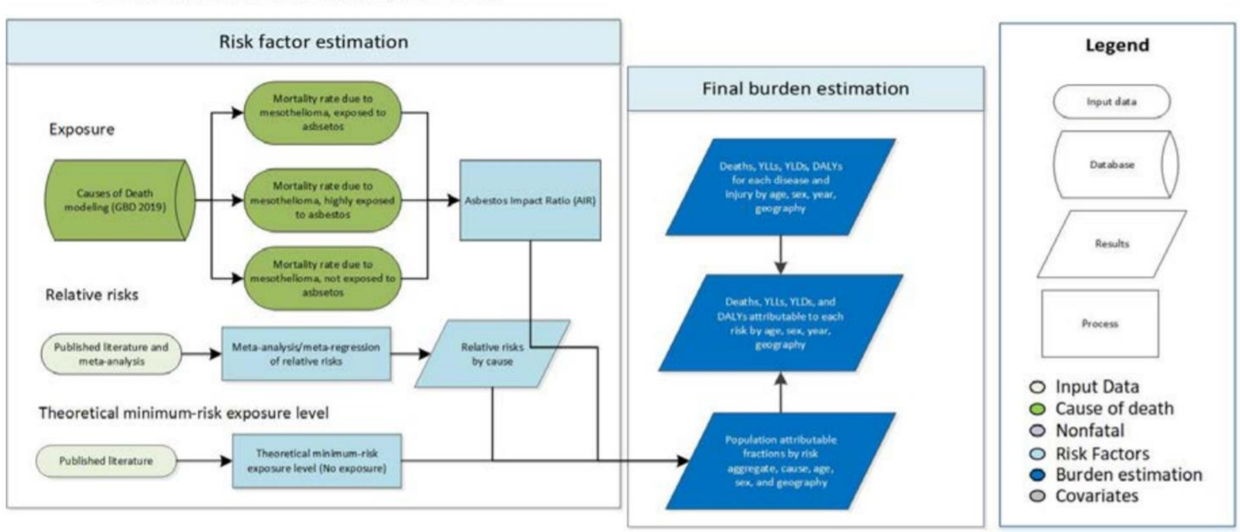

GBD = Global Burden of Disease Study; YLL = Years of life lost; YLD = Years lived with disability; DALYs = Disability-adjusted life-years.

Input data and methodological summary

Exposure

*Case definition, occupational carcinogens**

Proportion of the population that was ever occupationally exposed to carcinogens at high or low exposure levels, based on population distributions across 17 economic activities: agriculture, hunting, forestry; fishing; mining and quarrying; manufacturing; electricity, gas, and water; construction; wholesale and retail trade/repair; hospitality; transport, storage, and communication; financial intermediation; real

estate/renting; public administration/defense and compulsory social security; education; health and social work; other community/social/personal service activities; private households; and, extra-territorial organisations/bodies.

Exposure was estimated for ages 15 and older.

*Occupational carcinogens include: arsenic, benzene, beryllium, cadmium, chromium, diesel engine exhaust, formaldehyde, nickel, polycyclic aromatic hydrocarbons, silica, sulfuric acid, and trichloroethylene.

Case definition, asbestos
Proportion of the population occupationally exposed to asbestos, using mesothelioma death rate as an analogue.

Exposure was estimated for ages 15 and older

Input data
Primary inputs were obtained from the International Labour Organization (ILO).[1-4] These inputs included raw data on economic activity proportions, occupation proportions, fatal injury rates, and employment to population ratio estimates. No data on informal employment was included due to data sparseness. In 2017, a systematic review was conducted in order to collect the underlying microdata from the ILO's estimates to aid in re-extraction at greater levels of granularity. Where freely available, survey datasets were downloaded from the survey organisations in question. Other datasets were obtained through submission of requests to agencies and through the GBD collaborator network. Microdata were tabulated in order to create survey-weighted estimates of economic activities and occupations for the GBD geographies and years. Various classification systems were adjusted to match the ISIC Rev.3 classification (for economic activities) and ISCO 1988 classification (for occupations).

In GBD 2019, a substantial amount of new ILO data were added. The new data comprise 1197 new unique location-years, including 174 unique locations and 13 unique years (2006–2018). Additionally, a number of old microdata were re-extracted.

For occupational asbestos, primary inputs were obtained through GBD 2019 cause of death estimates and published studies.[3, 5, 6]

Uncertainty for inputs where microdata were unavailable was generated by fitting a Loess curve to the data and determining the standard deviation of the data from the fitted curve.

Modelling strategy

A spatiotemporal Gaussian process regression (ST-GPR) was used to generate estimates for all years and locations for the primary inputs. Space-time parameters were chosen by maximising out-of-sample cross-validation and minimising RMSE. A number of different study-level covariates were used in the linear regression models. The linear models for each of the 46 different ST-GPR models used in occupational exposure estimation are listed below. Although there might appear to be duplicates, there is a distinction between occupation and economic activity (detailed in the footnotes). For example, "skilled agriculture/fisheries" involves the proportion of the workforce doing agricultural and fishing work, while "agriculture, hunting, forestry" and "fishing" involve the proportion of the workforce employed in those respective industries (ie, one doesn't have to be actually doing agricultural or fishing work – someone

who transports crops would count as being employed in this industry, but their occupation would fall under "plant and machine operators & assemblers"). Additionally, each model included random effects at the region and super-region levels. The covariates are explained in greater detail below.

ST-GPR model	Linear regression equation
Employment (% of population employed)	*logit*(*data*) = *gov_exp* + *prop_muslim* + *education*
Armed forces*	*logit*(*data*) = *sdi* + *education* + *urbanicity*
Management*	*logit*(*data*) = *sdi* + *education* + *urbanicity*
Professional Occupations*	*logit*(*data*) = *sdi* + *education* + *urbanicity*
Scientific/technicians*	*logit*(*data*) = *sdi* + *education* + *urbanicity*
Clerical work*	*logit*(*data*) = *sdi* + *education* + *urbanicity*
Service & shop/market sales workers*	*logit*(*data*) = *sdi* + *education* + *urbanicity*
Skilled agriculture/fisheries*	*logit*(*data*) = *sdi* + *latitude* + *urbanicity*
Craft and relate trades*	*logit*(*data*) = *sdi* + *education* + *urbanicity*
Plant and machine operators & assemblers*	*logit*(*data*) = *sdi* + *education* + *urbanicity*
Elementary occupations*	*logit*(*data*) = *sdi* + *education* + *urbanicity*
Agriculture, hunting, forestry†	*logit*(*data*) = *sdi* + *latitude* + *urbanicity*
Fishing†	*logit*(*data*) = log(*coastal_prop* + 0.01)
Mining/quarrying†	*logit*(*data*) = *sdi* + log(*coastal_prop* + 0.01) + *urbanicity* + *asbestos*
Manufacturing†	*logit*(*data*) = *sdi* + *education* + *urbanicity*
Electricity/gas/water supply†	*logit*(*data*) = log(*sdi*) + *urbanicity* + *temperature*
Construction†	*logit*(*data*) = *sdi* + *urbanicity*
Wholesale and retail trade/repair†	*logit*(*data*) = *sdi* + *education* + *urbanicity*
Hospitality†	*logit*(*data*) = *sdi* + *urbanicity*
Transport/storage/communications†	*logit*(*data*) = *sdi* + *urbanicity* + *vehicles_pc*
Financial intermediation†	*logit*(*data*) = *sdi* + *urbanicity*
Real estate/renting†	*logit*(*data*) = *sdi* + *urbanicity*
Public administration/defence†	*logit*(*data*) = *sdi* + *urbanicity*
Education†	*logit*(*data*) = *sdi* + *education* + *urbanicity*
Health and social work†	*logit*(*data*) = log(*sdi*) + log(*health_exp*)
Other community/social/personal service activities†	*logit*(*data*) = *sdi* + *urbanicity*
Private households†	*logit*(*data*) = *sdi* + *urbanicity*
Extraterritorial organisations and bodies†	*logit*(*data*) = *sdi* + *urbanicity*
All occupational injuries models‡	*logit*(*data*) = *sdi*

**Proportion of workforce working this type of occupation*
†Proportion of workforce employed in this type of economic activity
‡There are 18 different models, corresponding to one for each type of economic activity and a "total" model

Covariate	Description
gov_exp	Total government expenditure
prop_muslim	Proportion of population that is Muslim
education	Age-standardised years of education per capita
sdi	Socio-demographic Index

urbanicity	Proportion of population living in urban areas
latitude	Absolute value of average latitude of country's center point
coastal_prop	Percentage of total country area within 10 km of a coastal zone
asbestos	Asbestos consumption (metric tons per year per capita)
temperature	Population-weighted mean temperature
vehicles_pc	Number of 2- and 4-wheeled vehicles per capita
health_exp	Total health expenditure per capita

Occupational carcinogens, occupational noise, and occupational particulates
Prevalence of exposure to these risks was determined using the following equation:

$$Prevalence\ of\ Exposure_{c,y,s,a,r,l} = \sum_{EA} Proportion_{EA,c,y} * EAP_{c,y,s,a} * Exposure\ rate_{EA,r,l,d}$$

where:

EAP = economically active population	c = country	r = risk
EA = economic activity	d = duration	s = sex
a = age	l = level of exposure	y = year

Exposure rate (proportion of population exposed) was provided by expert group recommendations and literature.[7-9] The CAREX (carcinogen exposure) database[8] was used in order to quantify the association between exposure by industry/carcinogen to SDI across all the countries in the database. This effect was used to predict exposure in countries that were not included in CAREX. Duration was considered for occupational carcinogens through application of occupational turnover factors12 and for occupational noise and particulates by calculating cumulative exposure as the average exposure over the lifetime (the past 50 years) for each age/sex cohort.

Occupational asbestos
Prevalence of exposure to asbestos was estimated using the asbestos impact ratio (AIR), which is equivalent to the excess deaths due to mesothelioma observed in a population divided by excess deaths due to mesothelioma in a population heavily exposed to asbestos. Formally, this is defined using the following equation:

$$AIR = \frac{Mort_{c,y,s} - N_{c,y,s}}{Mort^{*}_{c,ys,} - N_{c,y,s}}$$

where:
Mort = Mortality rate due to mesothelioma
Mort* = Mortality rate due to mesothelioma in population highly exposed to asbestos
N = Mortality rate due to mesothelioma in population not exposed to asbestos
c = country
y = year
s = sex

Mortality rate due to mesothelioma was estimated using GBD 2019 causes of death results. Mortality rate due to mesothelioma in populations not exposed to asbestos was calculated using the model in Lin and colleagues,[5] while the mortality rate due to high exposure to asbestos was estimated using Goodman and colleagues' model.[6] Asbestos exposure prevalence created using the AIR was used to estimate population attributable fractions (PAFs) for all asbestos-associated causes except for mesothelioma. Custom PAFs were calculated for mesothelioma by using the ratio of the excess mortality with respect to an unexposed population (Mort – N) divided by the mortality rate in the population in question (Mort). This calculation assumes that all mesothelioma is a product of occupational asbestos exposure and could potentially overestimate the burden due to occupational asbestos exposure in populations with high non-occupational asbestos exposure.

Theoretical minimum-risk exposure level
For all occupational risks, the theoretical minimum-risk exposure level was assumed to be no exposure to that risk.

Relative risks
Relative risks were obtained for all occupational risks by conducting a systematic review of published meta-analyses. This review was last updated for GBD 2016.

References
For methodological summaries included on pages 90-94: ***Occupational risks***

1. International Labour Organization (ILO). International Labour Organization Database (ILOSTAT) - Employment by Sex and Economic Activity. International Labour Organization (ILO).

2. International Labour Organization (ILO). International Labour Organization Database (ILOSTAT) - Employment by Sex and Occupation. International Labour Organization (ILO).

3. International Labour Organization (ILO). International Labour Organization Database (ILOSTAT) – Fatal Injuries by Sex and Economic Activity. International Labour Organization (ILO).

4. International Labour Organization (ILO). International Labour Organization LABORSTA Economically Active Population, Estimates and Projections, October 2011. International Labour Organization (ILO), 2011.

5. Lin R-T, Takahashi K, Karjalainen A, *et al*. Ecological association between asbestos-related diseases and historical asbestos consumption: an international analysis. *Lancet* 2007; **369**: 844–9.

6. Goodman M, Morgan RW, Ray R, Malloy CD, Zhao K. Cancer in asbestos-exposed occupational cohorts: a meta-analysis. *Cancer Causes Control* 1999; **10**: 453–65.

7. Wilson DH, Walsh PG, Sanchez L, *et al*. The epidemiology of hearing impairment in an Australian adult population. *Int J Epidemiol* 1999; **28**: 247–52.

8. Kauppinen T, Toikkanen J, Pedersen D, *et al*. Occupational exposure to carcinogens in the European Union. *Occup Environ Med* 2000; **57**(1): 10–18.

9. Driscoll T, Nelson DI, Steenland K, *et al*. The global burden of non-malignant respiratory disease due to occupational airborne exposures. *American Journal of Industrial Medicine* 2005; **48**(6): 432-445.

<u>Smoking</u>

Flowchart

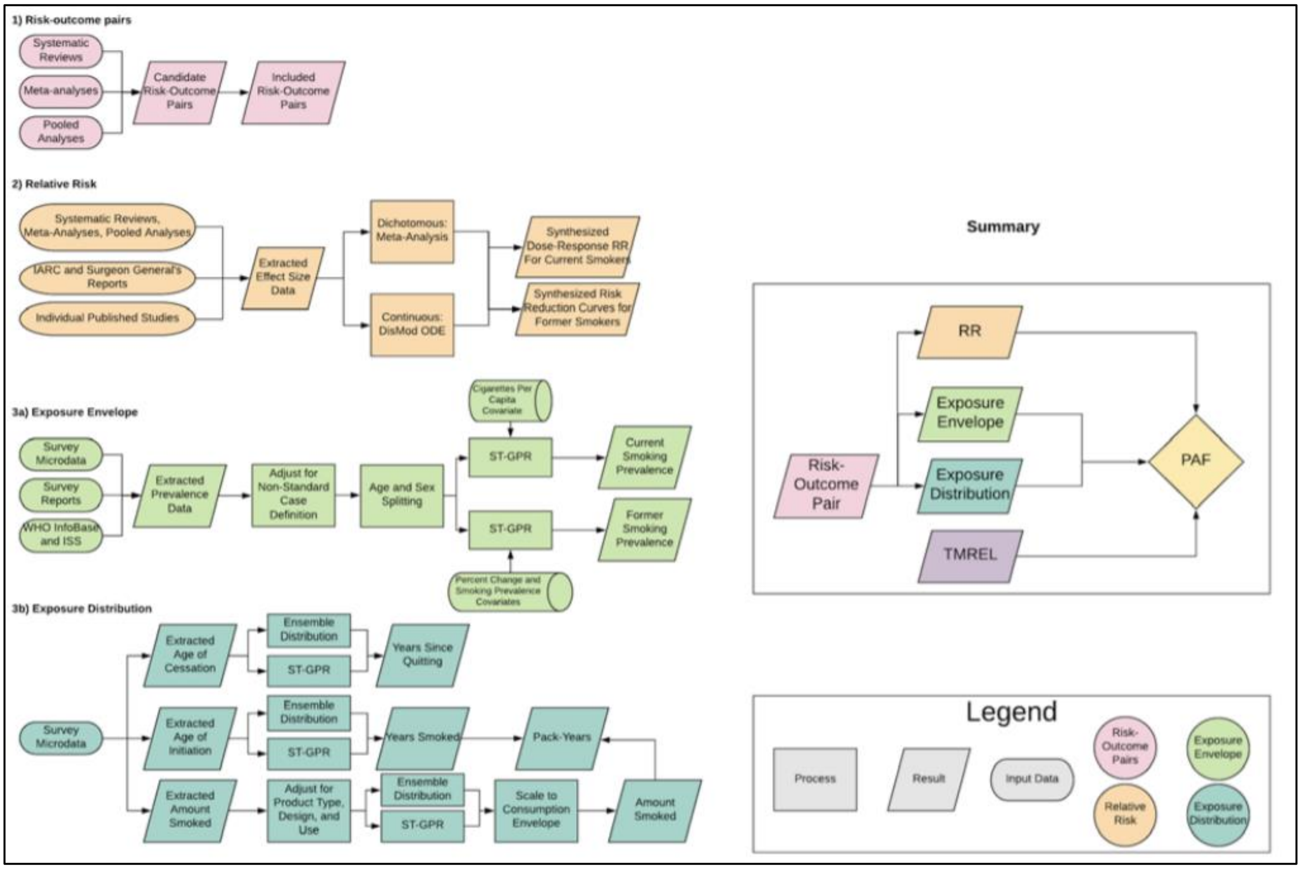

IARC = International Agency for Research on Cancer; RR = relative risk; WHO = World Health Organization; ST-GPR = spaciotemporal Gaussian process regression; PAF = Population attributable fraction; TMREL = Theoretical minimum-risk exposure level.

Input data and methodological summary

Exposure

Case definition

We estimated the prevalence of current smoking and the prevalence of former smoking using data from cross-sectional nationally representative household surveys. We defined current smokers as individuals who currently use any smoked tobacco product on a daily or occasional basis. We defined former smokers as individuals who quit using all smoked tobacco products for at least six months, where possible, or according to the definition used by the survey.

Input data

We extracted primary data from individual level microdata and survey report tabulations. We extracted data on current, former, and/or ever smoked tobacco use reported as any combination of frequency of use (daily, occasional, and unspecified, which includes both daily and occasional smokers) and type of

smoked tobacco used (all smoked tobacco, cigarettes, hookah, and other smoked tobacco products such as cigars or pipes), resulting in 36 possible combinations. Other variants of tobacco products, for example hand-rolled cigarettes, were grouped into the four type categories listed above based on product similarities.

For microdata, we extracted relevant demographic information, including age, sex, location, and year, as well as survey metadata, including survey weights, primary sampling units, and strata. This information allowed us to tabulate individual-level data in the standard GBD five-year age-sex groups and produce accurate estimates of uncertainty. For survey report tabulations, we extracted data at the most granular age-sex group provided.

Crosswalk
Our GBD smoking case definitions were current smoking of any tobacco product and former smoking of any tobacco product. All other data points were adjusted to be consistent with either of these definitions. Some sources contained information on more than one case definition and these sources were used to develop the adjustment coefficient to transform alternative case definitions to the GBD case definition. The adjustment coefficient was the beta value derived from a linear model with one predictor and no intercept. We used the same crosswalk adjustment coefficients as in GBD 2017, and thus we have not included a methods explanation in this appendix, as it has been detailed previously.

Age and sex splitting
As in GBD 2017, we split data reported in broader age groups than the GBD 5-year age groups or as both sexes combined by adapting the method reported in Ng et al to split using a sex- geography- time specific reference age pattern.[1] We separated the data into two sets: a training dataset, with data already falling into GBD sex-specific 5-year age groups, and a split dataset, which reported data in aggregated age or sex groups. We then used spatiotemporal Gaussian process regression (ST-GPR) to estimate sex-geography-time-specific age patterns using data in the training dataset. The estimated age patterns were used to split each source in the split dataset.

The ST-GPR model used to estimate the age patterns for age-sex splitting used an age weight parameter value that minimises the effect of any age smoothing. This parameter choice allowed the estimated age pattern to be driven by data, rather than being enforced by any smoothing parameters of the model. Because these age-sex split data points were to be incorporated in the final ST-GPR exposure model, we did not want to doubly enforce a modelled age pattern for a given sex-location-year on a given aggregate data point.

Modelling strategy

Smoking prevalence modelling
We used ST-GPR to model current and former smoking prevalence. The model is nearly identical to that in GBD 2017. Full details on the ST-GPR method are reported elsewhere in the appendix. Briefly, the mean function input to GPR is a complete time series of estimates generated from a mixed effects hierarchical linear model plus weighted residuals smoothed across time, space, and age. The linear model formula for current smoking, fit separately by sex using restricted maximum likelihood in R, is:

$$logit(p_{g,a,t}) = \beta_0 + \beta_1 CPC_{g,t} + \sum_{k=2}^{19} \beta_k I_{A[a]} + \alpha_s + \alpha_r + \alpha_g + \epsilon_{g,a,t}$$

where $CPC_{g,t}$ is the tobacco consumption covariate by geography g and time t, described above, $I_{A[a]}$ is a dummy variable indicating specific age group A that the prevalence point $p_{g,a,t}$ captures, and α_s, α_r, and α_g are super-region, region, and geography random intercepts, respectively. Random effects were used in model fitting but not in prediction.

The linear model formula for former smoking is:

$$logit(p_{g,a,t}) = \beta_0 + \beta_1 PctChange_{A[a],g,t} + \beta_3 CSP_{A[a],g,t} + \sum_{k=3}^{20} \beta_k I_{A[a]} + \alpha_s + \alpha_r + \alpha_g + \epsilon_{g,a,t}$$

where $PctChange_{A[a],g,t}$ is the percentage change in current smoking prevalence from the previous year, and $CSP_{A[a],g,t}$ is the current smoking prevalence by specific age group A, geography g, and time t that point $p_{g,a,t}$ captures, both derived from the current smoking ST-GPR model defined above.

Supply-side estimation

The methods for modelling supply-side-level data were changed substantially from those used in GBD 2017. The raw data were domestic supply (USDA Global Surveillance Database and UN FAO) and retail supply (Euromonitor) of tobacco. Domestic supply was calculated as production + imports - exports. The data went through three rounds of outliering. First, they were age-sex split using daily smoking prevalence to generate number of cigarettes per smoker per day for a given location-age-sex-year. If more than 12 points for a particular source-location-year (equal to over 1/3 of the split points) were above the given thresholds, that source-location-year was outliered. A point would not be outliered if it was (in cigarettes per smoker): under five (10–14 year olds); under 20 (males, 15–19 year olds); under 18 (females, 15–19 year olds); under 38/35 and over three (males/females, 20+ year olds). These thresholds were chosen by visualising histograms of the data for each age-sex, as well as with expert knowledge about reasonable consumption levels. In the second round of outliering, the mean tobacco per capita value over a 10-year window was calculated. If a point was over 70% of that mean value away from the mean value, it was outliered. The 70% limit was chosen using histograms of these distances. Additionally, some manual outliering was performed to account for edge cases. Finally, data smoothing was performed by taking a three-year rolling mean over each location-year.

Next, a simple imputation to fill in missing years was performed for all series to remove compositional bias from our final estimates. Since the data from our main sources covered different time periods, by imputing a complete time series for each data series, we reduced the probability that compositional bias of the sources was leading to biased final estimates. To impute the missing years for each series, we modelled the log ratio of each pair of sources as a function of an intercept and nested random effects on super-region, region, and location. The appropriate predicted ratio was multiplied by each source that we did have, and then the predictions were averaged to get the final imputed value. For example, if source A was missing for a particular location-year, but sources B and C were present, then we predicted A twice: once from the modelled ratio of A to B, and again from the modelled ratio of A to C. These two predictions were then averaged. For some locations where there was limited overlap between series, the predicted ratio did not make sense, and a regional ratio was used.

Finally, variance was calculated both across series (within a location-year) as well as across years (within a location-source). Additionally, if a location-year had one imputed point was, the variance was multiplied by 2. If a location-year had two imputed points, the variance was multiplied by 4. The average estimates in each location-year were the input to an ST-GPR model. For this, we used a simple mixed

effects model, which was modelled in log space with nested location random effects. Subnational estimates were then further modelled by splitting the country-level estimates using current smoking prevalence.

Theoretical minimum-risk exposure level
The theoretical minimum-risk exposure level is 0.

Exposure among current and former smokers
Identical to GBD 2017, we estimated exposure among current smokers for two continuous indicators: cigarettes per smoker per day and pack-years. Pack-years incorporates aspects of both duration and amount. One pack-year represents the equivalent of smoking one pack of cigarettes (assuming a 20-cigarette pack) per day for one year. Since the pack-years indicator collapses duration and intensity into a single dimension, one pack-year of exposure can reflect smoking 40 cigarettes per day for six months or smoking 10 cigarettes per day for two years.

To produce these indicators, we simulated individual smoking histories based on distributions of age of initiation and amount smoked. We informed the simulation with cross-sectional survey data capturing these indicators, modelled at the mean level for all locations, years, ages, and sexes using ST-GPR. We rescaled estimates of cigarettes per smoker per day to an envelope of cigarette consumption based on supply-side data. We estimated pack-years of exposure by summing samples from age- and time-specific distributions of cigarettes per smoker for a birth cohort in order to capture both age trends and time trends and avoid the common assumption that the amount someone currently smokes is the amount they have smoked since they began smoking. All distributions were age-, sex-, and region- specific ensemble distributions, which were found to outperform any single distribution.

We estimated exposure among former smokers using years since cessation. We utilised ST-GPR to model mean age of cessation using cross-sectional survey data capturing age of cessation. Using these estimates, we generated ensemble distributions of years since cessation for every location, year, age group, and sex.

Relative risk
The same risk-outcome pairs from GBD 2017 were used: tuberculosis, lower respiratory tract infections, oesophageal cancer, stomach cancer, bladder cancer, liver cancer, laryngeal cancer, lung cancer, breast cancer, cervical cancer, colon and rectum cancer, lip and oral cancer, nasopharyngeal cancer, other pharyngeal cancer, pancreatic cancer, kidney cancer, leukaemia, ischaemic heart disease, ischaemic stroke, haemorrhagic stroke, subarachnoid haemorrhage, atrial fibrillation and flutter, aortic aneurysm, peripheral arterial disease, chronic obstructive pulmonary disease, other chronic respiratory diseases, asthma, peptic ulcer disease, gallbladder and biliary tract diseases, Alzheimer disease and other dementias, Parkinson disease (protective), multiple sclerosis, type-II diabetes, rheumatoid arthritis, low back pain, cataracts, macular degeneration, and fracture.

Dose-response risk curves
Input data for relative risks were nearly the same as in GBD 2017. The only addition was for chronic obstructive pulmonary disease, for which a few additional studies were included. We synthesised effect sizes by cigarettes per smoker per day, pack-years, and years since quitting from cohort and case-control studies to produce nonlinear dose-response curves using a Bayesian meta-regression model. For outcomes with significant differences in effect size by sex or age, we produced sex- or age-specific risk curves.

We estimated risk curves of former smokers compared to never smokers taking into account the rate of risk reduction among former smokers seen in the cohort and case-control studies, and the cumulative exposure among former smokers within each age, sex, location, and year group.

Population attributable fraction

As in GBD 2017, we estimated PAFs based on the following equation:

$$PAF = \frac{p(n) + p(f)\int \exp(x) * rr(x) + p(c)\int \exp(y) * rr(y) - 1}{p(n) + p(f)\int \exp(x) * rr(x) + p(c)\int \exp(y) * rr(y)}$$

where $pp(nn)$ is the prevalence of never smokers, $pp(ff)$ is the prevalence of former smokers, $pp(cc)$ is the prevalence of current smokers, $\exp(xx)$ is a distribution of years since quitting among former smokers, $rrrr(xx)$ is the relative risk for years since quitting, $\exp(yy)$ is a distribution of cigarettes per smoker per day or pack-years, and $rrrr(yy)$ is the relative risk for cigarettes per smoker per day or pack-years.

We used pack-years as the exposure definition for cancers and chronic respiratory diseases, and cigarettes per smoker per day for cardiovascular diseases and all other health outcomes.

References

For methodological summaries included on pages 96-100: ***Smoking***

1. Ng M, Freeman MK, Fleming TD, *et al*. Smoking Prevalence and Cigarette Consumption in 187 Countries, 1980–2012. *JAMA* 2014 Jan 8; **311**(2): 183–92.

Chewing tobacco

Flowchart

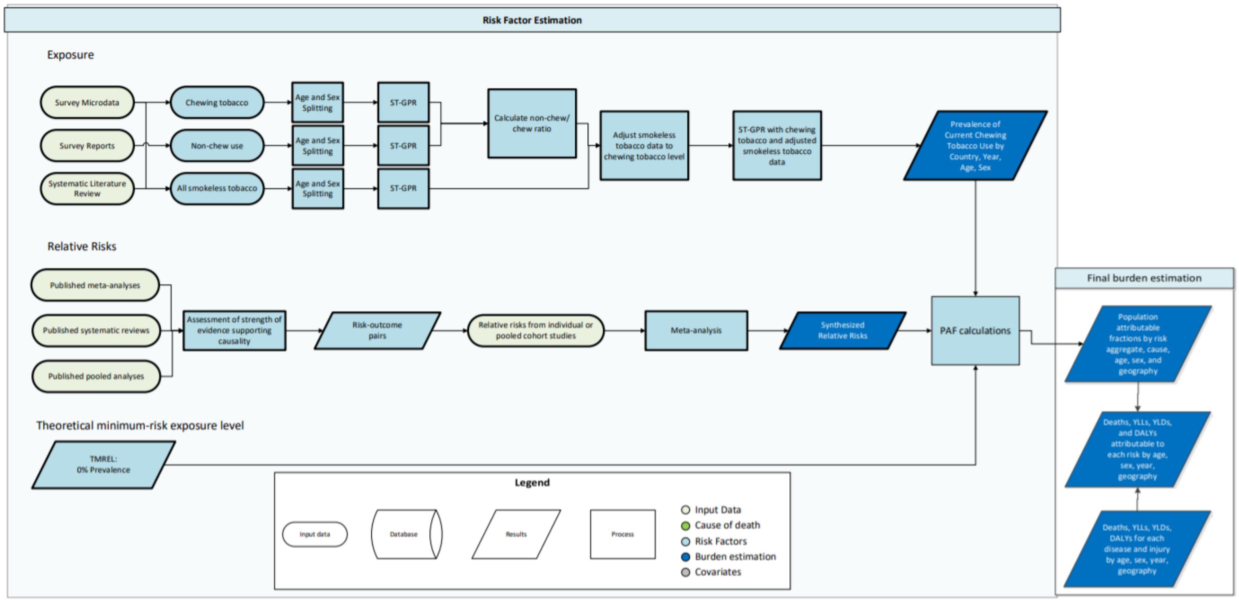

TMREL = Theoretical minimum-risk exposure level; ST-GPR = spaciotemporal Gaussian process regression; PAF = Population attributable fraction; YLL = Years of life lost; YLD = Years lived with disability; DALYs = Disability-adjusted life-years.

Input data and methodological summary

Exposure

Case definition

Current chewing tobacco use is defined as current use (use within the last 30 days where possible, or according to the closest definition available from the survey) of any frequency (any, daily, or less than daily). Chewing tobacco includes local products, such as betel quid with tobacco.

Input data

As in GBD 2017, we included sources that reported primary chewing tobacco, non-chew smokeless tobacco, and all smokeless tobacco use among respondents over age 10. To be eligible for inclusion, sources had to be representative for their level of estimation (ie, national sources needed to be nationally representative, subnational sources subnationally representative). We included only self-reported use data and excluded data from questions asking about others' tobacco use behaviours.

We extracted primary data from individual-level microdata and survey report tabulations on chewing tobacco, non-chew smokeless tobacco, and all smokeless tobacco use. We extracted data on current, former, and/or ever use as well as frequency of use (daily, occasional, and unspecified, which includes both daily and occasional smokers). Products that do not include tobacco, such as betel quid without tobacco, were excluded or estimated separately as part of the drug use risk factor, if applicable.

For microdata, we extracted relevant demographic information, including age, sex, location, and year, as well as survey metadata, including survey weights, primary sampling units, and strata. This information allowed us to tabulate individual-level data in the standard GBD five-year age-sex groups and produce accurate estimates of uncertainty. For survey report tabulations, we extracted data at the most granular age-sex group provided.

Age and sex splitting
We split data reported in broader age groups than the GBD five-year age groups or as both sexes combined by adapting the method reported in Ng and colleagues (http://jamanetwork.com/journals/jama/fullarticle/1812960) to split using a sex-geography-time-specific reference age pattern. We separated the data into two sets: a training dataset, with data already falling into GBD sex-specific five-year age groups, and a split dataset, which reported data in aggregated age or sex groups. We then used spatiotemporal Gaussian process regression (ST-GPR) to estimate sex-geography-time-specific age patterns using data in the training dataset. The estimated age patterns were then used to split each source in the split dataset.

The ST-GPR model used to estimate the age patterns for age-sex splitting used an age weight parameter value that minimises the effect of any age smoothing. This parameter choice allows the estimated age pattern to be driven by data, rather than being enforced by any smoothing parameters of the model. Because these age-sex-split datapoints will be incorporated in the final ST-GPR exposure model, we do not want to doubly enforce a modelled age pattern for a given sex-location-year on a given aggregate datapoint. We run three separate ST-GPR models for age-sex splitting – one for each smokeless tobacco category (chew, non-chew, and all smokeless).

Modelling strategy

Prevalence modelling
We used a ST-GPR to model chewing tobacco prevalence. Full details on the ST-GPR method are reported elsewhere in the Appendix. Briefly, the mean function input to GPR is a complete time series of estimates generated from a mixed effects hierarchical linear model plus weighted residuals smoothed across time, space, and age. The linear model formula for chewing tobacco, fit separately by sex using restricted maximum likelihood in R, is:

$$logit(p_{g,a,t}) = \beta_0 + \sum_{k=1}^{18} \beta_k I_{A[a]} + \alpha_s + \alpha_r + \alpha_g + \epsilon_{g,a,t}$$

Where $I_{A[\alpha]}$ is a dummy variable indicating specific age group A that the prevalence point $p_{g,a,t}$ captures, and α_s, α_r, and α_g are super-region, region, and geography random intercepts, respectively. The hyperparameters are the same as in GBD 2017.

We run three ST-GPR models for each prevalence category – one for each smokeless tobacco category (chew, non-chew, and all smokeless).

All smokeless tobacco prevalence adjustment
Using the 1000 draws from each of the prevalence ST-GPR models, we calculated 1000 draws of chewing tobacco prevalence divided by the sum of chewing tobacco and non-chewing tobacco prevalence

for each location, age group, sex, and year. The draws were unordered, as we did not want to enforce an assumption about the relationship between the levels of chewing tobacco and non-chewing tobacco prevalence.

The draws of the ratio of chewing to non-chewing tobacco were then multiplied by the draws from the all smokeless tobacco prevalence model to adjust the estimates to chewing tobacco prevalence. These were then averaged to get the mean estimate. The variance across the ratios was calculated for each location, year, age, and sex, and was added to the variance from the original all smokeless tobacco draws.

Final chewing tobacco prevalence model
To calculate the final chewing tobacco prevalence, we ran an additional ST-GPR model with both the original chewing tobacco data (post-age-sex splitting), as well as the adjusted data. These adjusted data add more information to the model – as surveys will often only ask about all smokeless tobacco consumption – while taking into consideration the uncertainty from the ratio calculation.

Theoretical minimum-risk exposure level
The theoretical minimum risk exposure level is that everyone in the population has been a lifelong nonuser of chewing tobacco.

Relative risk

As in GBD 2017, we included outcomes based on the strength of available evidence supporting a causal relationship. There was sufficient evidence to include Lip and oral cavity cancer and Oesophageal cancer as health outcomes caused by chewing tobacco use.

Relative risk estimates were derived from prospective cohort studies and population-based case-control studies. We used the same underlying effect size estimates from prospective cohort studies and population-based case-control studies as in GBD 2017. Briefly, we did not include hospital-based case control studies due to concerns over representativeness. We only included sources that adequately adjusted for major confounders, especially smoking status. Summary effect size estimates were calculated in R, using the 'metafor' package. We performed a random effects meta-analysis using the DerSimonian and Laird method, which does not assume a true effect size but considers each input study as selected from a random sample of all possible sets of studies for the outcome of interest. The random-effects method allows for more variation between the studies, and incorporates this variance into the estimation process. We used an inverse-variance weighting method to determine component study weights. We found significantly different relative risks for oral cancer for males and females, and estimated relative risks separately by sex for oral cancer alone.

<u>Secondhand smoke</u>

Flowchart

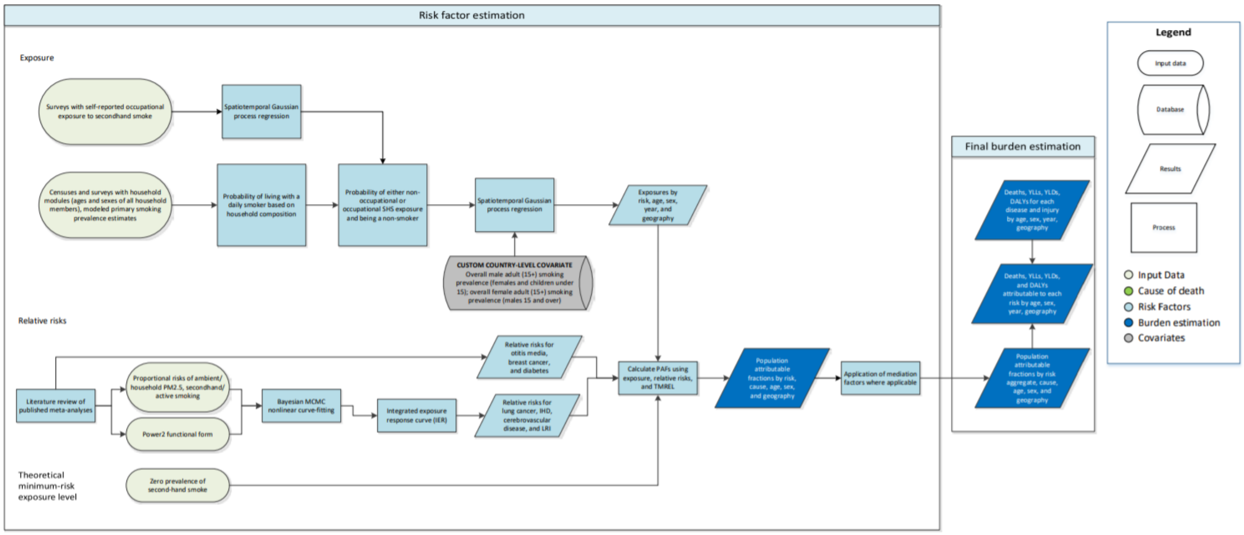

PM2.5 = particulate matter ≤ 2.5 micrometres; MCMC = Markov Chain Monte Carlo; SHS = second-hand smoke; IHD = ischaemic heart disease; LRI = lower respiratory infections; PAFs = Population attributable fraction; TMREL = Theoretical minimum-risk exposure level; YLL = Years of life lost; YLD = Years lived with disability; DALYs = Disability-adjusted life-years.

Input data and methodological summary

Exposure

Case definition

We define secondhand smoke exposure as current exposure to secondhand tobacco smoke at home, at work, or in other public places. We use household composition as a proxy for non-occupational secondhand smoke exposure and make the assumption that all persons living with a daily smoker are exposed to tobacco smoke. We use surveys to estimate the proportion of individuals exposed to secondhand smoke at work. We only consider non-smokers to be exposed to secondhand smoke. Non-smokers are defined as all persons who are not daily smokers. Ex-smokers and occasional smokers are considered non-smokers in this analysis. Exposure is evaluated for both children and adults.

Input data

To calculate the proportion of non-smokers who live with at least one smoker, we used unit record data on household composition, which included the ages and sexes of all persons living in the same household. Our sources included representative major survey series with a household composition module, including the Demographic Health Surveys (DHS), the Multiple Indicator Cluster Surveys (MICS), and the Living Standards Measurement Surveys (LSMS); and national and subnational censuses, which included those captured in the IPUMS project and identified using the Global Health Data Exchange catalog (GHDx).

To calculate the proportion of individuals exposed to secondhand smoke at work, by age and sex, we used cross-sectional surveys that ask respondents about self-reported occupational secondhand smoke exposure. Sources include the Global Adult Tobacco Surveys, Eurobarometer Surveys, and WHO STEPS Surveys. We identified sources using the GHDx.

No major changes have been introduced to data inputs since 2016. Given the nature of the data used in our models (microdata), no crosswalk for case definition adjustment or age- and sex-splitting processes were required. Estimates of daily smoking prevalence in each location were also used in our calculations, as described in the modelling strategy section below.

Modelling strategy

Identical to GBD 2017, we estimated the probability that each person is living with a smoker and is also a non-smoker themselves using set theory. First, household composition data were used at the individual level to capture the ages and sexes of each person in the household. Second, we analysed surveys with both household composition data and tobacco use questions and determined that the distribution of household size, mean age of the household members, and the age distribution were not significantly different between households with and without a self-reported smoker. Since we did not find that household composition varied between smokers and non-smokers, we then used the GBD 2019 primary daily smoking prevalence model to calculate the probability that each household member is a daily smoker. Next, we used the probability of the union of sets on each individual household member to calculate the overall probability that at least one of the other household members was a daily smoker. As in GBD 2017, we incorporated occupational exposure by modelling prevalence of current exposure to secondhand smoke at work, by age, sex, location, and year, using ST-GPR. In order to avoid double counting we calculated the probability that an individual is exposed through either non-occupational exposure or occupational exposure, given their age, sex, and household composition. Finally, we multiplied this probability of exposure by the probability that the individual is not a smoker themselves (ie, 1 minus primary daily smoking prevalence for that person's location, year, age, and sex). We then collapsed these individual-level probabilities to produce average probabilities of exposure by location, year, age, and sex.

These probabilities were modelled in the GBD ST-GPR framework, which generates exposure estimates from a mixed effects hierarchical linear model plus weighted residuals smoothed across time, space, and age. The linear model formula was fit separately by sex using restricted maximum likelihood in R.

We used the sex-specific overall daily smoking prevalence for adults (age 15 and older) as a country-level covariate in the model. The overall male adult daily smoking prevalence was used as the covariate for females of all ages and for males under age 15. The overall female adult daily smoking prevalence was used as the covariate for males age 15 and older.

All input datapoints from the probability calculation had a measure of uncertainty (variance and sample size) coming from the uncertainty of the primary smoking prevalence model and the sample size from the unit record data going into the modelling process. Geographical random effects were used in model fitting but were not used in prediction.

Theoretical minimum-risk exposure level

The theoretical minimum-risk exposure level for secondhand smoke is zero exposure among non-smokers, meaning that non-smokers would not live with any primary smokers.

Relative risks

The same risk-outcome pairs from GBD 2017 were used. For children ages 0-14, we estimated the burden of otitis media attributable to secondhand smoke exposure. For all ages we estimated then burden of

lower respiratory infections (LRI), and for adults greater than or equal to 25 years of age we estimated the burden of lung cancer, chronic obstructive pulmonary disease (COPD), ischaemic heart disease, and cerebrovascular disease attributable to secondhand smoke exposure, breast cancer, and type 2 diabetes.

For lung cancer, ischaemic heart disease, cerebrovascular disease, and LRI, we used country-specific relative risks created using integrated exposure response curves (IER) for $PM_{2.5}$ air pollution. IER curve calculation was updated with the GBD 2019 cigarettes per smoker estimates. The relative risks for otitis media,[1] breast cancer,[2] and diabetes[3] are derived from published meta-analyses and are the same as the ones used in the previous GBD cycle.

We used the standard GBD population attributable fraction (PAF) equation to estimate burden based on exposure and relative risks.

References
For methodological summaries included on pages 104-106: ***Secondhand smoke***

1. Jones LL, Hassanien A, Cook DG, Britton J, Leonardi-Bee J. Parental smoking and the risk of middle ear disease in children. *Arch Pediatr Adolesc Med* 2012; **166**: 18–27.

2. Macacu A, Autier P, Boniol M, Boyle P. Active and passive smoking and risk of breast cancer: a meta-analysis. *Breast Cancer Res Treat* 2015; **154**:213–224.

3. Zhu B, Wu X, Wang X, Zheng Q, Sun G. The association between passive smoking and type 2 diabetes: a meta-analysis. *Asia-Pacific Journal of Public Health* 2014; **26**:226-237.

<u>Alcohol use</u>

Flowchart

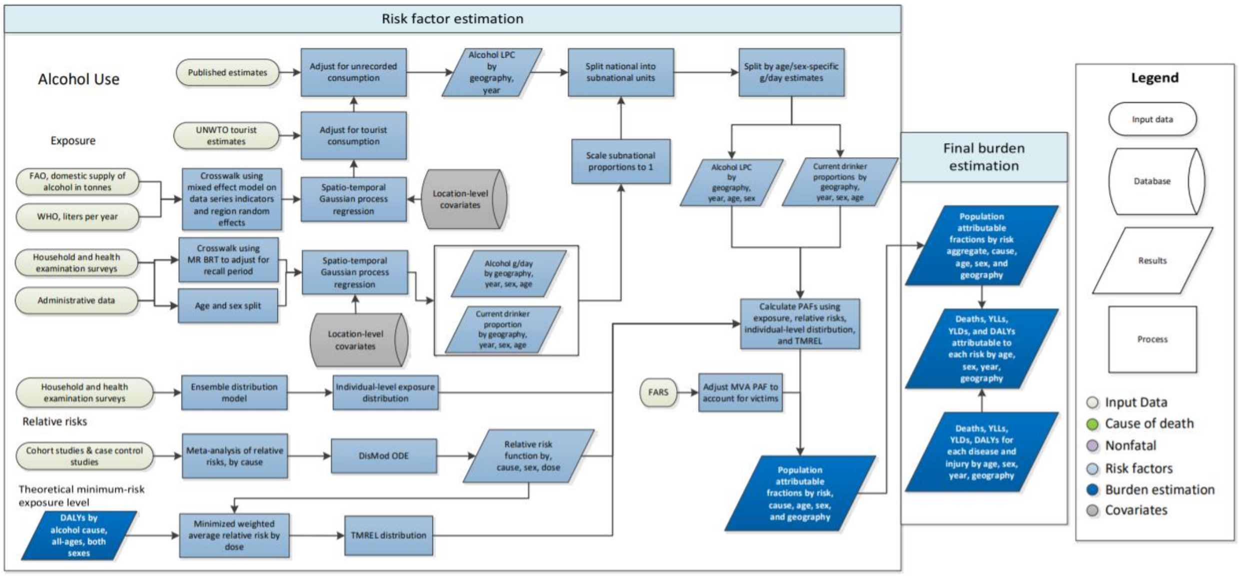

FAO = food and agriculture organization; WHO = World Health Organization; UWNTO = World Tourism Organization; MR BRT = a network meta-regression; DisMod ODE = the "engine" of DisMod-MR 2.1; TMREL = Theoretical minimum-risk exposure level; LPC = litres per capita; MVA = motor vehicle accidents; PAF = Population attributable fraction; FARS = Fatal Accident Reporting System; YLL = Years of life lost; YLD = Years lived with disability; DALYs = Disability-adjusted life-years.

Input data and methodological summary

Exposure

Case definition

We defined exposure as the grams per day of pure alcohol consumed among current drinkers. We constructed this exposure using the indicators outlined below:

1. Current drinkers, defined as the proportion of individuals who have consumed at least one alcoholic beverage (or some approximation) in a 12-month period.
2. Alcohol consumption (in grams per day), defined as grams of alcohol consumed by current drinkers, per day, over a 12-month period.
3. Alcohol litres per capita stock, defined in litres per capita of pure alcohol, over a 12-month period.

We also used three additional indicators to adjust alcohol exposure estimates to account for different types of bias:

1. Number of tourists within a location, defined as the total amount of visitors to a location within a 12-month period.
2. Tourists' duration of stay, defined as the number of days resided in a hosting country.

Unrecorded alcohol stock, defined as a percentage of the total alcohol stock produced outside established markets.

Input data

A systematic review of the literature was performed to extract data on our primary indicators. The Global Health Exchange (GHDx), IHME's online database of health-related data, was searched for population survey data containing participant-level information from which we could formulate the required alcohol use indicators on current drinkers and alcohol consumption. Data sources were included if they captured a sample representative of the geographical location under study. We documented relevant survey variables from each data source in a spreadsheet and extracted using STATA 13.1 and R 3.3. A total of 6172 potential data sources were available in the GHDx, of which 5091 have been screened and 1125 accepted.

Estimates of current drinking prevalence were split by age and sex where necessary. First, studies that reported prevalence for both sexes were split using a region-specific sex ratio estimated using MR-BRT. Second, where studies reported estimates across non-GBD age groups, these were split into standard five-year age groups using the global age pattern estimated by ST-GPR.

MR-BRT sex splitting adjustment factors for current drinking

Data input	Gamma	Beta coefficient, log (95% CI)	Adjustment factor*
Female: Male	0	-0.16 (-0.17, -0.14)	0.85
Age < 50	0	0.06 (0.06, 0.06)	1.07
East Asia	0.36	-1.02 (-1.74, -0.29)	0.36
Southeast Asia	0.64	-1.06 (-2.34, 0.22)	0.35
Central Asia	0.41	-0.35 (-1.16, 0.46)	0.70
Central Europe	0.18	-0.21 (-0.58, 0.14)	0.80
Eastern Europe	0.10	-0.07 (-0.28, 0.14)	0.93
High-income Asia Pacific	1.27	-1.11 (-4.90, 2.68)	0.33
Western Europe	0.08	0.03 (-0.14, 0.20)	1.03
Southern Latin America	1.26	-0.67 (-4.18, 2.84)	0.51
High-income North America	0.09	-0.07 (-0.26, 0.11)	0.93
Caribbean	0.25	-0.52 (-1.02, -0.03)	0.59
Andean Latin America	0.76	-0.16 (-1.66, 1.34)	0.85
Central Latin America	0.30	-0.52 (-1.12, 0.08)	0.59
Tropical Latin America	0.08	-0.61 (-0.79, -0.44)	0.54
North Africa and Middle East	1.21	-1.44 (-3.91, 1.03)	0.24
South Asia	0.71	-1.17 (-2.57, 0.23)	0.31
Eastern sub-Saharan Africa	0.28	-0.53 (-1.10, 0.03)	0.58
Southern sub-Saharan Africa	0.20	-0.16 (-0.56, 0.23)	0.85
Western sub-Saharan Africa	0.32	-0.19 (-0.83, 0.45)	0.83
Oceania	0.94	-0.54 (-2.42, 1.34)	0.58

**Adjustment factor is the transformed beta coefficient in normal space and can be interpreted as the factor by which the alternative case definition is adjusted to reflect the ratio by which both-sex data points were split.*

To allow for the inclusion of data that did not meet our reference definition for current drinking, two crosswalks were performed using MR-BRT. The first crosswalk converted estimates of one-month drinking prevalence to what they would be if data represented estimates of 12-month drinking prevalence. This crosswalk incorporated two binary covariates: male and age ≥ 50. The second crosswalk converted estimates of one-week drinking prevalence to 12-month drinking prevalence. This crosswalk incorporated

age < 20 and male as covariates. The covariates utilised in both crosswalks were included as both x and z covariates. A uniform prior of 0 was set as the upper bound for the beta coefficients to enforce the logical constraint that one-month and one-week prevalence could not be greater than 12-month prevalence.

MR-BRT crosswalk adjustment factors for alcohol use current drinking model

Data input	Reference or alternative case definition	Gamma	Beta coefficient, logit (95% CI)
12-month prevalence	Ref	---	---
1-month prevalence	Alt	0.22	-0.60 (-1.05, -0.16)
Age ≥ 50		0.13	0.16 (-0.10, 0.43)
Male		0.29	0.01 (-0.57, 0.59)
1-week prevalence	Alt	0.46	-1.51 (-2.42, -0.59)
Age < 20		0.47	-0.29 (-1.34, 0.76)
Male		0.00	0.38 (0.15, 0.60)

The methods for modelling supply-side-level data were changed substantially from those used in GBD 2017. The raw data are domestic supply (WHO GISAH; FAO) and retail supply (Euromonitor) of litres of pure ethanol consumed. Domestic supply is calculated as the sum of production and imports, subtracting exports. The WHO and FAO sources were combined, so that FAO data were only used if there were no data available for that location-year from WHO. This was done because the WHO source takes into consideration FAO values when available. Since the WHO data are given in more granular alcohol types, the following adjustments were made:

$$LPC\ Pure\ Ethanol = 0.13 * \left(\frac{Wine}{0.973}\right)$$

$$LPC\ Pure\ Ethanol = 0.05 * \left(\frac{Beer}{0.989}\right)$$

$$LPC\ Pure\ Ethanol = 0.4 * \left(\frac{Spirits}{0.91}\right)$$

Three outliering strategies are used to omit implausible datapoints and data that created implausible model fluctuations. First, estimates from the current drinking model are used to calculate the grams of alcohol consumed per drinker per day. A point is outliered if the grams of pure ethanol per drinker per day for a given source-location-year is greater than 100 (approximately ten drinks). These thresholds were chosen by using expert knowledge about reasonable consumption levels. In the second round of outliering, the mean liters per capita value over a ten-year window is calculated. If a point is over 70% of that mean value away from the mean value, it is outliered. The 70% limit was chosen using histograms of these distances. Additionally, some manual outliering is performed to account for edge cases. Finally, data smoothing is performed by taking a three-year rolling mean over each location-year.

Next, an imputation to fill in missing years is performed for all series to remove compositional bias from our final estimates. Since the data from our main sources cover different time periods, by imputing a complete time series for each data series, we reduce the probability that compositional bias of the sources

is leading to biased final estimates. To impute the missing years for each series, we model the log ratio of each pair of sources as a function of an intercept and nested random effects on superregion, region, and location. The appropriate predicted ratio is multiplied by the source that we do have, which generates an estimated value for the missing source. For some locations where there was limited overlap between series, the predicted ratio did not make sense, and a regional ratio was used.

Finally, variance was calculated both across series (within a location-year) as well as across years (within a location-source). Additionally, if a location-year had one imputed point, the variance was multiplied by 2. If a location-year had two imputed points, the variance was multiplied by 4. The average estimates in each location-year were the input to an ST-GPR model. This uses a mixed-effects model modelled in log space with nested location random effects.

We obtained data on the number of tourists and their duration of stay from the UNWTO.[3] We applied a crosswalk across different tourist categories, similar to the one used for the litres per capita data, to arrive at a consistent definition (ie, visitors to a country).

We obtained estimates on unrecorded alcohol stock from data available in WHO GISAH database,[2] consisting of 189 locations. For locations with no data available, the national or regional average was used.

For relative risks, in GBD 2016 we performed a systematic literature review of all cohort and case-control studies reporting a relative risk, hazard ratio, or odds ratio for any risk-outcome pairs studied in GBD 2016. Studies were included if they reported a categorical or continuous dose for alcohol consumption, as well as uncertainty measures for their outcomes, and the population under study was representative.

Modelling strategy

While population-based surveys provide accurate estimates of the prevalence of current drinkers, they typically underestimate real alcohol consumption levels.[10-12] As a result, we considered the litre per capita input to be a better estimate of overall volume of consumption. Per capita consumption, however, does not provide age- and sex-specific consumption estimates needed to compute alcohol attributable burden of disease. Therefore, we use the age-sex pattern of consumption among drinkers modelled from the population survey data and the overall volume of consumption from FAO, GISAH, and Euromonitor to determine the total amount of alcohol consumed within a location. In the paragraphs below, we outline how we estimated each primary input in the alcohol exposure model, as well as how we combined these inputs to arrive at our final estimate of grams per day of pure alcohol. We estimated all models below using 1000 draws.

For data obtained through surveys, we used spatiotemporal Gaussian process regression (ST-GPR) to construct estimates for each location/year/age/sex. We chose to use ST-GPR due to its ability to leverage information across the nearby locations or time periods. We also modelled the alcohol litres per capita (LPC) data, as well as the total number of tourists, using ST-GPR.

Given the heterogeneous nature of the estimates on unrecorded consumption, as well as the wide variation across countries and time periods, we took 1000 draws from the uniform distribution of the lowest and highest estimates available for a given country. We did this to incorporate the diffuse uncertainty within the unrecorded estimates reported. We used these 1000 draws in the equation below.

We adjusted the alcohol LPC for unrecorded consumption using the following equation:

$$Alcohol\ LPC = \frac{Alcohol\ LPC}{(1 - \%\ Unrecorded)}$$

We then adjusted the estimates for alcohol LPC for tourist consumption by adding in the per capita rate of consumption abroad and subtracting the per capita rate of tourist consumption domestically.

$$Alcohol\ LPC_d = Unadjusted\ Alcohol\ LPC_d + Alcohol\ LPC_{Domestic\ consumption\ abroad} - Alcohol\ LPC_{Tourist\ consumption\ domestically}$$

$$Alcohol\ LPC_i = \frac{\sum_l Tourist\ Population_l * Proportion\ of\ tourists_{i,l} * Unadjusted\ Alcohol\ LPC_l * \frac{Average\ length\ of\ stay_{i,l}}{365} *}{Population_d}$$

where:

l is the set of all locations, i is either Domestic consumption abroad *or* Tourist consumption domestically, *and d is a domestic location.*

After adjusting alcohol LPC by tourist consumption and unrecorded consumption for all location/years reported, sex-specific and age-specific estimates were generated by incorporating estimates modelled in ST-GPR for percentage of current drinkers within a location/year/sex/age, as well as consumption trends modelled in the ST-GPR grams per day model. We do this by first calculating the proportion of total consumption for a given location/year by age and sex, using the estimates of alcohol consumed per day, the population size, and the percentage of current drinkers. We then multiply this proportion of total stock for a given location/year/sex/age by the total stock for a given location/year to calculate the consumption in terms of litres per capita for a given location/year/sex/age. We then convert these estimates to be in terms of grams/per day. The following equations describe these calculations:

$$Proportion\ of\ total\ consumption_{l,y,s,a} = \frac{Alcohol\ \ g/day_{l,y,s,a} * Population_{l,y,s,a} * \%\ Current\ drinkers_{l,y,s,a}}{\sum_{s,a} Alcohol\ \ g/day_{l,y,s,a} * Population_{l,y,s,a} * \%\ Current\ drinkers_{l,y,s,a}}$$

$$Alcohol\ LPC_{l,y,s,a} = \frac{Alcohol\ LPC_{l,y} * Population_{l,y} * Proportion\ of\ total\ consumption_{l,y,s,a}}{\%\ Current\ drinkers_{l,y,s,a} * Population_{l,y,s,a}}$$

$$Alcohol\ g/day_{l,y,s,a} = Alcohol\ LPC_{l,y,s,a} * \frac{1000}{365}$$

where:

l is a location, y is a year, s is a sex, and a is an age group.

We then used the gamma distribution to estimate individual-level variation within location, year, sex, age drinking populations, following the recommendations of other published alcohol studies.[7,8] We chose parameters of the gamma distribution based on the mean and standard deviation of the 1,000 draws of alcohol g/day exposure for a given population. Standard deviation was calculated using the following formula.[15] We tested several alternative models using our data and found this model performed best.

$$standard\ deviation = mean * (0.087 * female + 1.171)$$

Theoretical minimum-risk exposure level

We calculated TMREL by first calculating the overall risk attributable to alcohol. We did this by weighting each relative risk curve by the share of overall DALYs for a given cause. We then took the minimum of this overall-risk curve as the TMREL of alcohol use. More formally,

$$TMREL = argmin\ average\ overall\ risk_{\omega}(g/day)$$

$$Average\ overall\ risk_{\omega}(g/day) = \sum_{i}^{\omega} RR_i(g/day) * \frac{DALY_i}{\sum_i^{\omega} DALY_i}$$

where:

ω is the set of causes associated with alcohol, i is a given cause from that set, DALY is the global DALY rate in 2010, and RR is the dose response curve for a given cause and exposure level in grams per day.

In other words, we chose TMREL as being the exposure that minimises your risk of suffering burden from any given cause related to alcohol. We weight the risk for a particular cause in our aggregation by the proportion of DALYs due to that cause (eg, since more observed people die from ischaemic heart disease [IHD], we weight the risk for IHD more in the above calculation of average risk compared to, say, diabetes, even if both have the same relative risk for a given level of consumption).

Relative risks

We used the studies identified through the systematic review to calculate a dose-response, modelled using DisMod ODE. We chose DisMod ODE rather than a conventional mixed effects meta-regression because of its ability to estimate nonparametric splines over doses (ie, for most alcohol causes, there is a non linear relationship with different doses) and incorporate heterogeneous doses through doseintegration (ie, most studies report doses categorically in wide ranges. DisMod ODE estimates specific doses when categories overlap across studies, through an integration step.). We used the results of the meta-regression to estimate a non-parametric curve for all doses between zero and 150 g/day and their corresponding relative risks. For all causes, we assumed the relative risk was the same for all ages and sexes, with the exception of ischaemic heart disease, ischaemic stroke, haemorrhagic stroke, and diabetes, which we estimated by sex.

For outcomes that are by definition caused by alcohol, such as liver cancer or cirrhosis due to alcohol use, PAFs are set to 1. PAFs for cirrhosis due to all causes that are in excess of the proportion of all cirrhosis burden due to alcohol are proportionally redistributed over cirrhosis due to hepatitis B, hepatitis C, and other causes.

Regarding injuries outcomes, we constructed relative risks based on chronic exposure to alcohol rather than acute exposure immediately preceding injury, which has a weaker relationship to the outcome, though still significant.[15,16,18-21] We decided to use chronic exposure given the lack of available data on acute exposure, as well as the lack of cohort studies using acute exposure as a metric. Further, using chronic exposure allowed us to construct relative risks curves for unintentional injuries, interpersonal violence, motor vehicle accidents, and self-harm using the same method as reported above.

In the case of motor vehicle accidents, we adjusted the PAF to account for victims of drunk drivers who are involved in accidents. Using data from the Fatality Analysis Reporting System in the US,[17] we calculated the average number of fatalities in a car crash involving alcohol, as well as the percentage of those fatalities distributed by age and sex. We aggregated FARS data across the years 1985–2015, given there was little variation in the data temporally and the number of cases in old age groups had too much variance when constructing estimates by year. To adjust PAFs, we multiplied attributable deaths by the average number of fatalities from FARS and redistributed the PAF among each population, based on the probability of being a victim to a certain drunk driver by age and sex, based on the FARS data. The following equation describes this process:

$$Adjusted\ PAF_i = \frac{\sum_d PAF_d * DALY_d * Avg\ Fatalities_d * \ P(i\ is\ a\ victim)_d}{DALY_i}$$

where:

i is a population by location, year, age, sex and d is the set of all age and sex exposed groups within that location and year.

Population attributable fraction

For all causes, we defined PAF as:

$$PAF(x) = \frac{P_A + \int_0^{150} P(x) * RR_C(x)\,dx - 1}{P_A + \int_0^{150} P(x) * RR_C(x)\,dx} \qquad P(x) = P_C * \Gamma(\boldsymbol{p})$$

where:

P_c is the prevalence of current drinkers, P_a is the prevalence of abstainers, $RR_c(x)$ is the relative risk function for current drinkers and ***p*** *are parameters determined by the mean and sd of exposure*

We performed the above equation for 1000 draws of the exposure and relative risk models. We then used the estimated PAF draws to calculate YLL, YLDs, and DALYs, as per the other risk factors.

References

For methodological summaries included on pages 107-113: ***Alcohol use***

1. Food and Agriculture Organization of the United Nations (FAO). FAOSTAT Food Balance Sheets, October 2014. Rome, Italy: Food and Agriculture Organization of the United Nations (FAO).

2. World Health Organization (WHO). WHO Global Health Observatory - Recorded adult per capita alcohol consumption, Total per country. Geneva, Switzerland: World Health Organization (WHO).

3. UN World Tourism Organization (UNWTO). UN World Tourism Organization Compendium of Tourism Statistics 2015 [Electronic]. Madrid, Spain: UN World Tourism Organization (UNWTO), 2016.

4. Ramstedt M. How much alcohol do you buy? A comparison of self-reported alcohol purchases with actual sales. *Addiction* 105.4 (2010): 649-654.

5. Stockwell T, Donath S, Cooper-Stanbury M, Chikritzhs T, Catalano P, Mateo C. Under-reporting of alcohol consumption in household surveys: a comparison of quantity–frequency, graduated–frequency and recent recall. *Addiction* 99.8 (2004): 1024-1033.

6. Kerr WC, and Greenfield TK. Distribution of alcohol consumption and expenditures and the impact of improved measurement on coverage of alcohol sales in the 2000 National Alcohol Survey. *Alcoholism: Clinical and Experimental Research* 31.10 (2007): 1714- 1722.

7. Taylor B, Irving HM, Kanteres F, *et al*. The more you drink, the harder you fall: a systematic review and metaanalysis of how acute alcohol consumption and injury or collision risk increase together. *Drug and alcohol dependence* 110.1 (2010): 108-116.

8. Vinson DC, Guilherme B, and Cheryl JC. The risk of intentional injury with acute and chronic alcohol exposures: a case-control and case-crossover study. *Journal of studies on alcohol* 64.3 (2003): 350-357.

9. Vinson DC, Maclure M, Reidinger C, Smith GS, *et al*. A population-based case-crossover and case-control study of alcohol and the risk of injury. *Journal of studies on alcohol* 64.3 (2003): 358-366.

10. Fatal Accident Reporting System (FARS). National Highway Traffic Safety Administration, National Center for Statistics and Analysis Data Reporting and Information Division (NVS-424); 1985, 1990, 1995, 2000, 2005, 2010, 2015.

11. Chen L-H, Baker SP, and Li G. Drinking history and risk of fatal injury: comparison among specific injury causes. *Accident Analysis & Prevention* 37.2 (2005): 245-251.

12. Bell NS, Amoroso PJ, Yore MM, Smith GS, Jones BH. Self-reported risk-taking behaviors and hospitalization for motor vehicle injury among active duty army personnel. *American journal of preventive medicine* 18.3 (2000): 85-95.

13. Margolis KL, Kerani RP, McGovern P, *et al*. Risk factors for motor vehicle crashes in older women. *The Journals of Gerontology Series A: Biological Sciences and Medical Sciences* 57.3 (2002): M186-M191.

14. Sorock GS, Chen L-H, Gonzalgo SR, Baker SP. Alcohol-drinking history and fatal injury in older adults. *Alcohol* 40.3 (2006): 193-199.

15. Kehoe T, Gmel G, Shield KD, Gmel G, Rehm J. Determining the best population-level alcohol consumption model and its impact on estimates of alcohol-attributable harms. *Population Health Metrics* 10 6. (2012).

Drug use

Flowchart

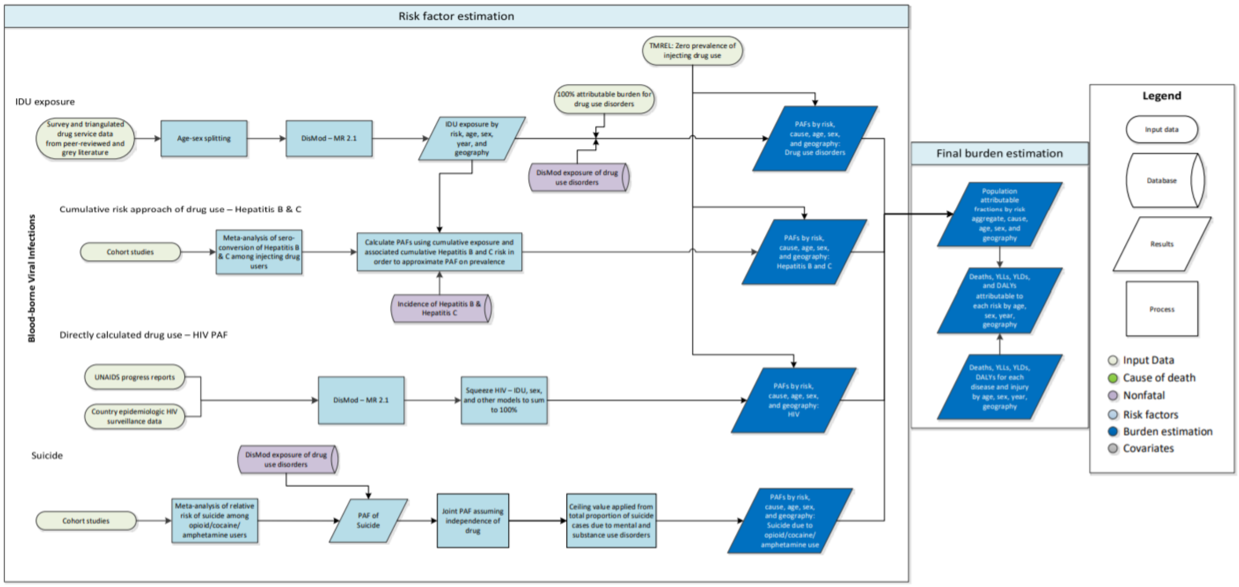

DisMod – MR 2.1 = disease model - Bayesian meta-regression; IDU = injection drug use; TMREL = Theoretical minimum-risk exposure level; PAF= Population attributable fraction; UNAIDS = Joint United Nations Programme on HIV/AIDS; HIV = human immunodeficiency virus; YLL = Years of life lost; YLD = Years lived with disability; DALYs = Disability-adjusted life-years.

Input data and methodological summary

Exposure

Case definition

The drug use risk factor includes four dimensions of exposure. First, we include 100% attribution of drug use disorder estimates. Second, estimates of prevalence of opioid, amphetamine, and cocaine use disorder are used as exposures for risk of suicide. These drug use disorders are defined based on DSM or ICD diagnostic criteria. Third, instead of starting with an exposure model to estimate the proportion of HIV cases due to injection drug use (IDU), we model the PAF directly, alongside proportion of HIV cases due to sexual transmission and other routes of transmission, which mainly includes blood transfusions. Finally, prevalence of injection drug use is used to model risk of Hepatitis B and C viruses (HBV and HCV, respectively). Injecting drug users are at high risk of bloodborne infections due to the use of shared needles and injection equipment. Injecting drug use is defined as current injection drug use among individuals aged 15 to 64. The theoretical minimum-risk exposure level (TMREL) for drug use is defined as zero exposure to drug use.

To estimate the burden of HIV cases attributable to IDU, we extracted data on the proportion of notified HIV cases by transmission route – sexual intercourse, injecting drug use, and other – from a number of agencies that conduct surveillance of HIV across the globe.[1-8]

The prevalence of current injecting drug use was estimated using data from a multistage process of systematic review. It involved multiple stages of peer and expert review, including review by the Reference Group to the UN on HIV and injecting drug use,[9] with searches of the peer-reviewed literature

in addition to an extensive review of online grey literature databases in the drug and alcohol and HIV fields.

In order to generate a pooled incidence rate/absolute relative risk for viral hepatitis among people who inject drugs, we conducted a meta-analysis of longitudinal epidemiological studies that reported a hepatitis B or hepatitis C incidence rate among persons who inject drugs.[10-25] We calculated confidence intervals for the incidence rate (where no CI was reported) from a Poisson distribution around the number of cases.

We excluded studies that focused on non-representative subgroups, such as recent injectors or adolescents, because hepatitis incidence is far higher in those groups than for all people who inject drugs (eg, Larney and colleagues).[26] We did not vary incidence among active injectors according to the availability of blood borne virus-prevention strategies (eg, NSPs, opioid substitution therapy) because too few studies have examined different levels of incidence according to variable coverage, and we were not able to estimate coverage by country over time. In any case, in most countries, effective coverage of virus-prevention strategies remains low among people who inject drugs.[27]

Inputs to the model also include estimates of the incidence of hepatitis B and hepatitis C, coming from estimation of non-fatal health outcomes in GBD. Full details on the inputs and modelling process to produce these estimates are available in the disease-specific appendices in the GBD 2019 diseases and injuries manuscript.

Modelling strategy

Burden of HIV attributable to injecting drug use

We estimated the proportion of HIV cases attributable to three transmission categories (sex, IDU, and other) for all country-time periods using DisMod-MR 2.1. In previous rounds, data for estimating the proportion of HIV cases attributable to IDU were age-split using the age pattern of the IDU exposure model and sex-split in DisMod. In GBD 2019, these data were age- and sex-split using the estimated IDU exposure age-sex pattern, resulting in increases in the proportion of HIV due to IDU among men and decreases among women. We scaled the proportions from each of the three transmission models (sex, IDU, and other) to ensure that they fit the total HIV transmission envelope by country, year, age and sex. Scaled estimates are used as direct population attributable fractions, meaning that the proportion coming from the model is the proportion of HIV deaths or DALYs attributable to IDU.

Burden of hepatitis B and hepatitis C attributable to injecting drug use

To estimate the relative contribution of IDU to hepatitis B and C disease burden at the country, regional, and global level, we used a cohort method. We recalibrated individuals according to history of injecting drug use and their accumulated risk of incident hepatitis B and C due to IDU. We made use of data on prevalence of current injecting drug use, pooled in DisMod-MR 2.1; a meta-analysis of incidence rates of hepatitis B and hepatitis C among people who inject drugs; and estimates of population-level incidence of hepatitis B and C between 1990 and 2019. We used back-extrapolations to estimate incidence before 1990.

To estimate the lifetime risk of being infected with hepatitis B or C, we undertook a cohort analysis for each country, year, age, and sex category and estimated the probability of an individual having been infected in each preceding year. One of the main inputs to this cohort method was the probability of having injected drugs in a specific age cohort in a given calendar year. For example, for a cohort of 40-year-olds in 2015, the relevant probability in 2005 is the estimated prevalence of injecting drug use among 30-year-olds.

DisMod-MR 2.1 was used to estimate the prevalence of injecting drug use with year as a covariate to estimate the trends over time. DisMod makes an average estimate of the change in drug use over the time period 1990–2019, and we took draws from a normal distribution of the coefficient to project IDU prevalence backward in time to 1960 from baseline level in 1990 (assuming there was little injecting drug use before the 1960s). In GBD 2019, prevalence of IDU was estimated as a single parameter prevalence model in DisMod, as opposed to a full compartmental model, because factoring in cause specific mortality resulted in underestimating prevalence in certain locations, particularly in the north Africa and Middle East and south Asia super-regions.

Theoretical minimum-risk exposure level

The theoretical minimum-risk exposure level is defined as zero exposure to drug use.

Relative risk

We used a pooled absolute risk of hepatitis C and hepatitis B among those who have ever used injecting drugs. Input data for this pooled absolute risk are described above, and there were no methodological or data changes to this parameter in GBD 2019.

In GBD 2019, we updated the relative risk of suicide among those with substance use disorders to include new studies. Six new studies were included in a meta-analysis on the relative risk of suicide due to opioid, amphetamine, or cocaine use disorders.[28-54] The meta-analysis was conducted using MR-BRT.

Compared to GBD 2017, the new data added resulted in a decrease in the relative risks and, therefore, burden of suicide due to the use of opioids, amphetamines, and cocaine.

References

For methodological summaries included on pages 115-117: ***Drug use***

1. European Centre for Disease Prevention. HIV/AIDS surveillance in Europe 2014 Solna, Sweden. http://ecdc.europa.eu/en/publications/surveillance_reports/HIV_STI_and_blood_borne_viruses/Pages/HIV_STI_and_blood_borne_viruses.aspx: ECDC, 2014.

2. Family Health International, Bureau of AIDS TB and STIs Department of Disease Control. The Asian Epidemic Model (AEM) Projections for HIV/AIDS in Thailand:2005-2025. Bangkok: Family Health International (FHI) and Bureau of AIDS, TB and STIs, Department of Disease Control, Ministry of Public Health, Thailand, 2008.

3. Kirby Institute. 2015 Annual Surveillance Report of HIV, viral hepatitis, STIs. Sydney, New South Wales. https://kirby.unsw.edu.au/surveillance/2015-annual-surveillance-report-hiv-viral-hepatitisstis: Kirby Institute, UNSW Australia, 2015.

4. Kirby Institute. Australian NSP survey national data report 2015. Sydney, New South Wales: Kirby Institute, University of New South Wales, 2015.

5. Country reports for Global AIDS Response Progress Reporting [Internet]. UNAIDS. 2014.

6. UNAIDS. UNAIDS Country reports. Geneva: Joint United Nations Programme on HIV/AIDS. http://www.unaids.org/en/regionscountries/countries, 2015.

7. United States Center for Disease Control and Prevention. HIV/AIDS Statistics. Atlanta, Georgia: US CDC. http://www.cdc.gov/hiv/statistics/index.html, 2015.

8. Gouws E, White PJ, Stover J, Brown T. Short term estimates of adult HIV incidence by mode of transmission: Kenya and Thailand as examples. *Sex Transm Infect.* 2006; **82** Suppl 3:iii51-5.

9. Mathers BM, Degenhardt L, Phillips B, *et al.* Global epidemiology of injecting drug use and HIV among people who inject drugs: a systematic review. Lancet. 2008; **372**(9651): 1733-45.

10. Jackson JB, Wei L, Liping F, *et al.* Prevalence and Seroincidence of Hepatitis B and Hepatitis C Infection in High Risk People Who Inject Drugs in China and Thailand. *Hepatitis research and treatment.* 2014; 2014.

11. Månsson A-S, Moestrup T, Nordenfelt E, Widell A. Continued transmission of hepatitis B and C viruses, but no transmission of human immunodeficiency virus among intravenous drug users participating in a syringe/needle exchange program. Scandinavian Journal of Infectious Diseases. 2000;32(3):253-8.

12. Blomé MA, Björkman P, Flamholc L, Jacobsson H, Molnegren V, Widell A. Minimal transmission of HIV despite persistently high transmission of hepatitis C virus in a Swedish needle exchange program. *Journal of viral hepatitis*. 2011; **18**(12):831-9.

13. Hagan H, McGough JP, Thiede H, Weiss NS, Hopkins S, Alexander ER. Syringe exchange and risk of infection with hepatitis B and C viruses. *American Journal of Epidemiology*. 1999; **149**(3):203-13.

14. Crofts N, Aitken CK. Incidence of bloodborne virus infection and risk behaviours in a cohort of injecting drug users in Victoria in 1990-1995. *Medical Journal of Australia*. 1997; **167**(1): 17-20.

15. Roy K, Goldberg D, Taylor A, *et al*. A method to detect the incidence of hepatitis C infection among injecting drug users in Glasgow 1993–98. *Journal of Infection* 2001; **43**(3): 200-5. 213

16. Abou-Saleh M, Davis P, Rice P, *et al*. The effectiveness of behavioural interventions in the primary prevention of hepatitis C amongst injecting drug users: a randomised controlled trial and lessons learned. *Harm Reduction Journal*. 2008; **5**(1):1.

17. Turner KM, Hutchinson S, Vickerman P, *et al*. The impact of needle and syringe provision and opiate substitution therapy on the incidence of hepatitis C virus in injecting drug users: pooling of UK evidence. *Addiction* 2011; **106**(11): 1978-88.

18. Grebely J, Lima VD, Marshall BD, *et al*. Declining incidence of hepatitis C virus infection among people who inject drugs in a Canadian setting, 1996-2012. *PloS One* 2014; **9**(6): e97726.

19. Foley S, Abou-Saleh MT. Risk behaviors and transmission of hepatitis C in injecting drug users. Addictive Disorders & Their Treatment. 2009;8(1):13-21.

20. Craine N, Hickman M, Parry J, *et al*. Incidence of hepatitis C in drug injectors: the role of homelessness, opiate substitution treatment, equipment sharing, and community size. *Epidemiology and Infection* 2009; **137**(09): 1255-65.

21. Villano SA, Vlahov D, Nelson KE, Lyles CM, Cohn S, Thomas DL. Incidence and risk factors for hepatitis C among injection drug users in Baltimore, Maryland. *Journal of Clinical Microbiology* 1997; 35(12): 3274-7.

22. Maher L, Jalaludin B, Chant KG, *et al*. Incidence and risk factors for hepatitis C seroconversion in injecting drug users in Australia. *Addiction* 2006; **101**(10): 1499- 508.

23. Lucidarme D, Bruandet A, Ilef D, *et al*. Incidence and risk factors of HCV and HIV infections in a cohort of intravenous drug users in the North and East of France. *Epidemiology and Infection* 2004; **132**(04): 699-708.

24. Partanen A, Malin K, Perälä R, *et al*. Riski-tutkimus 2000- 2003. Pistämällä huumeita käyttävien seurantatutkimus. A-Klinikkasäätiön Raporttisarja nro 52. Helsinki: A-Klinikkasäätiön, 2006.

25. Van Den Berg C, Smit C, Van Brussel G, Coutinho R, Prins M. Full participation in harm reduction programmes is associated with decreased risk for human immunodeficiency virus and hepatitis C virus: evidence from the Amsterdam Cohort Studies among drug users. *Addiction* 2007; **102**(9): 1454-62.

26. Larney S, Kopinski H, Beckwith CG, *et al*. Incidence and prevalence of hepatitis C in prisons and other closed settings: results of a systematic review and metaanalysis. *Hepatology* 2013; **58**(4): 1215-24.

27. Degenhardt L, Mathers B, Vickerman P, Rhodes T, Latkin C, Hickman M. Prevention of HIV infection for people who inject drugs: Why individual, structural, and combination approaches are needed. *The Lancet* 2010; **376**: 285-301.

28. Pavarin RM. Cocaine consumption and death risk: a follow-up study on 347 cocaine addicts in the metropolitan area of Bologna. *Ann Ist Super Sanita* 2008; **44**(1): 91-8.

29. Tyndall MW, Craib KJ, Currie S, Li K, O'Shaughnessy MV, Schechter MT. Impact of HIV infection on mortality in a cohort of injection drug users. *J Acquir Immune Defic Syndr* 2001; **28**(4): 351-7.

30. Miller CL, Kerr T, Strathdee SA, Li K, Wood E. Factors associated with premature mortality among young injection drug users in Vancouver. Harm Reduct J. 2007; 4: 1.

31. Galli M, Musicco M. Mortality of intravenous drug users living in Milan, Italy: role of HIV-1 infection. COMCAT Study Group. AIDS. 1994; 8(10): 1457-63.

32. Manfredi R, Sabbatani S, Agostini D. Trend of mortality observed in a cohort of drug addicts of the metropolitan area of Bologna, North-Eastern Italy, during a 25-year-period. *Coll Antropol* 2006; **30**(3): 479-88.

33. Eskild A, Magnus P, Samuelsen SO, Sohlberg C, Kittelsen P. Differences in mortality rates and causes of death between HIV positive and HIV negative intravenous drug users. *Int J Epidemiol* 1993; **22**(2): 315-20.

34. Ødegård E, Amundsen EJ, Kielland KB. Fatal overdoses and deaths by other causes in a cohort of Norwegian drug abusers – a competing risk approach. *Drug Alcohol Depend* 2007; **89**(2-3): 176-82.

35. Rossow I. Suicide among drug addicts in Norway. *Addiction* 1994; **89**(12): 1667-73.

36. Risser D, Hönigschnabl S, Stichenwirth M, Pfudl S, Sebald D, Kaff A, Bauer G. Mortality of opiate users in Vienna, Austria. *Drug Alcohol Depend.* 2001; **64**(3): 251-6.

37. Bartu A, Freeman NC, Gawthorne GS, Codde JP, Holman CDJ. Mortality in a cohort of opiate and amphetamine users in Perth, Western Australia. *Addiction* 2004; **99**(1): 53-60.

38. Degenhardt L, Randall D, Hall W, Law M, Butler T, Burns L. Mortality among clients of a state-wide opioid pharmacotherapy program over 20 years: risk factors and lives saved. *Drug Alcohol Depend.* 2009; **105**(1): 9–15.

39. Tait RJ, Ngo HTT, Hulse GK. Mortality in heroin users 3 years after naltrexone implant or methadone maintenance treatment. *J Subst Abuse Treat.* 2008; **35**(2): 116-24.

40. Vlahov D, Galai N, Safaeian M, *et al*. Effectiveness of highly active antiretroviral therapy among injection drug users with late-stage human immunodeficiency virus infection. *Am J Epidemiol.* 2005; **161**(11): 999-1012.

41. Vlahov D, Wang C, Ompad D, *et al*, Collaborative Injection Drug User Study. Mortality risk among recent-onset injection drug users in five U.S. cities. *Subst Use Misuse*. 2008; **43**(3-4): 413-28.

42. Oppenheimer E, Tobutt C, Taylor C, Andrew T. Death and survival in a cohort of heroin addicts from London clinics: a 22-year follow-up study. *Addiction* 1994; **89**(10): 1299-308.

43. Goldstein A, Herrera J. Heroin addicts and methadone treatment in Albuquerque: a 22-year follow-up. *Drug Alcohol Depend* 1995; **40**(2): 139-50.

44. Soyka M, Apelt SM, Lieb M, Wittchen H-U. One-year mortality rates of patients receiving methadone and buprenorphine maintenance therapy: a nationally representative cohort study in 2694 patients. *J Clin Psychopharmacol* 2006; **26**(6): 657-60.

45. Fugelstad A, Agren G, Romelsjö A. Changes in mortality, arrests, and hospitalizations in nonvoluntarily treated heroin addicts in relation to methadone treatment. *Subst Use Misuse* 1998; **33**(14): 2803-17.

46. Stenbacka M, Leifman A, Romelsjo A. Mortality Among Opiate Abusers in Stockholm: A Longitudinal Study. *Heroin Addict Relate Clin Probl* 2007; **9**(3): 41-50.

47. Fugelstad A, Annell A, Rajs J, Agren G. Mortality and causes and manner of death among drug addicts in Stockholm during the period 1981-1992. *Acta Psychiatr Scand* 1997; **96**(3): 169-75.

48. Antolini G, Pirani M, Morandi G, Sorio C. [Gender difference and mortality in a cohort of heroin users in the Provinces of Modena and Ferrara, 1975-1999]. *Epidemiol Prev* 2006; **30**(2): 91-9.

49. Digiusto E, Shakeshaft A, Ritter A, O'Brien S, Mattick RP, NEPOD Research Group. Serious adverse events in the Australian National Evaluation of Pharmacotherapies for Opioid Dependence (NEPOD). *Addiction* 2004; **9**9(4): 450-60.

50. Brancato V, Delvecchio G, Simone P. [Survival and mortality in a cohort of heroin addicts in 1985- 1994]. *Minerva Med* 1995; **86**(3): 97-9.

51. Wang C, Vlahov D, Galai N, *et al*. The effect of HIV infection on overdose mortality. *AIDS* 2005; **19**(9): 935-42.

52. Auckloo MBKM, Davies BB. Post-mortem toxicology in violent fatalities in Capte Town, South Africa: A preliminary investigation. *J Foresnsic Leg Med* 2019; **63**:18-25.

53. Brådvik L. Suicide risk and mental disorders. *Int J Environ Res Publ Health* 2019; **15**(9):2028.

54. Merrall E, Bird S, Hutchinson SJ. A record-linkage study of drug-related death and suicide after hospital discharge among drug-treatment clients in Scotland, 1996-2006. *Addiction* 2012; **102**(2).

<u>Dietary risks</u>

Flowchart

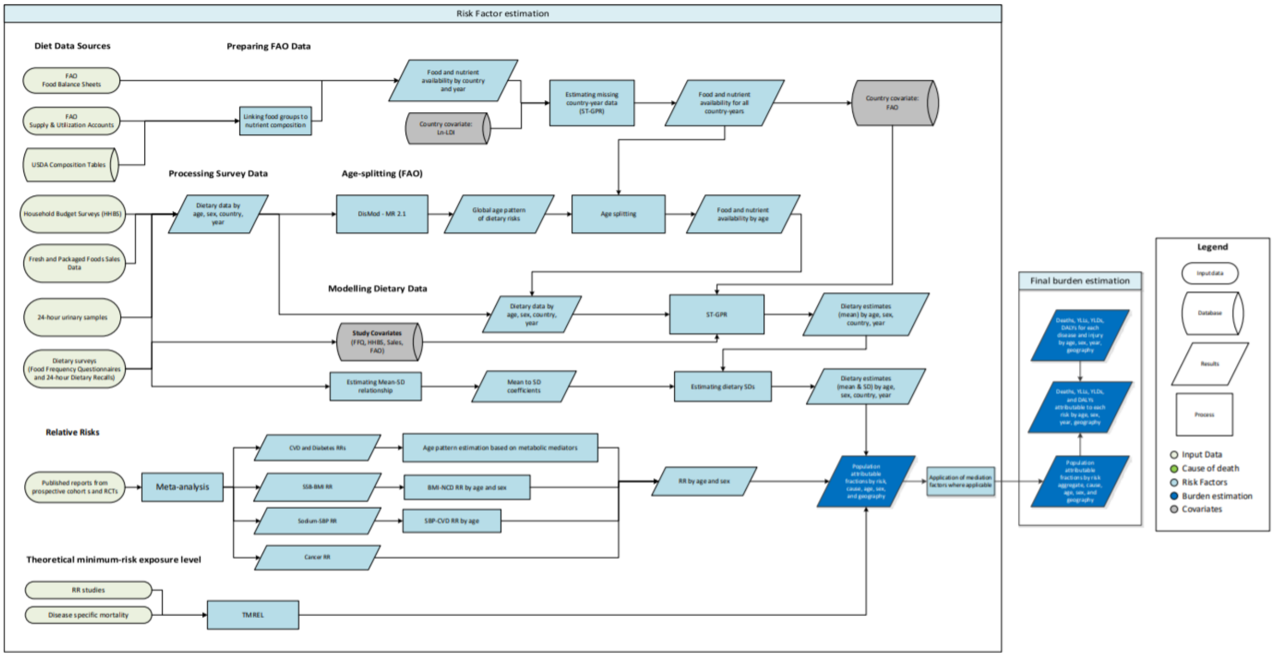

FAO = food and agriculture organization; USDA = United States Department of Agriculture; RCTs = randomised controlled trials; RR = relative risk; TMREL = Theoretical minimum-risk exposure level; LDI = lag distributed income per capita (I$): gross domestic product per capita that has been smoothed over the preceding 10 years; DisMod – MR 2.1 = disease model - Bayesian meta-regression; ST-GPR = spaciotemporal Gaussian process regression; FFQ = Food Frequency Questionnaire; HHBS = Household Budge Survey; CVD = cardiovascular disease; SSB = sugar-sweetened beverages; BMI = body-mass index; SBP = systolic blood pressure; NCD = non-communicable disease; YLL = Years of life lost; YLD = Years lived with disability; DALYs = Disability-adjusted life-years.

Input data and methodological summary

Exposure

Case definitions

**Risk**	**Definition**
Diet low in fruit	Average daily consumption (in grams per day) of less than 310-340 grams of fruit including fresh, frozen, cooked, canned, or dried fruit, excluding fruit juices and salted or pickled fruits
Diet low in vegetables	Average daily consumption (in grams per day) of less than 280-320 grams of vegetables, including fresh, frozen, cooked, canned, or dried vegetables and excluding legumes and salted or pickled vegetables, juices, nuts and seeds, and starchy vegetables such as potatoes or corn
Diet low in whole grains	Average daily consumption (in grams per day) of less than 140-160 grams of whole grains (bran, germ, and endosperm in their natural proportion) from breakfast cereals, bread, rice, pasta, biscuits, muffins, tortillas, pancakes, and other sources

Diet low in milk	Average daily consumption (in grams per day) of less than 360-500 grams of milk including non-fat, low-fat, and full-fat milk, excluding soy milk and other plant derivatives
Diet high in red meat	Any intake (in grams per day) of red meat including beef, pork, lamb, and goat but excluding poultry, fish, eggs, and all processed meats
Diet high in processed meat	Any intake (in grams per day) of meat preserved by smoking, curing, salting, or addition of chemical preservatives
Diet low in fibre	Average daily consumption (in grams per day) of less than 21-22 grams of fibre from all sources including fruits, vegetables, grains, legumes, and pulses
Diet low in calcium	Average daily consumption (in grams per day) of less than 1.06-1.1 grams of calcium from all sources, including milk, yogurt, and cheese
Diet high in sodium	Average 24-hour urinary sodium excretion (in grams per day) greater than 1-5 grams

Input data

In GBD 2019, we included new dietary recall sources from a literature search of PubMed and new sources from the IHME GHDx yearly known survey series updates in our models. We also conducted a new systematic review for sodium. As in GBD 2017, the dietary data that we use in the models comes from multiple sources, including nationally and subnationally representative nutrition surveys, household budget surveys, accounts of national sales from the Euromonitor, and availability data from the United Nations FAO Supply and Utilization Accounts (SUA).

The availability data for food groups in GBD were previously based on the FAO Food Balance Sheets (FBS), which provide tabulated and processed data of national food supply. In GBD 2019, to more accurately characterise the national availability of various food groups, we used more disaggregated data on food commodities that were included in FAO SUA and recreated the national availability of each food group based on the GBD definition of the food group. We modelled missing country-year data from FAO using a spatiotemporal Gaussian process regression and lag-distributed country income as the covariate. For nutrient availability, we continued to use data from Global Nutrient Database.[1]

For each dietary factor, we estimated the global age pattern of consumption based on nutrition surveys (ie, 24-hour diet recall) and applied that age pattern to the all-age data (availability, sales and household budget surveys) before the data source bias adjustment.

Our gold-standard data source for all dietary risks (except sodium) is 24-hour dietary recall surveys where food and nutrient intake are reported or convertible to grams per person per day; the gold-standard data source for sodium is 24-hour urinary sodium. The other data sources we use – household budget surveys, food frequency questionnaires, sales, and availability – are treated as alternate definitions for dietary intake and crosswalked to the gold-standard definition. In GBD 2016 and GBD 2017, we determined the bias adjustment factors from a mixed effects linear regression. In GBD 2019, we used MR-BRT (a network meta-regression) to determine the adjustment factors for non-gold standard data points. Coefficients for these models can be found below in "**MR-BRT crosswalk adjustment factors for all dietary risks**".

Types of data sources (other than 24-hour dietary recall) and covariates used in modelling of each dietary factor

	Data sources				Country-level covariate
	Sales	FFQ[1]	HBS[2]	FAO	
Diet low in fruits	●	●	●	●	Lag distributed income
Diet low in vegetables	●	●	●	●	Energy availability (kcal)
Diet low in whole grains	-	●	-	●	Energy availability (kcal)
Diet low nuts and seeds	-	-	●	●	Energy availability (kcal)
Diet low in milk	●	●	●	●	Energy availability (kcal)
Diet high in red meat	●	●	●	●	Energy availability (kcal)
Diet high in processed meat	●	●	●	-	Energy availability (kcal), pigs per capita
Diet low in legumes	●	●	-	●	Energy availability (kcal)
Diet high in sugar-sweetened beverages	●	●	●	-	Energy availability (kcal), availability of sugar
Diet low in fibre	-	●	-	●	Energy availability (kcal)
Diet suboptimal in calcium	-	●	-	●	Energy availability (kcal)
Diet low in seafood omega-3 fatty acids	-	-	-	●	Lag distributed income, proportion landlocked area
Diet low in polyunsaturated fatty acids	-	●	-	●	Lag distributed income
Diet high in trans fatty acids	●	●	-	-	
Diet high in sodium[3]	-	-	-	-	

[1]Food Frequency Questionnaire
[2]Household Budge Survey
[3]For sodium, we used data from the 24-hour urinary sodium and 24-hour dietary recall

MR-BRT crosswalk adjustment factors for all dietary risks

**Dietary risk**	**Sex**	**Data input**	**Reference or alternative case definition**	**Gamma**	**Beta coefficient log (95% CI)**	**Adjustment factor***
Calcium	---	DR	Ref	0.24	---	---
Calcium	Female	FAO	Alt		0.04 (0.04, 0.5)	0.96 (0.64, 1.65)
Calcium	Female	FFQ	Alt		-0.04 (-0.04, 0.43)	1.04 (0.59, 1.53)
Calcium	Male	FAO	Alt		0.17 (0.17, 0.63)	0.84 (0.73, 1.88)
Calcium	Male	FFQ	Alt		0.09 (0.09, 0.55)	0.91 (0.67, 1.74)
Fibre	---	DR	Ref	0.33	---	---
Fibre	Female	FAO	Alt		0.56 (0.56, 1.17)	0.57 (0.93, 3.23)
Fibre	Female	FFQ	Alt		0.27 (0.27, 0.88)	0.76 (0.69, 2.41)
Fibre	Male	FAO	Alt		0.55 (0.55, 1.17)	0.57 (0.92, 3.22)
Fibre	Male	FFQ	Alt		0.26 (0.26, 0.88)	0.77 (0.69, 2.4)
Fruit	---	DR	Ref	0.76	---	---
Fruit	Female	FAO	Alt		0.36 (0.36, 1.83)	0.7 (0.31, 6.21)

Fruit	Female	Sales	Alt		0.73 (0.73, 2.19)	0.48 (0.45, 8.98)
Fruit	Female	FFQ	Alt		-0.15 (-0.15, 1.32)	1.17 (0.19, 3.73)
Fruit	Female	HHBS	Alt		0.23 (0.23, 1.71)	0.79 (0.27, 5.5)
Fruit	Male	FAO	Alt		0.32 (0.32, 1.79)	0.73 (0.3, 5.97)
Fruit	Male	Sales	Alt		0.69 (0.69, 2.16)	0.5 (0.43, 8.64)
Fruit	Male	FFQ	Alt		-0.19 (-0.19, 1.28)	1.21 (0.18, 3.58)
Fruit	Male	HHBS	Alt		0.19 (0.19, 1.66)	0.83 (0.26, 5.27)
Legumes	---	DR	Ref	0.74	---	---
Legumes	Female	FAO	Alt		-0.08 (-1.49, 1.39)	1.08 (0.22, 4)
Legumes	Female	Sales	Alt		-0.9 (-2.31, 0.56)	2.47 (0.1, 1.75)
Legumes	Female	FFQ	Alt		-0.53 (-1.94, 0.95)	1.7 (0.14, 2.58)
Legumes	Male	FAO	Alt		0.06 (-1.35, 1.53)	0.94 (0.26, 4.61)
Legumes	Male	Sales	Alt		-0.76 (-2.16, 0.7)	2.14 (0.12, 2.01)
Legumes	Male	FFQ	Alt		-0.39 (-1.79, 1.09)	1.47 (0.17, 2.98)
Milk	---	DR	Ref	1.06	---	---
Milk	Female	FAO	Alt		0.27 (0.27, 2.57)	0.76 (0.16, 13.01)
Milk	Female	Sales	Alt		0.01 (0.01, 2.31)	0.99 (0.13, 10.11)
Milk	Female	FFQ	Alt		0.46 (0.46, 2.78)	0.63 (0.18, 16.2)
Milk	Female	HHBS	Alt		-0.61 (-0.61, 1.69)	1.84 (0.07, 5.4)
Milk	Male	FAO	Alt		0.28 (0.28, 2.58)	0.75 (0.17, 13.17)
Milk	Male	Sales	Alt		0.03 (0.03, 2.33)	0.97 (0.13, 10.23)
Milk	Male	FFQ	Alt		0.48 (0.48, 2.8)	0.62 (0.18, 16.43)
Milk	Male	HHBS	Alt		-0.59 (-0.59, 1.7)	1.81 (0.07, 5.48)
Nuts	---	DR	Ref	1.58	---	---
Nuts	Female	FAO	Alt		0.49 (0.49, 3.63)	0.62 (0.06, 37.68)
Nuts	Female	FFQ	Alt		-0.34 (-0.34, 2.76)	1.41 (0.02, 15.75)
Nuts	Female	HHBS	Alt		-0.72 (-0.72, 2.42)	2.06 (0.02, 11.27)
Nuts	Male	FAO	Alt		0.6 (0.6, 3.73)	0.55 (0.07, 41.65)
Nuts	Male	FFQ	Alt		-0.23 (-0.23, 2.87)	1.26 (0.03, 17.58)
Nuts	Male	HHBS	Alt		-0.62 (-0.62, 2.54)	1.85 (0.02, 12.66)
Omega-3	---	DR	Ref	0.12	---	---
Omega-3	Male	FAO	Alt		-1.15 (-1.15, -0.92)	3.16 (0.25, 0.4)
Omega-3	Female	FAO	Alt		-1.01 (-1.01, -0.78)	2.75 (0.29, 0.46)
Proc. meat	---	DR	Ref	1.21	---	---
Proc. meat	Female	Sales	Alt		0.79 (0.79, 3.14)	0.46 (0.19, 23.07)
Proc. meat	Female	FFQ	Alt		-0.3 (-0.3, 2.25)	1.35 (0.05, 9.49)
Proc. meat	Female	HHBS	Alt		-0.46 (-0.46, 1.89)	1.59 (0.05, 6.63)
Proc. meat	Male	Sales	Alt		0.95 (0.95, 3.3)	0.39 (0.22, 27.03)
Proc. meat	Male	FFQ	Alt		-0.13 (-0.13, 2.42)	1.14 (0.06, 11.2)
Proc. meat	Male	HHBS	Alt		-0.3 (-0.3, 2.06)	1.35 (0.06, 7.82)
PUFA	---	DR	Ref	0.14	---	---
PUFA	Female	FAO	Alt		-0.14 (-0.14, 0.14)	1.15 (0.65, 1.15)
PUFA	Female	FFQ	Alt		1.05 (1.05, 1.43)	0.35 (1.96, 4.18)
PUFA	Male	FAO	Alt		-0.18 (-0.18, 0.1)	1.2 (0.62, 1.1)
PUFA	Male	FFQ	Alt		1 (1, 1.38)	0.37 (1.87, 3.98)
Red meat	---	DR	Ref	0.83	---	---
Red meat	Female	FAO	Alt		0.89 (0.89, 2.54)	0.41 (0.45, 12.69)
Red meat	Female	Sales	Alt		1.09 (1.09, 2.74)	0.34 (0.54, 15.49)
Red meat	Female	FFQ	Alt		-0.34 (-0.34, 1.6)	1.4 (0.11, 4.95)

Red meat	Female	HHBS	Alt		0.45 (0.45, 2.1)	0.64 (0.29, 8.18)
Red meat	Male	FAO	Alt		0.89 (0.89, 2.54)	0.41 (0.45, 12.66)
Red meat	Male	Sales	Alt		1.09 (1.09, 2.74)	0.34 (0.54, 15.43)
Red meat	Male	FFQ	Alt		-0.34 (-0.34, 1.6)	1.4 (0.11, 4.94
Red meat	Male	HHBS	Alt		0.45 (0.45, 2.1)	0.64 (0.29, 8.15)
Sodium	---	Urinary sodium	Ref	0.39	---	---
Sodium	Female	DR	Alt		-0.02 (-0.02, 0.85)	1.02 (0.38, 2.34)
Sodium	Female	FFQ	Alt		0.47 (0.47, 1.29)	0.63 (0.69, 3.64)
Sodium	Male	DR	Alt		-0.06 (-0.06, 0.8)	1.06 (0.38, 2.23)
Sodium	Male	FFQ	Alt		0.43 (0.43, 1.26)	0.65 (0.67, 3.52)
SSBs	---	DR	Ref	0.61	---	---
SSBs	Female	Sales	Alt		0.15 (0.15, 1.43)	0.86 (0.37, 4.17)
SSBs	Female	FFQ	Alt		-0.01 (-0.01, 1.32)	1.01 (0.3, 3.75)
SSBs	Female	HHBS	Alt		-0.59 (-0.59, 0.68)	1.8 (0.18, 1.98)
SSBs	Male	Sales	Alt		0.35 (0.35, 1.63)	0.7 (0.45, 5.1)
SSBs	Male	FFQ	Alt		0.19 (0.19, 1.53)	0.83 (0.37, 4.6)
SSBs	Male	HHBS	Alt		-0.39 (-0.39, 0.89)	1.48 (0.22, 2.43)
Trans fat	---	DR	Ref	0.22	---	---
Trans fat	Male	Sales	Alt		-0.23 (-1.27, 0.94)	1.25 (0.28, 2.55)
Trans fat	Female	Sales	Alt		-0.23 (-1.27, 0.94)	1.25 (0.28, 2.55)
Trans fat	Male	FFQ	Alt		0.59 (-2.72, 4.23)	0.56 (0.07, 68.72)
Trans fat	Female	FFQ	Alt		0.86 (-2.63, 4.9)	0.42 (0.07, 134.0)
Vegetables	---	DR	Ref	0.64	---	---
Vegetables	Female	FAO	Alt		0.12 (0.12, 1.33)	0.89 (0.31, 3.78)
Vegetables	Female	Sales	Alt		0.62 (0.62, 1.83)	0.54 (0.51, 6.21)
Vegetables	Female	FFQ	Alt		-0.05 (-0.05, 1.16)	1.05 (0.26, 3.18)
Vegetables	Female	HHBS	Alt		0.1 (0.1, 1.31)	0.91 (0.3, 3.69)
Vegetables	Male	FAO	Alt		0.16 (0.16, 1.37)	0.85 (0.32, 3.94)
Vegetables	Male	Sales	Alt		0.66 (0.66, 1.87)	0.52 (0.53, 6.49)
Vegetables	Male	FFQ	Alt		-0.01 (-0.01, 1.2)	1.01 (0.27, 3.32)
Vegetables	Male	HHBS	Alt		0.14 (0.14, 1.35)	0.87 (0.32, 3.85)
Whole grains	---	DR	Ref	0.69	---	---
Whole grains	Female	FAO	Alt		1.94 (1.94, 3.37)	0.14 (1.82, 29.05)
Whole grains	Female	FFQ	Alt		-0.35 (-0.35, 1.37)	1.42 (0.13, 3.94)
Whole grains	Male	FAO	Alt		2.09 (2.09, 3.52)	0.12 (2.12, 33.76)
Whole grains	Male	FFQ	Alt		-0.2 (-0.2, 1.52)	1.22 (0.15, 4.58)

**Adjustment factor is the transformed beta coefficient in normal space and can be interpreted as the factor by which the alternative case definition is adjusted to reflect what it would have been if measured as the reference.*

Modelling strategy

Exposure model

We use a spatiotemporal Gaussian process regression (ST-GPR) framework to estimate the mean intake of each dietary factor by age, sex, country, and year. In GBD 2019, we removed lag-distributed income as a covariate from most of our models and added country-level energy availability. To characterise the distribution of each dietary factor at the population level, we use an ensemble approach that separately fit 12 distributions for individual-level microdata to specific to each data source's sampled population. The respective goodness of fit of each family was assessed, and a weighting scheme was determined to

optimise overall fit to the unique distribution of each risk factor. A global mean of the weights for each risk factor's data sources was created. We then determined the standard deviation of each population's consumption through a linear regression that captured the relationship between the standard deviation and mean of intake in nationally representative nutrition surveys using 24-hour diet recalls:

$$ln\ (Standard\ deviation) = \beta_0 + \beta_1 \times ln\ (Mean_i)$$

Then we applied the coefficients of this regression to the outputs of our ST-GPR model to calculate the standard deviation of intake by age, sex, year, and country. We also quantified the within-person variation in consumption of each dietary component and adjusted the standard deviations accordingly.

Theoretical minimum-risk exposure level

The dietary TRMELs were updated for GBD 2019. For harmful dietary risks other than sodium, TMREL was set to zero. For protective dietary risk factors, we first calculated the level of intake associated with the lowest risk of mortality from each disease endpoint based on the 85th percentile of intake across all epidemiological studies included in the meta-analysis of the risk-outcome pair. Then we calculated the TMREL as the weighted average of these numbers using the global number of deaths from each outcome as the weight.

Relative risks

For GBD 2019, we performed systematic reviews for each dietary risk and its related outcomes. Using the sources identified during these searches, we incorporated the most recent epidemiological evidence assessing the relationship between each GBD dietary risk factor and related outcomes in our relative risk analysis. After evaluating all available evidence, we found sufficient evidence on the casual relationship for 8 new R-O pairs and insufficient evidence for 5 old R-O pairs. Based on these results, we updated the R-O pairs used the GBD dietary risk factor analysis in the following ways:

Removed:
Diet low in fruit and nasopharynx cancer
Diet low in fruit and other pharynx cancer
Diet low in fruit and oesophageal cancer
Diet low in fruit and larynx cancer
Diet low in whole grains and haemorrhagic stroke

Added:
Diet low in whole grains and colon and rectum cancer
Diet high in red meat and breast cancer
Diet high in red meat and ischaemic heart disease
Diet high in red meat and haemorrhagic stroke
Diet high in red meat and ischaemic stroke
Diet low in fibre and ischaemic stroke
Diet low in fibre and haemorrhagic stroke
Diet low in fibre and diabetes mellitus

Additionally, based on the most recent epidemiological evidence and GBD 2019 newly developed methods for characterising the risk curve, we updated the dose-response curve of relative risks for all dietary risks. For sodium, we continued to estimate its effect on cardiovascular disease based on the effect of sodium on systolic blood pressure.

There is a well-documented attenuation of the risk for cardiovascular disease due to metabolic risks factors throughout one's life. To incorporate this age trend in the relative risks, we first identified the median age-at-event across all cohorts and considered that as the reference age group. We then assigned our newly estimated risk curves to this reference age group. Then, we derived the percentage change in relative risks between each age group and the reference age group by averaging percentage changes in relative risks of all metabolic mediators. The three cardiovascular disease outcomes for dietary risks are haemorrhagic stroke (including intracerebral hemmorhage and subarachnoid hemmorhage), ischaemic stroke, and ischaemic heart disease, and the effects of dietary risks on them are mediated through high systolic blood pressure, cholesterol (not included for haemorrhagic stroke), and fasting plasma glucose. Since the effect of diet is estimated independently of body-mass index (BMI) in the GBD, BMI was not included as a mediator in the RR age trend analysis.

References

For methodological summaries included on pages 122-128: ***Dietary risks***

1. Schmidhuber J, Sur P, Fay K, *et al*. The Global Nutrient Database: Availability of Macronutrients and Micronutrients in 195 Countries from 1980 to 2013. *The Lancet Planetary Health*, vol. 2, no. 8, 2018, doi:10.1016/s2542-5196(18)30170-0.

<u>Unsafe sex</u>

Flowchart

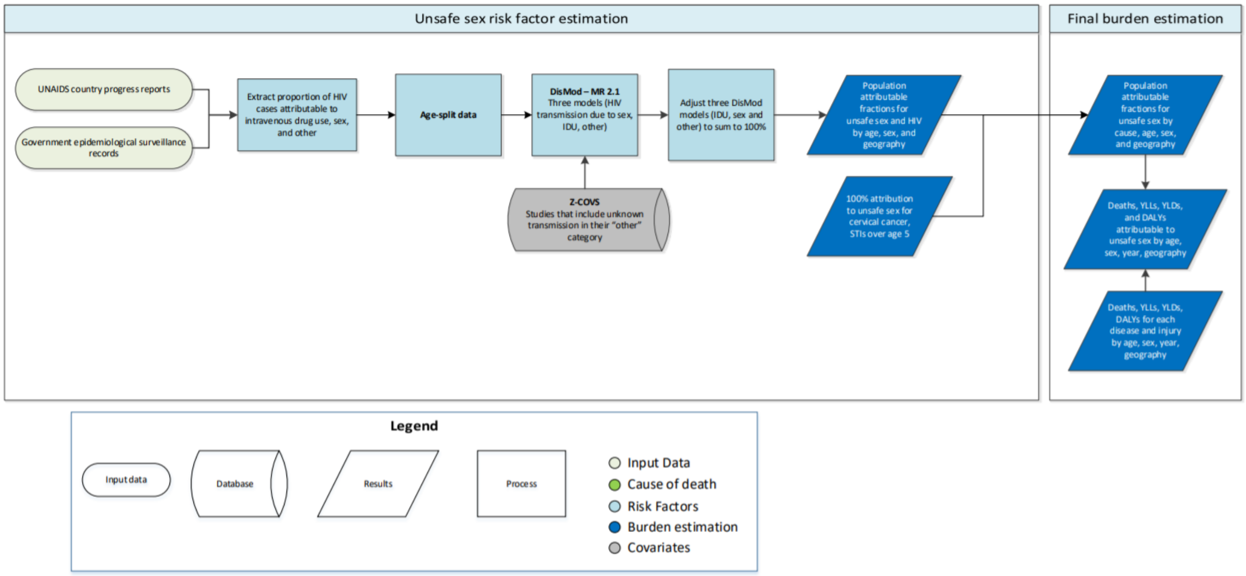

UNAIDS = Joint United Nations Programme on HIV/AIDS; HIV = human immunodeficiency virus; DisMod-MR 2.1 = disease model - Bayesian meta-regression; IDU = injectable drug use; STIs = sexually transmitted infections; YLL = Years of life lost; YLD = Years lived with disability; DALYs = Disability-adjusted life-years.

Input data and methodological summary

Exposure

Case definition

Unsafe sex is defined as the risk of disease due to sexual transmission. The outcomes associated with unsafe sex that we estimate for GBD include HIV, cervical cancer, and all sexually transmitted diseases (STDs) except for those in neonates from vertical transmission, including HIV, *Opthalmia neonatorum* and neonatal syphilis. We assumed 100% of cervical cancer and STDs were attributable to unsafe sex and modelled the proportion of HIV incidence occurring through sexual transmission to estimate the attributable burden for HIV due to unsafe sex. The theoretical minimum level (TMREL) for unsafe sex is defined as the absence of disease transmission due to sexual contact.

Input data

To be used in our models, sources must report HIV cases attributable to various modes of transmission. We screened UNAIDS country progress reports and searched government epidemiological surveillance records for these data. The primary data sources we used were UNAIDS, the European CDC, and the US CDC.

We excluded all extractions where the “other” category for HIV transmissions accounted for greater than 25% of all cases. We believe that such high proportions raise concerns about the quality of reporting.

Modelling strategy

We modelled the proportion of HIV cases attributable to unsafe sex. To do this we collected and cleaned data, ran three DisMod-MR models (HIV attributable to sex, HIV attributable to injection drug use, HIV

attributable to other routes of transmission), adjusted results of the three DisMod-MR models to sum to one, and then assigned the proportions as direct PAFs.
No country-level covariates were included in the models. We tested an injection drug use (IDU) covariate – an opioid use covariate in the proportion HIV due to drug use model – but found no significant coefficients, so excluded them from the final model.

Since all-age and both-sex datapoints represent the vast majority of the available data, we derived an age sex pattern for the HIV-IDU transmission model from the age-sex pattern present in the GBD 2017 population attributable fraction for hepatitis B attributable to IDU (the model for injecting drug use and hepatitis estimates the cumulative exposure to injecting drug use to capture all infections in people with a history of injecting even if in a more distant past). Assuming the proportion of HIV due to other transmission is constant over age and by sex, the age-sex pattern for the proportion of HIV due to sexual transmission was set to be the complement to 1 of the age-sex pattern for the proportion of HIV due to IDU. The all-age and both-sex data were split according to these age-sex patterns, and the three HIV transmission DisMod-MR models were run on the age- and sex-split data. In previous GBD rounds, only age-splitting had used this approach, while sex-splitting occurred within DisMod-MR. Since most data are for both sexes combined, using the sex ratio – in addition to the age pattern from the IDU-Hepatitis B PAF – is much more informative. The impact of this change resulted in general increases in proportion HIV due to sexual transmission among females, as they generally had lower IDU rates compared to males.

In GBD 2019, we also changed the proportion HIV due to sex DisMod-MR model to run in complement (1-proportion) space. Since proportions were high in most countries, modelling in complement space resulted in a better model fit. Additional priors were set to inform an age pattern: zero proportion HIV transmission due to IDU before age 15, zero proportion HIV transmission due to sex before age 10 (100 in complement space), and 100% transmission due to other before age 10. The results from these HIV transmission models were adjusted to sum to 100% for a given country-year-age-sex group at each of 1,000 draws.

Theoretical minimum-risk exposure level
The theoretical minimum level used for unsafe sex is the absence of disease transmission due to sexual contact.

Population attributable fraction calculation
Based on evidence in the literature, we attributed 100% of cervical cancer to unsafe sex. These sources state that HPV infection is necessary for cervical cancer to develop and that HPV is only spread through sexual contact. The proportion of STDs attributable to unsafe sex was also 100%.

For HIV, the results from the single parameter proportion DisMod-MR model for HIV transmission due to sex after squeezing were used directly as the population attributable fraction.

<u>Low physical activity</u>

Flowchart

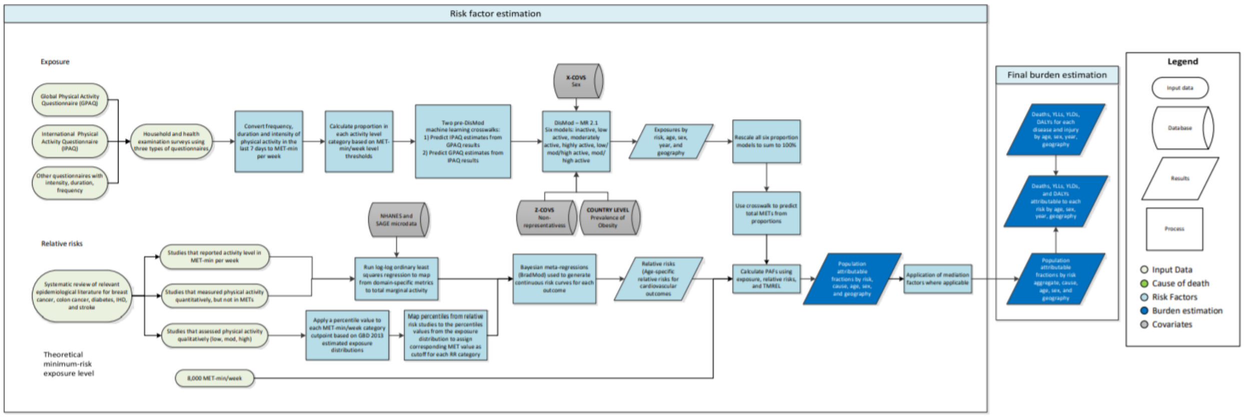

IHD = ischaemic heart disease; MET = metabolic equivalent of task; GBD = Global Burden of Disease Study; NHANES = National Health and Nutrition Examination Survey; PAF = Population attributable fraction; TMREL = Theoretical minimum-risk exposure level; YLL = Years of life lost; YLD = Years lived with disability; DALYs = Disability-adjusted life-years.

Input data and methodological summary

Exposure

Case definition

We measure physical activity performed by adults older than 25 years of age, for duration of at least ten minutes at a time, across all domains of life (leisure/recreation, work/household and transport). We use frequency, duration and intensity of activity to calculate total metabolic equivalent-minutes per week. MET (Metabolic Equivalent) is the ratio of the working metabolic rate to the resting metabolic rate. One MET is equivalent to 1 kcal/kg/hour and is equal to the energy cost of sitting quietly. A MET is also defined as the oxygen uptake in ml/kg/min with one MET equal to the oxygen cost of sitting quietly, around 3.5 ml/kg/min.

Input data

We included surveys of the general adult population that captured self-reported physical activity in all domains of life (leisure/recreation, work/household and transport), where random sampling was used. Data were primarily derived from two standardised questionnaires: The Global Physical Activity Questionnaire (GPAQ)[1] and the International Physical Activity Questionnaire (IPAQ)[2], although we included other survey instruments that asked about intensity, frequency and duration of physical activities performed across all activity domains.

Due to a lack of a consistent relationship on the individual level between activity performed in each domain and total activity, we were not able to use studies that included only recreational/leisure activities.

Physical activity level is categorised by total MET-minutes per week using four categories based on rounded values closest to the quartiles of the global distribution of total MET-minutes/week. The lower limit for the Level 1 category (600 MET-min/week) is the recommended minimum amount of physical activity to get any health benefit. We used four categories with higher thresholds rather than the GPAQ

and IPAQ recommended 3 categories to better capture any additional protective effects from higher activity levels.

- Level 0: < 600 MET-min/week (inactive)
- Level 1: 600-3999 MET-min/week (low-active)
- Level 2: 4000-7,999 MET-min/week (moderately-active)
- Level 3: ≥ 8,000 MET-min/week (highly active)

The GHDx was used to locate all surveys that use the GPAQ or IPAQ questionnaire. Although there were many other surveys that focused specifically on leisure activity, we were unable to use these sources because they did not comprise all three domains (work, transport and leisure). In addition, we excluded any surveys that did not report frequency, duration, and intensity of activity.

Modelling strategy

DisMod modelling

For this round of the GBD, we have chosen to use a machine learning crosswalk to predict IPAQ estimates for GPAQ results and GPAQ estimates for IPAQ results, with original and estimated results then being combined to get one comprehensive IPAQ dataset and one comprehensive GPAQ dataset. We then estimated the proportion of each country/year/age/sex subpopulation in each of the above four activity levels using 12 separate Dismod models (one set of six for IPAQ and one for GPAQ). We use six categories of physical activity prevalence rather than four to accommodate the different MET-minute/week cutoffs presented in tabulated data sources where individual unit record data was not available. Since the accepted threshold/definition for inactivity is consistently <600 MET-minutes/week, the vast majority of tabulated data was broken down into proportion inactive (model A) and proportion low, moderate or highly active (model B).

	**Label**	**MET-min/week**	**Name of sequelae in online visualisation tool**
A	inactive	<600	Physical inactivity and low physical activity, inactive
B	low/moderately/highly active	00	Physical inactivity and low physical activity, low/moderately/highly active
C	low active	600-3999	Physical inactivity and low physical activity, low active
D	moderately/highly active	>4000	Physical inactivity and low physical activity, moderately/highly active
E	moderately active	4000-7999	Physical inactivity and low physical activity, moderately active
F	highly active	000	Physical inactivity and low physical activity, highly active

These models have mesh points at 0 15 25 35 45 55 65 75 85 100, and a study-level fixed effect on integrand variance (Z-cov) for whether a study was nationally representative or not, to account for the heterogeneity introduced by studies that are not generalisable to the entire population. They also have national level fixed effects on prevalence of obesity.

After DisMod, we rescale each of the 6 models specific to each data source so that the proportions sum to one. Since we have the most data for models A and B, we rescale the sum of the proportion in each category to be equal to one. Next we rescale the sum of model C and D to be equal to the rescaled value

from model B. Then we rescale the sum of models E and F to be equal to the rescaled value from model D. After these three rescales we are left with a proportion for each of the four categories that all sum to 1. Scaled results for each data source are then hybridised to produce only one set of results for the prevalence of the four categories of physical activity.

Similar to the previous round, we have not directly estimated total MET-minutes per week globally. Although, this year we made use of two specific machine learning algorithms (Random Forest and XGBoost) that were trained using data that could characterise the relationship between total MET-mins/week and each of the categorical prevalences of physical activity. This resulted in country-year-age-sex specific estimates of total physical activity in the form of MET-minutes per week.

Utilising microdata on total MET-mins per week from individual-level surveys, we characterised the distribution of activity level at the population level. We then used an ensemble approach to distribution fitting, borrowing characteristics from individual distributions to tailor a unique distribution to fit the data using a weighting scheme. We characterised the standard deviation of each population's activity through a linear regression that captured the relationship between standard deviation and mean activity levels in nationally representative IPAQ surveys:

$$ln\ (Standard\ deviation) = \beta_0 + \beta_1 \times ln\ (Mean_i) + \beta_2 \times Age_i + \beta_3 \times SR_i + \beta_4 \times Fem_i$$

Age_i is the youngest age in population *i*'s age group, SR_i, is the super-region in which the population lives, and Fem_i is a Boolean value depicting whether the population is female. We then applied the coefficients of this regression to the outputs of our estimate of total MET-minutes per week regression outputs to calculate the standard deviation by country, year, age, and sex.

Theoretical minimum-risk exposure level

The theoretical minimum-risk exposure level for physical inactivity is 3000-4500 MET-min per week, which was calculated as the exposure at which minimal deaths across outcomes occurred.[3]

Relative risk

We used a dose-response meta-analysis of prospective cohort studies to estimate the effect size of the change in physical activity level on breast cancer, colon cancer, diabetes, ischemic heart disease and ischemic stroke.[3]

There is a well-documented attenuation of the risk for cardiovascular disease and diabetes due to metabolic risks factors throughout one's life. To incorporate this age trend in the relative risks, we first identified the median age-at-event across all cohorts and considered that as the reference age-group. We then assigned our risk curves to this reference age group. Then, we derived the percent change in relative risks between each age group and the reference age group by averaging percentage changes in relative risks of all metabolic mediators.

References

For methodological summaries included on pages 131-133: ***Low physical activity***

1. World Health Organization. Global Physical Activity Questionnaire (GPAQ) Analysis Guide. 2011. Geneva, Switzerland: WHO Google Scholar. 2013

2. IPAQ Research Committee. Guidelines for data processing and analysis of the International Physical Activity Questionnaire (IPAQ)–short and long forms. Retrieved September. 2005;17:2008.

3. Kyu HH, Bachman VF, Alexander LT, *et al*. Physical activity and risk of breast cancer, colon cancer, diabetes, ischemic heart disease, and ischemic stroke events: systematic review and doseresponse meta-analysis for the Global Burden of Disease Study 2013. *BMJ* 2016 Aug 9;354:i3857. doi: 10.1136/bmj.i3857.

High fasting plasma glucose

Flowchart

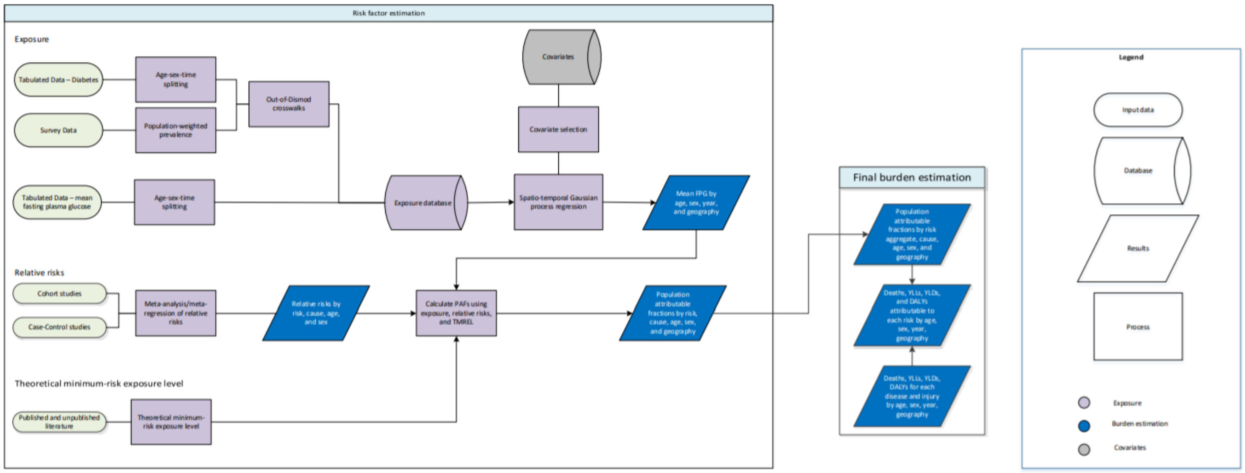

PAF = Population attributable fraction; TMREL = Theoretical minimum-risk exposure level; FPG = fasting plasma glucose; YLL = Years of life lost; YLD = Years lived with disability; DALYs = Disability-adjusted life-years.

Input data and methodological summary

Exposure

Case definition

High fasting plasma glucose (FPG) is measured as the mean FPG in a population, where FPG is a continuous exposure in units of mmol/L. Since FPG is along a continuum, we define high FPG as any level above the TMREL, which is 4.8-5.4 mmol/L.

Input data

We conducted a systematic review for FPG and diabetes in GBD 2019. We use all available sources on FPG and prevalence of diabetes in the FPG model.

1. Search terms:

Diabetes Mellitus search string: (diabetes[TI] AND (prevalence[TIAB] OR incidence[TIAB])) OR ('Diabetes Mellitus'[MeSH Terms] AND 'epidemiology'[MeSH Terms]) OR (diabetes[TI] AND 'epidemiology'[MeSH Terms]) NOT gestational[All Fields] NOT ('neoplasms'[MeSH Terms] OR 'neoplasms'[All Fields] OR 'cancer'[All Fields]) NOT ('mice'[MeSH Terms] OR 'mice'[All Fields]) NOT ('schizophrenia'[MeSH Terms] OR 'schizophrenia'[All Fields]) NOT ('emigrants and immigrants'[MeSH Terms] OR ('emigrants'[All Fields] AND 'immigrants'[All Fields]) OR 'emigrants and immigrants'[All Fields] OR 'immigrants'[All Fields]) NOT ('pregnancy'[MeSH Terms] OR 'pregnancy'[All Fields] OR 'gestation'[All Fields]) NOT ('rats'[MeSH Terms] OR 'rats'[All Fields] OR 'rat'[All Fields]) NOT ('kidney'[MeSH Terms] OR 'kidney'[All Fields]) NOT renal[All Fields] NOT ('vitamins'[Pharmacological Action] OR 'vitamins'[MeSH Terms] OR 'vitamins'[All Fields] OR 'vitamin'[All Fields])

And

FPG search string: ((“glucose”[Mesh] OR “hyperglycemia”[Mesh] OR “prediabetic state”[Mesh]) AND "Geographic Locations"[Mesh] NOT "United States"[Mesh]) AND ("humans"[Mesh] AND

"adult"[MeSH]) AND ("Data Collection"[Mesh] OR "Health Services Research"[Mesh] OR "Population Surveillance"[Mesh] OR "Vital statistics"[Mesh] OR "Population"[Mesh] OR "Epidemiology"[Mesh] OR surve*[TiAb]) NOT Comment[ptyp] NOT Case Reports[ptyp]) NOT "hospital"[TiAb]

Search date: October 17, 2018. The search took place for the following dates: 10/15/2017-10/16/2018. The number of studies returned was 717, and the number of studies extracted was 36.

Data inputs come from 3 sources:

- Estimates of mean FPG in a representative population
- Individual-level data of fasting plasma glucose measured from surveys
- Estimates of diabetes prevalence in a representative population

Data sources that did not report mean FPG or prevalence of diabetes are excluded from analysis. When a study reported both mean fasting plasma glucose (FPG) and prevalence of diabetes, we use the mean FPG for exposure estimates. Where possible, individual-level data supersede any data described in a study. Individual-level data are aggregated to produce estimates for each 5-year age group, sex, location, and year of a survey.

Modelling strategy

Data processing
We perform several processing steps to the data in order to address sampling and measurement inconsistencies that will ensure the data are comparable.

1. *Small sample size*
   Estimates in a sex and age group with a sample size <30 persons is considered a small sample size. In order to avoid small sample size problems that may bias estimates, data are collapsed into the next age group in the same study till the sample size reach at least 30 persons. The intent of collapsing the data is to preserve as much granularity between age groups as possible. If the entire study sample consists of <30 persons and did not include a population-weight, the study is excluded from the modelling process.

2. *Crosswalks*
   We predicted mean FPG from diabetes prevalence using an ensemble distribution. We characterised the distribution of FPG using individual-level data. Details on the ensemble distribution can be found elsewhere in the Appendix. Before predicting mean FPG from prevalence of diabetes, we ensured that the prevalence of diabetes was based on the reference case definition: fasting plasma glucose (FPG) >126 mg/dL (7 mmol/L) or on treatment. For more details on how the case-definition crosswalk is conducted, please see the diabetes mellitus appendix in "Global, regional, and national incidence, prevalence, and years lived with disability for 354 diseases and injuries for 195 countries, 1990–2019: a systematic analysis for the Global Burden of Disease Study 2019".[3]

Exposure modelling
Exposure estimates are produced for every year between 1980 to 2019 for each national and subnational location, sex, and for each 5-year age group starting from 25 years. As in previous rounds of GBD, we used a Spatio-Temporal Gaussian Process Regression (ST-GPR) framework to model the mean fasting plasma glucose at the location-, year-, age-, and sex- level.

Fasting plasma glucose is frequently tested or reported in surveys aiming at assessing the prevalence of diabetes mellitus. In these surveys, the case definition of diabetes may include both a glucose test and questions about treatment for diabetes. People with positive history of diabetes treatment may be excluded from the FPG test. Thus, the mean FPG in these surveys would not represent the mean FPG in the entire population. In this event, we estimated the prevalence of diabetes assuming a definition of FPG>126 mg/dL (7mmol/L), then crosswalked it to our reference case definition, and then predicted mean FPG.

To inform our estimates in data-sparse countries, we systematically tested a range of covariates and selected age specific prevalence of obesity as a covariate based on direction of the coefficient and significance level.

Mean FPG is estimated using a mixed-effects linear regression, run separately by sex:

$$\text{logit}(\text{FPG}_{c,a,t}) = \beta_0 + \beta_1 p_{\text{overweight}_{c,a,t}} + \sum_{k=2}^{16} \beta_k I_{A[a]} + \alpha_s + \alpha_r + \alpha_c + \epsilon_{c,a,t}$$

where $p_{\text{overweight}_{c,a,t}}$ is the prevalence of overweight, $I_{A[a]}$ is an indicator variable for a fixed effect on a given 5-year age group, and α_s α_r α_c are random effects at the super-region, region, and country level, respectively. The estimates were then propagated through the ST-GPR framework to obtain 1000 draws for each location, year, age, and sex.

Theoretical minimum-risk exposure level

The theoretical minimum-risk exposure level (TMREL) for FPG is 4.8-5.4 mmol/L. This was calculated by taking the person-year weighted average of the levels of FPG that were associated with the lowest risk of mortality in the pooled analyses of prospective cohort studies.[1]

Relative risks

GBD 2019 estimates 15 outcomes due to high fasting plasma glucose (continuous risk) or diabetes (categorical risk).

**Risk**	**Outcome**
Fasting plasma glucose	Ischemic heart disease
Fasting plasma glucose	Ischemic stroke
Fasting plasma glucose	Subarachnoid hemorrhage
Fasting plasma glucose	Intracerebral hemorrhage
Fasting plasma glucose	Peripheral vascular disease
Fasting plasma glucose	Type 1 diabetes
Fasting plasma glucose	Type 2 diabetes
Fasting plasma glucose	Chronic kidney disease due to Type 1 diabetes
Fasting plasma glucose	Chronic kidney disease due to Type 2 diabetes
Diabetes mellitus	Drug-resistant tuberculosis
Diabetes mellitus	Drug-susceptible tuberculosis
Diabetes mellitus	Multidrug-resistant tuberculosis without extensive drug resistance

Diabetes mellitus	Extensively drug-resistant tuberculosis
Diabetes mellitus	Liver cancer due to NASH
Diabetes mellitus	Liver cancer due to other causes
Diabetes mellitus	Pancreatic cancer
Diabetes mellitus	Ovarian cancer
Diabetes mellitus	Colon and rectum cancer
Diabetes mellitus	Bladder cancer
Diabetes mellitus	Lung cancer
Diabetes mellitus	Breast cancer
Diabetes mellitus	Glaucoma
Diabetes mellitus	Cataracts
Diabetes mellitus	Dementia

Relative risks for High Fasting Plasma Glucose (continuous risk)

After a review of the chronic kidney disease literature, we determined that there is only an attributable risk of chronic kidney disease due to diabetes type 1 and chronic kidney disease due to diabetes type 2 to FPG. Thus, in GBD 2019 we removed chronic kidney disease due to glomerulonephritis, chronic kidney disease due to hypertension, chronic kidney disease due to other causes as an outcome.

Relative risks (RR) were obtained from dose-response meta-analysis of prospective cohort studies. Please see the citation list for a full list of studies that are utilised. For cardiovascular outcomes, we estimated age-specific RRs using DisMod-MR 2.1 with log (RR) as the dependent variable and median age at event as the independent variable with an intercept at age 110. Morbidity and mortality directly caused by diabetes type 1 and diabetes type 2 is considered directly attributable to FPG.

<u>References</u>

For methodological summaries included on pages 135-138: ***High fasting plasma glucose***

1. Singh GM, Danaei G, Farzadfar F, *et al*. The age-specific quantitative effects of metabolic risk factors on cardiovascular diseases and diabetes: a pooled analysis. *PloS One* 2013; **8**: e65174.

High body-mass index

Flowchart, Adult (ages 20+) high body-mass index

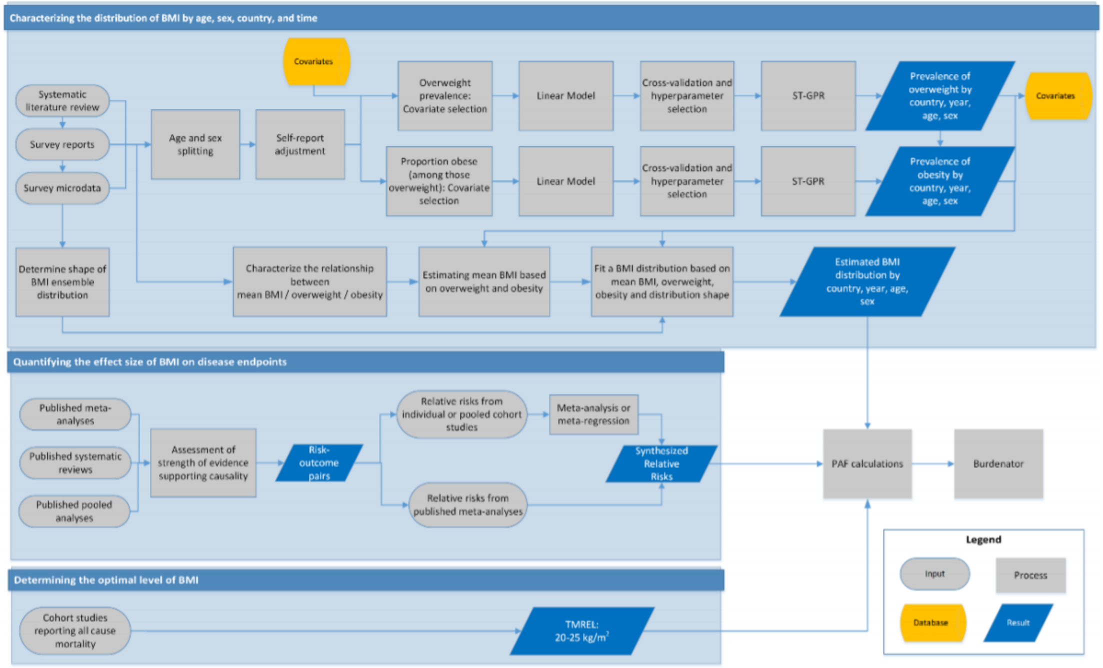

BMI = body-mass index; ST-GPR = spaciotemporal Gaussian process regression; PAF = Population attributable fraction; TMREL = Theoretical minimum-risk exposure level.

Flowchart, Childhood (ages 2-19) high body-mass index

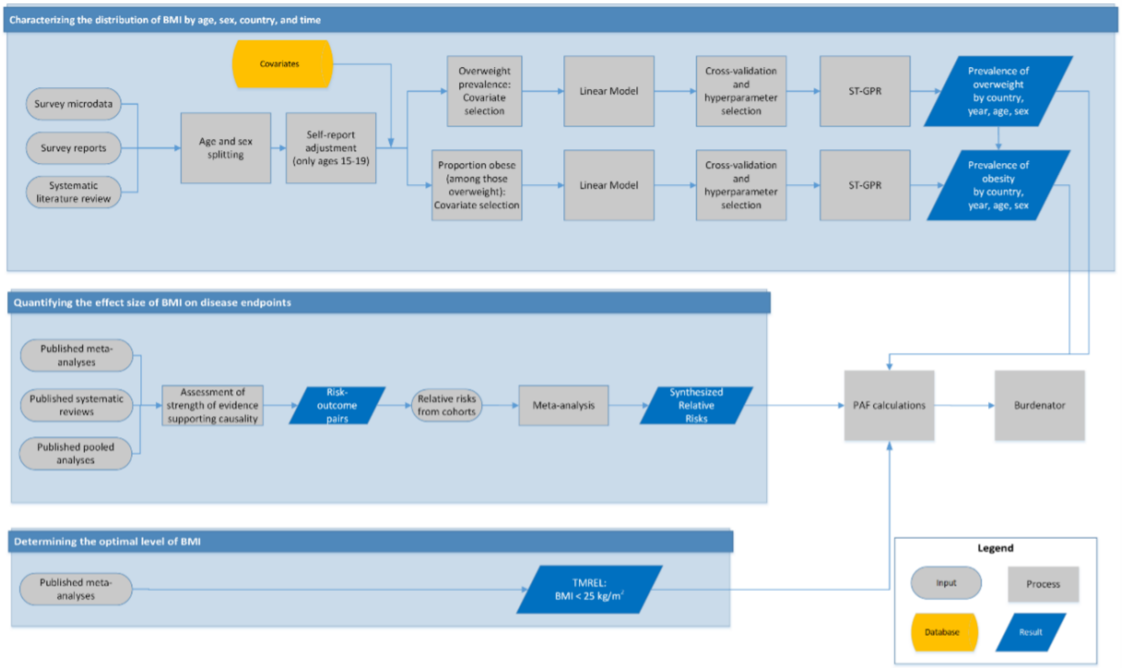

BMI = body-mass index; ST-GPR = spaciotemporal Gaussian process regression; PAF = Population attributable fraction; TMREL = Theoretical minimum-risk exposure level;

Input data and methodological summary

Exposure

Case definition

High body-mass index (BMI) for adults (ages 20+) is defined as BMI greater than 20 to 25 kg/m^2. High BMI for children (ages 1–19) is defined as being overweight or obese based on International Obesity Task Force standards.

Input data

In GBD 2019, new data were added from sources included in the annual GHDx update of known survey series. We conducted a systematic review in GBD 2017 to identify studies providing nationally or subnationally representative estimates of overweight prevalence, obesity prevalence, or mean body-mass index (BMI). We limited the search to literature published between January 1, 2016, and December 31, 2016, to update the systematic literature search previously performed as part of GBD 2015.

The search for adults was conducted on 4 January 2017, using the following terms:

((("Body Mass Index"[Mesh] OR "Overweight"[Mesh] OR "Obesity"[Mesh]) AND ("Geographic Locations"[Mesh] NOT “United States”[Mesh]) AND ("humans"[Mesh] AND "adult"[MeSH]) AND ("Data Collection"[Mesh] OR "Health Services Research"[Mesh] OR "Population Surveillance"[Mesh] OR "Vital statistics"[Mesh] OR "Population"[Mesh] OR "Epidemiology"[Mesh] OR "surve*"[TiAb]) NOT (Comment[ptyp] OR Case Reports[ptyp] OR "hospital"[TiAb])) AND ("2016/01/01"[Date Publication] : "2016/12/31"[Date - Publication]))

The search for children was conducted on 4 August 2016, using the following terms: ((("Body Mass Index"[Mesh] OR "Overweight"[Mesh] OR "Obesity"[Mesh]) AND ("Geographic Locations"[Mesh] NOT “United States”[Mesh]) AND ("humans"[Mesh] AND "child"[MeSH]) AND ("Data Collection"[Mesh] OR "Health Services Research"[Mesh] OR "Population Surveillance"[Mesh] OR "Vital statistics"[Mesh] OR "Population"[Mesh] OR "Epidemiology"[Mesh] OR "surve*"[TiAb]) NOT (Comment[ptyp] OR Case Reports[ptyp] OR "hospital"[TiAb])) AND ("2016/01/01"[Date - Publication] : "2016/12/31"[Date - Publication]))

Eligibility criteria

We included representative studies providing data on mean BMI or prevalence of overweight or obesity among adults or children. For adults, studies were included if they defined overweight as BMI≥25 kg/m^2 and obesity as BMI≥30 kg/m^2, or if estimates using those cutoffs could be back-calculated from reported categories. For children (children ages 2–19), studies were included if they used International Obesity Task Force (IOTF) standards to define overweight and obesity thresholds. We only included studies reporting data collected after January 1, 1980. Studies were excluded if they used non-random samples (eg, casecontrol studies or convenience samples), conducted among specific subpopulations (eg, pregnant women, racial or ethnic minorities, immigrants, or individuals with specific diseases), used alternative methods to assess adiposity (eg, waist-circumference, skin-fold thickness, or hydrodensitometry), had sample sizes of less than 20 per age-sex group, or provided inadequate information on any of the inclusion criteria. We also excluded review articles and non-English-language articles.

Data collection process

Where individual-level survey data were available, we computed mean BMI using weight and height. We then used BMI to determine the prevalence of overweight and obesity. For individuals aged over 19 years, we considered them to be overweight if their BMI was greater than or equal to 25 kg/m^2, and obese if their BMI was greater than or equal to 30 kg/m^2. For individuals aged 2 to 19 years, we used monthly IOTF cutoffs[2] to determine overweight and obese status when age in months was available. When only age in years was available, we used the cutoff for the midpoint of that year. Obese individuals were also considered to be overweight. We excluded studies using the World Health Organization (WHO) standards or country-specific cutoffs to define childhood overweight and obesity. At the individual level, we considered BMI<10 kg/m^2 and BMI>70 kg/m^2 to be biologically implausible and excluded those observations.

The rationale for choosing to use the IOTF cutoffs over the WHO standards has been described elsewhere.[1] Briefly, the IOTF cutoffs provide consistent child-specific standards for ages 2–18 derived from surveys covering multiple countries. By contrast, the WHO growth standards apply to children under age 5, and the WHO growth reference applies to children ages 5–19. The WHO growth reference for children ages 5–19 was derived from United States data, which are less representative than the multinational data used by IOTF. Additionally, the switch between references at age 5 can produce artificial discontinuities. Given that we estimate global childhood overweight and obesity for ages 2–19 (with ages 19 using standard adult cutoffs), the IOTF cutoffs were preferable. Additionally, we found that IOTF cutoffs were more commonly used in scientific literature covering childhood obesity. From report and literature data, we extracted data on mean BMI, prevalence of overweight, and prevalence of obesity, measures of uncertainty for each, and sample size, by the most granular age and sex groups available. Additionally, we extracted the same study-level covariates as were extracted from microdata (measurement, urbanicity, and representativeness), as well as location and year.

In addition to the primary indicators described above, we extracted relevant survey-design variables, including primary sampling unit, strata, and survey weights, which were used to tabulate individual-level microdata and produce accurate measures of uncertainty. We extracted three study-level covariates: 1) whether height and weight data were measured or self-reported; 2) whether the study was predominantly conducted in an urban area, rural area, or both; and 3) the level of representativeness of the study (national or subnational).

Finally, we extracted relevant demographic indicators, including location, year, age, and sex. We estimated the standard error of the mean from individual-level data, where available, and used the reported standard error of the mean for published data. When multiple data sources were available for the same country, we included all of them in our analysis. If data from the same data source were available in multiple formats such as individual-level data and tabulated data, we used individual-level data.

Modelling strategy

Age and sex splitting

Any report or literature data provided in age groups wider than the standard five-year age groups or as both sexes combined were split using the approach used by Ng and colleagues.[2] Briefly, age-sex patterns were identified using sources with data on multiple age-sex groups and these patterns were applied to split aggregated report and literature data. Uncertainty in the age-sex split was propagated by multiplying the standard error of the data by the square root of the number of splits performed. We did not propagate the uncertainty in the age pattern and sex pattern used to split the data as they seemed to have small effect.

Self-report bias adjustment

We included both measured and self-reported data. We tested for bias in self-report data compared to measured data, which is considered to be the gold-standard. There was no clear direction of bias for children ages 2–14, so for these age groups we only included measured data. For individuals ages 15 and above, we adjusted self-reported data for overweight prevalence and obesity prevalence. In GBD 2017, the self-report bias adjustment used a nested hierarchical mixed-effects regression model. This approach was updated in GBD 2019 to utilise the power of MR-BRT. For both overweight and obesity, we fit sex-specific MR-BRT models on the logit difference between measured and self-reported with a fixed effect on super-region. The bias coefficients derived from these two models are below.

MR-BRT self-report crosswalk adjustment factors for overweight prevalence

**Model**	**Data input**	**Reference or alternative case definition**	**Gamma**	**Beta coefficient logit (95% CI)**
Females	Measured data	Ref	0.26	---
	Self-reported data (southeast Asia, east Asia, and Oceania	Alt		-0.53 (-1.03, -0.04)
	Self-reported data (central Europe, eastern Europe, and central Asia)	Alt		-0.20 (-0.69, 0.30)
	Self-reported data (high-income)	Alt		-0.25 (-0.75, 0.24)
	Self-reported data (Latin America and Caribbean)	Alt		-0.19 (-0.69, 0.31)
	Self-report data (north Africa and Middle East)	Alt		-0.38 (-0.89, 0.11)
	Self-report data (south Asia)	Alt		0.36 (-0.14, 0.85)
	Self-report data (sub-Saharan Africa)	Alt		-0.26 (-0.76, 0.24)
Males	Measured data	Ref	0.43	---
	Self-reported data (southeast Asia, east Asia, and Oceania	Alt		-0.36 (-1.17, 0.50)
	Self-reported data (central Europe, eastern Europe, and central Asia)	Alt		-0.03 (-0.84, 0.82)
	Self-reported data (high-income)	Alt		0.05 (-0.77, 0.87)
	Self-reported data (Latin America and Caribbean)	Alt		-0.02 (-0.84, 0.81)
	Self-report data (north Africa and Middle East)	Alt		-0.21 (-1.04, 0.61)
	Self-report data (south Asia)	Alt		0.53 (-0.28, 1.37)
	Self-report data (sub-Saharan Africa)	Alt		-0.27 (-1.09, 0.55)

MR-BRT self-report crosswalk adjustment factors for obesity prevalence

**Model**	**Data input**	**Reference or alternative case definition**	**Gamma**	**Beta coefficient logit (95% CI)**
Females	Measured data	Ref	0.38	---
	Self-reported data (southeast Asia, east Asia, and Oceania	Alt		-0.11 (-0.86, 0.64)

	Self-reported data (central Europe, eastern Europe, and central Asia)	Alt		-0.95 (-1.70, -0.19)
	Self-reported data (high-income)	Alt		-0.42 (-1.16, 0.34)
	Self-reported data (Latin America and Caribbean)	Alt		-0.41 (-1.16, 0.34)
	Self-report data (north Africa and Middle East)	Alt		-0.48 (-1.23, 0.27)
	Self-report data (south Asia)	Alt		0.50 (-0.25, 1.26)
	Self-report data (sub-Saharan Africa)	Alt		-0.41 (-1.16, 0.34)
Males	Measured data	Ref	0.74	---
	Self-reported data (southeast Asia, east Asia, and Oceania	Alt		0.04 (-1.41, 1.53)
	Self-reported data (central Europe, eastern Europe, and central Asia)	Alt		-0.79 (-2.25, 0.71)
	Self-reported data (high-income)	Alt		-0.13 (-1.58, 1.40)
	Self-reported data (Latin America and Caribbean)	Alt		-0.26 (-1.70, 1.21)
	Self-report data (north Africa and Middle East)	Alt		-0.33 (-1.77, 1.16)
	Self-report data (south Asia)	Alt		0.66 (-0.78, 2.15)
	Self-report data (sub-Saharan Africa)	Alt		-0.41 (-1.86, 1.08)

Prevalence estimation for overweight and obesity
After adjusting for self-report bias and splitting aggregated data into five-year age-sex groups, we used spatiotemporal Gaussian process regression (ST-GPR) to estimate the prevalence of overweight and obesity. This modelling approach has been described in detail elsewhere.

The linear model, which when added to the smoothed residuals forms the mean prior for GPR is as follows:

$$\text{logit}(\text{overweight})_{c,a,t} = \beta_0 + \beta_1\text{energy}_{c,t} + \beta_2\text{SDI}_{c,t} + \beta_3\text{vehicles}_{c,t} + \beta_4\text{agriculture}_{c,t} + \sum_{k=5}^{21}\beta_k I_{A[a]} + \alpha_s + \alpha_r + \alpha_c$$

$$\text{logit}(\text{obesity}/\text{overweight})_{c,a,t} = \beta_0 + \beta_1\text{energy}_{c,t} + \beta_2\text{SDI}_{c,t} + \beta_3\text{vehicles}_{c,t} + \sum_{k=4}^{21}\beta_k I_{A[a]} + \alpha_s + \alpha_r + \alpha_c$$

where $\text{energy}_{c,t}$ is ten-year lag-distributed energy consumption per capita, $\text{SDI}_{c,t}$ is a composite index of development including lag-distributed income per capita, education, and fertility, $\text{vehicles}_{c,t}$ is the number of two- or four-wheel vehicles per capita, and $\text{agriculture}_{c,t}$ is the proportion of the population working in agriculture. $I_{A[a]}$ is a dummy variable indicating specific age group A that the prevalence point captures, and α_s, α_r, and α_c are super-region, region, and country random intercepts, respectively. Random effects were used in model fitting but were not used in prediction.

We tested all combinations of the following covariates to see which performed best in terms of in-sample AIC for the overweight linear model and the obesity as a proportion of overweight linear model: ten-year lag-distributed energy per capita, proportion of the population living in urban areas, SDI, lag-distributed income per capita, educational attainment (years) per capita, proportion of the population working in agriculture, grams of sugar adjusted for energy per capita, grams of sugar not adjusted for energy per

capita, and the number of two- or four-wheeled vehicles per capita. We selected these candidate covariates based on theory as well as reviewing covariates used in other publications. The final linear model was selected based on 1) if the direction of covariates matched what is expected from theory, 2) all the included covariates were significant, and 3) minimising in-sample AIC. The covariate selection process was performed using the dredge package in R.

Estimating mean BMI

To estimate the mean BMI for adults in each country, age, sex, and time period 1980–2019, we first used the following nested hierarchical mixed-effects model, fit using restricted maximum likelihood on data from sources containing estimates of all three indicators (prevalence of overweight, prevalence of obesity, and mean BMI), in order to characterise the relationship between overweight, obesity, and mean BMI:

$$\log(\mathrm{BMI}_{c,a,s,t}) = \beta_0 + \beta_1 \mathrm{ow}_{c,a,s,t} + \beta_2 \mathrm{ob}_{c,a,s,t} + \beta_3 \mathrm{sex} + \sum_{k=4}^{20} \beta_k \mathrm{I}_{A[a]} + \alpha_s(1 + \mathrm{ow}_{c,a,s,t} + \mathrm{ob}_{c,a,s,t}) + \alpha_r(1 + \mathrm{ow}_{c,a,s,t} + \mathrm{ob}_{c,a,s,t}) + \alpha_c(1 + \mathrm{ow}_{c,a,s,t} + \mathrm{ob}_{c,a,s,t}) + \epsilon_{c,a,s,t}$$

where $\mathrm{ow}_{c,a,s,t}$ is the prevalence of overweight in country c, age a, sex s, and year t, $\mathrm{ob}_{c,a,s,t}$ is the prevalence of obesity in country c, age a, sex s, and year t, sex is a fixed effect on sex, $\mathrm{I}_{A[a]}$ is an indicator variable for age, and α_s, α_r, and α_c are random effects at the super-region, region, and country, respectively. The model was run in Stata 13.

We applied 1000 draws of the regression coefficients to the 1000 draws of overweight prevalence and obesity prevalence produced through ST-GPR to estimate 1000 draws of mean BMI for each country, year, age, and sex. This approach ensured that overweight prevalence, obesity prevalence, and mean BMI were correlated at the draw level and uncertainty was propagated.

Estimating BMI distribution

We used the ensemble distribution approach described in the manuscript. We fit ensemble weights by source and sex, with source- and sex-specific weights averaged across all sources included to produce the final global weights. The ensemble weights were fit on measured microdata. The final ensemble weights were exponential = 0.002, gamma = 0.028, inverse gamma = 0.085, log-logistic = 0.187, Gumbel = 0.220, Weibull = 0.011, log-normal = 0.058, normal = 0.012, beta = 0.136, mirror gamma = 0.008, and mirror Gumbel = 0.113.

One thousand draws of BMI distributions for each location, year, age group, and sex estimated were produced by fitting an ensemble distribution using 1000 draws of estimated mean BMI, 1000 draws of estimated standard deviation, and the ensemble weights. Estimated standard deviation was produced by optimising a standard deviation to fit estimated overweight prevalence draws and estimated obesity prevalence draws.

Assessment of risk-outcome pairs

Risk-outcome pairs were defined based on strength of available evidence supporting a causal effect. We performed a systematic review of published meta-analyses, pooled analyses, and systematic reviews available through PubMed using the following search string: ("Body Mass Index"[Mesh] OR "Overweight"[Mesh] OR "Obesity"[Mesh]) AND (Meta-Analysis[ptyp] OR "systematic review"[tiab] OR "pooled analysis"[tiab]). Inclusion criteria are 1) the health outcome is included in GBD, 2) at least one

prospective cohort is included, and 3) that the summary effect size is statistically significant. For outcomes meeting inclusion criteria we completed causal criteria tables to evaluate the strength of evidence supporting a causal relationship. Gallbladder disease, cataract, multiple myeloma, gout, non-Hodgkin lymphoma, asthma, Alzheimer's disease, and atrial fibrillation were added as new outcomes in GBD 2016, resulting in a total of 38 outcomes.

Theoretical minimum-risk exposure level

For adults (ages 20+), the theoretical minimum risk exposure level (TMREL) of BMI (20–25 kg/m^2) was determined based on the BMI level that was associated with the lowest risk of all-cause mortality in prospective cohort studies.[3]

For children (ages 2–19), the TMREL is "normal weight", that is, not overweight or obese, based on IOTF cutoffs.

Relative risks

The relative risk per five-unit change in BMI for each disease endpoint was obtained from meta-analyses, and where available, pooled analyses of prospective observational studies. In cases where a relative risk per five-unit change in BMI was not available we computed our own dose-response meta-analysis using two-step generalised least squares for time trends estimation methods.

For childhood outcomes (ages 2–19), we computed categorical relative risks for overweight and obesity using a random effects meta-analysis.

References

For methodological summaries included on pages 139-145: ***High fasting plasma glucose***

1. Cole, TJ, and T Lobstein. Extended International (IOTF) Body Mass Index Cut-Offs for Thinness, Overweight and Obesity. *Pediatric Obesity* 2012; 7(4): 284–94.

2. Ng M, Fleming T, Robinson M, *et al*. Global, regional, and national prevalence of overweight and obesity in children and adults during 1980–2013: a systematic analysis for the Global Burden of Disease Study 2013. *The Lancet* 2014; **384**: 766–81.

3. Angelantonio ED, Bhupathiraju SN, Wormser D, *et al*. Body-mass index and all-cause mortality: individual-participant-data meta-analysis of 239 prospective studies in four continents. *The Lancet* 2016; **388**: 776–86.

Additional Methodology Tables and Figures

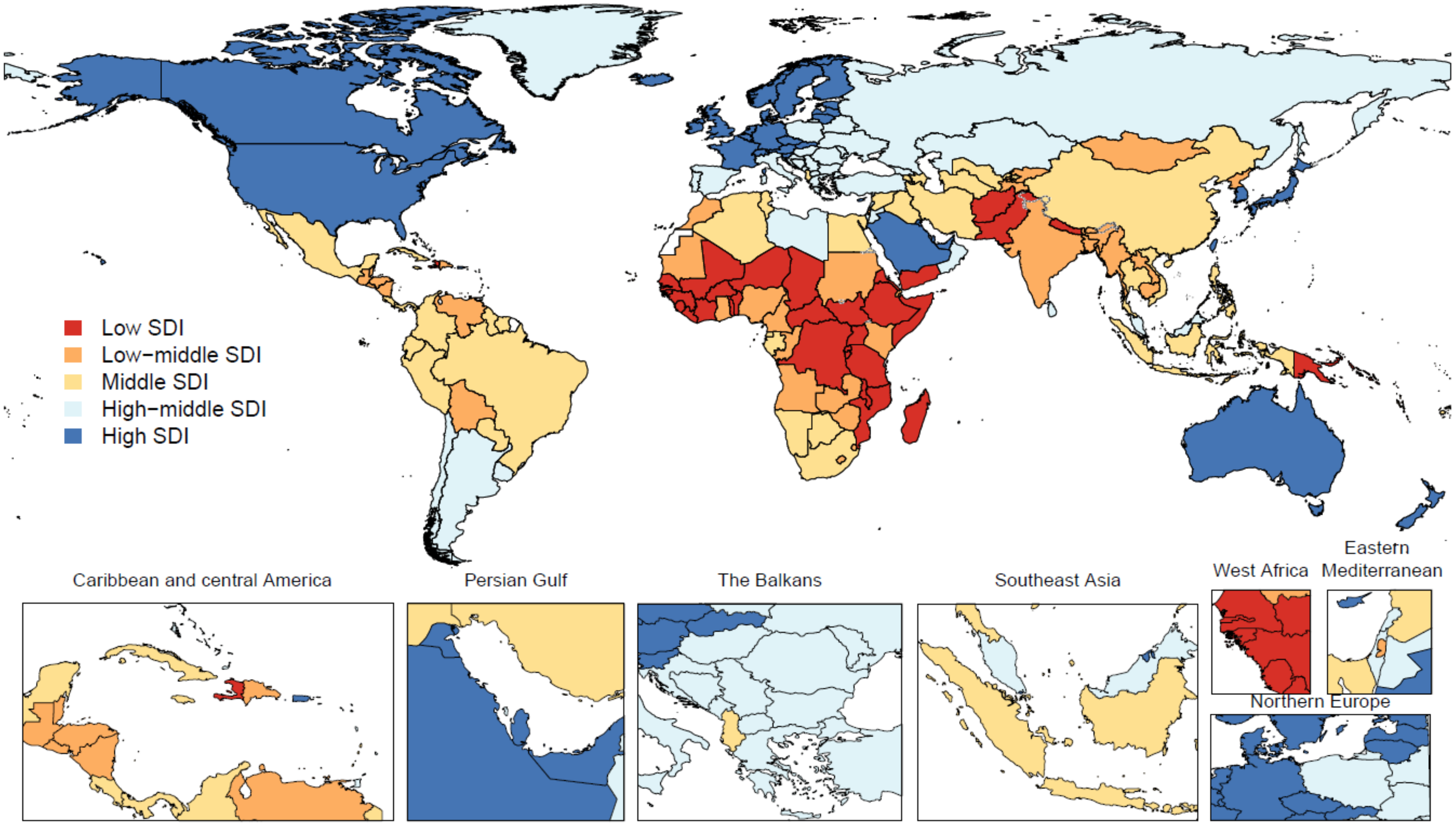

Appendix Figure 4: Socio-demographic Index quintiles for the Global Burden of Disease Study 2019. SDI = Socio-demographic Index.

Appendix Table 7: SDI quintiles for countries estimated in GBD 2019

SDI Quintile	Locations included based on SDI values in 2019 from GBD 2019 results
High SDI	Andorra, Australia, Austria, Belgium, Bermuda, Brunei, Canada, Cyprus, Czechia, Denmark, Estonia, Finland, France, Germany, Guam, Iceland, Ireland, Japan, Kuwait, Latvia, Lithuania, Luxembourg, Monaco, Netherlands, New Zealand, Norway, Puerto Rico, Qatar, San Marino, Saudi Arabia, Singapore, Slovakia, Slovenia, South Korea, Sweden, Switzerland, Taiwan (Province of China), United Arab Emirates, United Kingdom, United States of America
High-middle SDI	American Samoa, Antigua and Barbuda, Argentina, The Bahamas, Bahrain, Barbados, Belarus, Bosnia and Herzegovina, Bulgaria, Chile, Cook Islands, Croatia, Dominica, Georgia, Greece, Greenland, Hungary, Israel, Italy, Jordan, Kazakhstan, Lebanon, Libya, Malaysia, Malta, Mauritius, Moldova, Montenegro, Niue, North Macedonia, Northern Mariana Islands, Oman, Palau, Poland, Portugal, Romania, Russia, Saint Kitts and Nevis, Serbia, Seychelles, Spain, Sri Lanka, Trinidad and Tobago, Turkey, Ukraine, Virgin Islands, Uruguay
Middle SDI	Albania, Algeria, Armenia, Azerbaijan, Botswana, Brazil, China, Colombia, Costa Rica, Cuba, Ecuador, Egypt, Equatorial Guinea, Fiji, Gabon, Grenada, Guyana, Indonesia, Iran, Iraq, Jamaica, Mexico, Namibia, Nauru, Panama, Paraguay, Peru, Philippines, Saint Lucia, Saint Vincent and the Grenadines, Samoa, South Africa, Suriname, Syria, Thailand, Tokelau, Tonga, Tunisia, Turkmenistan, Uzbekistan, Vietnam
Low-middle SDI	Angola, Bangladesh, Belize, Bhutan, Bolivia, Cambodia, Cameroon, Cape Verde, Congo (Brazzaville), Djibouti, Dominican Republic, El Salvador, Eswatini, Federated States of Micronesia, Ghana, Guatemala, Honduras, India, Kenya, Kiribati, Kyrgyzstan, Laos, Lesotho, Maldives, Marshall Islands, Mauritania, Mongolia, Morocco, Myanmar, Nicaragua, Nigeria, North Korea, Palestine, São Tomé and Príncipe, Sudan, Tajikistan, Timor-Leste, Tuvalu, Vanuatu, Venezuela, Zambia, Zimbabwe
Low SDI	Afghanistan, Benin, Burkina Faso, Burundi, Central African Republic, Chad, Comoros, Côte d'Ivoire, DR Congo, Eritrea, Ethiopia, The Gambia, Guinea, Guinea-Bissau, Haiti, Liberia, Madagascar, Malawi, Mali, Mozambique, Nepal, Niger, Pakistan, Papua New Guinea, Rwanda, Senegal, Sierra Leone, Solomon Islands, Somalia, South Sudan, Tanzania, Togo, Uganda, Yemen

SDI = Socio-demographic Index; GBD = Global Burden of Disease study.

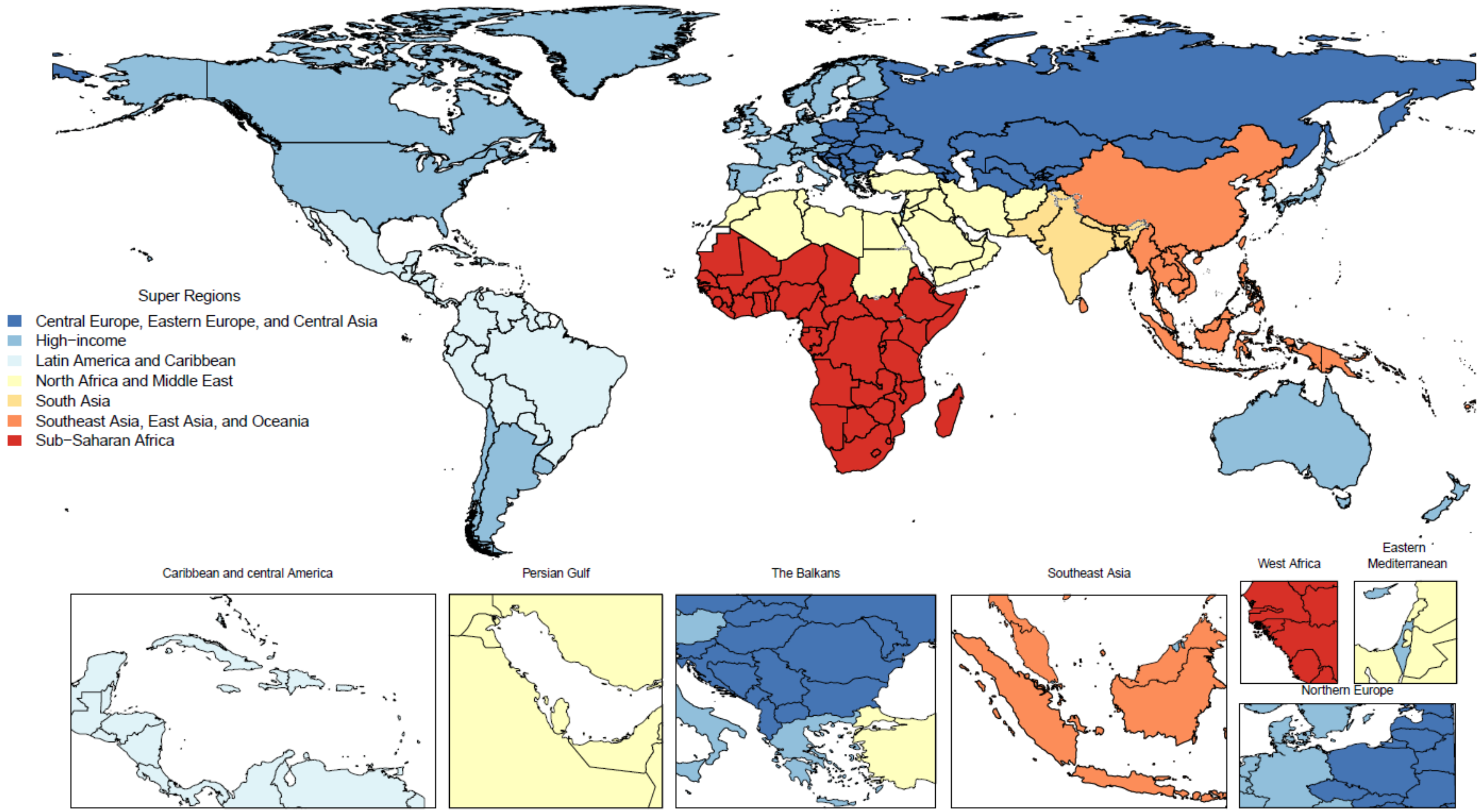

Appendix Figure 5: Map of GBD world super-regions, 2019. There are several geographic locations where estimates are not available (eg, Western Sahara, French Guiana) as they were not modelled locations in the Global Burden of Diseases, Injuries, and Risk Factors 2019 study; these locations are white in this map. GBD = Global Burden of Diseases, Injuries, and Risk Factors Study.

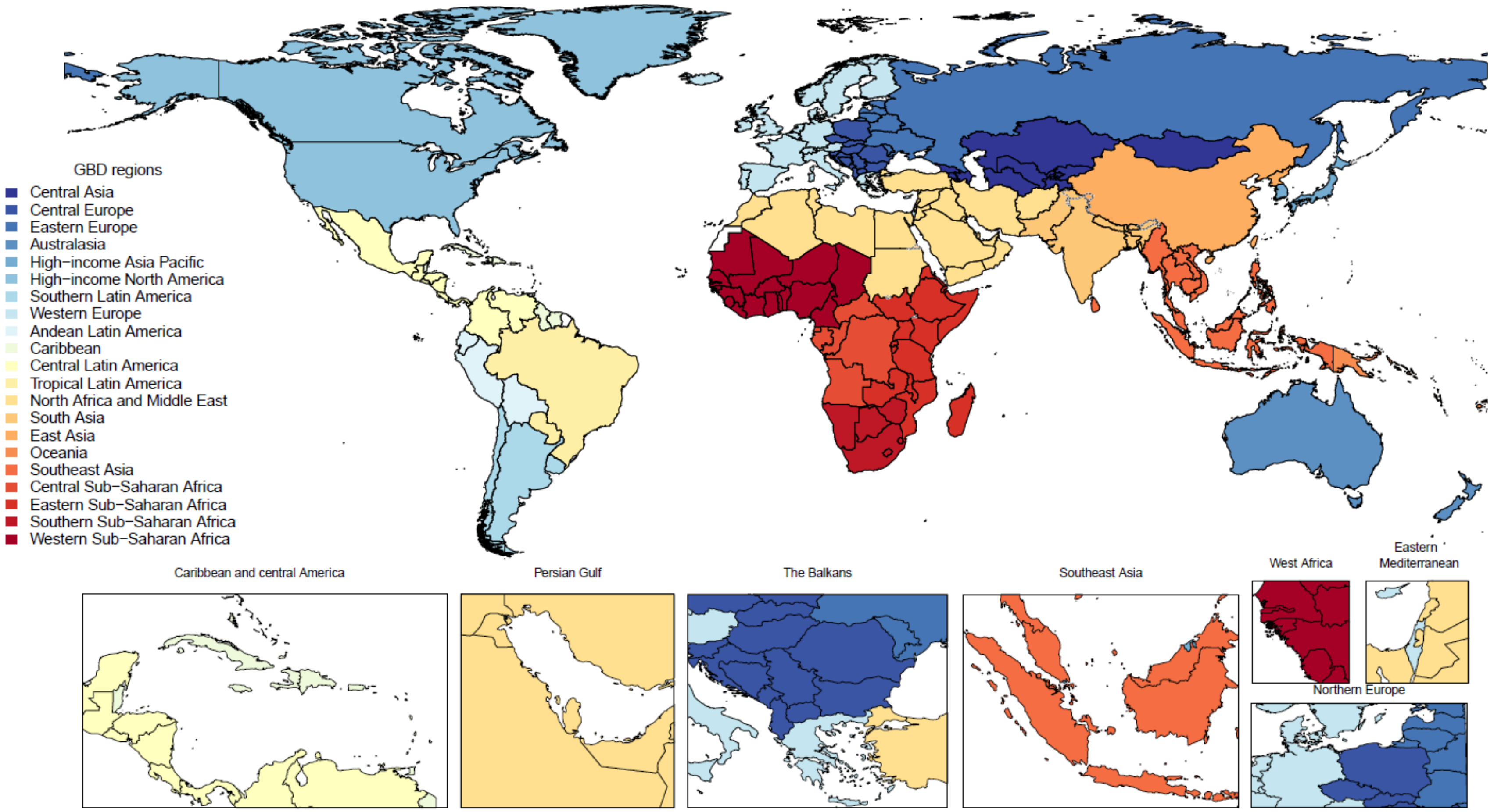

Appendix Figure 6: Map of GBD world regions, 2019. There are several geographic locations where estimates are not available (eg, Western Sahara, French Guiana) as they were not modelled locations in the Global Burden of Diseases, Injuries, and Risk Factors 2019 study; these locations are white in this map. GBD = Global Burden of Diseases, Injuries, and Risk Factors Study.

Appendix Table 8: Cause hierarchy with levels for all cancers in GBD 2019

GBD Cause	Level
All causes	**0**
Non-communicable diseases	1
Neoplasms*	2
Lip and oral cavity cancer	3
Nasopharynx cancer	3
Other pharynx cancer	3
Oesophageal cancer	3
Stomach cancer	3
Colon and rectum cancer	3
Liver cancer	3
Liver cancer due to hepatitis B	4
Liver cancer due to hepatitis C	4
Liver cancer due to alcohol use	4
Liver cancer due to NASH	4
Liver cancer due to other causes	4
Gallbladder and biliary tract cancer	3
Pancreatic cancer	3
Larynx cancer	3
Tracheal, bronchus, and lung cancer	3
Malignant skin melanoma	3
Non-melanoma skin cancer	3
Non-melanoma skin cancer (squamous-cell carcinoma)	4
Non-melanoma skin cancer (basal-cell carcinoma)	4
Breast cancer	3
Cervical cancer	3
Uterine cancer	3
Ovarian cancer	3
Prostate cancer	3
Testicular cancer	3
Kidney cancer	3
Bladder cancer	3
Brain and central nervous system cancer	3
Thyroid cancer	3
Mesothelioma	3
Hodgkin lymphoma	3
Non-Hodgkin lymphoma	3
Multiple myeloma	3
Leukaemia	3
Acute lymphoid leukaemia	4
Chronic lymphoid leukaemia	4
Acute myeloid leukaemia	4
Chronic myeloid leukaemia	4
Other leukaemia	4
Other malignant neoplasms	3
Other neoplasms	3

GBD Cause	Level
Myelodysplastic, myeloproliferative, and other haematopoietic neoplasms	4
Benign and in situ intestinal neoplasms	4
Benign and in situ cervical and uterine neoplasms	4
Other benign and in situ neoplasms	4

*****Where this analysis reports “Total Cancers”, this actually reflects an aggregate of the cancers with associated risk factor burden rather than “Neoplasms”.

This table shows the full list of cancers included under the Level 2 category “Neoplasms” in the GBD 2019 hierarchy. Rows in white represent cancers included in this analysis, as the GBD 2019 study estimated risk attributable burden for these causes for one or more risk factors. Rows in grey represent cancers that are not included in the results of this analysis, as the GBD study does not currently estimate risk attributable burden for these causes.

Appendix Table 9: GBD risk hierarchy with levels

Risk	Level
All risk factors	0
Environmental/occupational risks	1
Unsafe water, sanitation, and handwashing	2
Unsafe water source	3
Unsafe sanitation	3
No access to handwashing facility	3
Air pollution	2
Particulate matter pollution	3
Ambient particulate matter pollution	4
Household air pollution from solid fuels	4
Ambient ozone pollution	3
Non-optimal temperature	2
High temperature	3
Low temperature	3
Other environmental risks	2
Residential radon	3
Lead exposure	3
Occupational risks	2
Occupational carcinogens	3
Occupational exposure to asbestos	4
Occupational exposure to arsenic	4
Occupational exposure to benzene	4
Occupational exposure to beryllium	4
Occupational exposure to cadmium	4
Occupational exposure to chromium	4
Occupational exposure to diesel engine exhaust	4
Occupational exposure to formaldehyde	4
Occupational exposure to nickel	4
Occupational exposure to polycyclic aromatic hydrocarbons	4
Occupational exposure to silica	4
Occupational exposure to sulfuric acid	4
Occupational exposure to trichloroethylene	4
Occupational asthmagens	3
Occupational particulate matter, gases, and fumes	3
Occupational noise	3
Occupational injuries	3
Occupational ergonomic factors	3
Behavioural risks	1
Child and maternal malnutrition	2
Suboptimal breastfeeding	3
Non-exclusive breastfeeding	4
Discontinued breastfeeding	4
Child growth failure	3
Child underweight	4
Child wasting	4
Child stunting	4

Risk	Level
Low birthweight and short gestation	3
Short gestation	4
Low birthweight	4
Iron deficiency	3
Vitamin A deficiency	3
Zinc deficiency	3
Tobacco	2
Smoking	3
Chewing tobacco	3
Secondhand smoke	3
Alcohol use	2
Drug use	2
Dietary risks	2
Diet low in fruits	3
Diet low in vegetables	3
Diet low in legumes	3
Diet low in whole grains	3
Diet low in nuts and seeds	3
Diet low in milk	3
Diet high in red meat	3
Diet high in processed meat	3
Diet high in sugar-sweetened beverages	3
Diet low in fibre	3
Diet low in calcium	3
Diet low in seafood omega-3 fatty acids	3
Diet low in polyunsaturated fatty acids	3
Diet high in trans fatty acids	3
Diet high in sodium	3
Intimate partner violence	2
Childhood sexual abuse and bullying	2
Childhood sexual abuse	3
Bullying victimisation	3
Unsafe sex	2
Low physical activity	2
Metabolic risks	1
High fasting plasma glucose	2
High LDL cholesterol	2
High systolic blood pressure	2
High body-mass index	2
Low bone mineral density	2
Kidney dysfunction	2

This table shows the full list of risk factors included in the GBD 2019 hierarchy. Rows in white represent risk factors included in this analysis, as the GBD 2019 study estimated one or more cancer outcomes attributable to these risks. Rows in grey represent risk factors that are not included in the results of this analysis, as the GBD study does not currently attribute cancer burden from these risks.

Appendix Table 10: GBD 2019 Cancer risk-outcome pairs

Groups of risk factors*	Level 2 risk factors	Specific risk factors	Cancer outcomes
Environmental/Occupational risks	Air pollution	Ambient particulate matter pollution	Tracheal, bronchus, and lung cancer
		Household air pollution from solid fuels	Tracheal, bronchus, and lung cancer
	Other environmental risks	Residential radon	Tracheal, bronchus, and lung cancer
	Occupational risks	Occupational exposure to arsenic	Tracheal, bronchus, and lung cancer
		Occupational exposure to asbestos	Larynx cancer
		Occupational exposure to asbestos	Mesothelioma
		Occupational exposure to asbestos	Ovarian cancer (F)
		Occupational exposure to asbestos	Tracheal, bronchus, and lung cancer
		Occupational exposure to benzene	Leukaemia
		Occupational exposure to beryllium	Tracheal, bronchus, and lung cancer
		Occupational exposure to cadmium	Tracheal, bronchus, and lung cancer
		Occupational exposure to chromium	Tracheal, bronchus, and lung cancer
		Occupational exposure to diesel engine exhaust	Tracheal, bronchus, and lung cancer
		Occupational exposure to formaldehyde	Leukaemia
		Occupational exposure to formaldehyde	Nasopharynx cancer
		Occupational exposure to nickel	Tracheal, bronchus, and lung cancer
		Occupational exposure to polycyclic aromatic hydrocarbons	Tracheal, bronchus, and lung cancer
		Occupational exposure to silica	Tracheal, bronchus, and lung cancer
		Occupational exposure to sulfuric acid	Larynx cancer
		Occupational exposure to trichloroethylene	Kidney cancer
Behavioural risks	Tobacco	Smoking	Bladder cancer
		Smoking	Breast cancer (F)
		Smoking	Cervical cancer (F)
		Smoking	Colon and rectum cancer
		Smoking	Oesophageal cancer
		Smoking	Kidney cancer
		Smoking	Larynx cancer
		Smoking	Leukaemia
		Smoking	Lip and oral cavity cancer

Groups of risk factors*	Level 2 risk factors	Specific risk factors	Cancer outcomes
		Smoking	Liver cancer
		Smoking	Nasopharynx cancer
		Smoking	Other pharynx cancer
		Smoking	Pancreatic cancer
		Smoking	Prostate cancer (M)
		Smoking	Stomach cancer
		Smoking	Tracheal, bronchus, and lung cancer
		Chewing tobacco	Oesophageal cancer
		Chewing tobacco	Lip and oral cavity cancer
		Second-hand smoke	Breast cancer
		Second-hand smoke	Tracheal, bronchus, and lung cancer
	Alcohol use	Alcohol use	Breast cancer
		Alcohol use	Colon and rectum cancer
		Alcohol use	Oesophageal cancer
		Alcohol use	Larynx cancer
		Alcohol use	Lip and oral cavity cancer
		Alcohol use	Liver cancer
		Alcohol use	Nasopharynx cancer
		Alcohol use	Other pharynx cancer
	Drug use	Drug use	Liver cancer
	Dietary risks	Diet high in processed meat	Colon and rectum cancer
		Diet high in red meat	Breast cancer
		Diet high in red meat	Colon and rectum cancer
		Diet high in sodium	Stomach cancer
		Diet low in calcium	Colon and rectum cancer
		Diet low in fibre	Colon and rectum cancer
		Diet low in fruits	Oesophageal cancer
		Diet low in fruits	Tracheal, bronchus, and lung cancer
		Diet low in milk	Colon and rectum cancer
		Diet low in vegetables	Oesophageal cancer
		Diet low in whole grains	Colon and rectum cancer
	Unsafe sex	Unsafe sex	Cervical cancer (F)
	Low physical activity	Low physical activity	Colon and rectum cancer

Groups of risk factors*	Level 2 risk factors	Specific risk factors	Cancer outcomes
Metabolic risks	High body-mass index	High body-mass index	Breast cancer (F)
		High body-mass index	Colon and rectum cancer
		High body-mass index	Oesophageal cancer
		High body-mass index	Gallbladder and biliary tract cancer
		High body-mass index	Kidney cancer
		High body-mass index	Leukaemia
		High body-mass index	Liver cancer
		High body-mass index	Multiple myeloma
		High body-mass index	Non-Hodgkin lymphoma
		High body-mass index	Ovarian cancer
		High body-mass index	Pancreatic cancer
		High body-mass index	Thyroid cancer
		High body-mass index	Uterine cancer
	High fasting plasma glucose	High fasting plasma glucose	Bladder cancer
		High fasting plasma glucose	Breast cancer (F)
		High fasting plasma glucose	Colon and rectum cancer
		High fasting plasma glucose	Liver cancer
		High fasting plasma glucose	Ovarian cancer (F)
		High fasting plasma glucose	Pancreatic cancer
		High fasting plasma glucose	Tracheal, bronchus, and lung cancer

*Level 1 groups of risk factors are estimated in the Global Burden of Disease Study with age restrictions; estimation starts at 10 years for Environmental/Occupational Risks (yellow), at 15 years for Behavioural Risks (green), and at 20 years for Metabolic Risks (blue). Further detail on age restrictions by risk factor can be found in the methods appendix to "Global burden of 87 risk factors in 204 countries and territories, 1990–2019: a systematic analysis for the Global Burden of Disease Study 2019".[4] GBD = Global Burden of Disease Study; F = female.

Appendix Table 11: GBD 2019 risk factor hierarchy, exposure definition, theoretical minimum risk exposure level, and risk-cancer pairs included in GBD 2019

GBD Level	Risk factor	Exposure definition	Theoretical minimum risk exposure level	Cancers
0	**All risks measured**	**..**	**..**	**..**
1	**Environmental and occupational risks**	**..**	**..**	**..**
2	**Air pollution**	**..**	**..**	**..**
3	Particulate matter pollution	..	..	..
4	Ambient particulate matter pollution	Annual average daily exposure to outdoor air concentrations of particulate matter with an aerodynamic diameter of ≤2·5 µm (PM2·5), measured in µg/m³	Joint theoretical minimum risk exposure level for both household and ambient particulate matter pollution is a uniform distribution between 2·4 and 5·9 µg/m³, with burden attributed proportionally between household and particulate matter pollution on the basis of source of PM2·5 exposure in excess of theoretical minimum risk exposure level	Tracheal, bronchus, and lung cancer
4	Household air pollution from solid fuels	Individual exposure to PM2·5 due to use of solid cooking fuel	See ambient particulate matter pollution	Tracheal, bronchus, and lung cancer
2	**Other environmental risks**	**..**	**..**	**..**
3	Residential radon	Average daily exposure to indoor air radon levels measured in becquerels (radon disintegrations per second) per cubic metre (Bq/m³)	10 Bq/m³, corresponding to the outdoor concentration of radon	Tracheal, bronchus, and lung cancer
2	**Occupational risks**	**..**	**..**	**..**
3	Occupational carcinogens	..	..	..
4	Occupational exposure to asbestos	Proportion of the population with cumulative lifetime exposure to occupational asbestos	No occupational exposure to asbestos	Larynx cancer<br>Tracheal, bronchus, and lung cancer<br>Ovarian cancer<br>Mesothelioma
4	Occupational exposure to arsenic	Proportion of the population ever exposed to arsenic at work or through their occupation	No occupational exposure to arsenic	Tracheal, bronchus, and lung cancer
4	Occupational exposure to benzene	Proportion of the population ever exposed to benzene at work or through their occupation	No occupational exposure to benzene	Leukaemia
4	Occupational exposure to beryllium	Proportion of the population ever exposed to beryllium at work or through their occupation	No occupational exposure to beryllium	Tracheal, bronchus, and lung cancer

GBD Level	Risk factor	Exposure definition	Theoretical minimum risk exposure level	Cancers
4	Occupational exposure to cadmium	Proportion of the population ever exposed to cadmium at work or through their occupation	No occupational exposure to cadmium	Tracheal, bronchus, and lung cancer
4	Occupational exposure to chromium	Proportion of the population ever exposed to chromium at work or through their occupation	No occupational exposure to chromium	Tracheal, bronchus, and lung cancer
4	Occupational exposure to diesel engine exhaust	Proportion of the population ever exposed to diesel engine exhaust at work or through their occupation	No occupational exposure to diesel engine exhaust	Tracheal, bronchus, and lung cancer
4	Occupational exposure to formaldehyde	Proportion of the population ever exposed to formaldehyde at work or through their occupation	No occupational exposure to formaldehyde	Nasopharynx cancer<br>Leukaemia
4	Occupational exposure to nickel	Proportion of the population ever exposed to nickel at work or through their occupation	No occupational exposure to nickel	Tracheal, bronchus, and lung cancer
4	Occupational exposure to polycyclic aromatic hydrocarbons	Proportion of the population ever exposed to polycyclic aromatic hydrocarbons at work or through their occupation	No occupational exposure to polycyclic aromatic hydrocarbons	Tracheal, bronchus, and lung cancer
4	Occupational exposure to silica	Proportion of the population ever exposed to silica at work or through their occupation	No occupational exposure to silica	Tracheal, bronchus, and lung cancer
4	Occupational exposure to sulfuric acid	Proportion of the population ever exposed to sulphuric acid at work or through their occupation	No occupational exposure to sulfuric acid	Larynx cancer
4	Occupational exposure to trichloroethylene	Proportion of the population ever exposed to trichloroethylene at work or through their occupation	No occupational exposure to trichloroethylene	Kidney cancer
1	**Behavioural risks**	..	..	..
2	**Tobacco**	..	..	..
3	Smoking	Prevalence of current use of any smoked tobacco product and prevalence of former use of any smoked tobacco product; among current smokers, cigarette equivalents smoked per smoker per day and cumulative pack-years of exposure; among former smokers, number of years since quitting	All individuals are lifelong non-smokers	Lip and oral cavity cancer<br>Nasopharynx cancer<br>Other pharynx cancer<br>Oesophageal cancer<br>Stomach cancer<br>Colon and rectum cancer<br>Liver cancer<br>Pancreatic cancer<br>Larynx cancer<br>Tracheal, bronchus, and lung cancer<br>Breast cancer<br>Cervical cancer<br>Prostate cancer<br>Kidney cancer<br>Bladder cancer<br>Leukaemia

GBD Level	Risk factor	Exposure definition	Theoretical minimum risk exposure level	Cancers
3	Chewing tobacco	Current use of any chewing tobacco product	All individuals are lifelong non-users of chewing tobacco products	Lip and oral cavity cancer<br>Oesophageal cancer
3	Secondhand smoke	Average daily exposure to air particulate matter from second-hand smoke with an aerodynamic diameter smaller than 2·5 µg, measured in µg/m³, among non-smokers	No second-hand smoke exposure	Tracheal, bronchus, and lung cancer<br>Breast cancer
2	**Alcohol use**	**Average daily alcohol consumption of pure alcohol (measured in g per day) in current drinkers who had consumed alcohol during the past 12 months**	**Estimated distribution 0–10 g per day**	Lip and oral cavity cancer<br>Nasopharynx cancer<br>Other pharynx cancer<br>Oesophageal cancer<br>Colon and rectum cancer<br>Liver cancer<br>Larynx cancer<br>Breast cancer
2	**Drug use**	**Proportion of the population dependent upon opioids, cannabis, cocaine, or amphetamines; proportion of the population who have ever injected drugs**	**No drug use**	Liver cancer
2	**Dietary risks**	**..**	**..**	**..**
3	Diet low in fruits	Average daily consumption of fruits (fresh, frozen, cooked, canned, or dried, excluding fruit juices and salted or pickled fruits)	Consumption of fruit 200–300 g per day	Oesophageal cancer<br>Tracheal, bronchus, and lung cancer
3	Diet low in vegetables	Average daily consumption of vegetables (fresh, frozen, cooked, canned, or dried, excluding legumes and salted or pickled vegetables, juices, nuts and seeds, and starchy vegetables such as potatoes or corn)	Consumption of vegetables 290–430 g per day	Oesophageal cancer
3	Diet low in whole grains	Average daily consumption of whole grains (bran, germ, and endosperm in their natural proportion) from breakfast cereals, bread, rice, pasta, biscuits, muffins, tortillas, pancakes, and other sources	Consumption of whole grains 100–150 g per day	Colon and rectum cancer
3	Diet low in milk	Average daily consumption of milk, including non-fat, low-fat, and full-fat milk, excluding soy milk and other plant derivatives	Consumption of milk 350–520 g per day	Colon and rectum cancer
3	Diet high in red meat	Average daily consumption of red meat (beef, pork, lamb, and goat but excluding poultry, fish, eggs, and all processed meats)	Consumption of red meat 18–27 g per day	Colon and rectum cancer<br>Breast cancer
3	Diet high in processed meat	Average daily consumption of meat preserved by smoking, curing, salting, or addition of chemical preservatives	Consumption of processed meat 0–4 g per day	Colon and rectum cancer

GBD Level	Risk factor	Exposure definition	Theoretical minimum risk exposure level	Cancers
3	Diet low in fibre	Average daily intake of fibre from all sources including fruits, vegetables, grains, legumes, and pulses	Consumption of fibre 19–28 g per day	Colon and rectum cancer
3	Diet low in calcium	Average daily intake of calcium from all sources, including milk, yogurt, and cheese	Consumption of calcium 1·0–1·5 g per day	Colon and rectum cancer
3	Diet high in sodium	24-h urinary sodium measured in g per day	24-h urinary sodium 1–5 g per day	Stomach cancer
2	**Unsafe sex**	**Proportion of the population with exposure to sexual encounters that convey the risk of disease**	**No exposure to disease-causing pathogen through sex**	**Cervical cancer**
2	**Low physical activity**	**Average weekly physical activity at work, home, transport-related and recreational measured by MET min per week**	**All adults experience 3000–4500 MET min per week**	**Colon and rectum cancer**<br>**Breast cancer**
1	**Metabolic risks**	**..**	**..**	**..**
2	**High fasting plasma glucose**	**Serum fasting plasma glucose measured in mmol/L**	**4·8–5·4 mmol/L**	**Colon and rectum cancer**<br>**Pancreatic cancer**<br>**Tracheal, bronchus, and lung cancer**<br>**Breast cancer**<br>**Ovarian cancer**<br>**Bladder cancer**
2	**High body-mass index**	**Body-mass index, measured in kg/m²**	**20–25 kg/m²**	**Oesophageal cancer**<br>**Colon and rectum cancer**<br>**Liver cancer**<br>**Gallbladder and biliary tract cancer**<br>**Pancreatic cancer**<br>**Breast cancer**<br>**Uterine cancer**<br>**Ovarian cancer**<br>**Kidney cancer**<br>**Thyroid cancer**<br>**Non-Hodgkin lymphoma**<br>**Multiple myeloma**<br>**Leukaemia**

GBD = Global Burden of Disease Study; PM2.5 = particulate matter $\leq$ 2.5 micrometres; MET = Metabolic Equivalent; GBD = Global Burden of Disease Study.

Additional Results in Tables and Figures

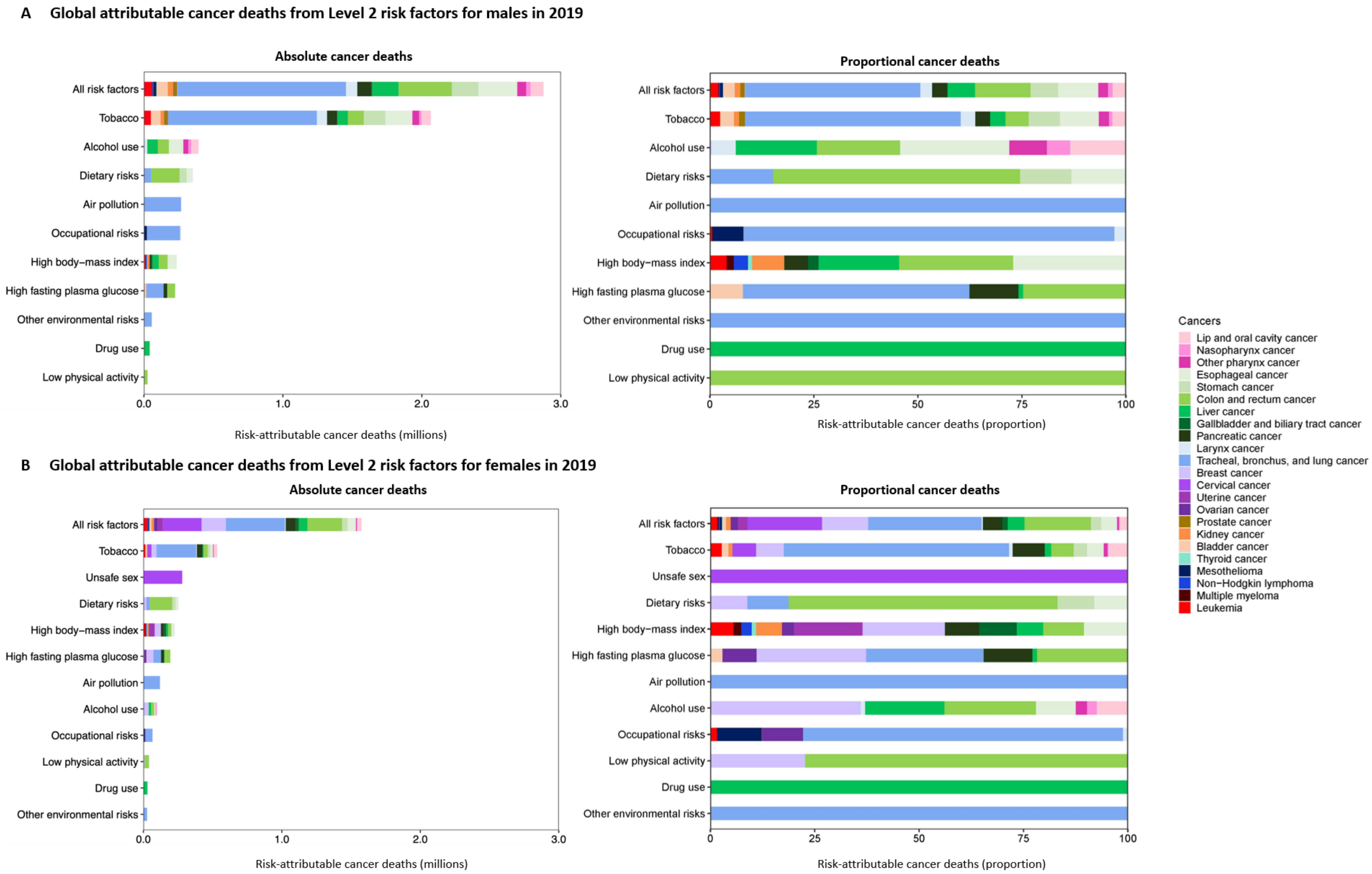

Appendix Figure 7: Global absolute and proportional cancer deaths attributable to Level 2 risk factors for (A) males and (B) females in 2019. “Air pollution” includes ambient particulate matter pollution and household air pollution from solid fuels. “Other environmental risks” include residential radon. “Occupational risks” include exposure to thirteen specific carcinogens. “Dietary risks” include nine specific risk factors relevant to cancer. “Tobacco” includes smoking, chewing tobacco, and secondhand smoke.

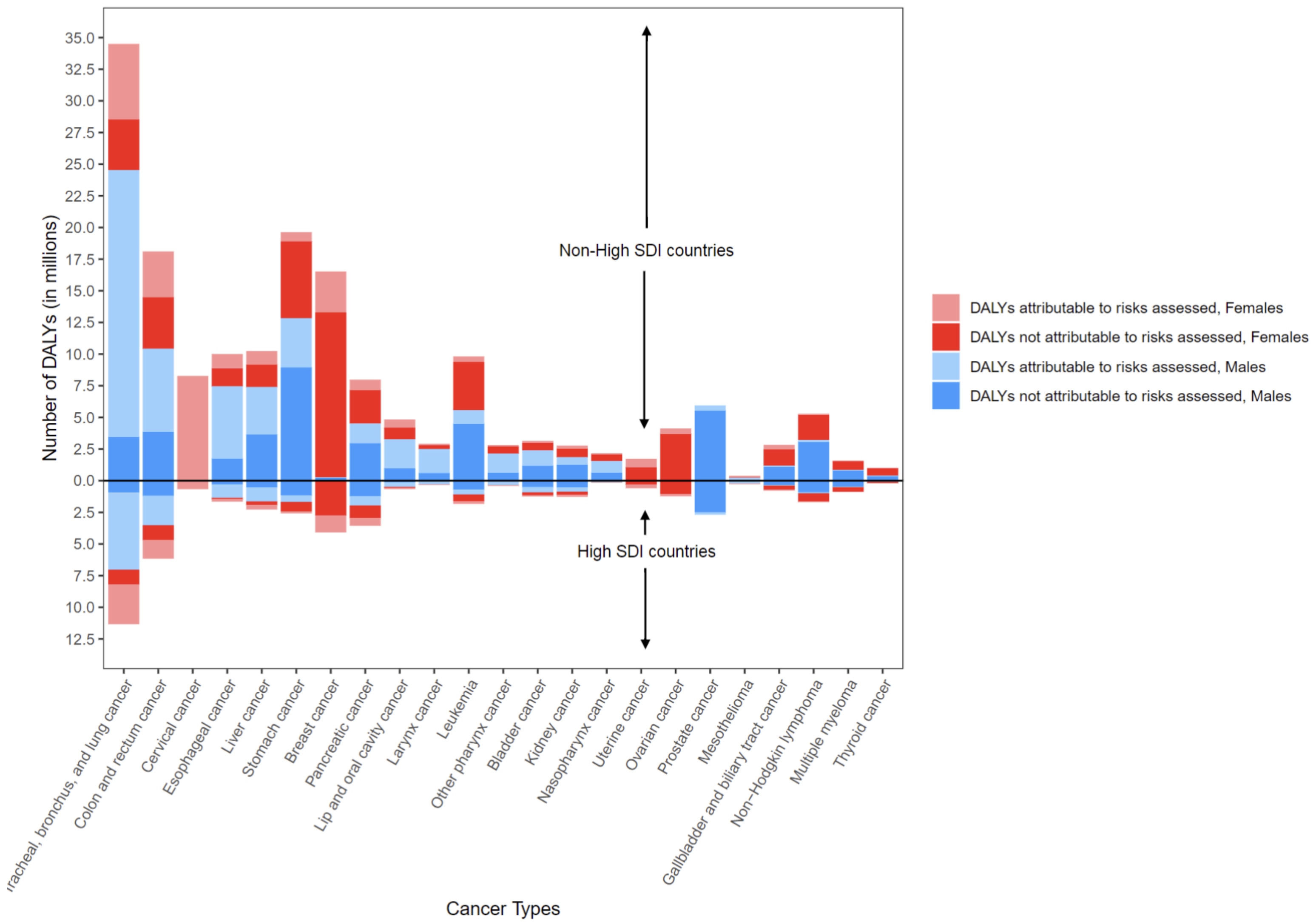

Appendix Figure 8: DALYs from cancers attributable to risk factors in 2019 by sex and SDI. Non-high SDI countries include low, low-middle, middle, and high-middle SDI countries. Cancer types are listed from left to right in order of greatest to least risk-attributable DALYs. DALY = disability-adjusted life-year; SDI = Socio-demographic Index.

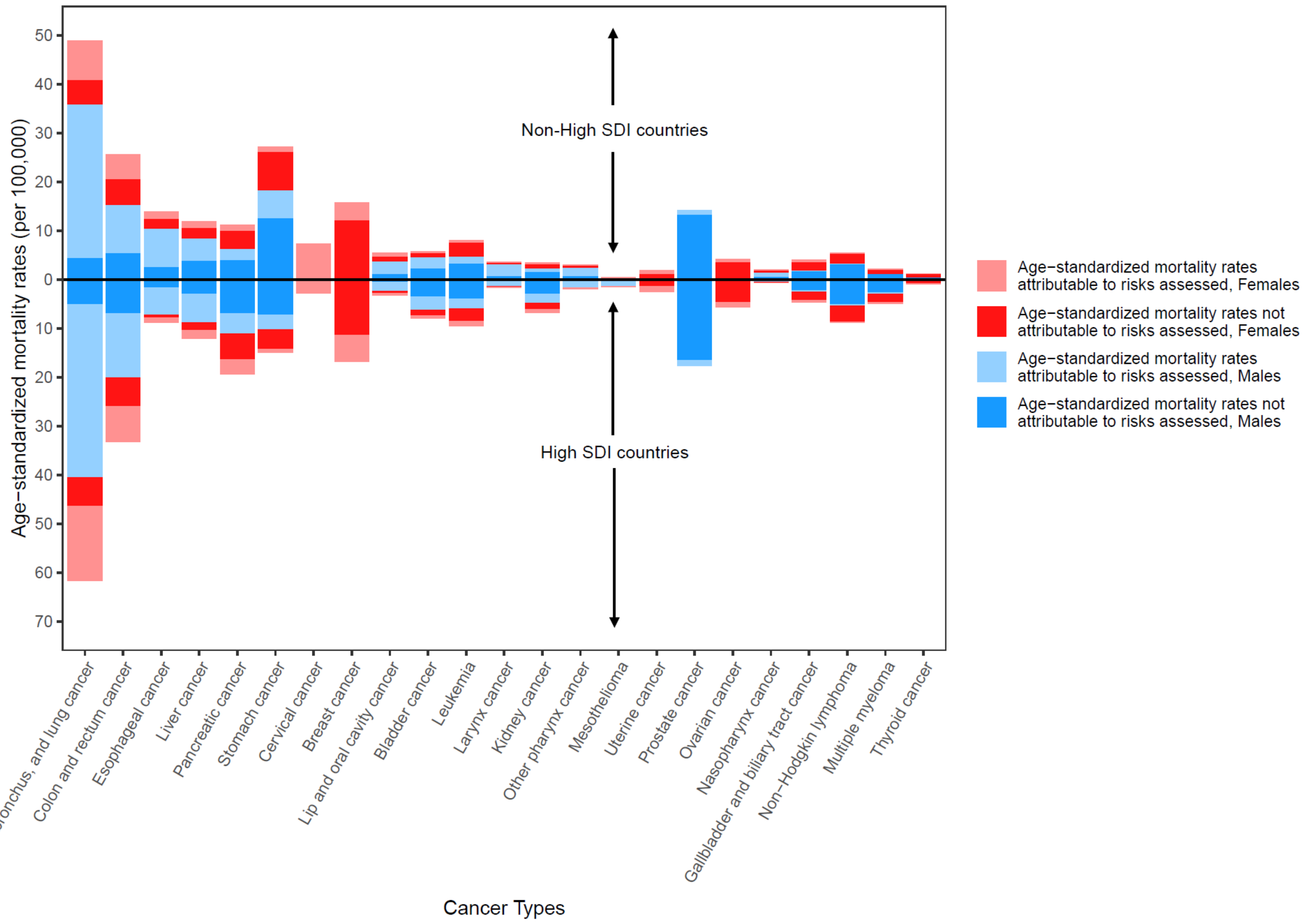

Appendix Figure 9: Age-standardised mortality rates from cancers attributable to risk factors in 2019 by sex and SDI. Non-high SDI countries include low, low-middle, middle, and high-middle SDI countries. Cancer types are listed from left to right in order of greatest to least risk-attributable age-standardised mortality rates. Further details on age-standardised mortality rates by sex and SDI can be found in Appendix Tables 20 & 22. SDI = Socio-demographic Index.

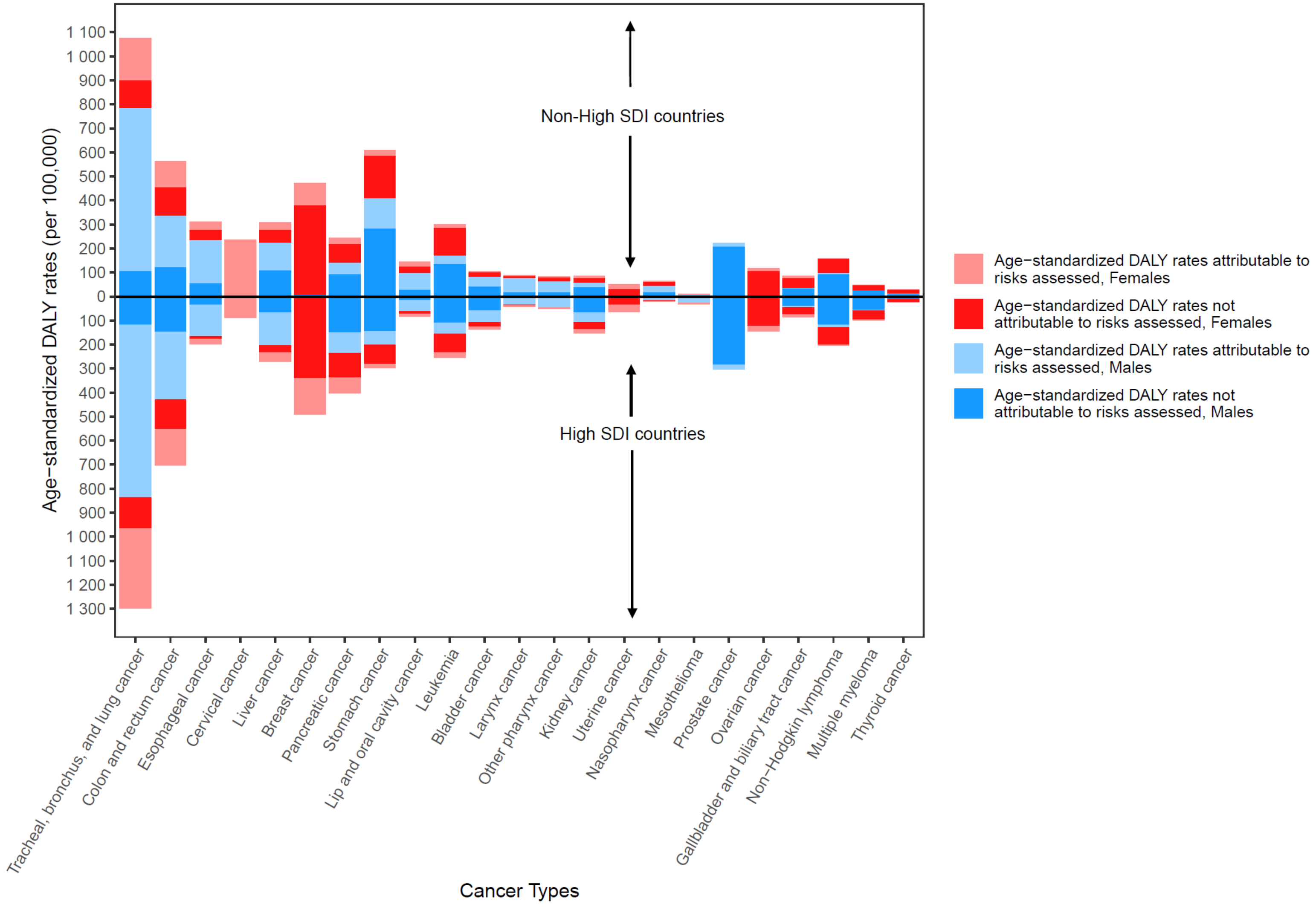

Appendix Figure 10: Age-standardised DALY rates from cancers attributable to risk factors in 2019 by sex and SDI. Non-high SDI countries include low, low-middle, middle, and high-middle SDI countries. Cancer types are listed from left to right in order of greatest to least risk-attributable age-standardised DALY rates. DALY = disability-adjusted life-year; SDI = Socio-demographic Index.

A. Environmental and occupational risks

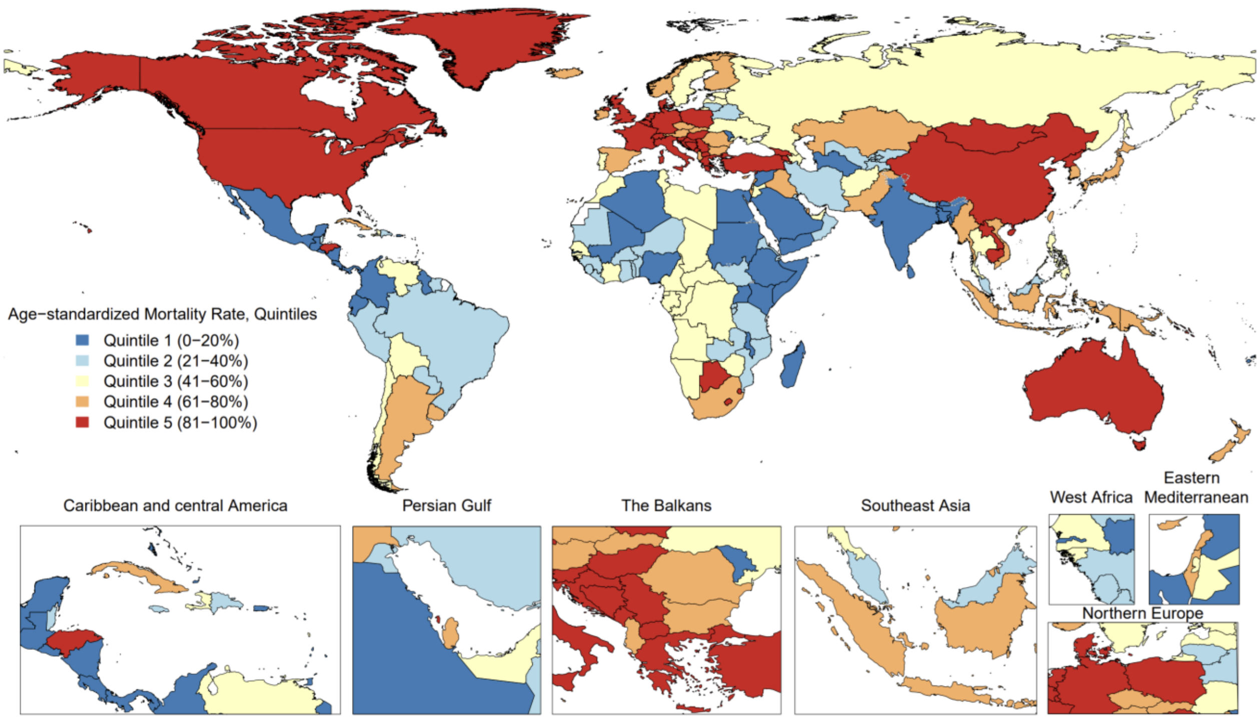

B. Behavioural risks

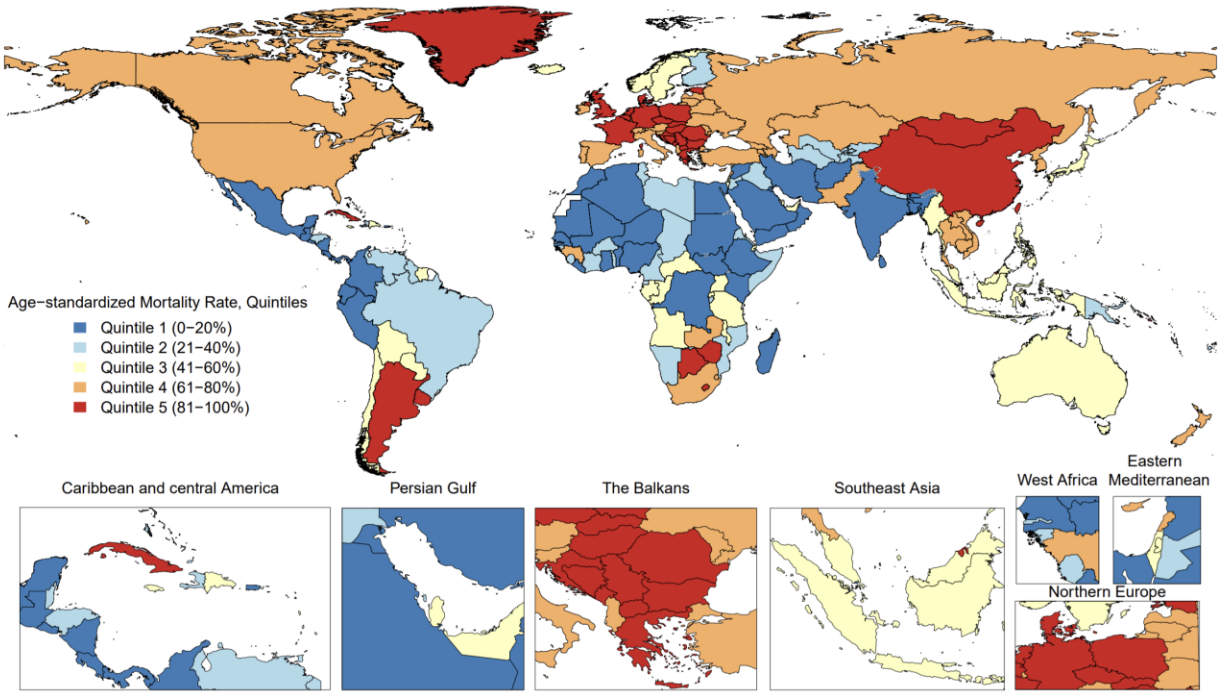

C. Metabolic risks

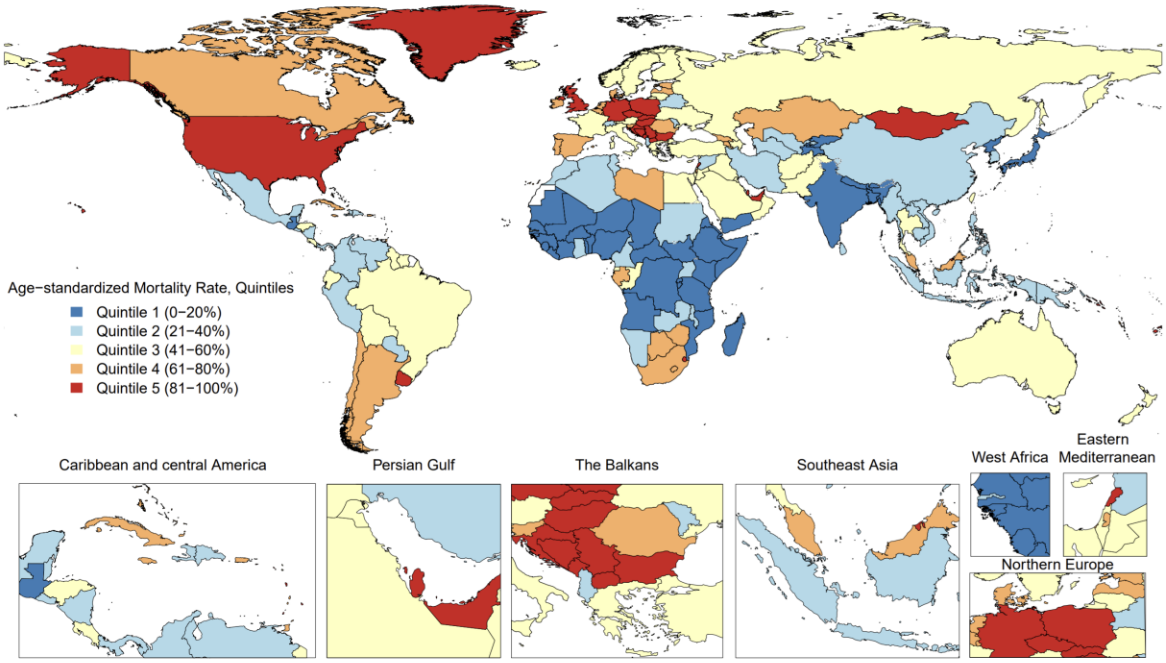

Appendix Figure 11: Global map of age-standardised mortality rate quintiles for cancer burden attributable to (A) environmental and occupational risks, (B) behavioural risks, and (C) metabolic risks, both sexes combined, 2019. Each map represents estimates at the national level. Quintiles are based on age-standardised mortality rates per 100,000 person-years. For panel (A) quintile 1: less than 3.8, quintile 2: 3.8 to <5.2, quintile 3: 5.2 to <6.8, quintile 4: 6.8 to <9.6, quintile 5: 9.6 and greater. For panel (B) quintile 1: less than 28.1, quintile 2: 28.1 to <35.8, quintile 3: 35.8 to <41.5, quintile 4: 41.5 to <53.4, quintile 5: 53.4 and greater. For panel (C) quintile 1: less than 7.6, quintile 2: 7.6 to <10.5, quintile 3: 10.5 to <13.5, quintile 4: 13.5 to <17.1, quintile 5: 17.1 and greater. There are several geographic locations where estimates are not available (eg, Western Sahara, French Guiana) as they were not modelled locations in the Global Burden of Diseases, Injuries, and Risk Factors 2019 study; these locations are white in this map.

A. Environmental and occupational risks

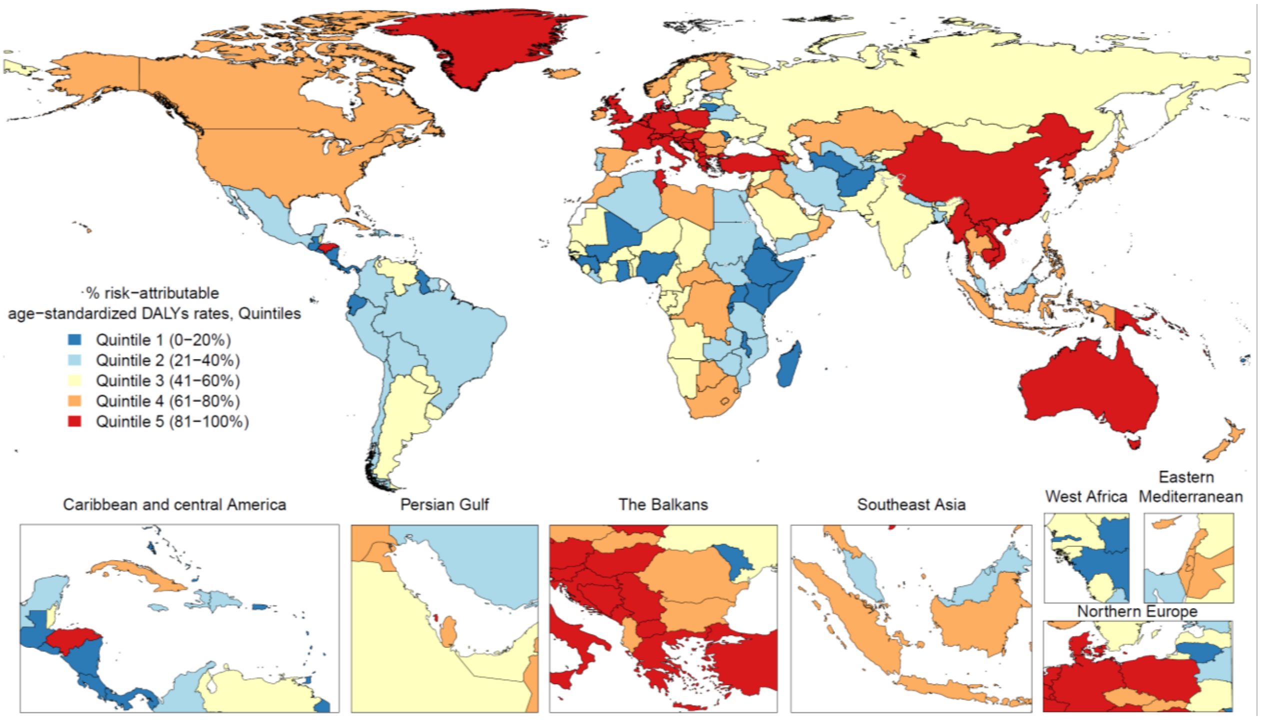

B. Behavioural risks

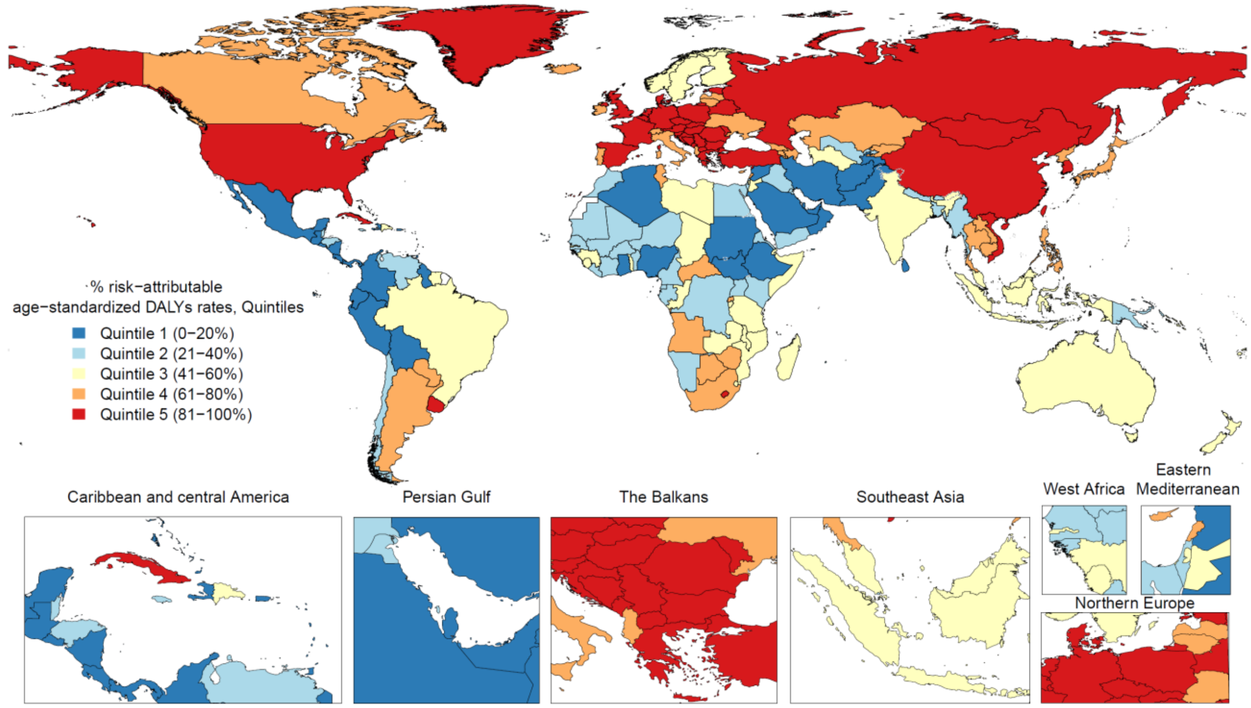

C. Metabolic risks

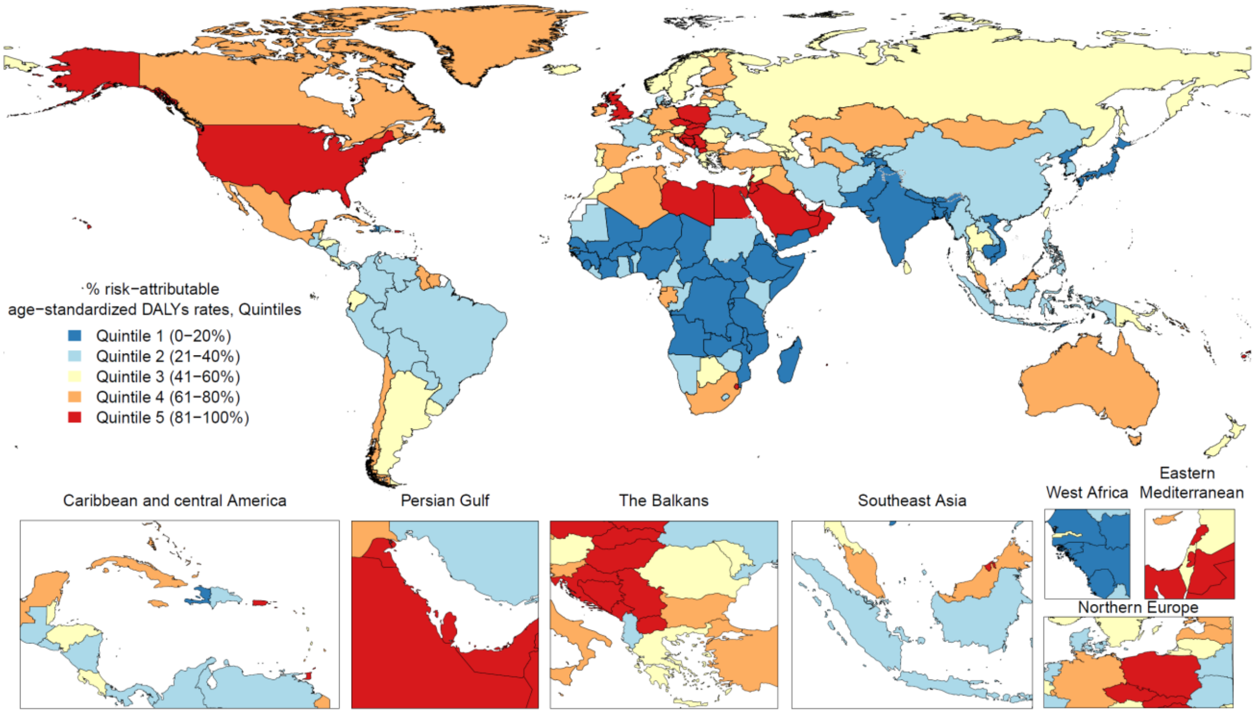

Appendix Figure 12: Global map of proportion of risk-attributable cancer age-standardised DALY rates over total cancer age-standardised DALY rates, by quintile for cancer burden attributable to (A) environmental and occupational risks, (B) behavioural risks, and (C) metabolic risks, both sexes combined, 2019. Each map represents estimates at the national level. Quintiles are based on the proportions: (*risk-attributable cancer age-standardised DALY rates per 100,000 person-years*) / (*total cancer age-standardised DALY rates per 100,000 person-years*). For panel (A) quintile 1: less than 3.3, quintile 2: 3.3 to <4.0, quintile 3: 4.0 to <4.9, quintile 4: 4.9 to <6.7, quintile 5: 6.7 and greater. For panel (B) quintile 1: less than 25.5, quintile 2: 25.5 to <29.1, quintile 3: 29.1 to <32.1, quintile 4: 32.1 to <36.5, quintile 5: 36.5 and greater. For panel (C) quintile 1: less than 5.9, quintile 2: 5.9 to <8.3, quintile 3: 8.3 to <9.3, quintile 4: 9.3 to <11.1, quintile 5: 11.1 and greater. There are several geographic locations where estimates are not available (eg, Western Sahara, French Guiana) as they were not modelled locations in the Global Burden of Diseases, Injuries, and Risk Factors 2019 study; these locations are white in this map.

A. Environmental and occupational risks

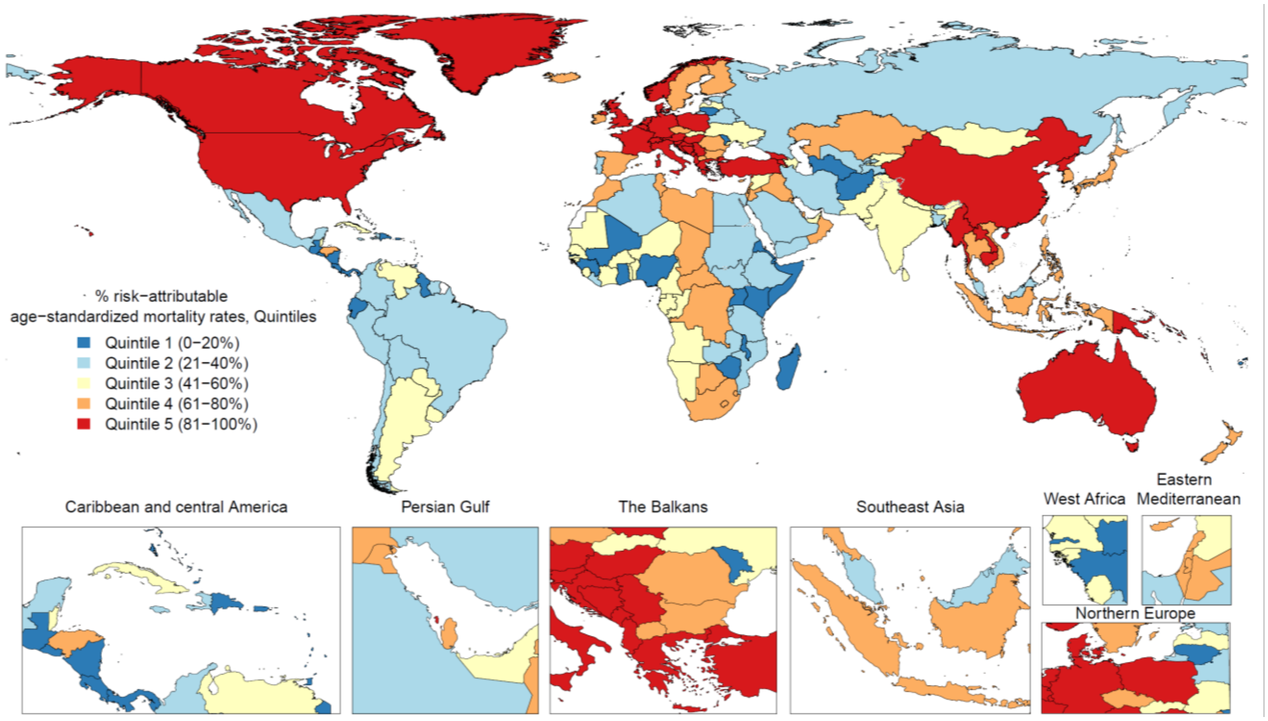

B. Behavioural risks

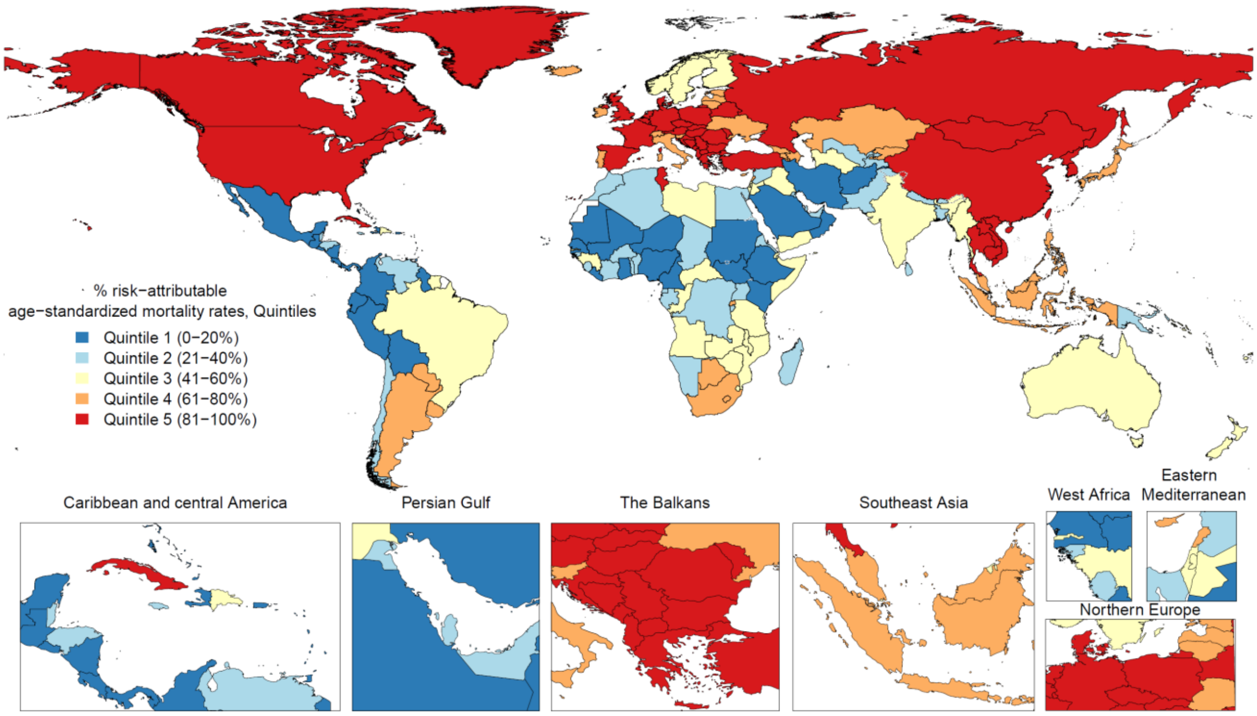

C. Metabolic risks

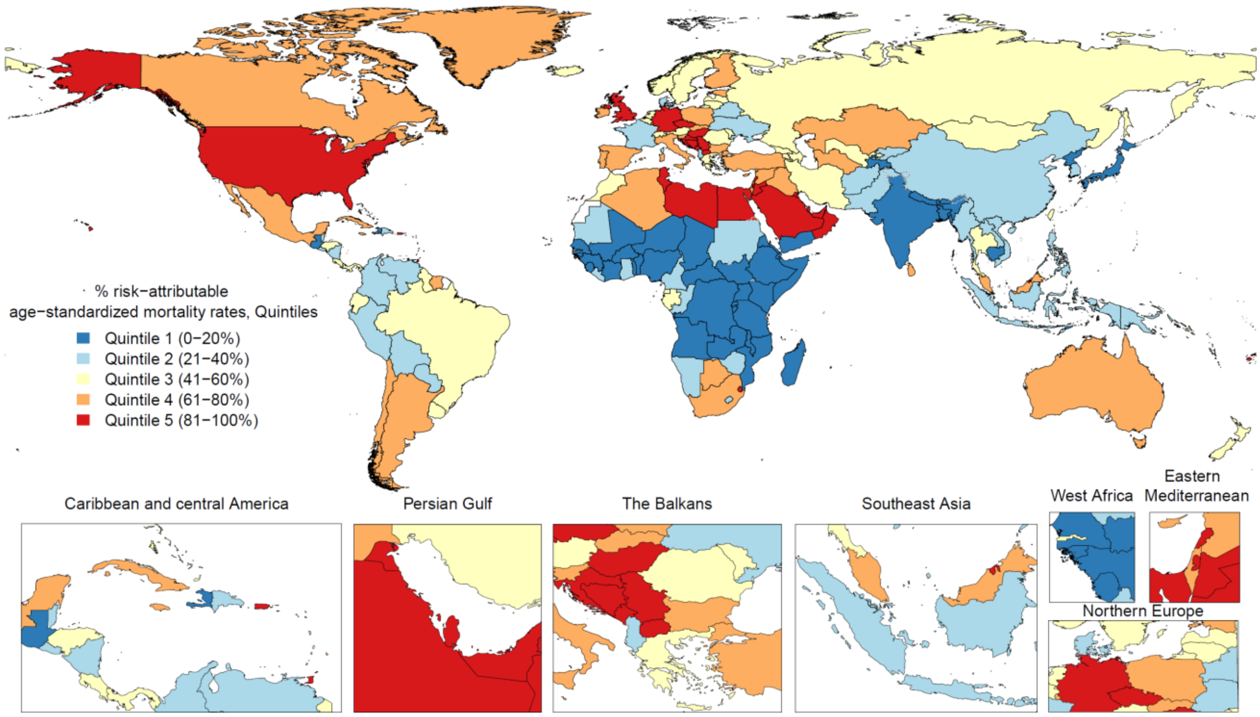

Appendix Figure 13: Global map of proportion of risk-attributable cancer age-standardised mortality rates over total cancer age-standardised mortality rates, by quintile for cancer burden attributable to (A) environmental and occupational risks, (B) behavioural risks, and (C) metabolic risks, both sexes combined, 2019. Each map represents estimates at the national level. Quintiles are based on the proportions: (*risk-attributable cancer age-standardised mortality rates per 100,000 person-years*) / (*total cancer age-standardised mortality rates per 100,000 person-years*). For panel (A) quintile 1: less than 3.5, quintile 2: 3.5 to <4.3, quintile 3: 4.3 to <5.2, quintile 4: 5.2 to <7.4, quintile 5: 7.4 and greater. For panel (B) quintile 1: less than 25.6, quintile 2: 25.6 to <29.3, quintile 3: 29.3 to <32.9, quintile 4: 32.9 to <37.6, quintile 5: 37.6 and greater. For panel (C) quintile 1: less than 6.7, quintile 2: 6.7 to <9.0, quintile 3: 9.0 to <10.3, quintile 4: 10.3 to <12.3, quintile 5: 12.3 and greater. There are several geographic locations where estimates are not available (eg, Western Sahara, French Guiana) as they were not modelled locations in the Global Burden of Diseases, Injuries, and Risk Factors 2019 study; these locations are white in this map.

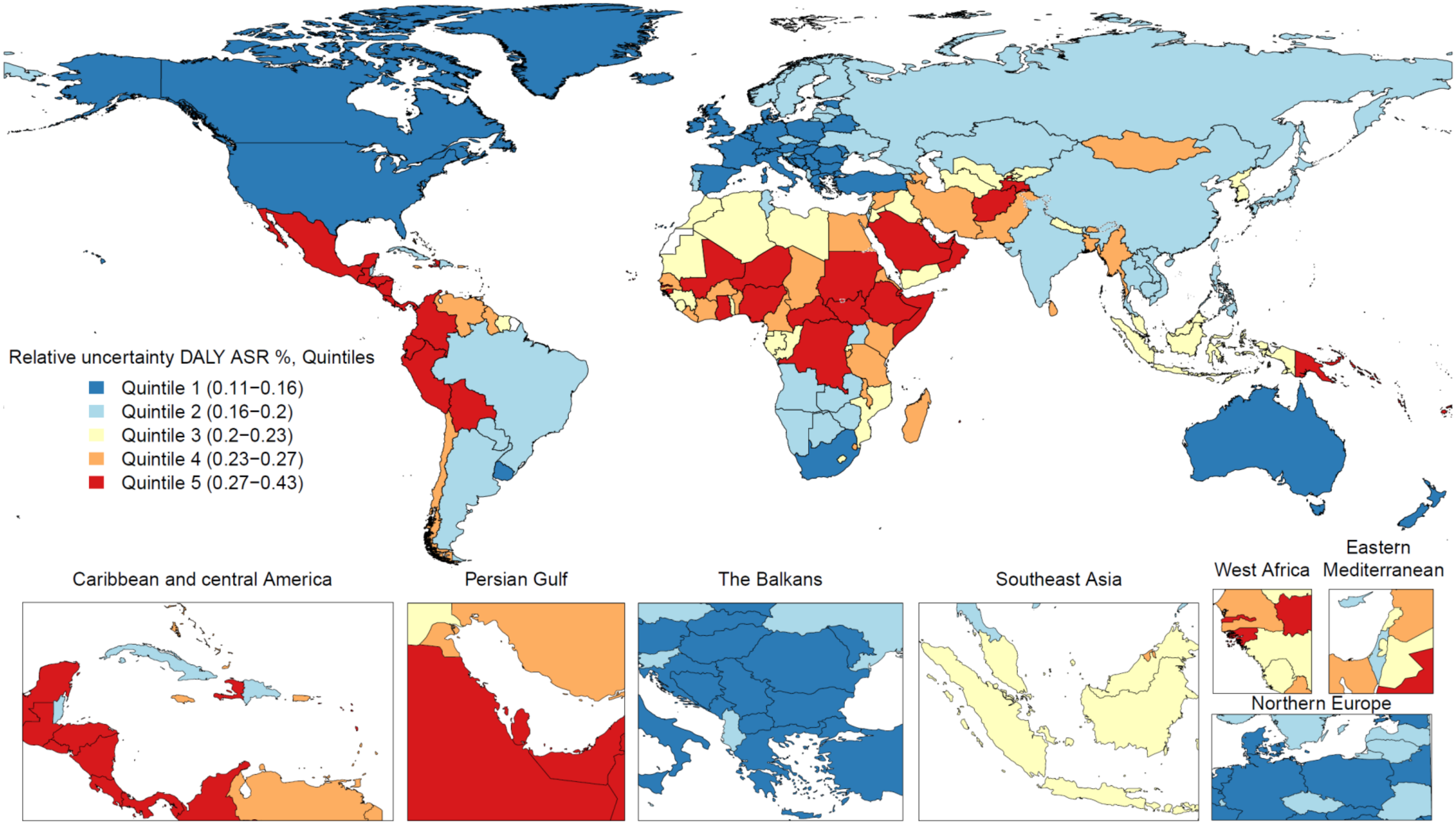

Appendix Figure 14: Relative uncertainty in the percent of risk-attributable cancer age-standardised DALY rates over total cancer (risk + non-risk) age-standardised DALY rates, by quintile, for both sexes combined in 2019. There are several geographic locations where estimates are not available (eg, Western Sahara, French Guiana) as they were not modelled locations in the Global Burden of Diseases, Injuries, and Risk Factors 2019 study; these locations are white in this map. DALY = disability-adjusted life-year; ASR = age-standardised rate.

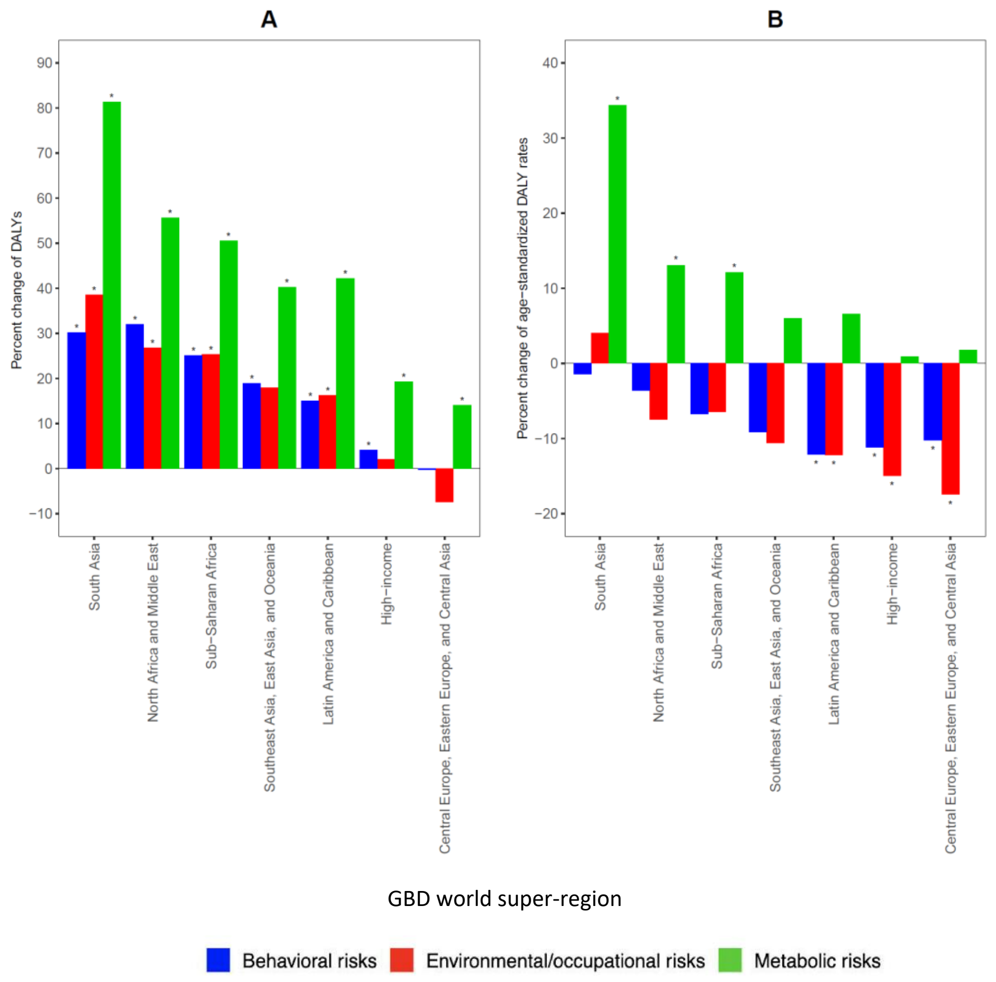

Appendix Figure 15: Percent change of attributable A. cancer DALY counts and B. age-standardised DALY rates for Level 1 risk factors by GBD super-regions, both sexes combined, 2010 - 2019. In panels A and B, each bar was color-coded by a corresponding Level 1 risk factor. * indicate 95% uncertainty intervals that do not include zero. DALYs = disability-adjusted life-years; SDI = Socio-demographic Index; GBD = Global Burden of Disease, Injuries, and Risks Factors Study.

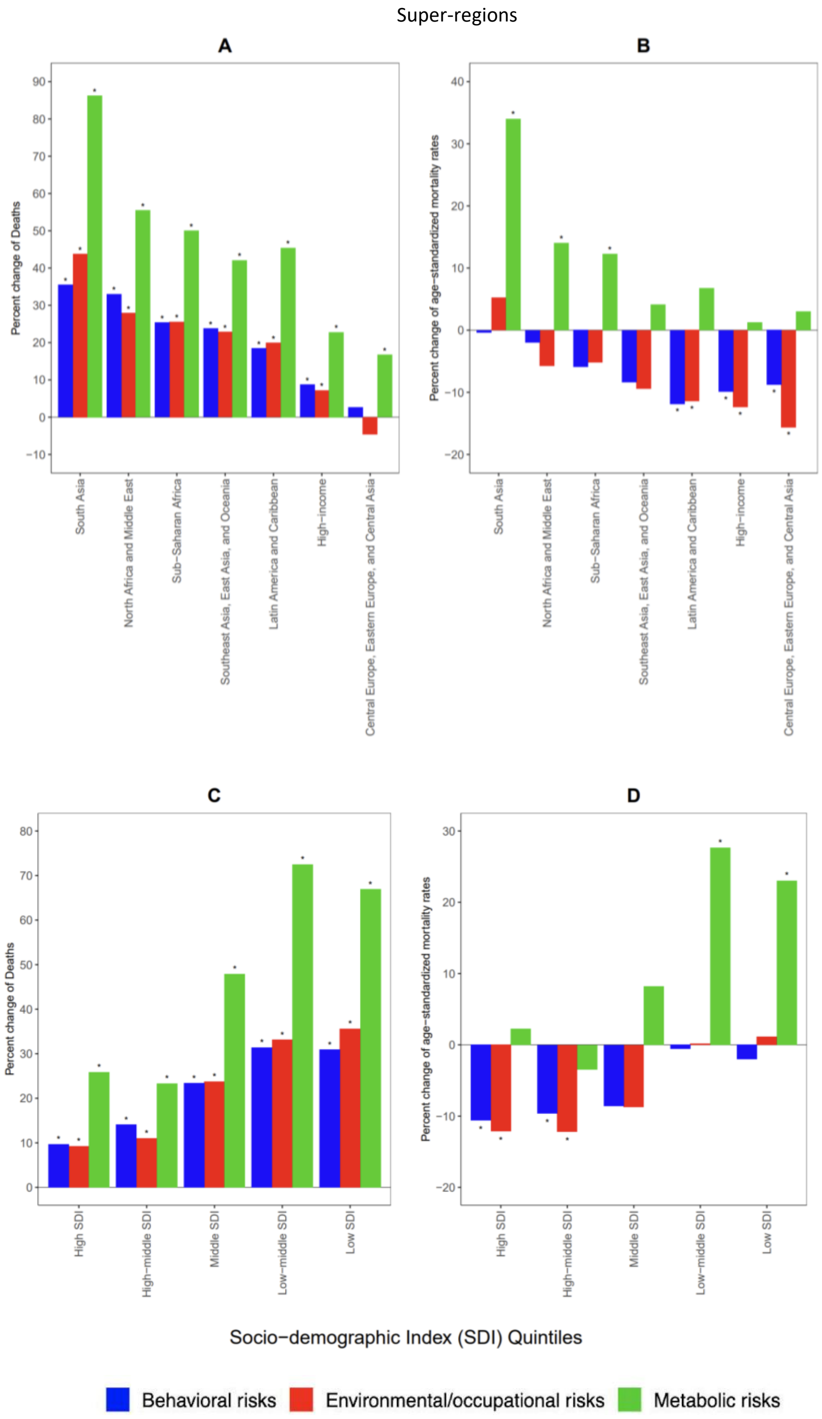

Appendix Figure 16: Percent change of attributable cancer deaths and age-standardised mortality rates for Level 1 risk factors by GBD super-regions and SDI quintiles, both sexes combined, 2010 - 2019. In panels A and B, each bar was color-coded by a corresponding Level 1 risk factor and indicates percentage of attributable cancer deaths (panel A) or percentage change of age-standardised mortality rates (panel B) for a GBD world super-region. In panels C and D, each bar indicates percentage of attributable cancer deaths (panel C) or percentage change of age-standardised mortality rates (panel D) for an SDI quintile. * indicate 95% uncertainty intervals that do not include zero. SDI = Socio-demographic Index. GBD = Global Burden of Disease, Injuries, and Risks Factors Study.

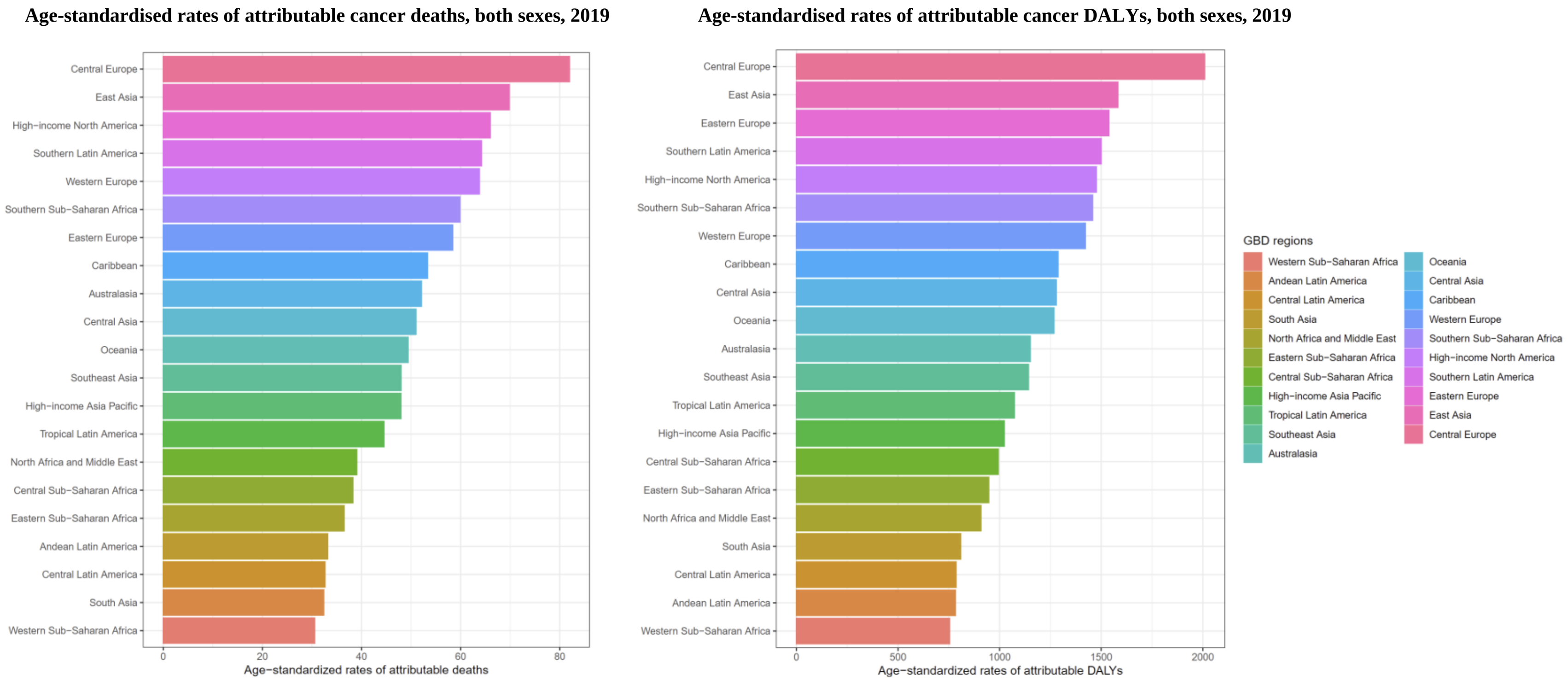

Appendix Figure 17: Regional age-standardised rates of attributable cancer deaths and DALYs, both sexes, 2019. GBD = Global Burden of Disease Study; DALYs = disability-adjusted life-years.

A Global

Males

Leading risk 2010	ASR of DALYs 2010	Leading risk 2019	ASR of DALYs 2019	Percentage change in ASR of DALYs 2010-2019
1 Smoking	1 356.4 (1 274.0 to 1 436.6)	1 Smoking	1 184.6 (1 067.6 to 1 310.8)	-12.7 (-20.9 to -3.8)
2 Alcohol use	272.0 (243.9 to 301.8)	2 Alcohol use	259.9 (227.8 to 292.9)	-4.5 (-13.4 to 4.5)
3 High body-mass index	141.1 (68.6 to 237.8)	3 High body-mass index	150.7 (77.1 to 247.5)	6.8 (-2.5 to 17.6)
4 Ambient particulate matter pollution	133.8 (97.5 to 169.8)	4 Ambient particulate matter pollution	126.5 (91.7 to 164.2)	-5.5 (-18.2 to 9.7)
5 High fasting plasma glucose	121.1 (29.6 to 257.5)	5 High fasting plasma glucose	120.4 (29.5 to 257.7)	-0.5 (-7.8 to 7.8)
6 Occupational exposure to asbestos	115.6 (81.2 to 150.7)	6 Occupational exposure to asbestos	93.8 (65.9 to 123.0)	-18.9 (-24.0 to -13.7)
7 Diet low in fruits	65.1 (32.6 to 105.1)	7 Diet low in whole grains	57.1 (21.9 to 75.4)	-3.0 (-10.0 to 4.5)
8 Diet low in whole grains	58.9 (22.6 to 77.2)	8 Diet low in milk	56.5 (36.6 to 77.2)	1.7 (-7.2 to 11.3)
9 Diet low in milk	55.5 (36.0 to 74.8)	9 Diet low in fruits	52.8 (26.5 to 84.6)	-18.9 (-28.6 to -7.9)
10 Diet low in calcium	51.5 (38.0 to 68.6)	10 Diet low in calcium	48.8 (34.9 to 66.1)	-5.1 (-14.2 to 3.9)

Females

Leading risk 2010	ASR of DALYs 2010	Leading risk 2019	ASR of DALYs 2019	Percentage change in ASR of DALYs 2010-2019
1 Smoking	254.2 (235.4 to 275.1)	1 Smoking	222.9 (202.1 to 243.5)	-12.3 (-16.6 to -7.4)
2 Unsafe sex	221.4 (194.6 to 246.1)	2 Unsafe sex	210.6 (177.7 to 234.9)	-4.8 (-12.8 to 3.7)
3 High body-mass index	114.9 (67.4 to 175.6)	3 High body-mass index	117.8 (71.3 to 175.0)	2.4 (-4.0 to 10.6)
4 High fasting plasma glucose	85.1 (22.6 to 175.0)	4 High fasting plasma glucose	91.0 (24.8 to 192.1)	6.9 (0.2 to 14.3)
5 Alcohol use	64.6 (57.4 to 72.4)	5 Alcohol use	58.3 (51.4 to 65.9)	-9.9 (-14.6 to -4.5)
6 Ambient particulate matter pollution	43.8 (31.3 to 55.7)	6 Ambient particulate matter pollution	46.3 (33.5 to 60.2)	5.8 (-8.2 to 22.1)
7 Secondhand smoke	39.2 (25.6 to 54.9)	7 Secondhand smoke	39.0 (25.5 to 55.9)	-0.7 (-11.5 to 11.8)
8 Diet low in whole grains	38.4 (14.7 to 50.4)	8 Diet low in milk	36.8 (23.3 to 49.0)	1.7 (-6.3 to 10.4)
9 Diet low in milk	36.2 (23.5 to 49.3)	9 Diet low in whole grains	36.6 (14.2 to 48.8)	-4.6 (-10.8 to 1.8)
10 Diet low in calcium	30.1 (21.7 to 41.0)	10 Diet low in calcium	28.7 (20.5 to 39.2)	-4.9 (-12.8 to 3.2)

Both sexes combined

Leading risk 2010	ASR of DALYs 2010	Leading risk 2019	ASR of DALYs 2019	Percentage change in ASR of DALYs 2010-2019
1 Smoking	774.1 (729.9 to 818.1)	1 Smoking	677.3 (616.4 to 740.3)	-12.5 (-19.6 to -4.8)
2 Alcohol use	164.4 (148.3 to 182.3)	2 Alcohol use	155.2 (138.4 to 173.5)	-5.6 (-12.9 to 2.2)
3 High body-mass index	127.9 (71.4 to 200.3)	3 High body-mass index	133.9 (76.2 to 206.8)	4.8 (-1.8 to 12.9)
4 Unsafe sex	112.6 (99.2 to 125.4)	4 Unsafe sex	107.2 (90.5 to 119.4)	-4.8 (-12.7 to 3.7)
5 High fasting plasma glucose	101.3 (27.4 to 207.0)	5 High fasting plasma glucose	104.2 (28.7 to 212.9)	2.9 (-2.8 to 9.5)
6 Ambient particulate matter pollution	86.3 (63.0 to 109.0)	6 Ambient particulate matter pollution	84.2 (62.1 to 108.3)	-2.4 (-12.5 to 10.1)
7 Occupational exposure to asbestos	61.1 (45.0 to 77.6)	7 Occupational exposure to asbestos	50.9 (37.8 to 64.7)	-16.7 (-21.8 to -11.5)
8 Diet low in whole grains	48.1 (18.4 to 63.0)	8 Diet low in whole grains	46.3 (17.8 to 61.1)	-3.6 (-9.1 to 1.9)
9 Diet low in milk	45.3 (29.4 to 61.2)	9 Diet low in milk	46.1 (29.8 to 62.2)	1.7 (-4.8 to 8.9)
10 Diet low in fruits	43.6 (22.1 to 68.7)	10 Secondhand smoke	38.5 (24.8 to 55.5)	-5.2 (-13.7 to 4.0)
11 Secondhand smoke	40.7 (26.6 to 57.9)	12 Diet low in fruits	36.0 (18.5 to 56.2)	-17.5 (-26.5 to -8.1)

B High SDI

Males

Leading risk 2010	ASR of DALYs 2010	Leading risk 2019	ASR of DALYs 2019	Percentage change in ASR of DALYs 2010-2019
1 Smoking	1 364.9 (1 299.9 to 1 423.3)	1 Smoking	1 126.5 (1 062.1 to 1 183.7)	-17.5 (-19.6 to -15.3)
2 Alcohol use	295.3 (269.7 to 322.6)	2 Alcohol use	273.3 (248.0 to 298.9)	-7.5 (-11.1 to -3.7)
3 Occupational exposure to asbestos	260.4 (189.7 to 329.6)	3 Occupational exposure to asbestos	215.6 (156.0 to 274.1)	-17.2 (-22.5 to -11.3)
4 High body-mass index	209.6 (112.6 to 325.3)	4 High body-mass index	209.9 (116.7 to 321.5)	0.1 (-3.6 to 5.6)
5 High fasting plasma glucose	163.2 (40.6 to 343.0)	5 High fasting plasma glucose	165.4 (41.7 to 345.7)	1.3 (-2.5 to 7.1)
6 Ambient particulate matter pollution	88.5 (61.0 to 123.3)	6 Diet low in whole grains	67.6 (25.1 to 88.6)	-7.0 (-9.6 to -4.1)
7 Diet low in whole grains	72.7 (27.3 to 96.3)	7 Ambient particulate matter pollution	65.6 (44.0 to 93.1)	-25.9 (-31.7 to -20.4)
8 Diet low in fruits	56.2 (25.8 to 88.4)	8 Diet low in milk	51.6 (28.8 to 76.2)	-7.3 (-11.0 to -3.3)
9 Diet low in milk	55.6 (30.9 to 81.0)	9 Diet low in fruits	46.8 (21.2 to 74.8)	-16.7 (-21.1 to -12.4)
10 Residential radon	41.5 (8.3 to 79.7)	10 Drug use	38.1 (30.7 to 47.9)	-0.5 (-10.4 to 7.4)
12 Drug use	38.4 (30.8 to 47.7)	11 Residential radon	35.2 (6.9 to 67.6)	-15.3 (-18.3 to -12.6)

Females

Leading risk 2010	ASR of DALYs 2010	Leading risk 2019	ASR of DALYs 2019	Percentage change in ASR of DALYs 2010-2019
1 Smoking	523.5 (492.8 to 554.5)	1 Smoking	467.2 (436.1 to 497.7)	-10.8 (-13.0 to -8.5)
2 High body-mass index	139.9 (87.9 to 203.8)	2 High body-mass index	140.6 (89.7 to 201.2)	0.5 (-2.6 to 4.6)
3 Alcohol use	129.9 (114.2 to 145.2)	3 High fasting plasma glucose	123.4 (33.5 to 252.8)	6.6 (2.9 to 11.5)
4 High fasting plasma glucose	115.7 (31.2 to 237.4)	4 Alcohol use	118.1 (103.3 to 132.6)	-9.0 (-13.3 to -5.0)
5 Unsafe sex	96.4 (90.0 to 100.5)	5 Unsafe sex	89.7 (81.9 to 95.8)	-7.0 (-10.7 to -2.8)
6 Diet low in whole grains	45.1 (16.9 to 59.5)	6 Diet low in whole grains	42.6 (15.9 to 56.5)	-5.6 (-8.4 to -2.7)
7 Diet high in red meat	43.7 (25.9 to 61.7)	7 Diet high in red meat	41.2 (24.2 to 58.9)	-5.8 (-10.2 to -1.1)
8 Ambient particulate matter pollution	39.9 (27.0 to 56.2)	8 Diet low in milk	32.3 (17.7 to 47.7)	-5.5 (-9.1 to -1.6)
9 Diet low in milk	34.2 (18.8 to 50.4)	9 Ambient particulate matter pollution	31.5 (20.0 to 46.0)	-21.2 (-28.0 to -15.9)
10 Occupational exposure to asbestos	31.8 (21.2 to 41.6)	10 Occupational exposure to asbestos	30.1 (19.8 to 40.3)	-5.3 (-14.7 to 4.2)

Both sexes combined

Leading risk 2010	ASR of DALYs 2010	Leading risk 2019	ASR of DALYs 2019	Percentage change in ASR of DALYs 2010-2019
1 Smoking	911.0 (868.5 to 949.8)	1 Smoking	776.0 (731.4 to 817.1)	-14.8 (-16.8 to -12.9)
2 Alcohol use	207.8 (189.8 to 226.7)	2 Alcohol use	192.2 (173.9 to 210.5)	-7.5 (-10.9 to -4.0)
3 High body-mass index	173.9 (103.5 to 258.1)	3 High body-mass index	174.7 (104.3 to 254.9)	0.5 (-2.6 to 4.7)
4 High fasting plasma glucose	136.8 (37.6 to 274.3)	4 High fasting plasma glucose	142.4 (40.1 to 284.6)	4.1 (0.4 to 8.8)
5 Occupational exposure to asbestos	133.1 (99.4 to 165.4)	5 Occupational exposure to asbestos	114.1 (85.8 to 141.8)	-14.2 (-19.9 to -8.2)
6 Ambient particulate matter pollution	62.3 (42.8 to 87.0)	6 Diet low in whole grains	54.4 (20.2 to 71.4)	-6.1 (-8.6 to -3.5)
7 Diet low in whole grains	57.9 (21.7 to 76.6)	7 Ambient particulate matter pollution	47.4 (31.3 to 67.7)	-23.9 (-29.9 to -18.7)
8 Unsafe sex	49.3 (46.1 to 51.4)	8 Unsafe sex	45.3 (41.2 to 48.5)	-8.1 (-11.8 to -3.9)
9 Diet low in milk	44.1 (24.4 to 64.5)	9 Diet low in milk	41.4 (22.9 to 61.1)	-6.2 (-9.5 to -2.6)
10 Diet low in fruits	38.4 (17.2 to 59.2)	10 Diet high in red meat	34.8 (18.4 to 53.4)	-5.0 (-8.9 to -0.3)
11 Diet high in red meat	36.7 (18.9 to 56.6)	11 Diet low in fruits	33.2 (15.2 to 51.3)	-13.5 (-17.1 to -10.0)

C High-middle SDI

Males

Leading risk 2010	ASR of DALYs 2010	Leading risk 2019	ASR of DALYs 2019	Percentage change in ASR of DALYs 2010-2019
1 Smoking	1 832.8 (1 713.2 to 1 948.9)	1 Smoking	1 574.1 (1 396.4 to 1 760.7)	-14.1 (-24.1 to -2.9)
2 Alcohol use	346.3 (308.7 to 386.0)	2 Alcohol use	312.4 (268.9 to 357.8)	-9.8 (-20.1 to 1.0)
3 Ambient particulate matter pollution	213.4 (159.6 to 269.0)	3 High body-mass index	190.6 (98.9 to 311.7)	3.9 (-7.1 to 15.7)
4 High body-mass index	183.4 (91.6 to 303.6)	4 Ambient particulate matter pollution	184.6 (132.6 to 240.8)	-13.5 (-27.7 to 2.7)
5 High fasting plasma glucose	162.7 (39.4 to 345.3)	5 High fasting plasma glucose	144.7 (34.2 to 315.1)	-11.0 (-19.4 to -1.7)
6 Occupational exposure to asbestos	125.5 (84.8 to 169.5)	6 Occupational exposure to asbestos	96.2 (64.5 to 131.7)	-23.3 (-31.2 to -14.6)
7 Diet low in whole grains	80.8 (31.9 to 104.4)	7 Diet low in whole grains	76.9 (30.1 to 102.4)	-4.8 (-13.5 to 4.1)
8 Diet low in fruits	70.4 (33.2 to 116.4)	8 Diet low in milk	66.8 (42.1 to 93.0)	4.5 (-7.9 to 18.2)
9 Secondhand smoke	66.9 (39.2 to 97.7)	9 Secondhand smoke	57.2 (33.5 to 86.7)	-14.4 (-26.1 to -0.9)
10 Diet low in milk	63.9 (40.4 to 88.6)	10 Diet low in fruits	55.0 (25.2 to 91.1)	-21.8 (-34.6 to -8.0)

Females

Leading risk 2010	ASR of DALYs 2010	Leading risk 2019	ASR of DALYs 2019	Percentage change in ASR of DALYs 2010-2019
1 Smoking	264.9 (241.9 to 290.7)	1 Smoking	250.0 (224.3 to 280.5)	-5.6 (-12.8 to 3.0)
2 Unsafe sex	171.0 (149.9 to 181.0)	2 Unsafe sex	154.7 (124.0 to 173.5)	-9.6 (-20.2 to 1.2)
3 High body-mass index	144.6 (87.0 to 212.0)	3 High body-mass index	140.1 (85.2 to 208.8)	-3.1 (-10.3 to 5.6)
4 High fasting plasma glucose	90.4 (24.1 to 186.3)	4 High fasting plasma glucose	86.7 (23.3 to 186.4)	-4.1 (-11.9 to 4.8)
5 Alcohol use	72.9 (64.2 to 81.7)	5 Ambient particulate matter pollution	63.6 (45.7 to 83.5)	1.8 (-15.5 to 22.0)
6 Ambient particulate matter pollution	62.5 (45.8 to 79.1)	6 Alcohol use	61.4 (53.0 to 70.5)	-15.8 (-22.5 to -8.0)
7 Secondhand smoke	49.6 (32.6 to 68.7)	7 Secondhand smoke	47.3 (30.8 to 68.5)	-4.6 (-17.9 to 10.8)
8 Diet low in whole grains	49.4 (19.4 to 64.0)	8 Diet low in whole grains	45.0 (18.0 to 58.8)	-8.9 (-16.2 to -0.5)
9 Diet low in milk	37.1 (23.0 to 52.3)	9 Diet low in milk	37.7 (23.2 to 51.7)	1.5 (-9.2 to 14.4)
10 Diet high in red meat	36.7 (21.1 to 54.0)	10 Diet high in red meat	34.7 (20.2 to 50.6)	-5.6 (-14.0 to 4.3)

Both sexes combined

Leading risk 2010	ASR of DALYs 2010	Leading risk 2019	ASR of DALYs 2019	Percentage change in ASR of DALYs 2010-2019
1 Smoking	978.4 (917.2 to 1 037.2)	1 Smoking	859.2 (773.1 to 952.3)	-12.2 (-21.2 to -2.7)
2 Alcohol use	200.4 (180.0 to 222.3)	2 Alcohol use	179.6 (157.4 to 203.6)	-10.4 (-19.0 to -0.7)
3 High body-mass index	163.3 (93.7 to 251.1)	3 High body-mass index	164.5 (94.4 to 251.9)	0.8 (-6.4 to 9.0)
4 Ambient particulate matter pollution	131.0 (98.0 to 163.4)	4 Ambient particulate matter pollution	119.1 (88.3 to 153.4)	-9.1 (-20.3 to 4.5)
5 High fasting plasma glucose	121.8 (32.5 to 248.5)	5 High fasting plasma glucose	112.3 (30.1 to 233.2)	-7.8 (-14.8 to -0.8)
6 Unsafe sex	88.6 (77.9 to 93.8)	6 Unsafe sex	79.5 (63.8 to 89.0)	-10.3 (-20.8 to 0.3)
7 Occupational exposure to asbestos	64.0 (45.7 to 83.9)	7 Diet low in whole grains	59.6 (23.5 to 78.2)	-6.1 (-12.4 to 0.2)
8 Diet low in whole grains	63.4 (24.9 to 81.9)	8 Secondhand smoke	51.3 (33.1 to 74.1)	-9.7 (-18.6 to -0.1)
9 Secondhand smoke	56.8 (37.3 to 81.2)	9 Diet low in milk	51.0 (31.9 to 70.2)	3.8 (-6.3 to 14.0)
10 Diet low in milk	49.1 (30.6 to 68.5)	10 Occupational exposure to asbestos	51.0 (36.4 to 67.1)	-20.3 (-28.3 to -12.1)

D Middle SDI

Males

Leading risk 2010	ASR of DALYs 2010	Leading risk 2019	ASR of DALYs 2019	Percentage change in ASR of DALYs 2010-2019
1 Smoking	1 415.8 (1 293.5 to 1 548.4)	1 Smoking	1 271.3 (1 088.1 to 1 481.6)	-10.2 (-23.9 to 5.6)
2 Alcohol use	268.1 (229.9 to 311.0)	2 Alcohol use	260.5 (217.5 to 308.7)	-2.8 (-18.1 to 14.1)
3 Ambient particulate matter pollution	168.3 (117.2 to 216.7)	3 Ambient particulate matter pollution	171.0 (119.9 to 228.1)	1.6 (-17.0 to 24.0)
4 High body-mass index	123.7 (51.8 to 230.9)	4 High body-mass index	142.8 (66.6 to 253.0)	15.4 (-3.0 to 41.0)
5 High fasting plasma glucose	99.4 (23.1 to 215.9)	5 High fasting plasma glucose	105.0 (24.6 to 233.1)	5.7 (-8.2 to 21.9)
6 Household air pollution from solid fuels	78.7 (42.8 to 123.9)	6 Diet low in milk	63.2 (42.2 to 84.2)	2.8 (-9.9 to 16.9)
7 Diet low in fruits	77.4 (37.4 to 132.9)	7 Diet low in calcium	62.2 (47.1 to 81.2)	-4.6 (-16.7 to 8.3)
8 Diet low in calcium	65.2 (51.7 to 81.4)	8 Diet low in fruits	57.7 (26.9 to 96.6)	-25.4 (-41.0 to -6.6)
9 Diet low in milk	61.5 (42.4 to 80.6)	9 Diet low in whole grains	51.3 (19.8 to 69.8)	2.2 (-10.8 to 16.6)
10 Diet high in sodium	52.6 (1.3 to 201.4)	10 Secondhand smoke	45.5 (25.7 to 70.0)	-2.7 (-21.0 to 18.1)
11 Diet low in whole grains	50.2 (19.3 to 67.0)	11 Household air pollution from solid fuels	41.9 (19.0 to 74.8)	-46.8 (-60.6 to -32.1)
12 Secondhand smoke	46.7 (26.6 to 70.1)	12 Diet high in sodium	38.5 (0.9 to 147.8)	-26.8 (-39.9 to -11.6)

Females

Leading risk 2010	ASR of DALYs 2010	Leading risk 2019	ASR of DALYs 2019	Percentage change in ASR of DALYs 2010-2019
1 Unsafe sex	228.5 (188.3 to 249.0)	1 Unsafe sex	204.6 (161.9 to 233.5)	-10.4 (-20.9 to 1.1)
2 Smoking	148.8 (132.1 to 169.1)	2 Smoking	128.2 (108.0 to 150.4)	-13.8 (-25.3 to -1.3)
3 High body-mass index	103.3 (54.9 to 168.7)	3 High body-mass index	109.8 (62.0 to 173.7)	6.2 (-6.2 to 24.2)
4 High fasting plasma glucose	72.7 (19.4 to 150.4)	4 High fasting plasma glucose	80.1 (21.1 to 170.1)	10.2 (-1.3 to 22.6)
5 Ambient particulate matter pollution	54.7 (37.2 to 71.8)	5 Ambient particulate matter pollution	63.0 (44.5 to 84.1)	15.3 (-5.3 to 40.6)
6 Secondhand smoke	48.4 (31.3 to 67.6)	6 Secondhand smoke	49.0 (30.9 to 71.7)	1.2 (-14.2 to 17.8)
7 Diet low in milk	39.7 (27.0 to 52.1)	7 Diet low in milk	39.3 (25.9 to 52.4)	-1.0 (-12.9 to 11.1)
8 Diet low in calcium	39.2 (30.0 to 50.3)	8 Diet low in calcium	35.9 (26.6 to 47.2)	-8.6 (-19.1 to 2.4)
9 Household air pollution from solid fuels	38.0 (22.7 to 56.1)	9 Alcohol use	35.9 (30.0 to 42.3)	-1.1 (-13.3 to 12.5)
10 Alcohol use	36.3 (31.2 to 42.1)	10 Diet low in whole grains	31.1 (12.1 to 42.4)	-1.7 (-13.4 to 10.5)
11 Diet low in whole grains	31.7 (12.1 to 42.5)	11 Household air pollution from solid fuels	22.7 (11.4 to 37.7)	-40.3 (-55.6 to -24.4)

Both sexes combined

Leading risk 2010	ASR of DALYs 2010	Leading risk 2019	ASR of DALYs 2019	Percentage change in ASR of DALYs 2010-2019
1 Smoking	761.4 (697.4 to 826.8)	1 Smoking	674.5 (583.0 to 775.7)	-11.4 (-23.8 to 3.6)
2 Alcohol use	149.9 (129.4 to 172.3)	2 Alcohol use	144.7 (122.4 to 168.5)	-3.5 (-17.0 to 11.9)
3 Unsafe sex	115.7 (95.4 to 126.2)	3 High body-mass index	126.0 (66.8 to 202.8)	11.1 (-1.7 to 29.7)
4 High body-mass index	113.5 (56.8 to 191.8)	4 Ambient particulate matter pollution	114.6 (82.4 to 148.6)	4.6 (-11.2 to 24.8)
5 Ambient particulate matter pollution	109.6 (76.4 to 140.9)	5 Unsafe sex	104.5 (82.7 to 119.2)	-9.7 (-20.3 to 1.9)
6 High fasting plasma glucose	85.2 (22.5 to 177.0)	6 High fasting plasma glucose	91.7 (24.6 to 191.4)	7.6 (-2.4 to 19.1)
7 Household air pollution from solid fuels	57.7 (33.2 to 87.5)	7 Diet low in milk	50.8 (34.0 to 66.9)	1.0 (-8.5 to 11.5)
8 Diet low in fruits	52.3 (25.6 to 88.9)	8 Diet low in calcium	48.5 (36.6 to 63.7)	-6.4 (-15.8 to 3.1)
9 Diet low in calcium	51.8 (40.7 to 65.6)	9 Secondhand smoke	47.0 (30.0 to 68.2)	-0.6 (-13.4 to 13.4)
10 Diet low in milk	50.3 (34.5 to 65.5)	10 Diet low in whole grains	40.8 (15.6 to 54.9)	0.4 (-8.9 to 10.8)
11 Secondhand smoke	47.3 (30.7 to 67.1)	11 Diet low in fruits	38.5 (18.4 to 63.4)	-26.3 (-39.8 to -10.4)
12 Diet low in whole grains	40.7 (15.5 to 54.4)	12 Household air pollution from solid fuels	31.9 (15.6 to 53.8)	-44.8 (-57.9 to -31.4)

E Low-middle SDI

Males

Leading risk 2010	ASR of DALYs 2010	Leading risk 2019	ASR of DALYs 2019	Percentage change in ASR of DALYs 2010-2019
1 Smoking	810.0 (748.7 to 877.9)	1 Smoking	772.7 (689.5 to 870.0)	-4.6 (-14.5 to 5.7)
2 Alcohol use	177.4 (151.4 to 205.6)	2 Alcohol use	198.4 (163.8 to 239.5)	11.8 (-3.2 to 29.2)
3 Household air pollution from solid fuels	81.6 (51.4 to 113.9)	3 Ambient particulate matter pollution	70.3 (46.0 to 94.7)	29.9 (7.6 to 61.6)
4 Chewing tobacco	60.5 (43.1 to 79.3)	4 High fasting plasma glucose	69.5 (16.7 to 149.2)	23.0 (10.0 to 38.5)
5 High fasting plasma glucose	56.5 (13.7 to 122.1)	5 High body-mass index	69.2 (32.9 to 120.7)	35.2 (19.6 to 58.4)
6 Ambient particulate matter pollution	54.1 (31.7 to 76.5)	6 Chewing tobacco	60.1 (40.9 to 82.6)	-0.6 (-18.1 to 20.2)
7 Diet low in fruits	51.8 (30.0 to 76.4)	7 Household air pollution from solid fuels	55.2 (31.7 to 81.4)	-32.4 (-46.0 to -18.8)
8 High body-mass index	51.2 (22.3 to 95.2)	8 Diet low in fruits	48.8 (28.7 to 72.8)	-5.8 (-16.7 to 6.6)
9 Diet low in calcium	41.4 (33.5 to 51.7)	9 Diet low in calcium	42.9 (32.8 to 54.9)	3.4 (-8.7 to 15.0)
10 Diet low in milk	38.1 (26.0 to 50.0)	10 Diet low in milk	41.9 (28.3 to 56.0)	10.2 (-3.3 to 23.6)

Females

Leading risk 2010	ASR of DALYs 2010	Leading risk 2019	ASR of DALYs 2019	Percentage change in ASR of DALYs 2010-2019
1 Unsafe sex	296.5 (261.9 to 359.4)	1 Unsafe sex	285.6 (244.6 to 342.2)	-3.7 (-14.9 to 8.0)
2 Smoking	109.0 (92.9 to 130.0)	2 Smoking	101.5 (84.4 to 123.4)	-6.9 (-16.5 to 3.4)
3 High body-mass index	60.2 (30.5 to 99.2)	3 High fasting plasma glucose	76.5 (20.1 to 165.5)	32.7 (17.6 to 49.6)
4 High fasting plasma glucose	57.6 (14.7 to 122.8)	4 High body-mass index	75.7 (42.1 to 119.3)	25.8 (12.8 to 44.1)
5 Chewing tobacco	43.9 (35.3 to 53.8)	5 Chewing tobacco	47.9 (35.9 to 62.4)	9.2 (-9.6 to 31.8)
6 Household air pollution from solid fuels	32.1 (22.3 to 42.8)	6 Diet low in milk	37.1 (24.5 to 49.4)	15.5 (-0.1 to 31.4)
7 Diet low in milk	32.1 (22.0 to 41.8)	7 Diet low in calcium	33.6 (25.5 to 43.9)	5.4 (-6.9 to 18.6)
8 Diet low in calcium	31.9 (24.8 to 40.1)	8 Alcohol use	31.1 (25.4 to 36.9)	13.0 (-0.7 to 29.1)
9 Alcohol use	27.5 (23.5 to 32.2)	9 Secondhand smoke	29.7 (16.6 to 43.9)	9.4 (-4.0 to 24.2)
10 Secondhand smoke	27.1 (14.8 to 39.9)	10 Diet low in whole grains	28.0 (10.8 to 38.3)	13.1 (-1.1 to 27.2)
11 Diet low in whole grains	24.7 (9.7 to 33.0)	11 Household air pollution from solid fuels	27.0 (17.0 to 38.2)	-16.0 (-31.5 to -1.4)

Both sexes combined

Leading risk 2010	ASR of DALYs 2010	Leading risk 2019	ASR of DALYs 2019	Percentage change in ASR of DALYs 2010-2019
1 Smoking	451.0 (418.2 to 488.9)	1 Smoking	424.4 (380.9 to 474.7)	-5.9 (-14.5 to 3.5)
2 Unsafe sex	149.3 (132.0 to 181.0)	2 Unsafe sex	145.3 (124.5 to 174.1)	-2.6 (-14.0 to 9.1)
3 Alcohol use	101.5 (87.3 to 116.9)	3 Alcohol use	112.6 (94.6 to 134.3)	10.9 (-2.5 to 27.1)
4 High fasting plasma glucose	56.9 (15.4 to 117.8)	4 High fasting plasma glucose	72.9 (19.9 to 151.5)	28.1 (18.1 to 39.9)
5 Household air pollution from solid fuels	56.3 (37.4 to 76.6)	5 High body-mass index	72.8 (39.1 to 116.0)	30.1 (18.1 to 48.7)
6 High body-mass index	55.9 (27.5 to 94.9)	6 Chewing tobacco	54.0 (41.7 to 68.3)	3.5 (-10.6 to 19.5)
7 Chewing tobacco	52.2 (41.9 to 63.0)	7 Ambient particulate matter pollution	45.7 (29.7 to 62.0)	35.0 (13.3 to 65.0)
8 Diet low in fruits	37.9 (22.2 to 55.7)	8 Household air pollution from solid fuels	40.6 (25.0 to 57.7)	-27.9 (-40.5 to -16.2)
9 Diet low in calcium	36.6 (29.2 to 45.8)	9 Diet low in milk	39.4 (26.5 to 52.0)	12.6 (1.8 to 23.2)
10 Diet low in milk	35.0 (24.0 to 45.7)	10 Diet low in calcium	38.1 (29.5 to 49.2)	4.1 (-5.8 to 13.6)
11 Ambient particulate matter pollution	33.8 (20.2 to 47.9)	11 Diet low in fruits	36.3 (21.6 to 53.4)	-4.1 (-12.9 to 5.6)

F Low SDI

Males

Leading risk 2010	ASR of DALYs 2010	Leading risk 2019	ASR of DALYs 2019	Percentage change in ASR of DALYs 2010-2019
1 Smoking	495.3 (428.3 to 566.4)	1 Smoking	463.9 (390.9 to 534.9)	-6.3 (-17.4 to 6.2)
2 Alcohol use	142.4 (119.8 to 166.7)	2 Alcohol use	152.3 (125.3 to 182.9)	6.9 (-5.9 to 21.0)
3 Household air pollution from solid fuels	92.9 (58.1 to 144.5)	3 Household air pollution from solid fuels	76.4 (48.4 to 111.9)	-17.8 (-30.0 to -4.6)
4 Chewing tobacco	47.6 (33.7 to 62.8)	4 High body-mass index	57.3 (24.2 to 104.9)	26.5 (9.6 to 59.9)
5 Diet low in fruits	46.4 (26.2 to 70.8)	5 High fasting plasma glucose	51.6 (12.9 to 113.9)	13.2 (0.9 to 27.8)
6 High fasting plasma glucose	45.6 (11.2 to 101.6)	6 Chewing tobacco	44.9 (30.4 to 62.5)	-5.7 (-23.1 to 13.1)
7 High body-mass index	45.3 (17.1 to 87.2)	7 Diet low in fruits	44.5 (25.3 to 68.1)	-4.1 (-15.4 to 7.5)
8 Diet low in calcium	38.3 (30.4 to 48.6)	8 Diet low in calcium	40.0 (31.5 to 51.1)	4.3 (-9.5 to 17.9)
9 Diet low in milk	30.7 (20.1 to 41.8)	9 Ambient particulate matter pollution	37.8 (21.6 to 57.9)	35.0 (7.7 to 78.4)
10 Ambient particulate matter pollution	28.0 (13.4 to 48.5)	10 Diet low in milk	33.0 (21.5 to 45.1)	7.7 (-7.1 to 21.6)

Females

Leading risk 2010	ASR of DALYs 2010	Leading risk 2019	ASR of DALYs 2019	Percentage change in ASR of DALYs 2010-2019
1 Unsafe sex	510.5 (406.8 to 607.9)	1 Unsafe sex	477.5 (374.3 to 591.4)	-6.5 (-16.5 to 5.6)
2 Smoking	96.1 (74.8 to 120.9)	2 Smoking	90.0 (70.8 to 115.7)	-6.4 (-15.9 to 3.9)
3 High body-mass index	55.3 (25.1 to 98.0)	3 High body-mass index	70.8 (35.7 to 117.5)	28.0 (13.2 to 51.2)
4 High fasting plasma glucose	47.3 (11.5 to 101.9)	4 High fasting plasma glucose	60.4 (14.9 to 131.6)	27.6 (14.0 to 44.8)
5 Alcohol use	40.5 (33.4 to 48.2)	5 Alcohol use	46.0 (36.7 to 55.8)	13.6 (-0.4 to 27.0)
6 Chewing tobacco	34.4 (25.5 to 44.4)	6 Chewing tobacco	36.5 (26.4 to 47.6)	6.1 (-13.7 to 30.6)
7 Diet low in calcium	31.0 (24.8 to 38.7)	7 Diet low in calcium	33.5 (26.4 to 41.7)	8.0 (-3.5 to 20.7)
8 Household air pollution from solid fuels	26.9 (17.6 to 42.2)	8 Diet low in milk	29.5 (19.4 to 39.5)	12.1 (0.1 to 25.6)
9 Diet low in milk	26.3 (17.6 to 35.1)	9 Household air pollution from solid fuels	27.4 (18.7 to 38.8)	1.8 (-14.2 to 18.4)
10 Diet low in fruits	25.1 (12.2 to 39.3)	10 Diet low in whole grains	24.6 (9.7 to 33.2)	10.6 (-1.1 to 24.2)
11 Diet low in whole grains	22.3 (9.0 to 29.4)	11 Diet low in fruits	24.5 (12.2 to 38.4)	-2.4 (-13.0 to 10.4)

Both sexes combined

Leading risk 2010	ASR of DALYs 2010	Leading risk 2019	ASR of DALYs 2019	Percentage change in ASR of DALYs 2010-2019
1 Smoking	293.7 (257.1 to 333.5)	1 Smoking	273.9 (235.2 to 311.1)	-6.7 (-16.4 to 4.0)
2 Unsafe sex	255.3 (203.5 to 304.0)	2 Unsafe sex	240.5 (188.6 to 297.9)	-5.8 (-15.8 to 6.3)
3 Alcohol use	91.3 (77.0 to 106.8)	3 Alcohol use	98.6 (81.1 to 116.6)	8.0 (-3.6 to 21.3)
4 Household air pollution from solid fuels	59.6 (38.6 to 88.7)	4 High body-mass index	64.2 (32.8 to 105.7)	27.3 (13.5 to 51.1)
5 High body-mass index	50.4 (23.2 to 89.7)	5 High fasting plasma glucose	55.9 (15.1 to 118.4)	20.7 (11.5 to 32.4)
6 High fasting plasma glucose	46.3 (12.4 to 97.3)	6 Household air pollution from solid fuels	51.5 (33.9 to 73.3)	-13.5 (-25.8 to -1.2)
7 Chewing tobacco	41.0 (32.1 to 50.0)	7 Chewing tobacco	40.7 (31.6 to 51.1)	-0.8 (-14.1 to 14.4)
8 Diet low in fruits	35.7 (19.5 to 54.3)	8 Diet low in calcium	36.7 (29.3 to 45.7)	5.9 (-4.4 to 15.8)
9 Diet low in calcium	34.6 (27.7 to 43.0)	9 Diet low in fruits	34.4 (18.9 to 52.8)	-3.6 (-12.5 to 5.9)
10 Diet low in milk	28.4 (18.8 to 38.3)	10 Diet low in milk	31.2 (20.6 to 41.9)	9.8 (-1.5 to 20.7)

Appendix Figure 18: Leading risk factors at the most detailed level for attributable cancer age-standardised DALY rates, 2010-2019 for (A) the global level, (B) high SDI quintile, (C) high-middle SDI quintile, (D) middle SDI quintile, (E) low-middle SDI quintile, and (F) low SDI quintile, for males, females, and both sexes combined. Data in parentheses are 95% uncertainty intervals (UIs). Rows are color-coded by the following: red color = environmental and occupational risk factors; blue color = behavioural risk factors; green color = metabolic risk factors. Dashed lines indicate decrease in rank; solid lines indicate increase or no change in rank. Risk factors at the most detailed level reflect the GBD hierarchy in which these categories of risks fall, ranging from Levels 2-4 (for more information on risk factor levels in the GBD hierarchy see Appendix table 9, p152). ASR = age-standardised rate; SDI = Socio-demographic Index; DALYs = disability-adjusted life-years.

A Global

Males

Leading risk 2010	ASR of Deaths 2010	Leading risk 2019	ASR of Deaths 2019	Percentage change in ASR of Deaths 2010-2019
1 Smoking	61.6 (57.7 to 65.3)	1 Smoking	54.6 (49.5 to 60.1)	-11.4 (-19.2 to -3.0)
2 Alcohol use	10.7 (9.5 to 11.9)	2 Alcohol use	10.3 (9.0 to 11.6)	-3.7 (-12.1 to 5.1)
3 Occupational exposure to asbestos	7.0 (4.9 to 9.0)	3 High fasting plasma glucose	6.3 (1.6 to 13.4)	-0.9 (-7.5 to 6.4)
4 High fasting plasma glucose	6.4 (1.6 to 13.5)	4 High body-mass index	6.3 (3.2 to 10.4)	6.4 (-2.0 to 16.1)
5 Ambient particulate matter pollution	6.0 (4.4 to 7.7)	5 Occupational exposure to asbestos	5.8 (4.1 to 7.5)	-17.0 (-21.5 to -12.3)
6 High body-mass index	5.9 (2.9 to 10.0)	6 Ambient particulate matter pollution	5.8 (4.2 to 7.5)	-4.2 (-16.5 to 10.6)
7 Diet low in fruits	2.9 (1.4 to 4.6)	7 Diet low in whole grains	2.7 (1.0 to 3.5)	-3.0 (-9.4 to 3.6)
8 Diet low in whole grains	2.7 (1.1 to 3.6)	8 Diet low in milk	2.6 (1.6 to 3.5)	1.8 (-6.3 to 10.6)
9 Diet low in milk	2.5 (1.6 to 3.4)	9 Diet low in fruits	2.4 (1.2 to 3.8)	-17.5 (-27.3 to -6.8)
10 Diet low in calcium	2.3 (1.7 to 3.1)	10 Diet low in calcium	2.2 (1.6 to 3.0)	-4.7 (-12.9 to 3.3)

Females

Leading risk 2010	ASR of Deaths 2010	Leading risk 2019	ASR of Deaths 2019	Percentage change in ASR of Deaths 2010-2019
1 Smoking	11.8 (10.8 to 12.8)	1 Smoking	10.5 (9.4 to 11.5)	-10.9 (-15.5 to -5.9)
2 Unsafe sex	6.8 (6.1 to 7.7)	2 Unsafe sex	6.5 (5.5 to 7.3)	-4.9 (-12.6 to 3.6)
3 High body-mass index	5.1 (3.0 to 7.8)	3 High body-mass index	5.2 (3.1 to 7.7)	1.7 (-3.9 to 8.9)
4 High fasting plasma glucose	4.2 (1.2 to 8.7)	4 High fasting plasma glucose	4.4 (1.2 to 9.3)	5.1 (-0.8 to 11.5)
5 Alcohol use	2.6 (2.2 to 2.9)	5 Alcohol use	2.3 (2.0 to 2.6)	-10.3 (-14.8 to -5.2)
6 Ambient particulate matter pollution	1.9 (1.4 to 2.5)	6 Ambient particulate matter pollution	2.1 (1.5 to 2.7)	7.3 (-6.0 to 22.7)
7 Diet low in whole grains	1.8 (0.7 to 2.4)	7 Diet low in whole grains	1.7 (0.7 to 2.3)	-5.2 (-10.7 to 0.4)
8 Diet low in milk	1.7 (1.1 to 2.3)	8 Diet low in milk	1.7 (1.1 to 2.3)	1.3 (-6.1 to 9.3)
9 Secondhand smoke	1.5 (0.9 to 2.0)	9 Secondhand smoke	1.5 (0.9 to 2.1)	1.2 (-9.8 to 13.4)
10 Diet low in calcium	1.4 (1.0 to 1.9)	10 Diet low in calcium	1.3 (0.9 to 1.8)	-4.6 (-12.0 to 2.8)

Both sexes combined

Leading risk 2010	ASR of Deaths 2010	Leading risk 2019	ASR of Deaths 2019	Percentage change in ASR of Deaths 2010-2019
1 Smoking	34.4 (32.2 to 36.3)	1 Smoking	30.6 (28.0 to 33.3)	-10.9 (-17.6 to -3.5)
2 Alcohol use	6.3 (5.7 to 7.1)	2 Alcohol use	6.0 (5.4 to 6.8)	-5.0 (-11.9 to 2.4)
3 High body-mass index	5.5 (3.0 to 8.6)	3 High body-mass index	5.7 (3.2 to 8.8)	4.0 (-1.7 to 11.0)
4 High fasting plasma glucose	5.2 (1.4 to 10.4)	4 High fasting plasma glucose	5.3 (1.5 to 10.6)	2.0 (-3.2 to 8.0)
5 Ambient particulate matter pollution	3.8 (2.8 to 4.8)	5 Ambient particulate matter pollution	3.8 (2.8 to 4.9)	-0.6 (-10.4 to 11.4)
6 Unsafe sex	3.6 (3.2 to 4.1)	6 Unsafe sex	3.4 (2.9 to 3.8)	-5.2 (-12.7 to 3.2)
7 Occupational exposure to asbestos	3.5 (2.6 to 4.5)	7 Occupational exposure to asbestos	3.0 (2.2 to 3.8)	-14.6 (-19.2 to -10.1)
8 Diet low in whole grains	2.3 (0.9 to 2.9)	8 Diet low in whole grains	2.2 (0.8 to 2.8)	-3.9 (-8.8 to 0.8)
9 Diet low in milk	2.1 (1.3 to 2.8)	9 Diet low in milk	2.1 (1.3 to 2.8)	1.6 (-4.3 to 8.1)
10 Diet low in fruits	1.9 (0.9 to 3.0)	10 Diet low in calcium	1.7 (1.2 to 2.4)	-4.5 (-10.7 to 1.7)
11 Diet low in calcium	1.8 (1.3 to 2.5)	12 Diet low in fruits	1.6 (0.8 to 2.5)	-16.2 (-24.9 to -7.3)

B High SDI

Males

Leading risk 2010	ASR of Deaths 2010	Leading risk 2019	ASR of Deaths 2019	Percentage change in ASR of Deaths 2010-2019
1 Smoking	63.7 (59.9 to 66.9)	1 Smoking	53.4 (49.7 to 56.6)	-16.2 (-18.0 to -14.3)
2 Occupational exposure to asbestos	15.3 (11.2 to 19.3)	2 Occupational exposure to asbestos	13.0 (9.5 to 16.5)	-15.1 (-19.7 to -10.3)
3 Alcohol use	12.0 (10.9 to 13.2)	3 Alcohol use	11.3 (10.2 to 12.4)	-6.1 (-9.6 to -2.2)
4 High body-mass index	8.8 (4.7 to 13.9)	4 High body-mass index	8.9 (4.8 to 13.9)	0.6 (-2.8 to 5.8)
5 High fasting plasma glucose	8.6 (2.2 to 18.0)	5 High fasting plasma glucose	8.7 (2.2 to 18.0)	1.4 (-2.2 to 6.9)
6 Ambient particulate matter pollution	4.2 (2.9 to 5.8)	6 Diet low in whole grains	3.2 (1.2 to 4.1)	-6.7 (-9.1 to -4.0)
7 Diet low in whole grains	3.4 (1.3 to 4.5)	7 Ambient particulate matter pollution	3.2 (2.1 to 4.5)	-23.6 (-29.8 to -17.8)
8 Diet low in fruits	2.6 (1.2 to 4.0)	8 Diet low in milk	2.4 (1.3 to 3.6)	-6.4 (-9.6 to -2.5)
9 Diet low in milk	2.6 (1.4 to 3.8)	9 Diet low in fruits	2.2 (1.0 to 3.5)	-15.1 (-19.4 to -11.1)
10 Residential radon	1.9 (0.4 to 3.7)	10 Drug use	1.7 (1.3 to 2.1)	3.2 (-6.6 to 11.1)
13 Drug use	1.6 (1.3 to 2.0)	11 Residential radon	1.7 (0.3 to 3.2)	-13.7 (-16.6 to -11.2)

Females

Leading risk 2010	ASR of Deaths 2010	Leading risk 2019	ASR of Deaths 2019	Percentage change in ASR of Deaths 2010-2019
1 Smoking	22.7 (21.0 to 24.2)	1 Smoking	20.7 (19.0 to 22.2)	-8.9 (-11.1 to -6.8)
2 High body-mass index	6.3 (3.9 to 9.1)	2 High body-mass index	6.3 (3.9 to 9.1)	0.2 (-2.7 to 4.0)
3 High fasting plasma glucose	5.7 (1.5 to 11.7)	3 High fasting plasma glucose	6.1 (1.7 to 12.4)	6.6 (2.9 to 11.6)
4 Alcohol use	4.8 (4.2 to 5.4)	4 Alcohol use	4.4 (3.8 to 5.0)	-8.1 (-12.1 to -4.1)
5 Unsafe sex	3.1 (2.8 to 3.2)	5 Unsafe sex	2.9 (2.6 to 3.1)	-6.5 (-10.0 to -2.5)
6 Diet low in whole grains	2.2 (0.8 to 2.8)	6 Diet low in whole grains	2.0 (0.8 to 2.7)	-5.9 (-8.6 to -3.3)
7 Occupational exposure to asbestos	1.9 (1.2 to 2.4)	7 Occupational exposure to asbestos	1.8 (1.1 to 2.4)	-4.1 (-13.1 to 4.7)
8 Ambient particulate matter pollution	1.8 (1.2 to 2.5)	8 Diet low in milk	1.6 (0.9 to 2.3)	-4.9 (-8.2 to -0.8)
9 Diet low in milk	1.6 (0.9 to 2.4)	9 Diet high in red meat	1.5 (0.9 to 2.3)	-5.1 (-9.1 to -0.7)
10 Diet high in red meat	1.6 (0.9 to 2.4)	10 Ambient particulate matter pollution	1.4 (0.9 to 2.1)	-18.7 (-25.8 to -13.2)

Both sexes combined

Leading risk 2010	ASR of Deaths 2010	Leading risk 2019	ASR of Deaths 2019	Percentage change in ASR of Deaths 2010-2019
1 Smoking	40.6 (38.1 to 42.7)	1 Smoking	35.4 (32.9 to 37.4)	-13.0 (-14.7 to -11.3)
2 Alcohol use	8.1 (7.3 to 8.9)	2 Alcohol use	7.6 (6.8 to 8.4)	-6.0 (-9.4 to -2.5)
3 Occupational exposure to asbestos	7.5 (5.7 to 9.3)	3 High body-mass index	7.5 (4.4 to 11.1)	0.6 (-2.1 to 4.4)
4 High body-mass index	7.5 (4.4 to 11.3)	4 High fasting plasma glucose	7.2 (2.0 to 14.4)	4.4 (1.0 to 8.9)
5 High fasting plasma glucose	6.9 (1.9 to 13.8)	5 Occupational exposure to asbestos	6.7 (5.1 to 8.3)	-11.5 (-16.6 to -6.4)
6 Ambient particulate matter pollution	2.8 (1.9 to 3.9)	6 Diet low in whole grains	2.6 (1.0 to 3.3)	-5.9 (-8.0 to -3.6)
7 Diet low in whole grains	2.7 (1.0 to 3.6)	7 Ambient particulate matter pollution	2.2 (1.5 to 3.2)	-21.3 (-27.6 to -15.8)
8 Diet low in milk	2.1 (1.1 to 3.0)	8 Diet low in milk	1.9 (1.1 to 2.9)	-5.3 (-8.1 to -1.7)
9 Diet low in fruits	1.7 (0.8 to 2.6)	9 Diet low in fruits	1.5 (0.7 to 2.3)	-11.6 (-15.1 to -8.4)
10 Unsafe sex	1.7 (1.5 to 1.7)	10 Unsafe sex	1.5 (1.4 to 1.6)	-8.1 (-11.6 to -4.2)

C High-middle SDI

Males

Leading risk 2010	ASR of Deaths 2010	Leading risk 2019	ASR of Deaths 2019	Percentage change in ASR of Deaths 2010-2019
1 Smoking	81.4 (75.7 to 86.5)	1 Smoking	71.3 (63.5 to 79.7)	-12.4 (-22.1 to -1.6)
2 Alcohol use	13.5 (12.0 to 15.0)	2 Alcohol use	12.3 (10.5 to 14.1)	-8.9 (-18.7 to 1.6)
3 Ambient particulate matter pollution	9.5 (7.1 to 12.0)	3 Ambient particulate matter pollution	8.5 (6.1 to 11.0)	-11.3 (-25.0 to 4.7)
4 High fasting plasma glucose	8.4 (2.1 to 17.7)	4 High body-mass index	7.9 (4.0 to 12.9)	3.6 (-6.0 to 14.4)
5 High body-mass index	7.6 (3.8 to 12.6)	5 High fasting plasma glucose	7.5 (1.8 to 16.0)	-10.9 (-18.8 to -2.5)
6 Occupational exposure to asbestos	7.0 (4.7 to 9.4)	6 Occupational exposure to asbestos	5.5 (3.7 to 7.5)	-21.3 (-28.7 to -13.3)
7 Diet low in whole grains	3.8 (1.5 to 4.9)	7 Diet low in whole grains	3.6 (1.4 to 4.7)	-5.0 (-12.9 to 2.8)
8 Diet low in fruits	3.0 (1.4 to 5.0)	8 Diet low in milk	3.0 (1.9 to 4.2)	3.6 (-7.8 to 15.8)
9 Secondhand smoke	3.0 (1.7 to 4.3)	9 Secondhand smoke	2.6 (1.5 to 3.9)	-12.1 (-24.3 to 1.8)
10 Diet low in milk	2.9 (1.8 to 4.1)	10 Diet low in fruits	2.4 (1.1 to 4.0)	-20.0 (-33.2 to -5.7)

Females

Leading risk 2010	ASR of Deaths 2010	Leading risk 2019	ASR of Deaths 2019	Percentage change in ASR of Deaths 2010-2019
1 Smoking	11.4 (10.4 to 12.4)	1 Smoking	10.9 (9.7 to 12.3)	-3.9 (-12.2 to 5.4)
2 High body-mass index	6.2 (3.8 to 9.2)	2 High body-mass index	6.1 (3.7 to 9.0)	-2.7 (-9.6 to 5.6)
3 Unsafe sex	5.4 (4.8 to 5.7)	3 Unsafe sex	4.9 (3.9 to 5.5)	-9.3 (-19.5 to 1.4)
4 High fasting plasma glucose	4.4 (1.2 to 9.1)	4 High fasting plasma glucose	4.2 (1.1 to 9.0)	-4.7 (-12.1 to 4.0)
5 Alcohol use	2.7 (2.4 to 3.1)	5 Ambient particulate matter pollution	2.8 (2.0 to 3.7)	4.9 (-12.5 to 25.7)
6 Ambient particulate matter pollution	2.7 (1.9 to 3.4)	6 Alcohol use	2.3 (2.0 to 2.6)	-16.0 (-22.4 to -8.1)
7 Diet low in whole grains	2.3 (0.9 to 3.0)	7 Diet low in whole grains	2.1 (0.9 to 2.8)	-8.4 (-14.9 to -0.8)
8 Secondhand smoke	1.8 (1.2 to 2.6)	8 Secondhand smoke	1.8 (1.2 to 2.6)	-2.0 (-16.1 to 13.7)
9 Diet low in milk	1.7 (1.1 to 2.4)	9 Diet low in milk	1.7 (1.0 to 2.4)	1.3 (-8.4 to 12.9)
10 Diet high in red meat	1.4 (0.8 to 2.2)	10 Diet high in red meat	1.3 (0.7 to 2.0)	-5.6 (-13.3 to 3.9)

Both sexes combined

Leading risk 2010	ASR of Deaths 2010	Leading risk 2019	ASR of Deaths 2019	Percentage change in ASR of Deaths 2010-2019
1 Smoking	41.7 (38.9 to 44.2)	1 Smoking	37.5 (33.7 to 41.5)	-10.1 (-19.0 to -0.7)
2 Alcohol use	7.5 (6.7 to 8.3)	2 High body-mass index	6.9 (3.9 to 10.6)	0.6 (-5.8 to 7.8)
3 High body-mass index	6.9 (3.9 to 10.5)	3 Alcohol use	6.8 (5.9 to 7.7)	-9.6 (-18.2 to -0.3)
4 High fasting plasma glucose	6.1 (1.7 to 12.2)	4 High fasting plasma glucose	5.6 (1.5 to 11.4)	-7.8 (-14.1 to -1.4)
5 Ambient particulate matter pollution	5.6 (4.2 to 7.0)	5 Ambient particulate matter pollution	5.3 (3.9 to 6.8)	-6.2 (-17.3 to 7.2)
6 Occupational exposure to asbestos	3.4 (2.4 to 4.4)	6 Occupational exposure to asbestos	2.8 (2.0 to 3.6)	-18.0 (-25.1 to -10.3)
7 Diet low in whole grains	2.9 (1.2 to 3.8)	7 Diet low in whole grains	2.7 (1.1 to 3.6)	-6.1 (-11.7 to -0.5)
8 Unsafe sex	2.9 (2.6 to 3.1)	8 Unsafe sex	2.6 (2.1 to 2.9)	-10.4 (-20.3 to 0.1)
9 Secondhand smoke	2.3 (1.5 to 3.3)	9 Diet low in milk	2.3 (1.4 to 3.2)	3.2 (-4.9 to 12.5)
10 Diet low in milk	2.2 (1.4 to 3.1)	10 Secondhand smoke	2.1 (1.4 to 3.1)	-7.3 (-16.8 to 2.8)

D Middle SDI

Males

Leading risk 2010	ASR of Deaths 2010	Leading risk 2019	ASR of Deaths 2019	Percentage change in ASR of Deaths 2010-2019
1 Smoking	64.2 (58.8 to 69.9)	1 Smoking	58.5 (50.4 to 67.8)	-8.9 (-22.3 to 6.5)
2 Alcohol use	10.3 (8.7 to 12.0)	2 Alcohol use	10.1 (8.4 to 12.0)	-1.7 (-16.8 to 16.1)
3 Ambient particulate matter pollution	7.6 (5.3 to 9.8)	3 Ambient particulate matter pollution	7.9 (5.5 to 10.4)	3.2 (-15.0 to 25.3)
4 High fasting plasma glucose	5.1 (1.2 to 11.0)	4 High body-mass index	5.6 (2.5 to 10.0)	15.5 (-2.4 to 40.7)
5 High body-mass index	4.8 (1.9 to 9.1)	5 High fasting plasma glucose	5.3 (1.3 to 11.7)	4.5 (-8.0 to 19.4)
6 Household air pollution from solid fuels	3.6 (1.9 to 5.6)	6 Diet low in milk	2.7 (1.9 to 3.7)	3.2 (-9.2 to 16.5)
7 Diet low in fruits	3.5 (1.7 to 6.0)	7 Diet low in calcium	2.7 (2.1 to 3.6)	-4.3 (-16.1 to 8.2)
8 Diet low in calcium	2.9 (2.3 to 3.6)	8 Diet low in fruits	2.6 (1.2 to 4.5)	-24.0 (-40.4 to -5.4)
9 Diet low in milk	2.7 (1.8 to 3.5)	9 Diet low in whole grains	2.2 (0.9 to 3.0)	2.6 (-9.5 to 16.4)
10 Occupational exposure to asbestos	2.5 (1.6 to 3.5)	10 Secondhand smoke	2.1 (1.2 to 3.3)	-1.4 (-18.9 to 19.7)
12 Diet low in whole grains	2.2 (0.8 to 2.9)	11 Occupational exposure to asbestos	2.1 (1.4 to 3.1)	-14.2 (-27.2 to 1.5)
13 Secondhand smoke	2.2 (1.2 to 3.3)	12 Household air pollution from solid fuels	1.9 (0.9 to 3.4)	-46.4 (-60.0 to -31.9)

Females

Leading risk 2010	ASR of Deaths 2010	Leading risk 2019	ASR of Deaths 2019	Percentage change in ASR of Deaths 2010-2019
1 Unsafe sex	7.6 (6.3 to 8.3)	1 Unsafe sex	6.8 (5.4 to 7.8)	-10.3 (-20.7 to 1.3)
2 Smoking	7.5 (6.6 to 8.4)	2 Smoking	6.4 (5.4 to 7.5)	-13.6 (-25.3 to -1.4)
3 High body-mass index	4.2 (2.2 to 7.1)	3 High body-mass index	4.5 (2.5 to 7.2)	6.4 (-5.7 to 23.7)
4 High fasting plasma glucose	3.5 (0.9 to 7.3)	4 High fasting plasma glucose	3.8 (1.0 to 8.0)	8.0 (-2.9 to 19.5)
5 Ambient particulate matter pollution	2.5 (1.7 to 3.3)	5 Ambient particulate matter pollution	2.9 (2.1 to 3.9)	16.9 (-3.6 to 41.9)
6 Secondhand smoke	1.9 (1.2 to 2.7)	6 Secondhand smoke	1.9 (1.2 to 2.8)	2.6 (-13.3 to 19.3)
7 Diet low in calcium	1.8 (1.4 to 2.3)	7 Diet low in milk	1.8 (1.2 to 2.4)	-0.6 (-12.1 to 10.6)
8 Diet low in milk	1.8 (1.2 to 2.4)	8 Diet low in calcium	1.7 (1.2 to 2.2)	-8.2 (-18.3 to 2.3)
9 Household air pollution from solid fuels	1.7 (1.0 to 2.5)	9 Diet low in whole grains	1.4 (0.5 to 1.9)	-1.4 (-12.8 to 10.0)
10 Alcohol use	1.5 (1.2 to 1.7)	10 Alcohol use	1.4 (1.2 to 1.7)	-3.7 (-17.0 to 10.2)
11 Diet low in whole grains	1.4 (0.5 to 1.9)	11 Household air pollution from solid fuels	1.0 (0.5 to 1.7)	-39.6 (-55.0 to -23.8)

Both sexes combined

Leading risk 2010	ASR of Deaths 2010	Leading risk 2019	ASR of Deaths 2019	Percentage change in ASR of Deaths 2010-2019
1 Smoking	34.1 (31.3 to 37.0)	1 Smoking	30.7 (26.6 to 35.1)	-10.2 (-22.1 to 4.1)
2 Alcohol use	5.7 (4.8 to 6.6)	2 Alcohol use	5.5 (4.7 to 6.5)	-2.9 (-16.7 to 13.1)
3 Ambient particulate matter pollution	4.9 (3.4 to 6.3)	3 Ambient particulate matter pollution	5.2 (3.8 to 6.8)	6.5 (-9.4 to 26.2)
4 High body-mass index	4.5 (2.2 to 7.8)	4 High body-mass index	5.0 (2.6 to 8.2)	10.9 (-1.5 to 29.0)
5 High fasting plasma glucose	4.2 (1.1 to 8.8)	5 High fasting plasma glucose	4.5 (1.2 to 9.3)	5.9 (-3.5 to 16.7)
6 Unsafe sex	3.9 (3.3 to 4.3)	6 Unsafe sex	3.5 (2.8 to 4.1)	-9.6 (-20.0 to 2.0)
7 Household air pollution from solid fuels	2.6 (1.5 to 4.0)	7 Diet low in milk	2.2 (1.5 to 3.0)	1.4 (-7.7 to 10.8)
8 Diet low in fruits	2.4 (1.1 to 4.1)	8 Diet low in calcium	2.2 (1.6 to 2.8)	-6.2 (-15.3 to 2.6)
9 Diet low in calcium	2.3 (1.8 to 2.9)	9 Secondhand smoke	2.0 (1.3 to 3.0)	0.5 (-12.5 to 14.5)
10 Diet low in milk	2.2 (1.5 to 2.9)	10 Diet low in whole grains	1.8 (0.7 to 2.4)	0.7 (-8.3 to 10.1)
11 Secondhand smoke	2.0 (1.3 to 2.9)	11 Diet low in fruits	1.8 (0.8 to 2.9)	-25.5 (-39.3 to -9.6)
12 Diet low in whole grains	1.8 (0.7 to 2.4)	12 Household air pollution from solid fuels	1.5 (0.7 to 2.5)	-44.1 (-57.4 to -30.6)

E Low-middle SDI

Males

Leading risk 2010	ASR of Deaths 2010	Leading risk 2019	ASR of Deaths 2019	Percentage change in ASR of Deaths 2010-2019
1 Smoking	34.9 (32.3 to 37.8)	1 Smoking	33.8 (30.4 to 38.0)	-3.0 (-12.9 to 7.3)
2 Alcohol use	6.4 (5.5 to 7.4)	2 Alcohol use	7.2 (5.9 to 8.7)	13.2 (-2.3 to 31.1)
3 Household air pollution from solid fuels	3.5 (2.2 to 4.8)	3 High fasting plasma glucose	3.4 (0.8 to 7.2)	22.1 (10.1 to 36.7)
4 High fasting plasma glucose	2.8 (0.7 to 6.0)	4 Ambient particulate matter pollution	3.0 (2.0 to 4.1)	31.2 (9.6 to 62.7)
5 Ambient particulate matter pollution	2.3 (1.4 to 3.3)	5 High body-mass index	2.7 (1.2 to 4.7)	35.3 (20.2 to 58.9)
6 Chewing tobacco	2.2 (1.6 to 2.9)	6 Household air pollution from solid fuels	2.4 (1.4 to 3.5)	-31.7 (-45.2 to -18.2)
7 Diet low in fruits	2.1 (1.2 to 3.1)	7 Chewing tobacco	2.3 (1.5 to 3.1)	1.1 (-16.5 to 22.3)
8 High body-mass index	2.0 (0.8 to 3.7)	8 Diet low in fruits	2.0 (1.2 to 3.0)	-4.6 (-15.3 to 7.3)
9 Diet low in calcium	1.8 (1.5 to 2.3)	9 Diet low in calcium	1.9 (1.5 to 2.5)	6.1 (-6.0 to 17.3)
10 Diet low in milk	1.6 (1.1 to 2.1)	10 Diet low in milk	1.8 (1.2 to 2.4)	12.3 (-0.9 to 24.7)

Females

Leading risk 2010	ASR of Deaths 2010	Leading risk 2019	ASR of Deaths 2019	Percentage change in ASR of Deaths 2010-2019
1 Unsafe sex	9.2 (8.1 to 11.4)	1 Unsafe sex	8.9 (7.6 to 10.8)	-3.8 (-14.4 to 7.4)
2 Smoking	5.0 (4.3 to 5.8)	2 Smoking	4.7 (3.9 to 5.5)	-6.0 (-15.7 to 4.0)
3 High fasting plasma glucose	2.6 (0.7 to 5.4)	3 High fasting plasma glucose	3.4 (0.9 to 7.2)	30.9 (16.6 to 46.3)
4 High body-mass index	2.5 (1.3 to 4.2)	4 High body-mass index	3.1 (1.7 to 5.0)	25.5 (12.8 to 44.4)
5 Chewing tobacco	1.8 (1.4 to 2.2)	5 Chewing tobacco	2.0 (1.5 to 2.6)	12.0 (-6.0 to 33.6)
6 Diet low in calcium	1.4 (1.1 to 1.8)	6 Diet low in milk	1.6 (1.1 to 2.2)	16.5 (1.4 to 31.5)
7 Diet low in milk	1.4 (1.0 to 1.8)	7 Diet low in calcium	1.5 (1.2 to 2.0)	7.4 (-4.9 to 20.2)
8 Household air pollution from solid fuels	1.4 (1.0 to 1.8)	8 Diet low in whole grains	1.2 (0.5 to 1.7)	13.3 (-0.2 to 26.6)
9 Diet low in whole grains	1.1 (0.4 to 1.4)	9 Household air pollution from solid fuels	1.2 (0.7 to 1.6)	-15.7 (-31.0 to -1.2)
10 Diet low in fruits	1.0 (0.6 to 1.5)	10 Alcohol use	1.1 (0.9 to 1.3)	12.3 (-1.0 to 27.7)
11 Alcohol use	1.0 (0.8 to 1.2)	12 Diet low in fruits	1.0 (0.6 to 1.5)	0.9 (-12.4 to 15.9)

Both sexes combined

Leading risk 2010	ASR of Deaths 2010	Leading risk 2019	ASR of Deaths 2019	Percentage change in ASR of Deaths 2010-2019
1 Smoking	19.3 (17.9 to 20.9)	1 Smoking	18.4 (16.6 to 20.6)	-4.6 (-12.8 to 4.7)
2 Unsafe sex	4.7 (4.2 to 5.8)	2 Unsafe sex	4.6 (3.9 to 5.6)	-2.6 (-13.3 to 8.6)
3 Alcohol use	3.6 (3.1 to 4.2)	3 Alcohol use	4.0 (3.4 to 4.8)	11.9 (-2.0 to 27.1)
4 High fasting plasma glucose	2.7 (0.7 to 5.5)	4 High fasting plasma glucose	3.4 (0.9 to 7.0)	26.5 (17.0 to 38.1)
5 Household air pollution from solid fuels	2.4 (1.6 to 3.2)	5 High body-mass index	2.9 (1.6 to 4.7)	29.7 (18.5 to 47.8)
6 High body-mass index	2.3 (1.1 to 3.8)	6 Chewing tobacco	2.1 (1.7 to 2.7)	6.1 (-7.7 to 22.4)
7 Chewing tobacco	2.0 (1.6 to 2.4)	7 Ambient particulate matter pollution	2.0 (1.3 to 2.6)	36.0 (15.0 to 65.8)
8 Diet low in calcium	1.6 (1.3 to 2.0)	8 Diet low in calcium	1.7 (1.3 to 2.2)	6.5 (-3.2 to 15.6)
9 Diet low in fruits	1.5 (0.9 to 2.3)	9 Household air pollution from solid fuels	1.7 (1.1 to 2.5)	-27.3 (-40.0 to -15.7)
10 Diet low in milk	1.5 (1.0 to 2.0)	10 Diet low in milk	1.7 (1.2 to 2.3)	14.2 (3.7 to 24.9)
11 Ambient particulate matter pollution	1.4 (0.9 to 2.0)	11 Diet low in fruits	1.5 (0.9 to 2.2)	-3.3 (-11.8 to 6.4)

F Low SDI

Males

Leading risk 2010	ASR of Deaths 2010	Leading risk 2019	ASR of Deaths 2019	Percentage change in ASR of Deaths 2010-2019
1 Smoking	21.4 (18.5 to 24.2)	1 Smoking	20.2 (17.2 to 23.0)	-5.6 (-16.1 to 5.8)
2 Alcohol use	5.3 (4.4 to 6.2)	2 Alcohol use	5.7 (4.7 to 6.8)	7.8 (-4.1 to 21.1)
3 Household air pollution from solid fuels	4.1 (2.5 to 6.2)	3 Household air pollution from solid fuels	3.3 (2.1 to 4.8)	-17.9 (-29.7 to -5.4)
4 High fasting plasma glucose	2.3 (0.6 to 5.1)	4 High fasting plasma glucose	2.6 (0.7 to 5.6)	12.3 (1.2 to 25.4)
5 Diet low in fruits	1.9 (1.1 to 2.9)	5 High body-mass index	2.2 (0.9 to 4.0)	26.0 (10.1 to 57.9)
6 Chewing tobacco	1.8 (1.3 to 2.4)	6 Diet low in fruits	1.8 (1.0 to 2.8)	-3.8 (-13.8 to 6.8)
7 High body-mass index	1.7 (0.6 to 3.4)	7 Diet low in calcium	1.8 (1.4 to 2.3)	5.3 (-8.2 to 18.2)
8 Diet low in calcium	1.7 (1.4 to 2.2)	8 Chewing tobacco	1.8 (1.2 to 2.4)	-2.2 (-19.3 to 15.7)
9 Diet low in milk	1.4 (0.9 to 1.9)	9 Ambient particulate matter pollution	1.6 (0.9 to 2.5)	37.0 (9.8 to 79.9)
10 Ambient particulate matter pollution	1.2 (0.6 to 2.1)	10 Diet low in milk	1.5 (1.0 to 2.0)	8.2 (-5.9 to 22.0)

Females

Leading risk 2010	ASR of Deaths 2010	Leading risk 2019	ASR of Deaths 2019	Percentage change in ASR of Deaths 2010-2019
1 Unsafe sex	16.0 (12.8 to 19.2)	1 Unsafe sex	15.1 (11.9 to 18.5)	-6.0 (-15.0 to 5.2)
2 Smoking	4.0 (3.2 to 4.9)	2 Smoking	3.9 (3.1 to 4.7)	-3.5 (-12.8 to 6.2)
3 High body-mass index	2.3 (1.0 to 4.1)	3 High body-mass index	2.9 (1.5 to 4.9)	27.9 (13.7 to 50.9)
4 High fasting plasma glucose	2.1 (0.5 to 4.5)	4 High fasting plasma glucose	2.7 (0.7 to 5.8)	27.1 (14.4 to 43.1)
5 Alcohol use	1.5 (1.2 to 1.7)	5 Alcohol use	1.7 (1.3 to 2.0)	12.7 (0.4 to 25.1)
6 Diet low in calcium	1.4 (1.1 to 1.7)	6 Diet low in calcium	1.5 (1.2 to 1.9)	9.4 (-1.5 to 22.1)
7 Chewing tobacco	1.4 (1.0 to 1.8)	7 Chewing tobacco	1.5 (1.1 to 2.0)	10.0 (-8.6 to 32.7)
8 Diet low in milk	1.2 (0.8 to 1.5)	8 Diet low in milk	1.3 (0.9 to 1.7)	13.2 (1.6 to 26.5)
9 Household air pollution from solid fuels	1.1 (0.8 to 1.8)	9 Household air pollution from solid fuels	1.2 (0.8 to 1.6)	2.1 (-13.7 to 19.0)
10 Diet low in fruits	1.0 (0.5 to 1.6)	10 Diet low in whole grains	1.1 (0.4 to 1.4)	11.8 (0.9 to 24.9)
11 Diet low in whole grains	1.0 (0.4 to 1.3)	11 Diet low in fruits	1.0 (0.5 to 1.6)	-1.2 (-11.5 to 10.9)

Both sexes combined

Leading risk 2010	ASR of Deaths 2010	Leading risk 2019	ASR of Deaths 2019	Percentage change in ASR of Deaths 2010-2019
1 Smoking	12.5 (10.9 to 14.1)	1 Smoking	11.8 (10.2 to 13.2)	-5.6 (-14.8 to 4.4)
2 Unsafe sex	8.1 (6.5 to 9.7)	2 Unsafe sex	7.7 (6.1 to 9.4)	-5.4 (-14.4 to 5.8)
3 Alcohol use	3.3 (2.8 to 3.9)	3 Alcohol use	3.6 (3.0 to 4.3)	8.5 (-2.1 to 20.7)
4 Household air pollution from solid fuels	2.6 (1.7 to 3.8)	4 High fasting plasma glucose	2.6 (0.7 to 5.5)	19.7 (11.7 to 30.0)
5 High fasting plasma glucose	2.2 (0.6 to 4.6)	5 High body-mass index	2.6 (1.3 to 4.3)	27.1 (14.1 to 51.0)
6 High body-mass index	2.0 (0.9 to 3.6)	6 Household air pollution from solid fuels	2.2 (1.5 to 3.1)	-13.5 (-25.4 to -1.9)
7 Chewing tobacco	1.6 (1.2 to 1.9)	7 Diet low in calcium	1.7 (1.3 to 2.1)	7.2 (-3.0 to 17.2)
8 Diet low in calcium	1.6 (1.2 to 1.9)	8 Chewing tobacco	1.6 (1.3 to 2.0)	3.1 (-9.6 to 17.9)
9 Diet low in fruits	1.5 (0.8 to 2.2)	9 Diet low in fruits	1.4 (0.8 to 2.1)	-3.0 (-11.3 to 5.7)
10 Diet low in milk	1.3 (0.8 to 1.7)	10 Diet low in milk	1.4 (0.9 to 1.9)	10.6 (-0.4 to 21.7)

Appendix Figure 19: Leading risk factors at the most detailed level for attributable cancer age-standardised death rates, 2010-2019 for (A) the global level, (B) high SDI quintile, (C) high-middle SDI quintile, (D) middle SDI quintile, (E) low-middle SDI quintile, and (F) low SDI quintile, for both sexes combined, males, and females. Data in parentheses are 95% uncertainty intervals (UIs). Rows are color-coded by the following: red color = environmental and occupational risk factors; blue color = behavioural risk factors; green color: = metabolic risk factors.

Dashed lines indicate decrease in rank; solid lines indicate increase or no change in rank. Risk factors at the most detailed level reflect the GBD hierarchy in which these categories of risks fall, ranging from Levels 2-4 (for more information on risk factor levels in the GBD hierarchy see Appendix table 9, p152). ASR = age-standardised rate; SDI = Socio-demographic Index; DALYs = disability-adjusted life-years.

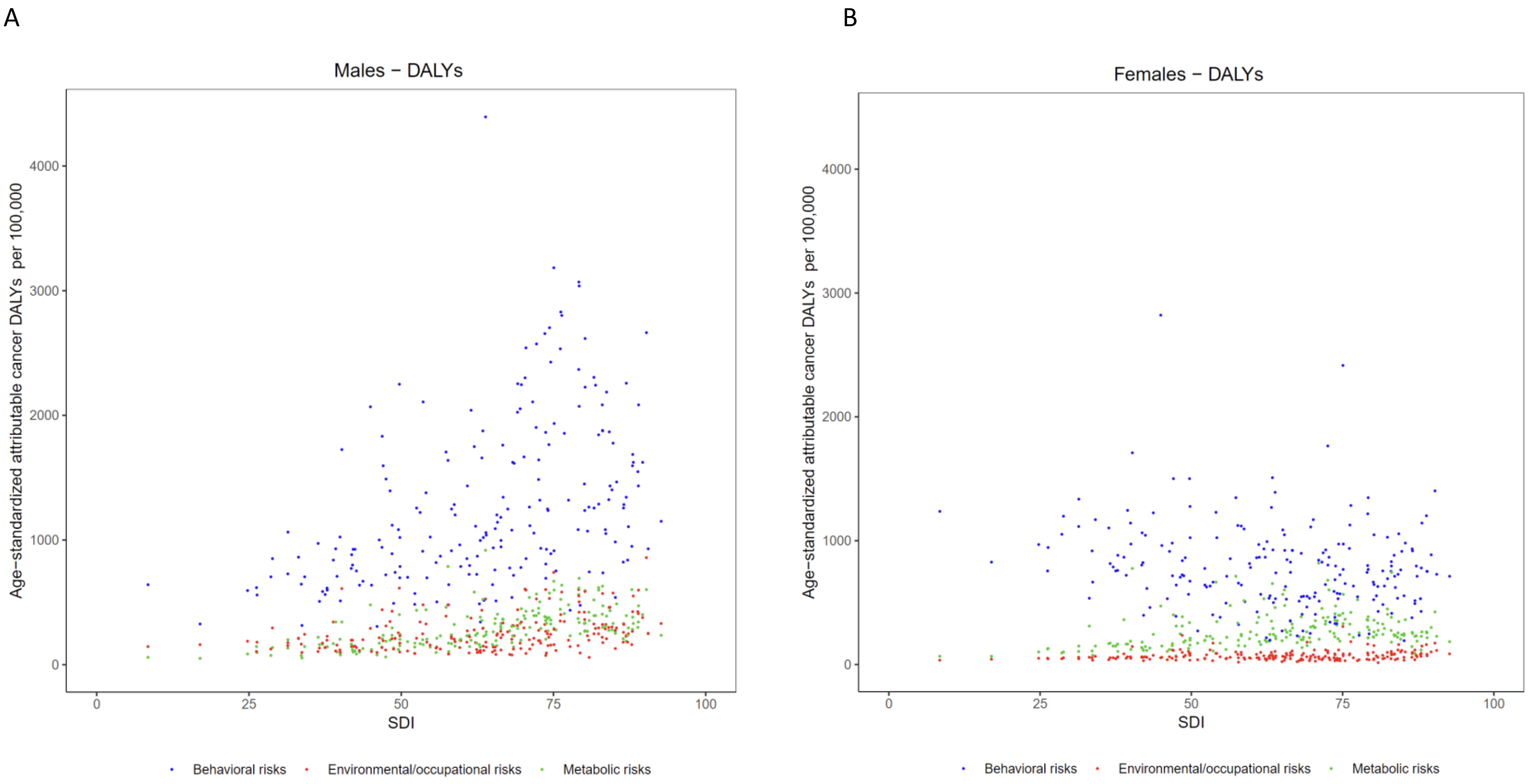

Appendix Figure 20: Level 1 risk-attributable age-standardised cancer DALY rates per 100,000, by SDI value for (A) males and (B) females. Datapoints in this figure represent each country estimated in the GBD study, ordered by its respective SDI value. Figure is color-coded by the following: blue = behavioural risks; red = environmental and occupational risks; green = metabolic risks. SDI = Socio-demographic Index; DALYs = disability-adjusted life-years.

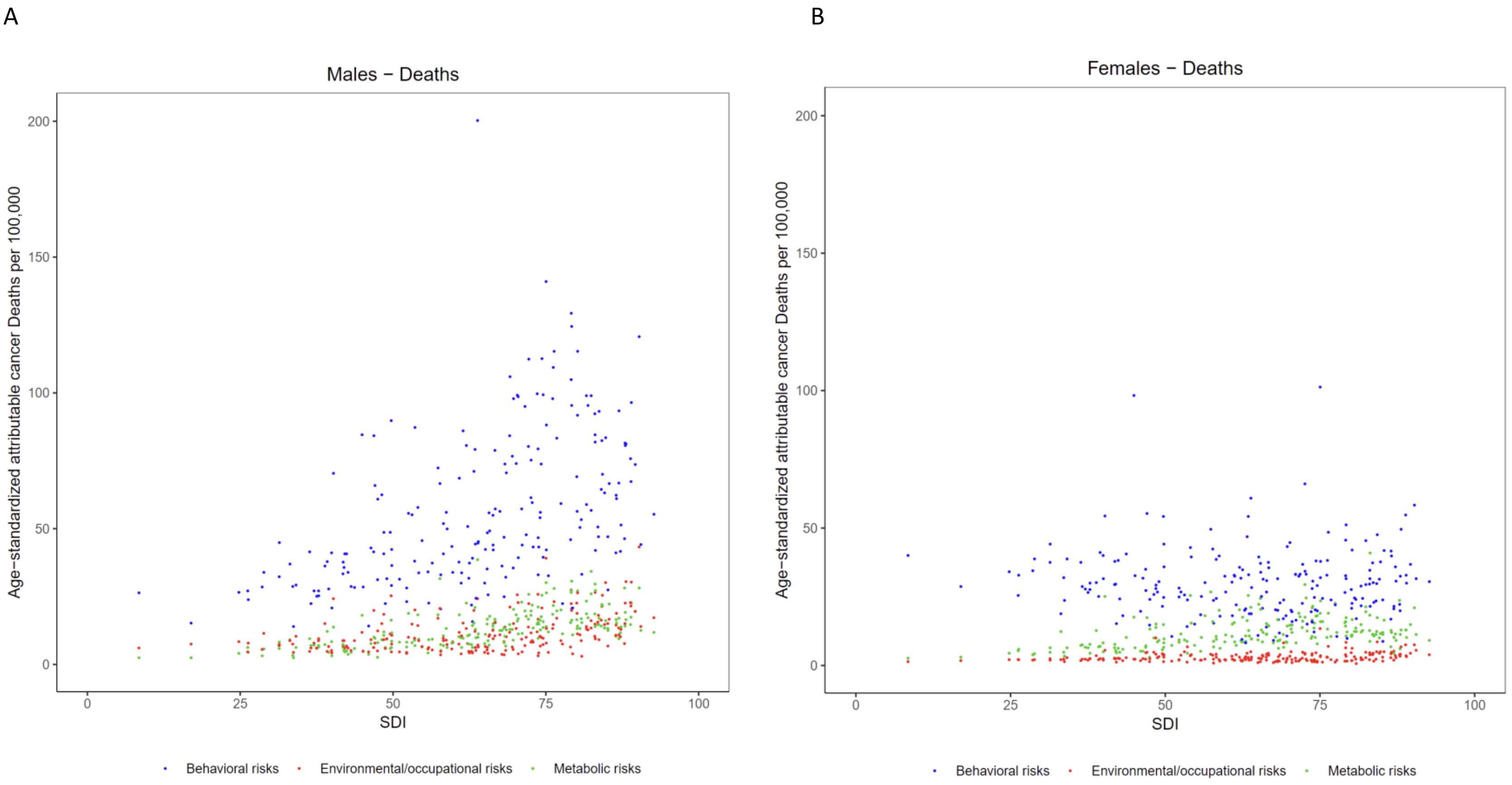

Appendix Figure 21: Level 1 risk-attributable age-standardised cancer mortality rates per 100,000, by SDI value for (A) males and (B) females. Datapoints in this figure represent each country estimated in the GBD study, ordered by its respective SDI value. Figure is color-coded by the following: blue = behavioural risks; red = environmental and occupational risks; green = metabolic risks. SDI = Socio-demographic Index.

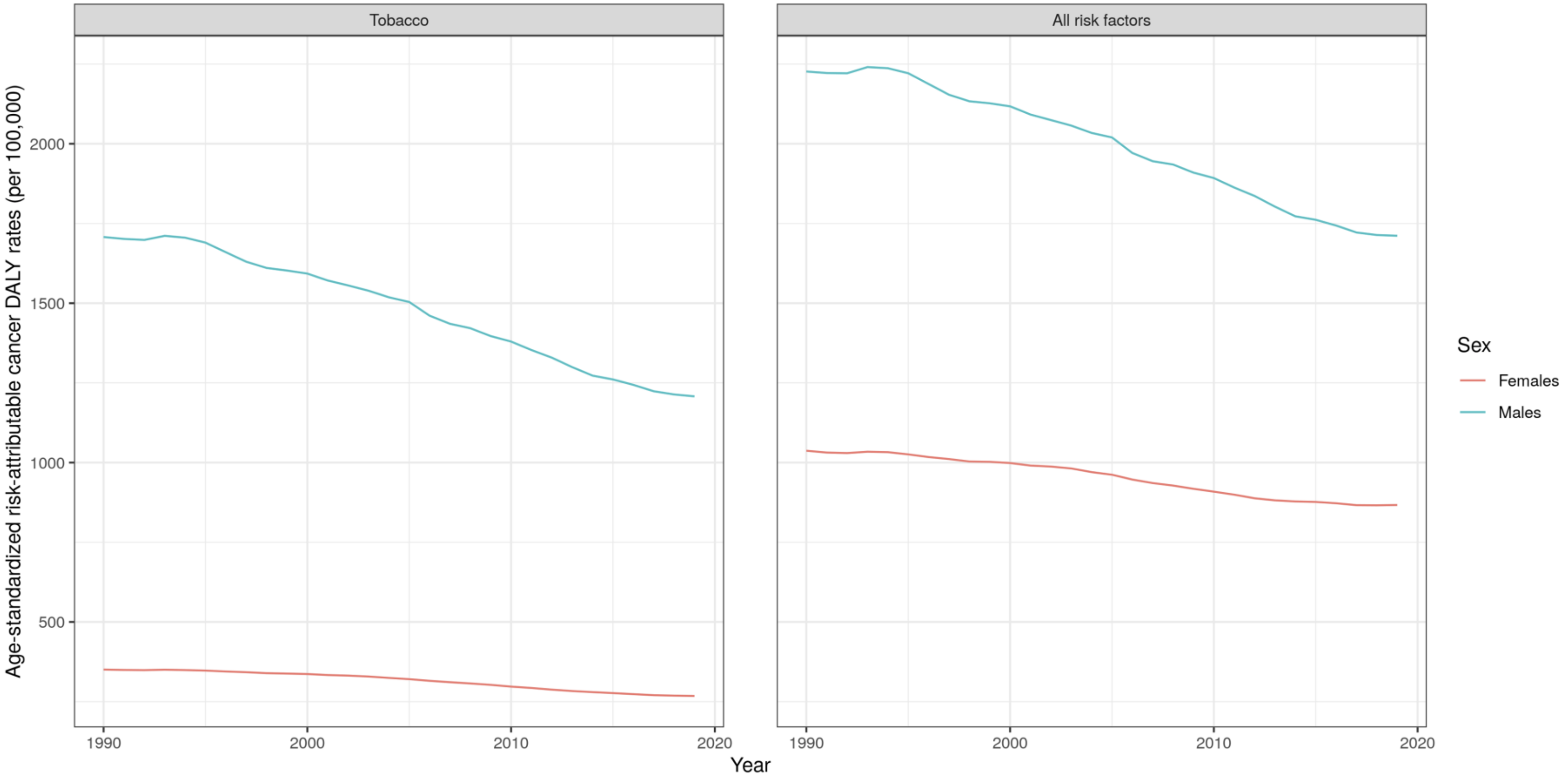

Appendix Figure 22: Trends in risk-attributable age-standardised cancer DALY rates, 1990-2019, for tobacco (left) and all risk factors estimated (right), by sex. DALY = disability-adjusted life-year.

Appendix Table 12: Global risk-attributable cancer deaths and DALYs in males and females reported in all-age numbers, age-standardised rates, and percentages of total cancer deaths and DALYs in 2019

Risk Factor Level in GBD	Risk factor	Male						Female					
		**Deaths, thousands (95% UI)**	**Age-standardised Mortality rate, per 100,000 (95% UI)**	**Percentage of risk-attributable cancer deaths out of total cancer (risk + non-risk) deaths (95% UI)**	**DALYs, thousands (95% UI)**	**Age-standardised DALY rate, per 100,000 (95% UI)**	**Percentage of risk-attributable cancer DALYs out of total cancer (risk + non-risk) DALYs (95% UI)**	**Deaths, thousands (95% UI)**	**Age-standardised Mortality rate, per 100,000 (95% UI)**	**Percentage of risk-attributable cancer deaths out of total cancer (risk + non-risk) deathss (95% UI)**	**DALYs, thousands (95% UI)**	**Age-standardised DALY rate, per 100,000 (95% UI)**	**Percentage of risk-attributable cancer DALYs out of total cancer (risk + non-risk) DALYs (95% UI)**
**0**	**All risk factors**	**2 880 (2 600 to 3 180)**	**77.6 (70.2 to 86.0)**	**50.6 (47.8 to 54.1)**	**67 500 (60 800 to 75 100)**	**1 711.6 (1 546.9 to 1 903.5)**	**48.0 (45.3 to 51.5)**	**1 580 (1 360 to 1 840)**	**36.1 (31.1 to 42.0)**	**36.3 (32.5 to 41.3)**	**37 600 (32 800 to 43 100)**	**866.9 (756.6 to 994.9)**	**34.3 (30.9 to 38.7)**
**1**	**Environmental/ occupational risks**	**538 (450 to 629)**	**14.7 (12.3 to 17.1)**	**9.5 (8.1 to 10.8)**	**11 900 (9 920 to 14 000)**	**304.9 (255.3 to 357.9)**	**8.5 (7.2 to 9.7)**	**198 (159 to 239)**	**4.5 (3.6 to 5.5)**	**4.6 (3.8 to 5.3)**	**4 380 (3 550 to 5 270)**	**100.6 (81.6 to 121.1)**	**4.0 (3.3 to 4.7)**
2	Air pollution	268 (197 to 344)	7.1 (5.2 to 9.1)	4.7 (3.6 to 5.9)	6 250 (4 640 to 8 070)	157.9 (116.8 to 203.6)	4.4 (3.4 to 5.5)	120 (88.8 to 151)	2.7 (2.0 to 3.4)	2.8 (2.1 to 3.4)	2 700 (2 000 to 3 390)	62.0 (45.9 to 77.9)	2.5 (1.9 to 3.1)
3	Particulate matter pollution	268 (197 to 344)	7.1 (5.2 to 9.1)	4.7 (3.6 to 5.9)	6 250 (4 640 to 8 070)	157.9 (116.8 to 203.6)	4.4 (3.4 to 5.5)	120 (88.8 to 151)	2.7 (2.0 to 3.4)	2.8 (2.1 to 3.4)	2 700 (2 000 to 3 390)	62.0 (45.9 to 77.9)	2.5 (1.9 to 3.1)
4	Ambient particulate matter pollution	216 (157 to 281)	5.8 (4.2 to 7.5)	3.8 (2.8 to 4.8)	5 000 (3 620 to 6 500)	126.5 (91.7 to 164.2)	3.6 (2.6 to 4.5)	91.3 (65.5 to 119)	2.1 (1.5 to 2.7)	2.1 (1.5 to 2.7)	2 020 (1 460 to 2 630)	46.3 (33.5 to 60.2)	1.8 (1.3 to 2.3)
4	Household air pollution from solid fuels	51.2 (27.7 to 82.2)	1.3 (0.7 to 2.1)	0.9 (0.5 to 1.5)	1 260 (687 to 2 010)	31.4 (17.1 to 50.2)	0.9 (0.5 to 1.4)	28.5 (16.0 to 44.5)	0.7 (0.4 to 1.0)	0.7 (0.4 to 1.0)	679 (384 to 1 050)	15.6 (8.8 to 24.2)	0.6 (0.4 to 1.0)
2	Other environmental risks	56.8 (11.3 to 110)	1.5 (0.3 to 2.9)	1.0 (0.2 to 1.9)	1 300 (258 to 2 540)	33.0 (6.5 to 64.4)	0.9 (0.2 to 1.8)	26.9 (5.22 to 52.2)	0.6 (0.1 to 1.2)	0.6 (0.1 to 1.2)	586 (115 to 1 140)	13.4 (2.6 to 26.0)	0.5 (0.1 to 1.0)
3	Residential radon	56.8 (11.3 to 110)	1.5 (0.3 to 2.9)	1.0 (0.2 to 1.9)	1 300 (258 to 2 540)	33.0 (6.5 to 64.4)	0.9 (0.2 to 1.8)	26.9 (5.22 to 52.2)	0.6 (0.1 to 1.2)	0.6 (0.1 to 1.2)	586 (115 to 1 140)	13.4 (2.6 to 26.0)	0.5 (0.1 to 1.0)
2	Occupational risks	267 (206 to 331)	7.5 (5.8 to 9.3)	4.7 (3.7 to 5.8)	5 520 (4 270 to 6 830)	144.4 (112.0 to 178.4)	3.9 (3.1 to 4.8)	66.5 (48.7 to 85.0)	1.5 (1.1 to 1.9)	1.5 (1.2 to 1.9)	1 440 (1 070 to 1 830)	33.1 (24.5 to 42.0)	1.3 (1.0 to 1.6)
3	Occupational carcinogens	267 (206 to 331)	7.5 (5.8 to 9.3)	4.7 (3.7 to 5.8)	5 520 (4 270 to 6 830)	144.4 (112.0 to 178.4)	3.9 (3.1 to 4.8)	66.5 (48.7 to 85.0)	1.5 (1.1 to 1.9)	1.5 (1.2 to 1.9)	1 440 (1 070 to 1 830)	33.1 (24.5 to 42.0)	1.3 (1.0 to 1.6)
4	Occupational exposure to asbestos	195 (139 to 255)	5.8 (4.1 to 7.5)	3.4 (2.4 to 4.4)	3 430 (2 400 to 4 510)	93.8 (65.9 to 123.0)	2.4 (1.7 to 3.2)	40.3 (25.7 to 52.6)	0.9 (0.6 to 1.2)	0.9 (0.6 to 1.2)	691 (455 to 892)	15.8 (10.4 to 20.4)	0.6 (0.4 to 0.8)
4	Occupational exposure to arsenic	6.66 (1.05 to 12.1)	0.2 (0.0 to 0.3)	0.1 (0.0 to 0.2)	185 (32.3 to 336)	4.5 (0.8 to 8.2)	0.1 (0.0 to 0.2)	3.10 (0.494 to 5.69)	0.1 (0.0 to 0.1)	0.1 (0.0 to 0.1)	85.4 (14.6 to 155)	2.0 (0.3 to 3.6)	0.1 (0.0 to 0.1)
4	Occupational exposure to benzene	1.06 (0.313 to 1.73)	0.0 (0.0 to 0.0)	0.0 (0.0 to 0.0)	48.8 (14.2 to 80.2)	1.2 (0.4 to 2.0)	0.0 (0.0 to 0.1)	0.808 (0.264 to 1.34)	0.0 (0.0 to 0.0)	0.0 (0.0 to 0.0)	37.0 (12.2 to 61.0)	0.9 (0.3 to 1.5)	0.0 (0.0 to 0.1)
4	Occupational exposure to beryllium	0.203 (0.152 to 0.264)	0.0 (0.0 to 0.0)	0.0 (0.0 to 0.0)	5.81 (4.35 to 7.52)	0.1 (0.1 to 0.2)	0.0 (0.0 to 0.0)	0.0978 (0.0746 to 0.123)	0.0 (0.0 to 0.0)	0.0 (0.0 to 0.0)	2.77(2.12 to 3.47)	0.1 (0.0 to 0.1)	0.0 (0.0 to 0.0)
4	Occupational exposure to cadmium	0.488 (0.371 to 0.614)	0.0 (0.0 to 0.0)	0.0 (0.0 to 0.0)	14.0 (10.7 to 17.6)	0.3 (0.3 to 0.4)	0.0 (0.0 to 0.0)	0.224 (0.172 to 0.284)	0.0 (0.0 to 0.0)	0.0 (0.0 to 0.0)	6.31 (4.86 to 7.98)	0.1 (0.1 to 0.2)	0.0 (0.0 to 0.0)
4	Occupational exposure to chromium	1.03 (0.841 to 1.26)	0.0 (0.0 to 0.0)	0.0 (0.0 to 0.0)	29.5 (24.2 to 35.8)	0.7 (0.6 to 0.9)	0.0 (0.0 to 0.0)	0.466 (0.378 to 0.568)	0.0 (0.0 to 0.0)	0.0 (0.0 to 0.0)	13.2 (10.7 to 16.0)	0.3 (0.2 to 0.4)	0.0 (0.0 to 0.0)
4	Occupational exposure to diesel engine exhaust	14.7 (12.1 to 17.7)	0.4 (0.3 to 0.4)	0.3 (0.2 to 0.3)	422 (347 to 510)	10.2 (8.4 to 12.3)	0.3 (0.3 to 0.4)	5.00 (4.05 to 6.08)	0.1 (0.1 to 0.1)	0.1 (0.1 to 0.1)	141 (114 to 171)	3.2 (2.6 to 3.9)	0.1 (0.1 to 0.2)
4	Occupational exposure to formaldehyde	0.771 (0.578 to 1.00)	0.0 (0.0 to 0.0)	0.0 (0.0 to 0.0)	34.8 (26.0 to 45.1)	0.8 (0.6 to 1.1)	0.0 (0.0 to 0.0)	0.347 (0.267 to 0.435)	0.0 (0.0 to 0.0)	0.0 (0.0 to 0.0)	16.0 (12.3 to 20.2)	0.4 (0.3 to 0.5)	0.0 (0.0 to 0.0)
4	Occupational exposure to nickel	6.64 (0.412 to 17.4)	0.2 (0.0 to 0.4)	0.1 (0.0 to 0.3)	186 (14.2 to 480)	4.5 (0.3 to 11.6)	0.1 (0.0 to 0.3)	2.69 (0.162 to 7.07)	0.1 (0.0 to 0.2)	0.1 (0.0 to 0.2)	74.6 (5.61 to 193)	1.7 (0.1 to 4.4)	0.1 (0.0 to 0.2)

Risk Factor Level in GBD	Risk factor	Male						Female					
		**Deaths, thousands (95% UI)**	**Age-standardised Mortality rate, per 100,000 (95% UI)**	**Percentage of risk-attributable cancer deaths out of total cancer (risk + non-risk) deaths (95% UI)**	**DALYs, thousands (95% UI)**	**Age-standardised DALY rate, per 100,000 (95% UI)**	**Percentage of risk-attributable cancer DALYs out of total cancer (risk + non-risk) DALYs (95% UI)**	**Deaths, thousands (95% UI)**	**Age-standardised Mortality rate, per 100,000 (95% UI)**	**Percentage of risk-attributable cancer deaths out of total cancer (risk + non-risk) deathss (95% UI)**	**DALYs, thousands (95% UI)**	**Age-standardised DALY rate, per 100,000 (95% UI)**	**Percentage of risk-attributable cancer DALYs out of total cancer (risk + non-risk) DALYs (95% UI)**
4	Occupational exposure to polycyclic aromatic hydrocarbons	3.63 (2.80 to 4.59)	0.1 (0.1 to 0.1)	0.1 (0.1 to 0.1)	104 (80.3 to 131)	2.5 (1.9 to 3.1)	0.1 (0.1 to 0.1)	1.64 (1.30 to 2.05)	0.0 (0.0 to 0.0)	0.0 (0.0 to 0.0)	46.3 (36.6 to 57.4)	1.1 (0.8 to 1.3)	0.0 (0.0 to 0.1)
4	Occupational exposure to silica	40.5 (18.2 to 64.1)	1.0 (0.4 to 1.6)	0.7 (0.3 to 1.1)	1 130 (513 to 1 790)	27.4 (12.4 to 43.4)	0.8 (0.4 to 1.3)	12.5 (5.19 to 19.9)	0.3 (0.1 to 0.5)	0.3 (0.1 to 0.5)	344 (142 to 547)	7.9 (3.3 to 12.6)	0.3 (0.1 to 0.5)
4	Occupational exposure to sulfuric acid	3.59 (1.52 to 6.67)	0.1 (0.0 to 0.2)	0.1 (0.0 to 0.1)	113 (48.1 to 209)	2.7 (1.2 to 5.0)	0.1 (0.0 to 0.1)	0.438 (0.188 to 0.799)	0.0 (0.0 to 0.0)	0.0 (0.0 to 0.0)	13.5 (5.81 to 24.7)	0.3 (0.1 to 0.6)	0.0 (0.0 to 0.0)
4	Occupational exposure to trichloroethylene	0.0554 (0.0117 to 0.102)	0.0 (0.0 to 0.0)	0.0 (0.0 to 0.0)	1.74 (0.367 to 3.23)	0.0 (0.0 to 0.1)	0.0 (0.0 to 0.0)	0.0231 (0.00506 to 0.0433)	0.0 (0.0 to 0.0)	0.0 (0.0 to 0.0)	0.690 (0.150 to 1.29)	0.0 (0.0 to 0.0)	0.0 (0.0 to 0.0)
**1**	**Behavioural risks**	**2 550 (2 320 to 2 810)**	**68.7 (62.5 to 75.5)**	**44.9 (43.1 to 47.0)**	**59 900 (54 200 to 65 900)**	**1 517.0 (1 377.3 to 1 672.0)**	**42.6 (40.8 to 44.8)**	**1 150 (1 030 to 1 260)**	**26.3 (23.8 to 29.0)**	**26.4 (25.1 to 28.2)**	**27 900 (25 400 to 30 700)**	**646.3 (587.2 to 710.5)**	**25.5 (24.2 to 27.2)**
2	Tobacco	2 070 (1 870 to 2 270)	55.5 (50.3 to 61.0)	36.3 (34.8 to 37.9)	47 600 (42 900 to 52 700)	1 207.7 (1 087.4 to 1 334.9)	33.9 (32.3 to 35.4)	534 (476 to 585)	12.2 (10.9 to 13.4)	12.3 (11.5 to 13.1)	11 700 (10 600 to 12 900)	267.8 (242.1 to 294.9)	10.7 (9.9 to 11.5)
3	Smoking	2 030 (1 840 to 2 240)	54.6 (49.5 to 60.1)	35.7 (34.2 to 37.4)	46 700 (42 100 to 51 700)	1 184.6 (1 067.6 to 1 310.8)	33.2 (31.7 to 34.7)	462 (414 to 504)	10.5 (9.4 to 11.5)	10.6 (10.0 to 11.4)	9 750 (8 840 to 10 700)	222.9 (202.1 to 243.5)	8.9 (8.3 to 9.6)
3	Chewing tobacco	30.8 (21.2 to 41.2)	0.8 (0.5 to 1.1)	0.5 (0.4 to 0.7)	885 (602 to 1 190)	21.7 (14.8 to 29.2)	0.6 (0.4 to 0.8)	24.8 (18.6 to 31.9)	0.6 (0.4 to 0.7)	0.6 (0.4 to 0.7)	619 (467 to 799)	14.3 (10.8 to 18.4)	0.6 (0.4 to 0.7)
3	Secondhand smoke	66.5 (38.4 to 101)	1.8 (1.0 to 2.7)	1.2 (0.7 to 1.8)	1 540 (898 to 2 320)	38.8 (22.6 to 58.8)	1.1 (0.6 to 1.6)	64.0 (41.1 to 92.4)	1.5 (0.9 to 2.1)	1.5 (1.0 to 2.1)	1 680 (1 100 to 2 420)	39.0 (25.5 to 55.9)	1.5 (1.0 to 2.2)
2	Alcohol use	394 (346 to 444)	10.3 (9.0 to 11.6)	6.9 (6.2 to 7.6)	10 500 (9 180 to 11 800)	259.9 (227.8 to 292.9)	7.4 (6.7 to 8.2)	101 (87.6 to 115)	2.3 (2.0 to 2.6)	2.3 (2.1 to 2.6)	2 520 (2 220 to 2 850)	58.3 (51.4 to 65.9)	2.3 (2.0 to 2.6)
2	Drug use	41.8 (34.2 to 51.0)	1.1 (0.9 to 1.4)	0.7 (0.6 to 0.9)	966 (784 to 1 180)	24.5 (20.0 to 29.9)	0.7 (0.6 to 0.8)	29.6 (21.8 to 38.8)	0.7 (0.5 to 0.9)	0.7 (0.5 to 0.9)	645 (491 to 835)	14.8 (11.2 to 19.1)	0.6 (0.4 to 0.7)
2	Dietary risks	352 (255 to 487)	9.7 (7.0 to 13.4)	6.2 (4.6 to 8.6)	8 350 (6 060 to 11 600)	213.2 (155.0 to 296.5)	5.9 (4.4 to 8.3)	253 (191 to 334)	5.8 (4.4 to 7.6)	5.8 (4.5 to 7.6)	5 600 (4 280 to 7 320)	129.2 (98.7 to 168.7)	5.1 (4.0 to 6.7)
3	Diet low in fruits	88.0 (43.1 to 142)	2.4 (1.2 to 3.8)	1.5 (0.8 to 2.4)	2 100 (1 050 to 3 360)	52.8 (26.5 to 84.6)	1.5 (0.7 to 2.4)	40.4 (21.2 to 60.0)	0.9 (0.5 to 1.4)	0.9 (0.5 to 1.4)	902 (484 to 1 330)	20.7 (11.1 to 30.5)	0.8 (0.4 to 1.2)
3	Diet low in vegetables	11.6 (1.71 to 23.2)	0.3 (0.0 to 0.6)	0.2 (0.0 to 0.4)	289 (43.9 to 576)	7.2 (1.1 to 14.4)	0.2 (0.0 to 0.4)	5.60 (0.868 to 10.9)	0.1 (0.0 to 0.2)	0.1 (0.0 to 0.3)	131 (20.9 to 258)	3.0 (0.5 to 5.9)	0.1 (0.0 to 0.2)
3	Diet low in whole grains	95.0 (36.4 to 125)	2.7 (1.0 to 3.5)	1.7 (0.6 to 2.2)	2 210 (849 to 2 920)	57.1 (21.9 to 75.4)	1.6 (0.6 to 2.0)	76.5 (29.7 to 100)	1.7 (0.7 to 2.3)	1.8 (0.7 to 2.3)	1 590 (618 to 2 120)	36.6 (14.2 to 48.8)	1.5 (0.6 to 1.9)
3	Diet low in milk	92.1 (59.3 to 126)	2.6 (1.6 to 3.5)	1.6 (1.1 to 2.2)	2 200 (1 430 to 3 010)	56.5 (36.6 to 77.2)	1.6 (1.0 to 2.1)	74.4 (46.9 to 100)	1.7 (1.1 to 2.3)	1.7 (1.1 to 2.3)	1 600 (1 010 to 2 130)	36.8 (23.3 to 49.0)	1.5 (0.9 to 2.0)
3	Diet high in red meat	30.4 (7.90 to 57.8)	0.8 (0.2 to 1.6)	0.5 (0.1 to 1.0)	748 (205 to 1 390)	19.1 (5.2 to 35.4)	0.5 (0.1 to 1.0)	44.9 (25.1 to 69.2)	1.0 (0.6 to 1.6)	1.0 (0.6 to 1.6)	1 140 (647 to 1 670)	26.3 (15.0 to 38.7)	1.0 (0.6 to 1.5)
3	Diet high in processed meat	17.7 (6.03 to 27.2)	0.5 (0.2 to 0.8)	0.3 (0.1 to 0.5)	405 (139 to 623)	10.5 (3.6 to 16.1)	0.3 (0.1 to 0.4)	16.2 (5.61 to 24.9)	0.4 (0.1 to 0.6)	0.4 (0.1 to 0.6)	330 (117 to 509)	7.6 (2.7 to 11.7)	0.3 (0.1 to 0.5)
3	Diet low in fibre	10.8 (4.25 to 20.7)	0.3 (0.1 to 0.6)	0.2 (0.1 to 0.4)	251 (99.6 to 476)	6.5 (2.6 to 12.4)	0.2 (0.1 to 0.3)	9.74 (3.95 to 18.9)	0.2 (0.1 to 0.4)	0.2 (0.1 to 0.4)	197 (80.2 to 381)	4.6 (1.8 to 8.8)	0.2 (0.1 to 0.4)
3	Diet low in calcium	80.0 (56.7 to 109)	2.2 (1.6 to 3.0)	1.4 (1.0 to 1.9)	1 900 (1 360 to 2 580)	48.8 (34.9 to 66.1)	1.4 (1.0 to 1.8)	57.9 (40.2 to 80.5)	1.3 (0.9 to 1.8)	1.3 (0.9 to 1.8)	1 240 (887 to 1 700)	28.7 (20.5 to 39.2)	1.1 (0.8 to 1.5)
3	Diet high in sodium	49.4 (1.30 to 193)	1.3 (0.0 to 5.2)	0.9 (0.0 to 3.4)	1 180 (30.5 to 4 550)	29.9 (0.8 to 115.3)	0.8 (0.0 to 3.2)	24.7 (0.782 to 102)	0.6 (0.0 to 2.3)	0.6 (0.0 to 2.4)	555 (17.5 to 2 270)	12.8 (0.4 to 52.5)	0.5 (0.0 to 2.1)
2	Unsafe sex	NA	NA	NA	NA	NA	NA	280 (239 to 314)	6.5 (5.5 to 7.3)	6.5 (5.6 to 7.1)	8 960 (7 550 to 9 980)	210.6 (177.7 to 234.9)	8.2 (7.0 to 8.8)
2	Low physical activity	26.6 (6.38 to 52.4)	0.8 (0.2 to 1.6)	0.5 (0.1 to 0.9)	479 (112 to 952)	13.3 (3.1 to 26.4)	0.3 (0.1 to 0.7)	40.5 (18.4 to 68.4)	0.9 (0.4 to 1.6)	0.9 (0.4 to 1.6)	724 (338 to 1 210)	16.6 (7.8 to 27.7)	0.7 (0.3 to 1.1)
**1**	**Metabolic risks**	**453 (221 to 760)**	**12.4 (6.0 to 20.9)**	**8.0 (3.9 to 13.5)**	**10 400 (5 170 to 17 400)**	**266.8 (132.4 to 443.9)**	**7.4 (3.7 to 12.4)**	**412 (216 to 667)**	**9.4 (4.9 to 15.2)**	**9.5 (5.1 to 15.2)**	**8 970 (4 860 to 14 200)**	**204.7 (110.8 to 324.0)**	**8.2 (4.5 to 12.9)**

Risk Factor Level in GBD	Risk factor	Male						Female					
		**Deaths, thousands (95% UI)**	**Age-standardised Mortality rate, per 100,000 (95% UI)**	**Percentage of risk-attributable cancer deaths out of total cancer (risk + non-risk) deaths (95% UI)**	**DALYs, thousands (95% UI)**	**Age-standardised DALY rate, per 100,000 (95% UI)**	**Percentage of risk-attributable cancer DALYs out of total cancer (risk + non-risk) DALYs (95% UI)**	**Deaths, thousands (95% UI)**	**Age-standardised Mortality rate, per 100,000 (95% UI)**	**Percentage of risk-attributable cancer deaths out of total cancer (risk + non-risk) deathss (95% UI)**	**DALYs, thousands (95% UI)**	**Age-standardised DALY rate, per 100,000 (95% UI)**	**Percentage of risk-attributable cancer DALYs out of total cancer (risk + non-risk) DALYs (95% UI)**
2	High fasting plasma glucose	225 (55.5 to 482)	6.3 (1.6 to 13.4)	4.0 (1.0 to 8.3)	4 600 (1 120 to 9 900)	120.4 (29.5 to 257.7)	3.3 (0.8 to 7.0)	195 (53.4 to 410)	4.4 (1.2 to 9.3)	4.5 (1.2 to 9.2)	3 980 (1 090 to 8 400)	91.0 (24.8 to 192.1)	3.6 (1.0 to 7.5)
2	High body-mass index	236 (120 to 389)	6.3 (3.2 to 10.4)	4.2 (2.1 to 6.9)	6 010 (3 090 to 9 900)	150.7 (77.1 to 247.5)	4.3 (2.2 to 7.0)	226 (136 to 340)	5.2 (3.1 to 7.7)	5.2 (3.1 to 7.8)	5 160 (3 130 to 7 690)	117.8 (71.3 to 175.0)	4.7 (2.8 to 7.0)

All estimates in this table are for total risk-attributable cancer burden. Columns showing percentages were calculated as: (*Percentage of risk-attributable cancer deaths or DALYs*) / (*total deaths or DALYs of all 29 cancer types*), specific to sex. The number on the left of each risk factor indicates its level in the GBD hierarchy; for more information on risk factor levels in the GBD hierarchy see Appendix table 9 (p152–153). An expanded version of this table is presented in Appendix table 13 (p194–199), which includes each risk-outcome pair included in this analysis. DALYs = disability-adjusted life-years; NA = not applicable due to sex restriction; UI = uncertainty interval.

Appendix Table 13: Global risk-attributable cancer deaths and DALYs in males and females reported in all-age numbers, age-standardised rates, and percentages of risk-attributable cancer deaths and DALYs in 2019 for all risk-cancer pairs measured

Risk Level in GBD	Risk Factor	Cancer type	Male						Female					
			Deaths, thousands (95% UI)	Age-standardised Mortality rate, per 100,000 (95% UI)	Percentage of risk-attributable cancer deaths out of total cancer (risk + non-risk) deaths (95% UI)	DALYs, thousands (95% UI)	Age-standardised DALY rate, per 100,000 (95% UI)	Percentage of risk-attributable cancer DALYs out of total cancer (risk + non-risk) DALYs (95% UI)	Deaths, thousands (95% UI)	Age-standardised Mortality rate, per 100,000 (95% UI)	Percentage of risk-attributable cancer deaths out of total cancer (risk + non-risk) deathss (95% UI)	DALYs, thousands (95% UI)	Age-standardised DALY rate, per 100,000 (95% UI)	Percentage of risk-attributable cancer DALYs out of total cancer (risk + non-risk) DALYs (95% UI)
**0**	**All risk factors**	**Total cancers**	**2 880 (2 600 to 3 180)**	**77.6 (70.2 to 86.0)**	**50.6 (47.8 to 54.1)**	**67 500 (60 800 to 75 100)**	**1 711.6 (1 546.9 to 1 903.5)**	**48.0 (45.3 to 51.5)**	**1 580 (1 360 to 1 840)**	**36.1 (31.1 to 42.0)**	**36.3 (32.5 to 41.3)**	**37 600 (32 800 to 43 100)**	**866.9 (756.6 to 994.9)**	**34.3 (30.9 to 38.7)**
**1**	**Environmental/ occupational risks**	**Total cancers**	**538 (450 to 629)**	**14.7 (12.3 to 17.1)**	**9.5 (8.1 to 10.8)**	**11 900 (9 920 to 14 000)**	**304.9 (255.3 to 357.9)**	**8.5 (7.2 to 9.7)**	**198 (159 to 239)**	**4.5 (3.6 to 5.5)**	**4.6 (3.8 to 5.3)**	**4 380 (3 550 to 5 270)**	**100.6 (81.6 to 121.1)**	**4.0 (3.3 to 4.7)**
2	Air pollution	Total cancers	268 (197 to 344)	7.1 (5.2 to 9.1)	4.7 (3.6 to 5.9)	6 250 (4 640 to 8 070)	157.9 (116.8 to 203.6)	4.4 (3.4 to 5.5)	120 (88.8 to 151)	2.7 (2.0 to 3.4)	2.8 (2.1 to 3.4)	2 700 (2 000 to 3 390)	62.0 (45.9 to 77.9)	2.5 (1.9 to 3.1)
3	Particulate matter pollution	Total cancers	268 (197 to 344)	7.1 (5.2 to 9.1)	4.7 (3.6 to 5.9)	6 250 (4 640 to 8 070)	157.9 (116.8 to 203.6)	4.4 (3.4 to 5.5)	120 (88.8 to 151)	2.7 (2.0 to 3.4)	2.8 (2.1 to 3.4)	2 700 (2 000 to 3 390)	62.0 (45.9 to 77.9)	2.5 (1.9 to 3.1)
4	Ambient particulate matter pollution	Total cancers	216 (157 to 281)	5.8 (4.2 to 7.5)	3.8 (2.8 to 4.8)	5 000 (3 620 to 6 500)	126.5 (91.7 to 164.2)	3.6 (2.6 to 4.5)	91.3 (65.5 to 119)	2.1 (1.5 to 2.7)	2.1 (1.5 to 2.7)	2 020 (1 460 to 2 630)	46.3 (33.5 to 60.2)	1.8 (1.3 to 2.3)
4	Ambient particulate matter pollution	Tracheal, bronchus, and lung cancer	216 (157 to 281)	5.8 (4.2 to 7.5)	15.6 (11.7 to 19.6)	5 000 (3 620 to 6 500)	126.5 (91.7 to 164.2)	15.8 (11.8 to 19.8)	91.3 (65.5 to 119)	2.1 (1.5 to 2.7)	13.9 (10.3 to 17.6)	2 020 (1 460 to 2 630)	46.3 (33.5 to 60.2)	14.1 (10.5 to 18.0)
4	Household air pollution from solid fuels	Total cancers	51.2 (27.7 to 82.2)	1.3 (0.7 to 2.1)	0.9 (0.5 to 1.5)	1 260 (687 to 2 010)	31.4 (17.1 to 50.2)	0.9 (0.5 to 1.4)	28.5 (16.0 to 44.5)	0.7 (0.4 to 1.0)	0.7 (0.4 to 1.0)	679 (384 to 1 050)	15.6 (8.8 to 24.2)	0.6 (0.4 to 1.0)
4	Household air pollution from solid fuels	Tracheal, bronchus, and lung cancer	51.2 (27.7 to 82.2)	1.3 (0.7 to 2.1)	3.7 (2.0 to 5.9)	1 260 (687 to 2 010)	31.4 (17.1 to 50.2)	4.0 (2.2 to 6.3)	28.5 (16.0 to 44.5)	0.7 (0.4 to 1.0)	4.3 (2.5 to 6.7)	679 (384 to 1 050)	15.6 (8.8 to 24.2)	4.8 (2.8 to 7.3)
2	Other environmental risks	Total cancers	56.8 (11.3 to 110)	1.5 (0.3 to 2.9)	1.0 (0.2 to 1.9)	1 300 (258 to 2 540)	33.0 (6.5 to 64.4)	0.9 (0.2 to 1.8)	26.9 (5.22 to 52.2)	0.6 (0.1 to 1.2)	0.6 (0.1 to 1.2)	586 (115 to 1 140)	13.4 (2.6 to 26.0)	0.5 (0.1 to 1.0)
3	Residential radon	Total cancers	56.8 (11.3 to 110)	1.5 (0.3 to 2.9)	1.0 (0.2 to 1.9)	1 300 (258 to 2 540)	33.0 (6.5 to 64.4)	0.9 (0.2 to 1.8)	26.9 (5.22 to 52.2)	0.6 (0.1 to 1.2)	0.6 (0.1 to 1.2)	586 (115 to 1 140)	13.4 (2.6 to 26.0)	0.5 (0.1 to 1.0)
3	Residential radon	Tracheal, bronchus, and lung cancer	56.8 (11.3 to 110)	1.5 (0.3 to 2.9)	4.1 (0.8 to 8.0)	1 300 (258 to 2 540)	33.0 (6.5 to 64.4)	4.1 (0.8 to 8.0)	26.9 (5.22 to 52.2)	0.6 (0.1 to 1.2)	4.1 (0.8 to 7.9)	586 (115 to 1 140)	13.4 (2.6 to 26.0)	4.1 (0.8 to 7.9)
2	Occupational risks	Total cancers	267 (206 to 331)	7.5 (5.8 to 9.3)	4.7 (3.7 to 5.8)	5 520 (4 270 to 6 830)	144.4 (112.0 to 178.4)	3.9 (3.1 to 4.8)	66.5 (48.7 to 85.0)	1.5 (1.1 to 1.9)	1.5 (1.2 to 1.9)	1 440 (1 070 to 1 830)	33.1 (24.5 to 42.0)	1.3 (1.0 to 1.6)
3	Occupational carcinogens	Total cancers	267 (206 to 331)	7.5 (5.8 to 9.3)	4.7 (3.7 to 5.8)	5 520 (4 270 to 6 830)	144.4 (112.0 to 178.4)	3.9 (3.1 to 4.8)	66.5 (48.7 to 85.0)	1.5 (1.1 to 1.9)	1.5 (1.2 to 1.9)	1 440 (1 070 to 1 830)	33.1 (24.5 to 42.0)	1.3 (1.0 to 1.6)
4	Occupational exposure to asbestos	Total cancers	195 (139 to 255)	5.8 (4.1 to 7.5)	3.4 (2.4 to 4.4)	3 430 (2 400 to 4 510)	93.8 (65.9 to 123.0)	2.4 (1.7 to 3.2)	40.3 (25.7 to 52.6)	0.9 (0.6 to 1.2)	0.9 (0.6 to 1.2)	691 (455 to 892)	15.8 (10.4 to 20.4)	0.6 (0.4 to 0.8)
4	Occupational exposure to asbestos	Larynx cancer	3.45 (1.86 to 5.28)	0.1 (0.1 to 0.2)	3.3 (1.7 to 5.0)	65.8 (35.1 to 103)	1.8 (1.0 to 2.8)	2.4 (1.2 to 3.7)	0.234 (0.106 to 0.377)	0.0 (0.0 to 0.0)	1.3 (0.6 to 2.1)	4.25 (1.94 to 6.81)	0.1 (0.0 to 0.2)	0.9 (0.4 to 1.5)
4	Occupational exposure to asbestos	Tracheal, bronchus, and lung cancer	172 (116 to 231)	5.1 (3.5 to 6.8)	12.4 (8.3 to 16.6)	2 950 (1 940 to 3 990)	81.2 (53.7 to 109.4)	9.3 (6.1 to 12.7)	26.5 (15.6 to 37.2)	0.6 (0.4 to 0.8)	4.0 (2.4 to 5.5)	417 (251 to 585)	9.5 (5.7 to 13.3)	2.9 (1.8 to 4.0)
4	Occupational exposure to asbestos	Ovarian cancer	NA	NA	NA	NA	NA	NA	6.56 (2.95 to 10.7)	0.1 (0.1 to 0.2)	3.3 (1.5 to 5.4)	113 (50.1 to 185)	2.6 (1.1 to 4.2)	2.1 (1.0 to 3.4)
4	Occupational exposure to asbestos	Mesothelioma	19.8 (18.4 to 21.2)	0.6 (0.5 to 0.6)	93.2 (90.8 to 95.3)	413 (382 to 450)	10.8 (10.0 to 11.7)	87.6 (83.8 to 90.9)	7.03 (4.93 to 7.97)	0.2 (0.1 to 0.2)	87.5 (82.7 to 91.0)	157 (105 to 183)	3.6 (2.4 to 4.2)	79.4 (72.6 to 84.6)
4	Occupational exposure to arsenic	Total cancers	6.66 (1.05 to 12.1)	0.2 (0.0 to 0.3)	0.1 (0.0 to 0.2)	185 (32.3 to 336)	4.5 (0.8 to 8.2)	0.1 (0.0 to 0.2)	3.10 (0.494 to 5.69)	0.1 (0.0 to 0.1)	0.1 (0.0 to 0.1)	85.4 (14.6 to 155)	2.0 (0.3 to 3.6)	0.1 (0.0 to 0.1)

Risk Level in GBD	Risk Factor	Cancer type	Male						Female					
			**Deaths, thousands (95% UI)**	**Age-standardised Mortality rate, per 100,000 (95% UI)**	**Percentage of risk-attributable cancer deaths out of total cancer (risk + non-risk) deaths (95% UI)**	**DALYs, thousands (95% UI)**	**Age-standardised DALY rate, per 100,000 (95% UI)**	**Percentage of risk-attributable cancer DALYs out of total cancer (risk + non-risk) DALYs (95% UI)**	**Deaths, thousands (95% UI)**	**Age-standardised Mortality rate, per 100,000 (95% UI)**	**Percentage of risk-attributable cancer deaths out of total cancer (risk + non-risk) deathss (95% UI)**	**DALYs, thousands (95% UI)**	**Age-standardised DALY rate, per 100,000 (95% UI)**	**Percentage of risk-attributable cancer DALYs out of total cancer (risk + non-risk) DALYs (95% UI)**
4	Occupational exposure to arsenic	Tracheal, bronchus, and lung cancer	6.66 (1.05 to 12.1)	0.2 (0.0 to 0.3)	0.5 (0.1 to 0.9)	185 (32.3 to 336)	4.5 (0.8 to 8.2)	0.6 (0.1 to 1.0)	3.10 (0.494 to 5.69)	0.1 (0.0 to 0.1)	0.5 (0.1 to 0.9)	85.4 (14.6 to 155)	2.0 (0.3 to 3.6)	0.6 (0.1 to 1.1)
4	Occupational exposure to benzene	Total cancers	1.06 (0.313 to 1.73)	0.0 (0.0 to 0.0)	0.0 (0.0 to 0.0)	48.8 (14.2 to 80.2)	1.2 (0.4 to 2.0)	0.0 (0.0 to 0.1)	0.808 (0.264 to 1.34)	0.0 (0.0 to 0.0)	0.0 (0.0 to 0.0)	37.0 (12.2 to 61.0)	0.9 (0.3 to 1.5)	0.0 (0.0 to 0.1)
4	Occupational exposure to benzene	Leukaemia	1.06 (0.313 to 1.73)	0.0 (0.0 to 0.0)	0.6 (0.2 to 0.9)	48.8 (14.2 to 80.2)	1.2 (0.4 to 2.0)	0.7 (0.2 to 1.2)	0.808 (0.264 to 1.34)	0.0 (0.0 to 0.0)	0.6 (0.2 to 0.9)	37.0 (12.2 to 61.0)	0.9 (0.3 to 1.5)	0.7 (0.2 to 1.2)
4	Occupational exposure to beryllium	Total cancers	0.203 (0.152 to 0.264)	0.0 (0.0 to 0.0)	0.0 (0.0 to 0.0)	5.81 (4.35 to 7.52)	0.1 (0.1 to 0.2)	0.0 (0.0 to 0.0)	0.0978 (0.0746 to 0.123)	0.0 (0.0 to 0.0)	0.0 (0.0 to 0.0)	2.77 (2.12 to 3.47)	0.1 (0.0 to 0.1)	0.0 (0.0 to 0.0)
4	Occupational exposure to beryllium	Tracheal, bronchus, and lung cancer	0.203 (0.152 to 0.264)	0.0 (0.0 to 0.0)	0.0 (0.0 to 0.0)	5.81 (4.35 to 7.52)	0.1 (0.1 to 0.2)	0.0 (0.0 to 0.0)	0.0978 (0.0746 to 0.123)	0.0 (0.0 to 0.0)	0.0 (0.0 to 0.0)	2.77 (2.12 to 3.47)	0.1 (0.0 to 0.1)	0.0 (0.0 to 0.0)
4	Occupational exposure to cadmium	Total cancers	0.488 (0.371 to 0.614)	0.0 (0.0 to 0.0)	0.0 (0.0 to 0.0)	14.0 (10.7 to 17.6)	0.3 (0.3 to 0.4)	0.0 (0.0 to 0.0)	0.224 (0.172 to 0.284)	0.0 (0.0 to 0.0)	0.0 (0.0 to 0.0)	6.31 (4.86 to 7.98)	0.1 (0.1 to 0.2)	0.0 (0.0 to 0.0)
4	Occupational exposure to cadmium	Tracheal, bronchus, and lung cancer	0.488 (0.371 to 0.614)	0.0 (0.0 to 0.0)	0.0 (0.0 to 0.0)	14.0 (10.7 to 17.6)	0.3 (0.3 to 0.4)	0.0 (0.0 to 0.1)	0.224 (0.172 to 0.284)	0.0 (0.0 to 0.0)	0.0 (0.0 to 0.0)	6.31 (4.86 to 7.98)	0.1 (0.1 to 0.2)	0.0 (0.0 to 0.1)
4	Occupational exposure to chromium	Total cancers	1.03 (0.841 to 1.26)	0.0 (0.0 to 0.0)	0.0 (0.0 to 0.0)	29.5 (24.2 to 35.8)	0.7 (0.6 to 0.9)	0.0 (0.0 to 0.0)	0.466 (0.378 to 0.568)	0.0 (0.0 to 0.0)	0.0 (0.0 to 0.0)	13.2 (10.7 to 16.0)	0.3 (0.2 to 0.4)	0.0 (0.0 to 0.0)
4	Occupational exposure to chromium	Tracheal, bronchus, and lung cancer	1.03 (0.841 to 1.26)	0.0 (0.0 to 0.0)	0.1 (0.1 to 0.1)	29.5 (24.2 to 35.8)	0.7 (0.6 to 0.9)	0.1 (0.1 to 0.1)	0.466 (0.378 to 0.568)	0.0 (0.0 to 0.0)	0.1 (0.1 to 0.1)	13.2 (10.7 to 16.0)	0.3 (0.2 to 0.4)	0.1 (0.1 to 0.1)
4	Occupational exposure to diesel engine exhaust	Total cancers	14.7 (12.1 to 17.7)	0.4 (0.3 to 0.4)	0.3 (0.2 to 0.3)	422 (347 to 510)	10.2 (8.4 to 12.3)	0.3 (0.3 to 0.4)	5.00 (4.05 to 6.08)	0.1 (0.1 to 0.1)	0.1 (0.1 to 0.1)	141 (114 to 171)	3.2 (2.6 to 3.9)	0.1 (0.1 to 0.2)
4	Occupational exposure to diesel engine exhaust	Tracheal, bronchus, and lung cancer	14.7 (12.1 to 17.7)	0.4 (0.3 to 0.4)	1.1 (0.9 to 1.2)	422 (347 to 510)	10.2 (8.4 to 12.3)	1.3 (1.1 to 1.6)	5.00 (4.05 to 6.08)	0.1 (0.1 to 0.1)	0.8 (0.6 to 0.9)	141 (114 to 171)	3.2 (2.6 to 3.9)	1.0 (0.8 to 1.2)
4	Occupational exposure to formaldehyde	Total cancers	0.771 (0.578 to 1.00)	0.0 (0.0 to 0.0)	0.0 (0.0 to 0.0)	34.8 (26.0 to 45.1)	0.8 (0.6 to 1.1)	0.0 (0.0 to 0.0)	0.347 (0.267 to 0.435)	0.0 (0.0 to 0.0)	0.0 (0.0 to 0.0)	16.0 (12.3 to 20.2)	0.4 (0.3 to 0.5)	0.0 (0.0 to 0.0)
4	Occupational exposure to formaldehyde	Nasopharynx cancer	0.397 (0.245 to 0.605)	0.0 (0.0 to 0.0)	0.8 (0.5 to 1.2)	17.0 (10.2 to 26.0)	0.4 (0.2 to 0.6)	1.0 (0.6 to 1.5)	0.121 (0.0693 to 0.185)	0.0 (0.0 to 0.0)	0.6 (0.4 to 0.9)	5.30 (2.96 to 8.15)	0.1 (0.1 to 0.2)	0.8 (0.5 to 1.2)
4	Occupational exposure to formaldehyde	Leukaemia	0.374 (0.289 to 0.478)	0.0 (0.0 to 0.0)	0.2 (0.2 to 0.2)	17.7 (13.4 to 22.9)	0.4 (0.3 to 0.6)	0.3 (0.2 to 0.3)	0.226 (0.171 to 0.283)	0.0 (0.0 to 0.0)	0.2 (0.1 to 0.2)	10.7 (8.04 to 13.6)	0.3 (0.2 to 0.3)	0.2 (0.2 to 0.3)
4	Occupational exposure to nickel	Total cancers	6.64 (0.412 to 17.4)	0.2 (0.0 to 0.4)	0.1 (0.0 to 0.3)	186 (14.2 to 480)	4.5 (0.3 to 11.6)	0.1 (0.0 to 0.3)	2.69 (0.162 to 7.07)	0.1 (0.0 to 0.2)	0.1 (0.0 to 0.2)	74.6 (5.61 to 193)	1.7 (0.1 to 4.4)	0.1 (0.0 to 0.2)
4	Occupational exposure to nickel	Tracheal, bronchus, and lung cancer	6.64 (0.412 to 17.4)	0.2 (0.0 to 0.4)	0.5 (0.0 to 1.2)	186 (14.2 to 480)	4.5 (0.3 to 11.6)	0.6 (0.0 to 1.5)	2.69 (0.162 to 7.07)	0.1 (0.0 to 0.2)	0.4 (0.0 to 1.1)	74.6 (5.61 to 193)	1.7 (0.1 to 4.4)	0.5 (0.0 to 1.4)
4	Occupational exposure to polycyclic aromatic hydrocarbons	Total cancers	3.63 (2.80 to 4.59)	0.1 (0.1 to 0.1)	0.1 (0.1 to 0.1)	104 (80.3 to 131)	2.5 (1.9 to 3.1)	0.1 (0.1 to 0.1)	1.64 (1.30 to 2.05)	0.0 (0.0 to 0.0)	0.0 (0.0 to 0.0)	46.3 (36.6 to 57.4)	1.1 (0.8 to 1.3)	0.0 (0.0 to 0.1)
4	Occupational exposure to polycyclic aromatic hydrocarbons	Tracheal, bronchus, and lung cancer	3.63 (2.80 to 4.59)	0.1 (0.1 to 0.1)	0.3 (0.2 to 0.3)	104 (80.3 to 131)	2.5 (1.9 to 3.1)	0.3 (0.3 to 0.4)	1.64 (1.30 to 2.05)	0.0 (0.0 to 0.0)	0.2 (0.2 to 0.3)	46.3 (36.6 to 57.4)	1.1 (0.8 to 1.3)	0.3 (0.3 to 0.4)

Risk Level in GBD	Risk Factor	Cancer type	Male						Female					
			Deaths, thousands (95% UI)	Age-standardised Mortality rate, per 100,000 (95% UI)	Percentage of risk-attributable cancer deaths out of total cancer (risk + non-risk) deaths (95% UI)	DALYs, thousands (95% UI)	Age-standardised DALY rate, per 100,000 (95% UI)	Percentage of risk-attributable cancer DALYs out of total cancer (risk + non-risk) DALYs (95% UI)	Deaths, thousands (95% UI)	Age-standardised Mortality rate, per 100,000 (95% UI)	Percentage of risk-attributable cancer deaths out of total cancer (risk + non-risk) deathss (95% UI)	DALYs, thousands (95% UI)	Age-standardised DALY rate, per 100,000 (95% UI)	Percentage of risk-attributable cancer DALYs out of total cancer (risk + non-risk) DALYs (95% UI)
4	Occupational exposure to silica	Total cancers	40.5 (18.2 to 64.1)	1.0 (0.4 to 1.6)	0.7 (0.3 to 1.1)	1 130 (513 to 1 790)	27.4 (12.4 to 43.4)	0.8 (0.4 to 1.3)	12.5 (5.19 to 19.9)	0.3 (0.1 to 0.5)	0.3 (0.1 to 0.5)	344 (142 to 547)	7.9 (3.3 to 12.6)	0.3 (0.1 to 0.5)
4	Occupational exposure to silica	Tracheal, bronchus, and lung cancer	40.5 (18.2 to 64.1)	1.0 (0.4 to 1.6)	2.9 (1.3 to 4.6)	1 130 (513 to 1 790)	27.4 (12.4 to 43.4)	3.6 (1.6 to 5.6)	12.5 (5.19 to 19.9)	0.3 (0.1 to 0.5)	1.9 (0.8 to 3.0)	344 (142 to 547)	7.9 (3.3 to 12.6)	2.4 (1.0 to 3.8)
4	Occupational exposure to sulfuric acid	Total cancers	3.59 (1.52 to 6.67)	0.1 (0.0 to 0.2)	0.1 (0.0 to 0.1)	113 (48.1 to 209)	2.7 (1.2 to 5.0)	0.1 (0.0 to 0.1)	0.438 (0.188 to 0.799)	0.0 (0.0 to 0.0)	0.0 (0.0 to 0.0)	13.5 (5.81 to 24.7)	0.3 (0.1 to 0.6)	0.0 (0.0 to 0.0)
4	Occupational exposure to sulfuric acid	Larynx cancer	3.59 (1.52 to 6.67)	0.1 (0.0 to 0.2)	3.4 (1.4 to 6.2)	113 (48.1 to 209)	2.7 (1.2 to 5.0)	4.0 (1.7 to 7.3)	0.438 (0.188 to 0.799)	0.0 (0.0 to 0.0)	2.5 (1.0 to 4.5)	13.5 (5.81 to 24.7)	0.3 (0.1 to 0.6)	2.9 (1.2 to 5.3)
4	Occupational exposure to trichloroethylene	Total cancers	0.0554 (0.0117 to 0.102)	0.0 (0.0 to 0.0)	0.0 (0.0 to 0.0)	1.74 (0.367 to 3.23)	0.0 (0.0 to 0.1)	0.0 (0.0 to 0.0)	0.0231 (0.00506 to 0.0433)	0.0 (0.0 to 0.0)	0.0 (0.0 to 0.0)	0.690 (0.150 to 1.29)	0.0 (0.0 to 0.0)	0.0 (0.0 to 0.0)
4	Occupational exposure to trichloroethylene	Kidney cancer	0.0554 (0.0117 to 0.102)	0.0 (0.0 to 0.0)	0.1 (0.0 to 0.1)	1.74 (0.367 to 3.23)	0.0 (0.0 to 0.1)	0.1 (0.0 to 0.1)	0.0231 (0.00506 to 0.0433)	0.0 (0.0 to 0.0)	0.0 (0.0 to 0.1)	0.690 (0.150 to 1.29)	0.0 (0.0 to 0.0)	0.1 (0.0 to 0.1)
**1**	**Behavioural risks**	**Total cancers**	**2 550 (2 320 to 2 810)**	**68.7 (62.5 to 75.5)**	**44.9 (43.1 to 47.0)**	**59 900 (54 200 to 65 900)**	**1 517.0 (1 377.3 to 1 672.0)**	**42.6 (40.8 to 44.8)**	**1 150 (1 030 to 1 260)**	**26.3 (23.8 to 29.0)**	**26.4 (25.1 to 28.2)**	**27 900 (25 400 to 30 700)**	**646.3 (587.2 to 710.5)**	**25.5 (24.2 to 27.2)**
2	Tobacco	Total cancers	2 070 (1 870 to 2 270)	55.5 (50.3 to 61.0)	36.3 (34.8 to 37.9)	47 600 (42 900 to 52 700)	1 207.7 (1 087.4 to 1 334.9)	33.9 (32.3 to 35.4)	534 (476 to 585)	12.2 (10.9 to 13.4)	12.3 (11.5 to 13.1)	11 700 (10 600 to 12 900)	267.8 (242.1 to 294.9)	10.7 (9.9 to 11.5)
3	Smoking	Total cancers	2 030 (1 840 to 2 240)	54.6 (49.5 to 60.1)	35.7 (34.2 to 37.4)	46 700 (42 100 to 51 700)	1 184.6 (1 067.6 to 1 310.8)	33.2 (31.7 to 34.7)	462 (414 to 504)	10.5 (9.4 to 11.5)	10.6 (10.0 to 11.4)	9 750 (8 840 to 10 700)	222.9 (202.1 to 243.5)	8.9 (8.3 to 9.6)
3	Smoking	Lip and oral cavity cancer	55.7 (45.1 to 66.7)	1.4 (1.2 to 1.7)	42.3 (35.2 to 48.6)	1 490 (1 190 to 1 800)	36.7 (29.2 to 44.3)	39.5 (31.9 to 46.2)	7.74 (5.72 to 9.73)	0.2 (0.1 to 0.2)	11.4 (8.8 to 14.0)	171 (124 to 220)	3.9 (2.8 to 5.0)	9.8 (7.3 to 12.2)
3	Smoking	Nasopharynx cancer	16.8 (12.2 to 21.5)	0.4 (0.3 to 0.5)	32.8 (24.0 to 41.0)	499 (357 to 647)	12.2 (8.8 to 15.7)	29.6 (21.2 to 37.5)	1.10 (0.692 to 1.55)	0.0 (0.0 to 0.0)	5.4 (3.4 to 7.4)	28.0 (17.3 to 40.3)	0.6 (0.4 to 0.9)	4.3 (2.6 to 6.0)
3	Smoking	Other pharynx cancer	49.1 (41.4 to 56.9)	1.2 (1.1 to 1.4)	55.8 (49.2 to 62.0)	1 330 (1 110 to 1 560)	32.6 (27.2 to 38.2)	53.3 (46.3 to 60.1)	4.54 (3.54 to 5.59)	0.1 (0.1 to 0.1)	17.4 (13.8 to 21.2)	109 (83.1 to 136)	2.5 (1.9 to 3.1)	14.9 (11.5 to 18.6)
3	Smoking	Oesophageal cancer	187 (156 to 219)	4.9 (4.1 to 5.8)	51.2 (47.0 to 55.1)	4 430 (3 690 to 5 190)	111.2 (92.7 to 130.3)	50.1 (46.0 to 54.0)	16.1 (12.9 to 19.3)	0.4 (0.3 to 0.4)	12.2 (10.3 to 14.3)	321 (260 to 384)	7.3 (5.9 to 8.8)	11.3 (9.4 to 13.4)
3	Smoking	Stomach cancer	155 (124 to 187)	4.2 (3.4 to 5.0)	25.3 (20.9 to 29.6)	3 480 (2 730 to 4 240)	88.7 (70.1 to 107.6)	24.0 (19.6 to 28.2)	16.9 (12.8 to 21.0)	0.4 (0.3 to 0.5)	4.9 (3.8 to 6.1)	328 (244 to 418)	7.5 (5.6 to 9.6)	4.3 (3.2 to 5.4)
3	Smoking	Colon and rectum cancer	114 (77.5 to 154)	3.1 (2.1 to 4.2)	19.2 (12.9 to 25.7)	2 640 (1 730 to 3 580)	67.5 (44.9 to 91.1)	18.9 (12.5 to 25.0)	28.6 (17.8 to 39.9)	0.7 (0.4 to 0.9)	5.8 (3.6 to 8.0)	587 (351 to 814)	13.4 (8.0 to 18.6)	5.7 (3.3 to 7.8)
3	Smoking	Liver cancer	77.3 (45.5 to 110)	2.0 (1.2 to 2.9)	23.2 (13.4 to 32.2)	1 960 (1 080 to 2 810)	48.6 (27.3 to 69.6)	21.6 (11.7 to 30.7)	8.54 (4.47 to 13.0)	0.2 (0.1 to 0.3)	5.7 (3.0 to 8.5)	170 (84.9 to 258)	3.9 (1.9 to 5.9)	4.9 (2.5 to 7.5)
3	Smoking	Pancreatic cancer	72.7 (60.5 to 85.1)	2.0 (1.6 to 2.3)	26.1 (22.4 to 29.9)	1 650 (1 360 to 1 950)	41.8 (34.7 to 49.3)	25.4 (21.6 to 29.1)	40.7 (32.8 to 48.8)	0.9 (0.7 to 1.1)	16.1 (13.3 to 19.0)	796 (658 to 940)	18.1 (15.0 to 21.4)	15.7 (13.0 to 18.3)
3	Smoking	Larynx cancer	73.4 (63.9 to 82.7)	1.9 (1.7 to 2.1)	69.5 (61.9 to 75.5)	1 910 (1 650 to 2 160)	47.3 (41.0 to 53.4)	68.1 (60.4 to 74.0)	4.87 (3.72 to 5.95)	0.1 (0.1 to 0.1)	27.4 (21.6 to 33.0)	117 (90.1 to 143)	2.7 (2.1 to 3.3)	25.3 (19.9 to 30.5)
3	Smoking	Tracheal, bronchus, and lung cancer	1 060 (952 to 1 170)	28.5 (25.7 to 31.3)	76.2 (74.6 to 77.8)	23 500 (21 100 to 26 000)	598.3 (538.9 to 661.3)	74.4 (72.7 to 76.1)	255 (230 to 277)	5.8 (5.2 to 6.3)	38.9 (36.7 to 40.9)	5 140 (4 710 to 5 550)	117.1 (107.3 to 126.4)	36.0 (33.7 to 38.1)
3	Smoking	Breast cancer	NA	NA	NA	NA	NA	NA	19.0 (13.6 to 24.8)	0.4 (0.3 to 0.6)	2.8 (1.9 to 3.6)	513 (362 to 674)	11.8 (8.3 to 15.5)	2.5 (1.8 to 3.3)
3	Smoking	Cervical cancer	NA	NA	NA	NA	NA	NA	30.1 (14.9 to 49.6)	0.7 (0.3 to 1.1)	10.8 (5.4 to 17.4)	894 (469 to 1 440)	20.8 (10.8 to 33.5)	10.0 (5.2 to 15.8)
3	Smoking	Prostate cancer	29.3 (12.8 to 46.6)	0.9 (0.4 to 1.4)	6.0 (2.7 to 9.3)	572 (253 to 918)	15.6 (6.9 to 25.0)	6.6 (3.0 to 10.1)	NA	NA	NA	NA	NA	NA
3	Smoking	Kidney cancer	25.0 (17.7 to 32.5)	0.7 (0.5 to 0.9)	23.0 (16.6 to 29.4)	581 (405 to 744)	14.8 (10.3 to 19.0)	21.2 (14.8 to 27.2)	5.15 (3.43 to 7.03)	0.1 (0.1 to 0.2)	8.9 (6.0 to 12.1)	106 (69.1 to 144)	2.4 (1.6 to 3.3)	8.0 (5.2 to 10.9)
3	Smoking	Bladder cancer	69.0 (52.1 to 85.5)	2.0 (1.5 to 2.5)	40.8 (31.5 to 49.9)	1 450 (1 130 to 1 770)	38.4 (29.6 to 46.8)	43.7 (34.0 to 51.8)	8.50 (5.87 to 11.2)	0.2 (0.1 to 0.3)	14.3 (10.0 to 18.6)	162 (113 to 210)	3.7 (2.6 to 4.8)	15.2 (10.9 to 19.4)

Risk Level in GBD	Risk Factor	Cancer type	Male						Female					
			**Deaths, thousands (95% UI)**	**Age-standardised Mortality rate, per 100,000 (95% UI)**	**Percentage of risk-attributable cancer deaths out of total cancer (risk + non-risk) deaths (95% UI)**	**DALYs, thousands (95% UI)**	**Age-standardised DALY rate, per 100,000 (95% UI)**	**Percentage of risk-attributable cancer DALYs out of total cancer (risk + non-risk) DALYs (95% UI)**	**Deaths, thousands (95% UI)**	**Age-standardised Mortality rate, per 100,000 (95% UI)**	**Percentage of risk-attributable cancer deaths out of total cancer (risk + non-risk) deathss (95% UI)**	**DALYs, thousands (95% UI)**	**Age-standardised DALY rate, per 100,000 (95% UI)**	**Percentage of risk-attributable cancer DALYs out of total cancer (risk + non-risk) DALYs (95% UI)**
3	Smoking	Leukaemia	50.1 (31.1 to 70.0)	1.4 (0.8 to 1.9)	26.6 (16.6 to 36.6)	1 220 (730 to 1 710)	31.0 (19.0 to 43.4)	18.2 (11.0 to 25.4)	14.5 (8.21 to 21.6)	0.3 (0.2 to 0.5)	9.9 (5.6 to 14.9)	314 (169 to 488)	7.2 (3.9 to 11.3)	6.3 (3.4 to 9.9)
3	Chewing tobacco	Total cancers	30.8 (21.2 to 41.2)	0.8 (0.5 to 1.1)	0.5 (0.4 to 0.7)	885 (602 to 1 190)	21.7 (14.8 to 29.2)	0.6 (0.4 to 0.8)	24.8 (18.6 to 31.9)	0.6 (0.4 to 0.7)	0.6 (0.4 to 0.7)	619 (467 to 799)	14.3 (10.8 to 18.4)	0.6 (0.4 to 0.7)
3	Chewing tobacco	Lip and oral cavity cancer	18.5 (11.0 to 27.3)	0.5 (0.3 to 0.7)	14.1 (8.6 to 20.0)	555 (326 to 820)	13.6 (8.0 to 20.1)	14.7 (9.0 to 21.0)	18.8 (13.9 to 24.1)	0.4 (0.3 to 0.6)	27.6 (21.5 to 33.8)	474 (349 to 611)	10.9 (8.0 to 14.1)	27.1 (20.9 to 33.2)
3	Chewing tobacco	Oesophageal cancer	12.3 (7.44 to 17.7)	0.3 (0.2 to 0.5)	3.4 (2.0 to 4.9)	330 (201 to 476)	8.1 (4.9 to 11.7)	3.8 (2.3 to 5.4)	6.00 (3.65 to 8.96)	0.1 (0.1 to 0.2)	4.5 (2.8 to 6.7)	145 (88.3 to 217)	3.3 (2.0 to 5.0)	5.1 (3.1 to 7.5)
3	Secondhand smoke	Total cancers	66.5 (38.4 to 101)	1.8 (1.0 to 2.7)	1.2 (0.7 to 1.8)	1 540 (898 to 2 320)	38.8 (22.6 to 58.8)	1.1 (0.6 to 1.6)	64.0 (41.1 to 92.4)	1.5 (0.9 to 2.1)	1.5 (1.0 to 2.1)	1 680 (1 100 to 2 420)	39.0 (25.5 to 55.9)	1.5 (1.0 to 2.2)
3	Secondhand smoke	Tracheal, bronchus, and lung cancer	66.3 (38.3 to 101)	1.8 (1.0 to 2.7)	4.8 (2.8 to 7.2)	1 530 (893 to 2 320)	38.7 (22.5 to 58.7)	4.8 (2.9 to 7.2)	47.2 (27.7 to 71.2)	1.1 (0.6 to 1.6)	7.2 (4.4 to 10.5)	1 130 (672 to 1 700)	25.9 (15.5 to 39.0)	7.9 (4.9 to 11.5)
3	Secondhand smoke	Breast cancer	0.184 (0.0457 to 0.323)	0.0 (0.0 to 0.0)	1.5 (0.4 to 2.7)	4.80 (1.19 to 8.48)	0.1 (0.0 to 0.2)	1.5 (0.4 to 2.6)	16.8 (3.96 to 29.0)	0.4 (0.1 to 0.7)	2.4 (0.6 to 4.3)	558 (134 to 968)	13.1 (3.1 to 22.7)	2.7 (0.7 to 4.7)
2	Alcohol use	Total cancers	394 (346 to 444)	10.3 (9.0 to 11.6)	6.9 (6.2 to 7.6)	10 500 (9 180 to 11 800)	259.9 (227.8 to 292.9)	7.4 (6.7 to 8.2)	101 (87.6 to 115)	2.3 (2.0 to 2.6)	2.3 (2.1 to 2.6)	2 520 (2 220 to 2 850)	58.3 (51.4 to 65.9)	2.3 (2.0 to 2.6)
2	Alcohol use	Lip and oral cavity cancer	52.9 (42.3 to 62.9)	1.4 (1.1 to 1.6)	40.2 (33.3 to 46.8)	1 540 (1 240 to 1 840)	37.9 (30.5 to 45.2)	41.1 (34.0 to 47.7)	7.50 (5.52 to 9.37)	0.2 (0.1 to 0.2)	11.1 (8.4 to 13.8)	183 (136 to 228)	4.2 (3.2 to 5.3)	10.5 (7.9 to 13.0)
2	Alcohol use	Nasopharynx cancer	22.1 (17.2 to 27.0)	0.5 (0.4 to 0.7)	43.2 (34.9 to 51.1)	736 (574 to 895)	17.9 (13.9 to 21.7)	43.7 (35.4 to 51.6)	2.35 (1.59 to 3.16)	0.1 (0.0 to 0.1)	11.5 (7.9 to 15.2)	74.1 (50.1 to 99.4)	1.7 (1.2 to 2.3)	11.4 (7.9 to 15.0)
2	Alcohol use	Other pharynx cancer	35.2 (27.3 to 43.5)	0.9 (0.7 to 1.1)	40.0 (31.8 to 48.1)	1 030 (801 to 1 270)	25.1 (19.5 to 30.9)	41.3 (32.8 to 49.5)	2.65 (1.82 to 3.53)	0.1 (0.0 to 0.1)	10.1 (7.1 to 13.3)	73.0 (50.5 to 97.9)	1.7 (1.2 to 2.3)	10.0 (7.1 to 13.1)
2	Alcohol use	Oesophageal cancer	104 (77.1 to 133)	2.7 (2.0 to 3.5)	28.4 (21.7 to 34.9)	2 610 (1 940 to 3 310)	64.8 (48.3 to 82.4)	29.6 (22.9 to 36.2)	9.72 (6.70 to 13.1)	0.2 (0.2 to 0.3)	7.3 (5.2 to 9.8)	209 (145 to 282)	4.8 (3.3 to 6.5)	7.4 (5.2 to 9.7)
2	Alcohol use	Colon and rectum cancer	78.7 (59.7 to 99.7)	2.2 (1.6 to 2.7)	13.3 (10.1 to 16.4)	1 940 (1 470 to 2 450)	49.4 (37.5 to 62.2)	13.9 (10.6 to 17.2)	22.0 (16.4 to 28.2)	0.5 (0.4 to 0.6)	4.5 (3.4 to 5.6)	467 (354 to 587)	10.7 (8.1 to 13.5)	4.5 (3.4 to 5.6)
2	Alcohol use	Liver cancer	76.8 (62.4 to 92.8)	2.0 (1.6 to 2.4)	23.0 (18.7 to 27.5)	1 940 (1 550 to 2 370)	48.4 (38.7 to 58.7)	21.5 (17.2 to 25.9)	19.2 (14.7 to 24.2)	0.4 (0.3 to 0.6)	12.7 (10.1 to 15.8)	439 (336 to 551)	10.1 (7.7 to 12.6)	12.6 (10.0 to 15.6)
2	Alcohol use	Larynx cancer	22.9 (13.7 to 31.1)	0.6 (0.3 to 0.8)	21.7 (12.9 to 29.2)	629 (380 to 853)	15.5 (9.3 to 21.0)	22.5 (13.6 to 30.2)	1.01 (0.487 to 1.55)	0.0 (0.0 to 0.0)	5.7 (2.8 to 8.6)	26.7 (13.0 to 40.8)	0.6 (0.3 to 0.9)	5.8 (2.9 to 8.7)
2	Alcohol use	Breast cancer	1.40 (1.10 to 1.74)	0.0 (0.0 to 0.0)	11.6 (9.3 to 14.1)	38.5 (30.0 to 48.4)	1.0 (0.7 to 1.2)	12.2 (9.8 to 15.0)	36.3 (29.5 to 43.5)	0.8 (0.7 to 1.0)	5.3 (4.3 to 6.3)	1 050 (850 to 1 260)	24.4 (19.7 to 29.2)	5.2 (4.2 to 6.2)
2	Drug use	Total cancers	41.8 (34.2 to 51.0)	1.1 (0.9 to 1.4)	0.7 (0.6 to 0.9)	966 (784 to 1 180)	24.5 (20.0 to 29.9)	0.7 (0.6 to 0.8)	29.6 (21.8 to 38.8)	0.7 (0.5 to 0.9)	0.7 (0.5 to 0.9)	645 (491 to 835)	14.8 (11.2 to 19.1)	0.6 (0.4 to 0.7)
2	Drug use	Liver cancer	41.8 (34.2 to 51.0)	1.1 (0.9 to 1.4)	12.5 (10.4 to 15.0)	966 (784 to 1 180)	24.5 (20.0 to 29.9)	10.7 (8.9 to 12.9)	29.6 (21.8 to 38.8)	0.7 (0.5 to 0.9)	19.6 (14.8 to 24.8)	645 (491 to 835)	14.8 (11.2 to 19.1)	18.5 (14.1 to 23.1)
2	Dietary risks	Total cancers	352 (255 to 487)	9.7 (7.0 to 13.4)	6.2 (4.6 to 8.6)	8 350 (6 060 to 11 600)	213.2 (155.0 to 296.5)	5.9 (4.4 to 8.3)	253 (191 to 334)	5.8 (4.4 to 7.6)	5.8 (4.5 to 7.6)	5 600 (4 280 to 7 320)	129.2 (98.7 to 168.7)	5.1 (4.0 to 6.7)
3	Diet low in fruits	Total cancers	88.0 (43.1 to 142)	2.4 (1.2 to 3.8)	1.5 (0.8 to 2.4)	2 100 (1 050 to 3 360)	52.8 (26.5 to 84.6)	1.5 (0.7 to 2.4)	40.4 (21.2 to 60.0)	0.9 (0.5 to 1.4)	0.9 (0.5 to 1.4)	902 (484 to 1 330)	20.7 (11.1 to 30.5)	0.8 (0.4 to 1.2)
3	Diet low in fruits	Oesophageal cancer	35.7 (10.0 to 78.8)	0.9 (0.3 to 2.1)	9.8 (2.8 to 21.9)	892 (263 to 1 930)	22.3 (6.5 to 48.4)	10.1 (2.9 to 22.2)	15.5 (5.19 to 30.4)	0.4 (0.1 to 0.7)	11.7 (4.0 to 23.1)	357 (129 to 668)	8.2 (3.0 to 15.4)	12.6 (4.6 to 23.8)
3	Diet low in fruits	Tracheal, bronchus, and lung cancer	52.3 (15.2 to 78.6)	1.4 (0.4 to 2.1)	3.8 (1.1 to 5.6)	1 200 (364 to 1 810)	30.5 (9.2 to 45.9)	3.8 (1.1 to 5.7)	24.9 (7.19 to 37.4)	0.6 (0.2 to 0.9)	3.8 (1.1 to 5.7)	545 (162 to 812)	12.5 (3.7 to 18.7)	3.8 (1.1 to 5.7)
3	Diet low in vegetables	Total cancers	11.6 (1.71 to 23.2)	0.3 (0.0 to 0.6)	0.2 (0.0 to 0.4)	289 (43.9 to 576)	7.2 (1.1 to 14.4)	0.2 (0.0 to 0.4)	5.60 (0.868 to 10.9)	0.1 (0.0 to 0.2)	0.1 (0.0 to 0.3)	131 (20.9 to 258)	3.0 (0.5 to 5.9)	0.1 (0.0 to 0.2)
3	Diet low in vegetables	Oesophageal cancer	11.6 (1.71 to 23.2)	0.3 (0.0 to 0.6)	3.2 (0.5 to 6.4)	289 (43.9 to 576)	7.2 (1.1 to 14.4)	3.3 (0.5 to 6.6)	5.60 (0.868 to 10.9)	0.1 (0.0 to 0.2)	4.2 (0.7 to 8.4)	131 (20.9 to 258)	3.0 (0.5 to 5.9)	4.6 (0.7 to 9.1)
3	Diet low in whole grains	Total cancers	95.0 (36.4 to 125)	2.7 (1.0 to 3.5)	1.7 (0.6 to 2.2)	2 210 (849 to 2 920)	57.1 (21.9 to 75.4)	1.6 (0.6 to 2.0)	76.5 (29.7 to 100)	1.7 (0.7 to 2.3)	1.8 (0.7 to 2.3)	1 590 (618 to 2 120)	36.6 (14.2 to 48.8)	1.5 (0.6 to 1.9)

Risk Level in GBD	Risk Factor	Cancer type	Male						Female					
			**Deaths, thousands (95% UI)**	**Age-standardised Mortality rate, per 100,000 (95% UI)**	**Percentage of risk-attributable cancer deaths out of total cancer (risk + non-risk) deaths (95% UI)**	**DALYs, thousands (95% UI)**	**Age-standardised DALY rate, per 100,000 (95% UI)**	**Percentage of risk-attributable cancer DALYs out of total cancer (risk + non-risk) DALYs (95% UI)**	**Deaths, thousands (95% UI)**	**Age-standardised Mortality rate, per 100,000 (95% UI)**	**Percentage of risk-attributable cancer deaths out of total cancer (risk + non-risk) deathss (95% UI)**	**DALYs, thousands (95% UI)**	**Age-standardised DALY rate, per 100,000 (95% UI)**	**Percentage of risk-attributable cancer DALYs out of total cancer (risk + non-risk) DALYs (95% UI)**
3	Diet low in whole grains	Colon and rectum cancer	95.0 (36.4 to 125)	2.7 (1.0 to 3.5)	16.0 (6.2 to 20.9)	2 210 (849 to 2 920)	57.1 (21.9 to 75.4)	15.9 (6.1 to 20.7)	76.5 (29.7 to 100)	1.7 (0.7 to 2.3)	15.6 (6.0 to 20.5)	1 590 (618 to 2 120)	36.6 (14.2 to 48.8)	15.4 (5.9 to 20.3)
3	Diet low in milk	Total cancers	92.1 (59.3 to 126)	2.6 (1.6 to 3.5)	1.6 (1.1 to 2.2)	2 200 (1 430 to 3 010)	56.5 (36.6 to 77.2)	1.6 (1.0 to 2.1)	74.4 (46.9 to 100)	1.7 (1.1 to 2.3)	1.7 (1.1 to 2.3)	1 600 (1 010 to 2 130)	36.8 (23.3 to 49.0)	1.5 (0.9 to 2.0)
3	Diet low in milk	Colon and rectum cancer	92.1 (59.3 to 126)	2.6 (1.6 to 3.5)	15.5 (10.1 to 20.9)	2 200 (1 430 to 3 010)	56.5 (36.6 to 77.2)	15.8 (10.3 to 21.1)	74.4 (46.9 to 100)	1.7 (1.1 to 2.3)	15.1 (9.8 to 20.5)	1 600 (1 010 to 2 130)	36.8 (23.3 to 49.0)	15.5 (10.1 to 20.9)
3	Diet high in red meat	Total cancers	30.4 (7.90 to 57.8)	0.8 (0.2 to 1.6)	0.5 (0.1 to 1.0)	748 (205 to 1 390)	19.1 (5.2 to 35.4)	0.5 (0.1 to 1.0)	44.9 (25.1 to 69.2)	1.0 (0.6 to 1.6)	1.0 (0.6 to 1.6)	1 140 (647 to 1 670)	26.3 (15.0 to 38.7)	1.0 (0.6 to 1.5)
3	Diet high in red meat	Colon and rectum cancer	30.1 (7.63 to 57.3)	0.8 (0.2 to 1.6)	5.1 (1.3 to 9.6)	738 (198 to 1 370)	18.8 (5.0 to 35.1)	5.3 (1.4 to 9.8)	22.8 (5.66 to 44.0)	0.5 (0.1 to 1.0)	4.6 (1.2 to 9.1)	496 (132 to 945)	11.4 (3.0 to 21.7)	4.8 (1.3 to 9.1)
3	Diet high in red meat	Breast cancer	0.362 (0.172 to 0.497)	0.0 (0.0 to 0.0)	3.0 (1.4 to 4.0)	9.71 (4.64 to 13.4)	0.2 (0.1 to 0.3)	3.1 (1.5 to 4.1)	22.1 (10.4 to 29.7)	0.5 (0.2 to 0.7)	3.2 (1.5 to 4.2)	641 (307 to 858)	14.9 (7.1 to 19.9)	3.2 (1.5 to 4.2)
3	Diet high in processed meat	Total cancers	17.7 (6.03 to 27.2)	0.5 (0.2 to 0.8)	0.3 (0.1 to 0.5)	405 (139 to 623)	10.5 (3.6 to 16.1)	0.3 (0.1 to 0.4)	16.2 (5.61 to 24.9)	0.4 (0.1 to 0.6)	0.4 (0.1 to 0.6)	330 (117 to 509)	7.6 (2.7 to 11.7)	0.3 (0.1 to 0.5)
3	Diet high in processed meat	Colon and rectum cancer	17.7 (6.03 to 27.2)	0.5 (0.2 to 0.8)	3.0 (1.0 to 4.6)	405 (139 to 623)	10.5 (3.6 to 16.1)	2.9 (1.0 to 4.5)	16.2 (5.61 to 24.9)	0.4 (0.1 to 0.6)	3.3 (1.2 to 5.1)	330 (117 to 509)	7.6 (2.7 to 11.7)	3.2 (1.2 to 4.9)
3	Diet low in fibre	Total cancers	10.8 (4.25 to 20.7)	0.3 (0.1 to 0.6)	0.2 (0.1 to 0.4)	251 (99.6 to 476)	6.5 (2.6 to 12.4)	0.2 (0.1 to 0.3)	9.74 (3.95 to 18.9)	0.2 (0.1 to 0.4)	0.2 (0.1 to 0.4)	197 (80.2 to 381)	4.6 (1.8 to 8.8)	0.2 (0.1 to 0.4)
3	Diet low in fibre	Colon and rectum cancer	10.8 (4.25 to 20.7)	0.3 (0.1 to 0.6)	1.8 (0.7 to 3.5)	251 (99.6 to 476)	6.5 (2.6 to 12.4)	1.8 (0.7 to 3.5)	9.74 (3.95 to 18.9)	0.2 (0.1 to 0.4)	2.0 (0.8 to 3.8)	197 (80.2 to 381)	4.6 (1.8 to 8.8)	1.9 (0.8 to 3.7)
3	Diet low in calcium	Total cancers	80.0 (56.7 to 109)	2.2 (1.6 to 3.0)	1.4 (1.0 to 1.9)	1 900 (1 360 to 2 580)	48.8 (34.9 to 66.1)	1.4 (1.0 to 1.8)	57.9 (40.2 to 80.5)	1.3 (0.9 to 1.8)	1.3 (0.9 to 1.8)	1 240 (887 to 1 700)	28.7 (20.5 to 39.2)	1.1 (0.8 to 1.5)
3	Diet low in calcium	Colon and rectum cancer	80.0 (56.7 to 109)	2.2 (1.6 to 3.0)	13.5 (9.7 to 18.2)	1 900 (1 360 to 2 580)	48.8 (34.9 to 66.1)	13.6 (9.9 to 18.3)	57.9 (40.2 to 80.5)	1.3 (0.9 to 1.8)	11.8 (8.4 to 16.1)	1 240 (887 to 1 700)	28.7 (20.5 to 39.2)	12.0 (8.7 to 16.4)
3	Diet high in sodium	Total cancers	49.4 (1.30 to 193)	1.3 (0.0 to 5.2)	0.9 (0.0 to 3.4)	1 180 (30.5 to 4 550)	29.9 (0.8 to 115.3)	0.8 (0.0 to 3.2)	24.7 (0.782 to 102)	0.6 (0.0 to 2.3)	0.6 (0.0 to 2.4)	555 (17.5 to 2 270)	12.8 (0.4 to 52.5)	0.5 (0.0 to 2.1)
3	Diet high in sodium	Stomach cancer	49.4 (1.30 to 193)	1.3 (0.0 to 5.2)	8.1 (0.2 to 31.6)	1 180 (30.5 to 4 550)	29.9 (0.8 to 115.3)	8.1 (0.2 to 31.7)	24.7 (0.782 to 102)	0.6 (0.0 to 2.3)	7.1 (0.2 to 29.5)	555 (17.5 to 2 270)	12.8 (0.4 to 52.5)	7.2 (0.2 to 29.4)
2	Unsafe sex	Total cancers	NA	NA	NA	NA	NA	NA	280 (239 to 314)	6.5 (5.5 to 7.3)	6.5 (5.6 to 7.1)	8 960 (7 550 to 9 980)	210.6 (177.7 to 234.9)	8.2 (7.0 to 8.8)
2	Unsafe sex	Cervical cancer	NA	NA	NA	NA	NA	NA	280 (239 to 314)	6.5 (5.5 to 7.3)	100.0 (100.0 to 100.0)	8 960 (7 550 to 9 980)	210.6 (177.7 to 234.9)	100.0 (100.0 to 100.0)
2	Low physical activity	Total cancers	26.6 (6.38 to 52.4)	0.8 (0.2 to 1.6)	0.5 (0.1 to 0.9)	479 (112 to 952)	13.3 (3.1 to 26.4)	0.3 (0.1 to 0.7)	40.5 (18.4 to 68.4)	0.9 (0.4 to 1.6)	0.9 (0.4 to 1.6)	724 (338 to 1 210)	16.6 (7.8 to 27.7)	0.7 (0.3 to 1.1)
2	Low physical activity	Colon and rectum cancer	26.6 (6.38 to 52.4)	0.8 (0.2 to 1.6)	4.5 (1.1 to 9.0)	479 (112 to 952)	13.3 (3.1 to 26.4)	3.4 (0.8 to 6.9)	32.1 (10.3 to 59.1)	0.7 (0.2 to 1.3)	6.5 (2.0 to 11.9)	526 (156 to 991)	12.0 (3.6 to 22.6)	5.1 (1.5 to 9.5)
2	Low physical activity	Breast cancer	NA	NA	NA	NA	NA	NA	8.48 (4.08 to 14.3)	0.2 (0.1 to 0.3)	1.2 (0.6 to 2.1)	198 (97.5 to 345)	4.6 (2.3 to 8.0)	1.0 (0.5 to 1.7)
**1**	**Metabolic risks**	**Total cancers**	**453 (221 to 760)**	**12.4 (6.0 to 20.9)**	**8.0 (3.9 to 13.5)**	**10 400 (5 170 to 17 400)**	**266.8 (132.4 to 443.9)**	**7.4 (3.7 to 12.4)**	**412 (216 to 667)**	**9.4 (4.9 to 15.2)**	**9.5 (5.1 to 15.2)**	**8 970 (4 860 to 14 200)**	**204.7 (110.8 to 324.0)**	**8.2 (4.5 to 12.9)**
2	High fasting plasma glucose	Total cancers	225 (55.5 to 482)	6.3 (1.6 to 13.4)	4.0 (1.0 to 8.3)	4 600 (1 120 to 9 900)	120.4 (29.5 to 257.7)	3.3 (0.8 to 7.0)	195 (53.4 to 410)	4.4 (1.2 to 9.3)	4.5 (1.2 to 9.2)	3 980 (1 090 to 8 400)	91.0 (24.8 to 192.1)	3.6 (1.0 to 7.5)
2	High fasting plasma glucose	Colon and rectum cancer	55.0 (10.2 to 124)	1.6 (0.3 to 3.6)	9.3 (1.7 to 20.9)	1 130 (205 to 2 570)	29.9 (5.4 to 67.8)	8.1 (1.5 to 18.5)	42.6 (7.86 to 98.7)	1.0 (0.2 to 2.2)	8.7 (1.6 to 19.6)	775 (144 to 1 790)	17.7 (3.3 to 40.9)	7.5 (1.4 to 17.1)
2	High fasting plasma glucose	Liver cancer	2.48 (0.514 to 5.78)	0.1 (0.0 to 0.2)	0.7 (0.2 to 1.8)	54.7 (11.3 to 128)	1.4 (0.3 to 3.3)	0.6 (0.1 to 1.4)	2.25 (0.401 to 5.15)	0.1 (0.0 to 0.1)	1.5 (0.3 to 3.4)	44.6 (8.04 to 104)	1.0 (0.2 to 2.4)	1.3 (0.2 to 3.0)
2	High fasting plasma glucose	Pancreatic cancer	25.9 (4.86 to 59.1)	0.7 (0.1 to 1.6)	9.3 (1.7 to 21.3)	538 (99.3 to 1 240)	14.0 (2.6 to 32.1)	8.3 (1.5 to 19.1)	22.4 (3.74 to 52.4)	0.5 (0.1 to 1.2)	8.9 (1.5 to 20.2)	406 (66.3 to 947)	9.3 (1.5 to 21.6)	8.0 (1.4 to 18.3)
2	High fasting plasma glucose	Tracheal, bronchus, and lung cancer	124 (21.1 to 286)	3.4 (0.6 to 7.9)	9.0 (1.5 to 20.6)	2 570 (433 to 5 910)	66.6 (11.2 to 153.4)	8.1 (1.4 to 18.8)	54.9 (10.8 to 129)	1.3 (0.2 to 2.9)	8.4 (1.6 to 19.2)	1 070 (212 to 2 550)	24.4 (4.8 to 58.2)	7.5 (1.4 to 17.4)

Risk Level in GBD	Risk Factor	Cancer type	Male						Female					
			**Deaths, thousands (95% UI)**	**Age-standardised Mortality rate, per 100,000 (95% UI)**	**Percentage of risk-attributable cancer deaths out of total cancer (risk + non-risk) deaths (95% UI)**	**DALYs, thousands (95% UI)**	**Age-standardised DALY rate, per 100,000 (95% UI)**	**Percentage of risk-attributable cancer DALYs out of total cancer (risk + non-risk) DALYs (95% UI)**	**Deaths, thousands (95% UI)**	**Age-standardised Mortality rate, per 100,000 (95% UI)**	**Percentage of risk-attributable cancer deaths out of total cancer (risk + non-risk) deathss (95% UI)**	**DALYs, thousands (95% UI)**	**Age-standardised DALY rate, per 100,000 (95% UI)**	**Percentage of risk-attributable cancer DALYs out of total cancer (risk + non-risk) DALYs (95% UI)**
2	High fasting plasma glucose	Breast cancer	NA	NA	NA	NA	NA	NA	51.1 (9.90 to 114)	1.2 (0.2 to 2.6)	7.4 (1.4 to 16.3)	1 240 (238 to 2 790)	28.5 (5.5 to 64.0)	6.1 (1.2 to 13.6)
2	High fasting plasma glucose	Ovarian cancer	NA	NA	NA	NA	NA	NA	15.7 (3.02 to 36.2)	0.4 (0.1 to 0.8)	7.9 (1.6 to 18.3)	354 (68.5 to 824)	8.1 (1.6 to 18.9)	6.6 (1.3 to 15.3)
2	High fasting plasma glucose	Bladder cancer	17.3 (2.99 to 38.6)	0.5 (0.1 to 1.2)	10.2 (1.8 to 22.9)	310 (53.4 to 694)	8.6 (1.5 to 19.1)	9.3 (1.6 to 20.9)	5.53 (1.01 to 12.5)	0.1 (0.0 to 0.3)	9.3 (1.7 to 20.9)	90.0 (16.2 to 203)	2.0 (0.4 to 4.6)	8.4 (1.6 to 19.1)
2	High body-mass index	Total cancers	236 (120 to 389)	6.3 (3.2 to 10.4)	4.2 (2.1 to 6.9)	6 010 (3 090 to 9 900)	150.7 (77.1 to 247.5)	4.3 (2.2 to 7.0)	226 (136 to 340)	5.2 (3.1 to 7.7)	5.2 (3.1 to 7.8)	5 160 (3 130 to 7 690)	117.8 (71.3 to 175.0)	4.7 (2.8 to 7.0)
2	High body-mass index	Oesophageal cancer	65.5 (12.6 to 136)	1.7 (0.3 to 3.5)	18.0 (3.6 to 37.2)	1 650 (321 to 3 420)	41.1 (7.9 to 85.1)	18.8 (3.8 to 38.5)	24.4 (1.30 to 53.9)	0.6 (0.0 to 1.2)	18.4 (1.0 to 40.3)	549 (29.1 to 1 200)	12.6 (0.7 to 27.5)	19.3 (1.0 to 41.7)
2	High body-mass index	Colon and rectum cancer	63.7 (33.8 to 102)	1.8 (0.9 to 2.8)	10.7 (5.7 to 17.2)	1 540 (831 to 2 430)	39.4 (21.2 to 62.3)	11.1 (6.0 to 17.5)	22.2 (9.89 to 39.9)	0.5 (0.2 to 0.9)	4.5 (2.0 to 8.1)	478 (215 to 860)	11.0 (4.9 to 19.8)	4.6 (2.0 to 8.3)
2	High body-mass index	Liver cancer	46.2 (15.5 to 93.4)	1.2 (0.4 to 2.4)	13.9 (4.7 to 27.9)	1 270 (425 to 2 560)	31.4 (10.4 to 63.2)	14.1 (4.8 to 28.2)	14.6 (2.51 to 32.2)	0.3 (0.1 to 0.7)	9.7 (1.7 to 20.6)	339 (58.9 to 748)	7.8 (1.4 to 17.2)	9.7 (1.7 to 20.7)
2	High body-mass index	Gallbladder and biliary tract cancer	5.83 (1.06 to 12.3)	0.2 (0.0 to 0.3)	8.0 (1.5 to 16.7)	134 (24.4 to 279)	3.4 (0.6 to 7.2)	8.5 (1.6 to 17.8)	20.3 (10.8 to 32.9)	0.5 (0.2 to 0.8)	20.4 (11.2 to 32.2)	434 (235 to 695)	9.9 (5.4 to 15.9)	21.3 (11.8 to 33.3)
2	High body-mass index	Pancreatic cancer	13.6 (-0.0832 to 33.2)	0.4 (0.0 to 0.9)	4.9 (0.0 to 11.9)	329 (-2.05 to 798)	8.3 (-0.1 to 20.1)	5.1 (0.0 to 12.4)	18.3 (6.48 to 32.8)	0.4 (0.1 to 0.7)	7.2 (2.5 to 12.7)	380 (133 to 674)	8.7 (3.0 to 15.4)	7.5 (2.6 to 13.1)
2	High body-mass index	Breast cancer	NA	NA	NA	NA	NA	NA	45.2 (18.8 to 81.2)	1.0 (0.4 to 1.8)	6.6 (2.7 to 11.9)	958 (306 to 1 820)	21.0 (6.3 to 40.6)	4.7 (1.5 to 9.1)
2	High body-mass index	Uterine cancer	NA	NA	NA	NA	NA	NA	36.5 (25.1 to 49.2)	0.8 (0.6 to 1.1)	39.8 (27.6 to 52.7)	936 (643 to 1 260)	21.5 (14.7 to 28.8)	40.2 (28.0 to 53.1)
2	High body-mass index	Ovarian cancer	NA	NA	NA	NA	NA	NA	6.31 (-0.177 to 14.3)	0.1 (0.0 to 0.3)	3.2 (-0.1 to 7.1)	168 (-4.67 to 380)	3.9 (-0.1 to 8.8)	3.1 (-0.1 to 7.0)
2	High body-mass index	Kidney cancer	17.8 (9.18 to 28.2)	0.5 (0.2 to 0.8)	16.4 (8.4 to 25.9)	446 (233 to 699)	11.2 (5.8 to 17.6)	16.3 (8.5 to 25.5)	13.9 (8.51 to 20.5)	0.3 (0.2 to 0.5)	24.0 (14.6 to 35.4)	306 (190 to 448)	7.0 (4.4 to 10.3)	23.2 (14.2 to 34.1)
2	High body-mass index	Thyroid cancer	2.21 (0.639 to 4.32)	0.1 (0.0 to 0.1)	11.9 (3.5 to 23.1)	62.4 (19.0 to 122)	1.6 (0.5 to 3.1)	12.2 (3.7 to 23.7)	2.45 (1.31 to 4.00)	0.1 (0.0 to 0.1)	9.1 (4.9 to 14.9)	65.2 (35.3 to 107)	1.5 (0.8 to 2.5)	9.0 (4.9 to 14.6)
2	High body-mass index	Non-Hodgkin lymphoma	8.16 (3.02 to 15.8)	0.2 (0.1 to 0.4)	5.6 (2.1 to 10.9)	221 (82.3 to 426)	5.6 (2.1 to 10.8)	5.2 (1.9 to 10.1)	5.64 (0.978 to 11.8)	0.1 (0.0 to 0.3)	5.2 (0.9 to 10.6)	135 (22.6 to 276)	3.1 (0.5 to 6.4)	4.9 (0.9 to 9.9)
2	High body-mass index	Multiple myeloma	3.92 (1.12 to 7.97)	0.1 (0.0 to 0.2)	6.5 (1.8 to 13.1)	91.6 (26.3 to 185)	2.3 (0.7 to 4.7)	6.7 (1.9 to 13.3)	4.09 (1.35 to 7.78)	0.1 (0.0 to 0.2)	7.7 (2.5 to 14.6)	88.2 (29.3 to 165)	2.0 (0.7 to 3.8)	7.9 (2.6 to 14.8)
2	High body-mass index	Leukaemia	9.24 (4.30 to 15.9)	0.3 (0.1 to 0.4)	4.9 (2.2 to 8.4)	255 (119 to 439)	6.5 (3.0 to 11.2)	3.8 (1.7 to 6.5)	12.5 (4.77 to 22.9)	0.3 (0.1 to 0.5)	8.6 (3.3 to 15.4)	330 (127 to 611)	7.7 (3.0 to 14.3)	6.6 (2.6 to 12.0)

“Total cancers” rows represent total risk-attributable cancer burden. Columns showing percentages for “Total cancers” rows were calculated as: (*Percentage of total risk-attributable cancer deaths or DALYs*) / (*total deaths or DALYs of all 29 cancer types*), specific to sex. Columns showing percentages for specific cancer type rows were calculated as: (*Percentage of risk-attributable deaths or DALYs due to specific cancer type*) / (*total deaths or DALYs* ***due to that cancer type*)**, specific to sex. Percentage of respective cancer deaths or DALYs for a specific cancer type indicates the percentage of attributable cancer deaths or DALYs out of the total deaths or DALYs of that cancer type. Number on the left of each risk factor indicates its level. DALYs = disability-adjusted life-years; ASR = age-standardised rate; GBD = Global Burden of Disease Study; NA = not applicable due to sex restriction; UI = uncertainty interval**.**

Appendix Table 14: Absolute and age-standardised deaths and DALYs attributable to risks assessed by cancer type in 2019, both sexes combined

GBD Risk Level	Risk factor	Cancer	Deaths			DALYs		
			Deaths, thousands (95% UI)	Age-standardised mortality rate, per 100,000 (95% UI)	Percentage of risk-attributable cancer deaths out of total cancer (risk + non-risk) deaths (95% UI)	DALYs, thousands (95% UI)	Age-standardised DALY rate, per 100,000 (95% UI)	Percentage of risk-attributable cancer DALYs out of total cancer (risk + non-risk) DALYs (95% UI)
**0**	**All risk factors**	**Total cancers**	**4 450 (4 010 to 4 940)**	**54.9 (49.3 to 61.0)**	**44.4 (41.3 to 48.4)**	**105 000 (95 000 to 116 000)**	**1 262.7 (1 142.8 to 1 398.7)**	**42.0 (39.1 to 45.6)**
**1**	**Environmental/occupational risks**	**Total cancers**	**737 (619 to 859)**	**9.1 (7.7 to 10.6)**	**7.3 (6.3 to 8.5)**	**16 300 (13 700 to 19 100)**	**196.1 (165.5 to 230.3)**	**6.5 (5.5 to 7.5)**
2	Air pollution	Total cancers	387 (288 to 490)	4.7 (3.5 to 6.0)	3.9 (2.9 to 4.8)	8 950 (6 680 to 11 300)	107.4 (80.1 to 136.0)	3.6 (2.7 to 4.4)
3	Particulate matter pollution	Total cancers	387 (288 to 490)	4.7 (3.5 to 6.0)	3.9 (2.9 to 4.8)	8 950 (6 680 to 11 300)	107.4 (80.1 to 136.0)	3.6 (2.7 to 4.4)
4	Ambient particulate matter pollution	Total cancers	308 (227 to 396)	3.8 (2.8 to 4.9)	3.1 (2.3 to 3.9)	7 020 (5 180 to 9 020)	84.2 (62.1 to 108.3)	2.8 (2.1 to 3.6)
4	Ambient particulate matter pollution	Tracheal, bronchus, and lung cancer	308 (227 to 396)	3.8 (2.8 to 4.9)	15.1 (11.3 to 18.9)	7 020 (5 180 to 9 020)	84.2 (62.1 to 108.3)	15.3 (11.5 to 19.1)
4	Household air pollution from solid fuels	Total cancers	79.8 (45.1 to 125)	1.0 (0.5 to 1.5)	0.8 (0.5 to 1.3)	1 940 (1 110 to 3 010)	23.1 (13.2 to 36.0)	0.8 (0.5 to 1.2)
4	Household air pollution from solid fuels	Tracheal, bronchus, and lung cancer	79.8 (45.1 to 125)	1.0 (0.5 to 1.5)	3.9 (2.2 to 6.1)	1 940 (1 110 to 3 010)	23.1 (13.2 to 36.0)	4.2 (2.4 to 6.5)
2	Other environmental risks	Total cancers	83.7 (16.5 to 162)	1.0 (0.2 to 2.0)	0.8 (0.2 to 1.6)	1 890 (374 to 3 650)	22.7 (4.5 to 43.9)	0.8 (0.2 to 1.5)
3	Residential radon	Total cancers	83.7 (16.5 to 162)	1.0 (0.2 to 2.0)	0.8 (0.2 to 1.6)	1 890 (374 to 3 650)	22.7 (4.5 to 43.9)	0.8 (0.2 to 1.5)
3	Residential radon	Tracheal, bronchus, and lung cancer	83.7 (16.5 to 162)	1.0 (0.2 to 2.0)	4.1 (0.8 to 7.9)	1 890 (374 to 3 650)	22.7 (4.5 to 43.9)	4.1 (0.8 to 8.0)
2	Occupational risks	Total cancers	334 (263 to 405)	4.2 (3.3 to 5.1)	3.3 (2.7 to 4.0)	6 960 (5 470 to 8 580)	84.4 (66.2 to 103.7)	2.8 (2.2 to 3.4)
3	Occupational carcinogens	Total cancers	334 (263 to 405)	4.2 (3.3 to 5.1)	3.3 (2.7 to 4.0)	6 960 (5 470 to 8 580)	84.4 (66.2 to 103.7)	2.8 (2.2 to 3.4)
4	Occupational exposure to asbestos	Total cancers	236 (176 to 296)	3.0 (2.2 to 3.8)	2.4 (1.8 to 3.0)	4 120 (3 060 to 5 240)	50.9 (37.8 to 64.7)	1.6 (1.2 to 2.1)
4	Occupational exposure to asbestos	Larynx cancer	3.68 (2.04 to 5.53)	0.0 (0.0 to 0.1)	3.0 (1.7 to 4.5)	70.0 (38.3 to 106)	0.9 (0.5 to 1.3)	2.1 (1.2 to 3.3)
4	Occupational exposure to asbestos	Tracheal, bronchus, and lung cancer	199 (140 to 257)	2.5 (1.8 to 3.3)	9.7 (6.9 to 12.5)	3 370 (2 340 to 4 450)	41.7 (29.0 to 55.0)	7.3 (5.1 to 9.7)
4	Occupational exposure to asbestos	Ovarian cancer	6.56 (2.95 to 10.7)	0.1 (0.0 to 0.1)	3.3 (1.5 to 5.4)	113 (50.1 to 185)	1.4 (0.6 to 2.3)	2.1 (1.0 to 3.4)
4	Occupational exposure to asbestos	Mesothelioma	26.8 (24.3 to 28.6)	0.3 (0.3 to 0.4)	91.7 (89.7 to 93.4)	569 (510 to 617)	6.9 (6.2 to 7.5)	85.2 (82.1 to 88.0)
4	Occupational exposure to arsenic	Total cancers	9.76 (1.55 to 17.7)	0.1 (0.0 to 0.2)	0.1 (0.0 to 0.2)	271 (44.8 to 486)	3.2 (0.5 to 5.7)	0.1 (0.0 to 0.2)
4	Occupational exposure to arsenic	Tracheal, bronchus, and lung cancer	9.76 (1.55 to 17.7)	0.1 (0.0 to 0.2)	0.5 (0.1 to 0.9)	271 (44.8 to 486)	3.2 (0.5 to 5.7)	0.6 (0.1 to 1.1)
4	Occupational exposure to benzene	Total cancers	1.87 (0.565 to 3.05)	0.0 (0.0 to 0.0)	0.0 (0.0 to 0.0)	85.8 (25.7 to 140)	1.1 (0.3 to 1.7)	0.0 (0.0 to 0.1)

GBD Risk Level	Risk factor	Cancer	Deaths			DALYs		
			Deaths, thousands (95% UI)	Age-standardised mortality rate, per 100,000 (95% UI)	Percentage of risk-attributable cancer deaths out of total cancer (risk + non-risk) deaths (95% UI)	DALYs, thousands (95% UI)	Age-standardised DALY rate, per 100,000 (95% UI)	Percentage of risk-attributable cancer DALYs out of total cancer (risk + non-risk) DALYs (95% UI)
4	Occupational exposure to benzene	Leukaemia	1.87 (0.565 to 3.05)	0.0 (0.0 to 0.0)	0.6 (0.2 to 0.9)	85.8 (25.7 to 140)	1.1 (0.3 to 1.7)	0.7 (0.2 to 1.2)
4	Occupational exposure to beryllium	Total cancers	0.301 (0.244 to 0.367)	0.0 (0.0 to 0.0)	0.0 (0.0 to 0.0)	8.58 (6.95 to 10.5)	0.1 (0.1 to 0.1)	0.0 (0.0 to 0.0)
4	Occupational exposure to beryllium	Tracheal, bronchus, and lung cancer	0.301 (0.244 to 0.367)	0.0 (0.0 to 0.0)	0.0 (0.0 to 0.0)	8.58 (6.95 to 10.5)	0.1 (0.1 to 0.1)	0.0 (0.0 to 0.0)
4	Occupational exposure to cadmium	Total cancers	0.712 (0.583 to 0.854)	0.0 (0.0 to 0.0)	0.0 (0.0 to 0.0)	20.3 (16.7 to 24.1)	0.2 (0.2 to 0.3)	0.0 (0.0 to 0.0)
4	Occupational exposure to cadmium	Tracheal, bronchus, and lung cancer	0.712 (0.583 to 0.854)	0.0 (0.0 to 0.0)	0.0 (0.0 to 0.0)	20.3 (16.7 to 24.1)	0.2 (0.2 to 0.3)	0.0 (0.0 to 0.1)
4	Occupational exposure to chromium	Total cancers	1.50 (1.29 to 1.75)	0.0 (0.0 to 0.0)	0.0 (0.0 to 0.0)	42.7 (36.6 to 49.7)	0.5 (0.4 to 0.6)	0.0 (0.0 to 0.0)
4	Occupational exposure to chromium	Tracheal, bronchus, and lung cancer	1.50 (1.29 to 1.75)	0.0 (0.0 to 0.0)	0.1 (0.1 to 0.1)	42.7 (36.6 to 49.7)	0.5 (0.4 to 0.6)	0.1 (0.1 to 0.1)
4	Occupational exposure to diesel engine exhaust	Total cancers	19.7 (17.0 to 22.9)	0.2 (0.2 to 0.3)	0.2 (0.2 to 0.2)	563 (485 to 655)	6.6 (5.7 to 7.7)	0.2 (0.2 to 0.3)
4	Occupational exposure to diesel engine exhaust	Tracheal, bronchus, and lung cancer	19.7 (17.0 to 22.9)	0.2 (0.2 to 0.3)	1.0 (0.8 to 1.1)	563 (485 to 655)	6.6 (5.7 to 7.7)	1.2 (1.1 to 1.4)
4	Occupational exposure to formaldehyde	Total cancers	1.12 (0.900 to 1.36)	0.0 (0.0 to 0.0)	0.0 (0.0 to 0.0)	50.8 (40.9 to 61.7)	0.6 (0.5 to 0.8)	0.0 (0.0 to 0.0)
4	Occupational exposure to formaldehyde	Nasopharynx cancer	0.518 (0.355 to 0.731)	0.0 (0.0 to 0.0)	0.7 (0.5 to 1.0)	22.3 (15.1 to 31.5)	0.3 (0.2 to 0.4)	1.0 (0.7 to 1.3)
4	Occupational exposure to formaldehyde	Leukaemia	0.600 (0.497 to 0.712)	0.0 (0.0 to 0.0)	0.2 (0.2 to 0.2)	28.5 (23.4 to 34.3)	0.4 (0.3 to 0.4)	0.2 (0.2 to 0.3)
4	Occupational exposure to nickel	Total cancers	9.33 (0.536 to 24.6)	0.1 (0.0 to 0.3)	0.1 (0.0 to 0.2)	261 (18.3 to 677)	3.1 (0.2 to 8.0)	0.1 (0.0 to 0.3)
4	Occupational exposure to nickel	Tracheal, bronchus, and lung cancer	9.33 (0.536 to 24.6)	0.1 (0.0 to 0.3)	0.5 (0.0 to 1.2)	261 (18.3 to 677)	3.1 (0.2 to 8.0)	0.6 (0.0 to 1.4)
4	Occupational exposure to polycyclic aromatic hydrocarbons	Total cancers	5.27 (4.36 to 6.24)	0.1 (0.1 to 0.1)	0.1 (0.0 to 0.1)	150 (123 to 177)	1.8 (1.5 to 2.1)	0.1 (0.1 to 0.1)
4	Occupational exposure to polycyclic aromatic hydrocarbons	Tracheal, bronchus, and lung cancer	5.27 (4.36 to 6.24)	0.1 (0.1 to 0.1)	0.3 (0.2 to 0.3)	150 (123 to 177)	1.8 (1.5 to 2.1)	0.3 (0.3 to 0.4)
4	Occupational exposure to silica	Total cancers	53.0 (23.8 to 84.4)	0.6 (0.3 to 1.0)	0.5 (0.2 to 0.8)	1 480 (666 to 2 350)	17.4 (7.8 to 27.7)	0.6 (0.3 to 0.9)
4	Occupational exposure to silica	Tracheal, bronchus, and lung cancer	53.0 (23.8 to 84.4)	0.6 (0.3 to 1.0)	2.6 (1.2 to 4.1)	1 480 (666 to 2 350)	17.4 (7.8 to 27.7)	3.2 (1.4 to 5.1)
4	Occupational exposure to sulfuric acid	Total cancers	4.03 (1.73 to 7.47)	0.0 (0.0 to 0.1)	0.0 (0.0 to 0.1)	126 (54.1 to 234)	1.5 (0.6 to 2.7)	0.1 (0.0 to 0.1)
4	Occupational exposure to sulfuric acid	Larynx cancer	4.03 (1.73 to 7.47)	0.0 (0.0 to 0.1)	3.3 (1.4 to 6.0)	126 (54.1 to 234)	1.5 (0.6 to 2.7)	3.9 (1.6 to 7.1)
4	Occupational exposure to trichloroethylene	Total cancers	0.0785 (0.0168 to 0.147)	0.0 (0.0 to 0.0)	0.0 (0.0 to 0.0)	2.43 (0.518 to 4.54)	0.0 (0.0 to 0.1)	0.0 (0.0 to 0.0)
4	Occupational exposure to trichloroethylene	Kidney cancer	0.0785 (0.0168 to 0.147)	0.0 (0.0 to 0.0)	0.0 (0.0 to 0.1)	2.43 (0.518 to 4.54)	0.0 (0.0 to 0.1)	0.1 (0.0 to 0.1)
1	**Behavioural risks**	**Total cancers**	**3 700 (3 420 to 4 020)**	**45.5 (42.1 to 49.4)**	**36.9 (35.3 to 38.9)**	**87 800 (81 100 to 95 400)**	**1 054.7 (974.1 to 1 145.3)**	**35.1 (33.6 to 36.9)**
2	Tobacco	Total cancers	2 600 (2 380 to 2 830)	31.9 (29.2 to 34.7)	25.9 (24.6 to 27.3)	59 300 (54 000 to 64 800)	711.7 (648.9 to 777.1)	23.7 (22.5 to 25.0)

GBD Risk Level	Risk factor	Cancer	Deaths			DALYs		
			**Deaths, thousands (95% UI)**	**Age-standardised mortality rate, per 100,000 (95% UI)**	**Percentage of risk-attributable cancer deaths out of total cancer (risk + non-risk) deaths (95% UI)**	**DALYs, thousands (95% UI)**	**Age-standardised DALY rate, per 100,000 (95% UI)**	**Percentage of risk-attributable cancer DALYs out of total cancer (risk + non-risk) DALYs (95% UI)**
3	Smoking	Total cancers	2 490 (2 280 to 2 720)	30.6 (28.0 to 33.3)	24.9 (23.6 to 26.2)	56 400 (51 300 to 61 700)	677.3 (616.4 to 740.3)	22.6 (21.3 to 23.9)
3	Smoking	Lip and oral cavity cancer	63.4 (51.2 to 76.4)	0.8 (0.6 to 0.9)	31.8 (26.4 to 36.9)	1 660 (1 310 to 2 020)	19.7 (15.6 to 23.9)	30.1 (24.1 to 35.4)
3	Smoking	Nasopharynx cancer	17.9 (13.0 to 23.0)	0.2 (0.2 to 0.3)	25.0 (18.1 to 31.6)	527 (374 to 684)	6.2 (4.4 to 8.1)	22.5 (15.9 to 28.8)
3	Smoking	Other pharynx cancer	53.6 (45.2 to 61.9)	0.6 (0.5 to 0.7)	47.0 (40.6 to 53.0)	1 440 (1 200 to 1 680)	17.1 (14.2 to 19.9)	44.6 (38.3 to 50.9)
3	Smoking	Oesophageal cancer	203 (170 to 237)	2.5 (2.1 to 2.9)	40.8 (37.0 to 44.5)	4 750 (3 980 to 5 540)	56.7 (47.6 to 66.1)	40.7 (36.6 to 44.5)
3	Smoking	Stomach cancer	172 (138 to 207)	2.1 (1.7 to 2.5)	18.0 (14.7 to 21.1)	3 810 (2 990 to 4 630)	45.8 (36.1 to 55.6)	17.2 (13.8 to 20.4)
3	Smoking	Colon and rectum cancer	143 (95.5 to 193)	1.8 (1.2 to 2.4)	13.2 (8.8 to 17.8)	3 230 (2 090 to 4 400)	38.9 (25.3 to 53.0)	13.3 (8.6 to 17.8)
3	Smoking	Liver cancer	85.9 (50.0 to 123)	1.0 (0.6 to 1.5)	17.7 (10.2 to 24.8)	2 130 (1 160 to 3 070)	25.3 (13.8 to 36.5)	17.0 (9.3 to 24.2)
3	Smoking	Pancreatic cancer	113 (98.8 to 128)	1.4 (1.2 to 1.6)	21.4 (18.9 to 23.8)	2 440 (2 110 to 2 770)	29.4 (25.4 to 33.4)	21.2 (18.6 to 23.6)
3	Smoking	Larynx cancer	78.3 (68.0 to 88.3)	0.9 (0.8 to 1.1)	63.4 (56.3 to 69.3)	2 020 (1 760 to 2 300)	24.0 (20.9 to 27.3)	62.0 (54.7 to 67.9)
3	Smoking	Tracheal, bronchus, and lung cancer	1 310 (1 200 to 1 430)	16.1 (14.7 to 17.5)	64.2 (61.9 to 66.4)	28 600 (26 000 to 31 300)	344.0 (313.2 to 375.3)	62.4 (60.1 to 64.7)
3	Smoking	Breast cancer	19.0 (13.6 to 24.8)	0.2 (0.2 to 0.3)	2.7 (1.9 to 3.5)	513 (362 to 674)	6.1 (4.3 to 8.1)	2.5 (1.7 to 3.3)
3	Smoking	Cervical cancer	30.1 (14.9 to 49.6)	0.4 (0.2 to 0.6)	10.8 (5.4 to 17.4)	894 (469 to 1 440)	10.6 (5.6 to 17.1)	10.0 (5.2 to 15.8)
3	Smoking	Prostate cancer	29.3 (12.8 to 46.6)	0.4 (0.2 to 0.6)	6.0 (2.7 to 9.3)	572 (253 to 918)	7.0 (3.1 to 11.3)	6.6 (3.0 to 10.1)
3	Smoking	Kidney cancer	30.1 (21.0 to 39.4)	0.4 (0.3 to 0.5)	18.1 (12.8 to 23.4)	687 (476 to 883)	8.3 (5.7 to 10.7)	17.0 (11.8 to 21.8)
3	Smoking	Bladder cancer	77.5 (58.3 to 96.7)	1.0 (0.7 to 1.2)	33.9 (25.9 to 41.8)	1 620 (1 250 to 1 980)	19.7 (15.2 to 24.1)	36.8 (28.5 to 44.0)
3	Smoking	Leukaemia	64.6 (39.3 to 91.4)	0.8 (0.5 to 1.1)	19.3 (12.0 to 27.2)	1 530 (896 to 2 160)	18.5 (10.8 to 26.0)	13.1 (7.8 to 18.7)
3	Chewing tobacco	Total cancers	55.6 (43.1 to 68.8)	0.7 (0.5 to 0.8)	0.6 (0.4 to 0.7)	1 500 (1 160 to 1 880)	17.9 (13.9 to 22.4)	0.6 (0.5 to 0.7)
3	Chewing tobacco	Lip and oral cavity cancer	37.3 (27.9 to 47.2)	0.5 (0.3 to 0.6)	18.7 (14.5 to 23.0)	1 030 (764 to 1 320)	12.3 (9.1 to 15.7)	18.7 (14.3 to 23.0)
3	Chewing tobacco	Oesophageal cancer	18.3 (12.7 to 24.7)	0.2 (0.2 to 0.3)	3.7 (2.6 to 5.0)	476 (328 to 644)	5.7 (3.9 to 7.7)	4.1 (2.8 to 5.5)
3	Secondhand smoke	Total cancers	130 (82.6 to 190)	1.6 (1.0 to 2.3)	1.3 (0.8 to 1.9)	3 220 (2 070 to 4 630)	38.5 (24.8 to 55.5)	1.3 (0.8 to 1.8)
3	Secondhand smoke	Tracheal, bronchus, and lung cancer	113 (67.5 to 170)	1.4 (0.8 to 2.1)	5.6 (3.3 to 8.1)	2 660 (1 580 to 3 990)	31.8 (19.0 to 47.8)	5.8 (3.5 to 8.5)
3	Secondhand smoke	Breast cancer	17.0 (4.00 to 29.4)	0.2 (0.0 to 0.4)	2.4 (0.6 to 4.2)	562 (135 to 976)	6.7 (1.6 to 11.6)	2.7 (0.7 to 4.7)
2	Alcohol use	Total cancers	495 (440 to 554)	6.0 (5.4 to 6.8)	4.9 (4.4 to 5.5)	13 000 (11 600 to 14 500)	155.2 (138.4 to 173.5)	5.2 (4.7 to 5.7)

GBD Risk Level	Risk factor	Cancer	Deaths			DALYs		
			Deaths, thousands (95% UI)	Age-standardised mortality rate, per 100,000 (95% UI)	Percentage of risk-attributable cancer deaths out of total cancer (risk + non-risk) deaths (95% UI)	DALYs, thousands (95% UI)	Age-standardised DALY rate, per 100,000 (95% UI)	Percentage of risk-attributable cancer DALYs out of total cancer (risk + non-risk) DALYs (95% UI)
2	Alcohol use	Lip and oral cavity cancer	60.4 (48.0 to 72.4)	0.7 (0.6 to 0.9)	30.3 (24.7 to 35.4)	1 730 (1 380 to 2 070)	20.6 (16.4 to 24.6)	31.4 (25.6 to 36.6)
2	Alcohol use	Nasopharynx cancer	24.5 (18.8 to 29.9)	0.3 (0.2 to 0.4)	34.2 (27.0 to 41.0)	811 (628 to 993)	9.6 (7.5 to 11.8)	34.7 (27.5 to 41.4)
2	Alcohol use	Other pharynx cancer	37.9 (29.0 to 47.0)	0.5 (0.3 to 0.6)	33.2 (26.0 to 39.9)	1 110 (854 to 1 370)	13.1 (10.1 to 16.2)	34.2 (26.9 to 41.0)
2	Alcohol use	Oesophageal cancer	114 (84.1 to 145)	1.4 (1.0 to 1.8)	22.8 (17.4 to 28.2)	2 820 (2 110 to 3 570)	33.6 (25.1 to 42.5)	24.1 (18.4 to 29.7)
2	Alcohol use	Colon and rectum cancer	101 (76.6 to 127)	1.3 (1.0 to 1.6)	9.3 (7.1 to 11.5)	2 410 (1 830 to 3 000)	29.1 (22.1 to 36.2)	9.9 (7.5 to 12.3)
2	Alcohol use	Liver cancer	96.1 (77.5 to 116)	1.2 (0.9 to 1.4)	19.8 (16.2 to 23.6)	2 380 (1 910 to 2 890)	28.4 (22.9 to 34.5)	19.0 (15.3 to 22.8)
2	Alcohol use	Larynx cancer	23.9 (14.1 to 32.6)	0.3 (0.2 to 0.4)	19.4 (11.6 to 26.3)	656 (395 to 895)	7.8 (4.7 to 10.6)	20.1 (12.2 to 27.2)
2	Alcohol use	Breast cancer	37.7 (30.7 to 45.1)	0.5 (0.4 to 0.6)	5.4 (4.4 to 6.4)	1 090 (880 to 1 300)	13.1 (10.6 to 15.6)	5.3 (4.3 to 6.3)
2	Drug use	Total cancers	71.5 (57.1 to 89.2)	0.9 (0.7 to 1.1)	0.7 (0.6 to 0.9)	1 610 (1 290 to 1 990)	19.4 (15.6 to 23.9)	0.6 (0.5 to 0.8)
2	Drug use	Liver cancer	71.5 (57.1 to 89.2)	0.9 (0.7 to 1.1)	14.7 (11.9 to 18.0)	1 610 (1 290 to 1 990)	19.4 (15.6 to 23.9)	12.9 (10.4 to 15.7)
2	Dietary risks	Total cancers	605 (454 to 811)	7.6 (5.7 to 10.1)	6.0 (4.6 to 8.2)	14 000 (10 500 to 18 800)	168.8 (127.1 to 226.9)	5.6 (4.2 to 7.6)
3	Diet low in fruits	Total cancers	128 (65.0 to 200)	1.6 (0.8 to 2.5)	1.3 (0.6 to 2.0)	3 000 (1 540 to 4 680)	36.0 (18.5 to 56.2)	1.2 (0.6 to 1.8)
3	Diet low in fruits	Oesophageal cancer	51.2 (15.2 to 109)	0.6 (0.2 to 1.3)	10.3 (3.1 to 22.2)	1 250 (384 to 2 600)	15.0 (4.6 to 31.1)	10.7 (3.4 to 22.6)
3	Diet low in fruits	Tracheal, bronchus, and lung cancer	77.2 (22.6 to 115)	1.0 (0.3 to 1.4)	3.8 (1.1 to 5.6)	1 750 (518 to 2 610)	21.0 (6.2 to 31.4)	3.8 (1.1 to 5.7)
3	Diet low in vegetables	Total cancers	17.2 (2.55 to 34.0)	0.2 (0.0 to 0.4)	0.2 (0.0 to 0.3)	420 (64.2 to 828)	5.0 (0.8 to 9.9)	0.2 (0.0 to 0.3)
3	Diet low in vegetables	Oesophageal cancer	17.2 (2.55 to 34.0)	0.2 (0.0 to 0.4)	3.5 (0.5 to 6.9)	420 (64.2 to 828)	5.0 (0.8 to 9.9)	3.6 (0.6 to 7.1)
3	Diet low in whole grains	Total cancers	171 (66.7 to 225)	2.2 (0.8 to 2.8)	1.7 (0.7 to 2.2)	3 810 (1 460 to 5 020)	46.3 (17.8 to 61.1)	1.5 (0.6 to 2.0)
3	Diet low in whole grains	Colon and rectum cancer	171 (66.7 to 225)	2.2 (0.8 to 2.8)	15.8 (6.1 to 20.7)	3 810 (1 460 to 5 020)	46.3 (17.8 to 61.1)	15.7 (6.0 to 20.6)
3	Diet low in milk	Total cancers	166 (107 to 226)	2.1 (1.3 to 2.8)	1.7 (1.1 to 2.2)	3 800 (2 460 to 5 120)	46.1 (29.8 to 62.2)	1.5 (1.0 to 2.1)
3	Diet low in milk	Colon and rectum cancer	166 (107 to 226)	2.1 (1.3 to 2.8)	15.3 (10.0 to 20.7)	3 800 (2 460 to 5 120)	46.1 (29.8 to 62.2)	15.6 (10.3 to 21.0)
3	Diet high in red meat	Total cancers	75.3 (35.9 to 126)	0.9 (0.4 to 1.6)	0.8 (0.4 to 1.3)	1 890 (964 to 3 000)	22.8 (11.6 to 36.4)	0.8 (0.4 to 1.2)
3	Diet high in red meat	Colon and rectum cancer	52.8 (13.6 to 101)	0.7 (0.2 to 1.3)	4.9 (1.2 to 9.4)	1 230 (333 to 2 310)	14.9 (4.0 to 28.0)	5.1 (1.3 to 9.5)
3	Diet high in red meat	Breast cancer	22.5 (10.6 to 30.1)	0.3 (0.1 to 0.4)	3.2 (1.5 to 4.2)	651 (312 to 870)	7.8 (3.7 to 10.4)	3.2 (1.5 to 4.2)
3	Diet high in processed meat	Total cancers	33.9 (11.6 to 52.1)	0.4 (0.1 to 0.7)	0.3 (0.1 to 0.5)	735 (263 to 1 130)	9.0 (3.2 to 13.7)	0.3 (0.1 to 0.5)

GBD Risk Level	Risk factor	Cancer	Deaths			DALYs		
			Deaths, thousands (95% UI)	Age-standardised mortality rate, per 100,000 (95% UI)	Percentage of risk-attributable cancer deaths out of total cancer (risk + non-risk) deaths (95% UI)	DALYs, thousands (95% UI)	Age-standardised DALY rate, per 100,000 (95% UI)	Percentage of risk-attributable cancer DALYs out of total cancer (risk + non-risk) DALYs (95% UI)
3	Diet high in processed meat	Colon and rectum cancer	33.9 (11.6 to 52.1)	0.4 (0.1 to 0.7)	3.1 (1.1 to 4.8)	735 (263 to 1 130)	9.0 (3.2 to 13.7)	3.0 (1.1 to 4.6)
3	Diet low in fibre	Total cancers	20.5 (8.21 to 39.8)	0.3 (0.1 to 0.5)	0.2 (0.1 to 0.4)	449 (178 to 858)	5.5 (2.2 to 10.5)	0.2 (0.1 to 0.3)
3	Diet low in fibre	Colon and rectum cancer	20.5 (8.21 to 39.8)	0.3 (0.1 to 0.5)	1.9 (0.7 to 3.6)	449 (178 to 858)	5.5 (2.2 to 10.5)	1.8 (0.7 to 3.5)
3	Diet low in calcium	Total cancers	138 (96.8 to 189)	1.7 (1.2 to 2.4)	1.4 (1.0 to 1.9)	3 140 (2 250 to 4 260)	38.2 (27.2 to 51.8)	1.3 (0.9 to 1.7)
3	Diet low in calcium	Colon and rectum cancer	138 (96.8 to 189)	1.7 (1.2 to 2.4)	12.7 (9.1 to 17.3)	3 140 (2 250 to 4 260)	38.2 (27.2 to 51.8)	12.9 (9.3 to 17.5)
3	Diet high in sodium	Total cancers	74.1 (2.12 to 295)	0.9 (0.0 to 3.6)	0.7 (0.0 to 2.9)	1 740 (48.7 to 6 800)	20.9 (0.6 to 82.1)	0.7 (0.0 to 2.7)
3	Diet high in sodium	Stomach cancer	74.1 (2.12 to 295)	0.9 (0.0 to 3.6)	7.7 (0.2 to 30.9)	1 740 (48.7 to 6 800)	20.9 (0.6 to 82.1)	7.8 (0.2 to 30.9)
2	Unsafe sex	Total cancers	280 (239 to 314)	3.4 (2.9 to 3.8)	2.8 (2.4 to 3.1)	8 960 (7 550 to 9 980)	107.2 (90.5 to 119.4)	3.6 (3.0 to 3.9)
2	Unsafe sex	Cervical cancer	280 (239 to 314)	3.4 (2.9 to 3.8)	100.0 (100.0 to 100.0)	8 960 (7 550 to 9 980)	107.2 (90.5 to 119.4)	100.0 (100.0 to 100.0)
2	Low physical activity	Total cancers	67.1 (25.8 to 122)	0.9 (0.3 to 1.6)	0.7 (0.3 to 1.2)	1 200 (455 to 2 160)	15.0 (5.7 to 26.9)	0.5 (0.2 to 0.9)
2	Low physical activity	Colon and rectum cancer	58.7 (16.9 to 112)	0.8 (0.2 to 1.5)	5.4 (1.5 to 10.4)	1 000 (262 to 1 940)	12.6 (3.4 to 24.2)	4.1 (1.1 to 8.1)
2	Low physical activity	Breast cancer	8.48 (4.08 to 14.3)	0.1 (0.1 to 0.2)	1.2 (0.6 to 2.1)	198 (97.5 to 345)	2.4 (1.2 to 4.2)	1.0 (0.5 to 1.7)
**1**	**Metabolic risks**	**Total cancers**	**865 (448 to 1 410)**	**10.7 (5.5 to 17.5)**	**8.6 (4.5 to 13.9)**	**19 400 (10 300 to 31 100)**	**234.0 (124.0 to 376.0)**	**7.8 (4.1 to 12.4)**
2	High fasting plasma glucose	Total cancers	419 (116 to 848)	5.3 (1.5 to 10.6)	4.2 (1.1 to 8.4)	8 580 (2 360 to 17 600)	104.2 (28.7 to 212.9)	3.4 (0.9 to 7.0)
2	High fasting plasma glucose	Colon and rectum cancer	97.6 (23.8 to 213)	1.2 (0.3 to 2.7)	9.0 (2.2 to 19.2)	1 900 (454 to 4 170)	23.3 (5.6 to 51.2)	7.8 (1.9 to 16.9)
2	High fasting plasma glucose	Liver cancer	4.73 (1.15 to 10.4)	0.1 (0.0 to 0.1)	1.0 (0.2 to 2.1)	99.3 (23.9 to 218)	1.2 (0.3 to 2.6)	0.8 (0.2 to 1.7)
2	High fasting plasma glucose	Pancreatic cancer	48.4 (11.5 to 104)	0.6 (0.1 to 1.3)	9.1 (2.2 to 19.3)	944 (221 to 2 040)	11.5 (2.7 to 24.7)	8.2 (1.9 to 17.6)
2	High fasting plasma glucose	Tracheal, bronchus, and lung cancer	179 (42.7 to 389)	2.2 (0.5 to 4.8)	8.8 (2.0 to 19.1)	3 640 (856 to 8 010)	44.1 (10.4 to 96.8)	7.9 (1.8 to 17.3)
2	High fasting plasma glucose	Breast cancer	51.1 (9.90 to 114)	0.6 (0.1 to 1.4)	7.3 (1.4 to 16.0)	1 240 (238 to 2 790)	14.9 (2.9 to 33.5)	6.0 (1.1 to 13.4)
2	High fasting plasma glucose	Ovarian cancer	15.7 (3.02 to 36.2)	0.2 (0.0 to 0.4)	7.9 (1.6 to 18.3)	354 (68.5 to 824)	4.3 (0.8 to 9.9)	6.6 (1.3 to 15.3)
2	High fasting plasma glucose	Bladder cancer	22.8 (4.69 to 49.0)	0.3 (0.1 to 0.6)	10.0 (2.1 to 21.3)	400 (81.6 to 866)	5.0 (1.0 to 10.7)	9.1 (1.9 to 19.6)
2	High body-mass index	Total cancers	463 (261 to 718)	5.7 (3.2 to 8.8)	4.6 (2.7 to 7.1)	11 200 (6 360 to 17 300)	133.9 (76.2 to 206.8)	4.5 (2.6 to 6.9)
2	High body-mass index	Oesophageal cancer	89.9 (27.9 to 171)	1.1 (0.3 to 2.1)	18.1 (5.8 to 35.2)	2 200 (682 to 4 170)	26.3 (8.1 to 49.9)	18.9 (6.0 to 36.0)
2	High body-mass index	Colon and rectum cancer	85.9 (46.8 to 137)	1.1 (0.6 to 1.7)	7.9 (4.3 to 12.4)	2 020 (1 120 to 3 180)	24.4 (13.5 to 38.5)	8.3 (4.6 to 13.0)

GBD Risk Level	Risk factor	Cancer	Deaths			DALYs		
			**Deaths, thousands (95% UI)**	**Age-standardised mortality rate, per 100,000 (95% UI)**	**Percentage of risk-attributable cancer deaths out of total cancer (risk + non-risk) deaths (95% UI)**	**DALYs, thousands (95% UI)**	**Age-standardised DALY rate, per 100,000 (95% UI)**	**Percentage of risk-attributable cancer DALYs out of total cancer (risk + non-risk) DALYs (95% UI)**
2	High body-mass index	Liver cancer	60.8 (24.2 to 115)	0.7 (0.3 to 1.4)	12.5 (5.0 to 24.1)	1 610 (629 to 3 050)	19.2 (7.6 to 36.4)	12.9 (5.1 to 24.8)
2	High body-mass index	Gallbladder and biliary tract cancer	26.1 (13.9 to 42.6)	0.3 (0.2 to 0.5)	15.2 (8.0 to 24.7)	568 (306 to 923)	6.9 (3.7 to 11.2)	15.7 (8.4 to 25.2)
2	High body-mass index	Pancreatic cancer	31.9 (12.0 to 59.7)	0.4 (0.1 to 0.7)	6.0 (2.2 to 11.4)	709 (256 to 1 330)	8.5 (3.1 to 16.0)	6.1 (2.2 to 11.7)
2	High body-mass index	Breast cancer	45.2 (18.8 to 81.2)	0.6 (0.2 to 1.0)	6.4 (2.6 to 11.8)	958 (306 to 1 820)	11.2 (3.5 to 21.4)	4.6 (1.5 to 8.9)
2	High body-mass index	Uterine cancer	36.5 (25.1 to 49.2)	0.4 (0.3 to 0.6)	39.8 (27.6 to 52.7)	936 (643 to 1 260)	11.2 (7.7 to 15.0)	40.2 (28.0 to 53.1)
2	High body-mass index	Ovarian cancer	6.31 (-0.177 to 14.3)	0.1 (0.0 to 0.2)	3.2 (-0.1 to 7.1)	168 (-4.67 to 380)	2.0 (-0.1 to 4.5)	3.1 (-0.1 to 7.0)
2	High body-mass index	Kidney cancer	31.7 (18.4 to 47.3)	0.4 (0.2 to 0.6)	19.0 (11.1 to 28.3)	752 (444 to 1 110)	9.0 (5.3 to 13.4)	18.6 (10.9 to 27.4)
2	High body-mass index	Thyroid cancer	4.66 (2.29 to 7.89)	0.1 (0.0 to 0.1)	10.2 (5.0 to 17.0)	128 (63.8 to 214)	1.5 (0.8 to 2.6)	10.4 (5.1 to 17.0)
2	High body-mass index	Non-Hodgkin lymphoma	13.8 (5.81 to 24.5)	0.2 (0.1 to 0.3)	5.4 (2.3 to 9.6)	356 (152 to 633)	4.3 (1.8 to 7.7)	5.1 (2.2 to 9.0)
2	High body-mass index	Multiple myeloma	8.02 (3.52 to 14.2)	0.1 (0.0 to 0.2)	7.1 (3.1 to 12.4)	180 (80.3 to 318)	2.2 (1.0 to 3.8)	7.2 (3.2 to 12.5)
2	High body-mass index	Leukaemia	21.7 (10.5 to 37.0)	0.3 (0.1 to 0.5)	6.5 (3.2 to 10.9)	584 (288 to 993)	7.1 (3.5 to 12.1)	5.0 (2.4 to 8.4)

Columns showing percentages for “Total cancers” rows were calculated as: (*Percentage of total risk-attributable cancer deaths or DALYs*) / (*total deaths or DALYs of all 29 cancer types*), both sexes combined. Columns showing percentages for specific cancer type rows were calculated as: (*Percentage of risk-attributable deaths or DALYs due to specific cancer type*) / (*total deaths or DALYs due to that cancer type*), both sexes combined. UI = uncertainty interval; DALY = disability-adjusted life-year; GBD = Global Burden of Disease Study.

Appendix Table 15: Global deaths attributable vs. not attributable to risks assessed for each cancer type by sex in 2019

	Global								
	Males			Females			Male:Female Ratio		
**Cancer**	**Deaths attributable to risks assessed (95% UI)**	**Deaths not attributable to risks assessed (95% UI)**	**Total deaths (95% UI)**	**Deaths attributable to risks assessed (95% UI)**	**Deaths not attributable to risks assessed (95% UI)**	**Total deaths (95% UI)**	**Deaths attributable to risks assessed (95% UI)**	**Deaths not attributable to risks assessed (95% UI)**	**Total deaths (95% UI)**
Bladder cancer	79 500 (60 100 to 100 000)	89 700 (69 900 to 110 000)	169 000 (157 000 to 181 000)	13 200 (8 040 to 19 800)	46 300 (38 400 to 53 500)	59 500 (52 300 to 64 600)	6.23 (4.50 to 8.23)	1.94 (1.64 to 2.24)	2.85 (2.64 to 3.09)
Breast cancer	1 870 (1 490 to 2 270)	10 200 (9 000 to 11 400)	12 100 (10 700 to 13 300)	175 000 (124 000 to 238 000)	514 000 (440 000 to 582 000)	689 000 (635 000 to 740 000)	0.01 (0.01 to 0.01)	0.02 (0.02 to 0.02)	0.02 (0.02 to 0.02)
Cervical cancer	NA	NA	NA	280 000 (239 000 to 314 000)	0 (0 to 0)	280 000 (239 000 to 314 000)	NA	NA	NA
Colon and rectum cancer	383 000 (333 000 to 435 000)	211 000 (167 000 to 255 000)	594 000 (551 000 to 638 000)	249 000 (204 000 to 296 000)	243 000 (199 000 to 288 000)	492 000 (438 000 to 532 000)	1.54 (1.38 to 1.74)	0.87 (0.75 to 1.00)	1.21 (1.11 to 1.33)
Gallbladder and biliary tract cancer	5 830 (1 060 to 12 300)	67 200 (53 100 to 76 000)	73 000 (59 500 to 80 400)	20 300 (10 800 to 32 900)	79 200 (61 900 to 93 700)	99 500 (81 700 to 114 000)	0.29 (0.06 to 0.57)	0.86 (0.69 to 1.05)	0.74 (0.60 to 0.84)
Kidney cancer	38 700 (29 000 to 48 900)	70 100 (59 000 to 80 700)	109 000 (101 000 to 116 000)	17 600 (12 500 to 24 000)	40 100 (33 400 to 46 200)	57 700 (52 200 to 61 900)	2.22 (1.74 to 2.76)	1.76 (1.56 to 2.02)	1.89 (1.76 to 2.03)
Larynx cancer	81 600 (72 700 to 90 600)	24 000 (18 600 to 30 400)	106 000 (97 800 to 115 000)	5 890 (4 800 to 7 060)	11 900 (10 500 to 13 600)	17 800 (16 200 to 19 700)	13.92 (11.95 to 15.98)	2.01 (1.62 to 2.47)	5.94 (5.28 to 6.63)
Leukaemia	57 500 (38 800 to 76 800)	131 000 (107 000 to 157 000)	188 000 (165 000 to 208 000)	26 100 (16 600 to 37 900)	120 000 (104 000 to 135 000)	146 000 (132 000 to 158 000)	2.24 (1.61 to 2.95)	1.09 (0.92 to 1.29)	1.29 (1.12 to 1.47)
Lip and oral cavity cancer	93 600 (80 800 to 106 000)	38 000 (30 500 to 46 700)	132 000 (118 000 to 145 000)	30 400 (25 300 to 36 000)	37 400 (32 400 to 42 900)	67 800 (60 800 to 75 700)	3.09 (2.56 to 3.75)	1.02 (0.83 to 1.27)	1.94 (1.71 to 2.20)
Liver cancer	190 000 (160 000 to 223 000)	143 000 (114 000 to 178 000)	334 000 (300 000 to 368 000)	64 300 (52 100 to 77 900)	86 600 (72 400 to 102 000)	151 000 (134 000 to 167 000)	2.98 (2.43 to 3.63)	1.66 (1.30 to 2.05)	2.22 (1.92 to 2.54)
Mesothelioma	19 800 (18 400 to 21 200)	1 440 (1 010 to 1 930)	21 200 (20 000 to 22 500)	7 030 (4 930 to 7 970)	994 (745 to 1 290)	8 030 (5 880 to 8 920)	2.85 (2.49 to 4.07)	1.48 (0.91 to 2.21)	2.67 (2.37 to 3.67)
Multiple myeloma	3 920 (1 120 to 7 970)	56 500 (46 800 to 62 800)	60 400 (50 700 to 67 100)	4 090 (1 350 to 7 780)	48 900 (41 100 to 55 000)	53 000 (45 100 to 58 300)	1.05 (0.27 to 2.77)	1.16 (0.96 to 1.38)	1.14 (0.97 to 1.34)

	Global								
	Males			Females			Male:Female Ratio		
**Cancer**	**Deaths attributable to risks assessed (95% UI)**	**Deaths not attributable to risks assessed (95% UI)**	**Total deaths (95% UI)**	**Deaths attributable to risks assessed (95% UI)**	**Deaths not attributable to risks assessed (95% UI)**	**Total deaths (95% UI)**	**Deaths attributable to risks assessed (95% UI)**	**Deaths not attributable to risks assessed (95% UI)**	**Total deaths (95% UI)**
Nasopharynx cancer	31 600 (26 400 to 36 900)	19 700 (15 700 to 24 400)	51 200 (46 000 to 57 000)	3 360 (2 480 to 4 270)	17 000 (15 000 to 19 300)	20 400 (18 200 to 22 800)	9.51 (7.71 to 11.82)	1.16 (0.93 to 1.43)	2.52 (2.15 to 2.91)
Non-Hodgkin lymphoma	8 160 (3 020 to 15 800)	137 000 (126 000 to 149 000)	146 000 (136 000 to 155 000)	5 640 (978 to 11 800)	103 000 (92 000 to 113 000)	109 000 (98 900 to 117 000)	1.56 (0.43 to 5.35)	1.33 (1.21 to 1.48)	1.34 (1.24 to 1.45)
Oesophageal cancer	279 000 (234 000 to 322 000)	86 400 (60 500 to 113 000)	366 000 (315 000 to 415 000)	60 400 (42 200 to 82 100)	72 100 (48 800 to 93 100)	133 000 (110 000 to 150 000)	4.74 (3.33 to 6.57)	1.22 (0.85 to 1.77)	2.77 (2.31 to 3.31)
Other pharynx cancer	64 300 (55 900 to 72 900)	23 700 (18 500 to 30 000)	88 000 (78 000 to 98 700)	6 360 (5 210 to 7 610)	19 800 (16 600 to 23 500)	26 200 (22 500 to 30 500)	10.18 (8.45 to 12.07)	1.20 (0.90 to 1.55)	3.38 (2.79 to 4.13)
Ovarian cancer	NA	NA	NA	27 200 (11 700 to 48 100)	171 000 (142 000 to 196 000)	198 000 (175 000 to 218 000)	NA	NA	NA
Pancreatic cancer	101 000 (78 400 to 132 000)	177 000 (145 000 to 206 000)	278 000 (258 000 to 299 000)	72 700 (50 800 to 98 900)	180 000 (149 000 to 209 000)	253 000 (226 000 to 274 000)	1.41 (1.08 to 1.81)	0.99 (0.86 to 1.13)	1.10 (1.01 to 1.22)
Prostate cancer	29 300 (12 800 to 46 600)	458 000 (393 000 to 560 000)	487 000 (420 000 to 594 000)	NA	NA	NA	NA	NA	NA
Stomach cancer	192 000 (133 000 to 302 000)	420 000 (306 000 to 503 000)	612 000 (544 000 to 678 000)	40 500 (14 800 to 114 000)	305 000 (231 000 to 354 000)	346 000 (308 000 to 382 000)	6.24 (2.49 to 10.06)	1.38 (1.18 to 1.58)	1.77 (1.53 to 2.03)
Thyroid cancer	2 210 (639 to 4 320)	16 400 (13 700 to 18 800)	18 600 (16 800 to 20 200)	2 450 (1 310 to 4 000)	24 500 (21 100 to 27 100)	26 900 (23 700 to 29 300)	0.92 (0.31 to 1.66)	0.67 (0.56 to 0.78)	0.69 (0.62 to 0.78)
Tracheal, bronchus, and lung cancer	1 210 000 (1 100 000 to 1 330 000)	173 000 (142 000 to 204 000)	1 390 000 (1 260 000 to 1 510 000)	427 000 (376 000 to 481 000)	229 000 (190 000 to 271 000)	657 000 (590 000 to 719 000)	2.85 (2.48 to 3.24)	0.76 (0.65 to 0.88)	2.12 (1.87 to 2.37)
Uterine cancer	NA	NA	NA	36 500 (25 100 to 49 200)	55 200 (42 500 to 68 300)	91 600 (82 400 to 102 000)	NA	NA	NA

NA = not applicable due to sex restriction; UI = uncertainty interval.

Appendix Table 16: Global age-standardised death rates attributable vs. not attributable to risks assessed for each cancer type by sex in 2019

	Global								
	Male			Female			Male:Female Ratio		
Cancer	Age-standardised mortality rate attributable to risks assessed (95% UI)	Age-standardised mortality rate not attributable to risks assessed (95% UI)	Total age-standardised mortality rates (95% UI)	Age-standardised mortality rate attributable to risks assessed (95% UI)	Age-standardised mortality rate not attributable to risks assessed (95% UI)	Total age-standardised mortality rates (95% UI)	Age-standardised mortality rate attributable to risks assessed (95% UI)	Age-standardised mortality rate not attributable to risks assessed (95% UI)	Total age-standardised mortality rates (95% UI)
Bladder cancer	2.3 (1.7 to 3.0)	2.8 (2.2 to 3.4)	5.1 (4.7 to 5.4)	0.3 (0.2 to 0.5)	1.1 (0.9 to 1.2)	1.4 (1.2 to 1.5)	7.97 (5.81 to 10.40)	2.63 (2.22 to 3.03)	3.76 (3.50 to 4.04)
Breast cancer	0.0 (0.0 to 0.1)	0.3 (0.2 to 0.3)	0.3 (0.3 to 0.4)	4.0 (2.8 to 5.4)	11.9 (10.2 to 13.5)	15.9 (14.7 to 17.1)	0.01 (0.01 to 0.02)	0.02 (0.02 to 0.03)	0.02 (0.02 to 0.02)
Cervical cancer	NA	NA	NA	6.5 (5.5 to 7.3)	0.0 (0.0 to 0.0)	6.5 (5.5 to 7.3)	NA	NA	NA
Colon and rectum cancer	10.7 (9.3 to 12.2)	5.9 (4.7 to 7.2)	16.6 (15.4 to 17.9)	5.7 (4.7 to 6.7)	5.6 (4.6 to 6.6)	11.2 (10.0 to 12.2)	1.89 (1.70 to 2.13)	1.07 (0.93 to 1.22)	1.48 (1.37 to 1.61)
Gallbladder and biliary tract cancer	0.2 (0.0 to 0.3)	1.9 (1.5 to 2.1)	2.1 (1.7 to 2.3)	0.5 (0.2 to 0.8)	1.8 (1.4 to 2.1)	2.3 (1.9 to 2.6)	0.35 (0.07 to 0.68)	1.06 (0.86 to 1.29)	0.91 (0.73 to 1.03)
Kidney cancer	1.0 (0.8 to 1.3)	1.9 (1.6 to 2.2)	3.0 (2.8 to 3.2)	0.4 (0.3 to 0.5)	0.9 (0.8 to 1.1)	1.3 (1.2 to 1.4)	2.64 (2.08 to 3.25)	2.10 (1.87 to 2.40)	2.25 (2.12 to 2.41)
Larynx cancer	2.1 (1.9 to 2.3)	0.6 (0.5 to 0.8)	2.7 (2.5 to 3.0)	0.1 (0.1 to 0.2)	0.3 (0.2 to 0.3)	0.4 (0.4 to 0.5)	15.85 (13.61 to 18.16)	2.28 (1.83 to 2.81)	6.74 (6.00 to 7.50)
Leukaemia	1.6 (1.0 to 2.1)	3.6 (3.0 to 4.3)	5.2 (4.6 to 5.7)	0.6 (0.4 to 0.9)	2.9 (2.5 to 3.3)	3.5 (3.2 to 3.8)	2.66 (1.90 to 3.53)	1.25 (1.05 to 1.47)	1.49 (1.29 to 1.68)
Lip and oral cavity cancer	2.4 (2.1 to 2.7)	1.0 (0.8 to 1.2)	3.4 (3.1 to 3.8)	0.7 (0.6 to 0.8)	0.9 (0.8 to 1.0)	1.6 (1.4 to 1.7)	3.49 (2.89 to 4.22)	1.16 (0.96 to 1.44)	2.19 (1.93 to 2.47)
Liver cancer	5.0 (4.2 to 5.8)	3.7 (3.0 to 4.6)	8.7 (7.9 to 9.6)	1.5 (1.2 to 1.8)	2.0 (1.7 to 2.4)	3.5 (3.1 to 3.8)	3.43 (2.79 to 4.17)	1.88 (1.49 to 2.31)	2.53 (2.21 to 2.86)
Mesothelioma	0.6 (0.5 to 0.6)	0.0 (0.0 to 0.0)	0.6 (0.6 to 0.6)	0.2 (0.1 to 0.2)	0.0 (0.0 to 0.0)	0.2 (0.1 to 0.2)	3.51 (3.07 to 5.03)	1.52 (0.95 to 2.28)	3.25 (2.88 to 4.45)
Multiple myeloma	0.1 (0.0 to 0.2)	1.6 (1.3 to 1.7)	1.7 (1.4 to 1.8)	0.1 (0.0 to 0.2)	1.1 (0.9 to 1.3)	1.2 (1.0 to 1.3)	1.26 (0.32 to 3.31)	1.42 (1.16 to 1.66)	1.40 (1.18 to 1.62)
Nasopharynx cancer	0.8 (0.7 to 0.9)	0.5 (0.4 to 0.6)	1.3 (1.2 to 1.4)	0.1 (0.1 to 0.1)	0.4 (0.3 to 0.4)	0.5 (0.4 to 0.5)	10.30 (8.37 to 12.79)	1.25 (1.00 to 1.54)	2.72 (2.33 to 3.14)
Non-Hodgkin lymphoma	0.2 (0.1 to 0.4)	3.8 (3.4 to 4.1)	4.0 (3.7 to 4.2)	0.1 (0.0 to 0.3)	2.4 (2.1 to 2.6)	2.5 (2.3 to 2.7)	1.84 (0.51 to 6.31)	1.57 (1.43 to 1.73)	1.58 (1.46 to 1.69)
Oesophageal cancer	7.4 (6.2 to 8.5)	2.3 (1.6 to 3.0)	9.7 (8.3 to 11.0)	1.4 (1.0 to 1.9)	1.6 (1.1 to 2.1)	3.0 (2.5 to 3.4)	5.48 (3.86 to 7.56)	1.44 (1.00 to 2.08)	3.22 (2.70 to 3.84)

	Global								
	Male			Female			Male:Female Ratio		
**Cancer**	**Age-standardised mortality rate attributable to risks assessed (95% UI)**	**Age-standardised mortality rate not attributable to risks assessed (95% UI)**	**Total age-standardised mortality rates (95% UI)**	**Age-standardised mortality rate attributable to risks assessed (95% UI)**	**Age-standardised mortality rate not attributable to risks assessed (95% UI)**	**Total age-standardised mortality rates (95% UI)**	**Age-standardised mortality rate attributable to risks assessed (95% UI)**	**Age-standardised mortality rate not attributable to risks assessed (95% UI)**	**Total age-standardised mortality rates (95% UI)**
Other pharynx cancer	1.6 (1.4 to 1.8)	0.6 (0.5 to 0.8)	2.2 (2.0 to 2.5)	0.1 (0.1 to 0.2)	0.5 (0.4 to 0.5)	0.6 (0.5 to 0.7)	11.27 (9.36 to 13.37)	1.33 (1.00 to 1.70)	3.73 (3.08 to 4.53)
Ovarian cancer	NA	NA	NA	0.6 (0.3 to 1.1)	3.9 (3.3 to 4.5)	4.6 (4.0 to 5.0)	NA	NA	NA
Pancreatic cancer	2.7 (2.1 to 3.6)	4.8 (4.0 to 5.6)	7.5 (7.0 to 8.1)	1.7 (1.2 to 2.3)	4.1 (3.4 to 4.8)	5.8 (5.1 to 6.2)	1.67 (1.28 to 2.14)	1.17 (1.03 to 1.34)	1.31 (1.21 to 1.44)
Prostate cancer	0.9 (0.4 to 1.4)	14.4 (12.3 to 17.7)	15.3 (13.0 to 18.6)	NA	NA	NA	NA	NA	NA
Stomach cancer	5.2 (3.6 to 8.2)	11.4 (8.3 to 13.6)	16.6 (14.8 to 18.3)	0.9 (0.3 to 2.6)	7.0 (5.3 to 8.1)	7.9 (7.1 to 8.8)	7.38 (2.95 to 11.86)	1.63 (1.41 to 1.86)	2.10 (1.83 to 2.38)
Thyroid cancer	0.1 (0.0 to 0.1)	0.4 (0.4 to 0.5)	0.5 (0.5 to 0.6)	0.1 (0.0 to 0.1)	0.6 (0.5 to 0.6)	0.6 (0.5 to 0.7)	1.08 (0.36 to 1.95)	0.80 (0.67 to 0.92)	0.82 (0.73 to 0.92)
Tracheal, bronchus, and lung cancer	32.8 (29.6 to 35.8)	4.6 (3.8 to 5.5)	37.4 (34.1 to 40.7)	9.7 (8.6 to 11.0)	5.2 (4.3 to 6.2)	15.0 (13.5 to 16.4)	3.37 (2.95 to 3.82)	0.88 (0.76 to 1.02)	2.50 (2.22 to 2.79)
Uterine cancer	NA	NA	NA	0.8 (0.6 to 1.1)	1.3 (1.0 to 1.6)	2.1 (1.9 to 2.3)	NA	NA	NA

NA = not applicable due to sex restriction; UI = uncertainty interval.

Appendix Table 17: Percent of global risk-attributable deaths over total cancer risk-attributable deaths by sex in 2019

	Percent of risk-attributable cancer deaths over total cancer risk-attributable deaths (95% UI)		
**Cancer**	**Male**	**Female**	**Both**
Tracheal, bronchus, and lung cancer	42.2 (39.7 to 44.6)	27.2 (24.4 to 30.1)	36.9 (34.2 to 39.3)
Colon and rectum cancer	13.3 (12.1 to 14.5)	15.8 (13.6 to 17.8)	14.2 (12.6 to 15.5)
Oesophageal cancer	9.7 (8.4 to 10.7)	3.8 (2.7 to 5.0)	7.6 (6.5 to 8.5)
Cervical cancer	NA	17.9 (14.8 to 20.5)	6.3 (5.3 to 7.2)
Liver cancer	6.6 (5.8 to 7.4)	4.1 (3.4 to 4.7)	5.7 (5.0 to 6.4)
Stomach cancer	6.6 (4.9 to 9.9)	2.5 (1.0 to 6.8)	5.2 (3.5 to 8.7)
Breast cancer	0.1 (0.1 to 0.1)	11.0 (8.6 to 13.7)	4.0 (3.0 to 5.1)
Pancreatic cancer	3.5 (2.8 to 4.4)	4.6 (3.5 to 5.8)	3.9 (3.2 to 4.8)
Lip and oral cavity cancer	3.3 (2.8 to 3.7)	1.9 (1.5 to 2.4)	2.8 (2.4 to 3.2)
Bladder cancer	2.8 (2.1 to 3.4)	0.8 (0.5 to 1.2)	2.1 (1.6 to 2.6)
Larynx cancer	2.8 (2.5 to 3.2)	0.4 (0.3 to 0.5)	2.0 (1.7 to 2.2)
Leukaemia	2.0 (1.4 to 2.6)	1.7 (1.1 to 2.3)	1.9 (1.3 to 2.5)
Other pharynx cancer	2.2 (1.9 to 2.6)	0.4 (0.3 to 0.5)	1.6 (1.4 to 1.8)
Kidney cancer	1.3 (1.0 to 1.7)	1.1 (0.8 to 1.4)	1.3 (1.0 to 1.5)
Uterine cancer	NA	2.3 (1.7 to 3.0)	0.8 (0.6 to 1.1)
Nasopharynx cancer	1.1 (0.9 to 1.2)	0.2 (0.2 to 0.3)	0.8 (0.7 to 0.9)
Prostate cancer	1.0 (0.4 to 1.6)	NA	0.7 (0.3 to 1.0)
Ovarian cancer	NA	1.7 (0.8 to 2.8)	0.6 (0.3 to 1.0)
Mesothelioma	0.7 (0.6 to 0.8)	0.4 (0.3 to 0.5)	0.6 (0.5 to 0.7)
Gallbladder and biliary tract cancer	0.2 (0.0 to 0.4)	1.3 (0.7 to 2.0)	0.6 (0.3 to 0.9)
Non-Hodgkin lymphoma	0.3 (0.1 to 0.5)	0.4 (0.1 to 0.7)	0.3 (0.1 to 0.5)
Multiple myeloma	0.1 (0.0 to 0.3)	0.3 (0.1 to 0.5)	0.2 (0.1 to 0.3)
Thyroid cancer	0.1 (0.0 to 0.1)	0.2 (0.1 to 0.2)	0.1 (0.1 to 0.2)

UI = uncertainty interval. NA = not applicable due to sex restriction.

Appendix Table 18: Global risk-attributable deaths vs. total deaths for each cancer by sex in 2019

	**Male**			**Female**			**Both sexes combined**		
**Cancer**	**Risk-attributable deaths (95% UI)**	**Total deaths (95% UI)**	**% of deaths that are risk-attributable (95% UI)**	**Risk-attributable deaths (95% UI)**	**Total deaths (95% UI)**	**% of deaths that are risk-attributable (95% UI)**	**Risk-attributable deaths (95% UI)**	**Total deaths (95% UI)**	**% of deaths that are risk-attributable (95% UI)**
Tracheal, bronchus, and lung cancer	1 210 000 (1 100 000 to 1 330 000)	1 390 000 (1 260 000 to 1 510 000)	87.5 (85.7 to 89.6)	427 000 (376 000 to 481 000)	657 000 (590 000 to 719 000)	65.1 (60.5 to 70.1)	1 640 000 (1 500 000 to 1 780 000)	2 040 000 (1 880 000 to 2 190 000)	80.3 (77.5 to 83.2)
Colon and rectum cancer	383 000 (333 000 to 435 000)	594 000 (551 000 to 638 000)	64.4 (57.5 to 71.4)	249 000 (204 000 to 296 000)	492 000 (438 000 to 532 000)	50.6 (42.4 to 58.5)	632 000 (551 000 to 722 000)	1 090 000 (1 000 000 to 1 150 000)	58.2 (51.1 to 65.2)
Oesophageal cancer	279 000 (234 000 to 322 000)	366 000 (315 000 to 415 000)	76.4 (70.1 to 82.9)	60 400 (42 200 to 82 100)	133 000 (110 000 to 150 000)	45.7 (32.1 to 61.0)	340 000 (287 000 to 393 000)	498 000 (438 000 to 551 000)	68.2 (60.8 to 75.2)
Cervical cancer	NA	NA	NA	280 000 (239 000 to 314 000)	280 000 (239 000 to 314 000)	100.0 (100.0 to 100.0)	280 000 (239 000 to 314 000)	280 000 (239 000 to 314 000)	100.0 (100.0 to 100.0)
Liver cancer	190 000 (160 000 to 223 000)	334 000 (300 000 to 368 000)	57.1 (48.8 to 64.7)	64 300 (52 100 to 77 900)	151 000 (134 000 to 167 000)	42.6 (35.5 to 50.1)	255 000 (218 000 to 297 000)	485 000 (444 000 to 526 000)	52.6 (45.6 to 59.4)
Stomach cancer	192 000 (133 000 to 302 000)	612 000 (544 000 to 678 000)	31.3 (22.0 to 49.1)	40 500 (14 800 to 114 000)	346 000 (308 000 to 382 000)	11.7 (4.3 to 32.9)	232 000 (148 000 to 416 000)	957 000 (871 000 to 1 030 000)	24.2 (15.7 to 43.1)
Breast cancer	1 870 (1 490 to 2 270)	12 100 (10 700 to 13 300)	15.5 (12.7 to 18.3)	175 000 (124 000 to 238 000)	689 000 (635 000 to 740 000)	25.4 (18.3 to 34.0)	177 000 (126 000 to 240 000)	701 000 (647 000 to 752 000)	25.2 (18.2 to 33.7)
Pancreatic cancer	101 000 (78 400 to 132 000)	278 000 (258 000 to 299 000)	36.3 (28.5 to 46.5)	72 700 (50 800 to 98 900)	253 000 (226 000 to 274 000)	28.8 (20.5 to 38.6)	174 000 (134 000 to 225 000)	531 000 (492 000 to 567 000)	32.7 (25.6 to 41.7)
Lip and oral cavity cancer	93 600 (80 800 to 106 000)	132 000 (118 000 to 145 000)	71.1 (65.6 to 76.0)	30 400 (25 300 to 36 000)	67 800 (60 800 to 75 700)	44.9 (39.2 to 50.4)	124 000 (109 000 to 139 000)	199 000 (182 000 to 218 000)	62.2 (57.5 to 66.5)
Bladder cancer	79 500 (60 100 to 100 000)	169 000 (157 000 to 181 000)	47.0 (35.5 to 58.3)	13 200 (8 040 to 19 800)	59 500 (52 300 to 64 600)	22.2 (13.8 to 32.8)	92 700 (68 400 to 119 000)	229 000 (211 000 to 243 000)	40.6 (29.6 to 51.2)
Larynx cancer	81 600 (72 700 to 90 600)	106 000 (97 800 to 115 000)	77.3 (71.0 to 82.0)	5 890 (4 800 to 7 060)	17 800 (16 200 to 19 700)	33.1 (27.6 to 38.5)	87 500 (77 500 to 97 100)	123 000 (115 000 to 133 000)	70.9 (65.0 to 75.6)
Leukaemia	57 500 (38 800 to 76 800)	188 000 (165 000 to 208 000)	30.5 (20.5 to 40.7)	26 100 (16 600 to 37 900)	146 000 (132 000 to 158 000)	17.9 (11.8 to 25.7)	83 700 (56 500 to 112 000)	335 000 (307 000 to 360 000)	25.0 (17.1 to 33.2)
Other pharynx cancer	64 300 (55 900 to 72 900)	88 000 (78 000 to 98 700)	73.1 (67.2 to 77.9)	6 360 (5 210 to 7 610)	26 200 (22 500 to 30 500)	24.3 (20.2 to 28.6)	70 700 (61 500 to 79 500)	114 000 (103 000 to 126 000)	61.9 (56.2 to 66.8)
Kidney cancer	38 700 (29 000 to 48 900)	109 000 (101 000 to 116 000)	35.5 (27.0 to 44.5)	17 600 (12 500 to 24 000)	57 700 (52 200 to 61 900)	30.5 (21.8 to 40.9)	56 300 (42 400 to 71 200)	166 000 (155 000 to 176 000)	33.8 (25.8 to 42.3)
Uterine cancer	NA	NA	NA	36 500 (25 100 to 49 200)	91 600 (82 400 to 102 000)	39.8 (27.6 to 52.7)	36 500 (25 100 to 49 200)	91 600 (82 400 to 102 000)	39.8 (27.6 to 52.7)
Nasopharynx cancer	31 600 (26 400 to 36 900)	51 200 (46 000 to 57 000)	61.6 (53.3 to 68.7)	3 360 (2 480 to 4 270)	20 400 (18 200 to 22 800)	16.5 (12.6 to 20.4)	34 900 (29 000 to 40 900)	71 600 (65 400 to 77 600)	48.8 (41.8 to 55.1)
Prostate cancer	29 300 (12 800 to 46 600)	487 000 (420 000 to 594 000)	6.0 (2.7 to 9.3)	NA	NA	NA	29 300 (12 800 to 46 600)	487 000 (420 000 to 594 000)	6.0 (2.7 to 9.3)

	Male			Female			Both sexes combined		
**Cancer**	**Risk-attributable deaths (95% UI)**	**Total deaths (95% UI)**	**% of deaths that are risk-attributable (95% UI)**	**Risk-attributable deaths (95% UI)**	**Total deaths (95% UI)**	**% of deaths that are risk-attributable (95% UI)**	**Risk-attributable deaths (95% UI)**	**Total deaths (95% UI)**	**% of deaths that are risk-attributable (95% UI)**
Ovarian cancer	NA	NA	NA	27 200 (11 700 to 48 100)	198 000 (175 000 to 218 000)	13.7 (5.8 to 23.9)	27 200 (11 700 to 48 100)	198 000 (175 000 to 218 000)	13.7 (5.8 to 23.9)
Mesothelioma	19 800 (18 400 to 21 200)	21 200 (20 000 to 22 500)	93.2 (90.8 to 95.3)	7 030 (4 930 to 7 970)	8 030 (5 880 to 8 920)	87.5 (82.7 to 91.0)	26 800 (24 300 to 28 600)	29 300 (26 700 to 31 000)	91.7 (89.7 to 93.4)
Gallbladder and biliary tract cancer	5 830 (1 060 to 12 300)	73 000 (59 500 to 80 400)	8.0 (1.5 to 16.7)	20 300 (10 800 to 32 900)	99 500 (81 700 to 114 000)	20.4 (11.2 to 32.2)	26 100 (13 900 to 42 600)	172 000 (145 000 to 189 000)	15.2 (8.0 to 24.7)
Non-Hodgkin lymphoma	8 160 (3 020 to 15 800)	146 000 (136 000 to 155 000)	5.6 (2.1 to 10.9)	5 640 (978 to 11 800)	109 000 (98 900 to 117 000)	5.2 (0.9 to 10.6)	13 800 (5 810 to 24 500)	255 000 (238 000 to 270 000)	5.4 (2.3 to 9.6)
Multiple myeloma	3 920 (1 120 to 7 970)	60 400 (50 700 to 67 100)	6.5 (1.8 to 13.1)	4 090 (1 350 to 7 780)	53 000 (45 100 to 58 300)	7.7 (2.5 to 14.6)	8 020 (3 520 to 14 200)	113 000 (99 500 to 122 000)	7.1 (3.1 to 12.4)
Thyroid cancer	2 210 (639 to 4 320)	18 600 (16 800 to 20 200)	11.9 (3.5 to 23.1)	2 450 (1 310 to 4 000)	26 900 (23 700 to 29 300)	9.1 (4.9 to 14.9)	4 660 (2 290 to 7 890)	45 600 (41 300 to 48 800)	10.2 (5.0 to 17.0)

UI = uncertainty interval. NA = not applicable due to sex restriction.

Appendix Table 19: Deaths attributable vs. not attributable to risks assessed for each cancer type by sex in 2019 in high SDI locations

	High SDI								
	Male			Female			Male:Female Ratio		
**Cancer**	**Deaths attributable to risks assessed (95% UI)**	**Deaths not attributable to risks assessed (95% UI)**	**Total deaths (95% UI)**	**Deaths attributable to risks assessed (95% UI)**	**Deaths not attributable to risks assessed (95% UI)**	**Total deaths (95% UI)**	**Deaths attributable to risks assessed (95% UI)**	**Deaths not attributable to risks assessed (95% UI)**	**Total deaths (95% UI)**
Bladder cancer	22 900 (15 100 to 30 600)	31 900 (23 800 to 39 700)	54 800 (49 900 to 58 300)	6 190 (3 880 to 8 920)	16 000 (12 500 to 19 200)	22 200 (18 700 to 24 400)	3.75 (3.02 to 4.52)	1.99 (1.70 to 2.29)	2.47 (2.34 to 2.69)
Breast cancer	340 (279 to 406)	1 250 (1 140 to 1 350)	1 590 (1 460 to 1 700)	56 400 (43 100 to 73 100)	110 000 (91 400 to 125 000)	166 000 (150 000 to 175 000)	0.01 (0.00 to 0.01)	0.01 (0.01 to 0.01)	0.01 (0.01 to 0.01)
Cervical cancer	NA	NA	NA	26 200 (22 800 to 28 100)	0 (0 to 0)	26 200 (22 800 to 28 100)	NA	NA	NA
Colon and rectum cancer	113 000 (99 000 to 128 000)	59 800 (46 200 to 74 200)	173 000 (161 000 to 181 000)	84 900 (69 300 to 101 000)	69 400 (54 700 to 84 200)	154 000 (133 000 to 166 000)	1.34 (1.22 to 1.50)	0.86 (0.75 to 0.98)	1.12 (1.07 to 1.22)
Oesophageal cancer	48 000 (42 400 to 53 200)	14 200 (9 140 to 19 100)	62 200 (58 800 to 65 200)	10 700 (8 180 to 13 200)	6 230 (3 950 to 8 690)	16 900 (14 900 to 18 300)	4.55 (3.74 to 5.70)	2.34 (1.50 to 3.60)	3.68 (3.46 to 3.99)
Gallbladder and biliary tract cancer	1 790 (316 to 3 830)	20 200 (14 800 to 22 600)	22 000 (16 400 to 23 900)	5 230 (2 720 to 8 690)	21 200 (15 200 to 25 500)	26 500 (19 700 to 30 200)	0.35 (0.07 to 0.66)	0.96 (0.74 to 1.20)	0.84 (0.62 to 1.00)
Kidney cancer	15 200 (11 100 to 19 400)	25 700 (21 000 to 29 900)	40 800 (38 300 to 42 700)	7 930 (5 750 to 10 400)	14 600 (11 700 to 17 100)	22 500 (19 700 to 24 100)	1.93 (1.56 to 2.30)	1.76 (1.56 to 2.02)	1.82 (1.73 to 1.95)
Larynx cancer	10 400 (9 350 to 11 200)	1 970 (1 290 to 2 860)	12 400 (11 700 to 12 900)	1 520 (1 250 to 1 750)	757 (567 to 975)	2 280 (2 030 to 2 490)	6.85 (6.17 to 7.76)	2.59 (2.11 to 3.12)	5.43 (4.99 to 5.98)
Leukaemia	17 900 (10 500 to 25 100)	31 400 (24 300 to 39 000)	49 400 (46 200 to 51 600)	10 600 (6 740 to 14 900)	25 200 (19 800 to 29 400)	35 900 (30 700 to 38 400)	1.69 (1.29 to 2.06)	1.25 (1.09 to 1.45)	1.38 (1.29 to 1.50)
Lip and oral cavity cancer	14 500 (13 200 to 15 800)	5 010 (4 060 to 6 090)	19 500 (18 400 to 20 600)	4 890 (4 120 to 5 620)	4 920 (4 000 to 5 690)	9 810 (8 420 to 10 700)	2.98 (2.71 to 3.30)	1.02 (0.88 to 1.18)	2.00 (1.84 to 2.22)
Liver cancer	50 500 (44 800 to 56 400)	24 900 (19 800 to 30 300)	75 400 (70 200 to 79 200)	18 900 (16 000 to 22 200)	17 900 (14 300 to 21 400)	36 800 (31 500 to 39 900)	2.68 (2.35 to 3.06)	1.39 (1.14 to 1.68)	2.05 (1.90 to 2.30)
Mesothelioma	11 800 (11 100 to 12 400)	234 (158 to 322)	12 100 (11 300 to 12 600)	2 560 (1 730 to 3 040)	159 (117 to 210)	2 720 (1 870 to 3 230)	4.69 (3.87 to 6.88)	1.50 (0.92 to 2.27)	4.50 (3.74 to 6.54)

	High SDI								
	Male			Female			Male:Female Ratio		
**Cancer**	**Deaths attributable to risks assessed (95% UI)**	**Deaths not attributable to risks assessed (95% UI)**	**Total deaths (95% UI)**	**Deaths attributable to risks assessed (95% UI)**	**Deaths not attributable to risks assessed (95% UI)**	**Total deaths (95% UI)**	**Deaths attributable to risks assessed (95% UI)**	**Deaths not attributable to risks assessed (95% UI)**	**Total deaths (95% UI)**
Multiple myeloma	1 980 (565 to 4 040)	23 900 (18 800 to 26 300)	25 800 (20 700 to 28 000)	1 890 (630 to 3 580)	20 400 (16 600 to 23 000)	22 300 (18 300 to 24 600)	1.15 (0.30 to 3.00)	1.18 (0.97 to 1.39)	1.17 (0.94 to 1.33)
Nasopharynx cancer	2 810 (2 430 to 3 160)	1 390 (1 070 to 1 750)	4 200 (3 880 to 4 530)	557 (448 to 660)	795 (674 to 917)	1 350 (1 230 to 1 460)	5.05 (4.58 to 5.59)	1.75 (1.51 to 2.00)	3.11 (2.93 to 3.33)
Non-Hodgkin lymphoma	3 400 (1 270 to 6 470)	43 000 (39 100 to 46 300)	46 400 (43 200 to 48 700)	2 320 (399 to 4 710)	35 700 (30 800 to 39 100)	38 100 (33 000 to 40 800)	1.58 (0.46 to 5.33)	1.21 (1.09 to 1.34)	1.22 (1.15 to 1.32)
Other pharynx cancer	11 300 (10 300 to 12 200)	2 380 (1 850 to 3 080)	13 700 (12 800 to 14 500)	1 800 (1 570 to 2 000)	953 (777 to 1 140)	2 750 (2 490 to 2 940)	6.29 (5.87 to 6.84)	2.50 (2.22 to 2.81)	4.97 (4.70 to 5.39)
Ovarian cancer	NA	NA	NA	10 300 (4 810 to 17 200)	46 300 (38 700 to 52 800)	56 600 (50 400 to 61 300)	NA	NA	NA
Pancreatic cancer	35 400 (26 600 to 46 900)	60 300 (48 800 to 70 200)	95 700 (90 000 to 100 000)	33 800 (24 900 to 44 100)	60 200 (48 500 to 70 700)	94 000 (82 100 to 101 000)	1.05 (0.82 to 1.32)	1.00 (0.86 to 1.16)	1.02 (0.97 to 1.11)
Prostate cancer	9 160 (3 960 to 15 400)	149 000 (122 000 to 201 000)	158 000 (131 000 to 215 000)	NA	NA	NA	NA	NA	NA
Stomach cancer	26 000 (17 800 to 42 500)	62 700 (46 500 to 71 700)	88 700 (81 300 to 93 200)	8 690 (4 460 to 20 200)	47 400 (35 600 to 54 800)	56 100 (46 900 to 61 300)	3.29 (2.07 to 4.10)	1.32 (1.22 to 1.48)	1.59 (1.49 to 1.75)
Thyroid cancer	624 (195 to 1 200)	3 250 (2 550 to 3 750)	3 870 (3 260 to 4 100)	576 (307 to 931)	4 930 (4 110 to 5 520)	5 510 (4 630 to 5 980)	1.11 (0.37 to 1.98)	0.66 (0.54 to 0.77)	0.70 (0.65 to 0.78)
Tracheal, bronchus, and lung cancer	311 000 (290 000 to 327 000)	43 200 (34 300 to 52 600)	354 000 (333 000 to 367 000)	161 000 (143 000 to 175 000)	62 500 (50 900 to 72 200)	224 000 (201 000 to 237 000)	1.93 (1.82 to 2.05)	0.69 (0.58 to 0.81)	1.58 (1.52 to 1.67)
Uterine cancer	NA	NA	NA	12 000 (8 390 to 15 800)	14 700 (10 900 to 18 400)	26 600 (24 000 to 28 100)	NA	NA	NA

UI = uncertainty interval; NA = not applicable due to sex restriction.

Appendix Table 20: Age-standardised death rates attributable vs. not attributable to risks assessed for each cancer type by sex in 2019 in high SDI locations

	High SDI								
	Male			Female			Male:Female Ratio		
Cancer	Age-standardised mortality rate attributable to risks assessed (95% UI)	Age-standardised mortality rate not attributable to risks assessed (95% UI)	Total age-standardised mortality rate (95% UI)	Age-standardised mortality rate attributable to risks assessed (95% UI)	Age-standardised mortality rate not attributable to risks assessed (95% UI)	Total age-standardised mortality rate (95% UI)	Age-standardised mortality rate attributable to risks assessed (95% UI)	Age-standardised mortality rate not attributable to risks assessed (95% UI)	Total age-standardised mortality rate (95% UI)
Bladder cancer	2.6 (1.7 to 3.5)	3.6 (2.7 to 4.5)	6.2 (5.6 to 6.6)	0.5 (0.3 to 0.7)	1.2 (1.0 to 1.5)	1.7 (1.5 to 1.9)	5.07 (4.17 to 6.02)	2.94 (2.51 to 3.34)	3.57 (3.41 to 3.77)
Breast cancer	0.0 (0.0 to 0.0)	0.1 (0.1 to 0.2)	0.2 (0.2 to 0.2)	5.6 (4.3 to 7.0)	11.2 (9.5 to 12.6)	16.7 (15.6 to 17.5)	0.01 (0.01 to 0.01)	0.01 (0.01 to 0.02)	0.01 (0.01 to 0.01)
Cervical cancer	NA	NA	NA	2.9 (2.6 to 3.1)	0.0 (0.0 to 0.0)	2.9 (2.6 to 3.1)	NA	NA	NA
Colon and rectum cancer	13.1 (11.5 to 14.8)	6.9 (5.4 to 8.6)	20.1 (18.8 to 21.0)	7.3 (6.1 to 8.5)	5.9 (4.7 to 7.1)	13.1 (11.7 to 14.0)	1.81 (1.68 to 1.99)	1.18 (1.04 to 1.32)	1.53 (1.47 to 1.61)
Gallbladder and biliary tract cancer	0.2 (0.0 to 0.4)	2.3 (1.7 to 2.6)	2.5 (1.9 to 2.7)	0.5 (0.2 to 0.7)	1.7 (1.3 to 2.1)	2.2 (1.7 to 2.5)	0.46 (0.10 to 0.88)	1.35 (1.00 to 1.66)	1.15 (0.84 to 1.32)
Kidney cancer	1.8 (1.3 to 2.3)	3.0 (2.5 to 3.5)	4.8 (4.5 to 5.0)	0.7 (0.5 to 0.9)	1.3 (1.1 to 1.5)	2.0 (1.8 to 2.1)	2.44 (1.98 to 2.91)	2.34 (2.08 to 2.64)	2.37 (2.27 to 2.48)
Larynx cancer	1.2 (1.1 to 1.3)	0.2 (0.2 to 0.3)	1.5 (1.4 to 1.5)	0.2 (0.1 to 0.2)	0.1 (0.1 to 0.1)	0.2 (0.2 to 0.2)	7.96 (7.18 to 8.92)	3.19 (2.59 to 3.85)	6.42 (5.93 to 6.98)
Leukaemia	2.1 (1.2 to 2.9)	3.9 (3.1 to 4.8)	6.0 (5.7 to 6.3)	1.0 (0.7 to 1.4)	2.5 (2.1 to 2.9)	3.5 (3.2 to 3.7)	2.09 (1.59 to 2.55)	1.57 (1.39 to 1.80)	1.72 (1.63 to 1.81)
Lip and oral cavity cancer	1.8 (1.6 to 1.9)	0.6 (0.5 to 0.7)	2.4 (2.2 to 2.5)	0.5 (0.4 to 0.5)	0.4 (0.4 to 0.5)	0.9 (0.8 to 0.9)	3.84 (3.53 to 4.17)	1.44 (1.27 to 1.62)	2.69 (2.51 to 2.91)
Liver cancer	5.9 (5.2 to 6.6)	2.9 (2.4 to 3.6)	8.9 (8.3 to 9.3)	1.8 (1.5 to 2.0)	1.5 (1.2 to 1.8)	3.3 (2.9 to 3.5)	3.36 (2.97 to 3.82)	1.94 (1.60 to 2.30)	2.70 (2.52 to 2.93)
Mesothelioma	1.3 (1.2 to 1.4)	0.0 (0.0 to 0.0)	1.4 (1.3 to 1.4)	0.2 (0.2 to 0.3)	0.0 (0.0 to 0.0)	0.3 (0.2 to 0.3)	5.75 (4.69 to 8.32)	1.55 (0.98 to 2.29)	5.37 (4.43 to 7.60)
Multiple myeloma	0.2 (0.1 to 0.5)	2.7 (2.2 to 3.0)	3.0 (2.4 to 3.2)	0.2 (0.1 to 0.3)	1.8 (1.5 to 2.1)	2.0 (1.7 to 2.3)	1.45 (0.37 to 3.77)	1.53 (1.22 to 1.78)	1.51 (1.18 to 1.68)

	High SDI								
	Male			Female			Male:Female Ratio		
**Cancer**	**Age-standardised mortality rate attributable to risks assessed (95% UI)**	**Age-standardised mortality rate not attributable to risks assessed (95% UI)**	**Total age-standardised mortality rate (95% UI)**	**Age-standardised mortality rate attributable to risks assessed (95% UI)**	**Age-standardised mortality rate not attributable to risks assessed (95% UI)**	**Total age-standardised mortality rate (95% UI)**	**Age-standardised mortality rate attributable to risks assessed (95% UI)**	**Age-standardised mortality rate not attributable to risks assessed (95% UI)**	**Total age-standardised mortality rate (95% UI)**
Nasopharynx cancer	0.4 (0.3 to 0.4)	0.2 (0.1 to 0.2)	0.5 (0.5 to 0.6)	0.1 (0.0 to 0.1)	0.1 (0.1 to 0.1)	0.2 (0.1 to 0.2)	5.77 (5.26 to 6.36)	2.00 (1.74 to 2.28)	3.53 (3.34 to 3.73)
Non-Hodgkin lymphoma	0.4 (0.2 to 0.8)	5.1 (4.6 to 5.5)	5.5 (5.1 to 5.7)	0.2 (0.0 to 0.4)	3.2 (2.8 to 3.5)	3.4 (3.0 to 3.6)	2.04 (0.59 to 6.95)	1.60 (1.45 to 1.75)	1.62 (1.54 to 1.71)
Oesophageal cancer	5.6 (5.0 to 6.2)	1.7 (1.1 to 2.2)	7.3 (6.9 to 7.6)	1.0 (0.8 to 1.2)	0.5 (0.3 to 0.7)	1.5 (1.4 to 1.6)	5.85 (4.83 to 7.28)	3.19 (2.02 to 4.95)	4.84 (4.58 to 5.13)
Other pharynx cancer	1.4 (1.3 to 1.5)	0.3 (0.2 to 0.4)	1.7 (1.6 to 1.8)	0.2 (0.2 to 0.2)	0.1 (0.1 to 0.1)	0.3 (0.3 to 0.3)	7.23 (6.78 to 7.81)	3.06 (2.75 to 3.41)	5.85 (5.56 to 6.29)
Ovarian cancer	NA	NA	NA	0.9 (0.4 to 1.6)	4.7 (4.0 to 5.3)	5.7 (5.2 to 6.1)	NA	NA	NA
Pancreatic cancer	4.1 (3.1 to 5.4)	7.0 (5.7 to 8.2)	11.1 (10.4 to 11.6)	3.1 (2.3 to 3.9)	5.2 (4.3 to 6.1)	8.3 (7.4 to 8.9)	1.33 (1.04 to 1.67)	1.34 (1.16 to 1.53)	1.34 (1.27 to 1.42)
Prostate cancer	1.0 (0.4 to 1.7)	16.6 (13.6 to 22.3)	17.6 (14.7 to 23.9)	NA	NA	NA	NA	NA	NA
Stomach cancer	3.0 (2.0 to 4.9)	7.2 (5.4 to 8.3)	10.2 (9.4 to 10.7)	0.8 (0.4 to 1.7)	4.0 (3.0 to 4.5)	4.7 (4.1 to 5.1)	4.29 (2.81 to 5.25)	1.82 (1.70 to 1.97)	2.16 (2.07 to 2.30)
Thyroid cancer	0.1 (0.0 to 0.1)	0.4 (0.3 to 0.4)	0.5 (0.4 to 0.5)	0.1 (0.0 to 0.1)	0.4 (0.4 to 0.5)	0.5 (0.4 to 0.5)	1.44 (0.48 to 2.54)	0.89 (0.74 to 1.04)	0.95 (0.88 to 1.03)
Tracheal, bronchus, and lung cancer	35.5 (33.2 to 37.3)	5.1 (4.1 to 6.2)	40.6 (38.3 to 42.1)	15.3 (13.8 to 16.4)	5.8 (4.8 to 6.7)	21.1 (19.4 to 22.1)	2.33 (2.21 to 2.43)	0.88 (0.76 to 1.03)	1.93 (1.87 to 1.99)
Uterine cancer	NA	NA	NA	1.2 (0.8 to 1.5)	1.4 (1.0 to 1.7)	2.5 (2.3 to 2.6)	NA	NA	NA

UI = uncertainty interval; NA = not applicable due to sex restriction.

Appendix Table 21: Deaths attributable vs. not attributable to risks assessed for each cancer type by sex in 2019 in non-high SDI locations

	Non-high SDI								
	Male			Female			Male:Female Ratio		
**Cancer**	**Deaths attributable to risks assessed (95% UI)**	**Deaths not attributable to risks assessed (95% UI)**	**Total deaths (95% UI)**	**Deaths attributable to risks assessed (95% UI)**	**Deaths not attributable to risks assessed (95% UI)**	**Total deaths (95% UI)**	**Deaths attributable to risks assessed (95% UI)**	**Deaths not attributable to risks assessed (95% UI)**	**Total deaths (95% UI)**
Bladder cancer	56 600 (43 300 to 70 100)	57 700 (46 100 to 71 400)	114 000 (105 000 to 124 000)	7 010 (3 950 to 11 200)	30 300 (25 400 to 35 100)	37 300 (33 200 to 41 000)	8.55 (5.55 to 12.34)	1.91 (1.59 to 2.24)	3.07 (2.78 to 3.38)
Breast cancer	1 530 (1 190 to 1 880)	8 980 (7 790 to 10 100)	10 500 (9 140 to 11 700)	118 000 (80 600 to 166 000)	404 000 (346 000 to 462 000)	522 000 (477 000 to 567 000)	0.01 (0.01 to 0.02)	0.02 (0.02 to 0.03)	0.02 (0.02 to 0.02)
Cervical cancer	NA	NA	NA	254 000 (213 000 to 287 000)	0 (0 to 0)	254 000 (213 000 to 287 000)	NA	NA	NA
Colon and rectum cancer	269 000 (230 000 to 310 000)	151 000 (121 000 to 183 000)	421 000 (381 000 to 461 000)	164 000 (134 000 to 196 000)	173 000 (143 000 to 206 000)	337 000 (305 000 to 370 000)	1.65 (1.44 to 1.92)	0.88 (0.74 to 1.02)	1.25 (1.11 to 1.40)
Oesophageal cancer	231 000 (190 000 to 272 000)	72 200 (51 000 to 94 500)	303 000 (253 000 to 352 000)	49 700 (33 600 to 69 600)	65 800 (43 500 to 85 000)	116 000 (93 800 to 132 000)	4.80 (3.21 to 6.80)	1.12 (0.78 to 1.64)	2.64 (2.14 to 3.26)
Gallbladder and biliary tract cancer	4 040 (745 to 8 560)	47 000 (37 900 to 54 100)	51 000 (41 400 to 57 500)	15 100 (8 120 to 24 500)	57 900 (44 700 to 70 000)	73 000 (59 500 to 86 500)	0.27 (0.06 to 0.53)	0.82 (0.64 to 1.03)	0.70 (0.56 to 0.82)
Kidney cancer	23 500 (17 700 to 29 800)	44 400 (37 200 to 51 500)	67 900 (62 100 to 73 900)	9 680 (6 600 to 13 600)	25 400 (21 400 to 29 400)	35 100 (32 100 to 38 400)	2.47 (1.87 to 3.23)	1.75 (1.53 to 2.04)	1.94 (1.76 to 2.13)
Larynx cancer	71 100 (63 200 to 79 500)	22 000 (17 100 to 27 900)	93 100 (85 900 to 102 000)	4 370 (3 440 to 5 340)	11 100 (9 810 to 12 700)	15 500 (14 000 to 17 300)	16.43 (13.67 to 19.51)	1.97 (1.58 to 2.43)	6.02 (5.26 to 6.79)
Leukaemia	39 600 (26 600 to 53 600)	99 400 (80 400 to 119 000)	139 000 (118 000 to 158 000)	15 500 (9 380 to 23 500)	94 700 (81 600 to 106 000)	110 000 (99 000 to 121 000)	2.64 (1.75 to 3.71)	1.05 (0.84 to 1.28)	1.27 (1.05 to 1.48)
Lip and oral cavity cancer	79 000 (66 700 to 90 800)	33 000 (26 200 to 41 600)	112 000 (98 200 to 126 000)	25 500 (20 500 to 30 900)	32 400 (27 800 to 37 700)	58 000 (51 200 to 65 300)	3.12 (2.51 to 3.93)	1.02 (0.81 to 1.30)	1.94 (1.67 to 2.23)
Liver cancer	140 000 (112 000 to 169 000)	118 000 (92 600 to 148 000)	258 000 (224 000 to 293 000)	45 300 (35 500 to 56 700)	68 700 (57 100 to 82 000)	114 000 (100 000 to 129 000)	3.11 (2.43 to 3.94)	1.73 (1.32 to 2.19)	2.27 (1.89 to 2.69)
Mesothelioma	7 960 (7 120 to 9 140)	1 200 (845 to 1 620)	9 160 (8 410 to 10 400)	4 470 (2 970 to 5 230)	835 (626 to 1 080)	5 310 (3 790 to 6 060)	1.81 (1.50 to 2.72)	1.47 (0.91 to 2.20)	1.75 (1.47 to 2.44)

	Non-high SDI								
	Male			Female			Male:Female Ratio		
**Cancer**	**Deaths attributable to risks assessed (95% UI)**	**Deaths not attributable to risks assessed (95% UI)**	**Total deaths (95% UI)**	**Deaths attributable to risks assessed (95% UI)**	**Deaths not attributable to risks assessed (95% UI)**	**Total deaths (95% UI)**	**Deaths attributable to risks assessed (95% UI)**	**Deaths not attributable to risks assessed (95% UI)**	**Total deaths (95% UI)**
Multiple myeloma	1 950 (551 to 4 050)	32 600 (26 000 to 37 400)	34 600 (27 800 to 39 600)	2 200 (720 to 4 240)	28 500 (23 700 to 32 800)	30 700 (25 900 to 34 700)	0.96 (0.25 to 2.60)	1.15 (0.91 to 1.41)	1.13 (0.91 to 1.37)
Nasopharynx cancer	28 700 (23 900 to 34 000)	18 300 (14 600 to 22 700)	47 000 (41 900 to 52 600)	2 800 (2 010 to 3 640)	16 200 (14 300 to 18 400)	19 000 (16 800 to 21 400)	10.41 (8.24 to 13.26)	1.13 (0.90 to 1.40)	2.48 (2.10 to 2.89)
Non-Hodgkin lymphoma	4 760 (1 760 to 9 430)	94 400 (84 900 to 104 000)	99 100 (90 800 to 108 000)	3 310 (554 to 6 930)	67 600 (60 400 to 74 500)	70 900 (64 600 to 77 400)	1.55 (0.42 to 5.42)	1.40 (1.24 to 1.57)	1.40 (1.26 to 1.55)
Other pharynx cancer	53 000 (45 100 to 61 100)	21 300 (16 400 to 27 200)	74 300 (64 300 to 84 800)	4 560 (3 540 to 5 740)	18 900 (15 700 to 22 500)	23 400 (19 800 to 27 600)	11.76 (9.28 to 14.54)	1.14 (0.84 to 1.48)	3.20 (2.56 to 3.98)
Ovarian cancer	NA	NA	NA	16 900 (6 640 to 30 700)	125 000 (103 000 to 145 000)	142 000 (122 000 to 160 000)	NA	NA	NA
Pancreatic cancer	65 500 (51 100 to 85 100)	117 000 (96 100 to 136 000)	182 000 (165 000 to 201 000)	38 900 (26 200 to 55 500)	120 000 (101 000 to 139 000)	159 000 (143 000 to 174 000)	1.73 (1.26 to 2.35)	0.98 (0.84 to 1.15)	1.15 (1.02 to 1.31)
Prostate cancer	20 100 (8 610 to 32 000)	308 000 (262 000 to 357 000)	328 000 (279 000 to 383 000)	NA	NA	NA	NA	NA	NA
Stomach cancer	166 000 (114 000 to 261 000)	357 000 (259 000 to 434 000)	523 000 (457 000 to 589 000)	31 700 (9 980 to 94 800)	258 000 (194 000 to 302 000)	289 000 (257 000 to 323 000)	7.40 (2.55 to 12.83)	1.39 (1.16 to 1.62)	1.81 (1.53 to 2.10)
Thyroid cancer	1 590 (474 to 3 160)	13 200 (11 000 to 15 100)	14 800 (13 200 to 16 300)	1 870 (995 to 3 070)	19 500 (16 800 to 21 800)	21 400 (18 700 to 23 600)	0.87 (0.29 to 1.58)	0.68 (0.56 to 0.79)	0.69 (0.60 to 0.80)
Tracheal, bronchus, and lung cancer	902 000 (793 000 to 1 020 000)	130 000 (106 000 to 154 000)	1 030 000 (910 000 to 1 160 000)	266 000 (228 000 to 309 000)	167 000 (137 000 to 201 000)	433 000 (379 000 to 490 000)	3.41 (2.82 to 4.04)	0.78 (0.65 to 0.92)	2.39 (2.01 to 2.79)
Uterine cancer	NA	NA	NA	24 500 (16 500 to 33 700)	40 400 (31 200 to 50 200)	64 900 (57 600 to 74 100)	NA	NA	NA

UI = uncertainty interval; NA = not applicable due to sex restriction.

Appendix Table 22: Age-standardised death rates attributable vs. not attributable to risks assessed for each cancer type by sex in 2019 in non-high SDI locations

	Non-high SDI								
	Male			Female			Male:Female Ratio		
Cancer	Age-standardised mortality rate attributable to risks assessed (95% UI)	Age-standardised mortality rate not attributable to risks assessed (95% UI)	Total age-standardised mortality rate (95% UI)	Age-standardised mortality rate attributable to risks assessed (95% UI)	Age-standardised mortality rate not attributable to risks assessed (95% UI)	Total age-standardised mortality rate (95% UI)	Age-standardised mortality rate attributable to risks assessed (95% UI)	Age-standardised mortality rate not attributable to risks assessed (95% UI)	Total age-standardised mortality rate (95% UI)
Bladder cancer	2.2 (1.7 to 2.7)	2.4 (1.9 to 2.9)	4.6 (4.2 to 4.9)	0.2 (0.1 to 0.4)	1.0 (0.8 to 1.1)	1.2 (1.0 to 1.3)	10.49 (6.82 to 15.31)	2.49 (2.08 to 2.91)	3.88 (3.53 to 4.25)
Breast cancer	0.1 (0.0 to 0.1)	0.3 (0.3 to 0.4)	0.4 (0.3 to 0.4)	3.5 (2.4 to 4.9)	11.8 (10.1 to 13.5)	15.3 (14.0 to 16.6)	0.02 (0.01 to 0.02)	0.03 (0.02 to 0.03)	0.02 (0.02 to 0.03)
Cervical cancer	NA	NA	NA	7.4 (6.2 to 8.3)	0.0 (0.0 to 0.0)	7.4 (6.2 to 8.3)	NA	NA	NA
Colon and rectum cancer	9.8 (8.4 to 11.2)	5.5 (4.3 to 6.6)	15.2 (13.9 to 16.6)	5.0 (4.1 to 6.0)	5.3 (4.4 to 6.3)	10.3 (9.3 to 11.4)	1.94 (1.70 to 2.24)	1.04 (0.88 to 1.21)	1.48 (1.32 to 1.65)
Gallbladder and biliary tract cancer	0.1 (0.0 to 0.3)	1.7 (1.4 to 2.0)	1.8 (1.5 to 2.1)	0.5 (0.2 to 0.7)	1.8 (1.4 to 2.1)	2.2 (1.8 to 2.6)	0.32 (0.07 to 0.63)	0.98 (0.77 to 1.22)	0.84 (0.67 to 0.98)
Kidney cancer	0.8 (0.6 to 1.0)	1.5 (1.3 to 1.8)	2.4 (2.2 to 2.6)	0.3 (0.2 to 0.4)	0.8 (0.7 to 0.9)	1.1 (1.0 to 1.2)	2.84 (2.15 to 3.69)	2.00 (1.75 to 2.32)	2.21 (2.02 to 2.42)
Larynx cancer	2.4 (2.1 to 2.7)	0.7 (0.6 to 0.9)	3.1 (2.9 to 3.4)	0.1 (0.1 to 0.2)	0.3 (0.3 to 0.4)	0.5 (0.4 to 0.5)	18.42 (15.32 to 21.84)	2.23 (1.78 to 2.75)	6.77 (5.94 to 7.62)
Leukaemia	1.4 (0.9 to 1.9)	3.3 (2.7 to 3.9)	4.7 (3.9 to 5.3)	0.5 (0.3 to 0.7)	2.9 (2.5 to 3.2)	3.3 (3.0 to 3.7)	3.06 (2.01 to 4.32)	1.15 (0.93 to 1.40)	1.41 (1.17 to 1.64)
Lip and oral cavity cancer	2.6 (2.2 to 3.0)	1.1 (0.9 to 1.4)	3.7 (3.2 to 4.1)	0.8 (0.6 to 0.9)	1.0 (0.8 to 1.1)	1.7 (1.5 to 2.0)	3.42 (2.75 to 4.27)	1.14 (0.91 to 1.44)	2.13 (1.85 to 2.46)
Liver cancer	4.7 (3.8 to 5.6)	3.8 (3.0 to 4.8)	8.5 (7.4 to 9.6)	1.4 (1.1 to 1.7)	2.1 (1.7 to 2.5)	3.4 (3.0 to 3.9)	3.47 (2.71 to 4.39)	1.86 (1.43 to 2.33)	2.49 (2.08 to 2.92)
Mesothelioma	0.3 (0.3 to 0.3)	0.0 (0.0 to 0.0)	0.3 (0.3 to 0.4)	0.1 (0.1 to 0.2)	0.0 (0.0 to 0.0)	0.2 (0.1 to 0.2)	2.13 (1.77 to 3.17)	1.52 (0.94 to 2.29)	2.03 (1.72 to 2.83)
Multiple myeloma	0.1 (0.0 to 0.1)	1.1 (0.9 to 1.3)	1.2 (1.0 to 1.4)	0.1 (0.0 to 0.1)	0.9 (0.7 to 1.0)	0.9 (0.8 to 1.0)	1.10 (0.29 to 2.99)	1.34 (1.07 to 1.63)	1.32 (1.06 to 1.58)
Nasopharynx cancer	0.9 (0.7 to 1.1)	0.6 (0.5 to 0.7)	1.5 (1.3 to 1.6)	0.1 (0.1 to 0.1)	0.5 (0.4 to 0.5)	0.6 (0.5 to 0.6)	11.15 (8.84 to 14.19)	1.21 (0.96 to 1.50)	2.66 (2.25 to 3.10)
Non-Hodgkin lymphoma	0.2 (0.1 to 0.3)	3.2 (2.8 to 3.5)	3.3 (3.0 to 3.6)	0.1 (0.0 to 0.2)	2.0 (1.8 to 2.2)	2.1 (1.9 to 2.3)	1.72 (0.47 to 6.01)	1.56 (1.38 to 1.75)	1.56 (1.41 to 1.72)
Oesophageal cancer	7.9 (6.5 to 9.2)	2.5 (1.8 to 3.3)	10.4 (8.7 to 12.0)	1.5 (1.0 to 2.1)	2.0 (1.3 to 2.6)	3.5 (2.8 to 4.0)	5.41 (3.63 to 7.65)	1.28 (0.89 to 1.86)	2.98 (2.43 to 3.68)
Other pharynx cancer	1.7 (1.4 to 2.0)	0.7 (0.5 to 0.9)	2.4 (2.1 to 2.7)	0.1 (0.1 to 0.2)	0.6 (0.5 to 0.7)	0.7 (0.6 to 0.8)	12.75 (10.07 to 15.69)	1.25 (0.93 to 1.63)	3.50 (2.82 to 4.36)

	Non-high SDI								
	Male			Female			Male:Female Ratio		
**Cancer**	**Age-standardised mortality rate attributable to risks assessed (95% UI)**	**Age-standardised mortality rate not attributable to risks assessed (95% UI)**	**Total age-standardised mortality rate (95% UI)**	**Age-standardised mortality rate attributable to risks assessed (95% UI)**	**Age-standardised mortality rate not attributable to risks assessed (95% UI)**	**Total age-standardised mortality rate (95% UI)**	**Age-standardised mortality rate attributable to risks assessed (95% UI)**	**Age-standardised mortality rate not attributable to risks assessed (95% UI)**	**Total age-standardised mortality rate (95% UI)**
Ovarian cancer	NA	NA	NA	0.5 (0.2 to 0.9)	3.6 (3.0 to 4.2)	4.1 (3.6 to 4.7)	NA	NA	NA
Pancreatic cancer	2.3 (1.8 to 3.0)	4.0 (3.3 to 4.7)	6.3 (5.8 to 7.0)	1.2 (0.8 to 1.7)	3.6 (3.0 to 4.2)	4.8 (4.3 to 5.3)	1.98 (1.45 to 2.67)	1.11 (0.96 to 1.30)	1.31 (1.16 to 1.49)
Prostate cancer	0.8 (0.3 to 1.3)	13.3 (11.2 to 15.5)	14.1 (11.9 to 16.6)	NA	NA	NA	NA	NA	NA
Stomach cancer	5.8 (4.0 to 9.1)	12.5 (9.1 to 15.1)	18.3 (16.1 to 20.5)	1.0 (0.3 to 2.9)	7.8 (5.9 to 9.2)	8.8 (7.8 to 9.8)	8.49 (2.95 to 14.65)	1.60 (1.35 to 1.85)	2.09 (1.78 to 2.41)
Thyroid cancer	0.1 (0.0 to 0.1)	0.5 (0.4 to 0.5)	0.5 (0.5 to 0.6)	0.1 (0.0 to 0.1)	0.6 (0.5 to 0.7)	0.6 (0.6 to 0.7)	0.99 (0.33 to 1.81)	0.79 (0.66 to 0.92)	0.81 (0.70 to 0.93)
Tracheal, bronchus, and lung cancer	31.4 (27.7 to 35.3)	4.4 (3.6 to 5.3)	35.9 (31.7 to 40.0)	8.0 (6.9 to 9.3)	5.0 (4.1 to 6.1)	13.0 (11.4 to 14.8)	3.94 (3.27 to 4.66)	0.89 (0.74 to 1.05)	2.76 (2.33 to 3.21)
Uterine cancer	NA	NA	NA	0.7 (0.5 to 1.0)	1.2 (0.9 to 1.5)	1.9 (1.7 to 2.2)	NA	NA	NA

UI = uncertainty interval; NA = not applicable due to sex restriction.

Appendix Table 23: Proportion of total cancer deaths vs. risk-attributable cancer deaths in high and non-high SDI settings in 2019, both sexes combined

Metric	Location	% of global cancer deaths (95% UI)
Risk-attributable cancer deaths	High SDI	26.5 (24.9 to 28.1)
Total cancer deaths	High SDI	25.4 (24.0 to 26.7)
Risk-attributable cancer deaths	Non-high SDI	73.5 (71.9 to 75.1)
Total cancer deaths	Non-high SDI	74.6 (73.3 to 76.0)

SDI = Socio-demographic Index; UI = uncertainty interval.

Appendix Table 24: Global percentages of risk-attributable cancer deaths and DALYs out of total cancer deaths and DALYs for both sexes, males, and females in 2019

Cause	Deaths			DALYs		
	**% of risk-attributable cancer deaths out of total cancer deaths, both sexes (95% UI)**	**% of risk-attributable cancer deaths out of total cancer deaths, males (95% UI)**	**% of risk-attributable cancer deaths out of total cancer deaths, females (95% UI)**	**% of risk-attributable cancer DALYs out of total cancer DALYs, both sexes (95% UI)**	**% of risk-attributable cancer DALYs out of total cancer DALYs, males (95% UI)**	**% of risk-attributable cancer DALYs out of total cancer DALYs, females (95% UI)**
Total cancers	44.4 (41.3 to 48.4)	50.6 (47.8 to 54.1)	36.3 (32.5 to 41.3)	42.0 (39.1 to 45.6)	48.0 (45.3 to 51.5)	34.3 (30.9 to 38.7)
Total cancers excluding Non-melanoma skin cancer	44.7 (41.5 to 48.7)	50.9 (48.1 to 54.4)	36.5 (32.6 to 41.5)	42.2 (39.3 to 45.8)	48.3 (45.5 to 51.7)	34.4 (31.1 to 38.8)

Total cancers = all 29 cancer groups (risk-attributable as well as not attributable to risks) estimated in the GBD 2019 study, including non-melanoma skin cancer. The calculation for estimates presented in “Total cancers” row was: (*total deaths or DALYs due to risk-attributable cancers*) / (*total deaths or DALYs of all 29 cancer types)*. The calculation for estimates presented in “Total cancers excluding Non-melanoma skin cancer” was: (*total deaths or DALYs due to risk-attributable cancers*) / (*total deaths or DALYs of all cancer types excluding non-melanoma skin cancer*). DALY = disability-adjusted life-year; UI = uncertainty interval.

Appendix Table 25: Change in age-standardised DALY rates and absolute DALYs from 2010 to 2019 for all risk factors combined by SDI quintile and GBD super-region, both sexes combined

Location	Percent change of age-standardised DALY rates, 2010 – 2019 (95% UI)	Percent change of absolute DALYs, 2010 – 2019 (95% UI)
**SDI Quintile**		
High SDI	-10.0 (-12.1 to -7.8)	7.7 (5.2 to 10.3)
High-middle SDI	-10.2 (-17.9 to -2.1)	11.2 (1.5 to 21.3)
Middle SDI	-7.2 (-17.6 to 4.4)	21.9 (8.0 to 37.3)
Low-middle SDI	1.5 (-6.4 to 9.9)	31.4 (21.0 to 42.4)
Low SDI	-0.3 (-8.7 to 8.7)	32.7 (20.5 to 45.4)
**GBD super-region**		
Central Europe, Eastern Europe, and Central Asia	-9.0 (-16.0 to -1.7)	1.2 (-6.6 to 9.2)
High-income	-9.4 (-10.9 to -7.8)	6.5 (4.6 to 8.4)
Latin America and Caribbean	-8.4 (-15.2 to -0.7)	20.1 (10.9 to 30.5)
North Africa and Middle East	-0.5 (-9.6 to 9.4)	36.1 (23.4 to 49.6)
South Asia	2.3 (-9.4 to 15.5)	35.1 (19.4 to 53.0)
Southeast Asia, East Asia, and Oceania	-7.7 (-21.1 to 7.1)	20.6 (2.7 to 40.8)
Sub-Saharan Africa	-4.2 (-12.8 to 5.6)	28.0 (15.4 to 43.1)

GBD = Global Burden of Disease Study; DALY = disability-adjusted life-year; SDI = Socio-demographic Index; UI = uncertainty interval.

Appendix Table 26: Change in age-standardised DALY rates and absolute DALYs from 2010 to 2019 by SDI quintile and GBD super-region, both sexes combined

	Percent change of age-standardised DALY rates (2010 - 2019)			Percent change of DALYs (2010 - 2019)			Absolute change of DALYs (2010 - 2019)		
**Location**	**Behavioural risks (95% UI)**	**Environmental/ occupational risks (95% UI)**	**Metabolic risks (95% UI)**	**Behavioural risks (95% UI)**	**Environmental/ occupational risks (95% UI)**	**Metabolic risks (95% UI)**	**Behavioural risks (95% UI)**	**Environmental/ occupational risks (95% UI)**	**Metabolic risks (95% UI)**
**SDI Quintile**									
High SDI	-12.1 (-13.9 to -10.2)	-14.7 (-18.4 to -10.7)	1.9 (-1.2 to 5.4)	5.0 (2.8 to 7.2)	4.1 (-0.4 to 8.7)	22.4 (18.6 to 26.5)	923 000 (514 000 to 1 350 000)	148 000 (8 580 to 328 000)	1 020 000 (564 000 to 1 630 000)
High-middle SDI	-11.2 (-19.3 to -2.6)	-14.4 (-23.1 to -4.9)	-3.0 (-9.6 to 4.8)	9.8 (-0.2 to 20.5)	6.7 (-4.3 to 18.5)	21.5 (13.0 to 31.1)	2 240 000 (147 000 to 4 590 000)	299 000 (13 200 to 825 000)	986 000 (504 000 to 1 730 000)
Middle SDI	-9.4 (-19.9 to 2.6)	-9.8 (-21.6 to 3.0)	9.2 (-1.3 to 23.2)	19.1 (5.1 to 34.9)	19.4 (3.6 to 36.6)	45.8 (31.1 to 64.4)	4 360 000 (1 150 000 to 7 740 000)	870 000 (164 000 to 1 600 000)	1 750 000 (900 000 to 3 050 000)
Low-middle SDI	-1.5 (-9.1 to 6.7)	-0.7 (-10.8 to 9.3)	28.8 (18.5 to 42.1)	27.5 (17.3 to 38.2)	29.5 (16.2 to 42.8)	70.1 (56.2 to 88.2)	2 540 000 (1 630 000 to 3 480 000)	417 000 (225 000 to 606 000)	836 000 (426 000 to 1 370 000)
Low SDI	-3.0 (-11.3 to 5.8)	0.5 (-11.6 to 12.7)	24.0 (13.4 to 40.0)	29.2 (17.4 to 41.7)	34.5 (17.9 to 51.3)	67.0 (52.1 to 89.1)	984 000 (579 000 to 1 400 000)	147 000 (82 900 to 214 000)	259 000 (133 000 to 419 000)
**GBD super-region**									
South Asia	-1.4 (-12.7 to 11.8)	4.0 (-11.7 to 19.9)	34.4 (17.6 to 55.7)	30.2 (15.4 to 47.6)	38.5 (17.4 to 59.9)	81.3 (58.6 to 110.5)	2 380 000 (1 220 000 to 3 670 000)	402 000 (192 000 to 627 000)	774 000 (364 000 to 1 360 000)
North Africa and Middle East	-3.6 (-12.4 to 6.2)	-7.5 (-20.9 to 8.7)	13.1 (2.6 to 23.8)	32.0 (19.7 to 45.4)	26.8 (8.6 to 48.4)	55.6 (40.7 to 71.4)	777 000 (474 000 to 1 110 000)	146 000 (49 100 to 258 000)	406 000 (228 000 to 664 000)
Sub-Saharan Africa	-6.7 (-15.1 to 3.4)	-6.5 (-16.9 to 5.3)	12.1 (2.8 to 23.5)	25.0 (12.5 to 39.9)	25.3 (10.6 to 42.0)	50.5 (37.1 to 67.2)	838 000 (410 000 to 1 360 000)	106 000 (45 200 to 175 000)	246 000 (139 000 to 400 000)
Southeast Asia, East Asia, and Oceania	-9.2 (-22.8 to 6.7)	-10.6 (-24.1 to 5.0)	6.0 (-7.5 to 25.0)	18.9 (0.7 to 40.5)	17.9 (-0.2 to 38.8)	40.3 (21.8 to 65.5)	5 650 000 (666 000 to 11 500 000)	1 160 000 (98 500 to 2 440 000)	1 750 000 (776 000 to 3 360 000)
Latin America and Caribbean	-12.1 (-18.4 to -5.1)	-12.2 (-20.1 to -2.9)	6.5 (-1.4 to 15.6)	15.0 (6.7 to 24.1)	16.3 (5.7 to 28.7)	42.2 (31.1 to 54.4)	576 000 (257 000 to 943 000)	81 900 (28 500 to 148 000)	410 000 (220 000 to 679 000)
High-income	-11.2 (-12.4 to -9.9)	-14.9 (-18.6 to -11.2)	0.9 (-1.4 to 3.7)	4.1 (2.5 to 5.8)	2.1 (-2.2 to 6.4)	19.3 (16.4 to 22.5)	857 000 (526 000 to 1 210 000)	84 300 (5 180 to 266 000)	996 000 (556 000 to 1 590 000)
Central Europe, Eastern Europe, and Central Asia	-10.3 (-17.3 to -3.1)	-17.5 (-24.9 to -9.8)	1.8 (-5.9 to 10.0)	-0.3 (-8.1 to 7.8)	-7.5 (-15.8 to 1.2)	14.1 (5.7 to 23.2)	23 200 (12 000 to 809 000)	97 200 (6 660 to 213 000)	267 000 (100 000 to 500 000)

UI = uncertainty interval; DALY = disability-adjusted life-year; SDI = Socio-demographic Index; GBD = Global Burden of Disease Study.

Appendix Table 27: Change in age-standardised mortality rates and absolute deaths from 2010 to 2019 by SDI quintile and GBD super-region, both sexes combined

	Percent change of age-standardised mortality rates (2010 - 2019)			Percent change of deaths (2010 - 2019)			Absolute change of deaths (2010 - 2019)		
**Location**	**Behavioural risks (95% UI)**	**Environmental/ occupational risks (95% UI)**	**Metabolic risks (95% UI)**	**Behavioural risks (95% UI)**	**Environmental/ occupational risks (95% UI)**	**Metabolic risks (95% UI)**	**Behavioural risks (95% UI)**	**Environmental/ occupational risks (95% UI)**	**Metabolic risks (95% UI)**
**SDI quintile**									
High SDI	-10.6 (-12.3 to -9.0)	-12.1 (-15.8 to -8.5)	2.2 (-0.6 to 5.5)	9.7 (7.6 to 11.8)	9.2 (4.7 to 13.6)	25.9 (22.3 to 29.8)	84 500 (64 100 to 105 000)	17 500 (8 250 to 27 300)	58 400 (31 100 to 95 900)
High-middle SDI	-9.6 (-17.4 to -1.3)	-12.2 (-20.9 to -2.9)	-3.5 (-9.6 to 3.7)	14.1 (4.1 to 24.7)	11.1 (0.0 to 22.8)	23.3 (15.4 to 32.5)	132 000 (39 200 to 227 000)	21 200 (2 510 to 43 500)	47 200 (24 500 to 82 400)
Middle SDI	-8.6 (-19.0 to 3.4)	-8.7 (-20.3 to 3.8)	8.2 (-1.7 to 21.3)	23.4 (9.0 to 40.0)	23.8 (7.5 to 41.0)	47.9 (33.9 to 66.4)	208 000 (81 200 to 342 000)	43 000 (14 500 to 73 500)	73 100 (36 900 to 128 000)
Low-middle SDI	-0.6 (-7.8 to 7.4)	0.2 (-9.8 to 9.9)	27.6 (18.0 to 40.2)	31.4 (21.7 to 42.3)	33.1 (19.5 to 46.2)	72.5 (58.9 to 89.9)	104 000 (71 800 to 139 000)	17 800 (10 300 to 25 100)	34 200 (17 100 to 56 700)
Low SDI	-2.0 (-9.2 to 5.7)	1.2 (-10.5 to 12.5)	23.0 (13.5 to 37.4)	30.9 (20.3 to 42.3)	35.6 (19.6 to 51.5)	66.9 (53.1 to 87.9)	34 400 (22 200 to 46 700)	5 620 (3 350 to 8 000)	9 890 (4 970 to 16 200)
**GBD super-region**									
South Asia	-0.4 (-11.6 to 12.9)	5.2 (-10.4 to 20.4)	34.0 (18.0 to 54.8)	35.5 (19.9 to 53.6)	43.8 (22.3 to 65.2)	86.3 (63.9 to 115.0)	98 000 (55 600 to 146 000)	17 000 (8 840 to 25 400)	31 800 (14 700 to 55 200)
North Africa and Middle East	-1.9 (-10.6 to 7.5)	-5.7 (-19.0 to 10.9)	14.0 (4.4 to 24.2)	33.1 (21.0 to 46.3)	27.9 (9.7 to 50.6)	55.5 (41.8 to 70.0)	30 600 (19 400 to 43 400)	5 860 (2 060 to 10 400)	16 000 (8 950 to 26 200)
Sub-Saharan Africa	-5.9 (-13.1 to 2.5)	-5.1 (-14.8 to 5.6)	12.2 (4.2 to 22.4)	25.4 (14.6 to 38.3)	25.6 (11.9 to 41.1)	50.0 (37.8 to 65.2)	28 000 (15 800 to 42 900)	4 090 (1 980 to 6 540)	9 650 (5 370 to 15 300)
Southeast Asia, East Asia, and Oceania	-8.4 (-21.4 to 7.0)	-9.4 (-22.5 to 5.8)	4.1 (-9.1 to 22.5)	23.8 (5.4 to 45.3)	22.9 (4.7 to 44.3)	42.1 (24.0 to 66.9)	284 000 (68 700 to 520 000)	60 800 (12 600 to 115 000)	73 800 (32 300 to 142 000)
Latin America and Caribbean	-11.8 (-17.8 to -5.4)	-11.4 (-19.0 to -2.6)	6.8 (-0.8 to 15.3)	18.5 (10.5 to 27.2)	19.9 (9.5 to 32.0)	45.4 (34.7 to 57.4)	27 600 (15 900 to 40 900)	4 170 (1 900 to 6 810)	18 600 (10 100 to 31 100)
High-income	-9.9 (-11.0 to -8.7)	-12.4 (-15.8 to -8.9)	1.2 (-0.9 to 3.9)	8.8 (7.1 to 10.4)	7.2 (3.0 to 11.2)	22.8 (20.1 to 26.0)	86 000 (67 800 to 104 000)	15 600 (6 230 to 25 700)	59 400 (31 300 to 96 400)
Central Europe, Eastern Europe, and Central Asia	-8.8 (-15.9 to -1.6)	-15.6 (-23.2 to -7.9)	3.0 (-4.6 to 11.0)	2.6 (-5.4 to 10.6)	-4.6 (-13.2 to 4.1)	16.8 (8.1 to 25.9)	8 690 (471 to 36 200)	2 390 (100 to 7 020)	13 600 (5 840 to 24 700)

UI = uncertainty interval; SDI = Socio-demographic Index; GBD = Global Burden of Disease Study.

Appendix Table 28: Percentage of cancer deaths, age-standardised mortality rate, DALYs, and age-standardised mortality rate attributable to risks over total cancer deaths and DALYs in 2019, both sexes combined, by country

Location	% of risk-attributable cancer deaths over total cancer deaths (95% UI)	% of risk-attributable cancer age-standardised mortality rate over total cancer age-standardised mortality rate (95% UI)	% of risk-attributable cancer DALYs over total cancer deaths (95% UI)	% of risk-attributable cancer age-standardised DALY rate over total cancer age-standardised DALY rate (95% UI))
**Global**	**44.4 (41.3 to 48.4)**	**44.0 (40.9 to 48.0)**	**42.0 (39.1 to 45.6)**	**41.4 (38.6 to 45.1)**
Afghanistan	21.9 (17.4 to 26.7)	24.8 (19.8 to 30.1)	18.0 (13.8 to 22.2)	23.0 (18.3 to 28.1)
Albania	46.1 (42.7 to 50.1)	44.2 (40.9 to 48.1)	43.2 (40.1 to 46.8)	39.3 (36.2 to 42.7)
Algeria	36.2 (32.4 to 40.3)	36.7 (32.8 to 41.0)	32.1 (28.9 to 35.8)	33.3 (30.0 to 37.2)
American Samoa	44.4 (38.8 to 50.4)	43.2 (37.5 to 49.3)	43.4 (38.1 to 49.3)	43.0 (37.7 to 48.8)
Andorra	47.8 (44.4 to 51.6)	47.8 (44.5 to 51.5)	47.9 (44.8 to 51.5)	46.2 (43.1 to 49.6)
Angola	38.0 (34.5 to 41.7)	37.9 (34.1 to 41.8)	34.6 (31.0 to 38.6)	38.5 (34.9 to 42.3)
Antigua and Barbuda	29.5 (25.6 to 34.6)	28.4 (24.5 to 33.5)	30.1 (26.5 to 35.0)	28.9 (25.4 to 33.7)
Argentina	44.4 (40.8 to 48.8)	44.4 (40.8 to 48.6)	44.1 (40.8 to 47.9)	43.5 (40.4 to 47.3)
Armenia	47.5 (43.8 to 51.9)	46.3 (42.7 to 50.6)	46.3 (42.9 to 50.2)	44.0 (40.7 to 47.7)
Australia	41.5 (38.4 to 45.1)	41.5 (38.5 to 45.0)	41.1 (38.2 to 44.2)	39.8 (37.1 to 42.8)
Austria	44.7 (41.2 to 48.7)	45.5 (42.2 to 49.2)	46.0 (42.9 to 49.6)	44.9 (42.1 to 48.1)
Azerbaijan	44.3 (39.7 to 49.7)	42.8 (38.2 to 48.5)	41.6 (37.5 to 46.6)	40.4 (36.3 to 45.4)
Bahamas	32.3 (28.2 to 37.1)	31.3 (27.3 to 36.1)	32.4 (28.6 to 36.7)	31.5 (27.8 to 35.8)
Bahrain	40.7 (35.3 to 46.7)	44.1 (38.3 to 50.3)	35.7 (30.8 to 41.2)	40.1 (34.6 to 45.9)
Bangladesh	32.1 (28.5 to 36.1)	32.5 (28.9 to 36.6)	28.4 (25.0 to 32.3)	29.4 (26.0 to 33.4)
Barbados	30.8 (26.6 to 35.8)	30.4 (26.4 to 35.3)	31.6 (27.7 to 36.2)	30.5 (26.8 to 34.9)
Belarus	46.4 (43.5 to 50.0)	45.6 (42.7 to 49.1)	46.6 (43.7 to 50.1)	44.3 (41.5 to 47.6)
Belgium	48.6 (45.0 to 52.4)	49.3 (45.9 to 52.8)	49.5 (46.3 to 52.9)	48.2 (45.4 to 51.3)
Belize	37.4 (33.8 to 41.5)	36.8 (33.1 to 41.1)	37.2 (34.0 to 40.7)	37.6 (34.3 to 41.4)
Benin	32.5 (29.1 to 37.0)	33.5 (29.6 to 38.3)	28.9 (25.7 to 32.9)	33.8 (30.4 to 38.2)
Bermuda	38.2 (34.2 to 42.5)	37.8 (34.0 to 42.0)	38.5 (34.9 to 42.5)	36.5 (33.2 to 40.2)
Bhutan	33.5 (29.6 to 38.1)	33.8 (29.9 to 38.5)	30.6 (27.0 to 35.1)	31.8 (28.2 to 36.3)
Bolivia (Plurinational State of)	30.6 (25.7 to 37.1)	30.1 (25.3 to 36.7)	29.6 (25.1 to 35.5)	30.3 (25.6 to 36.4)
Bosnia and Herzegovina	52.7 (48.8 to 57.1)	51.3 (47.5 to 55.6)	52.7 (49.1 to 56.7)	49.9 (46.6 to 53.7)

Location	% of risk-attributable cancer deaths over total cancer deaths (95% UI)	% of risk-attributable cancer age-standardised mortality rate over total cancer age-standardised mortality rate (95% UI)	% of risk-attributable cancer DALYs over total cancer deaths (95% UI)	% of risk-attributable cancer age-standardised DALY rate over total cancer age-standardised DALY rate (95% UI))
Botswana	45.6 (42.1 to 49.6)	45.1 (41.3 to 49.5)	44.0 (40.8 to 47.9)	45.1 (41.6 to 49.0)
Brazil	39.5 (36.4 to 43.6)	39.0 (35.9 to 43.1)	38.2 (35.5 to 41.9)	37.4 (34.7 to 41.0)
Brunei	39.2 (34.5 to 44.4)	41.2 (36.2 to 47.1)	34.8 (30.6 to 39.6)	37.6 (33.1 to 42.8)
Bulgaria	50.3 (46.8 to 54.4)	50.1 (46.7 to 53.8)	51.8 (48.6 to 55.5)	49.9 (46.8 to 53.2)
Burkina Faso	32.9 (29.3 to 37.5)	33.4 (29.5 to 38.3)	29.4 (25.3 to 33.7)	34.2 (30.6 to 38.8)
Burundi	33.3 (29.6 to 37.1)	33.5 (30.1 to 37.5)	29.9 (25.6 to 33.8)	34.1 (30.4 to 38.1)
Cape Verde	31.0 (26.7 to 36.9)	31.2 (26.8 to 37.3)	31.5 (27.5 to 36.8)	32.0 (27.8 to 37.5)
Cambodia	44.6 (41.2 to 48.6)	45.7 (42.1 to 49.8)	39.7 (36.5 to 43.6)	41.8 (38.6 to 45.8)
Cameroon	34.5 (29.9 to 39.1)	34.4 (29.6 to 39.4)	32.1 (28.1 to 36.1)	35.1 (30.5 to 39.7)
Canada	46.8 (43.7 to 50.3)	46.4 (43.3 to 49.7)	45.5 (42.5 to 48.6)	43.4 (40.5 to 46.4)
Central African Republic	37.8 (32.6 to 42.7)	36.9 (32.3 to 41.7)	35.5 (29.4 to 40.7)	37.8 (32.6 to 42.7)
Chad	32.7 (28.8 to 37.1)	33.8 (29.3 to 38.6)	29.0 (25.3 to 32.9)	34.7 (30.5 to 39.2)
Chile	37.7 (33.3 to 43.3)	37.3 (33.0 to 42.8)	38.1 (34.1 to 43.3)	37.0 (33.1 to 42.0)
China	50.9 (47.0 to 55.6)	49.8 (46.0 to 54.4)	48.6 (44.9 to 53.3)	46.4 (42.8 to 50.8)
Colombia	31.2 (27.0 to 37.1)	31.1 (27.0 to 37.0)	29.7 (26.0 to 35.0)	29.2 (25.6 to 34.3)
Comoros	32.8 (28.8 to 37.4)	32.7 (28.7 to 37.2)	31.6 (27.4 to 36.1)	32.6 (28.5 to 37.2)
Congo (Brazzaville)	39.1 (35.0 to 43.2)	37.9 (34.0 to 42.2)	38.0 (33.6 to 42.1)	38.8 (34.8 to 43.0)
Cook Islands	43.2 (37.9 to 49.0)	42.2 (37.0 to 47.9)	44.2 (38.9 to 50.0)	42.7 (37.5 to 48.4)
Costa Rica	31.4 (26.9 to 37.6)	31.4 (26.8 to 37.5)	30.3 (26.1 to 35.9)	29.9 (25.7 to 35.4)
Côte d'Ivoire	35.1 (31.4 to 39.4)	34.7 (30.6 to 39.7)	32.5 (29.3 to 36.5)	35.4 (31.7 to 39.7)
Croatia	49.3 (45.5 to 53.4)	49.2 (45.6 to 53.1)	50.4 (47.0 to 54.2)	48.5 (45.3 to 52.1)
Cuba	46.8 (43.2 to 51.1)	46.5 (43.0 to 50.8)	47.3 (43.9 to 51.3)	45.3 (42.1 to 49.4)
Cyprus	44.5 (40.8 to 48.9)	43.1 (39.3 to 47.6)	43.5 (40.0 to 47.4)	41.7 (38.4 to 45.5)
Czechia	50.3 (46.1 to 55.3)	49.7 (45.6 to 54.5)	50.5 (46.6 to 54.9)	48.5 (44.8 to 52.7)
Denmark	49.1 (46.3 to 52.4)	48.5 (45.7 to 51.7)	48.3 (45.4 to 51.5)	46.1 (43.3 to 49.2)
Djibouti	35.0 (31.0 to 40.1)	35.2 (31.2 to 39.9)	31.6 (27.2 to 36.9)	34.2 (30.1 to 39.2)
Dominica	28.1 (24.1 to 32.9)	28.0 (24.2 to 32.8)	29.4 (25.8 to 33.9)	28.7 (25.2 to 33.1)
Dominican Republic	37.9 (34.5 to 41.8)	37.8 (34.3 to 41.7)	36.4 (33.3 to 39.9)	36.8 (33.6 to 40.4)

Location	% of risk-attributable cancer deaths over total cancer deaths (95% UI)	% of risk-attributable cancer age-standardised mortality rate over total cancer age-standardised mortality rate (95% UI)	% of risk-attributable cancer DALYs over total cancer deaths (95% UI)	% of risk-attributable cancer age-standardised DALY rate over total cancer age-standardised DALY rate (95% UI))
DR Congo	34.4 (29.5 to 40.1)	33.9 (29.2 to 39.8)	32.4 (27.6 to 38.1)	34.8 (30.0 to 40.6)
Ecuador	29.7 (25.6 to 35.4)	29.5 (25.4 to 35.3)	28.1 (24.4 to 33.2)	28.6 (24.8 to 33.8)
Egypt	38.5 (33.9 to 43.2)	39.0 (34.4 to 43.9)	34.8 (30.6 to 39.2)	36.7 (32.3 to 41.3)
El Salvador	31.1 (26.7 to 37.3)	31.6 (27.2 to 37.8)	31.1 (27.1 to 36.7)	31.6 (27.5 to 37.2)
Equatorial Guinea	37.1 (33.2 to 41.4)	37.4 (33.1 to 42.0)	34.7 (30.9 to 38.6)	38.2 (34.1 to 42.3)
Eritrea	33.1 (29.1 to 37.0)	32.8 (29.0 to 36.9)	30.6 (26.6 to 34.5)	33.2 (29.2 to 37.1)
Estonia	44.2 (40.8 to 48.0)	44.6 (41.4 to 48.1)	45.0 (41.9 to 48.5)	43.7 (40.8 to 46.9)
Eswatini	42.1 (37.4 to 47.5)	42.4 (37.4 to 48.1)	39.7 (35.4 to 45.1)	41.9 (37.4 to 47.3)
Ethiopia	24.8 (21.3 to 30.0)	26.8 (23.2 to 31.7)	20.4 (16.8 to 26.2)	25.8 (22.3 to 31.2)
Federated States of Micronesia	47.7 (41.5 to 53.9)	46.1 (40.0 to 52.6)	45.3 (39.1 to 51.5)	45.6 (39.5 to 51.8)
Fiji	45.2 (39.2 to 51.6)	44.1 (37.9 to 50.7)	42.2 (36.5 to 47.9)	42.2 (36.3 to 48.1)
Finland	40.2 (36.3 to 44.7)	39.9 (36.2 to 44.3)	40.1 (36.5 to 44.3)	37.9 (34.5 to 41.7)
France	45.7 (42.8 to 48.8)	47.2 (44.5 to 50.0)	47.6 (45.0 to 50.4)	46.8 (44.4 to 49.4)
Gabon	38.1 (34.1 to 42.7)	37.4 (33.3 to 42.3)	37.1 (33.3 to 41.2)	37.9 (34.1 to 42.3)
The Gambia	36.7 (32.1 to 41.7)	37.8 (33.3 to 43.0)	33.9 (29.5 to 39.0)	37.6 (32.8 to 42.9)
Georgia	44.7 (40.9 to 49.3)	44.1 (40.4 to 48.5)	44.5 (40.9 to 48.7)	42.4 (38.9 to 46.3)
Germany	47.1 (43.2 to 51.5)	47.7 (44.1 to 51.8)	48.0 (44.7 to 52.1)	46.6 (43.5 to 50.3)
Ghana	29.6 (25.7 to 34.0)	29.7 (25.8 to 34.4)	27.8 (24.0 to 31.6)	29.5 (25.7 to 33.7)
Greece	48.2 (44.9 to 51.9)	49.3 (46.3 to 52.8)	49.4 (46.4 to 52.9)	47.8 (45.0 to 51.0)
Greenland	60.2 (56.9 to 63.5)	59.1 (55.7 to 62.5)	58.6 (55.5 to 61.9)	57.3 (54.2 to 60.7)
Grenada	33.1 (29.0 to 38.1)	32.2 (28.0 to 37.2)	33.6 (29.8 to 38.2)	32.7 (28.9 to 37.2)
Guam	45.8 (40.9 to 51.1)	45.3 (40.4 to 50.5)	44.7 (40.0 to 49.7)	43.7 (39.1 to 48.7)
Guatemala	29.2 (24.8 to 36.1)	29.3 (24.8 to 36.3)	28.0 (24.3 to 34.0)	29.7 (25.5 to 36.2)
Guinea	35.4 (31.5 to 40.1)	35.6 (31.4 to 40.6)	33.8 (30.3 to 38.0)	36.9 (33.1 to 41.4)
Guinea-Bissau	33.6 (29.0 to 38.9)	32.8 (28.0 to 38.2)	32.4 (27.7 to 37.6)	34.2 (29.4 to 39.4)
Guyana	34.5 (30.3 to 39.7)	33.0 (28.7 to 38.1)	34.6 (30.8 to 39.2)	34.1 (30.2 to 38.8)
Haiti	30.7 (26.3 to 35.2)	29.5 (25.2 to 34.4)	29.3 (24.7 to 34.4)	30.4 (25.9 to 35.0)
Honduras	36.4 (31.3 to 42.2)	36.5 (31.3 to 42.3)	33.9 (29.0 to 39.5)	35.7 (30.6 to 41.4)

Location	% of risk-attributable cancer deaths over total cancer deaths (95% UI)	% of risk-attributable cancer age-standardised mortality rate over total cancer age-standardised mortality rate (95% UI)	% of risk-attributable cancer DALYs over total cancer deaths (95% UI)	% of risk-attributable cancer age-standardised DALY rate over total cancer age-standardised DALY rate (95% UI))
Hungary	53.5 (49.9 to 57.7)	53.6 (50.2 to 57.6)	55.2 (52.0 to 58.9)	53.9 (50.8 to 57.3)
Iceland	43.1 (39.7 to 46.8)	43.1 (40.0 to 46.7)	42.5 (39.5 to 45.7)	41.0 (38.1 to 44.1)
India	37.3 (34.1 to 41.2)	37.4 (34.3 to 41.3)	34.7 (31.8 to 38.3)	35.4 (32.4 to 39.1)
Indonesia	39.5 (35.8 to 43.8)	39.8 (36.1 to 44.0)	36.0 (32.5 to 40.0)	36.7 (33.1 to 40.7)
Iran	31.9 (28.5 to 36.5)	32.3 (28.8 to 36.9)	28.9 (25.7 to 33.1)	29.6 (26.3 to 33.9)
Iraq	37.8 (34.2 to 41.9)	41.1 (37.2 to 45.5)	31.2 (28.1 to 34.9)	36.2 (32.7 to 40.3)
Ireland	45.8 (42.4 to 49.5)	45.4 (42.1 to 49.1)	44.5 (41.4 to 48.0)	43.3 (40.3 to 46.6)
Israel	38.5 (34.5 to 42.9)	38.4 (34.6 to 42.8)	37.3 (33.9 to 41.2)	36.6 (33.4 to 40.5)
Italy	45.2 (41.5 to 49.6)	45.3 (41.9 to 49.5)	45.2 (41.8 to 49.2)	43.0 (40.0 to 46.7)
Jamaica	35.7 (31.6 to 40.8)	36.0 (31.9 to 41.2)	36.1 (32.4 to 40.6)	36.0 (32.3 to 40.4)
Japan	41.0 (37.5 to 45.7)	41.8 (38.5 to 46.2)	42.0 (38.7 to 46.3)	40.6 (37.6 to 44.7)
Jordan	40.9 (36.7 to 45.2)	43.0 (38.8 to 47.4)	35.1 (31.2 to 39.1)	39.3 (35.2 to 43.6)
Kazakhstan	46.8 (42.9 to 51.6)	46.0 (42.2 to 51.0)	44.6 (41.0 to 48.9)	44.2 (40.6 to 48.6)
Kenya	31.2 (27.3 to 35.3)	31.2 (27.4 to 35.4)	29.0 (25.3 to 33.3)	31.4 (27.5 to 35.6)
Kiribati	55.7 (51.6 to 60.4)	56.4 (52.4 to 61.1)	51.8 (47.6 to 56.7)	54.3 (50.3 to 59.0)
Kuwait	39.1 (34.5 to 44.0)	41.8 (36.9 to 47.2)	34.1 (30.1 to 38.4)	38.1 (33.6 to 43.0)
Kyrgyzstan	40.5 (37.2 to 45.3)	40.6 (37.2 to 45.5)	38.1 (35.0 to 42.6)	39.3 (36.0 to 43.9)
Laos	43.8 (40.1 to 48.2)	45.3 (41.5 to 49.9)	39.0 (35.6 to 42.9)	42.1 (38.5 to 46.3)
Latvia	43.5 (39.5 to 47.9)	43.7 (39.9 to 47.9)	44.4 (40.7 to 48.7)	42.6 (39.1 to 46.6)
Lebanon	45.2 (41.0 to 50.3)	45.2 (41.0 to 50.3)	41.3 (37.4 to 45.8)	41.4 (37.6 to 45.9)
Lesotho	46.7 (42.0 to 51.5)	46.0 (41.2 to 50.8)	45.7 (41.0 to 50.9)	46.6 (41.8 to 51.6)
Liberia	33.7 (29.8 to 38.4)	32.8 (28.7 to 37.8)	32.7 (28.9 to 37.0)	34.4 (30.4 to 39.1)
Libya	40.6 (36.3 to 45.4)	42.2 (37.7 to 47.2)	36.7 (32.7 to 41.0)	39.0 (34.8 to 43.6)
Lithuania	42.7 (39.2 to 46.7)	42.8 (39.5 to 46.7)	43.4 (40.2 to 47.1)	41.8 (38.8 to 45.3)
Luxembourg	47.5 (43.4 to 51.8)	47.5 (43.5 to 51.7)	46.6 (42.8 to 50.5)	45.1 (41.5 to 48.7)
Madagascar	33.9 (29.9 to 37.9)	33.1 (29.6 to 37.0)	31.7 (27.0 to 35.7)	34.0 (30.1 to 38.0)
Malawi	33.4 (29.4 to 37.9)	36.1 (32.0 to 40.7)	27.7 (23.4 to 32.3)	34.4 (30.3 to 39.2)
Malaysia	41.7 (37.7 to 46.6)	42.2 (37.9 to 47.1)	38.4 (34.7 to 42.7)	39.2 (35.3 to 43.6)

Location	% of risk-attributable cancer deaths over total cancer deaths (95% UI)	% of risk-attributable cancer age-standardised mortality rate over total cancer age-standardised mortality rate (95% UI)	% of risk-attributable cancer DALYs over total cancer deaths (95% UI)	% of risk-attributable cancer age-standardised DALY rate over total cancer age-standardised DALY rate (95% UI))
Maldives	38.1 (34.7 to 41.9)	40.3 (36.6 to 44.3)	32.8 (29.5 to 36.2)	36.1 (32.6 to 39.7)
Mali	30.5 (26.8 to 35.9)	31.4 (27.5 to 37.2)	27.6 (23.9 to 32.6)	31.4 (27.6 to 36.8)
Malta	44.9 (41.1 to 49.8)	44.0 (40.3 to 48.6)	44.5 (41.0 to 48.8)	41.3 (38.2 to 45.2)
Marshall Islands	43.3 (36.3 to 50.5)	42.3 (35.5 to 49.7)	40.8 (33.8 to 47.7)	41.8 (35.0 to 48.9)
Mauritania	32.4 (28.8 to 36.7)	31.9 (28.3 to 36.4)	31.8 (28.5 to 35.5)	33.3 (29.9 to 37.3)
Mauritius	41.4 (36.0 to 48.0)	40.4 (35.1 to 46.8)	39.1 (33.9 to 45.2)	36.9 (32.1 to 42.6)
Mexico	33.6 (29.6 to 39.2)	33.6 (29.5 to 39.2)	31.1 (27.3 to 36.0)	31.2 (27.4 to 36.1)
Moldova	46.7 (43.5 to 50.6)	45.6 (42.4 to 49.4)	46.7 (43.6 to 50.3)	44.1 (41.1 to 47.5)
Monaco	46.3 (41.8 to 50.6)	46.4 (42.1 to 50.4)	47.0 (42.7 to 50.9)	44.5 (40.6 to 48.0)
Mongolia	48.3 (43.2 to 54.5)	47.9 (43.0 to 54.2)	46.5 (41.2 to 52.6)	47.4 (42.4 to 53.4)
Montenegro	57.6 (54.3 to 61.3)	56.1 (52.8 to 59.7)	57.3 (54.2 to 60.6)	54.8 (51.7 to 57.9)
Morocco	38.3 (34.4 to 42.6)	38.0 (34.1 to 42.3)	35.9 (32.1 to 40.0)	36.1 (32.3 to 40.2)
Mozambique	33.9 (30.1 to 37.7)	36.1 (32.5 to 40.3)	28.6 (24.5 to 32.5)	35.3 (31.5 to 39.1)
Myanmar	39.5 (35.0 to 44.1)	39.9 (35.2 to 44.6)	35.5 (31.4 to 39.8)	36.2 (32.1 to 40.6)
Namibia	35.9 (33.0 to 39.2)	36.5 (33.5 to 40.0)	33.7 (31.0 to 36.9)	35.5 (32.7 to 38.8)
Nauru	44.3 (38.5 to 49.9)	44.9 (38.9 to 50.8)	40.0 (34.4 to 45.2)	44.1 (38.2 to 49.7)
Nepal	33.3 (30.0 to 37.2)	33.9 (30.6 to 37.8)	29.8 (26.8 to 33.4)	31.3 (28.2 to 34.9)
Netherlands	48.4 (45.2 to 51.8)	47.9 (44.8 to 51.1)	47.8 (44.9 to 51.1)	45.7 (42.8 to 48.8)
New Zealand	42.0 (38.9 to 45.4)	41.5 (38.5 to 44.8)	40.8 (37.9 to 44.0)	38.9 (36.2 to 42.0)
Nicaragua	32.8 (28.6 to 38.4)	32.6 (28.3 to 38.3)	31.1 (27.2 to 36.1)	32.0 (28.0 to 37.4)
Niger	30.8 (27.0 to 35.8)	31.1 (27.0 to 36.7)	27.2 (23.5 to 31.8)	32.7 (28.6 to 37.9)
Nigeria	23.9 (20.4 to 28.9)	23.8 (19.8 to 29.5)	21.9 (18.9 to 25.6)	24.5 (21.0 to 29.5)
Niue	45.4 (39.3 to 52.2)	44.6 (38.6 to 51.4)	44.8 (39.0 to 51.1)	43.2 (37.6 to 49.3)
North Korea	44.3 (40.2 to 49.5)	43.6 (39.6 to 48.8)	42.5 (38.4 to 47.6)	41.3 (37.3 to 46.3)
North Macedonia	51.9 (48.0 to 56.4)	49.9 (46.0 to 54.4)	51.4 (47.9 to 55.5)	48.9 (45.5 to 52.9)
Northern Mariana Islands	50.1 (45.2 to 55.3)	47.6 (42.5 to 53.0)	49.2 (44.2 to 54.2)	46.6 (41.9 to 51.5)
Norway	38.4 (34.7 to 42.6)	38.5 (35.0 to 42.6)	38.5 (35.2 to 42.4)	37.3 (34.1 to 40.9)
Oman	28.9 (24.7 to 33.8)	31.1 (26.7 to 36.5)	24.8 (21.2 to 29.1)	29.2 (24.9 to 34.1)

Location	% of risk-attributable cancer deaths over total cancer deaths (95% UI)	% of risk-attributable cancer age-standardised mortality rate over total cancer age-standardised mortality rate (95% UI)	% of risk-attributable cancer DALYs over total cancer deaths (95% UI)	% of risk-attributable cancer age-standardised DALY rate over total cancer age-standardised DALY rate (95% UI))
Pakistan	32.0 (27.9 to 36.3)	34.8 (30.6 to 39.1)	26.4 (22.7 to 30.2)	31.2 (27.2 to 35.4)
Palau	47.4 (42.3 to 53.1)	44.8 (39.7 to 50.6)	47.1 (42.3 to 52.4)	44.8 (40.0 to 50.2)
Palestine	38.9 (35.2 to 43.2)	41.2 (36.9 to 46.0)	33.5 (30.2 to 37.2)	38.2 (34.5 to 42.4)
Panama	31.6 (27.6 to 36.7)	31.6 (27.7 to 36.8)	30.2 (26.6 to 34.8)	30.2 (26.6 to 34.8)
Papua New Guinea	37.1 (31.5 to 43.2)	38.2 (32.5 to 44.6)	32.9 (27.9 to 38.7)	36.6 (31.1 to 42.7)
Paraguay	43.0 (39.5 to 46.9)	43.3 (39.6 to 47.2)	40.9 (37.8 to 44.5)	41.8 (38.5 to 45.4)
Peru	26.6 (22.5 to 32.6)	26.7 (22.6 to 32.7)	26.0 (22.3 to 31.3)	26.1 (22.3 to 31.4)
Philippines	41.6 (38.0 to 45.4)	42.4 (38.7 to 46.2)	37.3 (34.0 to 40.7)	39.2 (35.9 to 42.8)
Poland	51.3 (47.6 to 55.5)	50.7 (47.2 to 54.8)	52.0 (48.7 to 55.8)	50.0 (46.8 to 53.7)
Portugal	40.3 (36.2 to 45.3)	41.5 (37.7 to 46.3)	42.6 (39.0 to 47.1)	41.9 (38.6 to 46.1)
Puerto Rico	36.2 (31.4 to 42.1)	36.3 (31.7 to 41.8)	36.6 (32.1 to 42.1)	35.1 (31.0 to 40.0)
Qatar	39.5 (34.0 to 45.3)	42.0 (36.3 to 48.4)	34.2 (29.4 to 39.4)	40.7 (35.1 to 46.6)
Romania	48.6 (45.5 to 52.3)	48.6 (45.6 to 52.0)	49.8 (46.9 to 53.1)	48.2 (45.4 to 51.4)
Russia	46.0 (42.4 to 50.2)	45.2 (41.6 to 49.3)	46.2 (42.8 to 50.1)	44.3 (41.0 to 48.0)
Rwanda	37.1 (33.3 to 41.4)	38.1 (34.3 to 42.5)	32.9 (29.0 to 37.7)	36.8 (33.0 to 41.2)
Saint Kitts and Nevis	31.4 (27.0 to 36.3)	29.7 (25.3 to 34.6)	31.8 (27.6 to 36.5)	30.1 (26.0 to 34.7)
Saint Lucia	32.7 (28.4 to 37.8)	32.0 (27.8 to 37.1)	34.0 (30.2 to 38.8)	32.9 (29.2 to 37.5)
Saint Vincent and the Grenadines	31.2 (27.5 to 36.2)	30.4 (26.6 to 35.3)	32.9 (29.3 to 37.4)	31.9 (28.5 to 36.4)
Samoa	41.3 (36.7 to 46.6)	41.5 (36.8 to 47.0)	38.8 (34.5 to 43.6)	40.3 (35.9 to 45.2)
San Marino	38.7 (34.6 to 43.4)	39.2 (35.2 to 43.7)	39.0 (35.2 to 43.4)	37.4 (33.7 to 41.5)
São Tomé and Príncipe	34.2 (30.4 to 39.0)	34.4 (30.2 to 39.6)	31.6 (28.1 to 35.9)	34.0 (30.4 to 38.6)
Saudi Arabia	33.2 (29.0 to 37.6)	35.7 (30.9 to 40.9)	29.6 (26.0 to 33.6)	33.6 (29.2 to 38.2)
Senegal	32.2 (28.6 to 36.7)	32.1 (28.1 to 37.0)	30.4 (27.0 to 34.5)	32.7 (29.2 to 37.1)
Serbia	52.8 (49.0 to 57.0)	51.7 (48.1 to 55.7)	53.6 (50.1 to 57.3)	51.8 (48.5 to 55.3)
Seychelles	43.8 (39.5 to 48.5)	43.1 (38.7 to 48.0)	42.9 (39.0 to 47.1)	41.9 (38.0 to 46.0)
Sierra Leone	33.9 (30.7 to 37.9)	34.5 (31.1 to 38.8)	30.7 (27.3 to 34.7)	34.9 (31.7 to 39.0)
Singapore	39.5 (35.6 to 44.3)	39.1 (35.1 to 43.9)	37.7 (34.0 to 42.1)	36.4 (32.9 to 40.8)

Location	% of risk-attributable cancer deaths over total cancer deaths (95% UI)	% of risk-attributable cancer age-standardised mortality rate over total cancer age-standardised mortality rate (95% UI)	% of risk-attributable cancer DALYs over total cancer deaths (95% UI)	% of risk-attributable cancer age-standardised DALY rate over total cancer age-standardised DALY rate (95% UI))
Slovakia	47.8 (44.0 to 52.2)	47.0 (43.3 to 51.3)	48.3 (44.8 to 52.2)	46.5 (43.2 to 50.4)
Slovenia	45.5 (41.5 to 50.0)	46.2 (42.4 to 50.5)	47.5 (43.9 to 51.5)	46.4 (43.0 to 50.2)
Solomon Islands	40.5 (33.2 to 46.9)	42.2 (35.5 to 48.8)	36.6 (28.8 to 42.8)	40.2 (33.0 to 46.7)
Somalia	33.5 (28.6 to 38.5)	35.0 (30.5 to 40.1)	29.2 (23.9 to 34.3)	35.1 (30.3 to 40.1)
South Africa	45.5 (42.3 to 49.0)	44.8 (41.5 to 48.6)	44.9 (41.9 to 48.2)	45.1 (42.1 to 48.5)
South Korea	45.8 (42.0 to 50.8)	45.0 (41.2 to 50.0)	44.3 (40.5 to 49.2)	42.4 (38.7 to 47.2)
South Sudan	30.6 (26.4 to 35.4)	31.8 (27.5 to 36.4)	26.2 (21.9 to 31.9)	31.2 (27.0 to 36.1)
Spain	46.8 (43.1 to 51.2)	48.3 (44.8 to 52.3)	48.9 (45.6 to 52.5)	47.9 (44.9 to 51.3)
Sri Lanka	37.5 (33.2 to 42.6)	36.9 (32.5 to 42.0)	35.0 (31.1 to 39.5)	33.9 (30.1 to 38.3)
Sudan	29.3 (25.4 to 34.0)	32.0 (27.7 to 37.1)	23.7 (20.3 to 27.7)	29.0 (25.1 to 33.7)
Suriname	39.8 (35.7 to 44.5)	39.1 (34.9 to 43.7)	39.2 (35.3 to 43.5)	38.3 (34.4 to 42.6)
Sweden	40.4 (37.0 to 44.1)	40.2 (37.0 to 43.8)	39.9 (36.8 to 43.5)	38.0 (35.0 to 41.3)
Switzerland	45.1 (41.8 to 48.8)	45.5 (42.4 to 49.0)	45.2 (42.2 to 48.5)	43.6 (40.8 to 46.8)
Syria	36.3 (32.5 to 40.6)	36.2 (32.0 to 40.5)	32.2 (28.4 to 36.1)	32.7 (28.8 to 36.6)
Taiwan (Province of China)	46.2 (42.2 to 50.9)	45.6 (41.7 to 50.2)	46.6 (43.0 to 50.8)	44.8 (41.4 to 48.9)
Tajikistan	30.5 (26.6 to 36.6)	32.0 (27.8 to 38.4)	26.7 (23.1 to 32.2)	29.7 (25.8 to 35.6)
Tanzania	35.1 (31.6 to 39.5)	36.9 (33.1 to 41.0)	30.1 (26.0 to 34.5)	36.1 (32.4 to 40.6)
Thailand	47.2 (43.4 to 51.5)	46.4 (42.7 to 50.7)	45.3 (41.5 to 49.5)	43.2 (39.6 to 47.2)
Timor-Leste	39.2 (35.3 to 43.8)	39.9 (36.0 to 44.6)	34.6 (30.9 to 39.5)	37.5 (33.8 to 42.1)
Togo	35.7 (32.1 to 39.7)	35.2 (31.6 to 39.6)	33.6 (30.0 to 37.3)	35.6 (32.0 to 39.6)
Tokelau	44.3 (38.7 to 50.5)	43.5 (37.9 to 49.6)	43.4 (38.3 to 49.2)	43.0 (37.9 to 48.7)
Tonga	44.2 (39.7 to 49.5)	44.7 (40.1 to 49.9)	42.2 (37.6 to 47.2)	43.4 (38.9 to 48.5)
Trinidad and Tobago	36.5 (32.1 to 41.8)	35.4 (31.1 to 40.5)	36.7 (32.7 to 41.5)	34.8 (31.0 to 39.4)
Tunisia	47.6 (43.4 to 52.0)	47.2 (42.9 to 51.7)	44.5 (40.6 to 48.7)	44.0 (40.0 to 48.1)
Turkey	47.9 (44.6 to 51.7)	47.3 (43.9 to 51.1)	46.2 (43.0 to 49.8)	45.2 (42.0 to 48.8)
Turkmenistan	39.7 (36.1 to 43.9)	39.8 (36.0 to 44.3)	35.9 (32.6 to 39.7)	36.6 (33.2 to 40.5)
Tuvalu	43.8 (38.5 to 49.7)	42.7 (37.5 to 48.8)	42.1 (36.9 to 47.7)	42.0 (36.9 to 47.7)
Uganda	32.7 (29.5 to 36.1)	33.5 (30.1 to 37.2)	29.5 (26.1 to 32.9)	34.1 (31.0 to 37.6)

Location	% of risk-attributable cancer deaths over total cancer deaths (95% UI)	% of risk-attributable cancer age-standardised mortality rate over total cancer age-standardised mortality rate (95% UI)	% of risk-attributable cancer DALYs over total cancer deaths (95% UI)	% of risk-attributable cancer age-standardised DALY rate over total cancer age-standardised DALY rate (95% UI))
Ukraine	45.4 (41.9 to 49.2)	44.1 (40.8 to 47.9)	44.7 (41.3 to 48.3)	41.8 (38.6 to 45.3)
United Arab Emirates	36.0 (30.9 to 41.3)	42.2 (34.9 to 49.6)	32.9 (28.3 to 37.6)	39.2 (32.9 to 45.5)
UK	49.7 (46.2 to 53.7)	49.4 (46.0 to 53.3)	49.0 (45.7 to 52.6)	47.2 (44.0 to 50.7)
Uruguay	42.6 (39.1 to 46.7)	43.4 (40.1 to 47.2)	44.0 (40.9 to 47.5)	43.5 (40.6 to 46.8)
USA	49.5 (45.9 to 53.4)	49.1 (45.6 to 52.9)	48.6 (45.1 to 52.2)	46.7 (43.5 to 50.3)
Uzbekistan	35.7 (32.3 to 40.3)	35.6 (31.9 to 40.5)	32.1 (29.1 to 36.1)	33.5 (30.3 to 37.8)
Vanuatu	40.3 (35.1 to 46.0)	40.2 (35.0 to 46.2)	37.3 (32.4 to 42.6)	39.4 (34.3 to 44.9)
Venezuela	36.4 (32.2 to 41.2)	35.8 (31.7 to 40.7)	36.0 (32.2 to 40.4)	35.4 (31.6 to 39.8)
Vietnam	46.5 (43.1 to 50.6)	46.2 (42.7 to 50.3)	44.7 (41.5 to 48.3)	44.3 (41.1 to 48.0)
Virgin Islands	34.4 (29.5 to 39.7)	33.5 (28.7 to 38.6)	34.6 (29.6 to 39.5)	33.3 (28.5 to 38.2)
Yemen	31.5 (28.3 to 35.4)	34.0 (30.5 to 38.2)	26.3 (23.3 to 29.9)	31.6 (28.5 to 35.5)
Zambia	36.1 (32.7 to 39.5)	37.3 (33.9 to 41.2)	32.1 (28.3 to 35.6)	37.1 (33.6 to 40.6)
Zimbabwe	40.3 (36.6 to 44.4)	40.0 (36.0 to 44.1)	39.0 (35.4 to 43.0)	40.5 (36.7 to 44.7)

All numbers in this table represent the percentage of all risk-attributable cancer deaths or DALYs out of the total (risk + non-risk-attributable) deaths or DALYs of all 29 cancer types. DALY = disability-adjusted life-year; UI = uncertainty interval.

Appendix Table 29: Attributable cancer deaths and DALYs in 2019 and percentage change of age-standardised death rates and DALY rates, 2010-2019 for all regions, countries, and territories

Location	All risk factors*				Environmental and occupational risks*				Behavioural risks*				Metabolic risks*			
	Death ASR	Death % change	DALY ASR	DALY % change	Death ASR	Death % change	DALY ASR	DALY % change	Death ASR	Death % change	DALY ASR	DALY % change	Death ASR	Death % change	DALY ASR	DALY % change
**Global**	**54.9 (49.3 to 61.0)**	**-6.9 (-12.8 to -0.9)**	**1262.7 (1142.8 to 1398.7)**	**-7.8 (-14.0 to -1.4)**	**9.1 (7.7 to 10.6)**	**-10.0 (-16.7 to -2.8)**	**196.1 (165.5 to 230.3)**	**-11.4 (-18.5 to -3.5)**	**45.5 (42.1 to 49.4)**	**-8.7 (-14.5 to -2.7)**	**1054.7 (974.1 to 1145.3)**	**-9.6 (-15.8 to -3.2)**	**10.7 (5.5 to 17.5)**	**2.8 (-2.2 to 8.8)**	**234.0 (124.0 to 376.0)**	**3.8 (-2.0 to 10.5)**
**Low SDI**	**33.9 (29.4 to 39.0)**	**0.8 (-6.6 to 8.8)**	**865.4 (749.5 to 995.3)**	**-0.3 (-8.7 to 8.7)**	**4.4 (3.4 to 5.6)**	**1.2 (-10.5 to 12.5)**	**102.9 (80.7 to 131.8)**	**0.5 (-11.6 to 12.7)**	**28.0 (24.7 to 31.7)**	**-2.0 (-9.2 to 5.7)**	**726.2 (636.4 to 827.5)**	**-3.0 (-11.3 to 5.8)**	**5.1 (2.4 to 8.7)**	**23.0 (13.5 to 37.4)**	**118.9 (56.7 to 198.0)**	**24.0 (13.4 to 40.0)**
**Low-middle SDI**	**38.7 (34.3 to 44.0)**	**2.5 (-5.1 to 10.4)**	**958.6 (850.4 to 1084.6)**	**1.5 (-6.4 to 9.9)**	**5.4 (4.5 to 6.4)**	**0.2 (-9.8 to 9.9)**	**127.7 (106.6 to 151.3)**	**-0.7 (-10.8 to 9.3)**	**32.5 (29.4 to 36.4)**	**-0.6 (-7.8 to 7.4)**	**808.9 (730.1 to 904.9)**	**-1.5 (-9.1 to 6.7)**	**6.2 (2.9 to 10.5)**	**27.6 (18.0 to 40.2)**	**143.7 (68.8 to 239.9)**	**28.8 (18.5 to 42.1)**
**Middle SDI**	**54.0 (47.3 to 61.8)**	**-6.5 (-16.8 to 4.7)**	**1250.8 (1091.5 to 1429.1)**	**-7.2 (-17.6 to 4.4)**	**9.3 (7.6 to 11.3)**	**-8.7 (-20.3 to 3.8)**	**207.3 (167.9 to 251.0)**	**-9.8 (-21.6 to 3.0)**	**45.4 (39.9 to 51.3)**	**-8.6 (-19.0 to 3.4)**	**1046.2 (924.0 to 1180.1)**	**-9.4 (-19.9 to 2.6)**	**9.4 (4.6 to 15.6)**	**8.2 (-1.7 to 21.3)**	**214.4 (109.5 to 351.6)**	**9.2 (-1.3 to 23.2)**
**High-middle SDI**	**62.3 (55.7 to 70.0)**	**-8.7 (-16.2 to -0.8)**	**1453.3 (1300.6 to 1627.8)**	**-10.2 (-17.9 to -2.1)**	**10.4 (8.5 to 12.3)**	**-12.2 (-20.9 to -2.9)**	**232.2 (191.8 to 275.6)**	**-14.4 (-23.1 to -4.9)**	**52.1 (47.3 to 57.5)**	**-9.6 (-17.4 to -1.3)**	**1222.7 (1108.4 to 1352.0)**	**-11.2 (-19.3 to -2.6)**	**12.2 (6.5 to 19.7)**	**-3.5 (-9.6 to 3.7)**	**271.9 (147.0 to 431.1)**	**-3.0 (-9.6 to 4.8)**
**High SDI**	**60.8 (55.4 to 66.7)**	**-8.5 (-10.4 to -6.6)**	**1346.8 (1249.8 to 1465.1)**	**-10.0 (-12.1 to -7.8)**	**10.3 (8.5 to 12.2)**	**-12.1 (-15.8 to -8.5)**	**201.4 (164.6 to 237.0)**	**-14.7 (-18.4 to -10.7)**	**49.5 (46.0 to 52.2)**	**-10.6 (-12.3 to -9.0)**	**1109.5 (1053.2 to 1162.7)**	**-12.1 (-13.9 to -10.2)**	**14.4 (7.7 to 23.0)**	**2.2 (-0.6 to 5.5)**	**310.3 (172.3 to 486.6)**	**1.9 (-1.2 to 5.4)**
**Central Europe, Eastern Europe, and Central Asia**	**65.4 (58.6 to 72.9)**	**-7.4 (-14.5 to -0.2)**	**1655.5 (1486.7 to 1841.7)**	**-9.0 (-16.0 to -1.7)**	**7.8 (6.1 to 9.6)**	**-15.6 (-23.2 to -7.9)**	**193.7 (149.6 to 239.0)**	**-17.5 (-24.9 to -9.8)**	**54.3 (49.9 to 59.5)**	**-8.8 (-15.9 to -1.6)**	**1394.4 (1277.0 to 1528.6)**	**-10.3 (-17.3 to -3.1)**	**14.8 (8.7 to 22.2)**	**3.0 (-4.6 to 11.0)**	**345.2 (206.3 to 510.3)**	**1.8 (-5.9 to 10.0)**
**Central Asia**	**51.0 (44.6 to 58.5)**	**-5.5 (-13.1 to 3.0)**	**1278.8 (1114.0 to 1472.7)**	**-7.3 (-15.3 to 1.6)**	**6.7 (5.3 to 8.4)**	**-7.5 (-18.3 to 4.8)**	**166.8 (131.8 to 212.4)**	**-9.7 (-20.4 to 2.5)**	**41.0 (37.0 to 45.7)**	**-7.6 (-15.1 to 0.6)**	**1035.2 (927.0 to 1155.3)**	**-9.5 (-17.3 to -0.7)**	**12.3 (7.1 to 18.7)**	**7.4 (-0.9 to 17.7)**	**293.0 (169.1 to 441.3)**	**6.2 (-2.4 to 17.3)**
Armenia	66.3 (54.4 to 78.9)	-8.7 (-22.8 to 7.2)	1610.2 (1310.9 to 1921.7)	-11.1 (-25.8 to 5.0)	13.8 (9.8 to 18.6)	-10.5 (-28.4 to 10.2)	333.6 (238.0 to 454.4)	-12.4 (-31.1 to 9.1)	53.2 (44.7 to 62.4)	-11.1 (-24.6 to 4.4)	1303.4 (1082.8 to 1546.9)	-13.6 (-27.3 to 2.4)	14.5 (7.8 to 23.8)	4.5 (-11.4 to 24.8)	331.2 (177.9 to 534.4)	3.3 (-13.4 to 24.4)
Azerbaijan	56.4 (45.9 to 70.7)	-0.5 (-14.7 to 16.9)	1412.3 (1143.6 to 1755.8)	-3.1 (-18.2 to 15.2)	6.8 (4.4 to 10.4)	-2.4 (-23.0 to 22.5)	181.7 (117.4 to 278.2)	-3.8 (-25.1 to 21.6)	46.3 (37.5 to 58.1)	-2.3 (-17.0 to 15.9)	1164.6 (936.9 to 1449.6)	-4.8 (-20.2 to 14.1)	13.0 (7.1 to 20.2)	13.4 (-1.8 to 33.1)	310.8 (169.8 to 479.7)	11.2 (-4.6 to 32.1)
Georgia	63.9 (53.0 to 76.2)	8.5 (-10.5 to 29.0)	1725.3 (1423.2 to 2065.5)	6.4 (-13.3 to 28.5)	11.0 (7.8 to 16.0)	20.8 (-5.8 to 56.0)	297.5 (213.2 to 434.8)	17.1 (-10.1 to 51.6)	52.4 (44.1 to 61.8)	7.7 (-11.6 to 28.5)	1427.3 (1183.6 to 1689.9)	5.7 (-14.1 to 27.8)	14.1 (7.4 to 23.4)	16.8 (-3.8 to 39.2)	358.0 (188.4 to 588.5)	15.1 (-6.4 to 37.9)
Kazakhstan	56.6 (47.8 to 66.3)	-17.4 (-28.6 to -6.0)	1435.1 (1203.5 to 1688.4)	-18.6 (-30.0 to -6.5)	7.2 (5.0 to 10.6)	-22.7 (-37.0 to -6.7)	181.7 (126.3 to 266.1)	-24.2 (-39.0 to -8.0)	44.8 (38.5 to 51.7)	-19.6 (-30.8 to -8.5)	1153.0 (988.4 to 1331.5)	-20.6 (-31.7 to -8.9)	15.1 (8.7 to 22.9)	-5.4 (-17.6 to 8.3)	357.5 (206.9 to 537.2)	-6.1 (-18.9 to 7.9)
Kyrgyzstan	36.7 (31.5 to 43.4)	-5.6 (-16.5 to 6.2)	938.8 (797.1 to 1108.7)	-8.2 (-19.1 to 4.3)	4.2 (3.0 to 6.1)	-11.1 (-25.3 to 3.0)	107.0 (76.8 to 155.0)	-13.6 (-27.5 to 0.7)	31.3 (27.0 to 36.2)	-6.4 (-17.1 to 5.3)	805.2 (693.0 to 942.3)	-9.2 (-20.1 to 3.3)	6.4 (3.6 to 9.9)	6.1 (-6.2 to 21.7)	151.8 (85.7 to 231.2)	5.0 (-8.1 to 20.9)
Mongolia	136.2 (106.4 to 174.0)	-4.4 (-21.2 to 16.2)	3116.5 (2400.3 to 4075.8)	-6.2 (-24.4 to 16.6)	12.8 (8.7 to 19.0)	-3.1 (-24.0 to 23.1)	287.4 (192.4 to 426.2)	-4.7 (-27.2 to 23.7)	117.0 (92.0 to 146.4)	-4.4 (-21.0 to 16.4)	2674.4 (2067.0 to 3425.7)	-6.3 (-24.3 to 16.3)	28.6 (14.3 to 48.3)	1.4 (-17.5 to 28.2)	666.1 (341.2 to 1126.0)	-0.5 (-20.2 to 28.1)
Tajikistan	35.7 (27.7 to 47.9)	-5.9 (-24.5 to 16.3)	843.5 (645.6 to 1139.9)	-9.2 (-28.4 to 14.7)	4.5 (3.0 to 6.3)	-7.8 (-28.8 to 17.9)	109.5 (74.9 to 152.5)	-12.2 (-32.9 to 14.2)	29.2 (23.0 to 39.1)	-10.4 (-28.6 to 10.7)	687.6 (535.4 to 930.9)	-13.7 (-32.2 to 9.1)	6.9 (3.1 to 12.1)	26.5 (0.3 to 60.9)	159.9 (73.3 to 278.1)	23.1 (-4.5 to 59.4)
Turkmenistan	35.6 (27.9 to 44.9)	9.9 (-10.9 to 37.5)	971.0 (757.3 to 1234.3)	8.3 (-13.1 to 37.4)	3.0 (1.9 to 4.8)	16.2 (-18.4 to 60.8)	84.9 (53.7 to 138.4)	15.4 (-22.2 to 70.3)	28.2 (22.6 to 35.3)	5.9 (-14.3 to 32.5)	776.7 (619.8 to 983.7)	4.3 (-16.3 to 32.3)	9.8 (5.3 to 14.9)	29.9 (3.3 to 63.5)	250.5 (135.6 to 380.8)	29.7 (2.7 to 66.0)
Uzbekistan	38.5 (31.6 to 46.5)	3.8 (-10.9 to 19.7)	975.8 (799.0 to 1185.0)	3.7 (-12.7 to 21.2)	4.5 (3.2 to 6.4)	8.5 (-12.3 to 31.4)	114.4 (80.8 to 162.2)	8.2 (-13.3 to 32.1)	29.7 (24.9 to 34.7)	0.4 (-14.3 to 15.8)	759.6 (625.7 to 905.4)	0.4 (-15.8 to 17.4)	9.9 (5.5 to 15.4)	20.3 (3.1 to 41.2)	236.4 (133.1 to 361.1)	20.4 (1.3 to 43.6)
**Central Europe**	**82.0 (71.0 to 94.9)**	**-6.1 (-17.3 to 5.7)**	**2009.1 (1728.6 to 2321.7)**	**-8.0 (-19.5 to 4.1)**	**11.8 (9.4 to 14.7)**	**-11.3 (-23.4 to 1.1)**	**289.2 (227.5 to 361.9)**	**-13.7 (-25.9 to -1.2)**	**68.1 (59.8 to 77.5)**	**-7.4 (-18.5 to 4.3)**	**1696.7 (1481.1 to 1936.3)**	**-9.3 (-20.8 to 2.9)**	**19.7 (11.0 to 31.0)**	**3.6 (-9.0 to 17.0)**	**441.3 (253.7 to 689.8)**	**2.2 (-10.8 to 16.2)**
Albania	50.0 (37.1 to 66.2)	11.9 (-15.2 to 43.0)	1145.9 (837.3 to 1529.8)	10.2 (-17.7 to 43.1)	8.4 (5.2 to 12.7)	4.5 (-23.7 to 39.3)	193.5 (117.9 to 293.1)	3.0 (-25.9 to 39.4)	43.5 (32.3 to 57.7)	12.1 (-15.2 to 43.6)	991.9 (726.3 to 1325.7)	10.1 (-18.0 to 43.1)	8.4 (4.2 to 14.7)	23.1 (-7.1 to 56.2)	185.5 (94.9 to 321.3)	23.2 (-8.5 to 59.5)
Bosnia and Herzegovina	82.9 (65.0 to 105.1)	2.1 (-19.3 to 27.1)	1960.9 (1524.2 to 2506.6)	0.7 (-22.2 to 27.4)	12.8 (8.9 to 17.8)	-11.5 (-33.4 to 14.1)	308.9 (213.8 to 431.5)	-12.5 (-35.0 to 14.4)	70.0 (55.2 to 87.6)	2.1 (-19.5 to 28.1)	1672.2 (1296.5 to 2119.2)	0.9 (-21.9 to 28.5)	20.5 (10.3 to 35.8)	10.0 (-12.3 to 35.4)	449.9 (225.4 to 787.7)	8.4 (-14.7 to 36.1)
Bulgaria	80.5 (63.7 to 101.2)	-4.2 (-24.1 to 19.8)	2173.1 (1699.3 to 2756.8)	-3.6 (-24.8 to 22.2)	8.5 (6.0 to 11.6)	-10.0 (-31.1 to 17.4)	235.6 (166.2 to 325.2)	-9.6 (-31.1 to 18.8)	69.5 (55.0 to 86.8)	-5.2 (-25.2 to 19.1)	1901.9 (1490.7 to 2404.6)	-4.6 (-25.9 to 21.3)	17.1 (9.3 to 28.6)	1.7 (-19.1 to 26.4)	419.2 (229.2 to 700.0)	3.2 (-18.7 to 29.9)
Croatia	77.8 (61.2 to 98.7)	-11.1 (-29.2 to 11.1)	1813.2 (1408.8 to 2328.0)	-13.2 (-31.8 to 10.4)	14.2 (9.8 to 19.4)	-19.6 (-41.0 to 8.6)	324.9 (224.4 to 450.4)	-22.2 (-43.9 to 6.6)	63.5 (50.8 to 79.7)	-12.3 (-30.0 to 9.8)	1506.5 (1177.8 to 1915.9)	-14.2 (-32.5 to 9.0)	19.7 (10.6 to 32.9)	-1.1 (-20.6 to 22.2)	421.6 (228.7 to 701.8)	-2.8 (-23.9 to 22.9)
Czechia	72.0 (57.6 to 89.7)	-14.9 (-29.8 to 2.7)	1640.0 (1313.1 to 2041.2)	-17.1 (-32.2 to 1.4)	9.2 (6.3 to 13.1)	-21.7 (-37.7 to -1.4)	208.7 (140.5 to 297.5)	-24.2 (-40.2 to -3.6)	56.5 (46.4 to 68.5)	-17.1 (-31.8 to 0.0)	1310.8 (1067.6 to 1602.2)	-19.2 (-34.4 to -1.5)	23.4 (12.7 to 38.5)	-3.4 (-20.7 to 16.4)	498.2 (276.5 to 814.1)	-5.2 (-23.0 to 16.0)
Hungary	96.9 (79.4 to 118.7)	-14.2 (-28.9 to 3.6)	2435.0 (1977.4 to 2991.0)	-16.4 (-31.5 to 1.8)	13.6 (9.6 to 18.6)	-17.0 (-33.6 to 3.7)	344.3 (240.4 to 476.4)	-20.0 (-36.5 to 1.2)	81.5 (67.2 to 99.0)	-15.2 (-29.9 to 2.6)	2085.0 (1708.0 to 2541.1)	-17.5 (-32.4 to 0.7)	23.0 (12.7 to 37.7)	-8.5 (-23.7 to 9.5)	521.4 (291.5 to 846.0)	-9.7 (-25.7 to 9.1)
North Macedonia	87.1 (68.2 to 109.1)	-4.9 (-23.9 to 18.3)	2126.8 (1639.4 to 2695.3)	-5.7 (-26.1 to 19.4)	12.5 (8.4 to 17.7)	-11.0 (-32.2 to 12.4)	322.9 (215.0 to 462.6)	-11.6 (-34.3 to 13.2)	73.4 (58.2 to 92.2)	-5.8 (-24.8 to 17.4)	1812.3 (1414.0 to 2304.5)	-6.5 (-27.2 to 18.5)	21.6 (11.1 to 37.2)	2.9 (-18.1 to 25.6)	485.9 (249.1 to 832.8)	1.7 (-19.8 to 26.4)
Montenegro	95.3 (79.0 to 115.0)	-1.0 (-16.9 to 16.9)	2358.0 (1947.9 to 2854.5)	-3.0 (-19.0 to 15.9)	13.8 (9.9 to 18.7)	-8.4 (-25.0 to 10.3)	347.3 (247.4 to 477.7)	-10.2 (-27.3 to 9.3)	82.8 (69.3 to 98.6)	-1.6 (-17.5 to 16.6)	2069.2 (1719.8 to 2490.1)	-3.5 (-19.6 to 15.7)	21.2 (11.2 to 35.7)	8.7 (-8.3 to 26.6)	477.9 (256.6 to 802.1)	6.8 (-10.3 to 26.0)
Poland	88.3 (73.2 to 106.4)	-4.2 (-19.1 to 13.0)	2083.7 (1726.8 to 2510.8)	-6.8 (-21.8 to 10.8)	14.8 (11.4 to 18.8)	-8.1 (-24.9 to 11.1)	344.8 (263.9 to 442.0)	-11.3 (-28.1 to 7.6)	72.6 (60.6 to 86.4)	-5.6 (-20.7 to 12.1)	1739.2 (1447.2 to 2086.5)	-8.1 (-23.3 to 9.8)	21.3 (11.4 to 33.8)	6.9 (-8.8 to 25.2)	467.0 (255.1 to 738.2)	4.8 (-11.0 to 23.3)
Romania	72.0 (58.8 to 87.4)	-1.6 (-18.8 to 18.4)	1920.1 (1568.4 to 2349.9)	-4.0 (-21.3 to 16.2)	7.8 (5.5 to 11.1)	-9.2 (-28.0 to 12.3)	207.1 (145.2 to 298.0)	-11.1 (-29.7 to 11.0)	61.7 (50.4 to 74.8)	-3.2 (-20.4 to 16.3)	1669.6 (1351.2 to 2036.6)	-5.4 (-22.9 to 14.5)	14.0 (8.2 to 21.8)	12.8 (-6.7 to 35.4)	338.5 (201.3 to 523.5)	10.9 (-8.9 to 33.8)
Serbia	94.7 (74.6 to 120.0)	-4.1 (-23.3 to 18.7)	2351.7 (1826.2 to 3005.6)	-5.8 (-25.8 to 18.2)	14.6 (10.1 to 20.2)	-12.8 (-33.5 to 12.4)	375.6 (258.9 to 524.7)	-14.4 (-35.8 to 11.8)	79.0 (62.8 to 98.2)	-5.3 (-24.2 to 17.4)	1993.2 (1560.6 to 2515.3)	-6.7 (-26.2 to 17.1)	23.3 (12.2 to 39.7)	6.8 (-13.1 to 32.3)	524.6 (277.9 to 893.8)	5.1 (-16.1 to 32.6)
Slovakia	71.8 (55.7 to 91.8)	-7.3 (-25.8 to 15.4)	1745.9 (1341.1 to 2260.7)	-8.8 (-28.2 to 14.9)	7.7 (5.2 to 11.1)	-14.4 (-35.0 to 13.8)	187.4 (126.2 to 271.4)	-15.0 (-35.9 to 12.8)	58.1 (45.8 to 73.5)	-8.8 (-26.7 to 13.9)	1441.4 (1116.8 to 1842.8)	-10.3 (-29.4 to 13.3)	18.6 (10.8 to 30.2)	1.3 (-19.7 to 26.5)	416.7 (241.8 to 671.6)	0.0 (-21.7 to 26.1)
Slovenia	66.5 (51.2 to 86.4)	-10.0 (-29.5 to 16.5)	1543.5 (1181.5 to 2041.2)	-11.3 (-31.4 to 15.7)	12.3 (8.2 to 17.5)	-15.0 (-38.3 to 16.1)	279.0 (185.4 to 404.8)	-17.4 (-40.5 to 14.4)	53.2 (41.4 to 69.3)	-9.4 (-28.9 to 17.5)	1265.1 (969.9 to 1663.0)	-10.9 (-31.2 to 16.5)	16.7 (9.2 to 27.4)	-7.4 (-27.3 to 18.4)	352.2 (193.4 to 575.2)	-8.0 (-28.8 to 19.0)
**Eastern Europe**	**58.4 (51.3 to 66.6)**	**-8.3 (-17.2 to 1.1)**	**1538.0 (1356.0 to 1753.5)**	**-9.0 (-18.0 to 0.5)**	**5.5 (4.0 to 7.1)**	**-21.8 (-31.8 to -11.7)**	**142.8 (102.3 to 185.0)**	**-22.4 (-32.7 to -12.1)**	**48.9 (43.3 to 54.9)**	**-9.4 (-18.4 to 0.2)**	**1307.1 (1155.0 to 1471.1)**	**-10.0 (-19.2 to 0.0)**	**12.2 (7.4 to 18.0)**	**1.3 (-7.8 to 12.0)**	**295.9 (183.7 to 436.6)**	**0.8 (-8.2 to 11.5)**
Belarus	57.4 (44.6 to 73.4)	-14.0 (-32.0 to 9.7)	1505.1 (1149.7 to 1949.8)	-15.6 (-34.4 to 9.4)	4.9 (3.2 to 7.1)	-29.1 (-49.8 to -0.9)	127.8 (82.9 to 189.9)	-30.2 (-53.0 to 1.6)	49.8 (38.7 to 63.7)	-14.7 (-32.8 to 9.0)	1317.5 (1017.0 to 1704.4)	-16.2 (-34.9 to 8.0)	10.2 (5.6 to 16.3)	-4.8 (-24.3 to 20.6)	247.4 (137.9 to 393.2)	-6.0 (-26.8 to 19.9)
Estonia	64.1 (50.3 to 80.9)	-7.5 (-26.5 to 16.3)	1525.9 (1186.1 to 1942.5)	-7.8 (-27.9 to 17.1)	5.2 (3.1 to 8.3)	-27.0 (-48.4 to -1.7)	119.3 (67.5 to 190.3)	-27.2 (-49.0 to -2.2)	53.5 (42.2 to 66.8)	-7.9 (-27.3 to 15.6)	1292.6 (1010.3 to 1639.1)	-8.4 (-28.6 to 16.1)	15.0 (8.8 to 23.2)	-1.4 (-22.2 to 24.5)	332.7 (196.3 to 516.1)	-0.9 (-23.1 to 25.7)
Latvia	62.2 (51.8 to 75.5)	-11.9 (-26.4 to 5.3)	1534.1 (1267.6 to 1870.4)	-12.9 (-27.9 to 5.1)	6.2 (4.0 to 9.3)	-28.5 (-45.6 to -8.2)	150.2 (97.4 to 231.6)	-29.6 (-46.7 to -8.7)	51.2 (42.6 to 61.4)	-12.2 (-26.8 to 5.0)	1282.2 (1059.8 to 1547.6)	-13.1 (-28.6 to 5.1)	14.6 (8.5 to 21.9)	-6.4 (-21.2 to 11.4)	335.5 (198.2 to 500.4)	-7.0 (-22.1 to 12.2)
Lithuania	59.6 (48.2 to 73.4)	-13.7 (-29.2 to 4.0)	1480.7 (1190.2 to 1844.1)	-15.2 (-31.0 to 3.5)	4.5 (3.0 to 6.7)	-33.6 (-49.4 to -15.5)	110.3 (72.2 to 164.2)	-33.9 (-49.9 to -15.6)	49.5 (40.7 to 60.0)	-14.1 (-29.7 to 3.2)	1253.9 (1011.5 to 1540.3)	-15.5 (-31.5 to 2.9)	13.2 (7.9 to 20.1)	-7.1 (-24.3 to 12.0)	302.3 (178.1 to 458.9)	-8.5 (-25.8 to 11.5)
Moldova	50.1 (43.0 to 58.1)	-16.0 (-26.6 to -4.5)	1378.2 (1181.8 to 1595.5)	-18.5 (-28.9 to -6.6)	3.5 (2.1 to 5.5)	-29.0 (-41.5 to -16.0)	95.9 (58.8 to 151.9)	-31.4 (-44.0 to -18.5)	42.6 (37.0 to 48.3)	-16.8 (-27.8 to -5.3)	1185.3 (1023.7 to 1352.4)	-19.4 (-30.0 to -7.5)	10.5 (6.1 to 16.2)	-7.5 (-19.3 to 6.6)	268.3 (156.5 to 409.9)	-9.5 (-21.4 to 5.2)
Russia	57.9 (49.3 to 67.8)	-12.5 (-23.6 to 0.5)	1499.5 (1273.2 to 1755.6)	-14.1 (-25.2 to -0.8)	5.5 (3.8 to 7.2)	-26.9 (-38.7 to -14.6)	138.2 (95.8 to 185.3)	-28.7 (-40.7 to -16.1)	48.4 (41.4 to 56.1)	-13.8 (-25.6 to -0.5)	1271.1 (1082.2 to 1477.7)	-15.1 (-26.7 to -1.4)	12.4 (7.5 to 18.4)	-1.3 (-12.5 to 12.6)	294.4 (180.8 to 434.5)	-2.8 (-14.1 to 11.3)
Ukraine	60.4 (50.2 to 71.7)	8.9 (-8.2 to 27.0)	1684.9 (1394.2 to 2014.3)	11.9 (-6.4 to 31.8)	6.0 (4.1 to 8.6)	1.2 (-19.9 to 24.5)	167.0 (112.3 to 241.7)	4.9 (-17.9 to 32.7)	50.8 (42.6 to 60.1)	8.4 (-9.7 to 27.3)	1434.2 (1194.6 to 1706.5)	11.3 (-8.2 to 31.7)	12.0 (7.2 to 18.1)	13.8 (-3.3 to 33.5)	310.0 (187.2 to 467.2)	16.9 (-0.6 to 37.6)

Location	All risk factors*				Environmental and occupational risks*				Behavioural risks*				Metabolic risks*			
	Death ASR	Death % change	DALY ASR	DALY % change	Death ASR	Death % change	DALY ASR	DALY % change	Death ASR	Death % change	DALY ASR	DALY % change	Death ASR	Death % change	DALY ASR	DALY % change
**High-income**	**61.1 (55.8 to 67.2)**	**-8.0 (-9.4 to -6.6)**	**1361.0 (1264.2 to 1478.7)**	**-9.4 (-10.9 to -7.8)**	**10.4 (8.5 to 12.2)**	**-12.4 (-15.8 to -8.9)**	**202.9 (166.9 to 240.3)**	**-14.9 (-18.6 to -11.2)**	**49.8 (46.5 to 52.5)**	**-9.9 (-11.0 to -8.7)**	**1126.7 (1072.5 to 1177.0)**	**-11.2 (-12.4 to -9.9)**	**14.5 (7.5 to 23.2)**	**1.2 (-0.9 to 3.9)**	**308.3 (167.8 to 485.5)**	**0.9 (-1.4 to 3.7)**
**Australasia**	**52.1 (47.3 to 57.2)**	**-8.1 (-11.0 to -5.1)**	**1151.2 (1064.5 to 1253.2)**	**-8.8 (-12.1 to -5.2)**	**10.7 (8.8 to 12.4)**	**-11.4 (-18.2 to -4.3)**	**197.4 (163.2 to 231.6)**	**-13.8 (-21.7 to -6.0)**	**39.0 (35.8 to 41.5)**	**-10.8 (-13.5 to -7.9)**	**893.5 (838.2 to 945.5)**	**-11.0 (-14.2 to -7.8)**	**13.0 (7.5 to 19.8)**	**0.7 (-4.0 to 6.2)**	**274.6 (163.9 to 409.0)**	**1.4 (-3.5 to 6.7)**
Australia	51.5 (46.7 to 56.8)	-8.2 (-11.6 to -4.9)	1137.9 (1048.7 to 1243.3)	-8.9 (-13.0 to -5.0)	10.9 (9.0 to 12.7)	-11.6 (-19.3 to -3.7)	200.4 (164.7 to 236.1)	-14.1 (-22.7 to -5.3)	38.2 (34.9 to 40.7)	-10.8 (-14.0 to -7.5)	877.6 (822.1 to 931.9)	-11.0 (-14.8 to -7.1)	13.0 (7.5 to 19.9)	-0.8 (-6.1 to 5.2)	274.2 (164.4 to 407.6)	-0.2 (-5.9 to 6.0)
New Zealand	55.2 (50.1 to 60.3)	-7.2 (-10.8 to -3.7)	1222.5 (1125.0 to 1325.8)	-7.9 (-11.2 to -4.3)	9.4 (7.6 to 11.3)	-10.4 (-17.6 to -3.3)	181.1 (144.6 to 219.3)	-12.2 (-19.5 to -4.9)	43.5 (40.1 to 46.4)	-10.4 (-13.7 to -7.1)	979.0 (917.6 to 1039.8)	-10.9 (-13.9 to -7.5)	13.0 (7.2 to 20.0)	9.6 (2.0 to 16.8)	276.4 (158.6 to 414.3)	10.4 (2.6 to 18.9)
**High-income Asia Pacific**	**48.0 (42.1 to 54.3)**	**-13.9 (-16.0 to -11.7)**	**1023.0 (925.0 to 1141.0)**	**-16.5 (-18.6 to -14.2)**	**7.3 (5.8 to 8.9)**	**-6.4 (-13.1 to 0.7)**	**133.5 (107.6 to 162.4)**	**-9.4 (-16.2 to -2.0)**	**41.6 (37.2 to 45.4)**	**-16.2 (-18.1 to -14.2)**	**895.9 (825.9 to 967.2)**	**-18.6 (-20.6 to -16.3)**	**7.5 (2.9 to 13.5)**	**0.1 (-6.3 to 6.5)**	**153.3 (62.6 to 273.9)**	**-2.8 (-9.0 to 3.8)**
Brunei	82.5 (69.6 to 98.3)	-5.0 (-15.1 to 6.1)	1722.1 (1453.9 to 2059.6)	-3.0 (-13.9 to 8.8)	8.6 (6.2 to 11.4)	3.2 (-16.2 to 27.3)	153.4 (109.3 to 206.8)	4.0 (-15.4 to 28.0)	64.6 (57.2 to 73.1)	-7.3 (-17.6 to 4.1)	1357.3 (1193.4 to 1537.3)	-5.1 (-16.0 to 6.9)	25.6 (10.6 to 44.5)	-4.0 (-14.9 to 8.1)	513.3 (216.5 to 890.7)	-1.7 (-13.2 to 11.9)
Japan	47.0 (41.2 to 53.2)	-13.8 (-16.1 to -11.8)	1005.2 (908.7 to 1118.6)	-15.8 (-17.9 to -13.5)	7.0 (5.5 to 8.5)	-7.5 (-14.6 to 0.0)	125.9 (100.5 to 153.0)	-10.4 (-17.7 to -2.8)	41.0 (36.7 to 44.9)	-16.0 (-18.0 to -13.8)	889.5 (818.5 to 958.4)	-17.6 (-19.6 to -15.4)	6.8 (2.6 to 12.6)	-1.0 (-7.5 to 5.8)	138.0 (53.6 to 250.2)	-3.0 (-9.4 to 4.0)
Singapore	36.2 (31.6 to 41.4)	-17.9 (-22.1 to -13.9)	758.0 (669.6 to 864.7)	-19.0 (-23.3 to -14.7)	6.4 (4.8 to 7.9)	-18.6 (-29.7 to -6.0)	116.0 (89.1 to 144.5)	-19.3 (-30.0 to -6.9)	28.1 (25.1 to 30.6)	-19.4 (-23.4 to -15.3)	592.4 (538.4 to 642.7)	-20.5 (-24.7 to -16.1)	8.5 (3.9 to 14.5)	-14.8 (-21.4 to -6.4)	177.1 (84.5 to 297.3)	-15.8 (-22.6 to -6.9)
South Korea	53.5 (46.9 to 60.7)	-12.5 (-17.3 to -7.5)	1113.6 (985.0 to 1259.7)	-17.7 (-22.5 to -12.6)	8.3 (6.4 to 10.7)	-3.0 (-14.0 to 10.4)	155.8 (119.0 to 200.4)	-8.2 (-18.3 to 4.3)	45.9 (41.3 to 51.1)	-14.9 (-19.5 to -9.9)	959.6 (870.1 to 1056.7)	-19.9 (-24.6 to -14.7)	9.6 (4.0 to 17.1)	1.5 (-6.5 to 11.4)	196.2 (84.8 to 344.5)	-4.9 (-12.4 to 4.6)
**High-income North America**	**66.0 (60.5 to 72.1)**	**-6.3 (-8.2 to -4.6)**	**1476.2 (1374.5 to 1597.4)**	**-7.2 (-9.0 to -5.5)**	**10.3 (8.3 to 12.3)**	**-14.2 (-19.3 to -9.1)**	**192.4 (151.8 to 232.2)**	**-16.4 (-21.4 to -11.1)**	**52.8 (49.5 to 55.4)**	**-8.3 (-10.2 to -6.5)**	**1194.5 (1139.1 to 1243.9)**	**-9.1 (-11.0 to -7.4)**	**18.6 (10.3 to 28.9)**	**1.6 (-1.3 to 5.1)**	**408.4 (235.6 to 623.3)**	**1.2 (-1.6 to 4.6)**
Canada	61.1 (55.7 to 66.7)	-8.7 (-11.9 to -5.6)	1327.3 (1224.2 to 1434.9)	-9.7 (-13.4 to -6.3)	11.1 (8.6 to 13.5)	-12.0 (-21.6 to -2.2)	204.9 (157.9 to 251.9)	-14.4 (-24.5 to -4.3)	49.6 (45.7 to 52.7)	-11.5 (-14.5 to -8.4)	1091.8 (1023.7 to 1152.5)	-12.3 (-15.6 to -9.1)	13.6 (7.6 to 21.1)	4.6 (-1.2 to 11.0)	289.8 (167.9 to 438.8)	4.0 (-1.9 to 10.6)
Greenland	139.4 (113.9 to 165.5)	-11.1 (-24.4 to 2.0)	3208.3 (2590.2 to 3847.6)	-12.9 (-26.5 to 1.2)	26.4 (18.5 to 36.8)	-14.2 (-31.3 to 3.3)	535.1 (364.2 to 777.5)	-16.0 (-33.6 to 1.8)	122.1 (101.0 to 143.4)	-12.5 (-25.5 to -0.1)	2833.8 (2304.7 to 3382.2)	-14.2 (-27.7 to -0.2)	25.0 (12.9 to 41.5)	6.4 (-10.2 to 23.2)	552.1 (296.9 to 909.3)	3.9 (-13.0 to 21.7)
United States of America	66.6 (61.0 to 72.8)	-6.1 (-8.0 to -4.3)	1493.8 (1391.2 to 1616.8)	-6.9 (-8.9 to -5.1)	10.2 (8.1 to 12.2)	-14.5 (-20.0 to -8.9)	190.9 (149.8 to 231.5)	-16.7 (-22.2 to -10.9)	53.1 (49.9 to 55.9)	-7.9 (-10.0 to -6.1)	1206.6 (1150.3 to 1256.9)	-8.8 (-10.8 to -6.9)	19.2 (10.6 to 29.9)	1.4 (-1.5 to 5.2)	422.7 (244.4 to 644.7)	1.0 (-1.8 to 4.6)
**Southern Latin America**	**64.2 (58.2 to 71.8)**	**-3.7 (-7.2 to -0.5)**	**1500.1 (1374.6 to 1654.7)**	**-5.0 (-8.8 to -1.5)**	**7.0 (5.7 to 8.6)**	**-6.2 (-15.4 to 4.8)**	**159.3 (130.4 to 195.5)**	**-8.5 (-17.4 to 2.4)**	**51.8 (48.6 to 55.3)**	**-6.0 (-9.2 to -2.9)**	**1234.6 (1163.6 to 1313.1)**	**-6.9 (-10.5 to -3.2)**	**16.1 (8.7 to 25.2)**	**9.7 (3.0 to 16.7)**	**340.7 (188.3 to 530.8)**	**8.0 (1.6 to 15.1)**
Argentina	69.6 (63.5 to 77.1)	-2.1 (-6.1 to 1.9)	1653.1 (1520.2 to 1811.0)	-3.6 (-8.0 to 0.7)	7.7 (6.1 to 9.7)	-6.4 (-16.4 to 5.8)	178.2 (142.8 to 223.6)	-8.7 (-18.4 to 3.5)	57.3 (53.8 to 61.0)	-4.1 (-8.0 to -0.2)	1385.3 (1306.9 to 1469.9)	-5.4 (-9.6 to -1.1)	16.4 (8.7 to 26.1)	12.4 (5.0 to 20.4)	351.0 (190.2 to 556.0)	10.9 (3.7 to 18.5)
Chile	49.2 (42.8 to 57.4)	-6.4 (-10.6 to -2.4)	1092.2 (968.8 to 1254.4)	-7.1 (-11.4 to -2.6)	5.4 (4.4 to 6.7)	-5.2 (-14.6 to 5.5)	113.8 (92.7 to 142.7)	-6.6 (-16.2 to 3.9)	36.5 (33.1 to 40.8)	-8.1 (-12.4 to -4.1)	832.3 (764.1 to 923.3)	-8.4 (-12.9 to -3.9)	15.2 (8.6 to 23.2)	-0.1 (-6.3 to 7.0)	313.7 (183.1 to 472.6)	-1.0 (-7.2 to 6.0)
Uruguay	79.4 (72.6 to 87.4)	-3.3 (-7.8 to 1.5)	1889.1 (1735.8 to 2061.4)	-4.7 (-9.5 to 0.7)	8.0 (6.0 to 10.6)	0.8 (-11.1 to 14.8)	190.9 (145.3 to 253.0)	-1.5 (-13.1 to 11.8)	66.6 (62.3 to 70.7)	-6.6 (-11.0 to -2.4)	1611.2 (1507.3 to 1717.6)	-7.5 (-12.2 to -2.8)	17.1 (9.1 to 27.0)	23.4 (9.8 to 40.5)	369.9 (199.6 to 574.8)	21.0 (8.0 to 38.0)
**Western Europe**	**63.8 (58.4 to 69.7)**	**-8.0 (-9.7 to -6.2)**	**1422.3 (1321.7 to 1536.8)**	**-9.8 (-11.8 to -7.7)**	**12.3 (10.2 to 14.3)**	**-12.9 (-17.3 to -8.6)**	**249.8 (207.3 to 293.6)**	**-15.8 (-20.6 to -11.1)**	**51.8 (48.4 to 54.4)**	**-9.4 (-11.0 to -7.9)**	**1177.8 (1119.3 to 1229.4)**	**-11.0 (-12.9 to -9.2)**	**14.8 (7.5 to 23.7)**	**-0.5 (-3.1 to 2.4)**	**303.5 (161.4 to 480.8)**	**-1.2 (-4.0 to 1.9)**
Andorra	76.5 (59.2 to 96.6)	-3.9 (-21.4 to 16.5)	1720.2 (1310.9 to 2200.4)	-4.8 (-23.2 to 16.7)	16.7 (11.3 to 23.3)	-8.8 (-29.1 to 16.0)	341.8 (227.0 to 498.5)	-10.2 (-31.5 to 15.4)	62.8 (48.2 to 78.4)	-5.8 (-22.7 to 14.0)	1432.2 (1082.1 to 1819.1)	-6.5 (-24.3 to 14.5)	16.4 (8.6 to 27.4)	7.1 (-12.3 to 30.3)	347.8 (185.0 to 576.0)	6.4 (-14.2 to 31.5)
Austria	54.9 (50.1 to 60.3)	-10.6 (-13.9 to -7.2)	1252.8 (1155.7 to 1361.7)	-12.9 (-16.8 to -9.1)	9.0 (7.1 to 11.2)	-18.4 (-26.8 to -9.7)	196.6 (149.7 to 247.8)	-21.8 (-30.1 to -13.2)	45.6 (42.8 to 48.4)	-11.3 (-14.7 to -7.9)	1062.2 (1001.3 to 1124.2)	-13.6 (-17.3 to -9.7)	11.7 (6.0 to 18.8)	-3.2 (-8.7 to 3.3)	243.0 (126.7 to 385.8)	-4.9 (-10.9 to 1.5)
Belgium	70.2 (64.8 to 76.3)	-8.1 (-11.7 to -4.5)	1559.8 (1448.9 to 1685.0)	-11.2 (-15.1 to -7.1)	16.2 (13.1 to 19.2)	-13.6 (-21.7 to -5.6)	327.4 (263.8 to 395.3)	-17.9 (-26.2 to -9.3)	58.2 (54.6 to 61.8)	-9.6 (-13.0 to -6.3)	1316.9 (1244.4 to 1392.0)	-12.4 (-16.3 to -8.5)	13.8 (6.9 to 22.7)	3.3 (-3.1 to 10.4)	283.3 (143.8 to 458.6)	0.8 (-5.6 to 7.9)
Cyprus	52.0 (45.0 to 60.2)	-9.7 (-19.3 to 0.5)	1096.3 (957.2 to 1257.5)	-9.5 (-19.3 to 0.5)	8.9 (7.0 to 11.1)	-21.1 (-33.3 to -7.3)	169.4 (134.3 to 210.1)	-21.5 (-33.0 to -8.4)	42.6 (37.7 to 47.7)	-9.7 (-19.8 to 0.8)	915.0 (814.5 to 1020.0)	-9.5 (-19.8 to 0.9)	12.8 (5.9 to 21.8)	-6.8 (-16.8 to 4.6)	248.0 (117.7 to 416.5)	-6.0 (-16.3 to 5.1)
Denmark	75.7 (69.2 to 82.6)	-16.9 (-20.8 to -12.7)	1602.2 (1477.3 to 1747.7)	-18.2 (-22.4 to -13.7)	14.6 (11.8 to 17.8)	-19.7 (-28.9 to -10.3)	287.1 (228.1 to 353.5)	-21.8 (-31.2 to -12.0)	64.3 (59.3 to 69.1)	-19.1 (-22.9 to -15.2)	1364.6 (1272.0 to 1463.4)	-20.2 (-24.3 to -16.0)	13.9 (7.1 to 22.5)	-5.0 (-11.0 to 2.7)	283.1 (146.3 to 450.6)	-6.3 (-12.4 to 1.3)
Finland	45.4 (40.4 to 51.4)	-6.6 (-11.2 to -1.5)	979.8 (873.4 to 1103.0)	-9.5 (-14.7 to -3.6)	8.3 (6.4 to 10.4)	-13.2 (-24.8 to -0.7)	159.8 (120.9 to 202.6)	-16.8 (-28.3 to -4.3)	34.2 (31.6 to 36.6)	-7.9 (-12.3 to -3.1)	755.8 (702.7 to 810.9)	-10.7 (-15.5 to -4.9)	12.7 (6.5 to 20.4)	0.8 (-5.7 to 7.4)	260.1 (135.6 to 411.8)	-1.9 (-8.4 to 5.2)
France	65.5 (60.0 to 70.8)	-9.1 (-12.5 to -5.8)	1536.9 (1431.3 to 1646.9)	-11.1 (-15.2 to -7.2)	14.2 (11.3 to 16.9)	-9.1 (-19.5 to 2.2)	315.0 (248.4 to 379.3)	-10.9 (-22.2 to 1.1)	54.3 (50.5 to 57.6)	-10.4 (-13.6 to -7.1)	1297.4 (1220.4 to 1368.5)	-12.4 (-16.1 to -8.7)	11.2 (6.1 to 17.7)	-2.7 (-8.4 to 2.8)	238.3 (130.3 to 372.3)	-3.5 (-9.9 to 2.5)
Germany	66.2 (60.1 to 72.8)	-8.4 (-12.0 to -5.1)	1490.5 (1365.7 to 1628.7)	-9.5 (-13.3 to -5.5)	11.3 (9.1 to 13.7)	-15.8 (-24.5 to -6.4)	232.4 (184.6 to 286.8)	-18.5 (-27.0 to -9.2)	53.5 (50.1 to 56.4)	-10.1 (-13.3 to -7.0)	1234.6 (1167.7 to 1304.1)	-10.9 (-14.3 to -7.2)	17.1 (8.7 to 27.8)	3.7 (-3.1 to 10.9)	350.9 (185.4 to 562.6)	3.7 (-3.6 to 11.0)
Greece	69.9 (64.0 to 75.5)	-0.2 (-4.5 to 4.2)	1567.4 (1444.1 to 1692.1)	-1.5 (-6.3 to 3.6)	11.0 (8.2 to 14.4)	-11.5 (-21.8 to -0.6)	239.9 (180.0 to 317.7)	-13.7 (-23.5 to -3.0)	60.8 (56.7 to 64.4)	0.0 (-4.3 to 4.7)	1377.9 (1292.4 to 1461.3)	-1.5 (-6.2 to 3.8)	13.4 (6.4 to 22.5)	6.2 (-0.7 to 13.2)	277.6 (135.9 to 464.5)	6.4 (-0.7 to 13.8)
Iceland	49.6 (43.6 to 56.4)	-14.6 (-22.1 to -5.7)	1097.4 (975.5 to 1241.3)	-13.0 (-21.1 to -3.8)	7.7 (5.9 to 9.6)	-14.7 (-26.7 to 1.3)	153.4 (118.2 to 190.8)	-12.1 (-25.2 to 4.1)	40.6 (36.2 to 45.1)	-17.0 (-24.6 to -8.8)	906.0 (820.3 to 1005.3)	-15.4 (-23.4 to -6.7)	11.4 (6.0 to 18.3)	-3.0 (-12.2 to 7.9)	242.5 (133.2 to 383.5)	-1.5 (-11.2 to 10.3)
Ireland	61.9 (56.0 to 68.3)	-10.2 (-15.1 to -4.9)	1307.4 (1191.2 to 1441.9)	-11.5 (-16.9 to -5.9)	9.0 (6.6 to 11.6)	-18.4 (-29.9 to -4.5)	172.8 (126.8 to 227.3)	-19.7 (-31.4 to -6.0)	51.1 (46.8 to 54.8)	-13.3 (-17.7 to -8.6)	1087.5 (1010.9 to 1170.0)	-14.2 (-18.8 to -9.2)	14.6 (7.7 to 23.2)	10.3 (1.1 to 20.0)	295.4 (161.0 to 463.0)	7.9 (-1.5 to 18.1)
Israel	47.2 (41.8 to 53.3)	-11.9 (-15.6 to -7.9)	1012.7 (909.9 to 1135.0)	-12.6 (-16.7 to -7.9)	6.9 (5.4 to 8.4)	-18.9 (-27.4 to -10.3)	146.7 (115.4 to 178.5)	-20.1 (-28.0 to -11.5)	36.4 (33.5 to 39.2)	-12.1 (-16.0 to -7.9)	795.5 (738.1 to 853.6)	-13.2 (-17.3 to -8.4)	12.9 (6.6 to 20.9)	-14.0 (-20.1 to -7.4)	255.9 (132.6 to 404.5)	-11.6 (-17.6 to -5.4)
Italy	57.8 (52.2 to 64.0)	-10.6 (-12.5 to -8.7)	1264.8 (1163.8 to 1382.4)	-11.6 (-13.4 to -9.6)	12.6 (10.4 to 14.7)	-15.5 (-20.4 to -10.8)	247.4 (204.8 to 288.7)	-18.2 (-23.4 to -13.5)	46.1 (42.4 to 49.0)	-10.9 (-12.9 to -8.7)	1027.7 (964.1 to 1083.5)	-12.0 (-14.0 to -10.0)	13.5 (6.5 to 22.4)	-12.8 (-16.8 to -7.4)	276.5 (136.9 to 452.9)	-11.9 (-15.8 to -6.8)
Luxembourg	60.1 (51.8 to 70.2)	-16.8 (-26.2 to -6.4)	1313.9 (1137.0 to 1524.5)	-18.6 (-28.1 to -8.0)	11.8 (8.9 to 15.1)	-21.3 (-34.7 to -4.5)	239.2 (177.2 to 307.4)	-23.5 (-36.8 to -7.3)	48.7 (42.7 to 55.2)	-19.5 (-28.7 to -9.6)	1081.2 (952.4 to 1228.5)	-21.1 (-30.6 to -11.1)	15.0 (7.5 to 25.6)	1.9 (-11.8 to 16.1)	301.0 (154.1 to 501.2)	-0.1 (-13.3 to 13.9)
Malta	47.3 (40.8 to 54.8)	-10.7 (-18.9 to -1.2)	1066.5 (928.3 to 1225.2)	-9.6 (-18.6 to 0.5)	9.5 (7.4 to 11.7)	-18.1 (-29.5 to -4.8)	191.7 (149.8 to 239.0)	-17.5 (-29.5 to -3.1)	37.3 (33.3 to 41.3)	-11.6 (-19.9 to -2.2)	863.4 (775.9 to 965.0)	-10.1 (-19.3 to 0.0)	11.9 (5.7 to 20.0)	-6.2 (-15.8 to 4.5)	244.5 (119.2 to 410.4)	-5.8 (-16.1 to 4.9)
Monaco	108.1 (85.9 to 129.4)	-6.4 (-21.7 to 9.0)	2443.0 (1906.6 to 2995.8)	-7.3 (-24.1 to 10.3)	24.1 (17.0 to 31.8)	-12.4 (-29.3 to 8.2)	498.5 (344.0 to 678.7)	-14.0 (-32.5 to 7.4)	87.2 (69.0 to 104.1)	-8.1 (-23.0 to 8.1)	2005.4 (1571.3 to 2452.0)	-8.7 (-25.3 to 9.7)	24.4 (12.3 to 40.6)	4.4 (-12.9 to 22.0)	512.4 (261.8 to 839.6)	3.5 (-15.0 to 23.3)
Netherlands	77.2 (70.4 to 84.3)	-8.5 (-12.3 to -4.6)	1638.3 (1509.8 to 1787.0)	-11.6 (-15.7 to -7.3)	17.6 (14.5 to 20.4)	-15.6 (-23.0 to -8.5)	339.5 (281.0 to 397.1)	-19.7 (-27.2 to -11.7)	63.6 (58.8 to 67.9)	-10.5 (-14.1 to -6.9)	1364.9 (1280.6 to 1454.4)	-13.6 (-17.5 to -9.5)	14.7 (7.2 to 23.8)	3.4 (-2.7 to 10.3)	297.0 (151.7 to 477.9)	1.5 (-4.9 to 8.4)
Norway	49.1 (43.9 to 55.0)	-17.3 (-20.4 to -14.3)	1051.5 (955.4 to 1163.8)	-19.5 (-22.5 to -16.5)	9.4 (7.5 to 11.8)	-21.0 (-27.2 to -14.3)	180.8 (141.4 to 227.4)	-24.1 (-30.6 to -17.2)	37.4 (34.4 to 40.0)	-22.4 (-25.2 to -19.8)	825.1 (771.3 to 878.3)	-23.6 (-26.4 to -21.0)	12.0 (5.8 to 19.9)	1.4 (-3.4 to 6.5)	239.1 (117.4 to 391.0)	-1.7 (-6.5 to 3.2)
Portugal	53.7 (48.1 to 60.5)	-10.1 (-13.8 to -6.3)	1301.0 (1177.7 to 1446.5)	-12.1 (-16.5 to -7.7)	5.3 (3.8 to 7.0)	-11.5 (-21.8 to 0.3)	121.6 (86.8 to 161.0)	-14.7 (-24.8 to -3.9)	44.0 (40.7 to 47.3)	-11.1 (-14.9 to -7.4)	1098.3 (1022.4 to 1179.2)	-12.9 (-17.4 to -8.4)	13.6 (6.6 to 22.2)	-6.2 (-11.2 to -0.5)	288.6 (145.5 to 467.9)	-7.8 (-13.3 to -1.5)
San Marino	67.9 (44.6 to 98.5)	-4.5 (-29.3 to 29.7)	1477.9 (941.4 to 2210.1)	-3.8 (-30.2 to 32.7)	12.8 (7.2 to 20.5)	-1.2 (-31.4 to 42.7)	251.9 (138.9 to 422.8)	-0.7 (-31.9 to 46.2)	54.9 (36.0 to 78.9)	-6.7 (-30.9 to 26.0)	1218.3 (770.5 to 1821.5)	-5.8 (-31.6 to 29.9)	15.0 (6.7 to 29.1)	4.0 (-22.7 to 39.5)	302.1 (133.0 to 584.5)	5.0 (-23.3 to 43.9)
Spain	60.6 (55.4 to 66.8)	-6.7 (-10.3 to -3.2)	1411.9 (1307.1 to 1536.8)	-8.5 (-12.5 to -4.6)	8.9 (6.8 to 11.3)	-12.9 (-23.0 to -1.7)	190.4 (143.5 to 244.8)	-16.1 (-25.8 to -5.2)	50.4 (46.9 to 53.3)	-7.3 (-10.9 to -3.9)	1202.2 (1130.7 to 1273.5)	-9.0 (-13.0 to -4.9)	14.8 (7.5 to 23.7)	-4.5 (-9.8 to 1.1)	309.3 (165.1 to 492.6)	-5.4 (-11.0 to 0.5)
Sweden	48.1 (43.4 to 53.0)	-5.6 (-8.5 to -2.7)	1007.6 (924.3 to 1102.9)	-8.8 (-11.6 to -6.0)	6.7 (5.2 to 8.7)	-11.9 (-19.4 to -4.9)	125.4 (97.1 to 166.0)	-16.8 (-23.8 to -9.7)	38.5 (35.5 to 40.9)	-6.8 (-9.7 to -3.9)	816.2 (766.9 to 863.3)	-9.9 (-12.7 to -7.1)	11.7 (6.1 to 18.7)	1.2 (-4.3 to 6.9)	236.7 (124.7 to 369.7)	-1.2 (-6.5 to 4.6)
Switzerland	50.7 (45.7 to 55.7)	-11.7 (-15.3 to -7.8)	1103.5 (1009.1 to 1203.5)	-15.1 (-19.1 to -10.7)	10.0 (8.0 to 12.0)	-20.4 (-28.7 to -11.7)	201.7 (160.4 to 244.2)	-23.8 (-32.6 to -14.8)	41.7 (38.3 to 44.5)	-13.1 (-16.5 to -9.3)	919.8 (855.9 to 982.5)	-16.7 (-20.4 to -12.5)	10.5 (5.2 to 17.1)	-2.5 (-8.0 to 5.5)	212.5 (107.1 to 342.6)	-4.0 (-9.9 to 4.3)
United Kingdom	73.8 (67.6 to 80.5)	-2.7 (-4.9 to -0.6)	1544.2 (1429.6 to 1673.8)	-5.0 (-7.2 to -2.8)	16.3 (13.8 to 18.6)	-7.1 (-10.8 to -3.5)	304.4 (256.8 to 348.6)	-10.9 (-15.1 to -6.8)	57.8 (54.0 to 61.1)	-5.5 (-7.5 to -3.5)	1232.8 (1169.2 to 1290.6)	-7.2 (-9.3 to -5.2)	19.5 (10.1 to 30.8)	11.1 (6.7 to 15.2)	390.0 (211.0 to 611.0)	8.4 (4.1 to 12.6)
**Latin America and Caribbean**	**39.5 (34.3 to 45.8)**	**-7.9 (-14.3 to -0.7)**	**948.2 (830.9 to 1092.4)**	**-8.4 (-15.2 to -0.7)**	**4.3 (3.6 to 5.2)**	**-11.4 (-19.0 to -2.6)**	**98.3 (80.8 to 118.5)**	**-12.2 (-20.1 to -2.9)**	**30.3 (27.5 to 34.0)**	**-11.8 (-17.8 to -5.4)**	**737.4 (669.9 to 825.3)**	**-12.1 (-18.4 to -5.1)**	**10.3 (5.7 to 16.1)**	**6.8 (-0.8 to 15.3)**	**232.4 (132.4 to 358.3)**	**6.5 (-1.4 to 15.6)**

Location	All risk factors* Death ASR	All risk factors* Death % change	All risk factors* DALY ASR	All risk factors* DALY % change	Environmental and occupational risks* Death ASR	Environmental and occupational risks* Death % change	Environmental and occupational risks* DALY ASR	Environmental and occupational risks* DALY % change	Behavioural risks* Death ASR	Behavioural risks* Death % change	Behavioural risks* DALY ASR	Behavioural risks* DALY % change	Metabolic risks* Death ASR	Metabolic risks* Death % change	Metabolic risks* DALY ASR	Metabolic risks* DALY % change
**Andean Latin America**	**33.2<br>(25.2 to 44.0)**	**-8.1<br>(-24.4 to 10.0)**	**782.6<br>(590.5 to 1036.4)**	**-9.5<br>(-26.7 to 10.1)**	**4.1<br>(3.0 to 5.4)**	**-11.3<br>(-31.5 to 12.1)**	**89.5<br>(65.0 to 118.2)**	**-11.8<br>(-32.8 to 13.8)**	**23.0<br>(17.9 to 30.1)**	**-12.1<br>(-27.2 to 5.1)**	**551.4<br>(428.0 to 720.3)**	**-13.2<br>(-29.9 to 5.5)**	**9.3<br>(5.3 to 14.8)**	**6.9<br>(-11.8 to 28.1)**	**207.7<br>(119.8 to 323.2)**	**6.0<br>(-13.2 to 29.3)**
Bolivia	50.3<br>(35.8 to 68.8)	4.5<br>(-11.3 to 22.1)	1168.0<br>(825.6 to 1609.4)	1.7<br>(-15.8 to 22.2)	6.3<br>(3.9 to 9.3)	3.2<br>(-18.2 to 27.3)	133.5<br>(84.0 to 197.1)	0.5<br>(-21.8 to 25.7)	36.6<br>(26.4 to 49.7)	1.7<br>(-14.1 to 18.5)	861.6<br>(615.0 to 1186.3)	-0.9<br>(-18.5 to 20.0)	12.6<br>(6.8 to 21.0)	18.7<br>(0.5 to 40.7)	279.8<br>(151.0 to 457.6)	16.2<br>(-3.6 to 40.2)
Ecuador	35.8<br>(26.7 to 47.7)	-6.0<br>(-25.3 to 18.4)	808.6<br>(595.7 to 1091.0)	-6.6<br>(-27.4 to 19.6)	3.4<br>(2.3 to 4.8)	-8.6<br>(-30.0 to 19.8)	70.0<br>(48.9 to 101.3)	-8.3<br>(-31.0 to 20.9)	25.3<br>(19.5 to 33.1)	-10.7<br>(-28.8 to 11.8)	574.2<br>(434.8 to 763.7)	-10.8<br>(-31.0 to 13.8)	11.0<br>(6.2 to 17.5)	9.7<br>(-12.8 to 39.6)	240.5<br>(139.8 to 378.3)	8.2<br>(-15.1 to 39.7)
Peru	27.6<br>(19.8 to 38.8)	-14.1<br>(-38.1 to 16.6)	662.8<br>(470.4 to 931.6)	-15.4<br>(-40.5 to 16.9)	3.9<br>(2.6 to 5.5)	-17.0<br>(-43.9 to 17.6)	87.0<br>(57.4 to 123.6)	-17.3<br>(-44.7 to 20.1)	18.4<br>(13.4 to 25.4)	-18.4<br>(-41.5 to 10.7)	453.7<br>(324.8 to 632.9)	-19.7<br>(-44.1 to 11.2)	7.7<br>(4.1 to 12.8)	1.5<br>(-26.1 to 37.7)	172.0<br>(93.6 to 280.1)	1.1<br>(-27.6 to 39.8)
**Caribbean**	**53.3<br>(44.6 to 62.7)**	**0.9<br>(-12.0 to 14.7)**	**1287.7<br>(1068.2 to 1514.6)**	**1.1<br>(-12.2 to 16.1)**	**5.3<br>(3.9 to 7.0)**	**-1.2<br>(-18.9 to 19.2)**	**124.8<br>(92.4 to 165.9)**	**-0.1<br>(-18.0 to 20.3)**	**43.0<br>(37.2 to 49.2)**	**-1.9<br>(-14.8 to 12.1)**	**1043.8<br>(891.5 to 1202.5)**	**-1.6<br>(-14.7 to 13.1)**	**13.1<br>(7.0 to 21.4)**	**13.9<br>(-0.9 to 31.3)**	**294.8<br>(162.4 to 472.8)**	**14.3<br>(-1.5 to 33.5)**
Antigua and Barbuda	41.4<br>(33.5 to 50.9)	3.6<br>(-9.4 to 17.8)	933.8<br>(756.2 to 1154.9)	2.9<br>(-11.9 to 18.8)	2.3<br>(1.5 to 3.4)	-3.2<br>(-23.5 to 20.9)	53.2<br>(35.4 to 76.5)	-3.9<br>(-24.1 to 20.8)	30.3<br>(26.2 to 35.4)	0.4<br>(-12.4 to 14.9)	690.2<br>(585.6 to 812.3)	-0.3<br>(-14.6 to 15.9)	13.4<br>(6.9 to 22.1)	14.9<br>(0.0 to 30.7)	287.1<br>(154.4 to 469.2)	14.8<br>(-1.7 to 33.0)
The Bahamas	48.3<br>(37.8 to 61.2)	-1.2<br>(-17.5 to 19.5)	1217.1<br>(941.5 to 1560.7)	-1.3<br>(-19.0 to 21.4)	3.8<br>(2.5 to 5.6)	-5.4<br>(-28.6 to 24.3)	94.3<br>(61.5 to 139.2)	-5.6<br>(-29.7 to 27.1)	35.1<br>(28.0 to 43.4)	-2.6<br>(-19.1 to 18.3)	896.6<br>(707.7 to 1131.9)	-2.8<br>(-20.7 to 19.6)	15.6<br>(8.3 to 25.3)	4.7<br>(-12.3 to 25.5)	368.2<br>(198.9 to 590.2)	5.0<br>(-13.5 to 27.3)
Barbados	51.7<br>(40.9 to 64.8)	3.1<br>(-13.9 to 21.2)	1201.2<br>(937.5 to 1507.1)	1.4<br>(-16.6 to 21.8)	2.8<br>(2.0 to 4.1)	5.9<br>(-18.3 to 36.3)	65.1<br>(45.1 to 93.5)	2.9<br>(-21.9 to 34.4)	36.1<br>(30.0 to 43.2)	2.1<br>(-14.4 to 20.1)	851.1<br>(696.2 to 1036.2)	-0.1<br>(-17.8 to 20.6)	18.7<br>(10.3 to 29.8)	7.4<br>(-10.1 to 25.7)	411.2<br>(230.8 to 649.7)	7.2<br>(-11.6 to 27.5)
Belize	40.1<br>(33.8 to 47.3)	-0.2<br>(-12.7 to 13.8)	1031.4<br>(874.2 to 1220.2)	-0.6<br>(-13.8 to 14.3)	5.1<br>(3.7 to 6.8)	5.2<br>(-13.4 to 28.0)	125.0<br>(91.0 to 163.7)	5.5<br>(-14.3 to 28.3)	30.6<br>(26.6 to 35.2)	-0.9<br>(-13.4 to 12.9)	793.0<br>(680.6 to 917.7)	-1.8<br>(-15.1 to 13.5)	9.8<br>(5.6 to 15.3)	5.2<br>(-8.6 to 20.1)	236.0<br>(139.8 to 358.7)	5.9<br>(-8.3 to 21.8)
Bermuda	51.0<br>(41.8 to 62.7)	-6.6<br>(-18.3 to 9.6)	1108.1<br>(906.5 to 1372.3)	-7.2<br>(-20.4 to 10.6)	6.8<br>(4.8 to 9.5)	-15.3<br>(-30.4 to 5.1)	139.7<br>(97.4 to 195.1)	-16.8<br>(-33.5 to 4.4)	38.1<br>(32.2 to 45.7)	-7.8<br>(-19.8 to 8.5)	837.0<br>(701.8 to 1009.2)	-8.5<br>(-21.4 to 8.9)	15.1<br>(8.3 to 24.3)	2.1<br>(-11.0 to 20.4)	315.6<br>(181.4 to 499.6)	1.7<br>(-12.6 to 21.1)
Cuba	67.8<br>(54.7 to 82.7)	0.1<br>(-16.7 to 20.5)	1571.0<br>(1259.3 to 1931.7)	-0.9<br>(-18.9 to 20.4)	7.4<br>(4.9 to 10.8)	-3.7<br>(-27.8 to 26.5)	173.0<br>(115.4 to 249.5)	-3.9<br>(-27.7 to 26.3)	56.9<br>(46.6 to 68.6)	-2.4<br>(-19.5 to 17.4)	1318.9<br>(1063.5 to 1601.9)	-3.4<br>(-21.5 to 17.9)	15.7<br>(8.3 to 26.3)	16.4<br>(-3.0 to 42.7)	347.2<br>(191.6 to 568.7)	16.2<br>(-4.7 to 43.7)
Dominica	56.4<br>(44.3 to 72.1)	4.3<br>(-9.8 to 22.0)	1336.0<br>(1040.6 to 1705.7)	3.9<br>(-11.7 to 23.3)	5.9<br>(4.1 to 8.3)	7.5<br>(-15.2 to 36.1)	133.3<br>(91.6 to 187.2)	7.0<br>(-17.0 to 37.4)	40.1<br>(32.8 to 49.4)	2.4<br>(-11.5 to 20.1)	976.0<br>(781.7 to 1222.1)	1.8<br>(-13.6 to 21.2)	17.9<br>(9.6 to 29.5)	11.1<br>(-4.0 to 29.6)	388.8<br>(209.3 to 626.9)	12.0<br>(-4.7 to 32.9)
Dominican Republic	44.7<br>(34.0 to 58.5)	12.2<br>(-16.4 to 51.1)	1066.5<br>(793.2 to 1427.8)	14.9<br>(-16.6 to 58.8)	4.0<br>(2.5 to 6.1)	13.9<br>(-20.8 to 64.5)	96.7<br>(58.1 to 148.7)	17.7<br>(-20.2 to 72.8)	37.7<br>(28.9 to 48.8)	8.3<br>(-18.9 to 46.1)	882.7<br>(659.5 to 1158.3)	10.5<br>(-20.2 to 54.1)	8.4<br>(4.2 to 14.2)	40.9<br>(5.3 to 93.4)	205.0<br>(107.3 to 338.1)	45.2<br>(5.1 to 104.8)
Grenada	56.3<br>(48.3 to 65.9)	-0.5<br>(-9.2 to 9.1)	1368.9<br>(1171.7 to 1587.5)	1.1<br>(-9.6 to 12.4)	4.3<br>(2.9 to 5.9)	-23.8<br>(-39.1 to -8.7)	103.3<br>(70.6 to 142.7)	-18.0<br>(-33.8 to -1.5)	40.3<br>(36.4 to 44.5)	-2.6<br>(-11.7 to 6.8)	996.9<br>(887.2 to 1116.0)	-0.8<br>(-10.9 to 10.6)	18.7<br>(10.1 to 29.1)	11.0<br>(1.0 to 23.2)	426.2<br>(237.9 to 651.8)	12.5<br>(1.1 to 25.5)
Guyana	43.0<br>(32.6 to 56.2)	1.5<br>(-20.8 to 27.7)	1137.1<br>(838.3 to 1512.1)	0.3<br>(-23.2 to 29.2)	2.9<br>(1.9 to 4.3)	-3.5<br>(-30.1 to 30.0)	75.7<br>(48.2 to 110.9)	-3.6<br>(-31.5 to 33.3)	31.4<br>(24.7 to 39.7)	-1.8<br>(-23.1 to 24.0)	849.9<br>(649.6 to 1097.4)	-2.9<br>(-26.0 to 26.1)	13.1<br>(6.7 to 22.4)	14.3<br>(-10.3 to 42.7)	316.4<br>(162.8 to 540.1)	14.2<br>(-12.3 to 45.9)
Haiti	44.8<br>(28.1 to 61.6)	-3.6<br>(-17.8 to 13.9)	1204.7<br>(733.8 to 1666.5)	-4.8<br>(-20.0 to 14.7)	5.7<br>(3.3 to 9.5)	-1.2<br>(-19.0 to 22.3)	135.7<br>(78.4 to 224.0)	-1.4<br>(-20.5 to 23.8)	34.9<br>(21.4 to 47.0)	-6.8<br>(-20.5 to 10.7)	968.5<br>(572.4 to 1338.9)	-7.9<br>(-23.1 to 11.0)	8.3<br>(3.3 to 16.1)	12.6<br>(-3.3 to 33.5)	193.1<br>(77.9 to 381.8)	12.1<br>(-4.4 to 34.9)
Jamaica	51.5<br>(39.8 to 66.2)	2.0<br>(-18.5 to 26.7)	1285.0<br>(986.3 to 1665.3)	2.4<br>(-19.8 to 29.1)	5.1<br>(3.5 to 7.2)	0.0<br>(-25.6 to 32.9)	125.8<br>(86.2 to 178.3)	0.6<br>(-25.8 to 35.5)	38.9<br>(31.0 to 48.7)	-1.9<br>(-21.4 to 21.9)	987.6<br>(776.5 to 1254.0)	-1.3<br>(-22.5 to 25.0)	15.2<br>(8.1 to 25.4)	14.3<br>(-8.6 to 39.8)	348.2<br>(186.0 to 579.7)	14.6<br>(-10.0 to 42.1)
Puerto Rico	34.6<br>(25.4 to 45.7)	-5.9<br>(-25.5 to 19.1)	812.1<br>(595.2 to 1077.2)	-4.6<br>(-25.9 to 22.7)	1.8<br>(1.1 to 2.8)	-4.8<br>(-33.0 to 33.6)	37.1<br>(22.2 to 60.3)	-3.5<br>(-33.3 to 37.1)	24.2<br>(18.6 to 30.7)	-8.5<br>(-28.1 to 15.9)	572.4<br>(435.1 to 735.4)	-6.9<br>(-27.9 to 19.8)	13.9<br>(7.6 to 22.9)	-1.1<br>(-21.7 to 25.6)	315.6<br>(176.2 to 515.7)	-0.1<br>(-21.8 to 28.2)
Saint Kitts and Nevis	49.1<br>(39.0 to 61.7)	8.0<br>(-5.9 to 24.7)	1143.4<br>(878.4 to 1456.3)	8.0<br>(-10.5 to 28.3)	4.1<br>(3.1 to 5.2)	14.7<br>(-4.2 to 37.4)	92.7<br>(67.3 to 122.8)	19.9<br>(-3.4 to 47.2)	33.9<br>(28.3 to 41.0)	4.6<br>(-9.9 to 22.7)	803.2<br>(629.4 to 1005.8)	4.5<br>(-14.4 to 26.2)	17.3<br>(9.1 to 27.8)	18.6<br>(3.9 to 37.1)	382.7<br>(206.2 to 612.6)	19.9<br>(2.2 to 41.1)
Saint Lucia	46.3<br>(37.6 to 57.7)	14.2<br>(-2.7 to 32.1)	1132.5<br>(917.3 to 1411.5)	10.2<br>(-7.7 to 29.2)	3.5<br>(2.3 to 4.9)	18.7<br>(-5.7 to 48.2)	83.3<br>(56.8 to 118.0)	15.3<br>(-8.4 to 44.0)	34.6<br>(29.4 to 40.9)	10.1<br>(-6.1 to 27.1)	861.6<br>(723.7 to 1031.1)	6.3<br>(-11.0 to 25.1)	14.2<br>(7.4 to 23.5)	26.6<br>(8.8 to 47.0)	319.9<br>(167.9 to 525.9)	24.4<br>(5.7 to 46.8)
Saint Vincent and the Grenadines	48.6<br>(40.5 to 58.8)	11.9<br>(-2.1 to 28.8)	1223.9<br>(1011.5 to 1472.3)	10.9<br>(-5.0 to 29.3)	3.1<br>(2.1 to 4.2)	14.7<br>(-7.2 to 40.2)	74.4<br>(51.0 to 102.4)	13.6<br>(-9.0 to 39.9)	36.2<br>(31.6 to 41.5)	9.8<br>(-3.9 to 26.6)	933.3<br>(802.3 to 1088.1)	8.5<br>(-7.3 to 27.1)	14.3<br>(7.5 to 23.0)	21.6<br>(5.7 to 40.5)	326.4<br>(176.5 to 519.7)	23.0<br>(5.8 to 44.2)
Suriname	47.8<br>(39.1 to 58.9)	7.4<br>(-9.4 to 26.9)	1197.3<br>(973.5 to 1474.7)	8.0<br>(-9.9 to 29.9)	4.3<br>(2.9 to 6.0)	2.4<br>(-21.0 to 30.1)	106.1<br>(71.8 to 147.6)	2.6<br>(-21.3 to 31.5)	37.9<br>(31.8 to 45.2)	4.1<br>(-12.4 to 23.4)	963.2<br>(802.3 to 1160.8)	5.1<br>(-13.2 to 26.4)	12.9<br>(6.4 to 22.3)	20.9<br>(1.4 to 43.0)	295.9<br>(151.2 to 501.4)	21.0<br>(0.3 to 44.0)
Trinidad and Tobago	39.7<br>(29.1 to 53.5)	-4.7<br>(-27.6 to 23.1)	968.3<br>(698.7 to 1313.0)	-5.6<br>(-29.9 to 24.7)	2.9<br>(1.7 to 4.5)	-11.2<br>(-37.9 to 23.1)	73.1<br>(44.5 to 115.1)	-10.7<br>(-37.9 to 25.3)	28.2<br>(21.6 to 37.0)	-7.4<br>(-29.7 to 19.7)	699.4<br>(520.1 to 930.6)	-8.3<br>(-32.1 to 21.5)	14.2<br>(7.5 to 24.4)	3.8<br>(-20.1 to 33.4)	327.1<br>(174.2 to 556.9)	3.8<br>(-21.9 to 35.5)
United States Virgin Islands	61.5<br>(49.7 to 74.7)	-4.1<br>(-15.9 to 7.7)	1409.4<br>(1113.0 to 1731.1)	-4.2<br>(-18.0 to 10.3)	7.5<br>(5.5 to 9.7)	0.1<br>(-17.0 to 19.5)	169.9<br>(120.7 to 224.4)	1.1<br>(-17.1 to 22.1)	41.6<br>(34.4 to 48.6)	-7.7<br>(-21.1 to 6.4)	961.9<br>(773.8 to 1153.9)	-8.0<br>(-23.5 to 8.1)	22.6<br>(12.7 to 35.3)	3.5<br>(-8.3 to 14.2)	496.9<br>(283.7 to 771.0)	3.8<br>(-9.8 to 16.4)
**Central Latin America**	**32.6<br>(26.4 to 40.2)**	**-3.6<br>(-16.1 to 10.6)**	**785.8<br>(634.7 to 965.8)**	**-3.0<br>(-16.5 to 12.1)**	**3.9<br>(3.0 to 4.9)**	**-8.3<br>(-21.7 to 8.4)**	**88.6<br>(69.4 to 111.6)**	**-7.9<br>(-21.8 to 10.3)**	**23.5<br>(19.9 to 27.9)**	**-8.0<br>(-20.0 to 5.9)**	**572.3<br>(479.0 to 684.3)**	**-7.3<br>(-20.4 to 8.0)**	**9.7<br>(5.2 to 15.8)**	**10.7<br>(-2.4 to 26.3)**	**220.6<br>(121.5 to 352.2)**	**11.9<br>(-2.3 to 28.1)**
Colombia	28.9<br>(21.4 to 39.0)	-10.9<br>(-30.2 to 12.0)	692.8<br>(508.6 to 932.8)	-10.8<br>(-31.6 to 14.0)	3.8<br>(2.5 to 5.4)	-14.4<br>(-35.6 to 13.0)	85.0<br>(57.4 to 120.2)	-14.2<br>(-36.6 to 14.9)	20.9<br>(16.1 to 27.5)	-14.8<br>(-33.3 to 7.8)	512.2<br>(388.4 to 677.6)	-14.7<br>(-34.5 to 9.1)	8.0<br>(4.1 to 14.1)	1.2<br>(-20.8 to 27.6)	178.3<br>(92.0 to 307.1)	2.2<br>(-21.1 to 29.7)
Costa Rica	37.0<br>(27.2 to 50.1)	-3.5<br>(-23.7 to 21.1)	842.6<br>(618.5 to 1141.5)	-3.1<br>(-24.8 to 24.0)	2.6<br>(1.7 to 3.8)	-7.9<br>(-30.5 to 20.2)	56.0<br>(38.0 to 81.4)	-8.4<br>(-31.6 to 21.0)	27.6<br>(21.3 to 36.3)	-7.4<br>(-26.7 to 16.1)	635.3<br>(483.2 to 842.8)	-6.8<br>(-27.8 to 19.1)	11.5<br>(6.0 to 19.5)	10.3<br>(-13.1 to 38.7)	249.9<br>(132.9 to 414.8)	10.7<br>(-14.6 to 41.3)
El Salvador	30.7<br>(22.2 to 42.4)	5.9<br>(-18.6 to 35.9)	778.3<br>(559.6 to 1065.9)	3.5<br>(-22.5 to 35.5)	3.0<br>(1.9 to 4.5)	-4.6<br>(-31.1 to 26.3)	71.2<br>(47.1 to 107.8)	-4.8<br>(-31.7 to 27.2)	22.5<br>(16.6 to 30.0)	3.8<br>(-20.7 to 33.5)	584.6<br>(424.6 to 791.1)	1.3<br>(-24.5 to 32.7)	8.3<br>(4.2 to 14.2)	19.4<br>(-10.3 to 53.1)	189.5<br>(97.2 to 323.0)	18.3<br>(-12.3 to 53.1)
Guatemala	33.5<br>(24.7 to 45.8)	-10.3<br>(-30.4 to 15.9)	825.1<br>(603.4 to 1132.9)	-10.8<br>(-33.0 to 18.2)	3.1<br>(2.2 to 4.2)	-15.0<br>(-36.0 to 11.5)	70.7<br>(49.8 to 96.7)	-15.1<br>(-37.6 to 13.4)	25.9<br>(19.6 to 34.9)	-13.4<br>(-33.3 to 11.6)	647.4<br>(479.0 to 880.7)	-13.9<br>(-35.0 to 13.9)	7.6<br>(3.6 to 13.3)	6.4<br>(-17.4 to 36.5)	171.7<br>(84.4 to 297.4)	7.9<br>(-18.3 to 41.4)
Honduras	47.9<br>(37.2 to 61.0)	9.5<br>(-8.2 to 33.7)	1124.5<br>(864.5 to 1448.0)	7.6<br>(-12.8 to 35.4)	9.7<br>(6.1 to 14.4)	10.3<br>(-10.8 to 37.3)	224.0<br>(139.5 to 335.5)	8.9<br>(-14.1 to 40.1)	34.0<br>(27.1 to 42.5)	6.2<br>(-10.4 to 28.9)	803.7<br>(625.3 to 1021.0)	4.1<br>(-15.4 to 30.8)	12.5<br>(6.2 to 21.1)	20.8<br>(1.6 to 46.9)	277.7<br>(139.3 to 468.7)	20.6<br>(-1.5 to 51.8)
Mexico	30.9<br>(25.1 to 37.8)	-2.7<br>(-15.4 to 10.7)	732.0<br>(597.8 to 893.8)	-1.5<br>(-14.8 to 13.3)	3.5<br>(2.8 to 4.3)	-8.4<br>(-20.9 to 7.1)	78.3<br>(62.9 to 96.4)	-7.4<br>(-20.7 to 9.0)	21.4<br>(18.0 to 25.2)	-8.3<br>(-20.1 to 4.6)	507.3<br>(426.0 to 605.6)	-6.8<br>(-19.3 to 6.9)	10.4<br>(5.7 to 16.6)	12.4<br>(-1.9 to 28.6)	238.8<br>(134.4 to 373.1)	13.6<br>(-1.6 to 30.8)
Nicaragua	34.6<br>(27.1 to 43.8)	-4.5<br>(-20.3 to 12.0)	784.4<br>(604.9 to 1008.4)	-4.1<br>(-21.8 to 16.4)	3.2<br>(2.3 to 4.3)	-8.8<br>(-25.8 to 10.8)	67.7<br>(49.6 to 92.1)	-6.8<br>(-25.9 to 15.6)	26.2<br>(21.1 to 32.3)	-8.1<br>(-23.3 to 8.0)	605.6<br>(477.3 to 758.6)	-7.9<br>(-25.4 to 11.8)	8.9<br>(4.5 to 14.7)	13.0<br>(-4.3 to 31.9)	187.0<br>(95.6 to 305.0)	15.8<br>(-3.9 to 39.1)
Panama	30.1<br>(22.2 to 40.5)	-4.0<br>(-24.9 to 21.0)	717.5<br>(523.9 to 961.3)	-3.5<br>(-25.9 to 24.4)	2.6<br>(1.7 to 3.9)	-13.7<br>(-35.2 to 14.8)	59.0<br>(39.3 to 88.5)	-11.6<br>(-34.3 to 18.1)	22.4<br>(17.1 to 29.0)	-9.4<br>(-29.2 to 14.4)	543.1<br>(407.3 to 708.7)	-8.6<br>(-30.3 to 17.6)	8.7<br>(4.4 to 15.2)	17.9<br>(-7.0 to 49.8)	191.8<br>(98.5 to 330.4)	19.0<br>(-8.3 to 53.2)
Venezuela	43.5<br>(32.3 to 58.4)	1.0<br>(-22.4 to 29.2)	1086.4<br>(795.3 to 1464.0)	1.5<br>(-23.5 to 32.1)	5.4<br>(3.5 to 8.0)	-5.6<br>(-30.9 to 23.9)	130.8<br>(84.9 to 191.5)	-6.0<br>(-31.6 to 25.5)	33.6<br>(25.5 to 43.8)	-2.1<br>(-24.6 to 25.0)	853.5<br>(635.0 to 1129.3)	-1.3<br>(-25.4 to 28.4)	10.5<br>(5.0 to 19.0)	13.9<br>(-11.5 to 44.2)	239.3<br>(115.0 to 429.2)	14.6<br>(-12.5 to 47.6)
**Tropical Latin America**	**44.5<br>(40.2 to 49.8)**	**-12.0<br>(-15.2 to -8.8)**	**1072.8<br>(979.9 to 1186.1)**	**-13.1<br>(-16.4 to -9.8)**	**4.6<br>(3.8 to 5.6)**	**-15.5<br>(-21.0 to -10.0)**	**104.2<br>(85.2 to 124.7)**	**-17.6<br>(-23.1 to -12.1)**	**35.9<br>(33.0 to 38.6)**	**-15.6<br>(-18.6 to -12.6)**	**876.2<br>(819.5 to 936.0)**	**-16.3<br>(-19.4 to -13.3)**	**10.5<br>(6.0 to 16.1)**	**2.4<br>(-1.7 to 7.4)**	**236.1<br>(139.8 to 357.3)**	**1.0<br>(-3.1 to 5.9)**
Brazil	44.4<br>(40.0 to 49.7)	-12.3<br>(-15.4 to -9.0)	1070.8<br>(976.5 to 1184.8)	-13.4<br>(-16.5 to -10.1)	4.6<br>(3.8 to 5.6)	-15.7<br>(-21.2 to -10.2)	104.0<br>(85.1 to 124.9)	-17.8<br>(-23.4 to -12.3)	35.8<br>(33.0 to 38.5)	-15.8<br>(-18.8 to -12.8)	873.7<br>(818.0 to 933.4)	-16.6<br>(-19.6 to -13.5)	10.6<br>(6.0 to 16.1)	2.1<br>(-2.0 to 7.2)	236.5<br>(140.0 to 357.5)	0.7<br>(-3.4 to 5.4)
Paraguay	48.2<br>(37.1 to 62.5)	-3.0<br>(-26.0 to 24.1)	1154.7<br>(870.0 to 1512.6)	-2.9<br>(-27.6 to 26.8)	4.8<br>(3.3 to 6.7)	-10.0<br>(-34.5 to 20.6)	111.1<br>(75.2 to 155.5)	-10.3<br>(-35.7 to 22.9)	40.6<br>(31.2 to 51.7)	-5.9<br>(-28.3 to 19.7)	977.3<br>(731.1 to 1269.0)	-5.6<br>(-29.9 to 23.5)	9.8<br>(5.0 to 16.6)	17.8<br>(-9.8 to 50.5)	217.2<br>(111.9 to 367.3)	17.5<br>(-12.0 to 51.7)
**North Africa and Middle East**	**39.1<br>(33.7 to 45.2)**	**1.1<br>(-7.6 to 10.8)**	**908.3<br>(782.2 to 1054.1)**	**-0.5<br>(-9.6 to 9.4)**	**6.5<br>(5.2 to 7.9)**	**-5.7<br>(-19.0 to 10.9)**	**150.5<br>(120.9 to 183.2)**	**-7.5<br>(-20.9 to 8.7)**	**29.9<br>(26.5 to 33.6)**	**-1.9<br>(-10.6 to 7.5)**	**695.0<br>(614.0 to 784.7)**	**-3.6<br>(-12.4 to 6.2)**	**11.1<br>(6.2 to 17.5)**	**14.0<br>(4.4 to 24.2)**	**251.6<br>(145.3 to 393.1)**	**13.1<br>(2.6 to 23.8)**
**North Africa and Middle East**	**39.1<br>(33.7 to 45.2)**	**1.1<br>(-7.6 to 10.8)**	**908.3<br>(782.2 to 1054.1)**	**-0.5<br>(-9.6 to 9.4)**	**6.5<br>(5.2 to 7.9)**	**-5.7<br>(-19.0 to 10.9)**	**150.5<br>(120.9 to 183.2)**	**-7.5<br>(-20.9 to 8.7)**	**29.9<br>(26.5 to 33.6)**	**-1.9<br>(-10.6 to 7.5)**	**695.0<br>(614.0 to 784.7)**	**-3.6<br>(-12.4 to 6.2)**	**11.1<br>(6.2 to 17.5)**	**14.0<br>(4.4 to 24.2)**	**251.6<br>(145.3 to 393.1)**	**13.1<br>(2.6 to 23.8)**
Afghanistan	37.9<br>(27.7 to 49.5)	4.9<br>(-11.9 to 22.6)	947.6<br>(649.5 to 1274.2)	3.5<br>(-14.3 to 23.6)	5.4<br>(3.2 to 8.7)	-7.2<br>(-26.0 to 18.5)	133.0<br>(73.3 to 220.1)	-6.9<br>(-26.9 to 20.4)	27.4<br>(20.2 to 35.4)	1.4<br>(-14.6 to 18.5)	687.6<br>(477.8 to 922.1)	0.2<br>(-16.8 to 19.1)	10.9<br>(5.4 to 18.5)	28.1<br>(7.5 to 57.7)	262.8<br>(132.8 to 448.4)	26.2<br>(4.2 to 57.6)
Algeria	27.7<br>(22.2 to 34.2)	-1.7<br>(-19.1 to 19.6)	612.7<br>(482.7 to 764.0)	-3.0<br>(-21.2 to 20.3)	2.9<br>(2.0 to 4.3)	-3.3<br>(-23.6 to 23.1)	67.0<br>(44.5 to 97.8)	-5.1<br>(-26.2 to 22.7)	20.9<br>(16.9 to 25.5)	-5.1<br>(-22.8 to 16.4)	463.0<br>(368.4 to 572.0)	-6.5<br>(-25.0 to 16.8)	8.6<br>(4.7 to 13.7)	12.6<br>(-6.5 to 36.9)	179.2<br>(97.9 to 286.4)	12.2<br>(-8.4 to 39.1)
Bahrain	48.4	-14.6	903.3	-14.8	10.7	-23.7	183.9	-24.0	31.9	-16.5	589.5	-16.8	20.9	-9.6	390.0	-9.5

Location	All risk factors*				Environmental and occupational risks*				Behavioural risks*				Metabolic risks*			
	Death ASR	Death % change	DALY ASR	DALY % change	Death ASR	Death % change	DALY ASR	DALY % change	Death ASR	Death % change	DALY ASR	DALY % change	Death ASR	Death % change	DALY ASR	DALY % change
	(37.6 to 61.6)	(-29.0 to 1.4)	(695.7 to 1158.9)	(-30.1 to 3.0)	(7.4 to 14.4)	(-42.2 to -2.0)	(128.3 to 250.0)	(-42.7 to -1.8)	(25.3 to 39.3)	(-31.4 to 0.3)	(462.3 to 730.9)	(-32.6 to 1.3)	(10.7 to 34.3)	(-24.9 to 7.4)	(205.8 to 628.8)	(-25.3 to 9.2)
Egypt	35.3	1.9	883.6	0.9	3.4	1.1	87.3	0.7	25.3	0.3	628.4	-0.8	12.6	9.6	317.7	8.3
	(25.4 to 47.6)	(-20.4 to 27.8)	(633.2 to 1217.0)	(-22.1 to 29.0)	(2.2 to 4.8)	(-23.6 to 30.5)	(57.4 to 125.4)	(-25.0 to 32.7)	(18.7 to 33.7)	(-21.5 to 25.6)	(455.6 to 847.8)	(-23.6 to 26.3)	(6.8 to 20.6)	(-15.1 to 39.0)	(172.1 to 518.9)	(-17.5 to 38.2)
Iran	30.8	11.7	684.9	10.9	3.9	7.5	89.9	6.7	23.3	8.1	516.9	7.5	8.8	28.6	190.7	27.9
	(27.3 to 35.5)	(6.2 to 18.8)	(606.8 to 784.3)	(5.4 to 18.2)	(3.2 to 4.8)	(0.5 to 15.7)	(72.7 to 109.1)	(-0.2 to 15.1)	(21.3 to 26.0)	(2.7 to 15.5)	(476.4 to 576.8)	(2.1 to 15.1)	(4.9 to 13.6)	(19.4 to 39.6)	(107.7 to 293.7)	(18.5 to 38.3)
Iraq	43.3	14.8	992.6	11.1	7.6	10.1	170.1	5.9	33.4	11.6	756.9	7.9	12.7	29.9	289.8	26.3
	(34.1 to 53.4)	(-2.5 to 35.0)	(753.5 to 1270.9)	(-6.6 to 33.0)	(5.4 to 10.0)	(-10.2 to 36.4)	(115.7 to 230.1)	(-15.2 to 32.0)	(26.2 to 39.6)	(-5.9 to 31.8)	(584.6 to 928.0)	(-10.0 to 29.4)	(6.5 to 21.5)	(10.2 to 52.8)	(146.9 to 491.1)	(5.6 to 51.6)
Jordan	39.2	-5.1	858.2	-4.7	5.2	-11.9	111.9	-11.5	29.8	-7.2	652.4	-6.4	13.1	2.9	281.9	2.4
	(32.3 to 47.5)	(-20.2 to 11.8)	(706.1 to 1041.3)	(-20.7 to 13.1)	(3.8 to 7.0)	(-30.9 to 10.3)	(81.3 to 150.4)	(-31.3 to 12.2)	(24.7 to 35.4)	(-23.1 to 10.5)	(536.3 to 777.1)	(-23.2 to 12.5)	(7.6 to 20.3)	(-13.0 to 22.5)	(164.7 to 433.3)	(-14.0 to 22.8)
Kuwait	29.7	-1.4	601.5	-4.8	4.8	11.9	93.1	5.1	20.2	-3.0	403.8	-6.6	12.2	2.5	248.2	-0.7
	(23.8 to 36.8)	(-15.6 to 15.5)	(487.1 to 745.2)	(-19.0 to 12.0)	(3.6 to 6.2)	(-11.0 to 40.0)	(69.6 to 120.0)	(-15.9 to 31.6)	(16.6 to 24.1)	(-18.6 to 15.1)	(332.4 to 484.5)	(-22.1 to 11.4)	(7.0 to 18.8)	(-11.3 to 19.8)	(147.0 to 374.2)	(-14.2 to 17.1)
Lebanon	65.1	0.3	1461.0	2.0	7.6	-9.8	179.7	-8.4	52.8	-1.4	1184.4	0.4	18.6	12.5	399.0	14.4
	(53.5 to 84.6)	(-11.5 to 13.8)	(1189.6 to 1883.6)	(-12.6 to 18.2)	(5.2 to 11.2)	(-23.0 to 5.6)	(123.1 to 264.2)	(-22.7 to 8.5)	(44.0 to 68.9)	(-13.6 to 12.0)	(972.3 to 1522.1)	(-14.0 to 16.7)	(9.6 to 31.5)	(-1.6 to 29.2)	(211.4 to 674.0)	(-2.8 to 34.4)
Libya	45.0	-0.4	1071.1	0.0	6.1	-12.0	146.3	-11.6	33.6	-0.7	801.2	-0.2	15.1	4.3	347.9	4.5
	(34.2 to 56.9)	(-21.0 to 22.5)	(808.8 to 1366.7)	(-20.9 to 24.5)	(4.0 to 8.7)	(-32.5 to 13.3)	(96.4 to 211.7)	(-32.8 to 14.8)	(25.7 to 41.5)	(-21.2 to 23.0)	(609.0 to 1002.9)	(-21.6 to 25.7)	(7.9 to 25.6)	(-16.3 to 26.8)	(179.8 to 583.9)	(-16.6 to 28.9)
Morocco	34.3	10.7	851.0	6.8	5.6	13.0	140.7	9.7	26.0	5.5	656.8	1.8	9.0	33.6	204.9	32.0
	(25.5 to 42.5)	(-9.1 to 34.9)	(623.3 to 1073.4)	(-13.5 to 31.1)	(3.7 to 7.9)	(-11.6 to 44.3)	(92.9 to 204.0)	(-14.9 to 40.9)	(19.7 to 31.7)	(-13.1 to 29.4)	(490.9 to 808.8)	(-17.5 to 25.4)	(4.3 to 15.5)	(10.6 to 61.6)	(98.0 to 358.9)	(8.6 to 61.2)
Oman	27.9	-13.9	581.8	-20.5	4.8	-25.0	98.2	-29.9	16.6	-18.3	342.1	-25.2	11.7	-0.9	245.9	-7.1
	(22.8 to 34.2)	(-24.0 to -2.0)	(463.5 to 724.0)	(-31.4 to -6.0)	(3.7 to 6.2)	(-39.2 to -4.6)	(73.1 to 131.2)	(-44.6 to -7.6)	(14.2 to 19.5)	(-28.1 to -6.3)	(286.7 to 414.6)	(-35.8 to -11.0)	(6.7 to 17.9)	(-12.6 to 12.0)	(144.1 to 374.3)	(-19.8 to 8.4)
Palestine	52.4	14.6	1171.4	14.2	6.8	9.8	160.6	10.1	40.7	10.4	904.8	10.3	16.4	34.2	360.2	32.8
	(43.7 to 62.8)	(-2.5 to 34.0)	(973.9 to 1414.3)	(-3.2 to 34.4)	(4.9 to 9.1)	(-9.9 to 30.0)	(117.5 to 214.9)	(-9.7 to 32.1)	(34.7 to 46.6)	(-5.8 to 27.7)	(766.6 to 1043.8)	(-7.0 to 28.9)	(7.8 to 27.8)	(14.0 to 58.6)	(177.4 to 597.9)	(12.7 to 57.5)
Qatar	60.4	-9.5	1074.1	-15.9	8.2	-14.8	155.0	-20.4	36.9	-9.1	653.7	-15.6	30.5	-8.4	537.9	-14.9
	(46.7 to 76.7)	(-25.8 to 9.8)	(816.4 to 1389.3)	(-32.6 to 4.2)	(5.9 to 11.1)	(-35.2 to 11.4)	(107.8 to 217.2)	(-42.5 to 7.6)	(28.9 to 45.5)	(-27.1 to 12.2)	(499.3 to 829.8)	(-33.7 to 6.2)	(17.7 to 47.1)	(-23.8 to 11.2)	(310.2 to 838.4)	(-30.3 to 4.9)
Saudi Arabia	26.1	-2.1	609.3	-0.7	3.1	-7.0	73.4	-6.6	16.6	-4.7	389.7	-3.8	11.6	6.0	262.8	8.5
	(20.7 to 32.6)	(-15.8 to 14.2)	(475.4 to 773.7)	(-16.7 to 17.7)	(2.3 to 4.0)	(-24.2 to 12.0)	(51.7 to 94.8)	(-25.7 to 14.0)	(13.6 to 20.0)	(-18.0 to 11.6)	(312.0 to 480.6)	(-19.6 to 13.9)	(6.9 to 17.4)	(-9.0 to 23.7)	(158.6 to 394.4)	(-8.9 to 28.2)
Sudan	28.5	7.4	660.7	4.9	3.3	-2.0	79.1	-3.2	22.1	2.0	509.9	-0.6	7.9	36.5	180.3	34.5
	(19.8 to 37.9)	(-7.9 to 26.6)	(452.1 to 902.9)	(-12.2 to 25.7)	(2.0 to 5.4)	(-21.4 to 23.5)	(48.3 to 126.6)	(-23.8 to 25.1)	(15.8 to 29.0)	(-12.7 to 20.7)	(353.7 to 688.0)	(-16.2 to 19.0)	(4.0 to 13.7)	(15.6 to 64.9)	(90.7 to 311.8)	(11.9 to 63.4)
Syria	27.7	-4.1	631.7	-3.0	3.4	-10.5	82.2	-9.7	21.3	-6.7	481.6	-5.6	8.0	6.3	179.3	7.1
	(20.4 to 36.2)	(-25.5 to 22.3)	(456.7 to 845.3)	(-26.7 to 26.1)	(2.4 to 4.8)	(-33.0 to 15.4)	(57.0 to 118.6)	(-33.9 to 19.7)	(16.1 to 27.5)	(-27.3 to 19.7)	(352.9 to 636.7)	(-28.4 to 23.2)	(4.1 to 13.4)	(-17.7 to 35.4)	(90.9 to 304.6)	(-19.2 to 40.3)
Tunisia	39.0	-1.6	890.7	-1.5	5.9	-6.8	143.0	-7.2	31.8	-4.0	729.8	-3.8	10.4	8.5	220.5	9.8
	(28.7 to 53.1)	(-24.3 to 25.6)	(642.5 to 1233.0)	(-25.3 to 28.3)	(3.8 to 8.9)	(-30.3 to 24.6)	(91.9 to 217.5)	(-31.3 to 25.4)	(23.5 to 42.8)	(-26.0 to 23.1)	(527.3 to 992.3)	(-27.0 to 25.6)	(5.0 to 19.3)	(-14.1 to 39.6)	(104.7 to 416.6)	(-14.2 to 43.1)
Turkey	57.0	-5.6	1345.6	-7.1	14.2	-11.5	333.1	-13.1	45.6	-8.0	1088.9	-9.4	13.0	3.4	287.8	2.8
	(45.4 to 70.7)	(-25.7 to 20.1)	(1058.9 to 1683.0)	(-27.6 to 19.8)	(10.1 to 19.1)	(-35.3 to 20.2)	(236.4 to 453.5)	(-36.8 to 20.0)	(36.3 to 55.9)	(-27.8 to 17.2)	(863.0 to 1348.4)	(-29.4 to 16.9)	(6.7 to 21.7)	(-17.3 to 31.2)	(151.1 to 474.4)	(-18.7 to 32.6)
United Arab Emirates	59.3	-20.0	1275.0	-16.7	6.6	-18.3	138.7	-15.5	39.0	-19.9	823.2	-17.3	26.9	-17.9	583.6	-14.2
	(46.6 to 76.0)	(-33.6 to -2.6)	(969.9 to 1671.1)	(-31.3 to 1.7)	(4.2 to 9.7)	(-37.2 to 7.0)	(89.9 to 199.9)	(-35.3 to 10.6)	(30.9 to 47.5)	(-33.6 to -3.2)	(640.2 to 1028.8)	(-32.2 to 0.9)	(15.4 to 42.8)	(-32.6 to 0.8)	(334.6 to 928.2)	(-30.2 to 6.4)
Yemen	31.6	1.4	751.4	1.4	3.8	-4.8	91.0	-4.8	27.8	0.4	659.0	0.2	4.7	12.1	109.9	13.9
	(24.0 to 41.5)	(-13.7 to 18.8)	(556.8 to 1003.7)	(-15.3 to 21.2)	(2.4 to 5.9)	(-22.9 to 18.1)	(58.0 to 144.2)	(-23.9 to 19.3)	(21.1 to 36.2)	(-14.5 to 18.1)	(489.2 to 875.7)	(-16.3 to 20.0)	(2.1 to 8.8)	(-4.8 to 32.6)	(49.3 to 200.4)	(-5.3 to 37.3)
**South Asia**	**32.4**	**3.4**	**808.8**	**2.3**	**4.1**	**5.2**	**97.5**	**4.0**	**27.1**	**-0.4**	**681.3**	**-1.4**	**5.1**	**34.0**	**118.6**	**34.4**
	**(27.8 to 37.6)**	**(-8.0 to 16.6)**	**(695.7 to 939.6)**	**(-9.4 to 15.5)**	**(3.3 to 4.9)**	**(-10.4 to 20.4)**	**(78.6 to 117.7)**	**(-11.7 to 19.9)**	**(23.7 to 31.0)**	**(-11.6 to 12.9)**	**(596.2 to 782.9)**	**(-12.7 to 11.8)**	**(2.3 to 8.7)**	**(18.0 to 54.8)**	**(54.9 to 199.6)**	**(17.6 to 55.7)**
**South Asia**	**32.4**	**3.4**	**808.8**	**2.3**	**4.1**	**5.2**	**97.5**	**4.0**	**27.1**	**-0.4**	**681.3**	**-1.4**	**5.1**	**34.0**	**118.6**	**34.4**
	**(27.8 to 37.6)**	**(-8.0 to 16.6)**	**(695.7 to 939.6)**	**(-9.4 to 15.5)**	**(3.3 to 4.9)**	**(-10.4 to 20.4)**	**(78.6 to 117.7)**	**(-11.7 to 19.9)**	**(23.7 to 31.0)**	**(-11.6 to 12.9)**	**(596.2 to 782.9)**	**(-12.7 to 11.8)**	**(2.3 to 8.7)**	**(18.0 to 54.8)**	**(54.9 to 199.6)**	**(17.6 to 55.7)**
Bangladesh	26.7	-7.1	644.7	-7.1	3.4	-7.5	81.1	-7.3	23.5	-9.1	567.6	-9.1	3.0	15.9	68.4	18.2
	(19.8 to 36.6)	(-24.9 to 11.8)	(470.4 to 890.8)	(-25.7 to 12.8)	(2.2 to 5.5)	(-33.3 to 21.1)	(51.6 to 132.8)	(-33.1 to 20.2)	(17.5 to 31.8)	(-26.5 to 9.6)	(417.8 to 779.2)	(-27.6 to 10.5)	(1.1 to 5.7)	(-6.2 to 44.8)	(27.4 to 128.3)	(-5.5 to 51.4)
Bhutan	29.1	4.4	680.4	1.9	3.7	6.1	80.9	4.0	23.6	0.8	551.5	-1.5	5.3	29.5	119.1	26.3
	(22.8 to 36.4)	(-10.7 to 19.1)	(505.1 to 869.8)	(-14.9 to 20.5)	(2.5 to 5.3)	(-12.5 to 26.6)	(55.8 to 116.7)	(-15.5 to 26.2)	(18.4 to 28.7)	(-13.5 to 15.9)	(413.0 to 703.0)	(-17.4 to 16.5)	(2.5 to 9.1)	(11.3 to 53.2)	(56.6 to 204.3)	(6.0 to 51.5)
India	31.1	6.8	775.8	4.9	3.8	9.7	89.8	7.9	26.0	2.7	654.4	1.0	4.9	42.9	112.1	42.6
	(26.1 to 37.1)	(-7.8 to 23.7)	(648.0 to 929.2)	(-9.7 to 22.0)	(3.0 to 4.7)	(-9.0 to 29.8)	(71.1 to 111.9)	(-11.1 to 27.9)	(22.4 to 30.6)	(-11.7 to 19.0)	(557.3 to 774.7)	(-13.4 to 17.7)	(2.2 to 8.3)	(22.0 to 68.7)	(51.5 to 188.1)	(21.5 to 69.4)
Nepal	33.6	11.5	798.7	9.5	4.3	15.6	97.8	13.4	29.0	7.1	685.0	5.1	4.5	53.8	102.1	54.4
	(26.2 to 41.1)	(-7.4 to 30.3)	(613.3 to 981.6)	(-9.6 to 30.4)	(2.9 to 5.8)	(-9.8 to 42.5)	(66.4 to 133.3)	(-11.2 to 42.4)	(22.7 to 34.7)	(-11.0 to 25.1)	(526.4 to 836.7)	(-13.5 to 25.4)	(1.9 to 8.0)	(26.0 to 89.9)	(44.0 to 180.7)	(25.6 to 94.6)
Pakistan	53.3	-6.1	1332.7	-6.5	7.7	-6.5	193.0	-7.0	43.1	-10.1	1083.2	-10.3	10.7	12.1	249.5	11.7
	(42.6 to 66.1)	(-24.4 to 18.5)	(1066.6 to 1663.5)	(-26.1 to 19.9)	(5.6 to 10.2)	(-29.4 to 24.3)	(139.0 to 258.8)	(-30.8 to 25.9)	(35.1 to 52.3)	(-29.1 to 15.0)	(881.6 to 1330.4)	(-29.7 to 16.8)	(4.8 to 18.9)	(-6.8 to 39.3)	(113.5 to 438.3)	(-8.6 to 40.6)
**Southeast Asia, East Asia, and Oceania**	**65.2**	**-7.1**	**1484.5**	**-7.7**	**12.5**	**-9.4**	**274.2**	**-10.6**	**56.2**	**-8.4**	**1271.3**	**-9.2**	**9.5**	**4.1**	**218.1**	**6.0**
	**(55.3 to 75.8)**	**(-20.1 to 7.5)**	**(1263.4 to 1731.1)**	**(-21.1 to 7.1)**	**(10.0 to 15.3)**	**(-22.5 to 5.8)**	**(218.9 to 337.9)**	**(-24.1 to 5.0)**	**(48.4 to 65.1)**	**(-21.4 to 7.0)**	**(1094.0 to 1471.9)**	**(-22.8 to 6.7)**	**(4.1 to 16.7)**	**(-9.1 to 22.5)**	**(98.4 to 379.7)**	**(-7.5 to 25.0)**
**East Asia**	**69.8**	**-8.8**	**1582.4**	**-9.2**	**14.0**	**-9.9**	**304.7**	**-10.8**	**60.7**	**-9.8**	**1366.2**	**-10.4**	**9.6**	**-0.3**	**221.0**	**2.0**
	**(58.0 to 83.0)**	**(-23.6 to 8.8)**	**(1307.0 to 1884.9)**	**(-24.9 to 9.5)**	**(11.1 to 17.4)**	**(-24.3 to 7.4)**	**(240.9 to 380.2)**	**(-26.1 to 7.4)**	**(51.0 to 71.6)**	**(-24.8 to 8.4)**	**(1143.3 to 1622.2)**	**(-26.4 to 9.0)**	**(4.0 to 17.2)**	**(-15.5 to 20.8)**	**(95.7 to 391.2)**	**(-13.9 to 25.1)**
China	70.1	-8.9	1584.4	-9.3	14.1	-10.2	307.2	-11.1	61.0	-9.9	1368.5	-10.5	9.6	-0.5	220.7	1.8
	(58.3 to 83.7)	(-24.2 to 9.3)	(1305.1 to 1897.9)	(-25.6 to 10.2)	(11.1 to 17.6)	(-24.9 to 7.6)	(241.0 to 385.4)	(-26.8 to 7.5)	(51.1 to 72.3)	(-25.6 to 9.1)	(1141.3 to 1632.0)	(-26.8 to 9.3)	(4.0 to 17.1)	(-16.1 to 20.8)	(95.9 to 391.0)	(-14.5 to 26.0)
North Korea	55.8	-5.7	1463.0	-5.9	12.2	-4.3	307.9	-4.7	48.6	-6.4	1279.5	-6.7	5.4	5.5	132.9	5.9
	(45.1 to 68.6)	(-16.8 to 8.1)	(1151.2 to 1820.3)	(-18.8 to 9.7)	(8.6 to 16.8)	(-18.0 to 11.6)	(209.0 to 432.5)	(-19.3 to 13.9)	(39.4 to 58.5)	(-17.4 to 7.3)	(1007.0 to 1577.7)	(-19.5 to 8.8)	(1.7 to 10.9)	(-7.9 to 21.6)	(41.0 to 270.2)	(-9.8 to 23.8)
Taiwan (Province of China)	66.2	-5.5	1600.9	-4.9	7.9	1.9	174.0	2.2	55.1	-8.2	1346.4	-7.3	13.4	5.5	308.8	6.2
	(51.5 to 86.9)	(-25.4 to 20.8)	(1222.2 to 2131.7)	(-25.7 to 23.6)	(5.6 to 10.8)	(-21.5 to 32.2)	(123.9 to 237.9)	(-22.4 to 33.3)	(43.0 to 71.6)	(-27.5 to 17.7)	(1043.0 to 1760.2)	(-27.5 to 20.5)	(6.0 to 23.9)	(-16.6 to 33.9)	(142.7 to 537.5)	(-17.9 to 37.2)
**Oceania**	**49.4**	**1.4**	**1267.8**	**0.5**	**8.7**	**1.3**	**206.3**	**1.1**	**37.1**	**-1.7**	**950.5**	**-2.5**	**12.8**	**11.5**	**325.7**	**10.4**
	**(38.1 to 63.0)**	**(-9.4 to 13.7)**	**(956.4 to 1639.2)**	**(-11.6 to 14.4)**	**(6.0 to 12.6)**	**(-13.0 to 18.9)**	**(141.2 to 298.1)**	**(-14.0 to 19.9)**	**(29.6 to 46.5)**	**(-11.8 to 10.0)**	**(740.2 to 1200.9)**	**(-14.1 to 10.6)**	**(6.0 to 22.5)**	**(-1.3 to 26.7)**	**(150.9 to 565.8)**	**(-4.0 to 26.9)**
American Samoa	65.8	2.3	1573.0	3.0	5.9	-17.2	131.0	-16.8	44.5	-0.5	1046.1	0.2	28.3	9.5	686.3	9.8
	(53.6 to 80.5)	(-8.9 to 14.6)	(1262.3 to 1938.4)	(-9.8 to 17.1)	(4.0 to 8.4)	(-32.4 to -0.7)	(87.8 to 189.6)	(-33.0 to 0.4)	(38.1 to 51.5)	(-11.5 to 10.5)	(876.0 to 1227.8)	(-12.1 to 13.0)	(16.3 to 44.1)	(-3.4 to 24.7)	(407.5 to 1052.7)	(-4.5 to 26.7)
Cook Islands	55.9	0.3	1304.5	1.1	5.0	-0.2	114.9	0.8	40.1	-3.3	930.2	-2.4	21.6	9.0	504.6	9.8
	(45.4 to 69.9)	(-12.0 to 14.2)	(1042.3 to 1657.0)	(-13.4 to 16.8)	(3.0 to 8.0)	(-17.9 to 22.3)	(70.1 to 178.5)	(-18.1 to 23.4)	(34.0 to 47.8)	(-15.2 to 10.1)	(761.7 to 1124.6)	(-16.0 to 12.3)	(11.7 to 34.2)	(-5.5 to 26.2)	(279.2 to 805.2)	(-7.8 to 28.2)
Federated States of Micronesia	77.6	3.5	1983.9	2.4	10.0	-2.7	239.0	-3.2	59.4	2.9	1503.1	1.8	22.8	8.4	590.8	7.2
	(54.2 to 103.5)	(-14.4 to 23.0)	(1318.1 to 2723.2)	(-17.6 to 24.6)	(5.7 to 16.1)	(-24.9 to 23.2)	(126.6 to 398.6)	(-28.3 to 24.3)	(42.3 to 78.0)	(-15.1 to 22.0)	(1014.1 to 2053.7)	(-20.0 to 24.2)	(11.5 to 40.0)	(-9.5 to 28.6)	(311.2 to 1025.3)	(-12.6 to 29.9)
Fiji	51.5	3.2	1279.7	1.9	3.1	-5.9	72.3	-5.9	35.5	0.3	893.2	-1.0	20.0	10.1	472.2	9.5
	(38.9 to 67.6)	(-16.9 to 27.7)	(951.4 to 1695.6)	(-20.0 to 29.0)	(2.0 to 4.5)	(-28.6 to 23.5)	(48.3 to 104.6)	(-29.5 to 25.5)	(26.5 to 44.8)	(-18.7 to 24.2)	(644.0 to 1160.6)	(-22.5 to 26.0)	(10.9 to 32.1)	(-12.1 to 36.2)	(265.9 to 747.2)	(-14.3 to 38.0)
Guam	50.1	8.7	1252.0	9.4	5.3	-1.0	128.2	2.0	37.8	6.0	941.9	6.9	15.2	21.3	379.8	21.1
	(40.5 to 60.9)	(-9.3 to 29.5)	(1006.2 to 1528.6)	(-9.5 to 31.6)	(3.3 to 8.3)	(-25.4 to 28.5)	(82.3 to 200.1)	(-22.7 to 32.0)	(31.3 to 45.3)	(-11.7 to 26.9)	(767.0 to 1133.6)	(-12.1 to 28.9)	(8.2 to 24.4)	(0.6 to 44.3)	(211.5 to 594.1)	(-0.2 to 45.0)
Kiribati	103.5	-1.4	2794.0	-2.9	6.3	-8.5	159.3	-9.1	91.5	-1.9	2456.6	-3.3	17.8	3.8	475.9	2.1
	(81.4 to 125.7)	(-13.9 to 11.2)	(2141.0 to 3503.5)	(-16.7 to 11.5)	(4.2 to 8.8)	(-25.3 to 9.9)	(104.2 to 230.1)	(-26.7 to 10.2)	(72.1 to 110.5)	(-14.2 to 10.9)	(1881.8 to 3064.1)	(-17.2 to 11.4)	(8.6 to 29.8)	(-9.9 to 18.2)	(244.6 to 787.7)	(-12.9 to 17.5)
Marshall Islands	68.9	0.5	1775.7	-0.9	9.3	-7.8	213.6	-9.5	50.9	-1.4	1309.6	-3.2	21.9	10.8	554.2	10.3
	(48.4 to 93.4)	(-14.9 to 18.4)	(1217.6 to 2436.7)	(-17.4 to 18.1)	(5.1 to 15.2)	(-28.4 to 17.0)	(115.6 to 352.3)	(-30.3 to 15.8)	(37.1 to 66.6)	(-16.9 to 16.4)	(928.1 to 1768.7)	(-19.3 to 15.8)	(9.4 to 40.9)	(-5.7 to 31.8)	(245.8 to 1021.9)	(-7.7 to 33.5)
Nauru	83.0	-3.1	2114.8	-4.7	7.2	-12.6	171.6	-13.3	65.5	-7.3	1658.8	-8.9	23.4	18.5	596.1	16.7
	(60.5 to 104.7)	(-17.6 to 14.4)	(1508.0 to 2718.3)	(-19.8 to 14.4)	(4.0 to 11.9)	(-34.5 to 13.6)	(91.5 to 291.4)	(-35.7 to 14.1)	(49.3 to 81.6)	(-21.4 to 9.0)	(1204.1 to 2112.2)	(-23.0 to 9.2)	(11.4 to 39.8)	(-2.8 to 45.2)	(297.0 to 997.2)	(-5.0 to 43.5)
Niue	61.7	-1.2	1484.3	-1.5	5.2	-2.2	120.6	-2.0	44.4	-3.9	1063.2	-4.0	23.3	6.3	555.1	5.7
	(48.0 to 77.7)	(-12.4 to 11.1)	(1126.7 to 1910.3)	(-14.5 to 13.1)	(3.1 to 8.3)	(-19.0 to 17.6)	(72.9 to 195.4)	(-19.1 to 18.3)	(36.3 to 53.1)	(-14.3 to 7.7)	(830.8 to 1314.1)	(-16.7 to 10.1)	(11.9 to 39.2)	(-5.7 to 19.8)	(288.7 to 928.3)	(-8.5 to 21.0)
Northern Mariana Islands	81.8	-1.8	1918.7	-2.0	8.4	-7.9	188.4	-7.6	62.6	-3.9	1457.0	-4.0	25.1	5.0	587.4	4.6
	(69.2 to 95.6)	(-12.4 to 9.6)	(1598.4 to 2268.7)	(-14.4 to 11.9)	(5.8 to 12.5)	(-23.3 to 10.4)	(128.4 to 283.6)	(-24.3 to 10.6)	(53.7 to 70.9)	(-14.2 to 7.2)	(1216.3 to 1692.6)	(-16.1 to 9.4)	(13.9 to 39.1)	(-6.3 to 18.3)	(340.8 to 893.7)	(-8.6 to 20.6)
Palau	82.5	-0.8	2060.4	-1.6	6.3	8.2	154.0	8.1	63.4	-3.8	1600.4	-4.5	24.3	8.0	575.0	7.6
	(64.5 to 104.6)	(-15.8 to 17.8)	(1592.8 to 2634.1)	(-18.2 to 18.5)	(3.6 to 11.0)	(-15.0 to 42.3)	(89.5 to 264.4)	(-15.7 to 40.5)	(50.4 to 78.6)	(-18.6 to 14.6)	(1248.6 to 2018.0)	(-20.8 to 15.7)	(12.2 to 40.5)	(-8.5 to 28.8)	(298.2 to 947.5)	(-10.1 to 30.0)
Papua New Guinea	44.1	0.6	1139.4	-0.2	9.6	1.0	224.1	0.1	33.4	-2.5	859.4	-3.2	10.0	11.3	261.2	10.7
	(32.2 to 59.5)	(-12.3 to 15.9)	(804.7 to 1544.0)	(-14.2 to 17.0)	(6.4 to 14.5)	(-16.2 to 23.3)	(148.3 to 339.7)	(-17.7 to 23.0)	(24.9 to 44.1)	(-15.1 to 12.3)	(628.9 to 1144.6)	(-16.8 to 13.3)	(4.0 to 19.2)	(-5.5 to 31.5)	(105.9 to 494.7)	(-8.1 to 33.3)

Location	All risk factors*				Environmental and occupational risks*				Behavioural risks*				Metabolic risks*			
	Death ASR	Death % change	DALY ASR	DALY % change	Death ASR	Death % change	DALY ASR	DALY % change	Death ASR	Death % change	DALY ASR	DALY % change	Death ASR	Death % change	DALY ASR	DALY % change
Samoa	44.1	-1.0	1135.4	-1.6	3.9	-6.9	93.8	-7.4	33.3	-3.0	854.1	-3.6	13.8	6.2	354.4	5.1
	(35.6 to 55.5)	(-13.7 to 14.9)	(879.1 to 1458.5)	(-16.6 to 16.8)	(2.7 to 5.3)	(-21.2 to 11.3)	(63.9 to 128.2)	(-23.4 to 12.3)	(27.5 to 40.6)	(-15.2 to 12.3)	(676.7 to 1077.4)	(-17.8 to 13.9)	(7.3 to 22.8)	(-10.1 to 24.7)	(193.6 to 575.7)	(-12.4 to 25.9)
Solomon Islands	80.7	11.3	2259.2	11.4	15.2	5.9	384.5	6.1	62.8	6.5	1725.7	5.9	19.5	37.5	557.2	40.1
	(57.5 to 108.9)	(-4.4 to 29.9)	(1520.7 to 3126.7)	(-6.2 to 33.2)	(8.3 to 24.5)	(-12.8 to 30.2)	(198.4 to 631.6)	(-14.5 to 32.8)	(45.3 to 84.2)	(-8.3 to 23.7)	(1159.5 to 2381.1)	(-10.4 to 26.4)	(8.9 to 34.8)	(14.9 to 63.8)	(264.5 to 974.1)	(15.4 to 70.0)
Tokelau	54.2	0.0	1351.7	-1.0	4.7	2.6	107.5	1.9	40.9	-2.8	1013.9	-4.0	16.7	11.1	415.1	10.3
	(42.0 to 70.2)	(-13.0 to 15.2)	(1013.8 to 1792.6)	(-15.1 to 16.5)	(2.5 to 8.7)	(-17.0 to 28.6)	(55.9 to 198.9)	(-18.6 to 28.9)	(32.8 to 50.9)	(-15.5 to 12.1)	(777.8 to 1307.7)	(-18.1 to 12.8)	(8.5 to 28.6)	(-4.2 to 30.0)	(217.6 to 706.7)	(-6.5 to 31.1)
Tonga	71.6	-1.2	1739.5	-1.8	7.5	-10.9	169.0	-11.1	55.7	-3.3	1311.8	-3.8	21.4	6.8	555.5	5.1
	(56.7 to 89.2)	(-14.7 to 14.6)	(1354.6 to 2215.1)	(-16.9 to 16.2)	(4.9 to 11.1)	(-29.0 to 9.2)	(109.9 to 254.6)	(-29.9 to 10.1)	(45.5 to 67.7)	(-16.3 to 11.8)	(1034.8 to 1631.5)	(-18.3 to 13.4)	(11.3 to 34.4)	(-9.1 to 26.3)	(302.2 to 884.3)	(-12.7 to 26.4)
Tuvalu	60.0	-0.4	1509.5	-1.6	5.4	-7.0	127.4	-7.3	46.8	-2.4	1168.5	-3.5	16.9	9.9	424.1	8.5
	(44.8 to 78.2)	(-15.8 to 17.7)	(1107.0 to 2013.1)	(-17.7 to 17.9)	(3.3 to 8.6)	(-28.7 to 16.6)	(78.2 to 205.1)	(-29.7 to 18.0)	(36.2 to 60.0)	(-17.4 to 15.1)	(882.2 to 1534.7)	(-19.4 to 15.9)	(7.8 to 30.0)	(-7.4 to 31.9)	(205.4 to 742.7)	(-9.9 to 32.0)
Vanuatu	54.1	2.7	1364.3	2.8	11.8	2.9	274.0	3.3	39.7	-0.9	992.8	-0.6	13.5	15.9	341.0	15.5
	(38.6 to 71.2)	(-11.3 to 20.1)	(949.7 to 1833.0)	(-13.3 to 23.3)	(7.3 to 18.4)	(-13.8 to 24.9)	(169.7 to 427.5)	(-13.7 to 26.0)	(28.6 to 52.3)	(-14.5 to 16.5)	(684.3 to 1345.8)	(-16.2 to 19.1)	(5.8 to 24.6)	(-2.1 to 37.5)	(157.1 to 604.3)	(-3.7 to 39.6)
**Southeast Asia**	**48.0**	**1.1**	**1142.1**	**-0.1**	**7.1**	**-8.8**	**167.5**	**-9.6**	**39.8**	**-1.5**	**939.6**	**-2.7**	**9.0**	**25.1**	**208.4**	**25.5**
	**(41.0 to 56.0)**	**(-9.1 to 11.7)**	**(977.7 to 1335.1)**	**(-10.8 to 11.3)**	**(5.5 to 8.8)**	**(-20.8 to 3.4)**	**(131.4 to 208.2)**	**(-21.7 to 2.8)**	**(34.8 to 45.3)**	**(-11.5 to 8.9)**	**(820.4 to 1079.2)**	**(-13.1 to 8.0)**	**(4.3 to 15.5)**	**(10.9 to 42.5)**	**(105.3 to 346.5)**	**(10.2 to 43.6)**
Cambodia	57.1	5.3	1350.9	4.0	10.8	1.0	251.9	0.2	49.6	2.2	1165.0	1.0	8.3	47.8	184.7	45.6
	(45.7 to 66.7)	(-10.1 to 18.9)	(1062.7 to 1623.3)	(-12.7 to 19.8)	(8.0 to 14.0)	(-16.6 to 21.6)	(184.0 to 326.6)	(-19.1 to 21.3)	(39.6 to 57.1)	(-12.7 to 14.9)	(913.6 to 1382.2)	(-15.2 to 16.6)	(3.3 to 14.9)	(25.9 to 76.7)	(75.4 to 327.7)	(21.3 to 79.2)
Indonesia	44.5	5.7	1037.3	3.3	6.9	-5.2	161.0	-7.1	36.8	3.8	843.9	0.9	7.9	28.1	189.6	31.9
	(35.0 to 54.6)	(-11.5 to 21.4)	(820.9 to 1283.6)	(-14.9 to 21.7)	(4.7 to 9.2)	(-23.8 to 13.5)	(110.0 to 214.1)	(-26.9 to 14.2)	(29.8 to 44.7)	(-13.5 to 20.8)	(684.2 to 1030.8)	(-16.7 to 19.8)	(3.5 to 14.3)	(6.5 to 53.4)	(90.1 to 334.2)	(6.5 to 60.5)
Lao s	51.7	3.5	1255.0	0.9	9.8	-0.4	233.0	-2.3	43.0	1.2	1034.0	-1.5	9.6	27.2	224.1	25.1
	(39.8 to 64.2)	(-10.9 to 22.3)	(940.2 to 1593.1)	(-14.9 to 21.4)	(6.7 to 13.2)	(-19.4 to 21.6)	(158.8 to 322.2)	(-22.4 to 20.8)	(33.0 to 53.0)	(-12.9 to 19.4)	(773.6 to 1299.3)	(-16.8 to 18.9)	(4.2 to 17.6)	(6.9 to 56.0)	(98.5 to 404.7)	(3.0 to 55.6)
Malaysia	51.4	2.9	1166.3	4.6	4.5	-11.4	104.2	-9.4	40.3	-0.3	905.1	1.5	14.2	18.4	314.7	19.1
	(40.0 to 65.1)	(-16.9 to 26.3)	(896.8 to 1510.0)	(-16.8 to 30.7)	(3.2 to 6.1)	(-32.7 to 14.8)	(73.4 to 142.1)	(-31.2 to 18.0)	(32.2 to 49.9)	(-20.1 to 21.7)	(715.7 to 1131.0)	(-19.8 to 27.0)	(7.3 to 24.5)	(-4.3 to 46.5)	(163.7 to 530.9)	(-5.4 to 50.1)
Maldives	29.7	-3.2	602.4	-3.7	3.1	-16.9	58.3	-15.0	24.9	-6.2	495.2	-6.9	6.1	21.9	128.3	20.4
	(24.1 to 35.7)	(-18.5 to 13.1)	(489.3 to 723.7)	(-19.8 to 14.1)	(2.2 to 4.4)	(-35.0 to 4.7)	(41.9 to 81.6)	(-32.8 to 5.1)	(20.4 to 29.2)	(-21.3 to 9.8)	(405.4 to 584.5)	(-23.1 to 10.3)	(2.7 to 10.4)	(2.4 to 47.4)	(61.7 to 212.7)	(-0.7 to 47.5)
Mauritius	36.4	0.5	852.0	-0.8	2.4	-12.9	58.7	-10.7	26.1	-3.8	607.2	-5.0	13.2	13.2	300.8	11.2
	(28.2 to 46.7)	(-17.6 to 21.7)	(653.1 to 1105.0)	(-19.9 to 21.2)	(1.5 to 3.5)	(-31.9 to 8.9)	(38.0 to 83.6)	(-30.5 to 12.3)	(21.3 to 31.8)	(-21.2 to 17.0)	(485.7 to 752.8)	(-23.4 to 17.0)	(6.8 to 22.2)	(-7.0 to 37.2)	(156.1 to 499.4)	(-9.7 to 36.2)
Myanmar	46.7	-4.9	1105.8	-7.6	9.2	-6.4	217.6	-8.2	38.0	-8.9	886.0	-11.6	8.9	19.3	202.1	15.5
	(36.6 to 59.5)	(-17.3 to 10.4)	(849.5 to 1425.5)	(-21.2 to 10.6)	(6.4 to 13.1)	(-23.6 to 15.6)	(148.5 to 314.4)	(-25.9 to 14.6)	(30.4 to 47.8)	(-20.8 to 5.7)	(688.4 to 1133.0)	(-24.9 to 6.1)	(3.7 to 16.1)	(3.3 to 42.1)	(89.4 to 357.6)	(-2.2 to 42.3)
Philippines	43.9	2.6	1105.4	2.5	5.7	-10.5	141.8	-10.5	36.7	-0.7	919.3	-0.8	8.4	26.5	204.5	28.5
	(35.9 to 53.6)	(-15.3 to 22.3)	(903.8 to 1351.4)	(-15.9 to 23.3)	(4.3 to 7.5)	(-29.3 to 10.2)	(105.5 to 185.7)	(-29.7 to 10.7)	(30.1 to 44.1)	(-18.1 to 18.9)	(751.8 to 1108.0)	(-18.7 to 19.9)	(4.0 to 14.3)	(4.2 to 55.2)	(100.8 to 339.2)	(4.7 to 59.0)
Seychelles	74.1	3.5	1765.0	2.9	4.2	3.7	100.8	3.9	60.2	0.5	1433.7	0.0	19.3	19.9	441.7	18.9
	(63.5 to 86.5)	(-6.6 to 15.2)	(1510.0 to 2044.4)	(-8.0 to 15.1)	(2.9 to 5.6)	(-10.6 to 21.4)	(73.2 to 134.7)	(-10.7 to 23.8)	(53.6 to 68.4)	(-9.3 to 11.8)	(1268.6 to 1635.9)	(-10.5 to 12.8)	(9.5 to 31.7)	(6.5 to 34.8)	(235.1 to 705.2)	(5.0 to 34.1)
Sri Lanka	28.2	-8.9	646.3	-9.1	3.3	-15.4	79.9	-15.1	20.7	-14.9	474.0	-14.7	8.2	13.9	177.0	13.2
	(20.5 to 37.9)	(-31.7 to 18.3)	(463.6 to 881.6)	(-33.0 to 20.0)	(2.2 to 4.8)	(-39.9 to 16.3)	(52.9 to 116.2)	(-40.4 to 19.1)	(15.4 to 27.2)	(-35.4 to 10.6)	(347.9 to 628.8)	(-36.9 to 12.5)	(4.0 to 14.5)	(-13.3 to 50.0)	(86.5 to 311.6)	(-15.9 to 50.6)
Thailand	53.6	-8.2	1278.2	-8.3	6.8	-20.0	157.3	-20.2	44.3	-10.7	1043.6	-10.9	10.9	15.1	263.5	15.6
	(39.6 to 70.9)	(-31.6 to 22.2)	(932.9 to 1700.8)	(-32.7 to 24.0)	(4.7 to 9.5)	(-42.0 to 10.0)	(107.0 to 219.1)	(-43.1 to 11.1)	(32.7 to 58.1)	(-33.6 to 18.5)	(762.9 to 1386.6)	(-35.1 to 20.5)	(5.2 to 19.4)	(-15.0 to 54.7)	(128.8 to 457.0)	(-15.8 to 57.8)
Timor-Leste	41.2	13.4	972.0	16.5	7.9	7.1	183.0	9.9	34.9	11.0	822.0	14.3	5.9	45.6	127.0	50.5
	(30.0 to 52.2)	(-6.7 to 34.6)	(684.3 to 1241.2)	(-5.7 to 42.3)	(5.3 to 10.9)	(-16.5 to 32.7)	(118.8 to 255.8)	(-14.9 to 39.4)	(25.5 to 43.2)	(-8.5 to 31.3)	(567.0 to 1038.5)	(-7.4 to 39.6)	(2.1 to 11.5)	(19.8 to 80.6)	(45.1 to 242.8)	(22.3 to 92.0)
Vietnam	56.5	3.4	1402.1	2.4	8.8	-6.4	222.6	-7.4	48.5	1.3	1213.5	0.8	8.3	33.8	178.5	33.3
	(44.8 to 68.6)	(-13.0 to 20.0)	(1102.3 to 1733.7)	(-15.7 to 21.6)	(6.2 to 11.9)	(-26.7 to 14.2)	(155.9 to 305.4)	(-28.2 to 15.5)	(39.0 to 58.5)	(-14.8 to 17.2)	(956.1 to 1496.5)	(-16.8 to 19.6)	(3.2 to 15.3)	(12.5 to 61.5)	(69.8 to 331.8)	(9.6 to 64.4)
**Sub-Saharan Africa**	**37.4**	**-3.1**	**937.1**	**-4.2**	**4.7**	**-5.1**	**107.5**	**-6.5**	**29.7**	**-5.9**	**765.3**	**-6.7**	**7.0**	**12.2**	**155.1**	**12.1**
	**(32.2 to 43.3)**	**(-10.5 to 5.3)**	**(795.3 to 1091.2)**	**(-12.8 to 5.6)**	**(3.8 to 5.9)**	**(-14.8 to 5.6)**	**(85.1 to 135.7)**	**(-16.9 to 5.3)**	**(25.7 to 33.8)**	**(-13.1 to 2.5)**	**(644.7 to 886.4)**	**(-15.1 to 3.4)**	**(3.7 to 11.2)**	**(4.2 to 22.4)**	**(84.4 to 243.7)**	**(2.8 to 23.5)**
**Central sub-Saharan Africa**	**38.3**	**0.8**	**993.4**	**-1.4**	**5.7**	**0.1**	**140.1**	**-1.1**	**30.6**	**-2.1**	**812.6**	**-4.0**	**6.1**	**26.6**	**138.6**	**26.3**
	**(28.7 to 48.4)**	**(-13.4 to 18.1)**	**(743.5 to 1256.3)**	**(-17.3 to 17.8)**	**(3.4 to 10.0)**	**(-17.8 to 23.0)**	**(83.4 to 252.2)**	**(-19.5 to 23.3)**	**(23.1 to 38.3)**	**(-15.8 to 15.2)**	**(607.8 to 1033.0)**	**(-19.2 to 14.8)**	**(2.6 to 11.2)**	**(9.5 to 55.1)**	**(61.4 to 253.5)**	**(7.5 to 57.9)**
Angola	43.3	-0.6	1106.6	-2.8	5.5	-8.2	133.9	-9.7	36.5	-3.0	946.0	-5.0	6.4	29.1	146.6	29.9
	(34.6 to 54.9)	(-15.2 to 19.7)	(860.2 to 1433.1)	(-19.2 to 20.5)	(3.9 to 8.1)	(-28.0 to 16.5)	(94.3 to 195.5)	(-30.1 to 16.7)	(29.0 to 46.3)	(-17.6 to 17.2)	(725.2 to 1225.7)	(-21.5 to 19.0)	(2.8 to 11.4)	(9.6 to 66.1)	(66.1 to 255.5)	(8.0 to 70.9)
Central African Republic	43.1	-8.7	1200.3	-9.3	6.3	-10.0	165.7	-10.4	35.9	-9.8	1019.6	-10.2	4.9	-0.9	119.4	-1.4
	(30.9 to 58.3)	(-24.4 to 8.6)	(829.6 to 1651.0)	(-26.5 to 10.5)	(3.3 to 11.5)	(-26.7 to 10.3)	(81.4 to 303.9)	(-28.1 to 11.7)	(25.6 to 47.8)	(-25.6 to 8.3)	(703.2 to 1398.0)	(-27.3 to 10.4)	(1.8 to 10.2)	(-17.5 to 18.8)	(43.1 to 247.1)	(-19.5 to 20.4)
Congo (Brazzaville)	50.3	0.7	1271.5	-2.6	6.7	-0.3	160.2	-2.5	39.1	-0.7	1008.3	-4.1	10.7	14.7	245.0	12.5
	(39.9 to 62.8)	(-15.5 to 19.9)	(972.5 to 1643.8)	(-20.4 to 20.2)	(4.4 to 10.3)	(-20.3 to 22.5)	(103.5 to 252.2)	(-24.1 to 22.7)	(30.9 to 49.0)	(-16.7 to 18.8)	(763.5 to 1323.0)	(-21.8 to 18.6)	(5.4 to 17.7)	(-3.6 to 37.3)	(128.5 to 403.5)	(-8.4 to 37.3)
DR Congo	34.9	1.5	912.2	-0.3	5.6	3.6	138.2	2.8	27.5	-2.0	737.1	-3.5	5.4	30.4	121.5	30.5
	(24.3 to 46.2)	(-15.2 to 22.3)	(639.3 to 1204.1)	(-18.8 to 22.5)	(2.8 to 11.2)	(-18.1 to 34.1)	(69.8 to 280.1)	(-19.8 to 35.0)	(19.3 to 35.8)	(-18.6 to 17.9)	(515.3 to 965.6)	(-21.1 to 18.8)	(2.2 to 10.5)	(9.3 to 67.9)	(50.1 to 233.8)	(7.7 to 72.2)
Equatorial Guinea	48.4	8.8	1151.0	4.8	6.4	5.7	145.3	3.9	35.3	5.2	863.5	1.5	13.0	28.1	280.8	25.5
	(34.8 to 68.4)	(-12.8 to 37.7)	(774.9 to 1727.0)	(-19.7 to 40.2)	(3.8 to 10.1)	(-19.9 to 37.3)	(84.0 to 237.0)	(-23.4 to 40.3)	(25.7 to 49.2)	(-15.9 to 33.5)	(590.5 to 1283.0)	(-22.9 to 36.5)	(6.6 to 22.7)	(3.4 to 62.5)	(140.5 to 496.8)	(-1.9 to 63.6)
Gabon	53.6	0.0	1309.5	-3.0	6.8	-1.7	160.5	-4.6	39.3	-2.6	986.6	-5.4	14.7	12.7	330.7	10.8
	(41.1 to 66.6)	(-16.4 to 17.8)	(973.8 to 1670.1)	(-21.0 to 18.4)	(4.1 to 10.5)	(-20.3 to 23.2)	(96.1 to 251.6)	(-24.4 to 22.0)	(30.4 to 47.5)	(-18.1 to 15.4)	(732.9 to 1235.0)	(-22.8 to 16.2)	(7.8 to 24.5)	(-4.1 to 33.4)	(175.9 to 557.0)	(-9.4 to 35.1)
**Eastern sub-Saharan**	**36.5**	**0.2**	**946.6**	**-1.1**	**3.7**	**1.5**	**85.1**	**0.6**	**30.4**	**-2.7**	**803.5**	**-3.8**	**5.6**	**24.0**	**129.2**	**24.3**
**Africa**	**(30.3 to 44.4)**	**(-7.8 to 8.7)**	**(770.0 to 1167.2)**	**(-10.9 to 8.6)**	**(2.7 to 5.5)**	**(-10.4 to 12.5)**	**(63.0 to 123.0)**	**(-11.6 to 12.3)**	**(25.3 to 36.8)**	**(-11.0 to 5.5)**	**(653.8 to 989.3)**	**(-13.2 to 5.9)**	**(2.7 to 9.2)**	**(12.6 to 44.8)**	**(65.2 to 211.6)**	**(11.2 to 46.3)**
Burundi	36.3	-6.9	988.5	-7.5	3.8	-2.6	90.6	-3.0	31.4	-8.3	871.0	-8.8	3.7	6.4	85.6	7.3
	(25.9 to 50.4)	(-22.1 to 10.4)	(684.5 to 1389.6)	(-24.5 to 12.7)	(2.4 to 6.1)	(-20.9 to 19.5)	(56.1 to 144.5)	(-22.2 to 19.3)	(22.5 to 43.3)	(-23.3 to 9.4)	(612.6 to 1231.0)	(-25.4 to 11.5)	(1.4 to 7.3)	(-9.6 to 28.8)	(33.0 to 166.9)	(-10.2 to 32.6)
Comoros	36.7	0.9	946.5	2.9	3.5	4.2	82.0	6.6	30.4	-1.5	796.3	0.7	5.9	13.6	138.5	16.4
	(28.0 to 47.9)	(-15.5 to 19.3)	(683.6 to 1282.6)	(-18.0 to 26.0)	(2.4 to 5.3)	(-15.7 to 26.9)	(54.2 to 121.1)	(-15.9 to 31.1)	(23.1 to 39.8)	(-17.4 to 17.3)	(566.4 to 1094.4)	(-20.3 to 24.2)	(2.8 to 10.1)	(-4.2 to 38.0)	(66.3 to 235.7)	(-5.7 to 44.3)
Djibouti	44.4	2.2	1103.2	0.2	4.4	7.7	99.9	5.9	38.4	0.2	963.6	-1.8	6.5	24.1	148.9	24.6
	(32.3 to 64.6)	(-15.6 to 24.5)	(747.8 to 1675.3)	(-19.9 to 25.5)	(2.5 to 8.1)	(-13.7 to 33.8)	(56.0 to 184.0)	(-17.3 to 33.7)	(27.6 to 55.5)	(-17.2 to 22.2)	(646.8 to 1479.1)	(-21.8 to 23.9)	(2.9 to 11.6)	(3.1 to 57.5)	(67.9 to 271.6)	(0.2 to 62.1)
Eritrea	42.5	-2.3	1161.2	-4.0	4.2	1.3	104.6	-0.4	34.8	-5.3	983.0	-6.5	6.2	19.1	140.3	19.0
	(30.7 to 56.4)	(-17.7 to 16.1)	(828.3 to 1567.6)	(-20.5 to 15.5)	(3.0 to 5.9)	(-17.8 to 24.6)	(73.7 to 146.1)	(-20.3 to 25.2)	(25.2 to 46.5)	(-20.6 to 11.9)	(694.4 to 1331.2)	(-23.1 to 12.0)	(2.7 to 10.8)	(-0.4 to 46.9)	(61.9 to 243.4)	(-1.2 to 47.2)
Ethiopia	23.5	5.0	595.4	1.8	3.1	1.5	68.7	0.4	18.8	2.3	491.2	-0.9	3.0	35.6	67.6	36.5
	(17.9 to 32.0)	(-6.8 to 17.7)	(450.7 to 817.3)	(-12.2 to 16.6)	(1.9 to 5.1)	(-19.0 to 27.0)	(42.5 to 110.5)	(-18.9 to 25.6)	(14.7 to 25.8)	(-10.2 to 15.0)	(379.3 to 688.4)	(-16.0 to 14.5)	(1.3 to 5.6)	(16.7 to 69.2)	(29.6 to 120.9)	(16.6 to 70.4)
Kenya	33.0	-5.4	831.8	-7.3	3.0	-3.0	68.8	-5.3	26.7	-9.6	683.4	-10.9	6.6	18.6	158.4	16.5
	(26.7 to 42.5)	(-16.7 to 7.9)	(664.6 to 1091.8)	(-19.7 to 8.0)	(2.2 to 4.3)	(-16.6 to 11.7)	(51.1 to 97.0)	(-19.7 to 10.6)	(21.8 to 34.5)	(-20.8 to 3.4)	(551.2 to 896.7)	(-23.4 to 3.8)	(3.4 to 11.1)	(3.3 to 41.9)	(81.7 to 263.5)	(-0.1 to 40.8)
Madagascar	31.6	-3.3	871.8	-4.1	3.2	-1.2	76.1	-1.4	26.4	-6.5	747.7	-7.0	4.3	23.4	101.4	25.4
	(22.9 to 42.1)	(-20.3 to 16.8)	(610.1 to 1175.9)	(-22.6 to 18.0)	(2.1 to 4.8)	(-20.8 to 23.1)	(50.6 to 116.0)	(-22.6 to 26.3)	(19.0 to 35.0)	(-22.7 to 13.5)	(524.8 to 1010.1)	(-24.8 to 15.3)	(1.9 to 7.6)	(-0.5 to 58.4)	(46.7 to 182.3)	(-0.8 to 63.8)
Malawi	45.7	-4.0	1193.1	-5.4	3.1	-3.3	70.9	-4.5	38.5	-6.1	1026.9	-7.3	8.3	10.8	194.1	10.7
	(35.3 to 57.8)	(-20.5 to 13.8)	(898.8 to 1570.6)	(-24.6 to 16.4)	(2.2 to 4.3)	(-21.3 to 15.1)	(50.8 to 100.6)	(-24.5 to 16.3)	(29.5 to 48.8)	(-23.0 to 12.5)	(753.2 to 1350.2)	(-26.8 to 14.0)	(3.7 to 14.5)	(-7.5 to 35.0)	(86.4 to 347.3)	(-9.7 to 38.6)
Mozambique	41.3	7.3	1078.0	6.6	4.5	4.6	101.6	4.4	34.8	5.3	929.8	4.8	5.4	29.2	120.4	30.3
	(31.5 to 53.2)	(-14.7 to 34.2)	(789.6 to 1430.1)	(-18.6 to 38.4)	(3.1 to 6.2)	(-16.2 to 31.3)	(69.2 to 143.3)	(-17.8 to 33.0)	(26.2 to 44.7)	(-17.3 to 31.8)	(659.9 to 1237.9)	(-20.7 to 36.7)	(2.3 to 9.5)	(4.9 to 67.0)	(53.2 to 208.9)	(4.1 to 70.3)
Rwanda	44.7	3.5	1117.7	1.4	4.4	8.9	98.7	8.2	38.8	1.1	979.2	-1.0	5.9	31.8	132.3	32.1
	(35.2 to 56.8)	(-10.3 to 21.2)	(846.1 to 1481.1)	(-15.4 to 22.9)	(2.9 to 6.9)	(-9.8 to 31.1)	(64.2 to 155.4)	(-12.6 to 32.3)	(30.5 to 49.5)	(-12.6 to 19.0)	(736.4 to 1298.4)	(-17.7 to 19.5)	(2.6 to 10.0)	(12.3 to 66.9)	(59.3 to 225.0)	(10.8 to 70.0)
Somalia	37.3	-5.2	1042.1	-6.2	3.3	-4.3	83.0	-4.8	33.5	-5.8	946.6	-6.8	2.7	1.9	64.1	1.1
	(24.8 to 54.1)	(-20.4 to 13.7)	(683.2 to 1519.6)	(-22.8 to 14.5)	(1.6 to 6.2)	(-22.6 to 18.1)	(41.1 to 151.9)	(-23.3 to 18.8)	(22.3 to 48.4)	(-20.8 to 13.4)	(623.7 to 1386.3)	(-23.1 to 14.2)	(0.8 to 6.2)	(-14.2 to 21.7)	(18.6 to 146.9)	(-16.3 to 22.9)
South Sudan	33.1	-2.2	832.9	-1.9	4.3	-4.9	97.3	-5.2	26.1	-4.5	670.5	-3.9	6.3	10.0	143.8	10.5
	(23.7 to 46.3)	(-18.3 to 18.0)	(569.1 to 1218.3)	(-19.8 to 21.2)	(2.6 to 6.4)	(-23.4 to 16.5)	(57.9 to 145.0)	(-24.6 to 17.2)	(18.6 to 36.1)	(-20.4 to 16.0)	(454.8 to 973.5)	(-21.9 to 19.8)	(3.0 to 11.0)	(-7.0 to 35.3)	(68.6 to 249.3)	(-9.6 to 39.0)
Tanzania	44.8	1.6	1144.2	1.0	4.6	6.3	106.7	5.2	37.7	-1.4	978.3	-1.7	7.0	23.8	163.4	23.7
	(35.0 to 57.5)	(-13.6 to 19.2)	(865.8 to 1516.0)	(-17.0 to 22.5)	(3.1 to 7.4)	(-13.8 to 26.3)	(70.5 to 171.0)	(-16.6 to 29.2)	(29.2 to 48.6)	(-16.3 to 15.7)	(735.0 to 1300.1)	(-19.6 to 19.1)	(3.6 to 11.3)	(3.7 to 53.3)	(85.4 to 264.8)	(1.3 to 56.6)
Uganda	48.8	2.1	1281.3	1.4	3.8	5.8	88.1	5.4	40.3	-1.9	1082.7	-2.2	8.4	31.1	198.0	30.9
	(39.2 to 59.0)	(-14.3 to 20.0)	(1005.4 to 1580.5)	(-17.2 to 23.8)	(2.8 to 5.2)	(-13.9 to 26.3)	(64.4 to 120.8)	(-14.2 to 27.9)	(32.5 to 48.2)	(-17.6 to 15.6)	(846.3 to 1335.9)	(-20.2 to 20.0)	(4.0 to 13.8)	(8.8 to 68.5)	(95.6 to 327.1)	(6.6 to 70.6)
Zambia	51.3	-4.5	1342.5	-6.4	5.1	-3.9	121.8	-3.8	43.0	-7.6	1141.5	-9.4	8.4	25.1	201.8	26.2
	(40.3 to 64.9)	(-21.1 to 15.9)	(1016.0 to 1737.4)	(-25.7 to 17.5)	(3.6 to 7.4)	(-25.3 to 20.1)	(83.0 to 176.5)	(-26.9 to 22.7)	(33.8 to 54.6)	(-24.0 to 13.1)	(859.4 to 1494.5)	(-27.9 to 13.3)	(4.2 to 14.3)	(1.7 to 63.6)	(104.4 to 339.4)	(-0.3 to 68.6)

Location	All risk factors*				Environmental and occupational risks*				Behavioural risks*				Metabolic risks*			
	Death ASR	Death % change	DALY ASR	DALY % change	Death ASR	Death % change	DALY ASR	DALY % change	Death ASR	Death % change	DALY ASR	DALY % change	Death ASR	Death % change	DALY ASR	DALY % change
**Southern sub-Saharan Africa**	**59.9 (53.8 to 66.6)**	**-14.9 (-20.7 to -7.5)**	**1457.7 (1303.4 to 1624.5)**	**-16.9 (-23.4 to -8.7)**	**8.3 (6.7 to 10.0)**	**-18.5 (-27.7 to -7.3)**	**186.1 (149.8 to 227.3)**	**-22.0 (-31.3 to -10.5)**	**46.7 (42.6 to 50.9)**	**-17.6 (-23.2 to -10.4)**	**1167.2 (1057.6 to 1281.7)**	**-19.1 (-25.7 to -10.9)**	**15.1 (8.9 to 22.4)**	**-5.8 (-12.4 to 2.5)**	**331.3 (198.5 to 489.9)**	**-7.6 (-15.2 to 1.7)**
Botswana	71.0 (53.2 to 92.8)	-3.8 (-21.6 to 17.5)	1749.1 (1234.1 to 2335.5)	-5.6 (-26.0 to 18.6)	10.7 (7.2 to 14.7)	-9.0 (-27.7 to 12.1)	243.7 (158.4 to 342.1)	-12.0 (-31.5 to 11.1)	56.1 (41.5 to 73.0)	-6.5 (-24.0 to 14.5)	1415.3 (995.5 to 1901.4)	-8.1 (-27.9 to 16.8)	16.2 (8.8 to 26.8)	14.9 (-7.2 to 42.8)	357.3 (193.4 to 593.0)	15.6 (-9.8 to 48.1)
Eswatini	73.2 (50.8 to 102.8)	-14.7 (-31.3 to 6.5)	1840.5 (1237.5 to 2674.1)	-17.0 (-34.3 to 7.4)	10.1 (6.1 to 14.9)	-17.0 (-34.8 to 7.9)	242.3 (140.9 to 369.6)	-19.7 (-38.7 to 7.8)	51.4 (36.1 to 71.4)	-17.1 (-32.9 to 4.6)	1319.9 (892.6 to 1911.4)	-19.4 (-36.3 to 5.1)	22.4 (12.3 to 36.2)	-8.2 (-24.7 to 14.1)	531.2 (289.5 to 875.2)	-9.5 (-27.3 to 15.1)
Lesotho	82.6 (59.4 to 111.5)	2.6 (-17.8 to 26.4)	2146.1 (1500.3 to 2997.7)	1.8 (-20.0 to 28.3)	12.2 (8.1 to 17.7)	-2.3 (-21.2 to 20.0)	304.2 (195.7 to 448.0)	-3.8 (-24.2 to 21.2)	67.4 (48.1 to 92.1)	1.4 (-18.9 to 25.0)	1781.7 (1230.2 to 2515.8)	0.8 (-21.1 to 26.4)	15.8 (8.3 to 26.1)	16.6 (-7.2 to 47.4)	372.5 (196.2 to 621.3)	17.3 (-8.8 to 52.3)
Namibia	41.4 (31.9 to 53.0)	5.1 (-13.8 to 27.8)	1023.7 (760.0 to 1373.4)	3.6 (-17.7 to 30.5)	5.5 (3.8 to 7.3)	1.1 (-16.5 to 22.7)	120.6 (81.9 to 168.1)	-0.7 (-19.7 to 24.1)	32.8 (25.7 to 42.0)	2.5 (-15.9 to 24.7)	827.4 (617.7 to 1107.4)	1.3 (-19.8 to 27.9)	7.8 (4.1 to 13.1)	21.2 (-1.3 to 50.2)	172.5 (90.9 to 293.7)	20.7 (-3.5 to 52.3)
South Africa	57.3 (50.8 to 64.8)	-17.8 (-24.2 to -9.5)	1373.2 (1221.1 to 1562.4)	-20.3 (-27.1 to -11.4)	8.4 (6.7 to 10.4)	-21.3 (-31.7 to -9.0)	187.0 (147.8 to 233.6)	-25.2 (-35.7 to -12.7)	44.2 (40.3 to 48.9)	-20.6 (-26.6 to -12.6)	1089.8 (989.1 to 1215.0)	-22.6 (-29.5 to -13.6)	15.2 (8.9 to 22.5)	-9.4 (-16.2 to -0.5)	330.2 (197.9 to 486.9)	-11.5 (-19.1 to -1.8)
Zimbabwe	72.1 (57.2 to 89.5)	-5.7 (-20.5 to 11.2)	1831.2 (1411.4 to 2300.5)	-6.7 (-23.3 to 11.9)	6.2 (4.4 to 8.1)	-5.1 (-22.3 to 14.2)	149.7 (105.7 to 201.5)	-6.2 (-24.8 to 14.0)	59.2 (46.7 to 72.4)	-8.4 (-22.9 to 7.4)	1523.6 (1173.6 to 1920.3)	-9.0 (-25.4 to 8.7)	14.4 (7.4 to 24.0)	10.8 (-8.8 to 33.8)	336.4 (176.8 to 554.4)	9.3 (-11.0 to 34.3)
**Western sub-Saharan Africa**	**30.6 (25.1 to 36.8)**	**2.2 (-9.8 to 15.3)**	**754.1 (616.1 to 913.5)**	**1.3 (-12.7 to 17.2)**	**4.2 (3.2 to 5.3)**	**-0.4 (-14.3 to 16.4)**	**92.9 (71.2 to 119.0)**	**-0.9 (-16.0 to 17.4)**	**23.5 (19.7 to 27.4)**	**-1.0 (-12.7 to 12.2)**	**597.2 (490.3 to 713.4)**	**-1.6 (-14.9 to 14.9)**	**5.9 (3.0 to 9.6)**	**21.8 (8.7 to 38.8)**	**127.7 (65.6 to 206.3)**	**21.8 (7.4 to 39.8)**
Benin	35.3 (27.2 to 45.3)	-3.1 (-17.0 to 12.8)	875.8 (653.3 to 1154.5)	-4.0 (-19.9 to 14.8)	4.9 (3.4 to 6.9)	-2.2 (-20.1 to 19.1)	112.0 (77.6 to 159.7)	-2.7 (-21.8 to 19.4)	27.3 (21.2 to 35.0)	-6.3 (-19.9 to 9.7)	689.5 (516.3 to 912.5)	-7.0 (-23.0 to 12.1)	6.9 (3.5 to 11.6)	12.0 (-4.3 to 32.3)	159.3 (81.7 to 261.5)	10.9 (-7.4 to 34.0)
Burkina Faso	36.0 (29.3 to 45.0)	12.7 (-1.3 to 32.1)	927.5 (727.9 to 1178.6)	13.5 (-3.9 to 37.0)	4.8 (3.3 to 7.1)	11.3 (-7.5 to 34.8)	109.3 (74.5 to 164.4)	13.2 (-7.7 to 38.9)	28.8 (23.4 to 35.8)	10.4 (-4.0 to 29.7)	769.7 (600.4 to 974.0)	11.2 (-6.2 to 35.7)	5.4 (2.4 to 9.3)	29.1 (13.4 to 51.8)	116.3 (53.5 to 197.9)	33.6 (15.0 to 61.7)
Cameroon	41.8 (30.4 to 55.7)	-2.5 (-18.7 to 17.4)	1039.2 (730.9 to 1426.0)	-4.7 (-22.9 to 18.5)	6.3 (4.3 to 9.0)	-1.0 (-21.6 to 23.2)	144.2 (95.4 to 209.9)	-2.3 (-23.6 to 23.4)	31.1 (22.9 to 41.2)	-6.4 (-21.9 to 14.1)	803.2 (564.5 to 1103.0)	-8.3 (-26.0 to 15.7)	9.3 (4.8 to 15.7)	14.8 (-3.8 to 37.4)	202.6 (105.5 to 339.9)	13.6 (-6.9 to 39.1)
Cape Verde	53.3 (43.4 to 65.9)	34.7 (14.8 to 54.5)	1212.6 (988.5 to 1496.0)	25.0 (5.6 to 45.8)	6.5 (4.4 to 9.0)	49.1 (11.7 to 90.5)	138.7 (97.2 to 190.3)	39.8 (8.4 to 76.1)	38.8 (32.6 to 46.4)	28.8 (9.8 to 47.1)	898.7 (748.3 to 1082.5)	19.4 (1.3 to 39.3)	14.9 (7.9 to 24.1)	56.6 (30.9 to 85.6)	328.2 (181.2 to 522.6)	48.8 (23.5 to 79.7)
Chad	36.5 (28.4 to 46.4)	-2.2 (-15.4 to 14.4)	923.9 (705.0 to 1187.6)	-3.5 (-18.4 to 15.7)	5.7 (3.6 to 8.9)	0.2 (-19.1 to 24.0)	128.2 (83.0 to 201.5)	-1.2 (-21.6 to 24.5)	30.4 (23.6 to 37.9)	-4.3 (-17.6 to 12.5)	785.1 (596.6 to 1000.3)	-5.5 (-20.3 to 13.8)	4.4 (1.8 to 8.0)	14.6 (-0.5 to 39.3)	95.3 (40.6 to 168.3)	15.3 (-2.3 to 42.0)
Côte d'Ivoire	39.1 (29.8 to 49.3)	-2.0 (-17.3 to 17.4)	958.6 (701.9 to 1247.7)	-3.3 (-21.4 to 20.2)	5.8 (4.1 to 8.2)	-1.5 (-21.4 to 23.1)	132.5 (89.8 to 187.6)	-2.2 (-23.6 to 25.9)	31.5 (24.4 to 39.7)	-4.0 (-19.3 to 15.5)	787.0 (580.6 to 1027.3)	-5.2 (-23.3 to 18.7)	7.0 (3.4 to 12.0)	12.7 (-4.8 to 35.7)	155.2 (79.4 to 263.4)	12.8 (-8.0 to 40.6)
The Gambia	40.0 (30.5 to 51.1)	7.3 (-11.9 to 30.3)	1054.9 (775.8 to 1386.8)	7.4 (-14.4 to 34.4)	3.6 (2.5 to 4.9)	7.0 (-13.9 to 30.3)	81.3 (56.2 to 114.8)	7.2 (-15.6 to 33.5)	31.2 (23.6 to 39.3)	3.5 (-15.5 to 26.1)	826.5 (599.3 to 1061.8)	3.6 (-17.6 to 30.5)	9.6 (4.8 to 16.3)	23.6 (-0.6 to 56.7)	254.4 (124.5 to 452.7)	23.7 (-3.8 to 61.7)
Ghana	34.6 (27.1 to 43.8)	3.5 (-10.7 to 21.5)	858.8 (667.3 to 1098.2)	1.5 (-14.9 to 22.8)	3.9 (2.9 to 5.1)	3.5 (-13.9 to 25.3)	92.5 (68.4 to 122.1)	3.3 (-14.9 to 27.3)	23.9 (19.2 to 29.7)	-0.7 (-15.3 to 17.7)	613.8 (476.8 to 790.7)	-2.9 (-20.2 to 18.6)	9.9 (5.7 to 15.9)	17.1 (0.5 to 42.6)	220.3 (124.9 to 345.5)	18.1 (-0.4 to 44.8)
Guinea	52.9 (41.2 to 65.8)	-0.3 (-16.8 to 19.7)	1410.7 (1076.3 to 1786.6)	-0.5 (-18.4 to 21.9)	4.8 (3.3 to 6.7)	-0.3 (-18.2 to 23.9)	115.2 (79.5 to 162.1)	-1.1 (-20.3 to 23.9)	45.1 (35.1 to 55.9)	-1.9 (-18.7 to 18.9)	1222.0 (940.4 to 1557.8)	-1.9 (-20.1 to 20.6)	7.4 (3.4 to 13.0)	15.9 (-3.4 to 39.7)	175.7 (81.5 to 310.6)	17.1 (-4.2 to 45.2)
Guinea-Bissau	43.4 (31.4 to 57.3)	-2.6 (-18.1 to 16.3)	1167.7 (819.9 to 1565.9)	-5.1 (-21.9 to 15.2)	6.2 (3.9 to 9.5)	-3.1 (-20.1 to 19.5)	149.4 (89.4 to 236.0)	-4.5 (-23.1 to 19.4)	34.3 (24.8 to 45.5)	-4.0 (-20.0 to 15.1)	953.4 (665.0 to 1291.9)	-6.5 (-23.3 to 15.0)	6.5 (2.7 to 11.8)	13.0 (-4.5 to 36.1)	148.2 (64.4 to 265.5)	11.9 (-6.8 to 37.5)
Liberia	33.6 (24.9 to 44.0)	-3.0 (-19.1 to 16.3)	841.9 (613.2 to 1126.4)	-3.8 (-22.2 to 18.4)	4.3 (2.8 to 6.4)	-2.3 (-22.6 to 22.5)	97.2 (62.6 to 145.7)	-1.9 (-23.0 to 24.7)	25.5 (19.2 to 33.5)	-5.8 (-22.1 to 14.1)	656.9 (475.6 to 875.7)	-6.5 (-24.6 to 15.9)	7.3 (3.7 to 12.4)	12.0 (-7.4 to 38.2)	168.9 (89.3 to 279.7)	11.9 (-9.3 to 40.5)
Mali	33.1 (25.7 to 42.7)	2.4 (-11.9 to 20.6)	848.9 (646.0 to 1120.2)	2.0 (-14.2 to 23.1)	3.5 (2.3 to 5.0)	1.4 (-15.9 to 22.6)	80.3 (53.5 to 118.1)	2.1 (-16.5 to 24.9)	26.5 (21.0 to 34.1)	0.9 (-13.4 to 19.0)	693.0 (531.9 to 915.1)	0.4 (-15.6 to 21.8)	5.9 (2.7 to 10.5)	15.6 (-0.7 to 37.2)	137.9 (62.6 to 240.5)	16.5 (-2.7 to 41.4)
Mauritania	33.0 (25.1 to 42.0)	-2.4 (-22.5 to 16.2)	787.6 (568.1 to 1027.5)	-5.4 (-28.2 to 16.4)	4.5 (3.0 to 6.5)	0.7 (-21.8 to 23.9)	97.9 (63.7 to 147.7)	-1.7 (-26.4 to 23.9)	24.8 (18.8 to 31.4)	-6.1 (-25.9 to 12.5)	611.1 (439.0 to 801.8)	-8.6 (-31.0 to 12.8)	7.4 (4.3 to 11.8)	15.0 (-7.8 to 38.0)	158.7 (90.5 to 251.5)	11.7 (-14.2 to 36.3)
Niger	27.7 (20.8 to 36.2)	6.1 (-10.4 to 26.6)	706.8 (521.6 to 937.2)	6.6 (-12.4 to 31.4)	4.6 (2.6 to 7.8)	5.2 (-13.7 to 29.5)	102.9 (57.3 to 171.1)	6.5 (-14.8 to 34.1)	22.3 (16.7 to 28.7)	4.5 (-11.3 to 25.3)	586.4 (430.9 to 774.8)	5.1 (-13.7 to 30.4)	2.9 (1.2 to 5.3)	29.3 (9.5 to 54.9)	61.9 (26.3 to 113.9)	30.8 (6.7 to 61.6)
Nigeria	23.2 (17.7 to 29.5)	3.1 (-19.0 to 34.4)	554.3 (408.3 to 717.3)	2.5 (-21.8 to 39.9)	3.2 (2.3 to 4.3)	-5.9 (-29.9 to 29.8)	69.5 (49.3 to 94.7)	-6.6 (-32.9 to 31.1)	17.3 (13.5 to 21.7)	-0.6 (-22.1 to 30.6)	429.3 (324.5 to 557.7)	-0.7 (-25.4 to 37.6)	4.6 (2.0 to 8.0)	29.9 (2.9 to 68.4)	95.2 (43.5 to 166.6)	29.9 (-0.3 to 75.2)
São Tomé and Príncipe	48.3 (38.3 to 60.1)	6.5 (-8.6 to 20.7)	1175.0 (918.8 to 1485.8)	3.3 (-14.0 to 20.7)	7.2 (5.0 to 9.8)	4.3 (-14.1 to 23.4)	161.6 (110.8 to 222.0)	4.0 (-16.4 to 24.0)	35.8 (28.2 to 44.4)	3.8 (-11.5 to 18.5)	899.4 (693.4 to 1131.8)	0.4 (-16.7 to 18.8)	10.7 (5.6 to 18.1)	24.8 (8.5 to 44.2)	229.4 (122.5 to 381.6)	23.9 (5.4 to 45.3)
Senegal	36.0 (28.2 to 45.9)	5.9 (-11.8 to 26.2)	877.6 (667.5 to 1133.3)	5.5 (-15.2 to 29.1)	5.6 (3.9 to 7.7)	9.5 (-12.2 to 34.6)	125.6 (86.4 to 173.7)	9.5 (-14.2 to 38.3)	27.5 (21.4 to 34.4)	2.2 (-15.4 to 23.0)	692.9 (526.4 to 890.5)	1.9 (-18.9 to 25.6)	7.5 (3.4 to 13.0)	17.3 (-2.4 to 41.1)	157.7 (75.3 to 274.2)	19.5 (-3.8 to 46.5)
Sierra Leone	35.8 (27.2 to 46.1)	0.4 (-16.2 to 22.3)	907.1 (673.9 to 1183.5)	-0.3 (-18.1 to 24.5)	5.0 (3.3 to 7.2)	2.9 (-17.2 to 30.3)	112.5 (73.5 to 167.1)	3.4 (-18.2 to 32.4)	30.2 (22.9 to 38.9)	-2.0 (-18.0 to 19.9)	780.2 (578.6 to 1019.5)	-2.7 (-20.4 to 21.9)	4.1 (1.9 to 7.2)	26.5 (6.0 to 60.6)	93.2 (43.6 to 161.9)	29.5 (6.1 to 68.1)
Togo	38.0 (29.9 to 48.2)	-2.8 (-18.4 to 16.3)	946.8 (722.8 to 1240.3)	-3.7 (-21.5 to 17.8)	5.1 (3.5 to 7.2)	-2.9 (-20.8 to 18.8)	119.3 (81.7 to 170.8)	-3.5 (-23.5 to 20.8)	31.6 (25.0 to 40.2)	-5.7 (-21.1 to 13.0)	799.7 (611.1 to 1050.3)	-6.4 (-24.1 to 15.5)	5.4 (2.7 to 9.1)	18.9 (0.1 to 43.5)	121.0 (61.2 to 199.8)	19.8 (-1.7 to 47.5)

Results are for both sexes combined. Data in parentheses are 95% uncertainty intervals (UIs). ASR = age-standardised rates; % change = percent change of age-standardised rates between 2010 and 2019; DALY = disability-adjusted life-year.

* indicates risk factors measured in this study

Appendix Table 30: Global numbers and age-standardised rates of risk-attributable total cancer deaths and DALYs, 2010 and 2019, and percentage change in global numbers and age-standardised rates of risk-attributable total cancer deaths and cancer DALYs, 2010-2019, both sexes combined

GBD Risk Level	Risk factor	Both sexes combined											
		Deaths						DALYs					
		Deaths in 2010, thousands (95% UI)	Age-standardised death rates, per 100,000, in 2010 (95% UI)	Deaths in 2019, thousands (95% UI)	Age-standardised death rates, per 100,000, in 2019 (95% UI)	Percent change in absolute deaths, 2010 – 2019 (95% UI)	Percent change in age-standardised death rates, 2010 – 2019 (95% UI)	DALYs in 2010, thousands (95% UI)	Age-standardised DALY rates, per 100,000, in 2010 (95% UI)	DALYs in 2019, thousands (95% UI)	Age-standardised DALY rates, per 100,000, in 2019 (95% UI)	Percent change in absolute DALYs, 2010 – 2019 (95% UI)	Percent change in age-standardised DALY rates, 2010 – 2019 (95% UI)
**0**	**All risk factors**	**3 700 (3 410 to 4 050)**	**59.0 (54.3 to 64.7)**	**4 450 (4 010 to 4 940)**	**54.9 (49.3 to 61.0)**	**20.4 (12.6 to 28.4)**	**-6.9 (-12.8 to -0.9)**	**89 900 (83 300 to 98 100)**	**1 369.2 (1 267.3 to 1 495.4)**	**105 000 (95 000 to 116 000)**	**1 262.7 (1 142.8 to 1 398.7)**	**16.8 (8.8 to 25.0)**	**-7.8 (-14.0 to -1.4)**
**1**	**Environmental/ occupational risks**	**631 (538 to 726)**	**10.1 (8.6 to 11.6)**	**737 (619 to 859)**	**9.1 (7.7 to 10.6)**	**16.7 (7.9 to 26.2)**	**-10.0 (-16.7 to -2.8)**	**14 400 (12 300 to 16 600)**	**221.2 (189.2 to 254.9)**	**16 300 (13 700 to 19 100)**	**196.1 (165.5 to 230.3)**	**13.1 (3.9 to 23.1)**	**-11.4 (-18.5 to -3.5)**
2	Air pollution	336 (256 to 412)	5.3 (4.0 to 6.5)	387 (288 to 490)	4.7 (3.5 to 6.0)	15.5 (3.4 to 28.3)	-10.5 (-19.8 to -0.8)	8 030 (6 110 to 9 850)	122.4 (93.2 to 150.2)	8 950 (6 680 to 11 300)	107.4 (80.1 to 136.0)	11.5 (-0.5 to 24.4)	-12.3 (-21.7 to -2.3)
3	Particulate matter pollution	336 (256 to 412)	5.3 (4.0 to 6.5)	387 (288 to 490)	4.7 (3.5 to 6.0)	15.5 (3.4 to 28.3)	-10.5 (-19.8 to -0.8)	8 030 (6 110 to 9 850)	122.4 (93.2 to 150.2)	8 950 (6 680 to 11 300)	107.4 (80.1 to 136.0)	11.5 (-0.5 to 24.4)	-12.3 (-21.7 to -2.3)
4	Ambient particulate matter pollution	240 (175 to 303)	3.8 (2.8 to 4.8)	308 (227 to 396)	3.8 (2.8 to 4.9)	28.4 (15.6 to 44.3)	-0.6 (-10.4 to 11.4)	5 650 (4 130 to 7 130)	86.3 (63.0 to 109.0)	7 020 (5 180 to 9 020)	84.2 (62.1 to 108.3)	24.2 (11.1 to 40.3)	-2.4 (-12.5 to 10.1)
4	Household air pollution from solid fuels	96.0 (58.9 to 142)	1.5 (0.9 to 2.2)	79.8 (45.1 to 125)	1.0 (0.5 to 1.5)	-16.9 (-28.8 to -3.5)	-35.5 (-44.6 to -25.1)	2 390 (1 460 to 3 510)	36.1 (22.1 to 53.3)	1 940 (1 110 to 3 010)	23.1 (13.2 to 36.0)	-18.8 (-30.2 to -6.1)	-35.9 (-44.8 to -25.9)
2	Other environmental risks	69.8 (13.6 to 135)	1.1 (0.2 to 2.2)	83.7 (16.5 to 162)	1.0 (0.2 to 2.0)	20.0 (12.1 to 29.0)	-7.4 (-13.4 to -0.5)	1 630 (320 to 3 150)	24.9 (4.9 to 48.3)	1 890 (374 to 3 650)	22.7 (4.5 to 43.9)	15.9 (8.0 to 25.3)	-9.1 (-15.2 to -1.7)
3	Residential radon	69.8 (13.6 to 135)	1.1 (0.2 to 2.2)	83.7 (16.5 to 162)	1.0 (0.2 to 2.0)	20.0 (12.1 to 29.0)	-7.4 (-13.4 to -0.5)	1 630 (320 to 3 150)	24.9 (4.9 to 48.3)	1 890 (374 to 3 650)	22.7 (4.5 to 43.9)	15.9 (8.0 to 25.3)	-9.1 (-15.2 to -1.7)
2	Occupational risks	289 (228 to 350)	4.7 (3.7 to 5.7)	334 (263 to 405)	4.2 (3.3 to 5.1)	15.6 (8.8 to 22.6)	-11.4 (-16.5 to -6.2)	6 160 (4 860 to 7 510)	95.9 (75.6 to 116.8)	6 960 (5 470 to 8 580)	84.4 (66.2 to 103.7)	13.0 (5.5 to 20.6)	-12.0 (-17.7 to -6.1)
3	Occupational carcinogens	289 (228 to 350)	4.7 (3.7 to 5.7)	334 (263 to 405)	4.2 (3.3 to 5.1)	15.6 (8.8 to 22.6)	-11.4 (-16.5 to -6.2)	6 160 (4 860 to 7 510)	95.9 (75.6 to 116.8)	6 960 (5 470 to 8 580)	84.4 (66.2 to 103.7)	13.0 (5.5 to 20.6)	-12.0 (-17.7 to -6.1)
4	Occupational exposure to asbestos	210 (155 to 266)	3.5 (2.6 to 4.5)	236 (176 to 296)	3.0 (2.2 to 3.8)	12.0 (5.8 to 18.2)	-14.6 (-19.2 to -10.1)	3 800 (2 800 to 4 840)	61.1 (45.0 to 77.6)	4 120 (3 060 to 5 240)	50.9 (37.8 to 64.7)	8.2 (1.5 to 15.4)	-16.7 (-21.8 to -11.5)
4	Occupational exposure to arsenic	7.89 (1.17 to 14.4)	0.1 (0.0 to 0.2)	9.76 (1.55 to 17.7)	0.1 (0.0 to 0.2)	23.6 (11.4 to 43.7)	-3.3 (-12.7 to 12.7)	227 (36.2 to 411)	3.4 (0.5 to 6.1)	271 (44.8 to 486)	3.2 (0.5 to 5.7)	19.4 (7.3 to 38.6)	-5.5 (-15.0 to 10.2)
4	Occupational exposure to benzene	1.63 (0.513 to 2.64)	0.0 (0.0 to 0.0)	1.87 (0.565 to 3.05)	0.0 (0.0 to 0.0)	14.2 (6.4 to 22.5)	-0.8 (-7.4 to 6.7)	77.4 (23.7 to 126)	1.1 (0.3 to 1.7)	85.8 (25.7 to 140)	1.1 (0.3 to 1.7)	10.9 (3.0 to 19.6)	-1.3 (-8.3 to 6.4)
4	Occupational exposure to beryllium	0.233 (0.192 to 0.276)	0.0 (0.0 to 0.0)	0.301 (0.244 to 0.367)	0.0 (0.0 to 0.0)	28.7 (13.6 to 44.8)	0.9 (-10.7 to 13.5)	6.90 (5.68 to 8.15)	0.1 (0.1 to 0.1)	8.58 (6.95 to 10.5)	0.1 (0.1 to 0.1)	24.4 (9.8 to 40.0)	-1.1 (-12.5 to 10.8)
4	Occupational exposure to cadmium	0.549 (0.455 to 0.642)	0.0 (0.0 to 0.0)	0.712 (0.583 to 0.854)	0.0 (0.0 to 0.0)	29.7 (14.3 to 46.2)	1.7 (-10.3 to 14.0)	16.2 (13.6 to 18.9)	0.2 (0.2 to 0.3)	20.3 (16.7 to 24.1)	0.2 (0.2 to 0.3)	25.3 (10.2 to 41.2)	-0.5 (-12.3 to 11.7)
4	Occupational exposure to chromium	1.14 (1.00 to 1.28)	0.0 (0.0 to 0.0)	1.50 (1.29 to 1.75)	0.0 (0.0 to 0.0)	31.8 (17.0 to 47.6)	3.4 (-8.5 to 15.6)	33.5 (29.4 to 37.6)	0.5 (0.4 to 0.6)	42.7 (36.6 to 49.7)	0.5 (0.4 to 0.6)	27.4 (13.0 to 42.9)	1.2 (-10.2 to 13.1)
4	Occupational exposure to diesel engine exhaust	14.7 (12.9 to 16.9)	0.2 (0.2 to 0.3)	19.7 (17.0 to 22.9)	0.2 (0.2 to 0.3)	33.7 (20.0 to 48.8)	4.8 (-5.8 to 16.9)	435 (380 to 497)	6.4 (5.6 to 7.4)	563 (485 to 655)	6.6 (5.7 to 7.7)	29.5 (15.9 to 44.1)	2.9 (-7.6 to 14.8)
4	Occupational exposure to formaldehyde	1.01 (0.818 to 1.22)	0.0 (0.0 to 0.0)	1.12 (0.900 to 1.36)	0.0 (0.0 to 0.0)	11.0 (0.4 to 22.1)	-3.9 (-12.6 to 5.6)	46.9 (38.1 to 57.5)	0.6 (0.5 to 0.8)	50.8 (40.9 to 61.7)	0.6 (0.5 to 0.8)	8.4 (-1.5 to 19.3)	-4.2 (-12.6 to 5.3)

GBD Risk Level	Risk factor	Both sexes combined											
		Deaths						DALYs					
		Deaths in 2010, thousands (95% UI)	Age-standardised death rates, per 100,000, in 2010 (95% UI)	Deaths in 2019, thousands (95% UI)	Age-standardised death rates, per 100,000, in 2019 (95% UI)	Percent change in absolute deaths, 2010 – 2019 (95% UI)	Percent change in age-standardised death rates, 2010 – 2019 (95% UI)	DALYs in 2010, thousands (95% UI)	Age-standardised DALY rates, per 100,000, in 2010 (95% UI)	DALYs in 2019, thousands (95% UI)	Age-standardised DALY rates, per 100,000, in 2019 (95% UI)	Percent change in absolute DALYs, 2010 – 2019 (95% UI)	Percent change in age-standardised DALY rates, 2010 – 2019 (95% UI)
4	Occupational exposure to nickel	7.60 (0.295 to 20.6)	0.1 (0.0 to 0.3)	9.33 (0.536 to 24.6)	0.1 (0.0 to 0.3)	22.8 (9.1 to 48.4)	-3.9 (-14.9 to 17.1)	220 (11.6 to 589)	3.3 (0.2 to 8.8)	261 (18.3 to 677)	3.1 (0.2 to 8.0)	18.6 (5.2 to 42.6)	-6.0 (-16.8 to 14.6)
4	Occupational exposure to polycyclic aromatic hydrocarbons	4.00 (3.36 to 4.68)	0.1 (0.1 to 0.1)	5.27 (4.36 to 6.24)	0.1 (0.1 to 0.1)	31.8 (17.6 to 47.8)	3.3 (-8.0 to 15.7)	118 (99.4 to 139)	1.7 (1.5 to 2.0)	150 (123 to 177)	1.8 (1.5 to 2.1)	27.4 (13.7 to 42.8)	1.2 (-9.6 to 13.2)
4	Occupational exposure to silica	43.4 (19.1 to 68.8)	0.7 (0.3 to 1.0)	53.0 (23.8 to 84.4)	0.6 (0.3 to 1.0)	22.0 (11.7 to 33.7)	-4.6 (-12.7 to 4.6)	1 250 (552 to 1 980)	18.6 (8.2 to 29.4)	1 480 (666 to 2 350)	17.4 (7.8 to 27.7)	18.0 (7.7 to 29.5)	-6.6 (-14.8 to 2.5)
4	Occupational exposure to sulfuric acid	3.41 (1.45 to 6.18)	0.1 (0.0 to 0.1)	4.03 (1.73 to 7.47)	0.0 (0.0 to 0.1)	18.1 (7.8 to 29.1)	-6.7 (-14.9 to 1.9)	109 (46.3 to 198)	1.6 (0.7 to 2.9)	126 (54.1 to 234)	1.5 (0.6 to 2.7)	15.6 (5.7 to 26.4)	-7.4 (-15.3 to 1.3)
4	Occupational exposure to trichloroethylene	0.0559 (0.0122 to 0.104)	0.0 (0.0 to 0.0)	0.0785 (0.0168 to 0.147)	0.0 (0.0 to 0.0)	40.5 (29.8 to 52.7)	11.2 (2.7 to 20.8)	1.77 (0.388 to 3.31)	0.0 (0.0 to 0.0)	2.43 (0.518 to 4.54)	0.0 (0.0 to 0.1)	37.5 (26.4 to 49.5)	10.7 (1.7 to 20.4)
**1**	**Behavioural risks**	**3 140 (2 950 to 3 350)**	**49.9 (46.8 to 53.2)**	**3 700 (3 420 to 4 020)**	**45.5 (42.1 to 49.4)**	**17.9 (10.4 to 26.0)**	**-8.7 (-14.5 to -2.7)**	**76 700 (72 600 to 81 900)**	**1 166.8 (1 103.0 to 1 245.7)**	**87 800 (81 100 to 95 400)**	**1 054.7 (974.1 to 1 145.3)**	**14.4 (6.5 to 22.5)**	**-9.6 (-15.8 to -3.2)**
2	Tobacco	2 240 (2 100 to 2 350)	35.6 (33.4 to 37.5)	2 600 (2 380 to 2 830)	31.9 (29.2 to 34.7)	16.2 (7.5 to 25.6)	-10.4 (-16.9 to -3.2)	52 700 (49 600 to 55 600)	807.4 (760.5 to 851.2)	59 300 (54 000 to 64 800)	711.7 (648.9 to 777.1)	12.6 (3.7 to 22.3)	-11.9 (-18.8 to -4.3)
3	Smoking	2 160 (2 030 to 2 270)	34.4 (32.2 to 36.3)	2 490 (2 280 to 2 720)	30.6 (28.0 to 33.3)	15.6 (6.9 to 25.3)	-10.9 (-17.6 to -3.5)	50 400 (47 600 to 53 300)	774.1 (729.9 to 818.1)	56 400 (51 300 to 61 700)	677.3 (616.4 to 740.3)	11.9 (2.8 to 21.8)	-12.5 (-19.6 to -4.8)
3	Chewing tobacco	41.2 (33.0 to 50.0)	0.6 (0.5 to 0.8)	55.6 (43.1 to 68.8)	0.7 (0.5 to 0.8)	34.9 (19.0 to 52.4)	6.2 (-6.2 to 19.9)	1 160 (920 to 1 420)	17.2 (13.7 to 21.0)	1 500 (1 160 to 1 880)	17.9 (13.9 to 22.4)	29.8 (13.6 to 47.9)	4.3 (-8.6 to 18.7)
3	Secondhand smoke	105 (68.6 to 151)	1.7 (1.1 to 2.4)	130 (82.6 to 190)	1.6 (1.0 to 2.3)	23.8 (12.8 to 35.8)	-3.5 (-12.0 to 5.8)	2 700 (1 760 to 3 830)	40.7 (26.6 to 57.9)	3 220 (2 070 to 4 630)	38.5 (24.8 to 55.5)	19.3 (8.7 to 30.9)	-5.2 (-13.7 to 4.0)
2	Alcohol use	406 (364 to 452)	6.3 (5.7 to 7.1)	495 (440 to 554)	6.0 (5.4 to 6.8)	21.8 (12.7 to 31.5)	-5.0 (-11.9 to 2.4)	11 000 (9 910 to 12 200)	164.4 (148.3 to 182.3)	13 000 (11 600 to 14 500)	155.2 (138.4 to 173.5)	18.3 (8.9 to 28.2)	-5.6 (-12.9 to 2.2)
2	Drug use	52.1 (41.6 to 64.4)	0.8 (0.7 to 1.0)	71.5 (57.1 to 89.2)	0.9 (0.7 to 1.1)	37.1 (27.3 to 47.8)	6.5 (-0.8 to 14.8)	1 230 (987 to 1 510)	18.8 (15.0 to 23.1)	1 610 (1 290 to 1 990)	19.4 (15.6 to 23.9)	31.1 (20.9 to 42.1)	3.2 (-4.7 to 11.6)
2	Dietary risks	516 (385 to 720)	8.4 (6.2 to 11.6)	605 (454 to 811)	7.6 (5.7 to 10.1)	17.3 (8.5 to 25.5)	-9.5 (-15.9 to -3.5)	12 300 (9 010 to 17 200)	187.7 (138.0 to 262.9)	14 000 (10 500 to 18 800)	168.8 (127.1 to 226.9)	13.6 (4.6 to 22.3)	-10.1 (-17.1 to -3.3)
3	Diet low in fruits	119 (59.7 to 187)	1.9 (0.9 to 3.0)	128 (65.0 to 200)	1.6 (0.8 to 2.5)	8.1 (-3.3 to 19.9)	-16.2 (-24.9 to -7.3)	2 870 (1 470 to 4 510)	43.6 (22.1 to 68.7)	3 000 (1 540 to 4 680)	36.0 (18.5 to 56.2)	4.5 (-6.8 to 16.3)	-17.5 (-26.5 to -8.1)
3	Diet low in vegetables	15.1 (2.28 to 29.7)	0.2 (0.0 to 0.5)	17.2 (2.55 to 34.0)	0.2 (0.0 to 0.4)	14.1 (5.4 to 23.3)	-12.1 (-18.7 to -4.5)	371 (58.1 to 729)	5.6 (0.9 to 11.1)	420 (64.2 to 828)	5.0 (0.8 to 9.9)	13.2 (4.2 to 22.3)	-10.6 (-17.9 to -3.3)
3	Diet low in whole grains	137 (52.9 to 178)	2.3 (0.9 to 2.9)	171 (66.7 to 225)	2.2 (0.8 to 2.8)	25.5 (18.9 to 32.0)	-3.9 (-8.8 to 0.8)	3 110 (1 190 to 4 090)	48.1 (18.4 to 63.0)	3 810 (1 460 to 5 020)	46.3 (17.8 to 61.1)	22.4 (15.3 to 29.5)	-3.6 (-9.1 to 1.9)
3	Diet low in milk	126 (80.9 to 171)	2.1 (1.3 to 2.8)	166 (107 to 226)	2.1 (1.3 to 2.8)	32.4 (24.5 to 41.0)	1.6 (-4.3 to 8.1)	2 950 (1 920 to 3 990)	45.3 (29.4 to 61.2)	3 800 (2 460 to 5 120)	46.1 (29.8 to 62.2)	28.7 (20.5 to 37.7)	1.7 (-4.8 to 8.9)
3	Diet high in red meat	59.2 (28.1 to 99.5)	1.0 (0.4 to 1.6)	75.3 (35.9 to 126)	0.9 (0.4 to 1.6)	27.2 (20.2 to 36.6)	-1.9 (-7.3 to 5.5)	1 520 (779 to 2 450)	23.1 (11.7 to 37.4)	1 890 (964 to 3 000)	22.8 (11.6 to 36.4)	23.8 (15.9 to 32.7)	-1.3 (-7.3 to 6.1)
3	Diet high in processed meat	29.1 (10.6 to 44.7)	0.5 (0.2 to 0.7)	33.9 (11.6 to 52.1)	0.4 (0.1 to 0.7)	16.8 (8.2 to 24.4)	-11.1 (-17.2 to -5.2)	643 (242 to 986)	10.0 (3.7 to 15.4)	735 (263 to 1 130)	9.0 (3.2 to 13.7)	14.3 (5.0 to 22.7)	-10.5 (-17.7 to -3.8)
3	Diet low in fibre	17.9 (7.28 to 34.3)	0.3 (0.1 to 0.6)	20.5 (8.21 to 39.8)	0.3 (0.1 to 0.5)	14.6 (8.4 to 21.1)	-12.2 (-16.5 to -7.5)	409 (167 to 773)	6.3 (2.6 to 11.9)	449 (178 to 858)	5.5 (2.2 to 10.5)	9.8 (2.6 to 17.1)	-12.7 (-18.0 to -7.2)
3	Diet low in calcium	111 (79.9 to 151)	1.8 (1.3 to 2.5)	138 (96.8 to 189)	1.7 (1.2 to 2.4)	23.8 (15.3 to 32.1)	-4.5 (-10.7 to 1.7)	2 620 (1 920 to 3 510)	40.2 (29.1 to 53.9)	3 140 (2 250 to 4 260)	38.2 (27.2 to 51.8)	19.8 (10.7 to 28.8)	-5.0 (-11.8 to 1.9)
3	Diet high in sodium	72.3 (2.04 to 285)	1.2 (0.0 to 4.5)	74.1 (2.12 to 295)	0.9 (0.0 to 3.6)	2.4 (-8.2 to 13.0)	-20.3 (-28.4 to -12.2)	1 770 (48.9 to 6 950)	26.8 (0.7 to 105.5)	1 740 (48.7 to 6 800)	20.9 (0.6 to 82.1)	-1.9 (-12.7 to 8.9)	-22.0 (-30.6 to -13.4)
2	Unsafe sex	238 (211 to 269)	3.6 (3.2 to 4.1)	280 (239 to 314)	3.4 (2.9 to 3.8)	17.9 (8.5 to 28.6)	-5.2 (-12.7 to 3.2)	7 820 (6 850 to 8 680)	112.6 (99.2 to 125.4)	8 960 (7 550 to 9 980)	107.2 (90.5 to 119.4)	14.6 (5.0 to 25.0)	-4.8 (-12.7 to 3.7)

GBD Risk Level	Risk factor	Both sexes combined											
		Deaths						DALYs					
		Deaths in 2010, thousands (95% UI)	Age-standardised death rates, per 100,000, in 2010 (95% UI)	Deaths in 2019, thousands (95% UI)	Age-standardised death rates, per 100,000, in 2019 (95% UI)	Percent change in absolute deaths, 2010 – 2019 (95% UI)	Percent change in age-standardised death rates, 2010 – 2019 (95% UI)	DALYs in 2010, thousands (95% UI)	Age-standardised DALY rates, per 100,000, in 2010 (95% UI)	DALYs in 2019, thousands (95% UI)	Age-standardised DALY rates, per 100,000, in 2019 (95% UI)	Percent change in absolute DALYs, 2010 – 2019 (95% UI)	Percent change in age-standardised DALY rates, 2010 – 2019 (95% UI)
2	Low physical activity	52.9 (20.1 to 94.5)	0.9 (0.4 to 1.6)	67.1 (25.8 to 122)	0.9 (0.3 to 1.6)	26.8 (19.7 to 33.5)	-5.1 (-10.5 to -0.1)	966 (370 to 1 720)	15.6 (6.0 to 27.9)	1 200 (455 to 2 160)	15.0 (5.7 to 26.9)	24.5 (17.1 to 31.7)	-4.2 (-9.9 to 1.3)
**1**	**Metabolic risks**	**643 (320 to 1 050)**	**10.4 (5.2 to 17.2)**	**865 (448 to 1 410)**	**10.7 (5.5 to 17.5)**	**34.7 (27.9 to 42.8)**	**2.8 (-2.2 to 8.8)**	**14 600 (7 440 to 23 500)**	**225.5 (115.3 to 364.8)**	**19 400 (10 300 to 31 100)**	**234.0 (124.0 to 376.0)**	**33.3 (25.8 to 42.0)**	**3.8 (-2.0 to 10.5)**
2	High fasting plasma glucose	312 (86.1 to 632)	5.2 (1.4 to 10.4)	419 (116 to 848)	5.3 (1.5 to 10.6)	34.2 (27.5 to 42.2)	2.0 (-3.2 to 8.0)	6 430 (1 740 to 13 100)	101.3 (27.4 to 207.0)	8 580 (2 360 to 17 600)	104.2 (28.7 to 212.9)	33.4 (26.1 to 42.2)	2.9 (-2.8 to 9.5)
2	High body-mass index	341 (188 to 536)	5.5 (3.0 to 8.6)	463 (261 to 718)	5.7 (3.2 to 8.8)	35.6 (27.8 to 45.2)	4.0 (-1.7 to 11.0)	8 360 (4 680 to 13 100)	127.9 (71.4 to 200.3)	11 200 (6 360 to 17 300)	133.9 (76.2 to 206.8)	33.7 (25.1 to 44.5)	4.8 (-1.8 to 12.9)

All numbers in this table represent total risk-attributable cancers included in this analysis. Results are for both sexes combined. The number on the left of each risk factor indicates its level in the GBD hierarchy; for more information on risk factor levels in the GBD hierarchy see Appendix table 9 (p152–153). An expanded version of this table is presented in Appendix table 33 (p248–253), which includes each risk-outcome pair included in this analysis. DALYs = disability-adjusted life-years; ASRs = age-standardised rates.

Appendix Table 31: Global numbers and age-standardised rates of risk-attributable total cancer deaths and DALYs, 2010 and 2019, and percentage change in global numbers and age-standardised rates of risk-attributable total cancer deaths and cancer DALYs, 2010-2019, males

GBD Risk Level	Risk factor	Males											
		Deaths						DALYs					
		Deaths in 2010, thousands (95% UI)	Age-standardised death rates, per 100,000, in 2010 (95% UI)	Deaths in 2019, thousands (95% UI)	Age-standardised death rates, per 100,000, in 2019 (95% UI)	Percent change in absolute deaths, 2010 – 2019 (95% UI)	Percent change in age-standardised death rates, 2010 – 2019 (95% UI)	DALYs in 2010, thousands (95% UI)	Age-standardised DALY rates, per 100,000, in 2010 (95% UI)	DALYs in 2019, thousands (95% UI)	Age-standardised DALY rates, per 100,000, in 2019 (95% UI)	Percent change in absolute DALYs, 2010 – 2019 (95% UI)	Percent change in age-standardised DALY rates, 2010 – 2019 (95% UI)
**0**	**All risk factors**	**2 420 (2 250 to 2 610)**	**84.9 (78.8 to 91.9)**	**2 880 (2 600 to 3 180)**	**77.6 (70.2 to 86.0)**	**18.9 (8.6 to 29.4)**	**-8.6 (-16.1 to -0.9)**	**58 600 (54 600 to 63 600)**	**1 892.5 (1 764.0 to 2 052.5)**	**67 500 (60 800 to 75 100)**	**1 711.6 (1 546.9 to 1 903.5)**	**15.2 (4.8 to 26.0)**	**-9.6 (-17.6 to -1.3)**
**1**	**Environmental/occupational risks**	**473 (399 to 544)**	**16.9 (14.3 to 19.6)**	**538 (450 to 629)**	**14.7 (12.3 to 17.1)**	**13.9 (3.6 to 25.5)**	**-12.9 (-20.4 to -4.5)**	**10 800 (9 210 to 12 500)**	**354.1 (301.6 to 408.3)**	**11 900 (9 920 to 14 000)**	**304.9 (255.3 to 357.9)**	**10.4 (-0.6 to 22.6)**	**-13.9 (-22.3 to -4.6)**
2	Air pollution	238 (181 to 293)	8.2 (6.2 to 10.1)	268 (197 to 344)	7.1 (5.2 to 9.1)	12.3 (-2.0 to 28.9)	-13.4 (-24.2 to -0.9)	5 760 (4 380 to 7 100)	185.2 (141.1 to 228.1)	6 250 (4 640 to 8 070)	157.9 (116.8 to 203.6)	8.5 (-5.6 to 25.1)	-14.8 (-25.7 to -2.0)
3	Particulate matter pollution	238 (181 to 293)	8.2 (6.2 to 10.1)	268 (197 to 344)	7.1 (5.2 to 9.1)	12.3 (-2.0 to 28.9)	-13.4 (-24.2 to -0.9)	5 760 (4 380 to 7 100)	185.2 (141.1 to 228.1)	6 250 (4 640 to 8 070)	157.9 (116.8 to 203.6)	8.5 (-5.6 to 25.1)	-14.8 (-25.7 to -2.0)
4	Ambient particulate matter pollution	174 (127 to 220)	6.0 (4.4 to 7.7)	216 (157 to 281)	5.8 (4.2 to 7.5)	24.6 (8.1 to 44.3)	-4.2 (-16.5 to 10.6)	4 140 (3 010 to 5 260)	133.8 (97.5 to 169.8)	5 000 (3 620 to 6 500)	126.5 (91.7 to 164.2)	20.6 (4.2 to 40.2)	-5.5 (-18.2 to 9.7)
4	Household air pollution from solid fuels	64.6 (38.3 to 97.8)	2.2 (1.3 to 3.3)	51.2 (27.7 to 82.2)	1.3 (0.7 to 2.1)	-20.7 (-33.5 to -6.0)	-38.9 (-48.7 to -27.6)	1 620 (967 to 2 440)	51.4 (30.7 to 77.5)	1 260 (687 to 2 010)	31.4 (17.1 to 50.2)	-22.3 (-34.5 to -7.9)	-39.0 (-48.5 to -27.7)
2	Other environmental risks	48.8 (9.46 to 94.3)	1.7 (0.3 to 3.3)	56.8 (11.3 to 110)	1.5 (0.3 to 2.9)	16.5 (6.6 to 28.9)	-10.5 (-17.8 to -1.2)	1 150 (225 to 2 230)	37.4 (7.3 to 72.4)	1 300 (258 to 2 540)	33.0 (6.5 to 64.4)	12.6 (2.9 to 25.4)	-11.9 (-19.4 to -2.0)
3	Residential radon	48.8 (9.46 to 94.3)	1.7 (0.3 to 3.3)	56.8 (11.3 to 110)	1.5 (0.3 to 2.9)	16.5 (6.6 to 28.9)	-10.5 (-17.8 to -1.2)	1 150 (225 to 2 230)	37.4 (7.3 to 72.4)	1 300 (258 to 2 540)	33.0 (6.5 to 64.4)	12.6 (2.9 to 25.4)	-11.9 (-19.4 to -2.0)
2	Occupational risks	236 (183 to 291)	8.8 (6.8 to 10.9)	267 (206 to 331)	7.5 (5.8 to 9.3)	13.3 (6.5 to 20.6)	-14.2 (-19.1 to -9.0)	4 990 (3 890 to 6 130)	169.0 (131.2 to 207.9)	5 520 (4 270 to 6 830)	144.4 (112.0 to 178.4)	10.6 (3.1 to 18.9)	-14.5 (-20.2 to -8.3)
3	Occupational carcinogens	236 (183 to 291)	8.8 (6.8 to 10.9)	267 (206 to 331)	7.5 (5.8 to 9.3)	13.3 (6.5 to 20.6)	-14.2 (-19.1 to -9.0)	4 990 (3 890 to 6 130)	169.0 (131.2 to 207.9)	5 520 (4 270 to 6 830)	144.4 (112.0 to 178.4)	10.6 (3.1 to 18.9)	-14.5 (-20.2 to -8.3)
4	Occupational exposure to asbestos	177 (126 to 230)	7.0 (4.9 to 9.0)	195 (139 to 255)	5.8 (4.1 to 7.5)	10.2 (3.7 to 16.9)	-17.0 (-21.5 to -12.3)	3 230 (2 270 to 4 240)	115.6 (81.2 to 150.7)	3 430 (2 400 to 4 510)	93.8 (65.9 to 123.0)	6.2 (-0.8 to 13.4)	-18.9 (-24.0 to -13.7)
4	Occupational exposure to arsenic	5.54 (0.807 to 10.2)	0.2 (0.0 to 0.3)	6.66 (1.05 to 12.1)	0.2 (0.0 to 0.3)	20.1 (6.0 to 43.4)	-6.0 (-17.0 to 12.9)	160 (25.1 to 292)	4.9 (0.7 to 8.9)	185 (32.3 to 336)	4.5 (0.8 to 8.2)	16.0 (2.1 to 38.6)	-8.0 (-18.9 to 10.5)
4	Occupational exposure to benzene	0.930 (0.280 to 1.52)	0.0 (0.0 to 0.0)	1.06 (0.313 to 1.73)	0.0 (0.0 to 0.0)	13.8 (4.6 to 23.0)	-1.2 (-9.1 to 7.1)	44.0 (13.2 to 72.1)	1.2 (0.4 to 2.0)	48.8 (14.2 to 80.2)	1.2 (0.4 to 2.0)	10.8 (1.7 to 20.3)	-1.5 (-9.5 to 7.0)
4	Occupational exposure to beryllium	0.164 (0.124 to 0.203)	0.0 (0.0 to 0.0)	0.203 (0.152 to 0.264)	0.0 (0.0 to 0.0)	23.8 (6.0 to 44.5)	-2.8 (-16.7 to 12.9)	4.85 (3.69 to 5.98)	0.1 (0.1 to 0.2)	5.81 (4.35 to 7.52)	0.1 (0.1 to 0.2)	19.9 (2.3 to 40.1)	-4.5 (-18.1 to 11.2)
4	Occupational exposure to cadmium	0.388 (0.304 to 0.474)	0.0 (0.0 to 0.0)	0.488 (0.371 to 0.614)	0.0 (0.0 to 0.0)	25.8 (7.3 to 47.2)	-1.3 (-15.6 to 15.1)	11.5 (8.99 to 14.1)	0.3 (0.3 to 0.4)	14.0 (10.7 to 17.6)	0.3 (0.3 to 0.4)	21.7 (3.9 to 42.5)	-3.1 (-17.0 to 12.7)
4	Occupational exposure to chromium	0.808 (0.682 to 0.930)	0.0 (0.0 to 0.0)	1.03 (0.841 to 1.26)	0.0 (0.0 to 0.0)	28.0 (9.8 to 47.8)	0.4 (-13.8 to 16.1)	23.8 (20.1 to 27.4)	0.7 (0.6 to 0.8)	29.5 (24.2 to 35.8)	0.7 (0.6 to 0.9)	23.9 (6.6 to 42.9)	-1.4 (-15.0 to 13.6)
4	Occupational exposure to diesel engine exhaust	11.3 (9.49 to 13.3)	0.4 (0.3 to 0.4)	14.7 (12.1 to 17.7)	0.4 (0.3 to 0.4)	30.4 (13.7 to 48.2)	2.3 (-10.7 to 16.1)	334 (280 to 392)	10.1 (8.5 to 11.9)	422 (347 to 510)	10.2 (8.4 to 12.3)	26.5 (10.5 to 44.0)	0.7 (-11.9 to 14.3)
4	Occupational exposure to formaldehyde	0.682 (0.518 to 0.873)	0.0 (0.0 to 0.0)	0.771 (0.578 to 1.00)	0.0 (0.0 to 0.0)	13.1 (0.5 to 27.4)	-2.2 (-12.7 to 9.6)	31.3 (23.6 to 40.6)	0.9 (0.7 to 1.1)	34.8 (26.0 to 45.1)	0.8 (0.6 to 1.1)	11.1 (-0.7 to 25.2)	-2.0 (-11.8 to 9.7)
4	Occupational exposure to nickel	5.55 (0.202 to 15.1)	0.2 (0.0 to 0.5)	6.64 (0.412 to 17.4)	0.2 (0.0 to 0.4)	19.6 (3.7 to 49.1)	-6.3 (-18.7 to 17.8)	161 (8.38 to 434)	4.9 (0.2 to 13.2)	186 (14.2 to 480)	4.5 (0.3 to 11.6)	15.6 (0.6 to 44.9)	-8.2 (-20.5 to 16.4)
4	Occupational exposure to polycyclic aromatic hydrocarbons	2.85 (2.24 to 3.46)	0.1 (0.1 to 0.1)	3.63 (2.80 to 4.59)	0.1 (0.1 to 0.1)	27.8 (9.7 to 48.2)	0.3 (-13.6 to 15.7)	84.1 (65.8 to 102)	2.5 (2.0 to 3.1)	104 (80.3 to 131)	2.5 (1.9 to 3.1)	23.7 (6.4 to 43.0)	-1.5 (-15.1 to 13.5)
4	Occupational exposure to silica	33.8 (14.9 to 53.1)	1.1 (0.5 to 1.7)	40.5 (18.2 to 64.1)	1.0 (0.4 to 1.6)	19.9 (7.4 to 34.6)	-6.1 (-15.9 to 5.4)	976 (430 to 1 540)	29.8 (13.1 to 46.8)	1 130 (513 to 1 790)	27.4 (12.4 to 43.4)	16.1 (3.7 to 30.2)	-7.8 (-17.7 to 3.5)

GBD Risk Level	Risk factor	**Males**											
		**Deaths**						**DALYs**					
		**Deaths in 2010, thousands (95% UI)**	**Age-standardised death rates, per 100,000, in 2010 (95% UI)**	**Deaths in 2019, thousands (95% UI)**	**Age-standardised death rates, per 100,000, in 2019 (95% UI)**	**Percent change in absolute deaths, 2010 – 2019 (95% UI)**	**Percent change in age-standardised death rates, 2010 – 2019 (95% UI)**	**DALYs in 2010, thousands (95% UI)**	**Age-standardised DALY rates, per 100,000, in 2010 (95% UI)**	**DALYs in 2019, thousands (95% UI)**	**Age-standardised DALY rates, per 100,000, in 2019 (95% UI)**	**Percent change in absolute DALYs, 2010 – 2019 (95% UI)**	**Percent change in age-standardised DALY rates, 2010 – 2019 (95% UI)**
4	Occupational exposure to sulfuric acid	3.05<br>(1.29 to 5.54)	0.1<br>(0.0 to 0.2)	3.59<br>(1.52 to 6.67)	0.1<br>(0.0 to 0.2)	17.7<br>(6.4 to 29.8)	-6.7<br>(-15.6 to 2.8)	98.0<br>(41.5 to 178)	2.9<br>(1.2 to 5.3)	113<br>(48.1 to 209)	2.7<br>(1.2 to 5.0)	15.2<br>(4.4 to 26.8)	-7.3<br>(-16.1 to 2.0)
4	Occupational exposure to trichloroethylene	0.0393<br>(0.00858 to 0.0730)	0.0<br>(0.0 to 0.0)	0.0554<br>(0.0117 to 0.102)	0.0<br>(0.0 to 0.0)	41.0<br>(27.4 to 55.6)	12.0<br>(1.2 to 23.5)	1.26<br>(0.278 to 2.34)	0.0<br>(0.0 to 0.1)	1.74<br>(0.367 to 3.23)	0.0<br>(0.0 to 0.1)	37.9<br>(24.2 to 52.2)	11.5<br>(0.4 to 23.1)
**1**	**Behavioural risks**	**2 180<br>(2 040 to 2 320)**	**76.2<br>(71.5 to 81.3)**	**2 550<br>(2 320 to 2 810)**	**68.7<br>(62.5 to 75.5)**	**17.4<br>(7.2 to 28.3)**	**-9.8<br>(-17.3 to -1.8)**	**52 600<br>(49 500 to 56 300)**	**1 700.7<br>(1 598.7 to 1 816.6)**	**59 900<br>(54 200 to 65 900)**	**1 517.0<br>(1 377.3 to 1 672.0)**	**13.7<br>(3.3 to 24.8)**	**-10.8<br>(-18.8 to -2.2)**
2	Tobacco	1 780<br>(1 680 to 1 890)	62.5<br>(58.5 to 66.2)	2 070<br>(1 870 to 2 270)	55.5<br>(50.3 to 61.0)	15.7<br>(5.1 to 27.0)	-11.2<br>(-19.1 to -2.9)	42 500<br>(39 900 to 45 100)	1 379.5<br>(1 296.4 to 1 458.8)	47 600<br>(42 900 to 52 700)	1 207.7<br>(1 087.4 to 1 334.9)	12.0<br>(1.2 to 23.6)	-12.5<br>(-20.6 to -3.7)
3	Smoking	1 760<br>(1 650 to 1 870)	61.6<br>(57.7 to 65.3)	2 030<br>(1 840 to 2 240)	54.6<br>(49.5 to 60.1)	15.5<br>(5.0 to 26.9)	-11.4<br>(-19.2 to -3.0)	41 800<br>(39 200 to 44 300)	1 356.4<br>(1 274.0 to 1 436.6)	46 700<br>(42 100 to 51 700)	1 184.6<br>(1 067.6 to 1 310.8)	11.8<br>(1.1 to 23.3)	-12.7<br>(-20.9 to -3.8)
3	Chewing tobacco	24.3<br>(17.5 to 32.0)	0.8<br>(0.6 to 1.0)	30.8<br>(21.2 to 41.2)	0.8<br>(0.5 to 1.1)	26.8<br>(6.6 to 49.1)	0.2<br>(-15.6 to 17.7)	721<br>(520 to 945)	21.9<br>(15.7 to 28.7)	885<br>(602 to 1 190)	21.7<br>(14.8 to 29.2)	22.8<br>(2.4 to 44.8)	-0.7<br>(-17.0 to 17.2)
3	Secondhand smoke	55.7<br>(32.2 to 82.4)	1.9<br>(1.1 to 2.9)	66.5<br>(38.4 to 101)	1.8<br>(1.0 to 2.7)	19.5<br>(5.3 to 36.5)	-8.0<br>(-18.8 to 4.7)	1 340<br>(777 to 1 980)	43.1<br>(25.0 to 63.9)	1 540<br>(898 to 2 320)	38.8<br>(22.6 to 58.8)	15.0<br>(1.2 to 31.7)	-9.9<br>(-20.6 to 3.0)
2	Alcohol use	319<br>(285 to 355)	10.7<br>(9.5 to 11.9)	394<br>(346 to 444)	10.3<br>(9.0 to 11.6)	23.5<br>(12.2 to 35.0)	-3.7<br>(-12.1 to 5.1)	8 750<br>(7 850 to 9 710)	272.0<br>(243.9 to 301.8)	10 500<br>(9 180 to 11 800)	259.9<br>(227.8 to 292.9)	19.6<br>(8.2 to 31.0)	-4.5<br>(-13.4 to 4.5)
2	Drug use	30.4<br>(25.2 to 36.7)	1.1<br>(0.9 to 1.3)	41.8<br>(34.2 to 51.0)	1.1<br>(0.9 to 1.4)	37.6<br>(25.1 to 51.6)	7.2<br>(-2.2 to 17.9)	736<br>(609 to 895)	23.6<br>(19.6 to 28.6)	966<br>(784 to 1 180)	24.5<br>(20.0 to 29.9)	31.3<br>(18.4 to 45.7)	3.7<br>(-6.2 to 14.9)
2	Dietary risks	303<br>(220 to 437)	10.8<br>(7.8 to 15.5)	352<br>(255 to 487)	9.7<br>(7.0 to 13.4)	16.3<br>(5.3 to 28.2)	-10.3<br>(-18.3 to -1.9)	7 430<br>(5 400 to 10 800)	240.0<br>(174.3 to 346.1)	8 350<br>(6 060 to 11 600)	213.2<br>(155.0 to 296.5)	12.4<br>(0.9 to 24.8)	-11.2<br>(-20.0 to -1.8)
3	Diet low in fruits	82.5<br>(41.2 to 133)	2.9<br>(1.4 to 4.6)	88.0<br>(43.1 to 142)	2.4<br>(1.2 to 3.8)	6.7<br>(-6.5 to 21.0)	-17.5<br>(-27.3 to -6.8)	2 040<br>(1 030 to 3 290)	65.1<br>(32.6 to 105.1)	2 100<br>(1 050 to 3 360)	52.8<br>(26.5 to 84.6)	2.8<br>(-9.8 to 16.9)	-18.9<br>(-28.6 to -7.9)
3	Diet low in vegetables	10.2<br>(1.46 to 20.5)	0.4<br>(0.0 to 0.7)	11.6<br>(1.71 to 23.2)	0.3<br>(0.0 to 0.6)	13.5<br>(3.7 to 24.9)	-13.2<br>(-20.6 to -4.5)	258<br>(38.0 to 518)	8.2<br>(1.2 to 16.5)	289<br>(43.9 to 576)	7.2<br>(1.1 to 14.4)	12.1<br>(2.3 to 22.8)	-11.9<br>(-19.8 to -3.2)
3	Diet low in whole grains	74.8<br>(28.6 to 98.1)	2.7<br>(1.1 to 3.6)	95.0<br>(36.4 to 125)	2.7<br>(1.0 to 3.5)	26.9<br>(18.0 to 36.4)	-3.0<br>(-9.4 to 3.6)	1 800<br>(688 to 2 360)	58.9<br>(22.6 to 77.2)	2 210<br>(849 to 2 920)	57.1<br>(21.9 to 75.4)	23.3<br>(14.1 to 33.3)	-3.0<br>(-10.0 to 4.5)
3	Diet low in milk	69.4<br>(45.0 to 93.5)	2.5<br>(1.6 to 3.4)	92.1<br>(59.3 to 126)	2.6<br>(1.6 to 3.5)	32.8<br>(21.6 to 44.9)	1.8<br>(-6.3 to 10.6)	1 710<br>(1 110 to 2 300)	55.5<br>(36.0 to 74.8)	2 200<br>(1 430 to 3 010)	56.5<br>(36.6 to 77.2)	28.7<br>(17.1 to 41.4)	1.7<br>(-7.2 to 11.3)
3	Diet high in red meat	22.8<br>(5.80 to 44.1)	0.8<br>(0.2 to 1.6)	30.4<br>(7.90 to 57.8)	0.8<br>(0.2 to 1.6)	33.3<br>(22.3 to 52.7)	1.9<br>(-6.0 to 16.6)	575<br>(153 to 1 080)	18.6<br>(4.9 to 35.4)	748<br>(205 to 1 390)	19.1<br>(5.2 to 35.4)	30.0<br>(17.5 to 49.8)	2.6<br>(-6.9 to 18.6)
3	Diet high in processed meat	15.0<br>(5.33 to 22.9)	0.6<br>(0.2 to 0.8)	17.7<br>(6.03 to 27.2)	0.5<br>(0.2 to 0.8)	18.6<br>(10.0 to 27.7)	-9.7<br>(-15.9 to -3.2)	351<br>(128 to 535)	11.7<br>(4.2 to 17.8)	405<br>(139 to 623)	10.5<br>(3.6 to 16.1)	15.6<br>(6.0 to 25.5)	-9.7<br>(-16.9 to -2.2)
3	Diet low in fibre	9.34<br>(3.78 to 17.9)	0.3<br>(0.1 to 0.7)	10.8<br>(4.25 to 20.7)	0.3<br>(0.1 to 0.6)	15.1<br>(6.8 to 23.8)	-11.6<br>(-17.6 to -5.4)	228<br>(94.5 to 430)	7.4<br>(3.1 to 14.0)	251<br>(99.6 to 476)	6.5<br>(2.6 to 12.4)	10.2<br>(1.1 to 19.7)	-12.4<br>(-19.2 to -5.1)
3	Diet low in calcium	64.6<br>(47.2 to 86.9)	2.3<br>(1.7 to 3.1)	80.0<br>(56.7 to 109)	2.2<br>(1.6 to 3.0)	23.8<br>(12.5 to 35.1)	-4.7<br>(-12.9 to 3.3)	1 590<br>(1 170 to 2 100)	51.5<br>(38.0 to 68.6)	1 900<br>(1 360 to 2 580)	48.8<br>(34.9 to 66.1)	19.8<br>(8.1 to 31.4)	-5.1<br>(-14.2 to 3.9)
3	Diet high in sodium	48.7<br>(1.28 to 190)	1.7<br>(0.0 to 6.6)	49.4<br>(1.30 to 193)	1.3<br>(0.0 to 5.2)	1.5<br>(-11.7 to 16.7)	-21.1<br>(-31.0 to -9.7)	1 220<br>(31.4 to 4 720)	38.8<br>(1.0 to 151.0)	1 180<br>(30.5 to 4 550)	29.9<br>(0.8 to 115.3)	-2.9<br>(-16.2 to 12.1)	-22.9<br>(-33.1 to -11.2)
2	Unsafe sex	NA	NA	NA	NA	NA	NA	NA	NA	NA	NA	NA	NA
2	Low physical activity	20.0<br>(4.72 to 39.8)	0.8<br>(0.2 to 1.6)	26.6<br>(6.38 to 52.4)	0.8<br>(0.2 to 1.6)	32.9<br>(23.6 to 43.8)	-1.4<br>(-7.8 to 6.2)	367<br>(88.3 to 740)	13.4<br>(3.2 to 27.0)	479<br>(112 to 952)	13.3<br>(3.1 to 26.4)	30.6<br>(20.5 to 41.3)	-1.0<br>(-8.2 to 6.8)
**1**	**Metabolic risks**	**337<br>(157 to 579)**	**12.1<br>(5.6 to 21.0)**	**453<br>(221 to 760)**	**12.4<br>(6.0 to 20.9)**	**34.6<br>(25.4 to 45.7)**	**2.4<br>(-4.0 to 10.7)**	**7 880<br>(3 830 to 13 300)**	**258.3<br>(123.9 to 438.7)**	**10 400<br>(5 170 to 17 400)**	**266.8<br>(132.4 to 443.9)**	**32.6<br>(22.6 to 44.6)**	**3.3<br>(-4.3 to 12.7)**
2	High fasting plasma glucose	171<br>(42.0 to 362)	6.4<br>(1.6 to 13.5)	225<br>(55.5 to 482)	6.3<br>(1.6 to 13.4)	31.6<br>(22.5 to 42.2)	-0.9<br>(-7.5 to 6.4)	3 540<br>(855 to 7 540)	121.1<br>(29.6 to 257.5)	4 600<br>(1 120 to 9 900)	120.4<br>(29.5 to 257.7)	30.0<br>(20.3 to 41.5)	-0.5<br>(-7.8 to 7.8)
2	High body-mass index	171<br>(82.6 to 289)	5.9<br>(2.9 to 10.0)	236<br>(120 to 389)	6.3<br>(3.2 to 10.4)	38.1<br>(26.6 to 51.2)	6.4<br>(-2.0 to 16.1)	4 450<br>(2 170 to 7 510)	141.1<br>(68.6 to 237.8)	6 010<br>(3 090 to 9 900)	150.7<br>(77.1 to 247.5)	35.0<br>(22.7 to 49.0)	6.8<br>(-2.5 to 17.6)

All numbers in this table represent total risk-attributable cancers included in this analysis. The number on the left of each risk factor indicates its level in the GBD hierarchy; for more information on risk factor levels in the GBD hierarchy see Appendix table 9 (p152–153). DALYs = disability-adjusted life-years; ASRs = age-standardised rates; NA = not applicable due to sex restriction.

Appendix Table 32: Global numbers and age-standardised rates of risk-attributable total cancer deaths and DALYs, 2010 and 2019, and percentage change in global numbers and age-standardised rates of risk-attributable total cancer deaths and cancer DALYs, 2010-2019, females

GBD Risk Level	Risk factor	Females											
		Deaths						DALYs					
		Deaths in 2010, thousands (95% UI)	Age-standardised death rates, per 100,000, in 2010 (95% UI)	Deaths in 2019, thousands (95% UI)	Age-standardised death rates, per 100,000, in 2019 (95% UI)	Percent change in absolute deaths, 2010 – 2019 (95% UI)	Percent change in age-standardised death rates, 2010 – 2019 (95% UI)	DALYs in 2010, thousands (95% UI)	Age-standardised DALY rates, per 100,000, in 2010 (95% UI)	DALYs in 2019, thousands (95% UI)	Age-standardised DALY rates, per 100,000, in 2019 (95% UI)	Percent change in absolute DALYs, 2010 – 2019 (95% UI)	Percent change in age-standardised DALY rates, 2010 – 2019 (95% UI)
**0**	**All risk factors**	**1 280<br>(1 130 to 1 470)**	**37.7<br>(33.3 to 43.4)**	**1 580<br>(1 360 to 1 840)**	**36.1<br>(31.1 to 42.0)**	**23.1<br>(15.3 to 30.8)**	**-4.4<br>(-10.4 to 1.5)**	**31 400<br>(28 000 to 35 600)**	**908.7<br>(812.0 to 1 033.1)**	**37 600<br>(32 800 to 43 100)**	**866.9<br>(756.6 to 994.9)**	**19.8<br>(11.7 to 28.2)**	**-4.6<br>(-11.0 to 2.2)**
**1**	**Environmental/ occupational risks**	**159<br>(131 to 188)**	**4.7<br>(3.9 to 5.5)**	**198<br>(159 to 239)**	**4.5<br>(3.6 to 5.5)**	**24.8<br>(12.6 to 38.5)**	**-3.3<br>(-12.8 to 7.2)**	**3 620<br>(3 010 to 4 250)**	**105.4<br>(87.7 to 123.9)**	**4 380<br>(3 550 to 5 270)**	**100.6<br>(81.6 to 121.1)**	**21.0<br>(8.2 to 35.1)**	**-4.6<br>(-14.7 to 6.6)**
2	Air pollution	97.3<br>(74.8 to 120)	2.9<br>(2.2 to 3.5)	120<br>(88.8 to 151)	2.7<br>(2.0 to 3.4)	23.1<br>(7.5 to 39.6)	-4.4<br>(-16.5 to 8.5)	2 270<br>(1 740 to 2 800)	66.0<br>(50.7 to 81.4)	2 700<br>(2 000 to 3 390)	62.0<br>(45.9 to 77.9)	18.8<br>(3.3 to 35.0)	-6.2<br>(-18.2 to 6.6)
3	Particulate matter pollution	97.3<br>(74.8 to 120)	2.9<br>(2.2 to 3.5)	120<br>(88.8 to 151)	2.7<br>(2.0 to 3.4)	23.1<br>(7.5 to 39.6)	-4.4<br>(-16.5 to 8.5)	2 270<br>(1 740 to 2 800)	66.0<br>(50.7 to 81.4)	2 700<br>(2 000 to 3 390)	62.0<br>(45.9 to 77.9)	18.8<br>(3.3 to 35.0)	-6.2<br>(-18.2 to 6.6)
4	Ambient particulate matter pollution	65.9<br>(47.1 to 84.1)	1.9<br>(1.4 to 2.5)	91.3<br>(65.5 to 119)	2.1<br>(1.5 to 2.7)	38.5<br>(21.2 to 58.5)	7.3<br>(-6.0 to 22.7)	1 500<br>(1 080 to 1 910)	43.8<br>(31.3 to 55.7)	2 020<br>(1 460 to 2 630)	46.3<br>(33.5 to 60.2)	34.2<br>(16.5 to 54.9)	5.8<br>(-8.2 to 22.1)
4	Household air pollution from solid fuels	31.4<br>(19.6 to 45.6)	0.9<br>(0.6 to 1.3)	28.5<br>(16.0 to 44.5)	0.7<br>(0.4 to 1.0)	-9.2<br>(-24.2 to 7.1)	-29.1<br>(-40.8 to -16.5)	767<br>(480 to 1 110)	22.2<br>(13.9 to 32.2)	679<br>(384 to 1 050)	15.6<br>(8.8 to 24.2)	-11.4<br>(-25.9 to 4.6)	-29.7<br>(-41.1 to -17.0)
2	Other environmental risks	21.0<br>(4.18 to 40.7)	0.6<br>(0.1 to 1.2)	26.9<br>(5.22 to 52.2)	0.6<br>(0.1 to 1.2)	28.1<br>(18.0 to 39.5)	-1.0<br>(-8.7 to 7.8)	472<br>(94.4 to 918)	13.8<br>(2.8 to 26.8)	586<br>(115 to 1 140)	13.4<br>(2.6 to 26.0)	24.1<br>(13.5 to 35.9)	-2.4<br>(-10.8 to 6.9)
3	Residential radon	21.0<br>(4.18 to 40.7)	0.6<br>(0.1 to 1.2)	26.9<br>(5.22 to 52.2)	0.6<br>(0.1 to 1.2)	28.1<br>(18.0 to 39.5)	-1.0<br>(-8.7 to 7.8)	472<br>(94.4 to 918)	13.8<br>(2.8 to 26.8)	586<br>(115 to 1 140)	13.4<br>(2.6 to 26.0)	24.1<br>(13.5 to 35.9)	-2.4<br>(-10.8 to 6.9)
2	Occupational risks	52.9<br>(39.7 to 65.9)	1.6<br>(1.2 to 2.0)	66.5<br>(48.7 to 85.0)	1.5<br>(1.1 to 1.9)	25.7<br>(15.1 to 37.1)	-3.0<br>(-11.0 to 5.8)	1 170<br>(884 to 1 460)	34.1<br>(25.7 to 42.6)	1 440<br>(1 070 to 1 830)	33.1<br>(24.5 to 42.0)	23.2<br>(12.0 to 35.5)	-3.0<br>(-11.7 to 6.6)
3	Occupational carcinogens	52.9<br>(39.7 to 65.9)	1.6<br>(1.2 to 2.0)	66.5<br>(48.7 to 85.0)	1.5<br>(1.1 to 1.9)	25.7<br>(15.1 to 37.1)	-3.0<br>(-11.0 to 5.8)	1 170<br>(884 to 1 460)	34.1<br>(25.7 to 42.6)	1 440<br>(1 070 to 1 830)	33.1<br>(24.5 to 42.0)	23.2<br>(12.0 to 35.5)	-3.0<br>(-11.7 to 6.6)
4	Occupational exposure to asbestos	33.1<br>(21.9 to 42.7)	1.0<br>(0.7 to 1.3)	40.3<br>(25.7 to 52.6)	0.9<br>(0.6 to 1.2)	21.7<br>(10.7 to 32.3)	-7.2<br>(-15.3 to 0.8)	579<br>(393 to 736)	17.1<br>(11.6 to 21.7)	691<br>(455 to 892)	15.8<br>(10.4 to 20.4)	19.4<br>(7.8 to 30.9)	-7.6<br>(-16.4 to 1.4)
4	Occupational exposure to arsenic	2.35<br>(0.359 to 4.40)	0.1<br>(0.0 to 0.1)	3.10<br>(0.494 to 5.69)	0.1<br>(0.0 to 0.1)	32.0<br>(16.0 to 54.1)	3.3<br>(-9.2 to 20.8)	67.1<br>(10.9 to 125)	1.9<br>(0.3 to 3.6)	85.4<br>(14.6 to 155)	2.0<br>(0.3 to 3.6)	27.4<br>(11.4 to 49.6)	0.8<br>(-11.8 to 18.5)
4	Occupational exposure to benzene	0.705<br>(0.229 to 1.16)	0.0<br>(0.0 to 0.0)	0.808<br>(0.264 to 1.34)	0.0<br>(0.0 to 0.0)	14.7<br>(4.4 to 26.9)	-0.3<br>(-8.9 to 10.5)	33.4<br>(10.8 to 54.6)	0.9<br>(0.3 to 1.5)	37.0<br>(12.2 to 61.0)	0.9<br>(0.3 to 1.5)	11.0<br>(0.8 to 23.6)	-1.2<br>(-10.3 to 10.0)
4	Occupational exposure to beryllium	0.0698<br>(0.0548 to 0.0849)	0.0<br>(0.0 to 0.0)	0.0978<br>(0.0746 to 0.123)	0.0<br>(0.0 to 0.0)	40.2<br>(19.0 to 64.4)	10.0<br>(-6.4 to 28.8)	2.05<br>(1.63 to 2.51)	0.1<br>(0.0 to 0.1)	2.77<br>(2.12 to 3.47)	0.1<br>(0.0 to 0.1)	35.0<br>(14.6 to 58.3)	7.3<br>(-8.8 to 25.7)
4	Occupational exposure to cadmium	0.161<br>(0.130 to 0.199)	0.0<br>(0.0 to 0.0)	0.224<br>(0.172 to 0.284)	0.0<br>(0.0 to 0.0)	39.2<br>(17.2 to 65.1)	9.2<br>(-7.2 to 28.9)	4.71<br>(3.77 to 5.83)	0.1<br>(0.1 to 0.2)	6.31<br>(4.86 to 7.98)	0.1<br>(0.1 to 0.2)	34.0<br>(12.7 to 58.4)	6.5<br>(-9.5 to 25.4)
4	Occupational exposure to chromium	0.330<br>(0.279 to 0.384)	0.0<br>(0.0 to 0.0)	0.466<br>(0.378 to 0.568)	0.0<br>(0.0 to 0.0)	41.2<br>(20.8 to 65.5)	10.8<br>(-5.3 to 30.0)	9.67<br>(8.09 to 11.2)	0.3<br>(0.2 to 0.3)	13.2<br>(10.7 to 16.0)	0.3<br>(0.2 to 0.4)	36.2<br>(16.3 to 59.6)	8.3<br>(-7.5 to 27.2)
4	Occupational exposure to diesel engine exhaust	3.46<br>(2.90 to 4.05)	0.1<br>(0.1 to 0.1)	5.00<br>(4.05 to 6.08)	0.1<br>(0.1 to 0.1)	44.5<br>(26.0 to 65.9)	13.3<br>(-1.2 to 29.8)	101<br>(85.1 to 119)	2.9<br>(2.5 to 3.5)	141<br>(114 to 171)	3.2<br>(2.6 to 3.9)	39.5<br>(21.1 to 60.3)	10.8<br>(-3.7 to 27.2)
4	Occupational exposure to formaldehyde	0.326<br>(0.253 to 0.408)	0.0<br>(0.0 to 0.0)	0.347<br>(0.267 to 0.435)	0.0<br>(0.0 to 0.0)	6.6<br>(-7.8 to 22.7)	-7.3<br>(-19.5 to 6.3)	15.6<br>(11.9 to 19.6)	0.4<br>(0.3 to 0.5)	16.0<br>(12.3 to 20.2)	0.4<br>(0.3 to 0.5)	3.0<br>(-10.3 to 17.5)	-8.6<br>(-20.5 to 4.0)
4	Occupational exposure to nickel	2.04<br>(0.0761 to 5.47)	0.1<br>(0.0 to 0.2)	2.69<br>(0.162 to 7.07)	0.1<br>(0.0 to 0.2)	31.6<br>(13.3 to 64.8)	3.1<br>(-11.3 to 30.2)	58.8<br>(2.86 to 155)	1.7<br>(0.1 to 4.5)	74.6<br>(5.61 to 193)	1.7<br>(0.1 to 4.4)	26.9<br>(8.9 to 58.0)	0.5<br>(-13.7 to 26.2)
4	Occupational exposure to polycyclic aromatic hydrocarbons	1.16<br>(0.943 to 1.42)	0.0<br>(0.0 to 0.0)	1.64<br>(1.30 to 2.05)	0.0<br>(0.0 to 0.0)	41.6<br>(21.3 to 65.9)	11.1<br>(-4.6 to 30.3)	33.9<br>(27.3 to 41.2)	1.0<br>(0.8 to 1.2)	46.3<br>(36.6 to 57.4)	1.1<br>(0.8 to 1.3)	36.6<br>(17.1 to 60.5)	8.6<br>(-6.9 to 27.8)
4	Occupational exposure to silica	9.64<br>(4.21 to 15.3)	0.3<br>(0.1 to 0.4)	12.5<br>(5.19 to 19.9)	0.3<br>(0.1 to 0.5)	29.3<br>(14.0 to 47.8)	1.2<br>(-10.9 to 15.8)	276<br>(121 to 438)	8.0<br>(3.5 to 12.7)	344<br>(142 to 547)	7.9<br>(3.3 to 12.6)	24.6<br>(9.4 to 42.6)	-1.3<br>(-13.5 to 13.0)
4	Occupational exposure to sulfuric acid	0.361<br>(0.151 to 0.653)	0.0<br>(0.0 to 0.0)	0.438<br>(0.188 to 0.799)	0.0<br>(0.0 to 0.0)	21.3<br>(8.8 to 34.3)	-4.4<br>(-14.2 to 5.9)	11.4<br>(4.71 to 20.6)	0.3<br>(0.1 to 0.6)	13.5<br>(5.81 to 24.7)	0.3<br>(0.1 to 0.6)	18.6<br>(7.1 to 31.1)	-5.1<br>(-14.4 to 5.0)
4	Occupational exposure to trichloroethylene	0.0166<br>(0.00363 to 0.0314)	0.0<br>(0.0 to 0.0)	0.0231<br>(0.00506 to 0.0433)	0.0<br>(0.0 to 0.0)	39.2<br>(28.0 to 52.2)	9.6<br>(0.7 to 19.8)	0.506<br>(0.111 to 0.962)	0.0<br>(0.0 to 0.0)	0.690<br>(0.150 to 1.29)	0.0<br>(0.0 to 0.0)	36.4<br>(25.3 to 49.3)	9.1<br>(0.1 to 19.3)
**1**	**Behavioural risks**	**961<br>(884 to 1 050)**	**28.3<br>(26.0 to 31.0)**	**1 150<br>(1 030 to 1 260)**	**26.3<br>(23.8 to 29.0)**	**19.2<br>(12.1 to 26.6)**	**-7.1<br>(-12.7 to -1.4)**	**24 100<br>(22 500 to 26 200)**	**696.2<br>(649.7 to 756.0)**	**27 900<br>(25 400 to 30 700)**	**646.3<br>(587.2 to 710.5)**	**15.9<br>(8.4 to 23.9)**	**-7.2<br>(-13.1 to -0.8)**

GBD Risk Level	Risk factor	Females											
		Deaths						DALYs					
		**Deaths in 2010, thousands (95% UI)**	**Age-standardised death rates, per 100,000, in 2010 (95% UI)**	**Deaths in 2019, thousands (95% UI)**	**Age-standardised death rates, per 100,000, in 2019 (95% UI)**	**Percent change in absolute deaths, 2010 – 2019 (95% UI)**	**Percent change in age-standardised death rates, 2010 – 2019 (95% UI)**	**DALYs in 2010, thousands (95% UI)**	**Age-standardised DALY rates, per 100,000, in 2010 (95% UI)**	**DALYs in 2019, thousands (95% UI)**	**Age-standardised DALY rates, per 100,000, in 2019 (95% UI)**	**Percent change in absolute DALYs, 2010 – 2019 (95% UI)**	**Percent change in age-standardised DALY rates, 2010 – 2019 (95% UI)**
2	Tobacco	452 (412 to 486)	13.4 (12.2 to 14.4)	534 (476 to 585)	12.2 (10.9 to 13.4)	18.2 (11.4 to 25.5)	-8.8 (-14.0 to -3.2)	10 200 (9 370 to 11 000)	297.1 (273.8 to 320.9)	11 700 (10 600 to 12 900)	267.8 (242.1 to 294.9)	14.9 (8.3 to 22.0)	-9.9 (-15.0 to -4.3)
3	Smoking	399 (365 to 430)	11.8 (10.8 to 12.8)	462 (414 to 504)	10.5 (9.4 to 11.5)	15.8 (9.8 to 22.4)	-10.9 (-15.5 to -5.9)	8 680 (8 030 to 9 400)	254.2 (235.4 to 275.1)	9 750 (8 840 to 10 700)	222.9 (202.1 to 243.5)	12.4 (6.7 to 18.7)	-12.3 (-16.6 to -7.4)
3	Chewing tobacco	16.9 (13.4 to 20.7)	0.5 (0.4 to 0.6)	24.8 (18.6 to 31.9)	0.6 (0.4 to 0.7)	46.4 (24.0 to 70.6)	14.2 (-3.3 to 32.8)	438 (345 to 539)	12.7 (10.0 to 15.6)	619 (467 to 799)	14.3 (10.8 to 18.4)	41.2 (18.3 to 65.6)	12.3 (-5.8 to 31.8)
3	Secondhand smoke	49.7 (32.0 to 69.5)	1.5 (0.9 to 2.0)	64.0 (41.1 to 92.4)	1.5 (0.9 to 2.1)	28.7 (14.6 to 44.2)	1.2 (-9.8 to 13.4)	1 360 (889 to 1 910)	39.2 (25.6 to 54.9)	1 680 (1 100 to 2 420)	39.0 (25.5 to 55.9)	23.4 (10.0 to 38.6)	-0.7 (-11.5 to 11.8)
2	Alcohol use	87.1 (76.3 to 98.2)	2.6 (2.2 to 2.9)	101 (87.6 to 115)	2.3 (2.0 to 2.6)	15.7 (9.8 to 22.2)	-10.3 (-14.8 to -5.2)	2 230 (1 980 to 2 500)	64.6 (57.4 to 72.4)	2 520 (2 220 to 2 850)	58.3 (51.4 to 65.9)	13.0 (7.0 to 19.7)	-9.9 (-14.6 to -4.5)
2	Drug use	21.7 (15.9 to 28.3)	0.6 (0.5 to 0.8)	29.6 (21.8 to 38.8)	0.7 (0.5 to 0.9)	36.4 (22.8 to 51.5)	5.7 (-4.9 to 17.4)	493 (365 to 633)	14.4 (10.6 to 18.5)	645 (491 to 835)	14.8 (11.2 to 19.1)	30.9 (16.6 to 46.8)	2.6 (-8.6 to 15.1)
2	Dietary risks	214 (164 to 288)	6.3 (4.9 to 8.5)	253 (191 to 334)	5.8 (4.4 to 7.6)	18.6 (10.3 to 27.5)	-8.5 (-14.9 to -1.9)	4 850 (3 730 to 6 580)	141.1 (108.4 to 191.3)	5 600 (4 280 to 7 320)	129.2 (98.7 to 168.7)	15.5 (6.8 to 25.0)	-8.4 (-15.4 to -1.0)
3	Diet low in fruits	36.3 (19.1 to 55.4)	1.1 (0.6 to 1.6)	40.4 (21.2 to 60.0)	0.9 (0.5 to 1.4)	11.3 (-2.3 to 25.5)	-13.8 (-24.3 to -2.8)	830 (440 to 1 260)	24.1 (12.8 to 36.8)	902 (484 to 1 330)	20.7 (11.1 to 30.5)	8.7 (-3.8 to 22.4)	-14.1 (-24.1 to -3.1)
3	Diet low in vegetables	4.86 (0.759 to 9.64)	0.1 (0.0 to 0.3)	5.60 (0.868 to 10.9)	0.1 (0.0 to 0.2)	15.2 (5.2 to 26.7)	-10.7 (-18.8 to -1.8)	113 (18.5 to 223)	3.3 (0.5 to 6.5)	131 (20.9 to 258)	3.0 (0.5 to 5.9)	15.8 (5.1 to 27.5)	-8.2 (-16.7 to 1.0)
3	Diet low in whole grains	61.8 (23.7 to 81.0)	1.8 (0.7 to 2.4)	76.5 (29.7 to 100)	1.7 (0.7 to 2.3)	23.8 (16.4 to 31.0)	-5.2 (-10.7 to 0.4)	1 320 (506 to 1 730)	38.4 (14.7 to 50.4)	1 590 (618 to 2 120)	36.6 (14.2 to 48.8)	21.0 (12.9 to 29.2)	-4.6 (-10.8 to 1.8)
3	Diet low in milk	56.4 (35.8 to 77.1)	1.7 (1.1 to 2.3)	74.4 (46.9 to 100)	1.7 (1.1 to 2.3)	31.9 (22.2 to 42.1)	1.3 (-6.1 to 9.3)	1 240 (808 to 1 690)	36.2 (23.5 to 49.3)	1 600 (1 010 to 2 130)	36.8 (23.3 to 49.0)	28.6 (18.6 to 39.5)	1.7 (-6.3 to 10.4)
3	Diet high in red meat	36.3 (19.8 to 55.8)	1.1 (0.6 to 1.7)	44.9 (25.1 to 69.2)	1.0 (0.6 to 1.6)	23.5 (15.2 to 31.8)	-4.1 (-10.3 to 2.5)	948 (525 to 1 390)	27.4 (15.2 to 40.2)	1 140 (647 to 1 670)	26.3 (15.0 to 38.7)	20.0 (11.4 to 28.6)	-3.8 (-10.7 to 3.4)
3	Diet high in processed meat	14.1 (5.11 to 21.8)	0.4 (0.2 to 0.7)	16.2 (5.61 to 24.9)	0.4 (0.1 to 0.6)	14.8 (5.2 to 22.4)	-12.5 (-19.6 to -6.6)	292 (109 to 450)	8.5 (3.2 to 13.2)	330 (117 to 509)	7.6 (2.7 to 11.7)	12.8 (2.3 to 21.4)	-11.4 (-19.6 to -4.6)
3	Diet low in fibre	8.55 (3.49 to 16.5)	0.3 (0.1 to 0.5)	9.74 (3.95 to 18.9)	0.2 (0.1 to 0.4)	14.0 (7.1 to 21.3)	-12.9 (-18.0 to -7.3)	180 (73.6 to 348)	5.2 (2.1 to 10.1)	197 (80.2 to 381)	4.6 (1.8 to 8.8)	9.3 (1.5 to 17.5)	-13.2 (-19.4 to -6.9)
3	Diet low in calcium	46.8 (32.9 to 64.5)	1.4 (1.0 to 1.9)	57.9 (40.2 to 80.5)	1.3 (0.9 to 1.8)	23.9 (14.3 to 33.5)	-4.6 (-12.0 to 2.8)	1 040 (747 to 1 410)	30.1 (21.7 to 41.0)	1 240 (887 to 1 700)	28.7 (20.5 to 39.2)	19.9 (10.1 to 30.2)	-4.9 (-12.8 to 3.2)
3	Diet high in sodium	23.7 (0.754 to 97.8)	0.7 (0.0 to 2.9)	24.7 (0.782 to 102)	0.6 (0.0 to 2.3)	4.3 (-8.5 to 16.3)	-19.1 (-28.8 to -9.7)	553 (17.3 to 2 260)	16.0 (0.5 to 65.4)	555 (17.5 to 2 270)	12.8 (0.4 to 52.5)	0.4 (-13.1 to 13.1)	-20.0 (-30.7 to -9.9)
2	Unsafe sex	238 (211 to 269)	6.8 (6.1 to 7.7)	280 (239 to 314)	6.5 (5.5 to 7.3)	17.9 (8.5 to 28.6)	-4.9 (-12.6 to 3.6)	7 820 (6 850 to 8 680)	221.4 (194.6 to 246.1)	8 960 (7 550 to 9 980)	210.6 (177.7 to 234.9)	14.6 (5.0 to 25.0)	-4.8 (-12.8 to 3.7)
2	Low physical activity	32.9 (15.0 to 54.8)	1.0 (0.5 to 1.7)	40.5 (18.4 to 68.4)	0.9 (0.4 to 1.6)	23.1 (16.2 to 30.1)	-7.0 (-12.2 to -1.7)	599 (287 to 990)	17.7 (8.5 to 29.1)	724 (338 to 1 210)	16.6 (7.8 to 27.7)	20.9 (13.4 to 28.3)	-6.1 (-11.7 to -0.1)
1	Metabolic risks	306 (159 to 495)	9.1 (4.7 to 14.7)	412 (216 to 667)	9.4 (4.9 to 15.2)	34.7 (27.5 to 43.4)	3.1 (-2.3 to 9.8)	6 680 (3 560 to 10 700)	196.5 (104.8 to 313.1)	8 970 (4 860 to 14 200)	204.7 (110.8 to 324.0)	34.2 (26.2 to 44.0)	4.2 (-2.0 to 11.6)
2	High fasting plasma glucose	142 (38.7 to 291)	4.2 (1.2 to 8.7)	195 (53.4 to 410)	4.4 (1.2 to 9.3)	37.4 (29.7 to 46.0)	5.1 (-0.8 to 11.5)	2 890 (769 to 5 950)	85.1 (22.6 to 175.0)	3 980 (1 090 to 8 400)	91.0 (24.8 to 192.1)	37.6 (29.1 to 47.2)	6.9 (0.2 to 14.3)
2	High body-mass index	170 (99.0 to 261)	5.1 (3.0 to 7.8)	226 (136 to 340)	5.2 (3.1 to 7.7)	33.0 (25.5 to 42.4)	1.7 (-3.9 to 8.9)	3 910 (2 290 to 5 970)	114.9 (67.4 to 175.6)	5 160 (3 130 to 7 690)	117.8 (71.3 to 175.0)	32.2 (23.9 to 42.7)	2.4 (-4.0 to 10.6)

All numbers in this table represent total risk-attributable cancers included in this analysis. The number on the left of each risk factor indicates its level in the GBD hierarchy; for more information on risk factor levels in the GBD hierarchy see Appendix table 9 (p152–153). DALYs = disability-adjusted life-years; ASRs = age-standardised rates.

Appendix Table 33: Global numbers and age-standardised rates of attributable cancer deaths and DALYs, 2010 and 2019, and percentage change in global numbers and age-standardised rates of attributable cancer deaths and cancer DALYs, 2010-2019, for all risk-cancer pairs measured, both sexes combined

GBD Risk Level	Risk factor	Cancer	Both sexes combined											
			Deaths						DALYs					
			Deaths in 2010, thousands (95% UI)	Age-standardised death rates, per 100,000, in 2010 (95% UI)	Deaths in 2019, thousands (95% UI)	Age-standardised death rates, per 100,000, in 2019 (95% UI)	Percent change in absolute deaths, 2010 – 2019 (95% UI)	Percent change in age-standardised death rates, 2010 – 2019 (95% UI)	DALYs in 2010, thousands (95% UI)	Age-standardised DALY rates, per 100,000, in 2010 (95% UI)	DALYs in 2019, thousands (95% UI)	Age-standardised DALY rates, per 100,000, in 2019 (95% UI)	Percent change in absolute DALYs, 2010 – 2019 (95% UI)	Percent change in age-standardised DALY rates, 2010 – 2019 (95% UI)
**0**	**All risk factors**	**Total cancers**	**3 700 (3 410 to 4 050)**	**59.0 (54.3 to 64.7)**	**4 450 (4 010 to 4 940)**	**54.9 (49.3 to 61.0)**	**20.4 (12.6 to 28.4)**	**-6.9 (-12.8 to -0.9)**	**89 900 (83 300 to 98 100)**	**1 369.2 (1 267.3 to 1 495.4)**	**105 000 (95 000 to 116 000)**	**1 262.7 (1 142.8 to 1 398.7)**	**16.8 (8.8 to 25.0)**	**-7.8 (-14.0 to -1.4)**
**1**	**Environmental/ occupational risks**	**Total cancers**	**631 (538 to 726)**	**10.1 (8.6 to 11.6)**	**737 (619 to 859)**	**9.1 (7.7 to 10.6)**	**16.7 (7.9 to 26.2)**	**-10.0 (-16.7 to -2.8)**	**14 400 (12 300 to 16 600)**	**221.2 (189.2 to 254.9)**	**16 300 (13 700 to 19 100)**	**196.1 (165.5 to 230.3)**	**13.1 (3.9 to 23.1)**	**-11.4 (-18.5 to -3.5)**
2	Air pollution	Total cancers	336 (256 to 412)	5.3 (4.0 to 6.5)	387 (288 to 490)	4.7 (3.5 to 6.0)	15.5 (3.4 to 28.3)	-10.5 (-19.8 to -0.8)	8 030 (6 110 to 9 850)	122.4 (93.2 to 150.2)	8 950 (6 680 to 11 300)	107.4 (80.1 to 136.0)	11.5 (-0.5 to 24.4)	-12.3 (-21.7 to -2.3)
3	Particulate matter pollution	Total cancers	336 (256 to 412)	5.3 (4.0 to 6.5)	387 (288 to 490)	4.7 (3.5 to 6.0)	15.5 (3.4 to 28.3)	-10.5 (-19.8 to -0.8)	8 030 (6 110 to 9 850)	122.4 (93.2 to 150.2)	8 950 (6 680 to 11 300)	107.4 (80.1 to 136.0)	11.5 (-0.5 to 24.4)	-12.3 (-21.7 to -2.3)
4	Ambient particulate matter pollution	Total cancers	240 (175 to 303)	3.8 (2.8 to 4.8)	308 (227 to 396)	3.8 (2.8 to 4.9)	28.4 (15.6 to 44.3)	-0.6 (-10.4 to 11.4)	5 650 (4 130 to 7 130)	86.3 (63.0 to 109.0)	7 020 (5 180 to 9 020)	84.2 (62.1 to 108.3)	24.2 (11.1 to 40.3)	-2.4 (-12.5 to 10.1)
4	Ambient particulate matter pollution	Tracheal, bronchus, and lung cancer	240 (175 to 303)	3.8 (2.8 to 4.8)	308 (227 to 396)	3.8 (2.8 to 4.9)	28.4 (15.6 to 44.3)	-0.6 (-10.4 to 11.4)	5 650 (4 130 to 7 130)	86.3 (63.0 to 109.0)	7 020 (5 180 to 9 020)	84.2 (62.1 to 108.3)	24.2 (11.1 to 40.3)	-2.4 (-12.5 to 10.1)
4	Household air pollution from solid fuels	Total cancers	96.0 (58.9 to 142)	1.5 (0.9 to 2.2)	79.8 (45.1 to 125)	1.0 (0.5 to 1.5)	-16.9 (-28.8 to -3.5)	-35.5 (-44.6 to -25.1)	2 390 (1 460 to 3 510)	36.1 (22.1 to 53.3)	1 940 (1 110 to 3 010)	23.1 (13.2 to 36.0)	-18.8 (-30.2 to -6.1)	-35.9 (-44.8 to -25.9)
4	Household air pollution from solid fuels	Tracheal, bronchus, and lung cancer	96.0 (58.9 to 142)	1.5 (0.9 to 2.2)	79.8 (45.1 to 125)	1.0 (0.5 to 1.5)	-16.9 (-28.8 to -3.5)	-35.5 (-44.6 to -25.1)	2 390 (1 460 to 3 510)	36.1 (22.1 to 53.3)	1 940 (1 110 to 3 010)	23.1 (13.2 to 36.0)	-18.8 (-30.2 to -6.1)	-35.9 (-44.8 to -25.9)
2	Other environmental risks	Total cancers	69.8 (13.6 to 135)	1.1 (0.2 to 2.2)	83.7 (16.5 to 162)	1.0 (0.2 to 2.0)	20.0 (12.1 to 29.0)	-7.4 (-13.4 to -0.5)	1 630 (320 to 3 150)	24.9 (4.9 to 48.3)	1 890 (374 to 3 650)	22.7 (4.5 to 43.9)	15.9 (8.0 to 25.3)	-9.1 (-15.2 to -1.7)
3	Residential radon	Total cancers	69.8 (13.6 to 135)	1.1 (0.2 to 2.2)	83.7 (16.5 to 162)	1.0 (0.2 to 2.0)	20.0 (12.1 to 29.0)	-7.4 (-13.4 to -0.5)	1 630 (320 to 3 150)	24.9 (4.9 to 48.3)	1 890 (374 to 3 650)	22.7 (4.5 to 43.9)	15.9 (8.0 to 25.3)	-9.1 (-15.2 to -1.7)
3	Residential radon	Tracheal, bronchus, and lung cancer	69.8 (13.6 to 135)	1.1 (0.2 to 2.2)	83.7 (16.5 to 162)	1.0 (0.2 to 2.0)	20.0 (12.1 to 29.0)	-7.4 (-13.4 to -0.5)	1 630 (320 to 3 150)	24.9 (4.9 to 48.3)	1 890 (374 to 3 650)	22.7 (4.5 to 43.9)	15.9 (8.0 to 25.3)	-9.1 (-15.2 to -1.7)
2	Occupational risks	Total cancers	289 (228 to 350)	4.7 (3.7 to 5.7)	334 (263 to 405)	4.2 (3.3 to 5.1)	15.6 (8.8 to 22.6)	-11. 4(-16.5 to -6.2)	6 160 (4 860 to 7 510)	95.9 (75.6 to 116.8)	6 960 (5 470 to 8 580)	84.4 (66.2 to 103.7)	13.0 (5.5 to 20.6)	-12.0 (-17.7 to -6.1)
3	Occupational carcinogens	Total cancers	289 (228 to 350)	4.7 (3.7 to 5.7)	334 (263 to 405)	4.2 (3.3 to 5.1)	15.6 (8.8 to 22.6)	-11.4 (-16.5 to -6.2)	6 160 (4 860 to 7 510)	95.9 (75.6 to 116.8)	6 960 (5 470 to 8 580)	84.4 (66.2 to 103.7)	13.0 (5.5 to 20.6)	-12.0 (-17.7 to -6.1)
4	Occupational exposure to asbestos	Total cancers	210 (155 to 266)	3.5 (2.6 to 4.5)	236 (176 to 296)	3.0 (2.2 to 3.8)	12.0 (5.8 to 18.2)	-14.6 (-19.2 to -10.1)	3 800 (2 800 to 4 840)	61.1 (45.0 to 77.6)	4 120 (3 060 to 5 240)	50.9 (37.8 to 64.7)	8.2 (1.5 to 15.4)	-16.7 (-21.8 to -11.5)
4	Occupational exposure to asbestos	Larynx cancer	3.24 (1.79 to 4.82)	0.1 (0.0 to 0.1)	3.68 (2.04 to 5.53)	0.0 (0.0 to 0.1)	13.6 (5.1 to 22.5)	-13.3 (-19.7 to -6.9)	63.8 (34.6 to 95.7)	1.0 (0.6 to 1.5)	70.0 (38.3 to 106)	0.9 (0.5 to 1.3)	9.8 (0.4 to 19.7)	-15.6 (-22.7 to -8.2)
4	Occupational exposure to asbestos	Tracheal, bronchus, and lung cancer	179 (125 to 233)	3.0 (2.1 to 3.9)	199 (140 to 257)	2.5 (1.8 to 3.3)	10.9 (4.2 to 17.5)	-15.6 (-20.5 to -10.6)	3 150 (2 160 to 4 160)	50.9 (35.0 to 67.0)	3 370 (2 340 to 4 450)	41.7 (29.0 to 55.0)	6.9 (-0.5 to 14.3)	-18.0 (-23.5 to -12.5)
4	Occupational exposure to asbestos	Ovarian cancer	5.50 (2.40 to 8.85)	0.1 (0.0 to 0.2)	6.56 (2.95 to 10.7)	0.1 (0.0 to 0.1)	19.3 (6.5 to 31.2)	-10.6 (-20.2 to -1.9)	96.0 (44.0 to 154)	1.6 (0.7 to 2.5)	113 (50.1 to 185)	1.4 (0.6 to 2.3)	18.0 (4.4 to 31.0)	-10.0 (-20.2 to -0.2)
4	Occupational exposure to asbestos	Mesothelioma	22.4 (20.4 to 23.8)	0.4 (0.3 to 0.4)	26.8 (24.3 to 28.6)	0.3 (0.3 to 0.4)	19.5 (15.2 to 23.6)	-7.9 (-11.1 to -4.8)	495 (443 to 535)	7.7 (6.9 to 8.2)	569 (510 to 617)	6.9 (6.2 to 7.5)	14.9 (9.8 to 20.0)	-9.8 (-13.7 to -5.8)

GBD Risk Level	Risk factor	Cancer	Both sexes combined											
			Deaths						DALYs					
			Deaths in 2010, thousands (95% UI)	Age-standardised death rates, per 100,000, in 2010 (95% UI)	Deaths in 2019, thousands (95% UI)	Age-standardised death rates, per 100,000, in 2019 (95% UI)	Percent change in absolute deaths, 2010 – 2019 (95% UI)	Percent change in age-standardised death rates, 2010 – 2019 (95% UI)	DALYs in 2010, thousands (95% UI)	Age-standardised DALY rates, per 100,000, in 2010 (95% UI)	DALYs in 2019, thousands (95% UI)	Age-standardised DALY rates, per 100,000, in 2019 (95% UI)	Percent change in absolute DALYs, 2010 – 2019 (95% UI)	Percent change in age-standardised DALY rates, 2010 – 2019 (95% UI)
4	Occupational exposure to arsenic	Total cancers	7.89 (1.17 to 14.4)	0.1 (0.0 to 0.2)	9.76 (1.55 to 17.7)	0.1 (0.0 to 0.2)	23.6 (11.4 to 43.7)	-3.3 (-12.7 to 12.7)	227 (36.2 to 411)	3.4 (0.5 to 6.1)	271 (44.8 to 486)	3.2 (0.5 to 5.7)	19.4 (7.3 to 38.6)	-5.5 (-15.0 to 10.2)
4	Occupational exposure to arsenic	Tracheal, bronchus, and lung cancer	7.89 (1.17 to 14.4)	0.1 (0.0 to 0.2)	9.76 (1.55 to 17.7)	0.1 (0.0 to 0.2)	23.6 (11.4 to 43.7)	-3.3 (-12.7 to 12.7)	227 (36.2 to 411)	3.4 (0.5 to 6.1)	271 (44.8 to 486)	3.2 (0.5 to 5.7)	19.4 (7.3 to 38.6)	-5.5 (-15.0 to 10.2)
4	Occupational exposure to benzene	Total cancers	1.63 (0.513 to 2.64)	0.0 (0.0 to 0.0)	1.87 (0.565 to 3.05)	0.0 (0.0 to 0.0)	14.2 (6.4 to 22.5)	-0.8 (-7.4 to 6.7)	77.4 (23.7 to 126)	1.1 (0.3 to 1.7)	85.8 (25.7 to 140)	1.1 (0.3 to 1.7)	10.9 (3.0 to 19.6)	-1.3 (-8.3 to 6.4)
4	Occupational exposure to benzene	Leukaemia	1.63 (0.513 to 2.64)	0.0 (0.0 to 0.0)	1.87 (0.565 to 3.05)	0.0 (0.0 to 0.0)	14.2 (6.4 to 22.5)	-0.8 (-7.4 to 6.7)	77.4 (23.7 to 126)	1.1 (0.3 to 1.7)	85.8 (25.7 to 140)	1.1 (0.3 to 1.7)	10.9 (3.0 to 19.6)	-1.3 (-8.3 to 6.4)
4	Occupational exposure to beryllium	Total cancers	0.233 (0.192 to 0.276)	0.0 (0.0 to 0.0)	0.301 (0.244 to 0.367)	0.0 (0.0 to 0.0)	28.7 (13.6 to 44.8)	0.9 (-10.7 to 13.5)	6.90 (5.68 to 8.15)	0.1 (0.1 to 0.1)	8.58 (6.95 to 10.5)	0.1 (0.1 to 0.1)	24.4 (9.8 to 40.0)	-1.1 (-12.5 to 10.8)
4	Occupational exposure to beryllium	Tracheal, bronchus, and lung cancer	0.233 (0.192 to 0.276)	0.0 (0.0 to 0.0)	0.301 (0.244 to 0.367)	0.0 (0.0 to 0.0)	28.7 (13.6 to 44.8)	0.9 (-10.7 to 13.5)	6.90 (5.68 to 8.15)	0.1 (0.1 to 0.1)	8.58 (6.95 to 10.5)	0.1 (0.1 to 0.1)	24.4 (9.8 to 40.0)	-1.1 (-12.5 to 10.8)
4	Occupational exposure to cadmium	Total cancers	0.549 (0.455 to 0.642)	0.0 (0.0 to 0.0)	0.712 (0.583 to 0.854)	0.0 (0.0 to 0.0)	29.7 (14.3 to 46.2)	1.7 (-10.3 to 14.0)	16.2 (13.6 to 18.9)	0.2 (0.2 to 0.3)	20.3 (16.7 to 24.1)	0.2 (0.2 to 0.3)	25.3 (10.2 to 41.2)	-0.5 (-12.3 to 11.7)
4	Occupational exposure to cadmium	Tracheal, bronchus, and lung cancer	0.549 (0.455 to 0.642)	0.0 (0.0 to 0.0)	0.712 (0.583 to 0.854)	0.0 (0.0 to 0.0)	29.7 (14.3 to 46.2)	1.7 (-10.3 to 14.0)	16.2 (13.6 to 18.9)	0.2 (0.2 to 0.3)	20.3 (16.7 to 24.1)	0.2 (0.2 to 0.3)	25.3 (10.2 to 41.2)	-0.5 (-12.3 to 11.7)
4	Occupational exposure to chromium	Total cancers	1.14 (1.00 to 1.28)	0.0 (0.0 to 0.0)	1.50 (1.29 to 1.75)	0.0 (0.0 to 0.0)	31.8 (17.0 to 47.6)	3.4 (-8.5 to 15.6)	33.5 (29.4 to 37.6)	0.5 (0.4 to 0.6)	42.7 (36.6 to 49.7)	0.5 (0.4 to 0.6)	27.4 (13.0 to 42.9)	1.2 (-10.2 to 13.1)
4	Occupational exposure to chromium	Tracheal, bronchus, and lung cancer	1.14 (1.00 to 1.28)	0.0 (0.0 to 0.0)	1.50 (1.29 to 1.75)	0.0 (0.0 to 0.0)	31.8 (17.0 to 47.6)	3.4 (-8.5 to 15.6)	33.5 (29.4 to 37.6)	0.5 (0.4 to 0.6)	42.7 (36.6 to 49.7)	0.5 (0.4 to 0.6)	27.4 (13.0 to 42.9)	1.2 (-10.2 to 13.1)
4	Occupational exposure to diesel engine exhaust	Total cancers	14.7 (12.9 to 16.9)	0.2 (0.2 to 0.3)	19.7 (17.0 to 22.9)	0.2 (0.2 to 0.3)	33.7 (20.0 to 48.8)	4.8 (-5.8 to 16.9)	435 (380 to 497)	6.4 (5.6 to 7.4)	563 (485 to 655)	6.6 (5.7 to 7.7)	29.5 (15.9 to 44.1)	2.9 (-7.6 to 14.8)
4	Occupational exposure to diesel engine exhaust	Tracheal, bronchus, and lung cancer	14.7 (12.9 to 16.9)	0.2 (0.2 to 0.3)	19.7 (17.0 to 22.9)	0.2 (0.2 to 0.3)	33.7 (20.0 to 48.8)	4.8 (-5.8 to 16.9)	435 (380 to 497)	6.4 (5.6 to 7.4)	563 (485 to 655)	6.6 (5.7 to 7.7)	29.5 (15.9 to 44.1)	2.9 (-7.6 to 14.8)
4	Occupational exposure to formaldehyde	Total cancers	1.01 (0.818 to 1.22)	0.0 (0.0 to 0.0)	1.12 (0.900 to 1.36)	0.0 (0.0 to 0.0)	11.0 (0.4 to 22.1)	-3.9 (-12.6 to 5.6)	46.9 (38.1 to 57.5)	0.6 (0.5 to 0.8)	50.8 (40.9 to 61.7)	0.6 (0.5 to 0.8)	8.4 (-1.5 to 19.3)	-4.2 (-12.6 to 5.3)
4	Occupational exposure to formaldehyde	Nasopharynx cancer	0.460 (0.313 to 0.639)	0.0 (0.0 to 0.0)	0.518 (0.355 to 0.731)	0.0 (0.0 to 0.0)	12.6 (-0.5 to 29.5)	-3.7 (-14.6 to 9.1)	20.1 (13.4 to 28.0)	0.3 (0.2 to 0.4)	22.3 (15.1 to 31.5)	0.3 (0.2 to 0.4)	10.9 (-2.5 to 27.5)	-3.4 (-14.3 to 9.2)
4	Occupational exposure to formaldehyde	Leukaemia	0.547 (0.460 to 0.644)	0.0 (0.0 to 0.0)	0.600 (0.497 to 0.712)	0.0 (0.0 to 0.0)	9.7 (-0.2 to 19.8)	-4.0 (-12.3 to 4.9)	26.7 (22.2 to 31.9)	0.4 (0.3 to 0.4)	28.5 (23.4 to 34.3)	0.4 (0.3 to 0.4)	6.5 (-3.1 to 16.5)	-4.8 (-13.5 to 4.1)
4	Occupational exposure to nickel	Total cancers	7.60 (0.295 to 20.6)	0.1 (0.0 to 0.3)	9.33 (0.536 to 24.6)	0.1 (0.0 to 0.3)	22.8 (9.1 to 48.4)	-3.9 (-14.9 to 17.1)	220 (11.6 to 589)	3.3 (0.2 to 8.8)	261 (18.3 to 677)	3.1 (0.2 to 8.0)	18.6 (5.2 to 42.6)	-6.0 (-16.8 to 14.6)
4	Occupational exposure to nickel	Tracheal, bronchus, and lung cancer	7.60 (0.295 to 20.6)	0.1 (0.0 to 0.3)	9.33 (0.536 to 24.6)	0.1 (0.0 to 0.3)	22.8 (9.1 to 48.4)	-3.9 (-14.9 to 17.1)	220 (11.6 to 589)	3.3 (0.2 to 8.8)	261 (18.3 to 677)	3.1 (0.2 to 8.0)	18.6 (5.2 to 42.6)	-6.0 (-16.8 to 14.6)
4	Occupational exposure to polycyclic aromatic hydrocarbons	Total cancers	4.00 (3.36 to 4.68)	0.1 (0.1 to 0.1)	5.27 (4.36 to 6.24)	0.1 (0.1 to 0.1)	31.8 (17.6 to 47.8)	3.3 (-8.0 to 15.7)	118 (99.4 to 139)	1.7 (1.5 to 2.0)	150 (123 to 177)	1.8 (1.5 to 2.1)	27.4 (13.7 to 42.8)	1.2 (-9.6 to 13.2)

GBD Risk Level	Risk factor	Cancer	Both sexes combined											
			Deaths						DALYs					
			Deaths in 2010, thousands (95% UI)	Age-standardised death rates, per 100,000, in 2010 (95% UI)	Deaths in 2019, thousands (95% UI)	Age-standardised death rates, per 100,000, in 2019 (95% UI)	Percent change in absolute deaths, 2010 – 2019 (95% UI)	Percent change in age-standardised death rates, 2010 – 2019 (95% UI)	DALYs in 2010, thousands (95% UI)	Age-standardised DALY rates, per 100,000, in 2010 (95% UI)	DALYs in 2019, thousands (95% UI)	Age-standardised DALY rates, per 100,000, in 2019 (95% UI)	Percent change in absolute DALYs, 2010 – 2019 (95% UI)	Percent change in age-standardised DALY rates, 2010 – 2019 (95% UI)
4	Occupational exposure to polycyclic aromatic hydrocarbons	Tracheal, bronchus, and lung cancer	4.00 (3.36 to 4.68)	0.1 (0.1 to 0.1)	5.27 (4.36 to 6.24)	0.1 (0.1 to 0.1)	31.8 (17.6 to 47.8)	3.3 (-8.0 to 15.7)	118 (99.4 to 139)	1.7 (1.5 to 2.0)	150 (123 to 177)	1.8 (1.5 to 2.1)	27.4 (13.7 to 42.8)	1.2 (-9.6 tSo 13.2)
4	Occupational exposure to silica	Total cancers	43.4 (19.1 to 68.8)	0.7 (0.3 to 1.0)	53.0 (23.8 to 84.4)	0.6 (0.3 to 1.0)	22.0 (11.7 to 33.7)	-4.6 (-12.7 to 4.6)	1 250 (552 to 1 980)	18.6 (8.2 to 29.4)	1 480 (666 to 2 350)	17.4 (7.8 to 27.7)	18.0 (7.7 to 29.5)	-6.6 (-14.8 to 2.5)
4	Occupational exposure to silica	Tracheal, bronchus, and lung cancer	43.4 (19.1 to 68.8)	0.7 (0.3 to 1.0)	53.0 (23.8 to 84.4)	0.6 (0.3 to 1.0)	22.0 (11.7 to 33.7)	-4.6 (-12.7 to 4.6)	1 250 (552 to 1 980)	18. 6(8.2 to 29.4)	1 480 (666 to 2 350)	17.4 (7.8 to 27.7)	18.0 (7.7 to 29.5)	-6.6 (-14.8 to 2.5)
4	Occupational exposure to sulfuric acid	Total cancers	3.41 (1.45 to 6.18)	0.1 (0.0 to 0.1)	4.03 (1.73 to 7.47)	0.0 (0.0 to 0.1)	18.1 (7.8 to 29.1)	-6.7 (-14.9 to 1.9)	109 (46.3 to 198)	1.6 (0.7 to 2.9)	126 (54.1 to 234)	1.5 (0.6 to 2.7)	15.6 (5.7 to 26.4)	-7.4 (-15.3 to 1.3)
4	Occupational exposure to sulfuric acid	Larynx cancer	3.41 (1.45 to 6.18)	0.1 (0.0 to 0.1)	4.03 (1.73 to 7.47)	0.0 (0.0 to 0.1)	18.1 (7.8 to 29.1)	-6.7 (-14.9 to 1.9)	109 (46.3 to 198)	1.6 (0.7 to 2.9)	126 (54.1 to 234)	1.5 (0.6 to 2.7)	15.6 (5.7 to 26.4)	-7.4 (-15.3 to 1.3)
4	Occupational exposure to trichloroethylene	Total cancers	0.0559 (0.0122 to 0.104)	0.0 (0.0 to 0.0)	0.0785 (0.0168 to 0.147)	0.0 (0.0 to 0.0)	40.5 (29.8 to 52.7)	11.2 (2.7 to 20.8)	1.77 (0.388 to 3.31)	0.0 (0.0 to 0.0)	2.43 (0.518 to 4.54)	0.0 (0.0 to 0.1)	37.5 (26.4 to 49.5)	10.7 (1.7 to 20.4)
4	Occupational exposure to trichloroethylene	Kidney cancer	0.0559 (0.0122 to 0.104)	0.0 (0.0 to 0.0)	0.0785 (0.0168 to 0.147)	0.0 (0.0 to 0.0)	40.5 (29.8 to 52.7)	11.2 (2.7 to 20.8)	1.77 (0.388 to 3.31)	0.0 (0.0 to 0.0)	2.43 (0.518 to 4.54)	0.0 (0.0 to 0.1)	37.5 (26.4 to 49.5)	10.7 (1.7 to 20.4)
**1**	**Behavioural risks**	**Total cancers**	**3 140 (2 950 to 3 350)**	**49.9 (46.8 to 53.2)**	**3 700 (3 420 to 4 020)**	**45.5 (42.1 to 49.4)**	**17.9 (10.4 to 26.0)**	**-8.7 (-14.5 to -2.7)**	**76 700 (72 600 to 81 900)**	**1 166.8 (1 103.0 to 1 245.7)**	**87 800 (81 100 to 95 400)**	**1 054.7 (974.1 to 1 145.3)**	**14.4 (6.5 to 22.5)**	**-9.6 (-15.8 to -3.2)**
2	Tobacco	Total cancers	2 240 (2 100 to 2 350)	35.6 (33.4 to 37.5)	2 600 (2 380 to 2 830)	31.9 (29.2 to 34.7)	16.2 (7.5 to 25.6)	-10.4 (-16.9 to -3.2)	52 700 (49 600 to 55 600)	807.4 (760.5 to 851.2)	59 300 (54 000 to 64 800)	711.7 (648.9 to 777.1)	12.6 (3.7 to 22.3)	-11.9 (-18.8 to -4.3)
3	Smoking	Total cancers	2 160 (2 030 to 2 270)	34.4 (32.2 to 36.3)	2 490 (2 280 to 2 720)	30.6 (28.0 to 33.3)	15.6 (6.9 to 25.3)	-10.9 (-17.6 to -3.5)	50 400 (47 600 to 53 300)	774.1 (729.9 to 818.1)	56 400 (51 300 to 61 700)	677.3 (616.4 to 740.3)	11.9 (2.8 to 21.8)	-12.5 (-19.6 to -4.8)
3	Smoking	Lip and oral cavity cancer	54.6 (45.3 to 63.2)	0.8 (0.7 to 1.0)	63.4 (51.2 to 76.4)	0.8 (0.6 to 0.9)	16.2 (5.2 to 26.7)	-9.5 (-18.0 to -1.2)	1 480 (1 180 to 1 750)	22.1 (17.8 to 26.1)	1 660 (1 310 to 2 020)	19.7 (15.6 to 23.9)	12.1 (1.5 to 23.1)	-11.1 (-19.7 to -2.4)
3	Smoking	Nasopharynx cancer	15.8 (11.4 to 20.1)	0.2 (0.2 to 0.3)	17.9 (13.0 to 23.0)	0.2 (0.2 to 0.3)	13.4 (-0.6 to 28.7)	-10. 3(-21.4 to 1.7)	476 (339 to 611)	7.0 (5.0 to 8.9)	527 (374 to 684)	6.2 (4.4 to 8.1)	10.6 (-2.8 to 25.4)	-10.7 (-21.9 to 1.0)
3	Smoking	Other pharynx cancer	43.3 (37.8 to 48.6)	0.7 (0.6 to 0.7)	53.6 (45.2 to 61.9)	0.6 (0.5 to 0.7)	23.9 (11.3 to 36.1)	-3.1 (-12.9 to 6.3)	1 200 (1 030 to 1 360)	18.0 (15.5 to 20.3)	1 440 (1 200 to 1 680)	17.1 (14.2 to 19.9)	19.8 (6.8 to 32.0)	-5.1 (-15.3 to 4.7)
3	Smoking	Oesophageal cancer	187 (149 to 209)	2.9 (2.3 to 3.3)	203 (170 to 237)	2.5 (2.1 to 2.9)	8.6 (-6.5 to 27.5)	-15.9 (-27.5 to -1.4)	4 560 (3 690 to 5 120)	69.5 (56.0 to 77.9)	4 750 (3 980 to 5 540)	56.7 (47.6 to 66.1)	4.0 (-11.2 to 23.2)	-18.3 (-30.0 to -3.4)
3	Smoking	Stomach cancer	174 (141 to 205)	2.8 (2.2 to 3.3)	172 (138 to 207)	2.1 (1.7 to 2.5)	-0.9 (-12.8 to 12.6)	-23.5 (-32.4 to -13.3)	3 990 (3 200 to 4 780)	61.4 (49.4 to 73.4)	3 810 (2 990 to 4 630)	45.8 (36.1 to 55.6)	-4.5 (-16.7 to 9.7)	-25.3 (-34.7 to -14.4)
3	Smoking	Colon and rectum cancer	120 (79.7 to 160)	1.9 (1.3 to 2.6)	143 (95.5 to 193)	1.8 (1.2 to 2.4)	19.3 (11.4 to 27.9)	-8.4 (-14.4 to -1.9)	2 760 (1 810 to 3 650)	42.7 (28.1 to 56.5)	3 230 (2 090 to 4 400)	38.9 (25.3 to 53.0)	16.8 (8.5 to 26.2)	-8.9 (-15.3 to -1.7)
3	Smoking	Liver cancer	70.3 (38.4 to 98.1)	1.1 (0.6 to 1.5)	85.9 (50.0 to 123)	1.0 (0.6 to 1.5)	22.1 (9.5 to 36.6)	-5.0 (-14.7 to 5.7)	1 780 (947 to 2 550)	26.9 (14.3 to 38.1)	2 130 (1 160 to 3 070)	25.3 (13.8 to 36.5)	19.2 (5.1 to 35.2)	-5.7 (-16.5 to 6.5)
3	Smoking	Pancreatic cancer	89.9 (79.5 to 101)	1.5 (1.3 to 1.6)	113 (98.8 to 128)	1.4 (1.2 to 1.6)	26.1 (19.0 to 33.6)	-3.5 (-8.7 to 2.1)	1 990 (1 750 to 2 240)	30.8 (27.0 to 34.6)	2 440 (2 110 to 2 770)	29.4 (25.4 to 33.4)	22.9 (15.3 to 31.1)	-4.4 (-10.4 to 1.9)
3	Smoking	Larynx cancer	69.4 (61.4 to 76.0)	1.1 (1.0 to 1.2)	78.3 (68.0 to 88.3)	0.9 (0.8 to 1.1)	12.7 (4.6 to 20.9)	-12.4 (-18.6 to -6.1)	1 850 (1 630 to 2 020)	27.9 (24.6 to 30.5)	2 020 (1 760 to 2 300)	24.0 (20.9 to 27.3)	9.5 (1.9 to 17.9)	-13.7 (-19.7 to -7.0)
3	Smoking	Tracheal, bronchus, and lung cancer	1 110 (1 060 to 1 170)	17.8 (16.9 to 18.7)	1 310 (1 200 to 1 430)	16.1 (14.7 to 17.5)	17.7 (8.0 to 28.4)	-9.5 (-16.9 to -1.4)	25 100 (23 900 to 26 300)	387.4 (369.0 to 406.4)	28 600 (26 000 to 31 300)	344.0 (313.2 to 375.3)	14.1 (4.2 to 25.5)	-11.2 (-18.9 to -2.6)
3	Smoking	Breast cancer	17.4 (12.6 to 22.8)	0.3 (0.2 to 0.4)	19.0 (13.6 to 24.8)	0.2 (0.2 to 0.3)	8.8 (4.7 to 13.3)	-15.7 (-18.8 to -12.0)	493 (351 to 647)	7.4 (5.3 to 9.7)	513 (362 to 674)	6.1 (4.3 to 8.1)	4.2 (0.0 to 8.8)	-17.3 (-20.7 to -13.6)
3	Smoking	Cervical cancer	28.8 (14.5 to 46.3)	0.4 (0.2 to 0.7)	30.1 (14.9 to 49.6)	0.4 (0.2 to 0.6)	4.7 (-3.8 to 13.1)	-17.1 (-23.6 to -10.1)	880 (464 to 1 410)	12.9 (6.7 to 20.7)	894 (469 to 1 440)	10.6 (5.6 to 17.1)	1.6 (-6.4 to 10.1)	-17.4 (-24.0 to -10.3)

GBD Risk Level	Risk factor	Cancer	Both sexes combined											
			Deaths						DALYs					
			Deaths in 2010, thousands (95% UI)	Age-standardised death rates, per 100,000, in 2010 (95% UI)	Deaths in 2019, thousands (95% UI)	Age-standardised death rates, per 100,000, in 2019 (95% UI)	Percent change in absolute deaths, 2010 – 2019 (95% UI)	Percent change in age-standardised death rates, 2010 – 2019 (95% UI)	DALYs in 2010, thousands (95% UI)	Age-standardised DALY rates, per 100,000, in 2010 (95% UI)	DALYs in 2019, thousands (95% UI)	Age-standardised DALY rates, per 100,000, in 2019 (95% UI)	Percent change in absolute DALYs, 2010 – 2019 (95% UI)	Percent change in age-standardised DALY rates, 2010 – 2019 (95% UI)
3	Smoking	Prostate cancer	25.2<br>(11.3 to 39.7)	0.4<br>(0.2 to 0.7)	29.3<br>(12.8 to 46.6)	0.4<br>(0.2 to 0.6)	16.4<br>(9.2 to 23.8)	-12.0<br>(-17.4 to -6.6)	496<br>(220 to 775)	8.0<br>(3.5 to 12.5)	572<br>(253 to 918)	7.0<br>(3.1 to 11.3)	15.2<br>(8.0 to 22.7)	-12.0<br>(-17.5 to -6.3)
3	Smoking	Kidney cancer	25.3<br>(18.0 to 32.7)	0.4<br>(0.3 to 0.5)	30.1<br>(21.0 to 39.4)	0.4<br>(0.3 to 0.5)	19.1<br>(12.1 to 25.8)	-8.4<br>(-13.6 to -3.4)	595<br>(417 to 764)	9.1<br>(6.4 to 11.8)	687<br>(476 to 883)	8.3<br>(5.7 to 10.7)	15.6<br>(8.6 to 22.8)	-9.7<br>(-15.3 to -4.3)
3	Smoking	Bladder cancer	65.4<br>(49.9 to 80.0)	1.1<br>(0.8 to 1.3)	77.5<br>(58.3 to 96.7)	1.0<br>(0.7 to 1.2)	18.6<br>(10.9 to 26.7)	-9.6<br>(-15.3 to -3.7)	1 400<br>(1 080 to 1 680)	21.9<br>(16.9 to 26.4)	1 620<br>(1 250 to 1 980)	19.7<br>(15.2 to 24.1)	15.7<br>(7.6 to 24.0)	-10.3<br>(-16.4 to -3.8)
3	Smoking	Leukaemia	56.9<br>(35.4 to 79.6)	0.9<br>(0.6 to 1.3)	64.6<br>(39.3 to 91.4)	0.8<br>(0.5 to 1.1)	13.6<br>(6.7 to 20.3)	-12.0<br>(-17.0 to -7.0)	1 400<br>(829 to 1 990)	21.3<br>(12.9 to 30.2)	1 530<br>(896 to 2 160)	18.5<br>(10.8 to 26.0)	9.3<br>(2.0 to 16.8)	-13.3<br>(-18.9 to -7.5)
3	Chewing tobacco	Total cancers	41.2<br>(33.0 to 50.0)	0.6<br>(0.5 to 0.8)	55.6<br>(43.1 to 68.8)	0.7<br>(0.5 to 0.8)	34.9<br>(19.0 to 52.4)	6.2<br>(-6.2 to 19.9)	1 160<br>(920 to 1 420)	17.2<br>(13.7 to 21.0)	1 500<br>(1 160 to 1 880)	17.9<br>(13.9 to 22.4)	29.8<br>(13.6 to 47.9)	4.3<br>(-8.6 to 18.7)
3	Chewing tobacco	Lip and oral cavity cancer	26.8<br>(20.5 to 33.2)	0.4<br>(0.3 to 0.5)	37.3<br>(27.9 to 47.2)	0.5<br>(0.3 to 0.6)	39.1<br>(20.4 to 60.2)	9.8<br>(-4.9 to 26.5)	772<br>(581 to 964)	11.4<br>(8.6 to 14.2)	1 030<br>(764 to 1 320)	12.3<br>(9.1 to 15.7)	33.2<br>(15.1 to 54.7)	7.5<br>(-7.2 to 24.6)
3	Chewing tobacco	Oesophageal cancer	14.4<br>(10.1 to 19.1)	0.2<br>(0.2 to 0.3)	18.3<br>(12.7 to 24.7)	0.2<br>(0.2 to 0.3)	27.0<br>(13.7 to 40.7)	-0.7<br>(-10.9 to 10.0)	387<br>(273 to 518)	5.8<br>(4.1 to 7.8)	476<br>(328 to 644)	5.7<br>(3.9 to 7.7)	23.0<br>(9.9 to 37.5)	-2.1<br>(-12.5 to 9.3)
3	Secondhand smoke	Total cancers	105<br>(68.6 to 151)	1.7<br>(1.1 to 2.4)	130<br>(82.6 to 190)	1.6<br>(1.0 to 2.3)	23.8<br>(12.8 to 35.8)	-3.5<br>(-12.0 to 5.8)	2 700<br>(1 760 to 3 830)	40.7<br>(26.6 to 57.9)	3 220<br>(2 070 to 4 630)	38.5<br>(24.8 to 55.5)	19.3<br>(8.7 to 30.9)	-5.2<br>(-13.7 to 4.0)
3	Secondhand smoke	Tracheal, bronchus, and lung cancer	91.5<br>(54.3 to 135)	1.4<br>(0.9 to 2.1)	113<br>(67.5 to 170)	1.4<br>(0.8 to 2.1)	24.0<br>(12.2 to 36.6)	-3.8<br>(-12.9 to 5.8)	2 230<br>(1 330 to 3 270)	33.9<br>(20.3 to 49.7)	2 660<br>(1 580 to 3 990)	31.8<br>(19.0 to 47.8)	19.2<br>(7.7 to 31.7)	-6.0<br>(-15.1 to 3.6)
3	Secondhand smoke	Breast cancer	13.9<br>(3.29 to 23.6)	0.2<br>(0.0 to 0.4)	17.0<br>(4.00 to 29.4)	0.2<br>(0.0 to 0.4)	22.5<br>(12.9 to 32.8)	-1.5<br>(-9.2 to 6.6)	471<br>(114 to 809)	6.8<br>(1.6 to 11.7)	562<br>(135 to 976)	6.7<br>(1.6 to 11.6)	19.4<br>(9.9 to 29.9)	-1.3<br>(-9.1 to 7.4)
2	Alcohol use	Total cancers	406<br>(364 to 452)	6.3<br>(5.7 to 7.1)	495<br>(440 to 554)	6.0<br>(5.4 to 6.8)	21.8<br>(12.7 to 31.5)	-5.0<br>(-11.9 to 2.4)	11 000<br>(9 910 to 12 200)	164.4<br>(148.3 to 182.3)	13 000<br>(11 600 to 14 500)	155.2<br>(138.4 to 173.5)	18.3<br>(8.9 to 28.2)	-5.6<br>(-12.9 to 2.2)
2	Alcohol use	Lip and oral cavity cancer	48.6<br>(40.0 to 56.4)	0.7<br>(0.6 to 0.9)	60.4<br>(48.0 to 72.4)	0.7<br>(0.6 to 0.9)	24.3<br>(12.6 to 35.9)	-2.1<br>(-11.3 to 6.9)	1 430<br>(1 180 to 1 670)	21.2<br>(17.4 to 24.6)	1 730<br>(1 380 to 2 070)	20.6<br>(16.4 to 24.6)	20.4<br>(8.3 to 32.1)	-2.8<br>(-12.5 to 6.7)
2	Alcohol use	Nasopharynx cancer	20.3<br>(15.7 to 24.6)	0.3<br>(0.2 to 0.4)	24.5<br>(18.8 to 29.9)	0.3<br>(0.2 to 0.4)	20.7<br>(6.1 to 37.3)	-2.7<br>(-14.5 to 10.7)	686<br>(534 to 831)	9.9<br>(7.7 to 12.0)	811<br>(628 to 993)	9.6<br>(7.5 to 11.8)	18.2<br>(4.1 to 34.6)	-2.3<br>(-13.9 to 11.1)
2	Alcohol use	Other pharynx cancer	29.0<br>(22.6 to 35.3)	0.4<br>(0.3 to 0.5)	37.9<br>(29.0 to 47.0)	0.5<br>(0.3 to 0.6)	30.6<br>(16.7 to 43.9)	3.1<br>(-7.9 to 13.6)	871<br>(681 to 1 060)	12.8<br>(10.0 to 15.6)	1 110<br>(854 to 1 370)	13.1<br>(10.1 to 16.2)	27.0<br>(12.6 to 40.7)	2.2<br>(-9.5 to 13.1)
2	Alcohol use	Oesophageal cancer	99.2<br>(71.4 to 127)	1.5<br>(1.1 to 2.0)	114<br>(84.1 to 145)	1.4<br>(1.0 to 1.8)	14.5<br>(-1.3 to 33.2)	-10.9<br>(-23.1 to 3.7)	2 570<br>(1 860 to 3 250)	38.7<br>(28.0 to 49.1)	2 820<br>(2 110 to 3 570)	33.6<br>(25.1 to 42.5)	9.8<br>(-5.8 to 28.5)	-13.2<br>(-25.5 to 1.6)
2	Alcohol use	Colon and rectum cancer	81.7<br>(62.9 to 101)	1.3<br>(1.0 to 1.6)	101<br>(76.6 to 127)	1.3<br>(1.0 to 1.6)	23.3<br>(15.2 to 31.7)	-5.2<br>(-11.2 to 1.0)	1 990<br>(1 520 to 2 450)	30.4<br>(23.2 to 37.4)	2 410<br>(1 830 to 3 000)	29.1<br>(22.1 to 36.2)	21.0<br>(12.3 to 30.3)	-4.4<br>(-11.1 to 2.8)
2	Alcohol use	Liver cancer	73.3<br>(59.8 to 88.1)	1.1<br>(0.9 to 1.4)	96.1<br>(77.5 to 116)	1.2<br>(0.9 to 1.4)	31.0<br>(21.9 to 40.4)	2.0<br>(-4.9 to 9.1)	1 860<br>(1 500 to 2 270)	28.1<br>(22.7 to 34.0)	2 380<br>(1 910 to 2 890)	28.4<br>(22.9 to 34.5)	27.8<br>(17.7 to 38.3)	1.3<br>(-6.4 to 9.4)
2	Alcohol use	Larynx cancer	20.5<br>(12.1 to 27.7)	0.3<br>(0.2 to 0.4)	23.9<br>(14.1 to 32.6)	0.3<br>(0.2 to 0.4)	16.8<br>(7.6 to 26.1)	-8.8<br>(-15.7 to -1.6)	577<br>(341 to 772)	8.6<br>(5.1 to 11.5)	656<br>(395 to 895)	7.8<br>(4.7 to 10.6)	13.7<br>(4.6 to 23.1)	-9.8<br>(-17.1 to -2.3)
2	Alcohol use	Breast cancer	33.6<br>(27.5 to 39.9)	0.5<br>(0.4 to 0.6)	37.7<br>(30.7 to 45.1)	0.5<br>(0.4 to 0.6)	12.3<br>(7.6 to 17.4)	-12.4<br>(-15.9 to -8.5)	997<br>(815 to 1 180)	14.8<br>(12.1 to 17.6)	1 090<br>(880 to 1 300)	13.1<br>(10.6 to 15.6)	9.1<br>(3.9 to 14.7)	-11.9<br>(-16.1 to -7.4)
2	Drug use	Total cancers	52.1<br>(41.6 to 64.4)	0.8<br>(0.7 to 1.0)	71.5<br>(57.1 to 89.2)	0.9<br>(0.7 to 1.1)	37.1<br>(27.3 to 47.8)	6.5<br>(-0.8 to 14.8)	1 230<br>(987 to 1 510)	18.8<br>(15.0 to 23.1)	1 610<br>(1 290 to 1 990)	19.4<br>(15.6 to 23.9)	31.1<br>(20.9 to 42.1)	3.2<br>(-4.7 to 11.6)
2	Drug use	Liver cancer	52.1<br>(41.6 to 64.4)	0.8<br>(0.7 to 1.0)	71.5<br>(57.1 to 89.2)	0.9<br>(0.7 to 1.1)	37.1<br>(27.3 to 47.8)	6.5<br>(-0.8 to 14.8)	1 230<br>(987 to 1 510)	18.8<br>(15.0 to 23.1)	1 610<br>(1 290 to 1 990)	19.4<br>(15.6 to 23.9)	31.1<br>(20.9 to 42.1)	3.2<br>(-4.7 to 11.6)
2	Dietary risks	Total cancers	516<br>(385 to 720)	8.4<br>(6.2 to 11.6)	605<br>(454 to 811)	7.6<br>(5.7 to 10.1)	17.3<br>(8.5 to 25.5)	-9.5<br>(-15.9 to -3.5)	12 300<br>(9 010 to 17 200)	187.7<br>(138.0 to 262.9)	14 000<br>(10 500 to 18 800)	168.8<br>(127.1 to 226.9)	13.6<br>(4.6 to 22.3)	-10.1<br>(-17.1 to -3.3)
3	Diet low in fruits	Total cancers	119<br>(59.7 to 187)	1.9<br>(0.9 to 3.0)	128<br>(65.0 to 200)	1.6<br>(0.8 to 2.5)	8.1<br>(-3.3 to 19.9)	-16.2<br>(-24.9 to -7.3)	2 870<br>(1 470 to 4 510)	43.6<br>(22.1 to 68.7)	3 000<br>(1 540 to 4 680)	36.0<br>(18.5 to 56.2)	4.5<br>(-6.8 to 16.3)	-17.5<br>(-26.5 to -8.1)
3	Diet low in fruits	Oesophageal cancer	51.5<br>(15.1 to 109)	0.8<br>(0.2 to 1.7)	51.2<br>(15.2 to 109)	0.6<br>(0.2 to 1.3)	-0.6<br>(-13.1 to 16.5)	-22.7<br>(-32.3 to -9.4)	1 290<br>(385 to 2 680)	19.5<br>(5.8 to 40.6)	1 250<br>(384 to 2 600)	15.0<br>(4.6 to 31.1)	-3.0<br>(-15.3 to 14.3)	-23.1<br>(-32.8 to -9.1)

GBD Risk Level	Risk factor	Cancer	Both sexes combined											
			**Deaths**						**DALYs**					
			**Deaths in 2010, thousands (95% UI)**	**Age-standardised death rates, per 100,000, in 2010 (95% UI)**	**Deaths in 2019, thousands (95% UI)**	**Age-standardised death rates, per 100,000, in 2019 (95% UI)**	**Percent change in absolute deaths, 2010 – 2019 (95% UI)**	**Percent change in age-standardised death rates, 2010 – 2019 (95% UI)**	**DALYs in 2010, thousands (95% UI)**	**Age-standardised DALY rates, per 100,000, in 2010 (95% UI)**	**DALYs in 2019, thousands (95% UI)**	**Age-standardised DALY rates, per 100,000, in 2019 (95% UI)**	**Percent change in absolute DALYs, 2010 – 2019 (95% UI)**	**Percent change in age-standardised DALY rates, 2010 – 2019 (95% UI)**
3	Diet low in fruits	Tracheal, bronchus, and lung cancer	67.3<br>(19.3 to 100)	1.1<br>(0.3 to 1.6)	77.2<br>(22.6 to 115)	1.0<br>(0.3 to 1.4)	14.7<br>(5.8 to 25.0)	-11.3<br>(-18.0 to -3.5)	1 580<br>(461 to 2 350)	24.1<br>(7.0 to 36.0)	1 750<br>(518 to 2 610)	21.0<br>(6.2 to 31.4)	10.7<br>(1.5 to 20.8)	-12.9<br>(-20.0 to -4.9)
3	Diet low in vegetables	Total cancers	15.1<br>(2.28 to 29.7)	0.2<br>(0.0 to 0.5)	17.2<br>(2.55 to 34.0)	0.2<br>(0.0 to 0.4)	14.1<br>(5.4 to 23.3)	-12.1<br>(-18.7 to -4.5)	371<br>(58.1 to 729)	5.6<br>(0.9 to 11.1)	420<br>(64.2 to 828)	5.0<br>(0.8 to 9.9)	13.2<br>(4.2 to 22.3)	-10.6<br>(-17.9 to -3.3)
3	Diet low in vegetables	Oesophageal cancer	15.1<br>(2.28 to 29.7)	0.2<br>(0.0 to 0.5)	17.2<br>(2.55 to 34.0)	0.2<br>(0.0 to 0.4)	14.1<br>(5.4 to 23.3)	-12.1<br>(-18.7 to -4.5)	371<br>(58.1 to 729)	5.6<br>(0.9 to 11.1)	420<br>(64.2 to 828)	5.0<br>(0.8 to 9.9)	13.2<br>(4.2 to 22.3)	-10.6<br>(-17.9 to -3.3)
3	Diet low in whole grains	Total cancers	137<br>(52.9 to 178)	2.3<br>(0.9 to 2.9)	171<br>(66.7 to 225)	2.2<br>(0.8 to 2.8)	25.5<br>(18.9 to 32.0)	-3.9<br>(-8.8 to 0.8)	3 110<br>(1 190 to 4 090)	48.1<br>(18.4 to 63.0)	3 810<br>(1 460 to 5 020)	46.3<br>(17.8 to 61.1)	22.4<br>(15.3 to 29.5)	-3.6<br>(-9.1 to 1.9)
3	Diet low in whole grains	Colon and rectum cancer	137<br>(52.9 to 178)	2.3<br>(0.9 to 2.9)	171<br>(66.7 to 225)	2.2<br>(0.8 to 2.8)	25.5<br>(18.9 to 32.0)	-3.9<br>(-8.8 to 0.8)	3 110<br>(1 190 to 4 090)	48.1<br>(18.4 to 63.0)	3 810<br>(1 460 to 5 020)	46.3<br>(17.8 to 61.1)	22.4<br>(15.3 to 29.5)	-3.6<br>(-9.1 to 1.9)
3	Diet low in milk	Total cancers	126<br>(80.9 to 171)	2.1<br>(1.3 to 2.8)	166<br>(107 to 226)	2.1<br>(1.3 to 2.8)	32.4<br>(24.5 to 41.0)	1.6<br>(-4.3 to 8.1)	2 950<br>(1 920 to 3 990)	45.3<br>(29.4 to 61.2)	3 800<br>(2 460 to 5 120)	46.1<br>(29.8 to 62.2)	28.7<br>(20.5 to 37.7)	1.7<br>(-4.8 to 8.9)
3	Diet low in milk	Colon and rectum cancer	126<br>(80.9 to 171)	2.1<br>(1.3 to 2.8)	166<br>(107 to 226)	2.1<br>(1.3 to 2.8)	32.4<br>(24.5 to 41.0)	1.6<br>(-4.3 to 8.1)	2 950<br>(1 920 to 3 990)	45.3<br>(29.4 to 61.2)	3 800<br>(2 460 to 5 120)	46.1<br>(29.8 to 62.2)	28.7<br>(20.5 to 37.7)	1.7<br>(-4.8 to 8.9)
3	Diet high in red meat	Total cancers	59.2<br>(28.1 to 99.5)	1.0<br>(0.4 to 1.6)	75.3<br>(35.9 to 126)	0.9<br>(0.4 to 1.6)	27.2<br>(20.2 to 36.6)	-1.9<br>(-7.3 to 5.5)	1 520<br>(779 to 2 450)	23.1<br>(11.7 to 37.4)	1 890<br>(964 to 3 000)	22.8<br>(11.6 to 36.4)	23.8<br>(15.9 to 32.7)	-1.3<br>(-7.3 to 6.1)
3	Diet high in red meat	Colon and rectum cancer	40.5<br>(9.81 to 79.5)	0.7<br>(0.2 to 1.3)	52.8<br>(13.6 to 101)	0.7<br>(0.2 to 1.3)	30.4<br>(22.2 to 46.5)	-0.1<br>(-6.3 to 12.4)	968<br>(247 to 1 850)	14.8<br>(3.7 to 28.5)	1 230<br>(333 to 2 310)	14.9<br>(4.0 to 28.0)	27.6<br>(18.5 to 44.7)	0.8<br>(-6.5 to 14.8)
3	Diet high in red meat	Breast cancer	18.7<br>(8.89 to 24.7)	0.3<br>(0.1 to 0.4)	22.5<br>(10.6 to 30.1)	0.3<br>(0.1 to 0.4)	20.4<br>(12.7 to 27.8)	-5.8<br>(-11.6 to -0.1)	556<br>(266 to 738)	8.2<br>(3.9 to 11.0)	651<br>(312 to 870)	7.8<br>(3.7 to 10.4)	17.2<br>(9.7 to 25.0)	-5.1<br>(-11.2 to 1.1)
3	Diet high in processed meat	Total cancers	29.1<br>(10.6 to 44.7)	0.5<br>(0.2 to 0.7)	33.9<br>(11.6 to 52.1)	0.4<br>(0.1 to 0.7)	16.8<br>(8.2 to 24.4)	-11.1<br>(-17.2 to -5.2)	643<br>(242 to 986)	10.0<br>(3.7 to 15.4)	735<br>(263 to 1 130)	9.0<br>(3.2 to 13.7)	14.3<br>(5.0 to 22.7)	-10.5<br>(-17.7 to -3.8)
3	Diet high in processed meat	Colon and rectum cancer	29.1<br>(10.6 to 44.7)	0.5<br>(0.2 to 0.7)	33.9<br>(11.6 to 52.1)	0.4<br>(0.1 to 0.7)	16.8<br>(8.2 to 24.4)	-11.1<br>(-17.2 to -5.2)	643<br>(242 to 986)	10.0<br>(3.7 to 15.4)	735<br>(263 to 1 130)	9.0<br>(3.2 to 13.7)	14.3<br>(5.0 to 22.7)	-10.5<br>(-17.7 to -3.8)
3	Diet low in fibre	Total cancers	17.9<br>(7.28 to 34.3)	0.3<br>(0.1 to 0.6)	20.5<br>(8.21 to 39.8)	0.3<br>(0.1 to 0.5)	14.6<br>(8.4 to 21.1)	-12.2<br>(-16.5 to -7.5)	409<br>(167 to 773)	6.3<br>(2.6 to 11.9)	449<br>(178 to 858)	5.5<br>(2.2 to 10.5)	9.8<br>(2.6 to 17.1)	-12.7<br>(-18.0 to -7.2)
3	Diet low in fibre	Colon and rectum cancer	17.9<br>(7.28 to 34.3)	0.3<br>(0.1 to 0.6)	20.5<br>(8.21 to 39.8)	0.3<br>(0.1 to 0.5)	14.6<br>(8.4 to 21.1)	-12.2<br>(-16.5 to -7.5)	409<br>(167 to 773)	6.3<br>(2.6 to 11.9)	449<br>(178 to 858)	5.5<br>(2.2 to 10.5)	9.8<br>(2.6 to 17.1)	-12.7<br>(-18.0 to -7.2)
3	Diet low in calcium	Total cancers	111<br>(79.9 to 151)	1.8<br>(1.3 to 2.5)	138<br>(96.8 to 189)	1.7<br>(1.2 to 2.4)	23.8<br>(15.3 to 32.1)	-4.5<br>(-10.7 to 1.7)	2 620<br>(1 920 to 3 510)	40.2<br>(29.1 to 53.9)	3 140<br>(2 250 to 4 260)	38.2<br>(27.2 to 51.8)	19.8<br>(10.7 to 28.8)	-5.0<br>(-11.8 to 1.9)
3	Diet low in calcium	Colon and rectum cancer	111<br>(79.9 to 151)	1.8<br>(1.3 to 2.5)	138<br>(96.8 to 189)	1.7<br>(1.2 to 2.4)	23.8<br>(15.3 to 32.1)	-4.5<br>(-10.7 to 1.7)	2 620<br>(1 920 to 3 510)	40.2<br>(29.1 to 53.9)	3 140<br>(2 250 to 4 260)	38.2<br>(27.2 to 51.8)	19.8<br>(10.7 to 28.8)	-5.0<br>(-11.8 to 1.9)
3	Diet high in sodium	Total cancers	72.3<br>(2.04 to 285)	1.2<br>(0.0 to 4.5)	74.1<br>(2.12 to 295)	0.9<br>(0.0 to 3.6)	2.4<br>(-8.2 to 13.0)	-20.3<br>(-28.4 to -12.2)	1 770<br>(48.9 to 6 950)	26.8<br>(0.7 to 105.5)	1 740<br>(48.7 to 6 800)	20.9<br>(0.6 to 82.1)	-1.9<br>(-12.7 to 8.9)	-22.0<br>(-30.6 to -13.4)
3	Diet high in sodium	Stomach cancer	72.3<br>(2.04 to 285)	1.2<br>(0.0 to 4.5)	74.1<br>(2.12 to 295)	0.9<br>(0.0 to 3.6)	2.4<br>(-8.2 to 13.0)	-20.3<br>(-28.4 to -12.2)	1 770<br>(48.9 to 6 950)	26.8<br>(0.7 to 105.5)	1 740<br>(48.7 to 6 800)	20.9<br>(0.6 to 82.1)	-1.9<br>(-12.7 to 8.9)	-22.0<br>(-30.6 to -13.4)
2	Unsafe sex	Total cancers	238<br>(211 to 269)	3.6<br>(3.2 to 4.1)	280<br>(239 to 314)	3.4<br>(2.9 to 3.8)	17.9<br>(8.5 to 28.6)	-5.2<br>(-12.7 to 3.2)	7 820<br>(6 850 to 8 680)	112.6<br>(99.2 to 125.4)	8 960<br>(7 550 to 9 980)	107.2<br>(90.5 to 119.4)	14.6<br>(5.0 to 25.0)	-4.8<br>(-12.7 to 3.7)
2	Unsafe sex	Cervical cancer	238<br>(211 to 269)	3.6<br>(3.2 to 4.1)	280<br>(239 to 314)	3.4<br>(2.9 to 3.8)	17.9<br>(8.5 to 28.6)	-5.2<br>(-12.7 to 3.2)	7 820<br>(6 850 to 8 680)	112.6<br>(99.2 to 125.4)	8 960<br>(7 550 to 9 980)	107.2<br>(90.5 to 119.4)	14.6<br>(5.0 to 25.0)	-4.8<br>(-12.7 to 3.7)
2	Low physical activity	Total cancers	52.9<br>(20.1 to 94.5)	0.9<br>(0.4 to 1.6)	67.1<br>(25.8 to 122)	0.9<br>(0.3 to 1.6)	26.8<br>(19.7 to 33.5)	-5.1<br>(-10.5 to -0.1)	966<br>(370 to 1 720)	15.6<br>(6.0 to 27.9)	1 200<br>(455 to 2 160)	15.0<br>(5.7 to 26.9)	24.5<br>(17.1 to 31.7)	-4.2<br>(-9.9 to 1.3)
2	Low physical activity	Colon and rectum cancer	46.0<br>(12.7 to 87.1)	0.8<br>(0.2 to 1.5)	58.7<br>(16.9 to 112)	0.8<br>(0.2 to 1.5)	27.5<br>(20.3 to 35.0)	-4.8<br>(-10.3 to 0.6)	801<br>(214 to 1 550)	13.1<br>(3.5 to 25.3)	1 000<br>(262 to 1 940)	12.6<br>(3.4 to 24.2)	25.4<br>(17.9 to 33.1)	-4.1<br>(-9.8 to 1.7)
2	Low physical activity	Breast cancer	6.92<br>(3.26 to 11.6)	0.1<br>(0.1 to 0.2)	8.48<br>(4.08 to 14.3)	0.1<br>(0.1 to 0.2)	22.5<br>(15.3 to 30.1)	-6.9<br>(-12.6 to -0.6)	164<br>(81.3 to 283)	2.5<br>(1.2 to 4.3)	198<br>(97.5 to 345)	2.4<br>(1.2 to 4.2)	20.4<br>(13.0 to 28.3)	-5.0<br>(-11.0 to 1.8)
**1**	**Metabolic risks**	**Total cancers**	**643<br>(320 to 1 050)**	**10.4<br>(5.2 to 17.2)**	**865<br>(448 to 1 410)**	**10.7<br>(5.5 to 17.5)**	**34.7<br>(27.9 to 42.8)**	**2.8<br>(-2.2 to 8.8)**	**14 600<br>(7 440 to 23 500)**	**225.5<br>(115.3 to 364.8)**	**19 400<br>(10 300 to 31 100)**	**234.0<br>(124.0 to 376.0)**	**33.3<br>(25.8 to 42.0)**	**3.8<br>(-2.0 to 10.5)**
2	High fasting plasma glucose	Total cancers	312<br>(86.1 to 632)	5.2<br>(1.4 to 10.4)	419<br>(116 to 848)	5.3<br>(1.5 to 10.6)	34.2<br>(27.5 to 42.2)	2.0<br>(-3.2 to 8.0)	6 430<br>(1 740 to 13 100)	101.3<br>(27.4 to 207.0)	8 580<br>(2 360 to 17 600)	104.2<br>(28.7 to 212.9)	33.4<br>(26.1 to 42.2)	2.9<br>(-2.8 to 9.5)

GBD Risk Level	Risk factor	Cancer	Both sexes combined											
			Deaths						DALYs					
			Deaths in 2010, thousands (95% UI)	Age-standardised death rates, per 100,000, in 2010 (95% UI)	Deaths in 2019, thousands (95% UI)	Age-standardised death rates, per 100,000, in 2019 (95% UI)	Percent change in absolute deaths, 2010 – 2019 (95% UI)	Percent change in age-standardised death rates, 2010 – 2019 (95% UI)	DALYs in 2010, thousands (95% UI)	Age-standardised DALY rates, per 100,000, in 2010 (95% UI)	DALYs in 2019, thousands (95% UI)	Age-standardised DALY rates, per 100,000, in 2019 (95% UI)	Percent change in absolute DALYs, 2010 – 2019 (95% UI)	Percent change in age-standardised DALY rates, 2010 – 2019 (95% UI)
2	High fasting plasma glucose	Colon and rectum cancer	71.8 (17.6 to 157)	1.2 (0.3 to 2.6)	97.6 (23.8 to 213)	1.2 (0.3 to 2.7)	35.8 (29.4 to 43.0)	2.5 (-2.2 to 7.8)	1 410 (337 to 3 090)	22.4 (5.4 to 49.2)	1 900 (454 to 4 170)	23.3 (5.6 to 51.2)	35.3 (28.3 to 43.1)	4.0 (-1.3 to 9.8)
2	High fasting plasma glucose	Liver cancer	3.25 (0.767 to 7.00)	0.1 (0.0 to 0.1)	4.73 (1.15 to 10.4)	0.1 (0.0 to 0.1)	45.7 (35.8 to 57.2)	11.7 (4.1 to 20.5)	68.9 (16.4 to 149)	1.1 (0.3 to 2.3)	99.3 (23.9 to 218)	1.2 (0.3 to 2.6)	44.2 (33.2 to 56.6)	12.2 (3.8 to 21.8)
2	High fasting plasma glucose	Pancreatic cancer	32.9 (7.78 to 71.1)	0.5 (0.1 to 1.2)	48.4 (11.5 to 104)	0.6 (0.1 to 1.3)	46.8 (39.6 to 55.0)	11.2 (5.9 to 17.2)	647 (150 to 1 410)	10.3 (2.4 to 22.3)	944 (221 to 2 040)	11.5 (2.7 to 24.7)	45.8 (37.9 to 54.8)	12.0 (6.1 to 18.6)
2	High fasting plasma glucose	Tracheal, bronchus, and lung cancer	139 (32.3 to 304)	2.3 (0.5 to 4.9)	179 (42.7 to 389)	2.2 (0.5 to 4.8)	28.4 (20.0 to 38.1)	-2.0 (-8.3 to 5.1)	2 860 (647 to 6 270)	45.0 (10.2 to 98.3)	3 640 (856 to 8 010)	44.1 (10.4 to 96.8)	27.1 (18.4 to 37.5)	-2.1 (-8.9 to 5.7)
2	High fasting plasma glucose	Breast cancer	36.9 (7.15 to 81.1)	0.6 (0.1 to 1.3)	51.1 (9.90 to 114)	0.6 (0.1 to 1.4)	38.5 (30.6 to 47.6)	5.3 (-0.5 to 12.1)	894 (170 to 1 980)	13.8 (2.6 to 30.6)	1 240 (238 to 2 790)	14.9 (2.9 to 33.5)	38.7 (29.8 to 49.0)	8.4 (1.4 to 16.3)
2	High fasting plasma glucose	Ovarian cancer	11.1 (2.22 to 25.8)	0.2 (0.0 to 0.4)	15.7 (3.02 to 36.2)	0.2 (0.0 to 0.4)	41.8 (31.3 to 52.1)	7.7 (0.0 to 15.4)	247 (49.4 to 576)	3.8 (0.8 to 8.9)	354 (68.5 to 824)	4.3 (0.8 to 9.9)	43.4 (32.1 to 55.0)	11.1 (2.0 to 19.8)
2	High fasting plasma glucose	Bladder cancer	17.0 (3.53 to 36.6)	0.3 (0.1 to 0.6)	22.8 (4.69 to 49.0)	0.3 (0.1 to 0.6)	34.4 (28.0 to 42.2)	0.8 (-3.9 to 6.5)	302 (62.1 to 652)	4.9 (1.0 to 10.6)	400 (81.6 to 866)	5.0 (1.0 to 10.7)	32.4 (25.3 to 40.7)	1.0 (-4.4 to 7.2)
2	High body-mass index	Total cancers	341 (188 to 536)	5.5 (3.0 to 8.6)	463 (261 to 718)	5.7 (3.2 to 8.8)	35.6 (27.8 to 45.2)	4.0 (-1.7 to 11.0)	8 360 (4 680 to 13 100)	127.9 (71.4 to 200.3)	11 200 (6 360 to 17 300)	133.9 (76.2 to 206.8)	33.7 (25.1 to 44.5)	4.8 (-1.8 to 12.9)
2	High body-mass index	Oesophageal cancer	69.6 (20.1 to 137)	1.1 (0.3 to 2.2)	89.9 (27.9 to 171)	1.1 (0.3 to 2.1)	29.2 (15.4 to 48.0)	0.0 (-10.5 to 14.5)	1 750 (512 to 3 480)	26.5 (7.7 to 52.6)	2 200 (682 to 4 170)	26.3 (8.1 to 49.9)	25.6 (11.3 to 45.3)	-1.0 (-12.2 to 14.5)
2	High body-mass index	Colon and rectum cancer	62.9 (33.2 to 101)	1.0 (0.5 to 1.6)	85.9 (46.8 to 137)	1.1 (0.6 to 1.7)	36.6 (29.5 to 44.0)	4.7 (-0.4 to 10.3)	1 500 (803 to 2 400)	23.0 (12.3 to 36.7)	2 020 (1 120 to 3 180)	24.4 (13.5 to 38.5)	35.1 (27.3 to 43.4)	6.3 (0.4 to 12.7)
2	High body-mass index	Liver cancer	42.3 (16.3 to 83.2)	0.7 (0.3 to 1.3)	60.8 (24.2 to 115)	0.7 (0.3 to 1.4)	43.6 (30.7 to 59.6)	12.2 (2.4 to 24.3)	1 140 (435 to 2 230)	17.0 (6.5 to 33.1)	1 610 (629 to 3 050)	19.2 (7.6 to 36.4)	41.3 (26.7 to 58.7)	13.0 (1.5 to 26.5)
2	High body-mass index	Gallbladder and biliary tract cancer	20.1 (10.2 to 33.4)	0.3 (0.2 to 0.6)	26.1 (13.9 to 42.6)	0.3 (0.2 to 0.5)	29.7 (21.7 to 40.4)	-1.3 (-7.3 to 6.8)	441 (225 to 725)	6.8 (3.5 to 11.2)	568 (306 to 923)	6.9 (3.7 to 11.2)	28.7 (20.0 to 40.2)	0.3 (-6.4 to 9.0)
2	High body-mass index	Pancreatic cancer	22.1 (8.14 to 41.8)	0.4 (0.1 to 0.7)	31.9 (12.0 to 59.7)	0.4 (0.1 to 0.7)	44.5 (38.0 to 51.7)	10.3 (5.1 to 15.8)	497 (176 to 960)	7.7 (2.8 to 14.8)	709 (256 to 1 330)	8.5 (3.1 to 16.0)	42.7 (35.4 to 50.5)	11.3 (5.6 to 17.2)
2	High body-mass index	Breast cancer	30.9 (11.7 to 56.8)	0.5 (0.2 to 0.9)	45.2 (18.8 to 81.2)	0.6 (0.2 to 1.0)	46.3 (33.4 to 70.3)	7.1 (-2.5 to 22.3)	629 (179 to 1 230)	10.4 (3.2 to 19.8)	958 (306 to 1 820)	11.2 (3.5 to 21.4)	52.4 (35.4 to 99.0)	8.2 (-4.8 to 28.9)
2	High body-mass index	Uterine cancer	28.6 (19.0 to 39.1)	0.5 (0.3 to 0.6)	36.5 (25.1 to 49.2)	0.4 (0.3 to 0.6)	27.4 (20.1 to 36.8)	-2.5 (-7.7 to 4.3)	745 (494 to 1 020)	11.4 (7.5 to 15.5)	936 (643 to 1 260)	11.2 (7.7 to 15.0)	25.6 (17.4 to 36.4)	-1.4 (-7.8 to 6.7)
2	High body-mass index	Ovarian cancer	4.70 (-0.143 to 10.8)	0.1 (0.0 to 0.2)	6.31 (-0.177 to 14.3)	0.1 (0.0 to 0.2)	34.1 (24.4 to 42.2)	3.9 (-3.6 to 10.0)	125 (-3.83 to 287)	1.9 (-0.1 to 4.3)	168 (-4.67 to 380)	2.0 (-0.1 to 4.5)	33.8 (23.6 to 43.0)	6.6 (-1.7 to 13.8)
2	High body-mass index	Kidney cancer	23.8 (13.7 to 35.9)	0.4 (0.2 to 0.6)	31.7 (18.4 to 47.3)	0.4 (0.2 to 0.6)	33.0 (27.2 to 39.5)	2.2 (-2.3 to 7.2)	575 (334 to 865)	8.8 (5.1 to 13.2)	752 (444 to 1 110)	9.0 (5.3 to 13.4)	30.7 (24.2 to 37.8)	2.8 (-2.2 to 8.3)
2	High body-mass index	Thyroid cancer	3.41 (1.62 to 5.80)	0.1 (0.0 to 0.1)	4.66 (2.29 to 7.89)	0.1 (0.0 to 0.1)	36.8 (29.0 to 46.3)	6.1 (0.0 to 13.4)	94.8 (45.4 to 161)	1.4 (0.7 to 2.4)	128 (63.8 to 214)	1.5 (0.8 to 2.6)	34.5 (26.4 to 45.0)	7.8 (1.3 to 16.0)
2	High body-mass index	Non-Hodgkin lymphoma	10.1 (4.20 to 18.4)	0.2 (0.1 to 0.3)	13.8 (5.81 to 24.5)	0.2 (0.1 to 0.3)	36.6 (30.6 to 43.6)	5.7 (1.3 to 11.7)	264 (110 to 483)	4.0 (1.7 to 7.3)	356 (152 to 633)	4.3 (1.8 to 7.7)	34.6 (28.0 to 42.8)	8.0 (2.8 to 14.5)
2	High body-mass index	Multiple myeloma	5.84 (2.52 to 10.4)	0.1 (0.0 to 0.2)	8.02 (3.52 to 14.2)	0.1 (0.0 to 0.2)	37.3 (30.8 to 44.5)	4.6 (-0.2 to 10.4)	132 (58.2 to 236)	2.1 (0.9 to 3.7)	180 (80.3 to 318)	2.2 (1.0 to 3.8)	35.9 (28.5 to 44.0)	5.7 (0.1 to 12.1)
2	High body-mass index	Leukaemia	16.8 (7.78 to 29.0)	0.3 (0.1 to 0.5)	21.7 (10.5 to 37.0)	0.3 (0.1 to 0.5)	29.7 (23.5 to 37.9)	1.4 (-3.3 to 7.5)	463 (212 to 806)	6.9 (3.2 to 12.0)	584 (288 to 993)	7.1 (3.5 to 12.1)	26.1 (18.5 to 35.6)	2.9 (-3.0 to 10.4)

Results are for both sexes combined. The number on the left of each risk factor indicates its level in the GBD hierarchy; for more information on risk factor levels in the GBD hierarchy see Appendix table 9 (p152–153). DALYs = disability-adjusted life-years; ASRs = age-standardised rates.

Appendix Table 34: Comparison of individual country studies and GBD 2019 study population-attributable fraction estimates

Country (ref)*	Individual study		GBD 2019 study	
	Risks	PAF (%)	Risks	GBD estimates for country and year comparable to individual study (% and 95% UI)
Australia, 2010 (Whiteman et al., 2015)[1]	Risk-attributable incident cancer cases, both sexes		Risk-attributable cancer deaths, both sexes	
	All included risks	31.8	All included risks	42.8 (40.0, 46.0)
	Tobacco smoke	13.4	Smoking	20.2 (19.2, 21.3)
	Alcohol	2.8	Alcohol use	7.0 (6.2, 7.7)
	Overweight and obesity	3.4	High body-mass index	6.3 (3.9, 9.0)
	Insufficient physical activity	1.6	Low physical activity	1.3 (0.5, 2.2)
	Diet - insufficient fiber	2.2	Diet low in fiber	0.3 (0.1, 0.6)
	Diet - red and processed meat	2.2	Diet high in red meat	1.8 (1.0, 2.5)
			Diet high in processed meat	0.7 (0.2, 1.0)
	Diet - insufficient vegetables	0.3	Diet low in vegetables	0.2 (0.0, 0.4)
	Diet - insufficient fruit	1.3	Diet low in fruits	1.1 (0.5, 1.9)
Canada, 2015 (Poirier et al., 2019)[2]	Risk-attributable incident cancer cases, both sexes		Risk-attributable cancer deaths, both sexes	
	All included risks	33.3	All included risks	47.4 (44.4, 50.7)
	Active tobacco smoking	17.5	Smoking	28.9 (27.4, 30.4)
	Passive tobacco smoking	0.8	Secondhand smoke	0.9 (0.6, 1.4)
	Physical inactivity	4.9	Low physical activity	1.0 (0.3, 1.8)
	Excess weight	3.1	High body-mass index	6.2 (3.7, 8.9)
	Alcohol consumption	1.8	Alcohol use	5.5 (4.9, 6.2)
	Low vegetable consumption	0.3	Diet low in vegetables	0.2 (0.0, 0.3)
	Low fruit consumption	0.7	Diet low in fruits	1.2 (0.4, 1.8)
	Red meat consumption	0.6	Diet high in red meat	1.0 (0.5, 1.7)
	Processed meat consumption	0.6	Diet high in processed meat	0.6 (0.2, 0.9)
	Outdoor air pollution (PM2.5)	0.9	Ambient particulate matter pollution	1.0 (0.4, 1.7)
	Residential radon	0.9	Residential radon	0.8 (0.1, 2.1)

Country (ref)*	Individual study		GBD 2019 study	
	Risks	PAF (%)	Risks	GBD estimates for country and year comparable to individual study (% and 95% UI)
China, 2005 (Wang et al., 2012)[3]	Risk-attributable cancer deaths, both sexes		Risk-attributable cancer deaths, both sexes	
	All included risks	57.4	All included risks	48.3 (44.7, 53.9)
	Tobacco smoking	22.6	Smoking	31.8 (30.0, 33.7)
	Low fruit intake	13	Diet low in fruits	2.7 (1.3, 4.9)
	Alcohol drinking	4.4	Alcohol	4.8 (3.8, 5.8)
	Low vegetable intake	3.6	Diet low in vegetables	0.2 (0.0, 0.4)
	Overweight and obesity	0.3	High body-mass index	2.8 (0.9, 5.8)
	Physical inactivity	0.3	Low physical activity	0.3 (0.1, 0.6)
	Occupational agents (asbestos, silica, coke oven emissions, chromate, benzene, benzidine, arsenic, wood dust, leather dust, rubber industry)	2.7	Occupational carcinogens (occupational exposure to asbestos, arsenic, benzene, beryllium, cadmium, chromium, diesel engine exhaust, formaldehyde, nickel, polycyclic aromatic hydrocarbons, silica, sulfuric acid, trichloroethylene)	2.0 (1.5, 2.7)
	Environmental agents (indoor radon, passive smoking)	0.7	Residential radon	0.9 (0.2, 1.8)
			Secondhand smoke	1.9 (1.2, 2.7)
France, 2000 (Boffetta et al., 2009)[4]	Risk-attributable cancer deaths, both sexes		Risk-attributable cancer deaths, both sexes	
	All included risks	35	All included risks	46.8 (44.3, 49.6)
	Tobacco smoking	23.9	Smoking	27.5 (26.0, 29.1)
	Alcohol drinking	6.9	Alcohol use	9.2 (8.3, 10.1)
	Occupation (asbestos, silica, polycyclic aromatic hydrocarbons, chromium VI, nickel, cadmium, aromatic amines, benzene, radon, wood dust, leather dust, painters, rubber industry)	2.4	Occupational carcinogens (occupational exposure to asbestos, arsenic, benzene, beryllium, cadmium, chromium, diesel engine exhaust, formaldehyde, nickel, polycyclic aromatic hydrocarbons, silica, sulfuric acid, trichloroethylene)	7.0 (5.4, 8.4)
	Overweight and obesity	1.6	High body-mass index	4.7 (2.6, 7.2)
	Lack of physical activity	1.6	Low physical activity	1.5 (0.6, 2.4)
	Pollutants	0.2	Secondhand smoke	0.9 (0.6, 1.4)

Country (ref)*	Individual study: Risks	Individual study: PAF (%)	GBD 2019 study: Risks	GBD 2019 study: GBD estimates for country and year comparable to individual study (% and 95% UI)
	(involuntary smoking from spouse, involuntary smoking at workplace, residential asbestos)			
Japan, 2005 (Inoue et al., 2012)[5]	Risk-attributable cancer deaths, both sexes		Risk-attributable cancer deaths, both sexes	
	All included risks	46.2	All included risks	44.5 (41.1, 49.3)
	Tobacco smoking	23.2	Smoking	29.1 (27.4, 30.8)
	Alcohol drinking	6.2	Alcohol use	4.9 (4.3, 5.5)
	Salt intake	1.4	Diet high in sodium	1.4 (0.0, 5.1)
	Body-mass index	0.8	High body-mass index	2.8 (1.0, 5.5)
	Fruit intake	0.8	Diet low in fruits	1.4 (0.7, 2.1)
	Vegetable intake	0.6	Diet low in vegetables	0.1 (0.0, 0.2)
	Physical inactivity	0.3	Low physical activity	0.9 (0.2, 1.8)
	Passive smoking	0.9	Secondhand smoke	0.9 (0.5, 1.3)
UK, 2010 (Parkin et al., 2011)[6]	Risk-attributable incident cancer cases, both sexes		Risk-attributable cancer deaths, both sexes	
	All included risks	42.7	All included risks	50.6 (47.4, 54.1)
	Tobacco smoke	19.4	Smoking	30.5 (29.1, 32.0)
	Alcohol consumption	4	Alcohol use	6.0 (5.3, 6.8)
	Deficit in intake of fruits and vegetables	4.7	Diet low in fruits	1.7 (0.9, 2.6)
			Diet low in vegetables	0.3 (0.0, 0.7)
	Red and preserved meat	2.7	Diet high in red meat	0.8 (0.4, 1.5)
			Diet high in processed meat	0.8 (0.3, 1.3)
	Deficit in intake of dietary fiber	1.5	Diet low in fiber	0.3 (0.1, 0.6)
	Excess intake of salt	0.5	Diet high in sodium	0.23 (0.0, 1.1)
	Overweight and obesity	5.5	High body-mass index	6.2 (3.6, 9.3)
	Physical exercise	1	Low physical activity	1.4 (0.6, 2.3)
	Occupational exposures	3.7	Occupational carcinogens	10.0 (8.2, 11.5)

Country (ref)*	Individual study		GBD 2019 study	
	Risks	PAF (%)	Risks	GBD estimates for country and year comparable to individual study (% and 95% UI)
			(including occupational exposure to asbestos, arsenic, benzene, beryllium, cadmium, chromium, diesel engine exhaust, formaldehyde, nickel, polycyclic aromatic hydrocarbons, silica, sulfuric acid, trichloroethylene)	
UK, 2015 (Brown et al., 2018)[7]	Risk-attributable incident cancer cases, both sexes		Risk-attributable cancer deaths, both sexes	
	All included risks	37.7	All included risks	49.8 (46.4, 53.6)
	Tobacco smoking	15.1	Smoking	28.6 (27.2, 30.2)
	Overweight and obesity	6.3	High body-mass index	6.5 (3.7, 9.6)
	Occupation	3.8	Occupational carcinogens<br>(including occupational exposure to asbestos, arsenic, benzene, beryllium, cadmium, chromium, diesel engine exhaust, formaldehyde, nickel, polycyclic aromatic hydrocarbons, silica, sulfuric acid, trichloroethylene)	9.8 (8.1, 11.2)
	Alcohol	3.3	Alcohol use	6.0 (5.2, 6.7)
	Insufficient fiber	3.3	Diet low in fiber	0.3 (0.1, 0.6)
	Processed meat	1.5	Diet high in processed meat	0.8 (0.3, 1.3)
	Air pollution	1	Air pollution	1.4 (0.9, 2.2)
	Insufficient physical activity	0.5	Low physical activity	1.4 (0.6, 2.3)
USA, 2014 (Islami et al., 2018)[8]	Risk-attributable cancer deaths, both sexes		Risk-attributable cancer deaths, both sexes	
	All included risks	45.1	All included risks	49.8 (46.3, 53.6)
	Cigarette smoking	28.8	Smoking	31.0 (29.5, 32.5)
	Excess body weight	6.5	High body-mass index	7.0 (4.4, 9.8)
	Alcohol intake	4.0	Alcohol use	4.3 (3.9, 4.7)
	Physical inactivity	2.2	Low physical activity	0.7 (0.3, 1.2)
	Low fruit/vegetable consumption	2.7	Diet low in fruits	1.2 (0.5, 1.9)
			Diet low in vegetables	0.2 (0.0, 0.3)

Country (ref)*	Individual study		GBD 2019 study	
	Risks	PAF	Risks	GBD estimates for country and year comparable to individual study
		(%)		(% and 95% UI)
	Low dietary fiber consumption	0.9	Diet low in fiber	0.3 (0.1, 0.5)
	Processed meat consumption	0.8	Diet high in processed meat	0.8 (0.3, 1.2)
	Red meat consumption	0.5	Diet high in red meat	1.1 (0.6, 1.7)
	Low dietary calcium consumption	0.5	Diet low in calcium	0.6 (0.3, 1.0)
	Secondhand smoke	0.7	Secondhand smoke	1.0 (0.6, 1.4)

* superscripts denote reference citations, which can be found on page 255. PAF = population-attributable fraction; GBD = Global Burden of Disease Study; MET = metabolic equivalent of task; $PM_{2.5}$ = particulate matter ≤ 2.5 micrometres.

References

For Appendix table 34 included on pages 254-258: ***Comparison of individual country studies and GBD 2019 study population-attributable fraction estimates***

1. Whiteman DC, Webb PM, Green AC, *et al.* Cancers in Australia in 2010 attributable to modifiable factors: summary and conclusions. *Aust N Z J Public Health.* 2015; **39**: 477–84.

2. Poirier AE, Ruan Y, Volesky KD, *et al.* The current and future burden of cancer attributable to modifiable risk factors in Canada: Summary of results. *Prev Med.* 2019; **122**: 140–7.

3. Wang JB, Jiang Y, Liang H, *et al.* Attributable causes of cancer in China. *Annals of Oncology.* 2012; **23**: 2983–9.

4. Boffetta P, Tubiana M, Hill C, *et al.* The causes of cancer in France. *Annals of Oncology.* 2009; **20**: 550–5.

5. Inoue M, Sawada N, Matsuda T, *et al.* Attributable causes of cancer in Japan in 2005—systematic assessment to estimate current burden of cancer attributable to known preventable risk factors in Japan. *Annals of Oncology.* 2012; **23**: 1362–9.

6. Parkin DM, Boyd L, Walker LC. 16. The fraction of cancer attributable to lifestyle and environmental factors in the UK in 2010. *Br J Cancer.* 2011; **105**: S77–81.

7. Brown KF, Rumgay H, Dunlop C, *et al.* The fraction of cancer attributable to modifiable risk factors in England, Wales, Scotland, Northern Ireland, and the United Kingdom in 2015. *Br J Cancer* 2018; **118**: 1130–41.

8. Islami F, Goding Sauer A, Miller KD, *et al.* Proportion and number of cancer cases and deaths attributable to potentially modifiable risk factors in the United States. *CA: A Cancer Journal for Clinicians.* 2018; **68**: 31–54.

Author Contributions

Managing the overall research enterprise
Kelly Compton, Lisa M Force, Simon I Hay, Christopher J L Murray, and Theo Vos.

Writing the first draft of the manuscript
Kelly Compton, Lisa M Force, Simon I Hay, Justin J Lang, Khanh Bao Tran, and Theo Vos.

Primary responsibility for applying analytical methods to produce estimates
Lisa M Force, Simon I Hay, Jonathan M Kocarnik, Christopher J L Murray, Theo Vos, and Rixing Xu.

Primary responsibility for seeking, cataloguing, extracting, or cleaning data; designing or coding figures and tables
Alistair R Acheson, Hannah Jacqueline Henrikson, Louise Penberthy, and Rixing Xu.

Providing data or critical feedback on data sources
Qamar Abbas, Hedayat Abbastabar, Hassan Abidi, Hassan Abolhassani, Hiwa Abubaker Ali, Isaac Yeboah Addo, Qorinah Estiningtyas Sakilah Adnani, Shailesh M Advani, Muhammad Sohail Afzal, Bright Opoku Ahinkorah, Rizwan Ahmad, Sajjad Ahmad, Sohail Ahmad, Sepideh Ahmadi, Haroon Ahmed, Muktar Beshir Ahmed, Tarik Ahmed Rashid, Mostafa Akbarzadeh-Khiavi, Addis Aklilu, Chisom Joyqueenet Akunna, Hanadi Al Hamad, Fares Alahdab, Fahad Mashhour Alanezi, Turki M Alanzi, Beriwan Abdulqadir Ali, Liaqat Ali, Syed Shujait Ali, Motasem Alkhayyat, Sami Almustanyir, Ala'a B Al-Tammemi, Haya Altawalah, Nelson Alvis-Guzman, Hubert Amu, Jason A Anderson, Mustafa Geleto Ansha, Maxwell Hubert Antwi, Sumadi Lukman Anwar, Jalal Arabloo, Olatunde Aremu, Ayele Mamo Argaw, Timur Aripov, Al Artaman, Judie Arulappan, Mulusew A Asemahagn, Zatollah Asemi, Tahira Ashraf, Seyyed Shamsadin Athari, Marcel Ausloos, Beatriz Paulina Ayala Quintanilla, Beatriz Paulina Ayala Quintanilla, Solomon Shitu Ayen, Saber Azami-Aghdash, Melkalem Mamuye Azanaw, Ahmed Y Y Azzam, Ashish D Badiye, Sara Bagherieh, Saeed Bahadory, Atif Amin Baig, Ahad Bakhtiari, Awraris Hailu Balchut/Bilchut, Maciej Banach, Indrajit Banerjee, Mainak Bardhan, Fabio Barra, Amadou Barrow, Abdul-Monim Mohammad Batiha, Alehegn Bekele Bekele, Alemayehu Sayih Belay, Amiel Nazer C Bermudez, Eduardo Bernabe, Akshaya Srikanth Bhagavathula, Neeraj Bhala, Soumitra S Bhuyan, Sadia Bibi, Bagas Suryo Bintoro, Belay Boda Abule Bodicha, Srinivasa Rao Bolla, Archith Boloor, Dejana Braithwaite, Michael Brauer, Muhammad Hammad Butt Butt, Luis Alberto Cámera, Francieli Cembranel, Promit Ananyo Chakraborty, Periklis Charalampous, Vijay Kumar Chattu, Jesus Lorenzo Chirinos-Caceres, Daniel Youngwhan Cho, William C S Cho, Devasahayam J Christopher, Dinh-Toi Chu, Aaron J Cohen, Natália Cruz-Martins, Xiaochen Dai, Lalit Dandona, Rakhi Dandona, Parnaz Daneshpajouhnejad, Aso Mohammad Darwesh, Saswati Das, Fitsum Wolde Demisse, Meseret Derbew Molla, Rupak Desai, Fikadu Nugusu Dessalegn, Samath Dhamminda Dharmaratne, Meghnath Dhimal, Mostafa Dianatinasab, Saeid Doaei, Linh Phuong Doan, Wendel Mombaque dos Santos, Haneil Larson Dsouza, Sareh Edalati, Fatemeh Eghbalian, Ebrahim Eini, Michael Ekholuenetale, Mohammad Hassan Emamian, Luchuo Engelbert Bain, Ryenchindorj Erkhembayar, Tahir Eyayu, Sayeh Ezzikouri, Adeniyi Francis Fagbamigbe, Ildar Ravisovich Fakhradiyev, Jawad Fares, Hossein Farrokhpour, Ali Fatehizadeh, Berhanu Elfu Feleke, Simone Ferrero, Lorenzo Ferro Desideri, Irina Filip, Roham Foroumadi, Takeshi Fukumoto, Peter Andras Gaal, Mohamed M Gad, Muktar A Gadanya, Tushar Garg, Teferi Gebru Gebremeskel, Belete Negese Belete Gemeda, Kazem Ghaffari, Mansour Ghafourifard, Ajnish Ghimire, Sherief Ghozy, Sherief Ghozy, Abraham Tamirat T Gizaw, James C Glasbey, Justyna Godos, Amit Goel, Mahaveer Golechha, Pouya Goleij, Mohamad Golitaleb, Bárbara Niegia Garcia Goulart, Giuseppe Grosso, Sapna Gupta, Veer Bala Gupta, Vivek Kumar Gupta, Alemu Guta, Atlas Haddadi Avval, Nima Hafezi-Nejad, Adel Hajj Ali, Arvin Haj-Mirzaian, Aram Halimi, Rabih Halwani,

Netanja I Harlianto, Josep Maria Haro, Ahmed I Hasaballah, Hamidreza Hasani, Soheil Hassanipour, Hannah Jacqueline Henrikson, Claudiu Herteliu, Kamal Hezam, Mbuzeleni Mbuzeleni Hlongwa, Nobuyuki Horita, Mehdi Hosseinzadeh, Mowafa Househ, Salman Hussain, Nawfal R Hussein, Segun Emmanuel Ibitoye, Pulwasha Maria Iftikhar, Kaire Innos, Pooya Iranpour, Nahlah Elkudssiah Ismail, Gaetano Isola, Linda Merin J, Mihajlo Jakovljevic, Mahsa Jalili, Shahram Jalilian, Tahereh Javaheri, Shubha Jayaram, Bedru Jemal, Jost B Jonas, Tamas Joo, Farahnaz Joukar, Jacek Jerzy Jozwiak, Mikk Jürisson, Vidya Kadashetti, Pradnya Vishal Kakodkar, Laleh R Kalankesh, Leila R Kalankesh, Farin Kamangar, Himal Kandel, Neeti Kapoor, André Karch, Bekalu Getnet Kassa, Joonas H Kauppila, Harkiran Kaur, Maryam Keramati, Mohammad Keykhaei, Himanshu Khajuria, Abbas Khan, Md Nuruzzaman Khan, Moien AB Khan, Khaled Khatab, Mahalaqua Nazli Khatib, Mohammad Amin Khazeei Tabari, Yun Jin Kim, Adnan Kisa, Sezer Kisa, Jonathan M Kocarnik, Sindhura Lakshmi Koulmane Laxminarayana, Kewal Krishan, Nuworza Kugbey, Mukhtar Kulimbet, Akshay Kumar, G Anil Kumar, Ambily Kuttikkattu, Dharmesh Kumar Lal, Judit Lám, Savita Lasrado, Caterina Ledda, Sang-woong Lee, Shaun Wen Huey Lee, Yo Han Lee, Samson Mideksa Legesse, James Leigh, Stephen S Lim, Gang Liu, Farzan Madadizadeh, Ahmad R Mafi, Ata Mahmoodpoor, Rashidul Alam Mahumud, Alaa Makki, Tauqeer Hussain Mallhi, Mohammed A Mamun, Fariborz Mansour-Ghanaei, Borhan Mansouri, Mohammad Ali Mansournia, Clara N Matei, Manu Raj Mathur, Jitendra Kumar Meena, Khalid Mehmood, Entezar Mehrabi Nasab, Addisu Melese, Walter Mendoza, Ritesh G Menezes, Tuomo J Meretoja, Belsity Temesgen Meselu, Mahboobeh Meshkat, Gelana Fekadu Worku Mijena, Seyed Kazem Mirinezhad, Hamed Mirzaei, Karzan Abdulmuhsin Mohammad, Mokhtar Mohammadi, Abdollah Mohammadian-Hafshejani, Reza Mohammadpourhodki, Shafiu Mohammed, Nagabhishek Moka, Ali H Mokdad, Mariam Molokhia, Sara Momtazmanesh, Lorenzo Monasta, Yousef Moradi, Ebrahim Mostafavi, Amin Mousavi Khaneghah, Sumaira Mubarik, Francesk Mulita, Efrén Murillo-Zamora, Christopher J L Murray, Ahamarshan Jayaraman Nagarajan, Shankar Prasad Nagaraju, Gabriele Nagel, Mohammadreza Naghipour, Tapas Sadasivan Nair, Sreenivas Narasimha Swamy, Aparna Ichalangod Narayana, Zuhair S Natto, Biswa Prakash Nayak, Wogene Wogene Negash, Kazem Nejati, Sandhya Neupane Kandel, Robina Khan Niazi, Hasti Nouraei, Chimezie Igwegbe Nzoputam, Ogochukwu Janet Nzoputam, Bogdan Oancea, Onome Bright Oghenetega, Ayodipupo Sikiru Oguntade, Andrew T Olagunju, Tinuke O Olagunju, Emad Omer, Abidemi E Emmanuel Omonisi, Sokking Ong, Obinna E Onwujekwe, Bilcha Oumer, Mahesh P A, Jagadish Rao Padubidri, Babak Pakbin, Keyvan Pakshir, Tamás Palicz, Adrian Pana, Anamika Pandey, Ashok Pandey, Shahina Pardhan, Seoyeon Park, Jay Patel, Siddhartha Pati, Louise Penberthy, Jeevan Pereira, Renato B Pereira, Simone Perna, Navaraj Perumalsamy, Richard G Pestell, Julian David Pillay, Zahra Zahid Piracha, Maarten J Postma, Akram Pourshams, Naeimeh Pourtaheri, Zahiruddin Quazi Syed, Mohammad Rabiee, Navid Rabiee, Amir Radfar, Raghu Anekal Radhakrishnan, Nasiru Raheem, Fakher Rahim, Amir Masoud Rahmani, Shayan Rahmani, Pradhum Ram, Kiana Ramezanzadeh, Juwel Rana, Chythra R Rao, Sowmya J Rao, Sina Rashedi, Amirfarzan Rashidi, David Laith Rawaf, Salman Rawaf, Lal Rawal, Reza Rawassizadeh, Andre M N Renzaho, Maryam Rezaei, Nima Rezaei, Peter Rohloff, Esperanza Romero-Rodríguez, Gholamreza Roshandel, Godfrey M Rwegerera, Basema Saddik, Mohammad Reza Saeb, Umar Saeed, Harihar Sahoo, Mirza Rizwan Sajid, Hedayat Salari, Marwa Rashad Salem, Abdallah M Samy, Juan Sanabria, Milena M Santric-Milicevic, Arash Sarveazad, Brijesh Sathian, Maheswar Satpathy, Mario Šekerija, Subramanian Senthilkumaran, Allen Seylani, Kenbon Seyoum, Feng Sha, Pritik A Shah, Masood Ali Shaikh, Mohammed Shannawaz, Javad Sharifi-Rad, Purva Sharma, Sara Sheikhbahaei, Adithi Shetty, K M Shivakumar, K M Shivakumar, Parnian Shobeiri, Sunil Shrestha, Negussie Boti Sidemo, Diego Augusto Santos Silva, Luís Manuel Lopes Rodrigues Silva, Wudneh Simegn, Achintya Dinesh Singh, Garima Singh, Jasvinder A Singh, Jitendra Kumar Singh, Samarjeet Singh Siwal, Valentin Yurievich Skryabin, Anna Aleksandrovna Skryabina, Matthew J Soeberg, Reed J D Sorensen, Sergey Soshnikov, Houman

Sotoudeh, Muhammad Suleman, Rizwan Suliankatchi Abdulkader, Miklós Szócska, Rafael Tabarés-Seisdedos, Takahiro Tabuchi, Hooman Tadbiri, Ensiyeh Taheri, Mircea Tampa, Ker-Kan Tan, Fisaha Haile Tesfay, Viveksandeep Thoguluva Chandrasekar, Nihal Thomas, Nikhil Kenny Thomas, Amir Tiyuri, Roman Topor-Madry, Mathilde Touvier, Marcos Roberto Tovani-Palone, Mai Thi Ngoc Tran, Irfan Ullah, Saif Ullah, Sana Ullah, Bhaskaran Unnikrishnan, Sahel Valadan Tahbaz, Pascual R Valdez, Shoban Babu Varthya, Sergey Konstantinovitch Vladimirov, Vasily Vlassov, Bay Vo, Theo Vos, Linh Gia Vu, Yasir Waheed, Mandaras Tariku Walde, Ronny Westerman, Melat Woldemariam, Suowen Xu, Seyed Hossein Yahyazadeh Jabbari, Naohiro Yonemoto, Mustafa Z Younis, Chuanhua Yu, Ismaeel Yunusa, Burhan Abdullah Zaman, Iman Zare, Zahra Zareshahrabadi, Mikhail Sergeevich Zastrozhin, Mohammad Zoladl.

Developing methods or computational machinery
Hiwa Abubaker Ali, Qorinah Estiningtyas Sakilah Adnani, Araz Ramazan Ahmad, Muktar Beshir Ahmed, Tarik Ahmed Rashid, Mostafa Akbarzadeh-Khiavi, Liaqat Ali, Solomon Shitu Ayen, Saber Azami-Aghdash, Hosein Azizi, Ahmed Y Y Azzam, Indrajit Banerjee, Alemayehu Sayih Belay, Akshaya Srikanth Bhagavathula, Soumitra S Bhuyan, Michael Brauer, Garland T Culbreth, Xiaochen Dai, Aso Mohammad Darwesh, Elham Davtalab Esmaeili, Fikadu Nugusu Dessalegn, Mostafa Dianatinasab, Saeid Doaei, Paul Narh Doku, Fatemeh Eghbalian, Tahir Eyayu, Ali Fatehizadeh, Berhanu Elfu Feleke, Kazem Ghaffari, Maryam Gholamalizadeh, Sherief Ghozy, Mehdi Hosseinzadeh, Mowafa Househ, Abhishek Jaiswal, Mahsa Jalili, Tahereh Javaheri, Abbas Khan, Mahalaqua Nazli Khatib, Adnan Kisa, Jonathan M Kocarnik, Sang-woong Lee, James Leigh, Rashidul Alam Mahumud, Alaa Makki, Mohammed A Mamun, Borhan Mansouri, Jitendra Kumar Meena, Khalid Mehmood, Mahboobeh Meshkat, Seyed Kazem Mirinezhad, Mokhtar Mohammadi, Ali H Mokdad, Yousef Moradi, Francesk Mulita, Christopher J L Murray, Biswa Prakash Nayak, Robina Khan Niazi, Emad Omer, Bilcha Oumer, Ashok Pandey, Shahina Pardhan, Siddhartha Pati, Zahiruddin Quazi Syed, Alireza Rafiei, Amir Masoud Rahmani, Vahid Rahmanian, Reza Rawassizadeh, Maryam Rezaei, Umar Saeed, Hedayat Salari, Abdallah M Samy, Maheswar Satpathy, Mohammed Shannawaz, Javad Sharifi-Rad, Rahim Ali Sheikhi, Negussie Boti Sidemo, Reed J D Sorensen, Ensiyeh Taheri, Roman Topor-Madry, Bay Vo, Theo Vos, Ronny Westerman, Rixing Xu, Fariba Zare, Mikhail Sergeevich Zastrozhin.

Providing critical feedback on methods or results
Amirali Aali, Qamar Abbas, Behzad Abbasi, Hedayat Abbastabar, Michael Abdelmasseh, Sherief Abd-Elsalam, Ahmed Abdelwahab Abdelwahab, Gholamreza Abdoli, Hanan Abdulkadir Abdulkadir, Aidin Abedi, Kedir Hussein Abegaz, Hassan Abidi, Richard Gyan Aboagye, Hassan Abolhassani, Abdorrahim Absalan, Yonas Derso Abtew, Hiwa Abubaker Ali, Basavaprabhu Achappa, Juan Manuel Acuna, Daniel Addison, Isaac Yeboah Addo, Oyelola A Adegboye, Miracle Ayomikun Adesina, Mohammad Adnan, Qorinah Estiningtyas Sakilah Adnani, Shailesh M Advani, Sumia Afrin, Muhammad Sohail Afzal, Manik Aggarwal, Bright Opoku Ahinkorah, Araz Ramazan Ahmad, Rizwan Ahmad, Sajjad Ahmad, Sohail Ahmad, Haroon Ahmed, Luai A Ahmed, Muktar Beshir Ahmed, Tarik Ahmed Rashid, Wajeeha Aiman, Gizachew Taddesse Akalu, Addis Aklilu, Maxwell Akonde, Chisom Joyqueenet Akunna, Hanadi Al Hamad, Fares Alahdab, Fahad Mashhour Alanezi, Turki M Alanzi, Saleh Ali Alessy, Abdelazeem M Algammal, Mohammed Khaled Al-Hanawi, Robert Kaba Alhassan, Liaqat Ali, Syed Shujait Ali, Yousef Alimohamadi, Vahid Alipour, Syed Mohamed Aljunid, Motasem Alkhayyat, Sadeq Ali Ali Al-Maweri, Sami Almustanyir, Nivaldo Alonso, Shehabaldin Alqalyoobi, Rajaa M Al-Raddadi, Rami H Hani Al-Rifai, Salman Khalifah Al-Sabah, Ala'a B Al-Tammemi, Nelson Alvis-Guzman, Edward Kwabena Ameyaw, Javad Javad Aminian Dehkordi, Hubert Amu, Ganiyu Adeniyi Amusa, Robert Ancuceanu, Yaregal Animut Animut, Amir Anoushiravani, Ali Arash Anoushirvani, Alireza Ansari-Moghaddam, Mustafa Geleto Ansha, Benny Antony, Maxwell Hubert Antwi, Razique Anwer, Anayochukwu Edward

Anyasodor, Jalal Arabloo, Morteza Arab-Zozani, Olatunde Aremu, Hany Ariffin, Timur Aripov, Al Artaman, Judie Arulappan, Raphael Taiwo Aruleba, Armin Aryannejad, Mulusew A Asemahagn, Mohammad Asghari-Jafarabadi, Tahira Ashraf, Mohammad Athar, Seyyed Shamsadin Athari, Maha Moh'd Wahbi Atout, Sameh Attia, Avinash Aujayeb, Marcel Ausloos, Leticia Avila-Burgos, Atalel Fentahun Awedew, Mamaru Ayenew Awoke, Beatriz Paulina Ayala Quintanilla, Tegegn Mulatu Ayana, Solomon Shitu Ayen, Davood Azadi, Sina Azadnajafabad, Saber Azami-Aghdash, Melkalem Mamuye Azanaw, Mohammadreza Azangou-Khyavy, Amirhossein Azari Jafari, Hosein Azizi, Ahmed Y Y Azzam, Amirhesam Babajani, Muhammad Badar, Ashish D Badiye, Nayereh Baghcheghi, Nader Bagheri, Sara Bagherieh, Atif Amin Baig, Jennifer L Baker, Ahad Bakhtiari, Ravleen Kaur Bakshi, Awraris Hailu Balchut/Bilchut, Maciej Banach, Indrajit Banerjee, Mainak Bardhan, Amadou Barrow, Nasir Z Bashir, Saurav Basu, Abdul-Monim Mohammad Batiha, Alehegn Bekele Bekele, Alemayehu Sayih Belay, Melaku Ashagrie Belete, Uzma Iqbal Belgaumi, Arielle Wilder Bell, Alemshet Yirga Berhie, Amiel Nazer C Bermudez, Eduardo Bernabe, Akshaya Srikanth Bhagavathula, Bharti Bhandari Bhandari, Nikha Bhardwaj, Pankaj Bhardwaj, Krittika Bhattacharyya, Vijayalakshmi S Bhojaraja, Soumitra S Bhuyan, Sadia Bibi, Bagas Suryo Bintoro, Mesfin Geremaw Birega Birega, Habitu Eshetu Birhan, Oleg Blyuss, Belay Boda Abule Bodicha, Archith Boloor, Dejana Braithwaite, Michael Brauer, Hermann Brenner, Christina Maree Buchanan, Norma B Bulamu, Maria Teresa Bustamante-Teixeira, Muhammad Hammad Butt Butt, Nadeem Shafique Butt, Zahid A Butt, Florentino Luciano Caetano dos Santos, Luis Alberto Cámera, Chao Cao, Francieli Cembranel, Ester Cerin, Promit Ananyo Chakraborty, Periklis Charalampous, Vijay Kumar Chattu, Odgerel Chimed-Ochir, Daniel Youngwhan Cho, William C S Cho, Devasahayam J Christopher, Dinh-Toi Chu, Isaac Sunday Chukwu, Joao Conde, Sandra Cortés, Natália Cruz-Martins, Omid Dadras, Fentaw Teshome Dagnaw, Saad M A Dahlawi, Xiaochen Dai, Lalit Dandona, Rakhi Dandona, Anna Danielewicz, An Thi Minh Dao, Reza Darvishi Cheshmeh Soltani, Aso Mohammad Darwesh, Saswati Das, Elham Davtalab Esmaeili, Fernando Pio De la Hoz, Sisay Abebe Debela, Azizallah Dehghan, Biniyam Demisse, Fitsum Wolde Demisse, Edgar Denova-Gutiérrez, Meseret Derbew Molla, Kalkidan Solomon Deribe, Rupak Desai, Markos Desalegn Desalegn, Fikadu Nugusu Dessalegn, samuel abebe A dessalegni, Abebaw Alemayehu Desta, Meghnath Dhimal, Mostafa Dianatinasab, Nancy Diao, Daniel Diaz, Lankamo Ena Digesa, Shilpi Gupta Dixit, Saeid Doaei, Linh Phuong Doan, Paul Narh Doku, Deepa Dongarwar, Wendel Mombaque dos Santos, Tim Robert Driscoll, Haneil Larson Dsouza, Oyewole Christopher Durojaiye, Sareh Edalati, Fatemeh Eghbalian, Elham Ehsani-Chimeh, Ebrahim Eini, Michael Ekholuenetale, Temitope Cyrus Ekundayo, Donatus U Ekwueme, Muhammed Elhadi, Mohammad Hassan Emamian, Luchuo Engelbert Bain, Daniel Berhanie Enyew, Tegegne Eshetu, Babak Eshrati, Sharareh Eskandarieh, Farshid Etaee, Tahir Eyayu, Ifeanyi Jude Ezeonwumelu, Adeniyi Francis Fagbamigbe, Saman Fahimi, Ildar Ravisovich Fakhradiyev, Emerito Jose A Faraon, Jawad Fares, Abbas Farmany, Umar Farooque, Hossein Farrokhpour, Abidemi Omolara Fasanmi, Ali Fatehizadeh, Wafa Fatima, Hamed Fattahi, Ginenus Fekadu, Berhanu Elfu Feleke, Allegra Allegra Ferrari, Irina Filip, Florian Fischer, Lisa M Force, Roham Foroumadi, Masoud Foroutan, Takeshi Fukumoto, Peter Andras Gaal, Mohamed M Gad, Nasrin Galehdar, Tushar Garg, Yosef Haile Gebremariam, Teferi Gebru Gebremeskel, Mathewos Alemu Gebremichael, Yohannes Fikadu Geda, Melaku Getachew, Motuma Erena Getachew, Kazem Ghaffari, Mansour Ghafourifard, Mohammad Ghasemi Nour, Fariba Ghassemi, Ajnish Ghimire, Nermin Ghith, Jamshid Gholizadeh Navashenaq, Sherief Ghozy, Syed Amir Gilani, Paramjit Singh Gill, Themba G Ginindza, Abraham Tamirat T Gizaw, James C Glasbey, Justyna Godos, Mahaveer Golechha, Davide Golinelli, Mohamad Golitaleb, Giuseppe Grosso, Habtamu Alganeh Guadie, Mohammed Ibrahim Mohialdeen Gubari, Temesgen Worku Gudayu, Maximiliano Ribeiro Guerra, Damitha Asanga Gunawardane, Bhawna Gupta, Sapna Gupta, Veer Bala Gupta, Vivek Kumar Gupta, Mekdes Kondale Gurara, Alemu Guta, Parham Habibzadeh, Atlas Haddadi Avval, Nima Hafezi-Nejad, Adel Hajj Ali, Arvin Haj-Mirzaian, Esam S Halboub, Aram Halimi, Rabih

Halwani, Randah R Hamadeh, Sajid Hameed, Samer Hamidi, Asif Hanif, Sanam Hariri, Netanja I Harlianto, Josep Maria Haro, Ahmed I Hasaballah, S M Mahmudul Hasan, Hamidreza Hasani, Seyedeh Melika Hashemi, Abbas M Hassan, Soheil Hassanipour, Simon I Hay, Khezar Hayat, Golnaz Heidari, Mohammad Heidari, Hannah Jacqueline Henrikson, Brenda Yuliana Herrera-Serna, Claudiu Herteliu, Kamal Hezam, Yuta Hiraike, Mbuzeleni Mbuzeleni Hlongwa, Ramesh Holla, Marianne Holm, Nobuyuki Horita, Mohammad Hoseini, Md Mahbub Hossain, Mohammad Bellal Hossain Hossain, Mohammad-Salar Hosseini, Ali Hosseinzadeh, Mehdi Hosseinzadeh, Mihaela Hostiuc, Mowafa Househ, Fernando N Hugo, Ayesha Humayun, Salman Hussain, Nawfal R Hussein, Bing-Fang Hwang, Segun Emmanuel Ibitoye, Pulwasha Maria Iftikhar, Kevin S Ikuta, Olayinka Stephen Ilesanmi, Irena M Ilic, Milena D Ilic, Mustapha Immurana, Kaire Innos, Lalu Muhammad Irham, Md Shariful Islam, Rakibul M Islam, Farhad Islami, Nahlah Elkudssiah Ismail, Gaetano Isola, Masao Iwagami, Linda Merin J, Abhishek Jaiswal, Mihajlo Jakovljevic, Mahsa Jalili, Elham Jamshidi, Sung-In Jang, Chinmay T Jani, Tahereh Javaheri, Umesh Umesh Jayarajah, Shubha Jayaram, Seyed Behzad Jazayeri, Rime Jebai, Bedru Jemal, Wonjeong Jeong, Ravi Prakash Jha, Har Ashish Jindal, Yetunde O John-Akinola, Jost B Jonas, Tamas Joo, Nitin Joseph, Farahnaz Joukar, Jacek Jerzy Jozwiak, Mikk Jürisson, Ali Kabir, Salah Eddine Oussama Kacimi, Vidya Kadashetti, Farima Farima Kahe, Pradnya Vishal Kakodkar, Laleh R Kalankesh, Leila R Kalankesh, Rohollah Kalhor, Vineet Kumar Kamal, Farin Kamangar, Ashwin Kamath, Tanuj Kanchan, Himal Kandel, Neeti Kapoor, Sitanshu Sekhar Kar, Shama D Karanth, Ibraheem M Karaye, André Karch, Amirali Karimi, Bekalu Getnet Kassa, Patrick DMC Katoto, Joonas H Kauppila, Harkiran Kaur, Abinet Gebremickael Kebede, Gemechu Gemechu Kejela, Maryam Keramati, Himanshu Khajuria, Abbas Khan, Abdul Aziz Khan Khan, Ejaz Ahmad Khan, Md Nuruzzaman Khan, Moien AB Khan, Javad Khanali, Khaled Khatab, Moawiah Mohammad Khatatbeh, Mahalaqua Nazli Khatib, Maryam Khayamzadeh, Hamid Reza Khayat Kashani, Mehdi Khezeli, Mahmoud Khodadost, Min Seo Kim, Yun Jin Kim, Adnan Kisa, Sezer Kisa, Miloslav Klugar, Jitka Klugarová, Jonathan M Kocarnik, Ali-Asghar Kolahi, Pavel Kolkhir, Parvaiz A Koul, Sindhura Lakshmi Koulmane Laxminarayana, Ai Koyanagi, Kewal Krishan, Yuvaraj Krishnamoorthy, Burcu Kucuk Bicer, Nuworza Kugbey, Akshay Kumar, G Anil Kumar, Narinder Kumar, Om P Kurmi, Ambily Kuttikkattu, Carlo La Vecchia, Arista Lahiri, Dharmesh Kumar Lal, Judit Lám, Qing Lan, Justin J Lang, Bagher Larijani, Savita Lasrado, Paolo Lauriola, Caterina Ledda, Sang-woong Lee, Shaun Wen Huey Lee, Wei-Chen Lee, Yeong Yeh Lee, Samson Mideksa Legesse, James Leigh, Elvynna Leong, Ming-Chieh Li, Stephen S Lim, Gang Liu, Jue Liu, Chun-Han Lo, László Lorenzovici, Mojgan Lotfi, Joana A Loureiro, Farzan Madadizadeh, Ahmad R Mafi, Sameh Magdeldin, Ata Mahmoodpoor, Morteza Mahmoudi, Marzieh Mahmoudimanesh, Rashidul Alam Mahumud, Azeem Majeed, Jamal Majidpoor, Alaa Makki, Konstantinos Christos Makris, Elaheh Malakan Rad, Mohammad-Reza Malekpour, Reza Malekzadeh, Ahmad Azam Malik, Tauqeer Hussain Mallhi, Mohammed A Mamun, Fariborz Mansour-Ghanaei, Borhan Mansouri, Mohammad Ali Mansournia, Lorenzo Giovanni Mantovani, Santi Martini, Miquel Martorell, Sahar Masoudi, Seyedeh Zahra Masoumi, Clara N Matei, Elezebeth Mathews, Manu Raj Mathur, Martin McKee, Jitendra Kumar Meena, Entezar Mehrabi Nasab, Addisu Melese, Walter Mendoza, Ritesh G Menezes, SIsay Derso Mengesha, Alexios-Fotios A Mentis, Andry Yasmid Mera Mera-Mamián, Mehari Woldemariam Merid, Amanual Getnet Mersha, Belsity Temesgen Meselu, Mahboobeh Meshkat, Tomislav Mestrovic, Junmei Miao Jonasson, Tomasz Miazgowski, Irmina Maria Michalek, Gelana Fekadu Worku Mijena, Ted R Miller, Shabir Ahmad Mir, Seyyedmohammadsadeq Mirmoeeni, Mohammad Mirza-Aghazadeh-Attari, Hamid Reza Mirzaei, Abay Sisay Misganaw, Sanjeev Misra, Esmaeil Mohammadi, Mokhtar Mohammadi, Abdollah Mohammadian-Hafshejani, Reza Mohammadpourhodki, Arif Mohammed, Shafiu Mohammed, Syam Mohan, Nagabhishek Moka, Ali H Mokdad, Alex Molassiotis, Mariam Molokhia, Kaveh Momenzadeh, Sara Momtazmanesh, Ute Mons, Ahmed Al Montasir, Fateme Montazeri, Arnulfo Montero, Mohammad Amin Moosavi, Yousef Moradi, Mostafa Moradi Sarabi, Lidia Morawska, Jakub Morze, Ebrahim

Mostafavi, Seyyed Meysam Mousavi, Amin Mousavi Khaneghah, Christine Mpundu-Kaambwa, Sumaira Mubarik, Francesk Mulita, Daniel Munblit, Sandra B Munro, Efrén Murillo-Zamora, Christopher J L Murray, Jonah Musa, Ashraf F Nabhan, Ahamarshan Jayaraman Nagarajan, Shankar Prasad Nagaraju, Mohammadreza Naghipour, Mukhammad David Naimzada, Tapas Sadasivan Nair, Atta Abbas Naqvi, Sreenivas Narasimha Swamy, Zuhair S Natto, Biswa Prakash Nayak, Rawlance Ndejjo, Seyed Aria Nejadghaderi, Sandhya Neupane Kandel, Huy Van Nguyen Nguyen, Robina Khan Niazi, Nafise Noroozi, Ali Nowroozi, Chimezie Igwegbe Nzoputam, Ogochukwu Janet Nzoputam, Bogdan Oancea, Onome Bright Oghenetega, Ropo Ebenezer Ogunsakin, Ayodipupo Sikiru Oguntade, Hassan Okati-Aliabad, Akinkunmi Paul Okekunle, Andrew T Olagunju, Tinuke O Olagunju, Isaac Iyinoluwa Olufadewa, Emad Omer, Abidemi E Emmanuel Omonisi, Obinna E Onwujekwe, Hans Orru, Stanislav S Otstavnov, Abderrahim Oulhaj, Bilcha Oumer, Oluwatomi Funbi Owopetu, Babatunji Emmanuel Oyinloye, Mahesh P A, Alicia Padron-Monedero, Jagadish Rao Padubidri, Babak Pakbin, Tamás Palicz, Adrian Pana, Anamika Pandey, Ashok Pandey, Suman Pant, Shahina Pardhan, Eun-Cheol Park, Eun-Kee Park, Seoyeon Park, Jay Patel, Rajan Paudel, Uttam Paudel, Mihaela Paun, Jeevan Pereira, Renato B Pereira, Simone Perna, Navaraj Perumalsamy, Richard G Pestell, Raffaele Pezzani, Cristiano Piccinelli, Julian David Pillay, Zahra Zahid Piracha, Tobias Pischon, Maarten J Postma, Ashkan Pourabhari Langroudi, Naeimeh Pourtaheri, Akila Prashant, Mirza Muhammad Fahd Qadir, Zahiruddin Quazi Syed, Mohammad Rabiee, Navid Rabiee, Amir Radfar, Raghu Anekal Radhakrishnan, Venkatraman Radhakrishnan, Mojtaba Raeisi, Alireza Rafiei, Fakher Rahim, Md Obaidur Rahman, Mosiur Rahman, Muhammad Aziz Rahman, Amir Masoud Rahmani, Shayan Rahmani, Vahid Rahmanian, Nazanin Rajai, Aashish Rajesh, Pradhum Ram, Kiana Ramezanzadeh, Juwel Rana, Kamal Ranabhat, Priyanga Ranasinghe, Chythra R Rao, Sowmya J Rao, Sina Rashedi, Mahsa Rashidi, Mohammad-Mahdi Rashidi, David Laith Rawaf, Salman Rawaf, Lal Rawal, Reza Rawassizadeh, Mohammad Sadegh Razeghinia, Ashfaq Ur Rehman, Inayat ur Rehman, Marissa B Reitsma, Andre M N Renzaho, Maryam Rezaei, Negar Rezaei, Nima Rezaei, Mohsen Rezaeian, Aziz Rezapour, Abanoub Riad, Reza Rikhtegar, Maria Rios-Blancas, Thomas J Roberts, Peter Rohloff, Esperanza Romero-Rodríguez, Gholamreza Roshandel, Godfrey M Rwegerera, Manjula S, Maha Mohamed Saber-Ayad, Bahar Saberzadeh-Ardestani, Siamak Sabour, Basema Saddik, Erfan Sadeghi, Umar Saeed, Azam Safary, Maryam Sahebazzamani, Harihar Sahoo, Mirza Rizwan Sajid, Hedayat Salari, Sana Salehi, Marwa Rashad Salem, Hamideh Salimzadeh, Yoseph Leonardo Samodra, Abdallah M Samy, Juan Sanabria, Senthilkumar Sankararaman, Francesco Sanmarchi, Milena M Santric-Milicevic, Muhammad Arif Nadeem Saqib, Arash Sarveazad, Fatemeh Sarvi, Brijesh Sathian, Maheswar Satpathy, Nicolas Sayegh, Ione Jayce Ceola Schneider, Michaël Schwarzinger, Mario Šekerija, Sadaf G Sepanlou, Allen Seylani, Feng Sha, Omid Shafaat, Pritik A Shah, Saeed Shahabi, Izza Shahid, Mohammad Amin Shahrbaf, Hamid R Shahsavari, Masood Ali Shaikh, Mohammed Feyisso Shaka, Elaheh Shaker, Mohammed Shannawaz, Mequannent Melaku Sharew Sharew, Javad Sharifi-Rad, Purva Sharma, Bereket Beyene Shashamo, Mahdi Sheikh, Sara Sheikhbahaei, Rahim Ali Sheikhi, Ali Sheikhy, Jeevan K Shetty, Ranjitha S Shetty, Kenji Shibuya, Hesamaddin Shirzad-Aski, K M Shivakumar, Siddharudha Shivalli, Velizar Shivarov, Parnian Shobeiri, Zahra Shokri Varniab, Seyed Afshin Shorofi, Sunil Shrestha, Migbar Mekonnen Sibhat, Sudeep K Siddappa Malleshappa, Negussie Boti Sidemo, Luís Manuel Lopes Rodrigues Silva, Guilherme Silva Julian, Nicola Silvestris, Wudneh Simegn, Achintya Dinesh Singh, Ambrish Singh, Garima Singh, Jasvinder A Singh, Jitendra Kumar Singh, Paramdeep Singh, Surjit Singh, Dhirendra Narain Sinha, Abiy H Sinke, Md Shahjahan Siraj, Freddy Sitas, Samarjeet Singh Siwal, Valentin Yurievich Skryabin, Anna Aleksandrovna Skryabina, Bogdan Socea, Matthew J Soeberg, Ahmad Sofi-Mahmudi, Yonatan Solomon, Mohammad Sadegh Soltani-Zangbar, Suhang Song, Yimeng Song, Reed J D Sorensen, Sergey Soshnikov, Houman Sotoudeh, Alieu Sowe, Mu'awiyyah Babale Sufiyan, Ryan Suk, Muhammad Suleman, Rizwan Suliankatchi Abdulkader, Saima Sultana, Daniel Sur, Miklós Szócska, Seidamir Pasha Tabaeian, Rafael Tabarés-Seisdedos, Seyyed Mohammad

Tabatabaei, Takahiro Tabuchi, Hooman Tadbiri, Ensiyeh Taheri, Majid Taheri, Moslem Taheri Soodejani, Ken Takahashi, Mircea Tampa, Ker-Kan Tan, Nathan Y Tat, Vivian Y Tat, Ahmad Tavakoli, Arash Tavakoli, Yohannes Tekalegn, Fisaha Haile Tesfay, Rekha Thapar, Aravind Thavamani, Viveksandeep Thoguluva Chandrasekar, Nihal Thomas, Nikhil Kenny Thomas, Jansje Henny Vera Ticoalu, Amir Tiyuri, Daniel Nigusse Tollosa, Roman Topor-Madry, Mathilde Touvier, Marcos Roberto Tovani-Palone, Eugenio Traini, Khanh Bao Tran, Mai Thi Ngoc Tran, Jaya Prasad Tripathy, Gebresilasea Gendisha Ukke, Irfan Ullah, Saif Ullah, Sana Ullah, Bhaskaran Unnikrishnan, Maryam Vaezi, Sahel Valadan Tahbaz, Pascual R Valdez, Constantine Vardavas, Shoban Babu Varthya, Siavash Vaziri, Diana Zuleika Velazquez, Massimiliano Veroux, Paul J Villeneuve, Francesco S Violante, Bay Vo, Theo Vos, Linh Gia Vu, Yasir Waheed, Mandaras Tariku Walde, Richard G Wamai, Cong Wang, Fang Wang, Yu Wang, Paul Ward, Abdul Waris, Ronny Westerman, Nuwan Darshana Wickramasinghe, Melat Woldemariam, Berhanu Woldu, Hong Xiao, Suowen Xu, Xiaoyue Xu, Lalit Yadav, Seyed Hossein Yahyazadeh Jabbari, Lin Yang, Fereshteh Yazdanpanah, Yigizie Yeshaw, Yazachew Yismaw, Naohiro Yonemoto, Mustafa Z Younis, Zabihollah Yousefi, Fatemeh Yousefian, Chuanhua Yu, Yong Yu, Ismaeel Yunusa, Mazyar Zahir, Nazar Zaki, Burhan Abdullah Zaman, Moein Zangiabadian, Fariba Zare, Armin Zarrintan, Mikhail Sergeevich Zastrozhin, Dongyu Zhang, Jianrong Zhang, Yunquan Zhang, Zhi-Jiang Zhang, Linghui Zhou, Sanjay Zodpey, Mohammad Zoladl.

Drafting the work or revising is critically for important intellectual content
Amirali Aali, Behzad Abbasi, Mohsen Abbasi-Kangevari, Zeinab Abbasi-Kangevari, Hedayat Abbastabar, Michael Abdelmasseh, Sherief Abd-Elsalam, Ahmed Abdelwahab Abdelwahab, Gholamreza Abdoli, Aidin Abedi, Kedir Hussein Abegaz, Hassan Abidi, Hassan Abolhassani, Eman Abu-Gharbieh, Alistair R Acheson, Juan Manuel Acuna, Daniel Addison, Isaac Yeboah Addo, Oyelola A Adegboye, Mohammad Adnan, Qorinah Estiningtyas Sakilah Adnani, Shailesh M Advani, Sumia Afrin, Muhammad Sohail Afzal, Manik Aggarwal, Bright Opoku Ahinkorah, Araz Ramazan Ahmad, Rizwan Ahmad, Sohail Ahmad, Sepideh Ahmadi, Luai A Ahmed, Muktar Beshir Ahmed, Wajeeha Aiman, Marjan Ajami, Gizachew Taddesse Akalu, Fares Alahdab, Abdelazeem M Algammal, Mohammed Khaled Al-Hanawi, Robert Kaba Alhassan, Liaqat Ali, Syed Shujait Ali, Motasem Alkhayyat, Sadeq Ali Ali Al-Maweri, Sami Almustanyir, Nivaldo Alonso, Rajaa M Al-Raddadi, Rami H Hani Al-Rifai, Salman Khalifah Al-Sabah, Ala'a B Al-Tammemi, Haya Altawalah, Nelson Alvis-Guzman, Firehiwot Amare, Mohammad Hosein Amirzade-Iranaq, Hubert Amu, Ganiyu Adeniyi Amusa, Robert Ancuceanu, Amir Anoushiravani, Ali Arash Anoushirvani, Mustafa Geleto Ansha, Sumadi Lukman Anwar, Anayochukwu Edward Anyasodor, Jalal Arabloo, Morteza Arab-Zozani, Olatunde Aremu, Hany Ariffin, Timur Aripov, Muhammad Arshad, Judie Arulappan, Raphael Taiwo Aruleba, Malke Asaad, Mulusew A Asemahagn, Reza Assadi, Maha Moh'd Wahbi Atout, Sameh Attia, Avinash Aujayeb, Marcel Ausloos, Leticia Avila-Burgos, Tewachew Awoke, Beatriz Paulina Ayala Quintanilla, Tegegn Mulatu Ayana, Solomon Shitu Ayen, Davood Azadi, Sina Azadnajafabad, Saber Azami-Aghdash, Melkalem Mamuye Azanaw, Mohammadreza Azangou-Khyavy, Amirhossein Azari Jafari, Ahmed Y Y Azzam, Amirhesam Babajani, Muhammad Badar, Ashish D Badiye, Sara Bagherieh, Atif Amin Baig, Jennifer L Baker, Awraris Hailu Balchut/Bilchut, Maciej Banach, Indrajit Banerjee, Mainak Bardhan, Francesco Barone-Adesi, Fabio Barra, Amadou Barrow, Nasir Z Bashir, Azadeh Bashiri, Saurav Basu, Aeysha Begum, Alehegn Bekele Bekele, Alemayehu Sayih Belay, Melaku Ashagrie Belete, Uzma Iqbal Belgaumi, Arielle Wilder Bell, Luis Belo, Habib Benzian, Eduardo Bernabe, Akshaya Srikanth Bhagavathula, Neeraj Bhala, Krittika Bhattacharyya, Vijayalakshmi S Bhojaraja, Sadia Bibi, Antonio Biondi, Mesfin Geremaw Birega Birega, Tone Bjørge, Belay Boda Abule Bodicha, Srinivasa Rao Bolla, Cristina Bosetti, Dejana Braithwaite, Hermann Brenner, Andrey Nikolaevich Briko, Nikolay Ivanovich Briko, Christina Maree Buchanan, Norma B Bulamu, Maria Teresa Bustamante-Teixeira, Muhammad Hammad Butt Butt, Nadeem Shafique Butt, Florentino Luciano Caetano dos Santos, Chao Cao, Yin Cao, Giulia Carreras, Márcia Carvalho, Francieli Cembranel, Ester Cerin, Vijay Kumar Chattu, Odgerel Chimed-Ochir, Daniel Youngwhan Cho, William C S Cho, Dinh-Toi Chu, Kelly Compton, Joao Conde, Sandra Cortés, Vera Marisa Costa, Natália

Cruz-Martins, Anna Danielewicz, Dragos Virgil Davitoiu, Biniyam Demisse, Fitsum Wolde Demisse, Edgar Denova-Gutiérrez, Meseret Derbew Molla, Diriba Dereje, Kalkidan Solomon Deribe, Rupak Desai, Markos Desalegn Desalegn, Fikadu Nugusu Dessalegn, Gashaw Dessie, Syed Masudur Rahman Dewan, Samath Dhamminda Dharmaratne, Meghnath Dhimal, Mostafa Dianatinasab, Daniel Diaz, Shilpi Gupta Dixit, Saeid Doaei, Linh Phuong Doan, Paul Narh Doku, Deepa Dongarwar, Wendel Mombaque dos Santos, Tim Robert Driscoll, Haneil Larson Dsouza, Oyewole Christopher Durojaiye, Sareh Edalati, Fatemeh Eghbalian, Elham Ehsani-Chimeh, Ebrahim Eini, Temitope Cyrus Ekundayo, Donatus U Ekwueme, Maha El Tantawi, Mostafa Ahmed Elbahnasawy, Iffat Elbarazi, Hesham Elghazaly, Muhammed Elhadi, Waseem El-Huneidi, Mohammad Hassan Emamian, Ryenchindorj Erkhembayar, Sharareh Eskandarieh, Juan Espinosa-Montero, Farshid Etaee, Azin Etemadimanesh, Tahir Eyayu, Sayeh Ezzikouri, Adeniyi Francis Fagbamigbe, Saman Fahimi, Emerito Jose A Faraon, Jawad Fares, Ali Fatehizadeh, Berhanu Elfu Feleke, Allegra Allegra Ferrari, Simone Ferrero, Lorenzo Ferro Desideri, Irina Filip, Florian Fischer, Lisa M Force, Masoud Foroutan, Takeshi Fukumoto, Peter Andras Gaal, Mohamed M Gad, Muktar A Gadanya, Abduzhappar Gaipov, Nasrin Galehdar, Silvano Gallus, Tushar Garg, Mariana Gaspar Fonseca, Yohannes Fikadu Geda, Yibeltal Yismaw Gela, Melaku Getachew, Motuma Erena Getachew, Kazem Ghaffari, Mansour Ghafourifard, Seyyed-Hadi Ghamari, Mohammad Ghasemi Nour, Fariba Ghassemi, Nermin Ghith, Jamshid Gholizadeh Navashenaq, Sherief Ghozy, Paramjit Singh Gill, Themba G Ginindza, Abraham Tamirat T Gizaw, James C Glasbey, Mahaveer Golechha, Davide Golinelli, Mohamad Golitaleb, Giuseppe Gorini, Giuseppe Grosso, Temesgen Worku Gudayu, Maximiliano Ribeiro Guerra, Damitha Asanga Gunawardane, Sapna Gupta, Veer Bala Gupta, Vivek Kumar Gupta, Alemu Guta, Parham Habibzadeh, Atlas Haddadi Avval, Nima Hafezi-Nejad, Adel Hajj Ali, Arvin Haj-Mirzaian, Aram Halimi, Rabih Halwani, Randah R Hamadeh, Sajid Hameed, Netanja I Harlianto, Josep Maria Haro, Risky Kusuma Hartono, Ahmed I Hasaballah, S M Mahmudul Hasan, Seyedeh Melika Hashemi, Abbas M Hassan, Simon I Hay, Golnaz Heidari, Mohammad Heidari, Zahra Heidarymeybodi, Claudiu Herteliu, Kamal Hezam, Yuta Hiraike, Ramesh Holla, Marianne Holm, Nobuyuki Horita, Md Mahbub Hossain, Mohammad Bellal Hossain Hossain, Mohammad-Salar Hosseini, Sorin Hostiuc, Mowafa Househ, Junjie Huang, Fernando N Hugo, Ayesha Humayun, Salman Hussain, Segun Emmanuel Ibitoye, Pulwasha Maria Iftikhar, Olayinka Stephen Ilesanmi, Irena M Ilic, Milena D Ilic, Mustapha Immurana, Kaire Innos, Lalu Muhammad Irham, Rakibul M Islam, Farhad Islami, Nahlah Elkudssiah Ismail, Gaetano Isola, Linda Merin J, Abhishek Jaiswal, Mihajlo Jakovljevic, Mahsa Jalili, Sung-In Jang, Chinmay T Jani, Umesh Umesh Jayarajah, Shubha Jayaram, Seyed Behzad Jazayeri, Rime Jebai, Wonjeong Jeong, Ravi Prakash Jha, Har Ashish Jindal, Yetunde O John-Akinola, Jost B Jonas, Tamas Joo, Nitin Joseph, Jacek Jerzy Jozwiak, Mikk Jürisson, Ali Kabir, Salah Eddine Oussama Kacimi, Vidya Kadashetti, Farima Farima Kahe, Vineet Kumar Kamal, Eswar Kandaswamy, Himal Kandel, HyeJung Kang, Neeti Kapoor, Sitanshu Sekhar Kar, Shama D Karanth, André Karch, Amirali Karimi, Bekalu Getnet Kassa, Patrick DMC Katoto, Joonas H Kauppila, Leila Keikavoosi-Arani, Gemechu Gemechu Kejela, Phillip M Kemp Bohan, Himanshu Khajuria, Abbas Khan, Ejaz Ahmad Khan, Gulfaraz Khan, Md Nuruzzaman Khan, Moien AB Khan, Javad Khanali, Khaled Khatab, Moawiah Mohammad Khatatbeh, Mahalaqua Nazli Khatib, Mahmoud Khodadost, Min Seo Kim, Yun Jin Kim, Adnan Kisa, Sezer Kisa, Jonathan M Kocarnik, Pavel Kolkhir, Farzad Kompani, Parvaiz A Koul, Sindhura Lakshmi Koulmane Laxminarayana, Ai Koyanagi, Kewal Krishan, Yuvaraj Krishnamoorthy, Nuworza Kugbey, Akshay Kumar, Narinder Kumar, Om P Kurmi, Carlo La Vecchia, Arista Lahiri, Judit Lám, Iván Landires, Justin J Lang, Bagher Larijani, Savita Lasrado, Jerrald Lau, Yeong Yeh Lee, Samson Mideksa Legesse, Elvynna Leong, Ayush Lohiya, Platon D Lopukhov, Mojgan Lotfi, Joana A Loureiro, Raimundas Lunevicius, Sameh Magdeldin, Soleiman Mahjoub, Ata Mahmoodpoor, Rashidul Alam Mahumud, Azeem Majeed, Konstantinos Christos Makris, Elaheh Malakan Rad, Mohammad-Reza Malekpour, Reza Malekzadeh, Ahmad Azam Malik, Tauqeer Hussain Mallhi, Sneha Deepak Mallya, Ana Laura Manda, Borhan Mansouri, Lorenzo Giovanni Mantovani, Miquel Martorell, Clara N Matei, Elezebeth Mathews, Vasundhara Mathur, Jitendra Kumar Meena, Ravi Mehrotra, Walter Mendoza, Ritesh G Menezes, Laverne G Mensah, Alexios-Fotios A Mentis, Andry Yasmid Mera Mera-Mamián, Tuomo J Meretoja, Mehari Woldemariam Merid, Belsity Temesgen Meselu, Mahboobeh Meshkat,

Tomislav Mestrovic, Tomasz Miazgowski, Irmina Maria Michalek, Ted R Miller, Shabir Ahmad Mir, Seyyedmohammadsadeq Mirmoeeni, Mohammad Mirza-Aghazadeh-Attari, Abay Sisay Misganaw, Esmaeil Mohammadi, Abdollah Mohammadian-Hafshejani, Arif Mohammed, Shafiu Mohammed, Syam Mohan, Nagabhishek Moka, Ali H Mokdad, Alex Molassiotis, Mariam Molokhia, Kaveh Momenzadeh, Sara Momtazmanesh, Lorenzo Monasta, Ahmed Al Montasir, Fateme Montazeri, Arnulfo Montero, Abdolvahab Moradi, Yousef Moradi, Mostafa Moradi Sarabi, Paula Moraga, Shane Douglas Morrison, Jakub Morze, Abbas Mosapour, Ebrahim Mostafavi, Seyyed Meysam Mousavi, Amin Mousavi Khaneghah, Francesk Mulita, Sandra B Munro, Efrén Murillo-Zamora, Christopher J L Murray, Jonah Musa, Ashraf F Nabhan, Ahamarshan Jayaraman Nagarajan, Shankar Prasad Nagaraju, Gabriele Nagel, Mukhammad David Naimzada, Tapas Sadasivan Nair, Sreenivas Narasimha Swamy, Hasan Nassereldine, Zuhair S Natto, Biswa Prakash Nayak, Sabina Onyinye Nduaguba, Wogene Wogene Negash, Seyed Aria Nejadghaderi, Sandhya Neupane Kandel, Robina Khan Niazi, Nurulamin M Noor, Maryam Noori, Ali Nowroozi, Virginia Nuñez-Samudio, Chimezie Igwegbe Nzoputam, Ogochukwu Janet Nzoputam, Bogdan Oancea, Oluwakemi Ololade Odukoya, Onome Bright Oghenetega, Ropo Ebenezer Ogunsakin, Ayodipupo Sikiru Oguntade, In-Hwan Oh, Akinkunmi Paul Okekunle, Andrew T Olagunju, Tinuke O Olagunju, Babayemi Oluwaseun Olakunde, Abidemi E Emmanuel Omonisi, Obinna E Onwujekwe, Hans Orru, Stanislav S Otstavnov, Bilcha Oumer, Babatunji Emmanuel Oyinloye, Mahesh P A, Alicia Padron-Monedero, Jagadish Rao Padubidri, Reza Pakzad, Tamás Palicz, Adrian Pana, Ashok Pandey, Shahina Pardhan, Eun-Cheol Park, Seoyeon Park, Jay Patel, Siddhartha Pati, Uttam Paudel, Hamidreza Pazoki Toroudi, Minjin Peng, Jeevan Pereira, Renato B Pereira, Simone Perna, Navaraj Perumalsamy, Richard G Pestell, Julian David Pillay, Zahra Zahid Piracha, Maarten J Postma, Akila Prashant, Mirza Muhammad Fahd Qadir, Zahiruddin Quazi Syed, Mohammad Rabiee, Navid Rabiee, Amir Radfar, Raghu Anekal Radhakrishnan, Venkatraman Radhakrishnan, Ata Rafiee, Nasiru Raheem, Fakher Rahim, Shayan Rahmani, Nazanin Rajai, Pradhum Ram, Kiana Ramezanzadeh, Kamal Ranabhat, Priyanga Ranasinghe, Chythra R Rao, Sowmya J Rao, Zubair Ahmed Ratan, David Laith Rawaf, Salman Rawaf, Lal Rawal, Andre M N Renzaho, Maryam Rezaei, Nazila Rezaei, Nima Rezaei, Saeid Rezaei, Abanoub Riad, Reza Rikhtegar, Thomas J Roberts, Esperanza Romero-Rodríguez, Gholamreza Roshandel, Godfrey M Rwegerera, Manjula S, Maha Mohamed Saber-Ayad, Bahar Saberzadeh-Ardestani, Siamak Sabour, Basema Saddik, Umar Saeed, Mohsen Safaei, Amirhossein Sahebkar, Harihar Sahoo, Mirza Rizwan Sajid, Hedayat Salari, Marwa Rashad Salem, Hamideh Salimzadeh, Yoseph Leonardo Samodra, Abdallah M Samy, Juan Sanabria, Francesco Sanmarchi, Milena M Santric-Milicevic, Fatemeh Sarvi, Maheswar Satpathy, Nicolas Sayegh, Ione Jayce Ceola Schneider, Mario Šekerija, Sadaf G Sepanlou, Allen Seylani, Kenbon Seyoum, Pritik A Shah, Saeed Shahabi, Izza Shahid, Mohammed Feyisso Shaka, Elaheh Shaker, Mohammed Shannawaz, Mequannent Melaku Sharew Sharew, Azam Sharifi, Javad Sharifi-Rad, Bereket Beyene Shashamo, Aziz Sheikh, Mahdi Sheikh, Sara Sheikhbahaei, Ali Sheikhy, Peter Robin Shepherd, Jeevan K Shetty, Kenji Shibuya, Reza Shirkoohi, K M Shivakumar, Siddharudha Shivalli, Velizar Shivarov, Parnian Shobeiri, Seyed Afshin Shorofi, Sunil Shrestha, Sudeep K Siddappa Malleshappa, Negussie Boti Sidemo, Diego Augusto Santos Silva, Luís Manuel Lopes Rodrigues Silva, Guilherme Silva Julian, Wudneh Simegn, Achintya Dinesh Singh, Garima Singh, Harpreet Singh, Jasvinder A Singh, Paramdeep Singh, Dhirendra Narain Sinha, Valentin Yurievich Skryabin, Anna Aleksandrovna Skryabina, Bogdan Socea, Ahmad Sofi-Mahmudi, Yonatan Solomon, Suhang Song, Sergey Soshnikov, Mu'awiyyah Babale Sufiyan, Ryan Suk, Muhammad Suleman, Saima Sultana, Daniel Sur, Miklós Szócska, Seidamir Pasha Tabaeian, Seyyed Mohammad Tabatabaei, Takahiro Tabuchi, Hooman Tadbiri, Majid Taheri, Iman M Talaat, Mircea Tampa, Ker-Kan Tan, Nathan Y Tat, Vivian Y Tat, Arash Tavakoli, Arash Tehrani-Banihashemi, Yohannes Tekalegn, Jansje Henny Vera Ticoalu, Mathilde Touvier, Marcos Roberto Tovani-Palone, Khanh Bao Tran, Mai Thi Ngoc Tran, Jaya Prasad Tripathy, Irfan Ullah, Saif Ullah, Bhaskaran Unnikrishnan, Marco Vacante, Sahel Valadan Tahbaz, Constantine Vardavas, Shoban Babu Varthya, Diana Zuleika Velazquez, Massimiliano Veroux, Francesco S Violante, Vasily Vlassov, Theo Vos, Linh Gia Vu, Yasir Waheed, Mandaras Tariku Walde, Richard G Wamai, Fang Wang, Ning Wang, Yu Wang, Paul Ward, Ronny Westerman, Nuwan Darshana Wickramasinghe, Lalit Yadav, Seyed Hossein Yahyazadeh Jabbari, Lin Yang, Yigizie Yeshaw, Naohiro Yonemoto, Fatemeh Yousefian,

Mazyar Zahir, Moein Zangiabadian, Iman Zare, Armin Zarrintan, Mikhail Sergeevich Zastrozhin, Mohammad A Zeineddine, Jianrong Zhang, Zhi-Jiang Zhang, Mohammad Zoladl.

Managing the estimation or publications process

Liaqat Ali, Hubert Amu, Solomon Shitu Ayen, Saber Azami-Aghdash, Melkalem Mamuye Azanaw, Ahmed Y Y Azzam, Indrajit Banerjee, Alemayehu Sayih Belay, Michael Brauer, Kelly Compton, Fikadu Nugusu Dessalegn, Syed Masudur Rahman Dewan, Fatemeh Eghbalian, Tahir Eyayu, Ali Fatehizadeh, Berhanu Elfu Feleke, Lisa M Force, Takeshi Fukumoto, Kazem Ghaffari, Sherief Ghozy, Alemu Guta, Simon I Hay, Mahsa Jalili, Abbas Khan, Rashidul Alam Mahumud, Borhan Mansouri, Mehari Woldemariam Merid, Ali H Mokdad, Christopher J L Murray, Bilcha Oumer, Mahesh P A, Ashok Pandey, Siddhartha Pati, Lal Rawal, Maryam Rezaei, Esperanza Romero-Rodríguez, Hedayat Salari, Abdallah M Samy, Negussie Boti Sidemo, Ensiyeh Taheri, Rixing Xu, Mikhail Sergeevich Zastrozhin.
